# Pseudorabies Virus UL4 protein promotes the ASC-dependent inflammasome activation and pyroptosis to exacerbate inflammation

**DOI:** 10.1371/journal.ppat.1012546

**Published:** 2024-09-24

**Authors:** Xiaohua Zhang, Guiyuan Chen, Junqing Yin, Lichen Nie, Linghao Li, Qian Du, Dewen Tong, Yong Huang

**Affiliations:** 1 College of Veterinary Medicine, Northwest A&F University, Yangling, China; 2 Engineering Research Center of Efficient New Vaccines for Animals, Ministry of Education, Yangling, China; 3 Key Laboratory of Ruminant Disease Prevention and Control (West), Ministry of Agriculture and Rural Affairs, Yangling, China; 4 Engineering Research Center of Efficient New Vaccines for Animals, Universities of Shaanxi Province, Yangling, China; University of Wisconsin-Madison, UNITED STATES OF AMERICA

## Abstract

Pseudorabies virus (PRV) infection causes systemic inflammatory responses and inflammatory damages in infected animals, which are associated with the activation of inflammasome and pyroptosis in infected tissues. Here, we identified a critical function of PRV non-structural protein UL4 that enhanced ASC-dependent inflammasome activation to promote pyroptosis. Whereas, the deficiency of viral UL4 was able to reduce ASC-dependent inflammasome activation and the occurrences of pyroptosis. Mechanistically, the 132–145 aa of UL4 permitted its translocation from the nucleus to the cytoplasm to interact with cytoplasmic ASC to promote the activation of NLRP3 and AIM2 inflammasome. Further research showed that UL4 promoted the phosphorylation levels of SYK and JNK to enhance the ASC phosphorylation, which led to the increase of ASC oligomerization, thus promoting the activation of NLRP3 and AIM2 inflammasome and enhanced GSDMD-mediated pyroptosis. In *vivo* experiments further showed that PRV UL4 (^132^DVAADAAAEAAAAE^145^) mutated strain (PRV-UL4^mut^) infection did not lead to a significant decrease in viral titers at 12 h. p. i, but it induced lower levels of IL-1β, IL-18, and GSDMD-NT, which led to an alleviated inflammatory infiltration and pathological damage in the lungs and brains, and a lower death rate compared with wild-type PRV strain infection. Taken together, our findings unravel that UL4 is an important viral regulator to manipulate the inflammasome signaling and pyroptosis of host cells to promote the pathogenicity of PRV, which might be further exploited as a new target for live attenuated vaccines or therapeutic strategies against pseudorabies in the future.

## Author summary

The activation of inflammasome and pyroptosis is associated with the rapid death of mice infected with PRV. However, the specific molecular mechanism by which PRV promotes inflammasome overactivation remains elusive. Here, we identified a critical function of PRV non-structural protein UL4 that enhanced ASC-dependent inflammasome activation to promote pyroptosis. Whereas viral UL4 deficiency reduced ASC-dependent inflammasome activation and pyroptosis. Mechanistically, the 132–145 aa of UL4 protein permitted its translocation to interact with ASC in the cytoplasm. During this process, UL4 promoted the phosphorylation levels of JNK and SYK, which enhanced the ASC phosphorylation, leading to increased ASC oligomerization, and ultimately enhanced the ASC-dependent inflammasome activation and subsequent pyropKtosis. In *vivo*, compared with PRV-WT infection, the PRV *UL4* gene (^132^DVAADAAAEAAAAE^145^) mutated strain (PRV-UL4^mut^) not only delayed the death time of pigs and mice but also alleviated the inflammatory infiltration and pathological damage in the lung and brain tissues. Altogether, our findings unravel UL4 as an important regulator of host immune response by manipulating the inflammasome signaling, which may be regarded as a novel live gene-deleted PRV vaccine candidate.

## 1. Introduction

Pseudorabies (PR) is a severe disease that mainly affects pigs and incidentally affects many other mammalian species, resulting in enormous economic losses worldwide, which is caused by pseudorabies virus (PRV) [1,2]. PRV belongs to the subfamily *Alphaherpesviridae*, which possesses a double-stranded DNA genome of about 140 kb consisting of short and long unique regions (US, UL) and inverted repeat sequences (IRS, TRS) flanking the US region [3]. The viral genome contains at least 70 open reading frames (ORFs), which encodes three subsets known as immediate-early (IE), early (E), and late (L) proteins depending on the sequential expression of the corresponding genes [4].

PRV UL4 is a late non-structural protein that localizes in the cytoplasm and small nuclear structures of infected cells, whose function is largely unknown. Homologs of the UL4 are present in other members of the *Alphaherpesviruses*, such as the herpes simplex virus type I (HSV-1), the bovine herpesvirus-1 (BHV-1), and varicella-zoster virus [5]. UL4 is not essential for PRV replication, while it might enhance the virion release [6,7]. However, the underlying mechanism by which UL4 mediates virion release remains to be elucidated.

PRV infection generates an inflammatory response in infected cells as well as inflammatory damage to infected tissues, such as encephalitis, and interstitial pneumonia [8,9]. Inflammasome is closely associated with the inflammatory response and can be activated during PRV infection [10,11]. After recognizing PAMPs or DAMPs, inflammasome assemble, which include the inflammasome-initiating sensor (NLRP1, NLRP3, NLRC4, AIM2 or pyrin, etc.), and inflammasome effector pro-CASP1, in the presence or absence of the inflammasome adaptor apoptosis-associated speck-like protein containing a CASP1 recruitment domain (ASC). When the ASC-dependent inflammasome is activated, it causes ASC speck formation as a platform to efficiently activate pro-CASP1, leading to the maturation of Pro-IL-1β and pro-IL-18, and the cleavage of the GSDMD, which drives GSDMD-mediated pyroptosis, a form of inflammatory cell death [12–14]. Inflammasome and pyroptosis are essential for innate immunity against infections, whereas overactivation of inflammasome or excessive pyroptosis, leads to several inflammatory diseases, including sepsis and autoimmune disorders [15,16]. For example, activated inflammasome upon SARS-CoV-2 infection is associated with COVID-19 severity [17,18]. The inflammasome is subjected to precise manipulation and regulated at multiple levels, with one level being posttranslational modification (PTM) of a variety of components of the inflammasome. Among different types of modifications, phosphorylation, and ubiquitination are known to play vital roles [19–23]. For example, murine ASC phosphorylation at tyrosine (Y) 144 (equivalent to human Y146) by SYK and JNK promotes the assembly of NLRP3 and AIM2 inflammasome [20], whereas phosphorylation events at S16, S193, Y60, and Y137 of murine ASC are all inhibitory for inflammasome activation [24].

Previous studies have found that PRV can activate NLRP3, AIM2, and IFI16 inflammasome [9,11,25]. However, the specific molecular mechanism by which PRV promotes inflammasome activation remains elusive. This study attempts to screen a key PRV protein that regulates the activation of inflammasome, to achieve the possibility of controlling the inflammatory process. In this study, we first screened that PRV UL4 protein promoted the activation of NLRP3 and AIM2 inflammasome and pyroptosis. At the same time, the PRV-*Δ*UL4 was constructed to clarify the UL4 effect on pyroptosis and inflammasome activation during infection. Subsequently, the specific mechanism and the key site of UL4 regulating inflammasome activation and pyroptosis were identified. The mutant PRV strain of this site was further constructed. Finally, the mutant PRV strain was used to inoculate animals to verify its role and mechanism in the pathogenesis of PRV infection. The obtained data will help to improve the understanding of the relationship between PRV and inflammasome, providing a deeper knowledge of the pathogenic mechanisms implicated in this disease.

## 2. Materials and methods

### Ethics statement

All animal experimental operations were performed in strict accordance with the recommendations in the Guide for the Care and Use of Laboratory Animals of the Ministry of Science and Technology of the People’s Republic of China. The animal experiments with mice and pigs obtained the support of the Institutional Animal Care and Use Committee (IACUC) of Northwest A&F University (permit number XN2023-0602).

### Cells and virus

Human embryonic kidney 293T (HEK293T) cells were propagated in Dulbecco’s minimum essential medium (DMEM) (12100–046; Invitrogen) supplemented with 10% heat-inactivated fetal bovine serum (FBS) (13011–8611; Tianhang Biotechnology), 5% CO_2_ at 37°C. Porcine alveolar macrophages (3D4/21, ATCC, CRL-2843) were cultured with RPMI 1640 (A4192301, Gibco) supplemented with 10% FBS, and 5% CO_2_ at 37°C. PRV strain (OR137576), HSV-1 strain (OP297886.1), and BHV-1(MT179818.1) were all stocked in our lab, PRV-*Δ*UL4, PRV-UL4^repair^ and PRV-UL4^mut^ were constructed in this research.

### Primary cell culture

Bone marrow-derived macrophages (BMDMs) were prepared as described below. Briefly, total bone marrow cells isolated from mouse femurs and tibias were resuspended in a culture medium (DMEM supplemented with 10% FBS, 100 U/mL penicillin/ streptomycin, and 30 ng/mL recombinant mouse M-CSF). Cells were inoculated at 1.5 × 10^6^/well in a 6-well plate with 2 mL culture medium per well and cultured for 6 days. After 3 days, the medium was changed, and floating non-adherent cells were washed away. Four to five million cells per mouse were routinely obtained. Microscopic examination confirmed that >95% of adherent cells were morphologically mature macrophages.

### Antibodies and reagents

The antibody anti-β-actin (A00702) was from Genscript; anti-NLRP3 (19771-1-AP), ASC (10500-1-AP), anti-p-JNK (Tyr185) (80024-1-RR), anti-JNK (66210-1-Ig) anti-SYK (66721-1-Ig) were provided by Proteintech; anti-p-SYK (Tyr525/526) (2710) and anti-AIM2 (53491) were obtained from Cell Signaling Technology; anti-IL-1β (A19635) was from Abclonal; anti-caspase-1 p10 (sc-514) and anti-Phosphotyrosine (sc-508) were from Santa Cruz; anti-GFP (YM3124), anti-HA (YM3003), anti-Flag (YM3808), Goat Anti Mouse IgG (H&L)-Alexa Fluor 594 (RS3608) and Goat Anti Rabbit IgG (H&L)-Alexa Fluor 488 (RS3211) were from Immunoway; p-ASC Y144 pAb (AP5631) was from ECM Biosciences; Horseradish peroxidase (HRP)-conjugated anti-mouse IgG (31430) and anti-rabbit IgG (31460) were provided by Invitrogen; Anti-UL4 of PRV polyclonal antibodies were produced in our lab. Protein G-agarose (sc-2002) and protein A-agarose (sc-2001) were purchased from Santa Cruz; the LDH Cytotoxicity Assay Kit was purchased from Beyotime (C0016). Porcine IL-1β ELISA KIT (SEKP-0001), Porcine IL-18 ELISA KIT (SEKP-0353), Mouse IL-18 ELISA KIT (SEKM-0019), and Mouse IL-1β ELISA KIT (SEKM-0002) were obtained from Solarbio; Nigericin (NG) (HY-127019), R406 (HY-12067), PRT062607 and Hydrochloride (HY-15323) were provided by MCE, lipopolysaccharide (LPS) (l2630), ATP (A1852) and Poly dA:dT (P0883) were from Sigma-Aldrich; Polyplus-transfection was from poly plus.

### Plasmid construction

Total RNA was extracted from PAMs or BMDMs stimulated with LPS and ATP, and then cDNA was synthesized using the reverse transcription kit (Vazyme, R201-01/02). The full-coding sequence (CDS) of porcine NLRP3, ASC, Pro-caspase-1, Pro-IL-1β, and murine AIM2, ASC, Pro-caspase-1, Pro-IL-1β were synthesized and were cloned into pCI-neo vector with the Flag tag sequence or HA tag sequence (Sangon Biotech). The CDS of PRV indicated proteins were amplified and cloned into the pEGFP-N1. The CDS of PRV full-length-UL4, UL4^mut^ truncated, or mutant UL4 was cloned into pEGFP-N1. The CDS of PRV full-length-UL4 was also cloned into the pCMV-HA vector. The CDS of HSV-1 UL4 and BHV-1 UL4 was cloned into pEGFP-N1. The sequence fidelity of all created was confirmed by DNA sequencing (Sangon Biotech). The HA-tagged ubiquitination plasmid (HA-Ub) was donated by Dr. Rui Zhang. The ubiquitination plasmids (K63 Ubiquitin-17606) were from addgene.

### Enzyme-linked immunosorbent assay (ELISA)

The concentrations of IL-1β and IL-18 in cell culture supernatant and the serum or tissues were measured by using ELISA kits according to the manufacturer’s instructions.

### Cell viability assay

Lactate dehydrogenase (LDH) assays were performed to determine cell viability. The indicated cell culture supernatant was harvested and cleared by centrifugation at 2,000 × g for 5 min, and 50 μL was used to perform the LDH assay according to the manufacturer’s instructions.

### NLRP3 inflammasome reconstitution assay

293T cells were seeded at 1.8 × 10^5^ cells per well on 24-well plates and transfected with pCI-Flag-NLRP3, pCI -Flag-ASC, pCI-Flag-pro-CASP1, and pCI-Flag-Pro-IL-1β, along with pEGFP-N1 or expression vector of viral indicated protein. At 24 h posttransfection, cells were treated with LPS/NG or LPS/ATP. Then, cell-free supernatants were harvested for IL-1β ELISA or expression on CASP1 (p10) and IL-1β (p17) proteins, and cells were lysed for western blotting.

### AIM2 inflammasome reconstitution assay

293T cells were seeded on 24-well plates and transfected with pCI- Flag-AIM2, pCI-Flag-ASC, pCI-Flag-Pro-caspase-1, and pCI-Flag-Pro-IL-1β, along with pEGFP-N1 or expression vector of viral indicated protein. At 24 h posttransfection, cells were then transfected with Poly dA:dT. Then, cell-free supernatants were harvested for murine IL-1β ELISA or expression on p17 and p10 proteins detection, and cells were lysed for western blotting.

### Immunoprecipitation assay

293T cells were seeded into 10-cm diameter dishes and transfected with indicated plasmids or 3D4/21 cells were infected with indicated virus (5 MOI) 12 h, then, the cells were lysed with lysis buffer as described before [26]. Next, protein G/A agarose beads were added to the cell lysate supernatant for 1 h at 4°C to pre-clean the nonspecific proteins. After pre-clean, the supernatant was incubated with the indicated antibodies, overnight at 4°C, followed by adding the protein G/A agarose beads for 2 h at room temperature. The beads were collected by centrifuging at 2,000 × g for 10 s and washed three times with PBS. Finally, the proteins bound to the beads were eluted for western blotting analysis.

### The construction of the PRV-*Δ*UL4, PRV-UL4^repair^, and PRV-UL4^mut^

Construction of the knockout (KO) plasmids used the pX335 plasmid vector (purchased from Addgene, MA, USA, digest with *Bsm* I (purchased from Thermo Fisher Scientific, MA, USA) and sgRNA sequences targeting the PRV *UL4* genes or *GFP* genes according to the website (http://crispr.mit.edu/), After transformation into *E*. *coli Stbl3*, the recombinant plasmids were isolated and verified by sequencing. The donor plasmids pCI-neo-UL4-UP-GFP-UL4-down, pCI-neo-GFP-UP-UL4-GFP-down or pCI-neo-GFP-UP-UL4^mut^-GFP-down was constructed using homologous recombinase. PRV DNA was used as the template to amplify the left and right homology arms of PRV *UL4*; pEGFP-N1-UL4 or the pEGFP-N1-UL4^mut^ was used as the template to amplify the UL4 or UL4^mut^. pEGFP-C1 plasmid was used as the template to amplify the *GFP*. To generate PRV-*Δ*UL4, the KO plasmids targeting PRV UL4 and donor plasmids with the homologous recombinant fragment were transfected into PK-15 cells. 24 h after transfection, PRV YL-2020 was added. The virus culture was harvested when a green fluorescent lesion was observed. Five rounds of plaque purification were performed in Vero cells to obtain pseudorabies UL4 gene deletion strain PRV-*Δ*UL4. To generate recombinant PRV (PRV-UL4^mut^ or PRV-UL4), the KO plasmids targeting GFP and donor plasmids with the homologous recombinant fragment were co-transfected into PK-15 cells. 24 h after transfection, PRV-*Δ*UL4 was added. The virus culture was harvested when a lesion without green fluorescent was observed. Finally, the recombinant PRV (PRV-UL4^mut^ or PRV-UL4) strain expressing UL4^mut^ or UL4 was amplified and purified in Vero cells by five rounds of phagocytosis. The sgRNA-induced mutation was determined by PCR and sequencing, and the UL4 or GFP expression was by western blotting.

### Western blotting analysis

Cells were lysed with lysis buffer supplemented with protease inhibitor mixture. Cell lysates or IP samples were fractionated by SDS-polyacrylamide gel electrophoresis (PAGE) and were blotted onto PVDF membranes, which were then blocked with PBS containing 5% nonfat dry milk, followed by incubation with the indicated primary antibody. After incubation with the secondary antibodies at room temperature for 1 h, the membrane was added Enhanced Chemiluminescence Substrate (Bio-Rad, Hercules, CA, USA) to develop color for observation of the protein expression. Western blotting of the protein in cell culture supernatant: The trichloroacetic acid was pre-cooled to -20°C, and mixed with the stored cell culture supernatants in a volume of 4: 1 on ice. Then, the mixture was placed at -20°C overnight, and centrifuged to collect the protein precipitation. Next, protein precipitation was prepared as the western blotting loading sample for detection.

### Immunofluorescence microscopy

Cells were fixed with 4% paraformaldehyde, then permeabilized with 0.1% TritonX-100, followed by indirect immunofluorescence detection using corresponding antibodies; nucleic acid was stained with 4,6-diamidino-2-phenylindole (DAPI). The stained samples were photographed by a Leica TCS SP8 laser scanning confocal microscope.

### ASC oligomerization detection

3D4/21 cells and BMDMs were transfected with indicated plasmids for 24 h or infected with the indicated PRV (5 MOI) for 12 h, then cells were treated with LPS/NG or Poly dA:dT. Cell lysates were centrifugated at 12, 000 rpm for 15 min. The supernatants of the lysates were mixed with SDS loading buffer for western blotting analysis with antibodies against ASC. The pellets of the lysates were washed with PBS and cross-linked using fresh DSS (4 mM, Sigma) at 37°C for 30 min. The cross-linked pellets were then spun down and the supernatant was mixed with SDS loading buffer for western blotting analysis.

### Animal experiments

Six-week-old specific-pathogen-free (SPF) C57BL/6 mice were purchased from Chengdu Dossy Experimental Animals Co., Ltd. (Chengdu, China). All mice were generated and housed in SPF barrier facilities at the Northwest Agriculture and Forestry University (NWAFU). Three-week-old cross-bred piglets were purchased from a native herd free of PCV2, PRV, porcine parvovirus, and other major swine pathogens as determined by PCR. All piglets were housed under the same conditions and treated similarly. All animal experiments were performed according to animal protocols approved by the Subcommittee on Research Animal Care at the NWAFU. Mice were randomly divided into three groups and inoculated with PRV (10^6^ TCID_50_), PRV-UL4^mut^ or Mock (same volume of DMEM) respectively. Pigs were randomly divided into three groups and inoculated with PRV (10^8^ TCID_50_), PRV-UL4^mut^ or Mock (same volume of DMEM) respectively. Survival and clinical symptoms were monitored daily. The peripheral blood and indicated tissues were collected at indicated days post-infection. The brains and lungs were removed and divided into two hemispheres; one portion was fixed in 10% formaldehyde, embedded in paraffin, and sectioned for hematoxylin and eosin (HE) [26,27]. The other portions were dissected and snap-frozen at −80°C. For the determination of cytokines DNA copies and indicated protein, individual tissue samples were homogenized and lysed as mentioned above. The samples were harvested and measured for cytokine concentrations by ELISA. Virus copy detection using plaque assay.

### Statistical analysis

The Data were analyzed using GraphPad Prism v8.0 software. Comparisons between the two groups were performed by unpaired t-test, while data of multiple groups were analyzed by One-way ANOVA and Bonferroni post-hoc test. Data are presented as mean ± SEM of biological replicates or typical photographs and are representative of three independent experiments.

## 3. Results

### 3.1 PRV UL4 facilitates the activation of NLRP3 inflammasome

**Fig 1:**
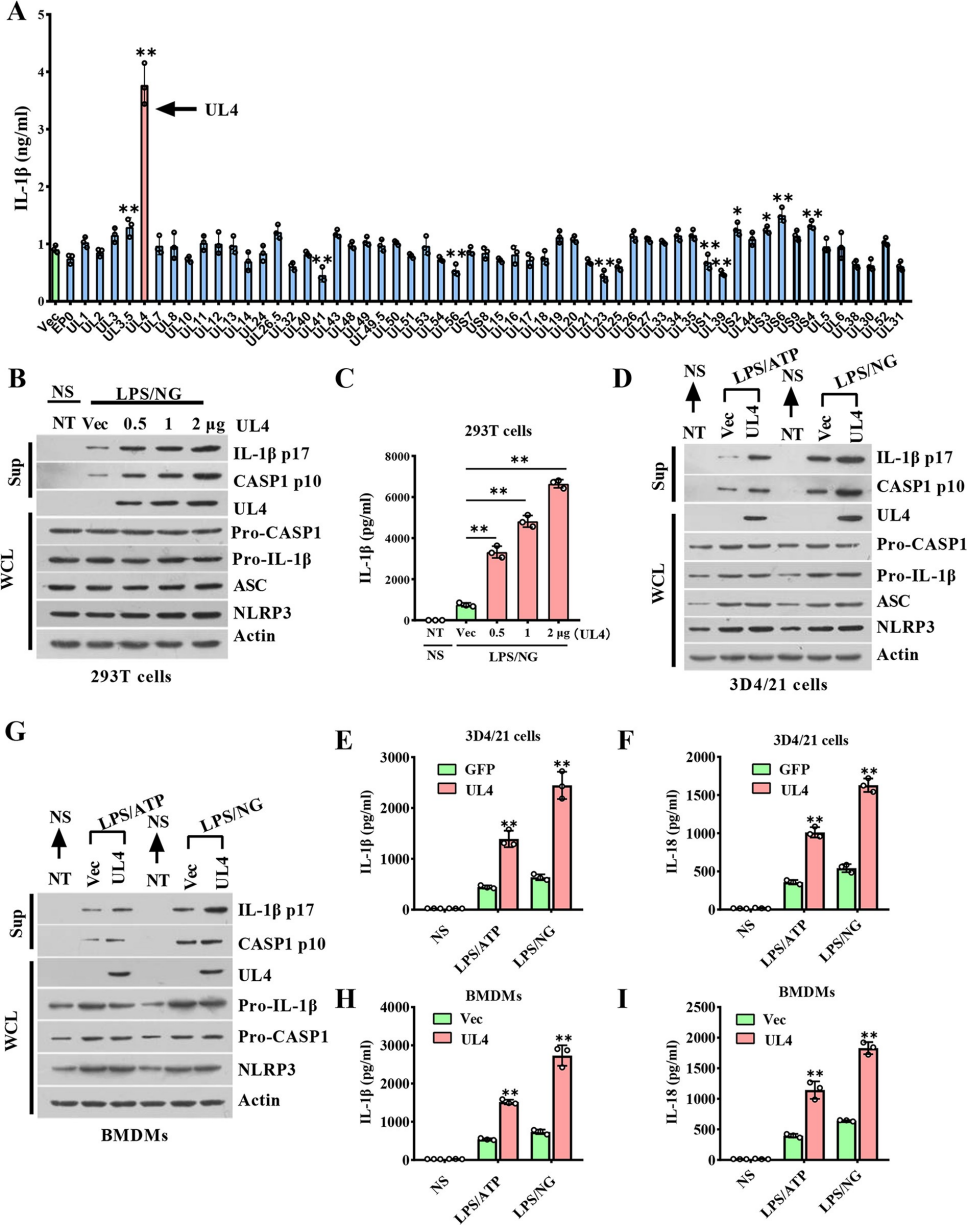
PRV UL4 is a promotor of NLRP3 inflammasome Activation. **(A)** ELISA was used to detect the effect of viral protein on IL-1β secretion in 293T cells reconstructed with NLRP3 inflammasome system. 293T cells were co-transfected with NLRP3 inflammasome system-associated plasmids encoding porcine NLRP3, ASC, procaspase-1, Pro-IL-1β, along with the plasmids encoding the indicated viral protein or empty vector (pEGFP-N1) plasmids for 24 h, then the cells were treated with LPS (1mg/mL) for 1 h and further stimulated with nigericin (2 μM) for another 6 h. **(B, C)** The role of UL4 on NLRP3 inflammasome system reconstructed in 293T cells. 293T cells were co-transfected with NLRP3 inflammasome system-associated plasmids, along with plasmids expressing different concentrations of GFP-UL4, or pEGFP-N1 (Vec) or without transfection (NT) for 24 h. Then, the cells were stimulated without (NS) or with LPS/NG. **(D-I)** The role of UL4 on NLRP3 inflammasome in 3D4/21 cells or BMDMs. 3D4/21 cells (D-F) or BMDMs (H-G) were transfected with plasmids encoding GFP-UL4 or Vec for 24 h, the cells were stimulated without (NS) or with LPS/NG or LPS/ATP. Precipitated conditioned media (Sup) and whole cell lysates (WCL) were analyzed by immunoblotting to detect expression levels of the indicated proteins (B, D, G), and the cell-free supernatants were subjected to ELISA for the secretion of IL-1β (C, E, H) and IL-18 (F, I). * *P* < 0.05; ** *P* < 0.01, compared with the Vec transfected cells with the same treatment.

To clarify which protein of PRV can regulate NLRP3 inflammasome activation, a cell system used to detect NLRP3 activation and IL-1β cleavage was established, in which 293T cells that lack the expression of endogenous inflammasome proteins were co-transfected with plasmids expressing NLRP3, ASC, pro-CASP1, and Pro-IL-1β. Meanwhile, we employed a widely used two-signal model of inflammasome activation, involving priming cells with LPS followed by stimulation with an activator of the NLRP3 inflammasome (ATP or Nigericin/NG). Using this system, we tested the effects of 56 viral proteins of PRV on NLRP3 inflammasome activation via transfection with the expression plasmid encoding each protein of PRV. Western blotting showed the expression of the indicated proteins (S1A Fig). As shown in Fig 1A, transfection with the expression plasmid encoding UL4, a PRV non-structural protein, preferentially promoted IL-1β secretion and NLRP3-mediated inflammasome activation compared with empty plasmid expression and other viral protein expression. Consistent with this finding, in response to LPS plus NG (LPS/NG) stimulation, more mature CASP1 (p10) and IL-1β (p17) proteins, and IL-1β secretion in the NLRP3 inflammasome reconstitution system were up-regulated by UL4 in a dose-dependent manner (Fig 1B and 1C). In addition, compared to the EP0-expressing cells, the production of p10 and p17 proteins were elevated in the UL4-expressing NLRP3 inflammasome reconstitution system reconstitution system (S1B Fig). These findings suggested that PRV UL4 protein might possess a function to promote the activation of NLRP3 inflammasome and IL-1β secretion. Next, we further tested the effects of UL4 on NLRP3 inflammasome activation in 3D4/21 and BMDMs. Consistently, compared to the empty vector-transfected cells, the production of p10 and p17 proteins, along with the secretion of IL-1β and IL-18 were elevated in the UL4-expressed-3D4/21 cells and UL4-expressed-BMDMs supernatant in response to LPS/NG or LPS /ATP stimulation (Fig 1D–1I). Notably, the levels of pro-CASP1 and Pro-IL-1β were not affected by UL4 expression in these cells (Fig 1D and 1G), suggesting that UL4 might play a role in mediating NLRP3 inflammasome activation instead of priming. Taken together, these data suggest that UL4 might enhance NLRP3 inflammasome activation to promote IL-1β and IL-18 production.

### 3.2 PRV UL4 also enhances the activation of AIM2 inflammasome

**Fig 2:**
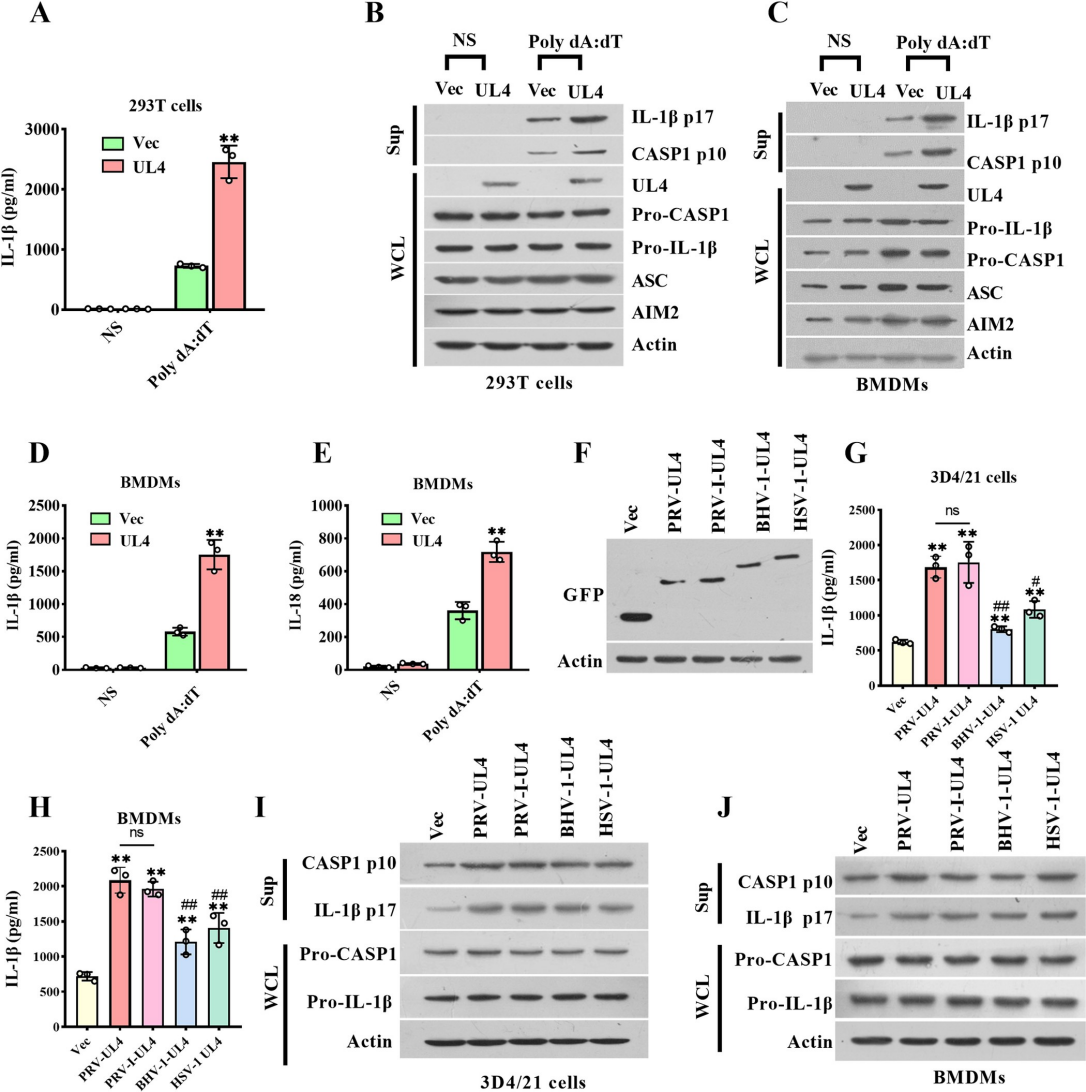
PRV UL4 also promotes AIM2-inflammasome activation. **(A, B)** The role of UL4 on the activation of AIM2 inflammasome reconstructed in 293T cells. 293T cells were transfected with AIM2 inflammasome system-associated plasmids expressing murine AIM2, ASC, pro-CASP1, Pro-IL-1β, along with plasmids encoding GFP-UL4 or GFP (Vec) for 24 h, then transfected without (NS) or with or Poly dA:dT (2 μM). **(C-E)** The role of UL4 on the activation of AIM2 inflammasome in BMDMs. BMDMs were transfected with plasmids expressing GFP-UL4 or GFP (Vec) for 24 h, then transfected without (NS) or with Poly dA:dT. **(F-J)** The effect of the different viral UL4 on inflammasome. The expression of the indicated viral UL4 protein was measured by western blotting (F). The effect of the different viral UL4 on NLRP3 inflammasome (G, I) or AIM2 inflammasome (H, J). 3D4/21 cells (G, I) and BMDMs (H, J) transfected with plasmids expressing the indicated viral UL4 protein for 24 h, followed by the stimulation of LPS/NG (G, I) or Poly dA:dT (H, I). Supernatants were subjected to ELISA for IL-1β secretion (A, D, G, H) or IL-18 secretion (E). Sup and WCL were analyzed by immunoblotting to detect the indicated proteins (B, C, I, J). ** *P* < 0.01, compared with the empty vector-expressed cells with the same treatment (A, D, E, G, H); ^#^ *P* < 0.05, ^##^ *P* < 0.01, versus the PRV-WT-infected cells (G, H).

It has been reported that the AIM2 inflammasome was also activated during PRV infection [25], thus we further determined the effect of PRV UL4 on its activation with an AIM2 inflammasome reconstitution model. In this system, UL4-exogenous expression 293T cells secreted a profoundly higher level of IL-1β in response to poly dA:dT, furthermore, the productions of CASP1 (p10) and IL-1β (p17) proteins were also facilitated by UL4, while the Pro-IL-1β and pro-CASP1 were not changed (Fig 2A and 2B). Simultaneously, the BMDMs expressed UL4 secreted more IL-1β and IL-18 and produced more p10 and p17 than the BMDMs without UL4 expression in response to Poly dA:dT treatment, but the levels of Pro-caspase-1 and Pro-IL-1β did not show difference in these cells (Fig 2C–2E). These results indicate that in addition to enhancing the activation of the NLRP3 inflammasome, PRV UL4 also enhances the activation of AIM2 inflammasome.

Since PRV belongs to the *Alphaherpesvirinae* subfamily of *Herpesvirus*, we further constructed UL4 eukaryotic expression vectors of other members of *Alphaherpesvirinae*, including HSV-1, BHV-1 and PRV genotype I to verify the effect of UL4 expression on NLRP3 and AIM2 inflammasome activation. The expression of the different viral UL4 proteins was shown in Fig 2F. Interestingly, the GFP-tagged UL4 homologs all promoted the IL-1β secretion, Pro-caspase-1 cleavage, and Pro-IL-1β maturation in both LPS/NG-stimulated 3D4/21 cells supernatants and Poly dA:dT-stimulated BMDMs supernatants (Fig 2G–2J). These results indicate that the promoted function of UL4 on NLRP3 and AIM2 inflammasome activation is partially conserved in the *Alphaherpesvirinae* homologs.

### 3.3 The UL4 null mutation alleviates inflammasome activation in PRV-infected cells

**Fig 3:**
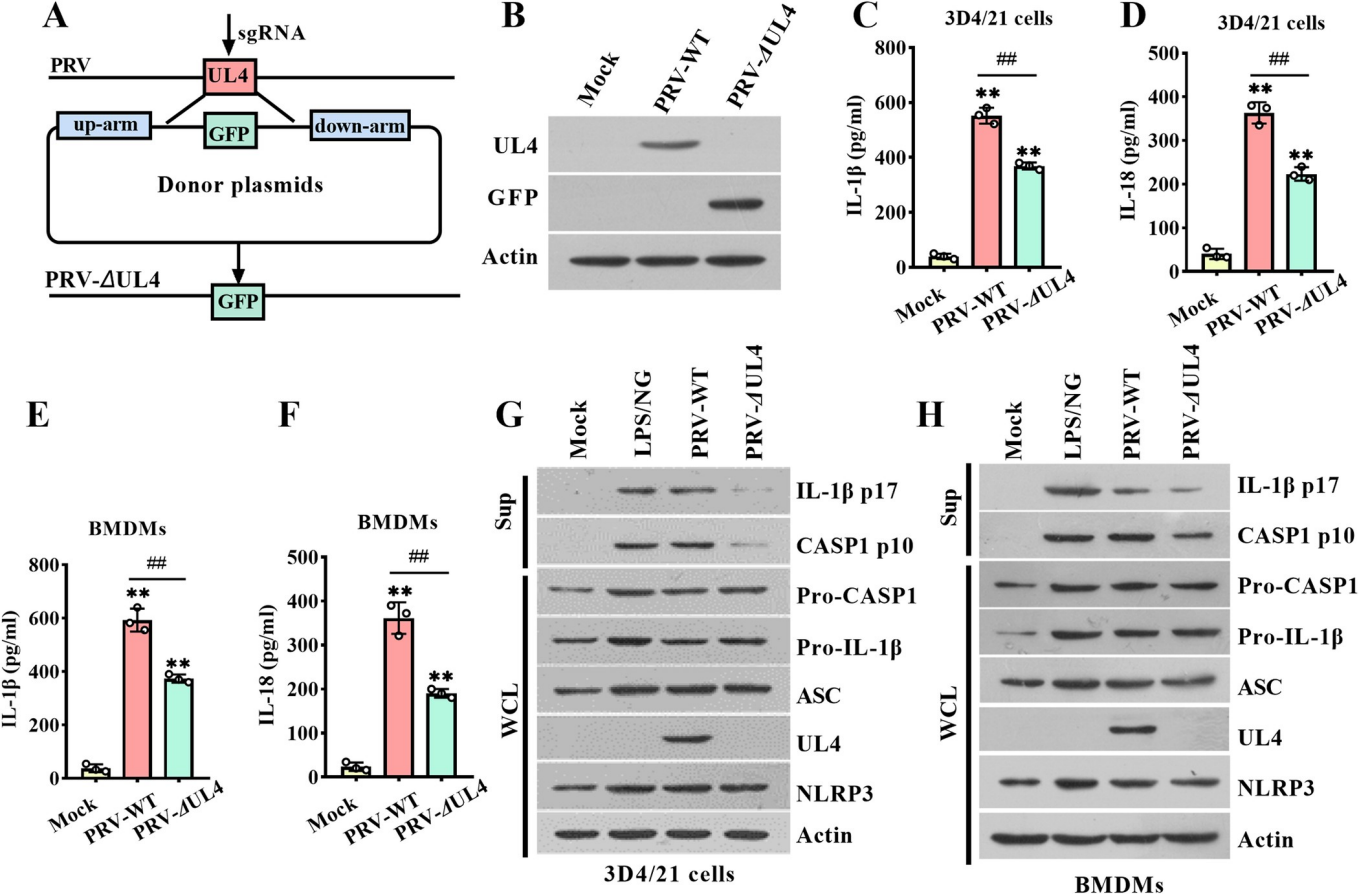
The UL4 null mutation alleviates inflammasome activation in PRV-infected cells. **(A, B)** Construction and identification of PRV-*Δ*UL4. The construction flowchart of *UL4* gene deletion virus strain (A). 3D4/21 cells were infected with Mock, PRV-WT, or PRV-*Δ*UL4 (5 MOI) for 12 h, then the expression of UL4 and GFP was detected by immunoblotting (B). **(C-H)** The effects of UL4 null mutation on the secretion of IL-1β and IL-18 in PRV-infected cells. 3D4/21 cells (C, D) or BMDMs **(**E, F) were infected with Mock, PRV-WT, or PRV-*Δ*UL4 (5 MOI) for 12 h. or treated with LPS/NG as the positive control. ELISA assay for IL-1β (C, E) and IL-18 (D, F) in supernatants was measured, and Sup and WCL were analyzed by immunoblotting for indicated protein (G, H). ** *P* < 0.01, compared with the Mock-infected cells (C-F); ^##^ *P* < 0.01, versus the PRV-WT-infected cells (C-F).

To investigate whether the production of UL4 after virus infection can affect NLRP3 inflammasome activation, we constructed a UL4 gene deletion strain (PRV-*Δ*UL4) by homologous recombination using the CRISPR/Cas9 technology. In the recombinant virus strain construction process as shown in Fig 3A, the expression of GFP was driven by the CMV promoter, and the recombinant fragment was inserted into the ORF of the PRV *UL4* gene to construct PRV-*Δ*UL4, in which the *UL4* gene was disabled. The PCR amplification results showed that wild-type PRV (PRV-WT) DNA was successfully amplified *UL4*, but not *GFP*, PRV-*Δ*UL4 DNA just did the opposite (S2A Fig). Similarly, western blotting analysis showed GFP but not UL4 expression was detectable in PRV-*Δ*UL4-infected cells in contrast to PRV-infected cells (Fig 3B), sequencing results were consistent with this (S2B Fig). Then, we examined the effect of UL4 deficiency on the inflammasome-mediated secretion of IL-1β and IL-18 during PRV infection in 3D4/21 cells and BMDMs. ELISA assays showed that, compared to the wild-type PRV infection, PRV-*Δ*UL4 infection secreted a profoundly lower level of IL-1β and IL-18 in 3D4/21 cells and BMDMs (Fig 3C–3F). Western blotting analysis also showed that UL4 deficiency impaired the generation of the IL-1β (p17) and CASP1 (p10) in 3D4/21 cells and BMDMs, while it unaffected the expression of pro-CASP1 or Pro-IL-1β (Fig 3G and 3H). There was no obviously difference in viral EP0 expression between the PRV-WT and the PRV-*Δ*UL4 in 3D4/21 cells and BMDMs at 6 and 12 h.p.i, while the viral EP0 expression in PRV-*Δ*UL4 infected cells was reduced compared to the PRV-WT infected cells at 24 h.p.i (S2C and S2D Fig). Moreover, there was no significant difference in copy numbers between the PRV and the PRV-*Δ*UL4 in 3D4/21 cells and BMDMs at 12 h.p.i (S2E and S2F Fig), which ruled out the differences due to different amounts of virus. In addition, we created a PRV-UL4^repair^ to show that the UL4-repair virus behaved like WT in growth kinetics BMDMs (S3C and S3D Fig) and inflammasome activation in the 3D4/21 and BMDMs (S2G–S2L Fig) at 12 h.p.i. These data further confirm that UL4 can enhance inflammasome activation to promote IL-1β and IL-18 production.

### 3.4 PRV UL4 promotes the pyroptosis of macrophage cells

**Fig 4:**
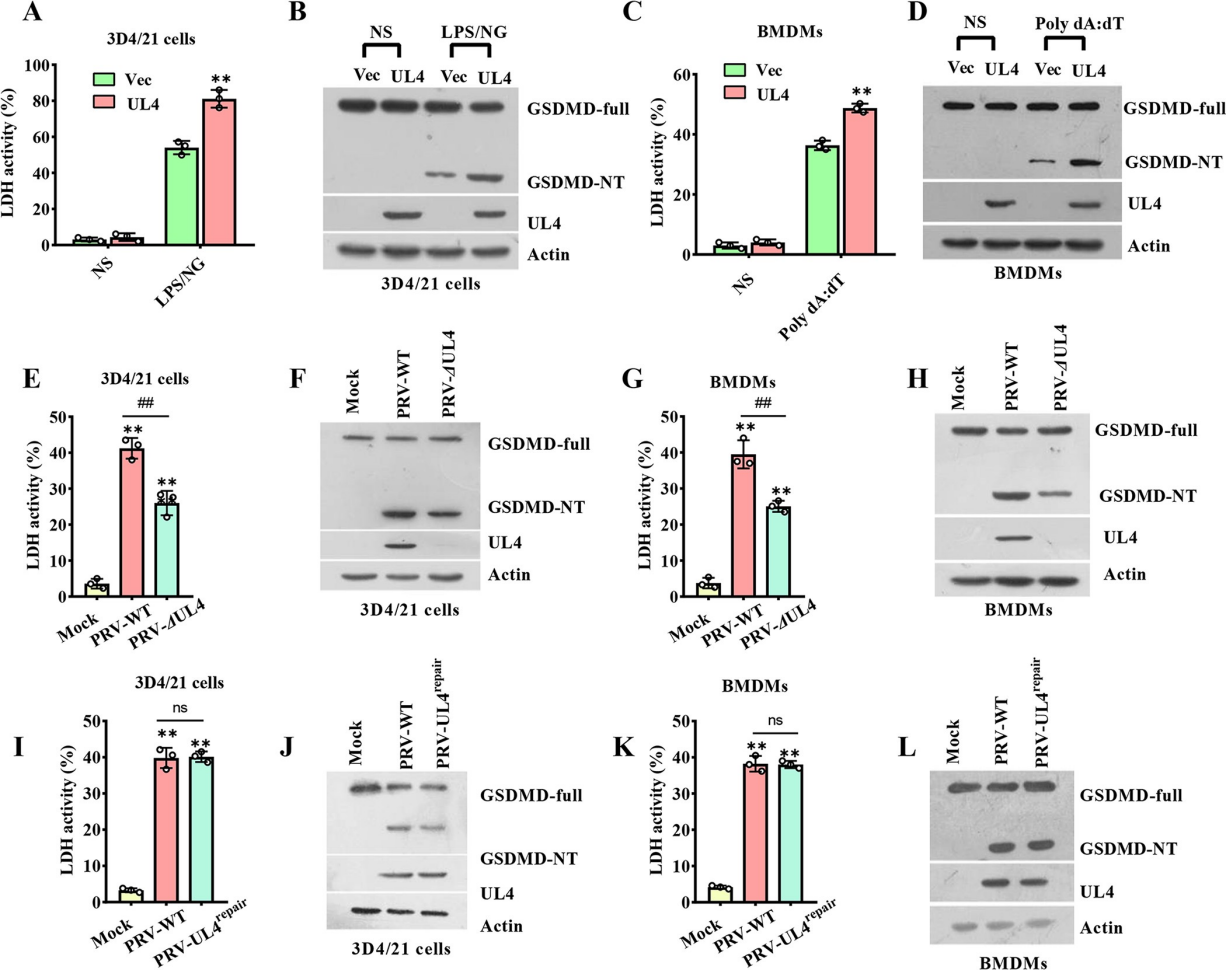
Viral UL4 protein promotes pyroptosis in macrophage cells. **(A-D)** Effect of UL4 on cell pyroptosis. 3D4/21 (A, B) or BMDMs (C, D) were transfected with plasmids encoding GFP-UL4 or GFP for 24 h, then the 3D4/21 cells were treated without (NS) or with LPS/NG; the BMDMs were treated without (NS) or with the Poly dA:dT. **(E-H)** Effect of UL4 deletion on cell pyroptosis. 3D4/21 cells (E, F) or BMDMs (G, H) were infected with Mock, PRV-WT, PRV-*Δ*UL4 (5 MOI) for 12 h. **(I-L)** Effect of UL4 repair on cell pyroptosis. 3D4/21 cells (I, J) or BMDMs (K, L) were infected with Mock, PRV-WT, and PRV-UL4^repair^ (5 MOI) for 12 h. Supernatants were collected to analyze the LDH activity (A, C, E, G, I, K); cell lysates were analyzed by immunoblotting to detect the indicated proteins (B, D, F, H, J, L). ** *P* < 0.01, compared with the Vec transfected cells (A, C) or the Mock-infected cells (E, G, I, K). ^##^ *P* < 0.01, compared with the PRV-WT infected cells with the same treatment (E, G, I, K).

Since inflammasome activation is associated with pyroptosis, we assessed the role of UL4 in regulating pyroptosis based on the release of a cytoplasmic enzyme, lactate dehydrogenase (LDH), and the cleavage of GSDMD. Indeed, UL4 expression significantly promoted the release of LDH in 3D4/21 cells and BMDMs stimulated by LPS/NG (Fig 4A). Consistent with this, the amount of the cleaved GSDMD (GSDMD-NT) was also increased in LPS/NG-stimulated 3D4/21 cells due to the expression of UL4 (Fig 4B). Similarly, the release of LDH (Fig 4C), the GSDMD-NT (Fig 4D), were both elevated in UL4-expressed BMDMs than that in BMDMs without UL4 expression when stimulated by Poly dA:dT. On the contrary, compared to the PRV infection, PRV-*Δ*UL4 infection induced less LDH (Fig 4E and 4G) and the GSDMD-NT amounts (Fig 4F and 4H). In addition, the LDH (Fig 4I and 4K) and the GSDMD-NT amounts (Fig 4J and 4L) after PRV-UL4^repair^ infection behaved like the PRV infection. These findings reveal that UL4 promotes pyroptosis in macrophage cells.

### 3.5 UL4 binds with ASC

**Fig 5:**
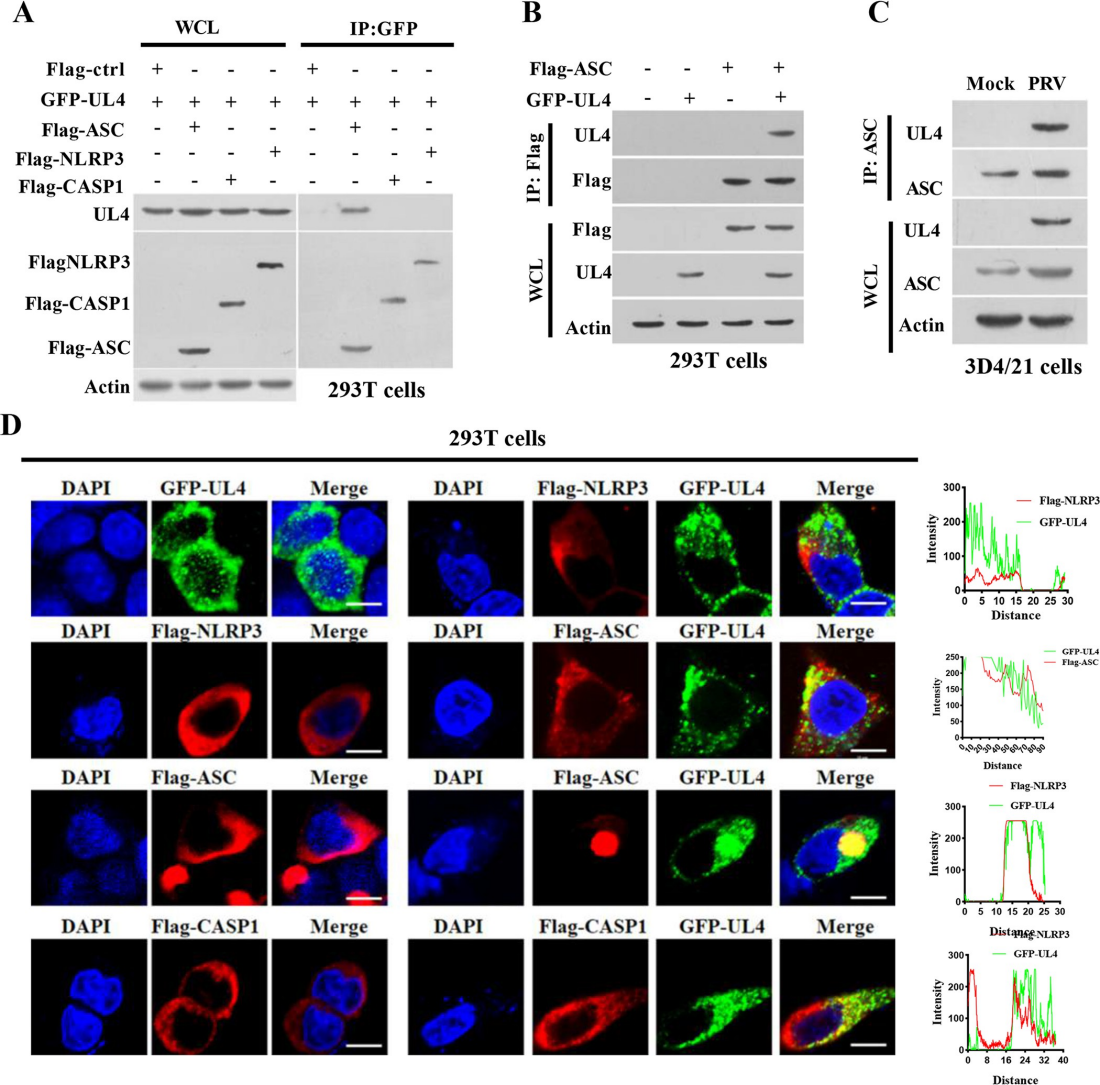
PRV UL4 interacts with ASC. **(A-C)** Interaction between the PRV UL4 and ASC was detected by Co-IP. 293T cells were co-transfected with plasmids expressing Flag-ctrl, Flag-NLRP3, Flag-CASP1, or Flag-ASC, along with GFP-UL4 for 24 h. Whole-cell lysates (WCL) were immunoprecipitated using the indicated antibody, followed by immunoblotting with indicated antibodies (A). 293T cells were transiently transfected with the indicated plasmids for 24 h and the interaction between GFP-UL4 and Flag-ASC was verified by Co-IP analysis (B). 3D4/21 cells were infected with Mock or PRV (5 MOI) for 12 h. WCL were immunoprecipitated with an anti-ASC antibody, followed by immunoblotting using indicated antibodies (C). **(D)** IFA analysis of the interaction between GFP-UL4 and Flag-NLRP3, Flag-ASC, and Flag-CASP1 in 293T cells co-transfected with indicated plasmids for 24 h, then visualized with confocal microscopy. Scale bar, 10 μm.

To investigate the mechanism by which PRV UL4 protein regulated the NLRP3 inflammasome, we first co-overexpressed HA-tagged UL4 and flagged-tagged NLRP3 inflammasome components (NLRP3, ASC, or CASP1) in 293T cells, and performed co-immunoprecipitation (Co-IP) to further delineate the association between viral UL4 and the components of inflammasome complex. Obviously, Co-IP assays showed that UL4 protein only interacted with ASC and failed to interact with NLRP3 or CASP1 in 293T cells (Fig 5A). Reciprocal Co-IP assays also confirmed that ASC interacted with UL4 (Fig 5B). Similarly, endogenous ASC was co-immunoprecipitated with UL4 in PRV-infected 3D4/21 cells (Fig 5C). Moreover, confocal microscope analysis revealed that Flag-NLRP3, Flag-ASC, and Flag-CASP1 were localized in the cytoplasm. There were two forms of ASC, most of which were dispersed in the cytoplasm, and a small amount of them were able to form a ring-like structure in the transfected 293T cells (Fig 5D), which was consistent with previous reports [28]. Interestingly, UL4 colocalized not only with the ASC scattered in the cytoplasm but also with the ASC together forming a ring-like structure in the cytosol, while UL4 failed to interact with NLRP3 protein or CASP1 in 293T cells (Fig 5D). Taken together, these findings demonstrate that UL4 binds ASC and may exert regulation of the ASC-dependent inflammasome.

### 3.6 The 132aa–145 aa of UL4 permits its translocation to the cytoplasm to interact with cytoplasmic ASC to promote the NLRP3 and AIM2 inflammasome activation

**Fig 6:**
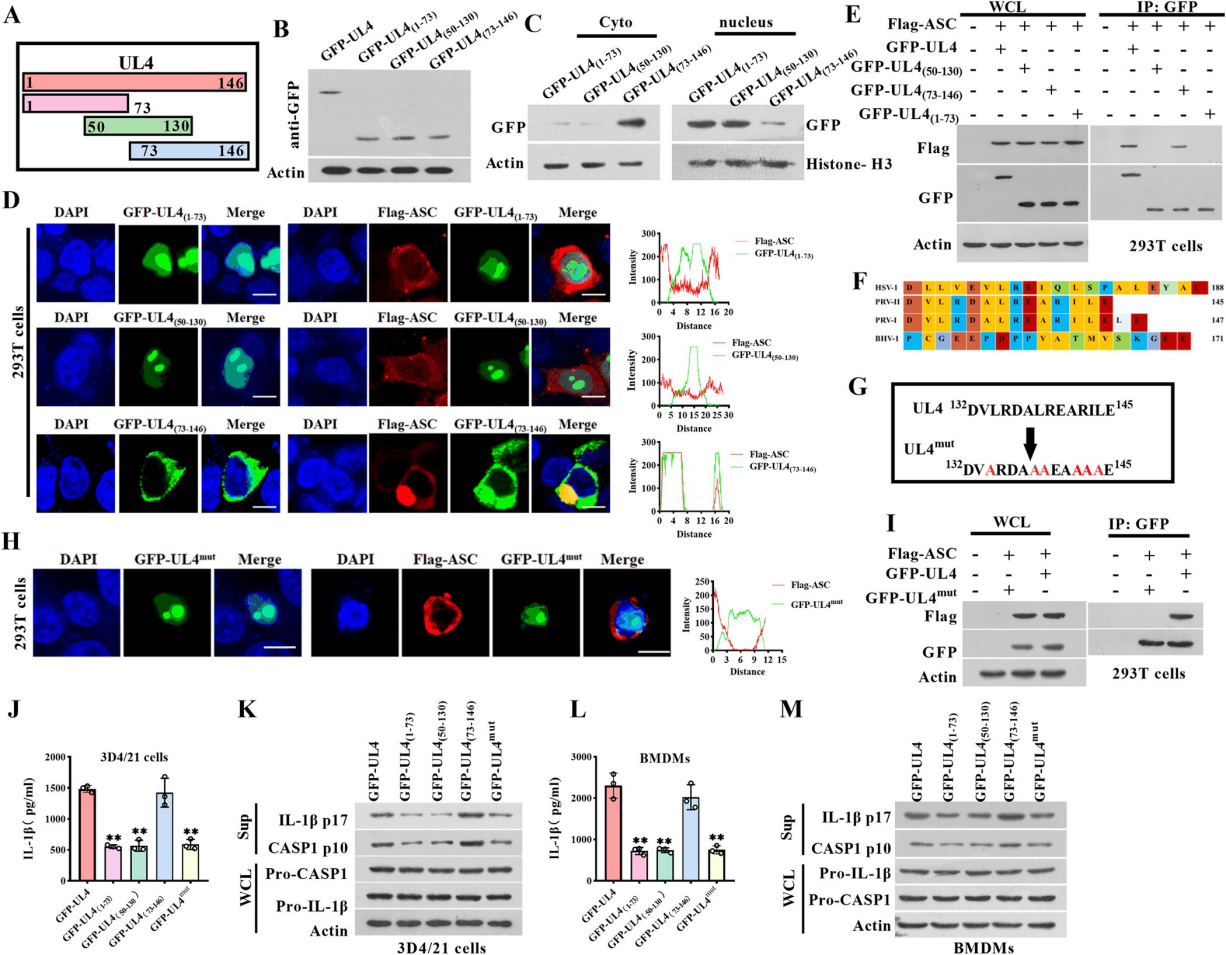
Sequence of UL4 is involved in inflammasome activation. **(A-C**) Identification of the truncated UL4 expression. Schematic diagram of PRV UL4 protein and truncated mutants UL4 protein (A). Western blotting detected the expression of UL4 and truncated mutants UL4 using GFP antibody (B, C). **(D, E)** Interaction between the truncated UL4 and ASC was detected by IFA and Co-IP. IFA analysis of the interaction between truncated mutants UL4 and Flag-ASC in 293T cells co-transfected with indicated plasmids for 24 h, then visualized with confocal microscopy (D). 293T cells were co-transfected with plasmids expressing porcine ASC and plasmids expressing UL4 protein or truncated UL4 protein as indicated for 24 h (E). WCL were immunoprecipitated with the indicated antibody, followed by immunoblotting using indicated antibodies. **(F-I)** Identification of key domains of UL4 and ASC interaction. Alignment diagram of Herpesvirus UL4 amino acid partial sequence (F). Schematic diagram of UL4 mutant (G). IFA analysis of the interaction between GFP-UL4^mut^ and Flag-ASC in 293T cells co-transfected with indicated plasmids for 24 h, then visualized with confocal microscopy (H). 293T cells were co-transfected with the indicated plasmids for 24 h. WCL were immunoprecipitated with the indicated antibody, followed by immunoblotting using indicated antibodies (I). **(J-M)** Identification of UL4 key domains that promote the activation of NLRP3 inflammasome and AIM2 inflammasome. 3D4/21 cells were respectively transfected with plasmids expressing UL4 protein or truncated mutants UL4 protein for 24 h, followed by the stimulation of LPS/NG (J, K). BMDMs were respectively transfected with plasmids expressing UL4 protein or truncated mutants UL4 protein for 24 h, followed by the stimulation of Poly dA:dT (L, M). Supernatants were subjected to ELISA for IL-1β secretion (J, L). Sup and WCL were analyzed by immunoblotting to detect the indicated proteins (K, M). ** *P* < 0.01, compared with the cells expressing UL4. Scale bar, 10 μm.

To identify the key region and amino acid residues of UL4 that are involved in the binding with ASC, we next measured the interaction between the ASC and truncated mutants of UL4: UL4_(1–73)_, UL4_(50–130)_, and UL4_(73–146)_ (Fig 6A). The expression of truncated UL4 was shown in Fig 6B. IFA assays and western blotting analysis showed that the UL4_(1–73)_ and UL4_(50–130)_ were accumulated exclusively in the nucleus, whereas mutant UL4_(73–146)_ distributed in cytoplasm patterns (Fig 6C and 6D). ASC only colocalized with GFP-UL4_(73–146)_, not with GFP-UL4_(1–73)_ and GFP-UL4_(50–130)_ (Fig 6D). Following the IFA results, Co-IP results also showed that ASC interacted with UL4_(73–146)_, but failed to interact with UL4_(1–73)_ and UL4_(50–130)_ in 293T cells (Fig 6E), suggesting that the domain contained in the fragment 73aa–146aa of UL4 protein is involved in the interaction with ASC.

Previous studies have revealed that the leucine-rich ^178^VEVLREIQL^186^ of HSV-1 UL4 acted as a functional nuclear export signal (NES) which was responsible for the export of UL4 out of the nucleus [5]. By comparing the sequence homology between HSV-1 UL4 and PRV UL4, we found that ^132^DVLRDALREARILE^145^ of PRV UL4 exists similar amino acid residues as ^178^VEVLREIQL^186^ of HSV-1 UL4 (Fig 6F). Then, the putative NES of PRV UL4 (^132^DVLRDALREARILE^145^) was mutated by replacing the leucine and hydrophobic amino acid residues with neutral alanine residues to create ^132^DVAADAAAEAAAAE^145^ (UL4^mut^) (Fig 6G). We found that the UL4^mut^ lost the ability to locate in the cytoplasm (Fig 6H) and could not interact with ASC (Fig 6I). Taken together, we demonstrated that the motif 132aa–145 aa of UL4 is the key domain for the translocation of UL4 out of the nucleus that determines its interaction with cytoplasmic ASC.

Then, the effect of UL4 protein mutants on the activation of the NLRP3 inflammasome was evaluated. ELISA assays showed that similar to the UL4 expressed cells, UL4_(73–146)_ assessed the ability to promote the IL-1β secretion, p17, and p10 levels, while UL4_(1–73)_, UL4_(50–130)_, and UL4^mut^ lost the ability to up-regulate the IL-1β secretion, p17 and p10 levels in the LPS/NG-stimulated 3D4/21 cells and Poly dA:dT-stimulated BMDMs (Fig 6J–6M). Taken together, these findings demonstrate that the sequence 132aa–145aa of UL4 is the key domain for its translocation out of the nucleus that determines its interaction with cytoplasmic ASC which is essential for promoting the NLRP3 and AIM2 inflammasome activation.

### 3.7 The UL4 mutation alleviates inflammasome activation and pyroptosis in PRV-infected cells

**Fig 7:**
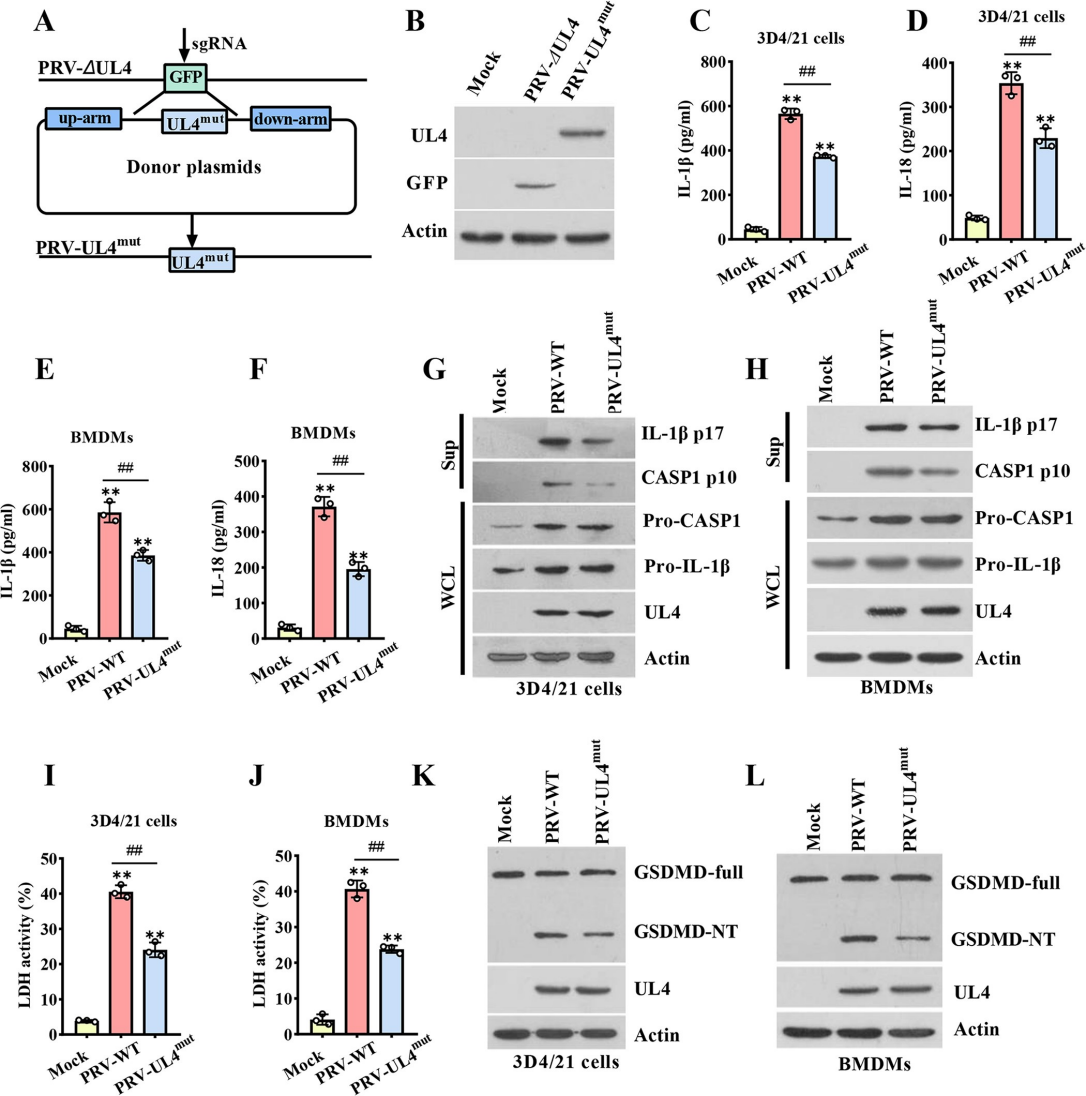
The UL4 mutation alleviates inflammasome activation and pyroptosis in PRV-infected cells. **(A, B)** Construction and identification of PRV-UL4^mut^. The construction flowchart of *UL4* gene mutation virus strain (A). 3D4/21 cells were infected with Mock, PRV-WT, or PRV-UL4^mut^ (5 MOI) for 12 h, then the expression of UL4 and GFP was detected by immunoblotting (B). **(C-H)** The effects of UL4 mutation on the secretion of IL-1β and IL-18 in PRV-infected cells. 3D4/21 cells (C, D, G) or BMDMs **(**E, F, H) were infected with Mock, PRV-WT, or PRV-UL4^mut^ (5 MOI) for 12 h. **(I-L)** Effect of UL4 mutation on cell pyroptosis. 3D4/21 cells (I, K) or BMDMs (J, L) were infected with Mock, PRV-WT, PRV-UL4^mut^ (5 MOI) for 12 h. ELISA assay for IL-1β (C, E) and IL-18 (D, F) in supernatants was measured, and Sup and WCL were analyzed by immunoblotting for indicated protein (G, H). Supernatants were collected to analyze the LDH activity (I, J); cell lysates were analyzed by immunoblotting to detect the indicated proteins (K, L). ** *P* < 0.01, compared with the Mock-infected cells, ^##^ *P* < 0.01, versus the PRV-WT-infected cells.

To investigate whether mutation of UL4 after virus infection can affect inflammasome activation and pyroptosis, we constructed a UL4 gene mutation strain (PRV-UL4^mut^) as shown in Fig 7A. The PCR amplification results showed that PRV DNA was successfully amplified *UL4* (S3A Fig). Similarly, western blotting analysis showed UL4 expression was detectable in PRV-UL4^mut^-infected cells (Fig 7B), sequencing results were consistent with this (S3B Fig). Then, we examined the effect of UL4 mutation on the inflammasome-mediated secretion of IL-1β and IL-18 during PRV infection in 3D4/21 cells and BMDMs. ELISA assays showed that, compared to the PRV-WT infection, PRV-UL4^mut^ infection secreted a profoundly lower level of IL-1β and IL-18 in 3D4/21 cells and BMDMs (Fig 7C–7F). Western blotting analysis also showed that UL4 mutation impaired the generation of the IL-1β p17 and CASP1 p10, while it unaffected the expression of pro-CASP1 or Pro-IL-1β (Fig 7G and 7H). Next, we assessed the effects of the mutation of UL4 on the pyroptosis induced by PRV. Compared to the PRV-WT infection, PRV-UL4^mut^ infection induced significantly decreased LDH (Fig 7I and 7J) and less GSDMD-NT amounts (Fig 7K and 7L). Interestingly, there was no significant difference in copy numbers between the PRV and the PRV-UL4^mut^ in 3D4/21 cells and BMDMs at 12 h.p.i (S3C and S3D Fig), which ruled out these differences due to different amounts of virus. These results further confirm the sequence 132 aa–145 aa of UL4 is the key domain in promoting ASC-dependent inflammasome activation and pyroptosis.

### 3.8 PRV UL4 promotes ASC oligomerization and speck formation

**Fig 8:**
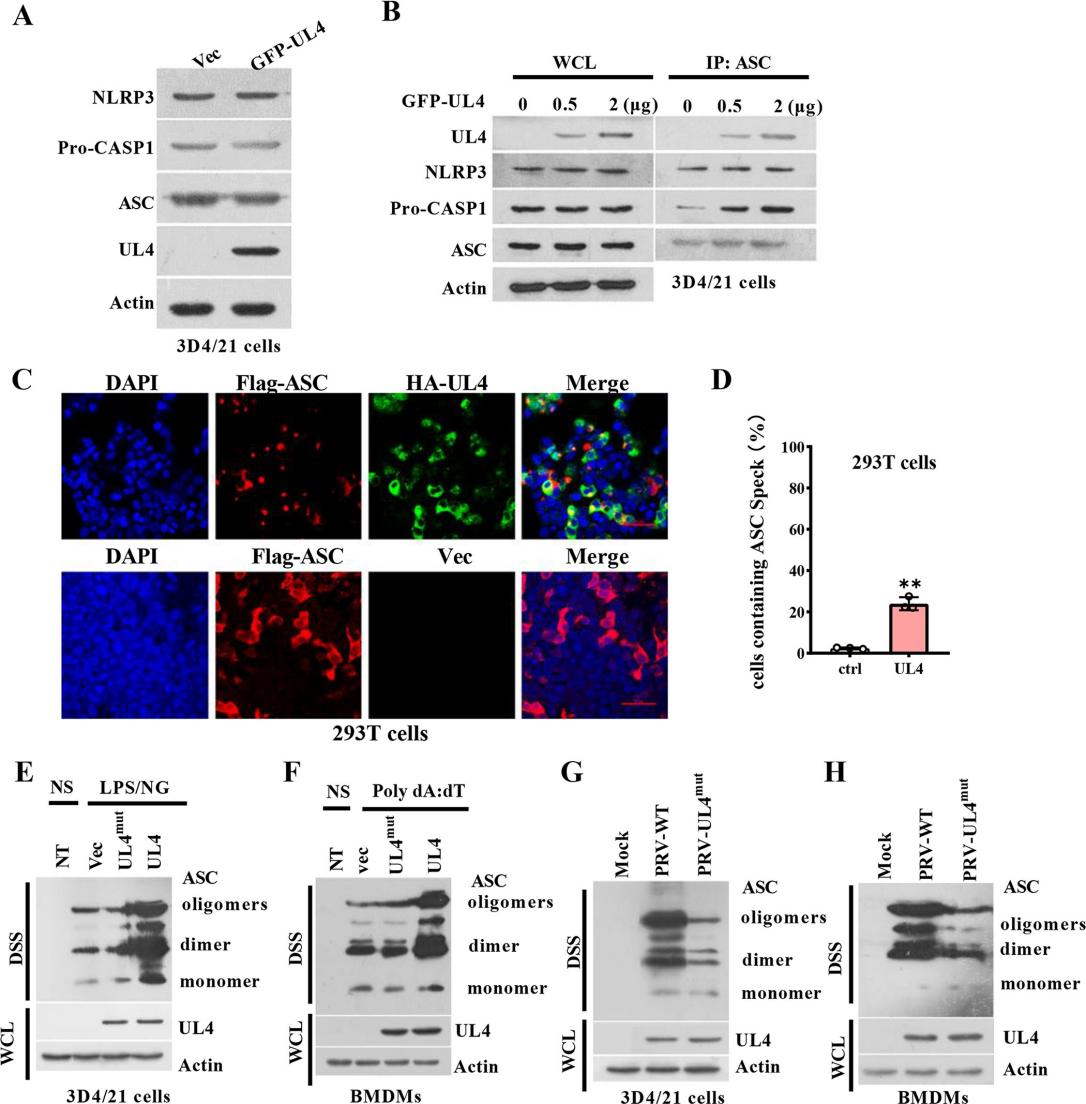
PRV UL4 augments ASC oligomerization and speck formation. **(A, B)** Detection of the effect of UL4 on NLRP3 inflammasome. 3D4/21 cells co-transfected with pEGFP-N1 or pEGFP-UL4 for 24 h were lysed, and the indicated protein expression levels were analyzed by immunoblotting (A). 3D4/21 cells were co-transfected with different concentrations of pEGFP-UL4 for 24 h, followed by stimulation of LPS/NG (B). WCL were immunoprecipitated using anti-ASC antibodies and analyzed using the indicated antibody. **(C, D)** The effect of UL4 on the formation of ASC speck. 293T cells were co-transfected plasmids encoding Flag-ASC with Vec (PCMV-HA) or plasmids encoding HA-UL4 for 24 h, ASC specks were detected by immunofluorescence staining using anti-Flag. Bars, 50 μm (C); Percentage of cells with ASC speck structures in the experiments in C (D). Quantified data are presented as mean ± SEM of the percentage of cells with ASC speck formation based on biological replicates. **(E-H)** The effect of UL4 on ASC oligomerization. 3D4/21 cells (E) or BMDMs (F) were respectively transfected with plasmids expressing UL4^mut^, UL4, or GFP (Vec), followed by stimulation of LPS/NG (E) or Poly dA:dT (F); 3D4/ 21 cells (G) or BMDMs (H) were infected with Mock, PRV-WT or PRV-UL4^mut^ for 12 h; then ASC in disuccinimidyl suberate (DSS)–treated Triton-insoluble fractions of indicated cells was analyzed by immunoblotting. ** *P* < 0.01, compared with the Vec transfected cells.

By co-transfection of the inflammasome components with UL4, we further verified the effect of UL4 expression on the activation of NLRP3 inflammasome. Consistent with the above results, UL4 did not affect the expression of inflammasome components (Fig 8A), indicating that UL4 mediated the process of NLRP3 inflammasome activation instead of priming. Although the interaction of NLRP3 and ASC was unaffected by UL4 protein in dose-dependent manners, the interaction between pro-CASP1 and ASC was enhanced by it in 293T cells (Fig 8B), which suggested that UL4 acted at or after ASC and NLRP3 speck formation. Activation of NLRP3 and AIM2 inflammasome involves the aggregation of the adaptor ASC and the formation of microscopically visible specks, which are cellular platforms to activate CASP1 [28]. Notably, a certain number of cells with ASC specks was detected in ASC-expressed cells, while ASC speck formation was increased when 293T cells were transfected with HA-UL4 (Fig 8C). Specifically, while 2% of NP-positive cells displayed ASC specks in 293T cells, 24% of NP-positive cells exhibited ASC specks in UL4-expressed cells (Fig 8D). Importantly, oligomerization of endogenous ASC stimulated by LPS/NG or Poly dA:dT was further enhanced in the presence of UL4 in 3D4/21 cells and BMDMs, while the mutation of UL4 alleviated this phenomenon (Fig 8E and 8F). Instead, the formation of ASC dimers and oligomers was profoundly reduced in PRV-UL4^mut^ infection cells compared with PRV-infected 3D4/21 cells and BMDMs (Fig 8G and 8H). Altogether, these data demonstrate that UL4 facilitates NLRP3 inflammasome activation by regulating ASC oligomerization and speck formation.

### 3.9 PRV UL4 promotes the ASC phosphorylation

**Fig 9:**
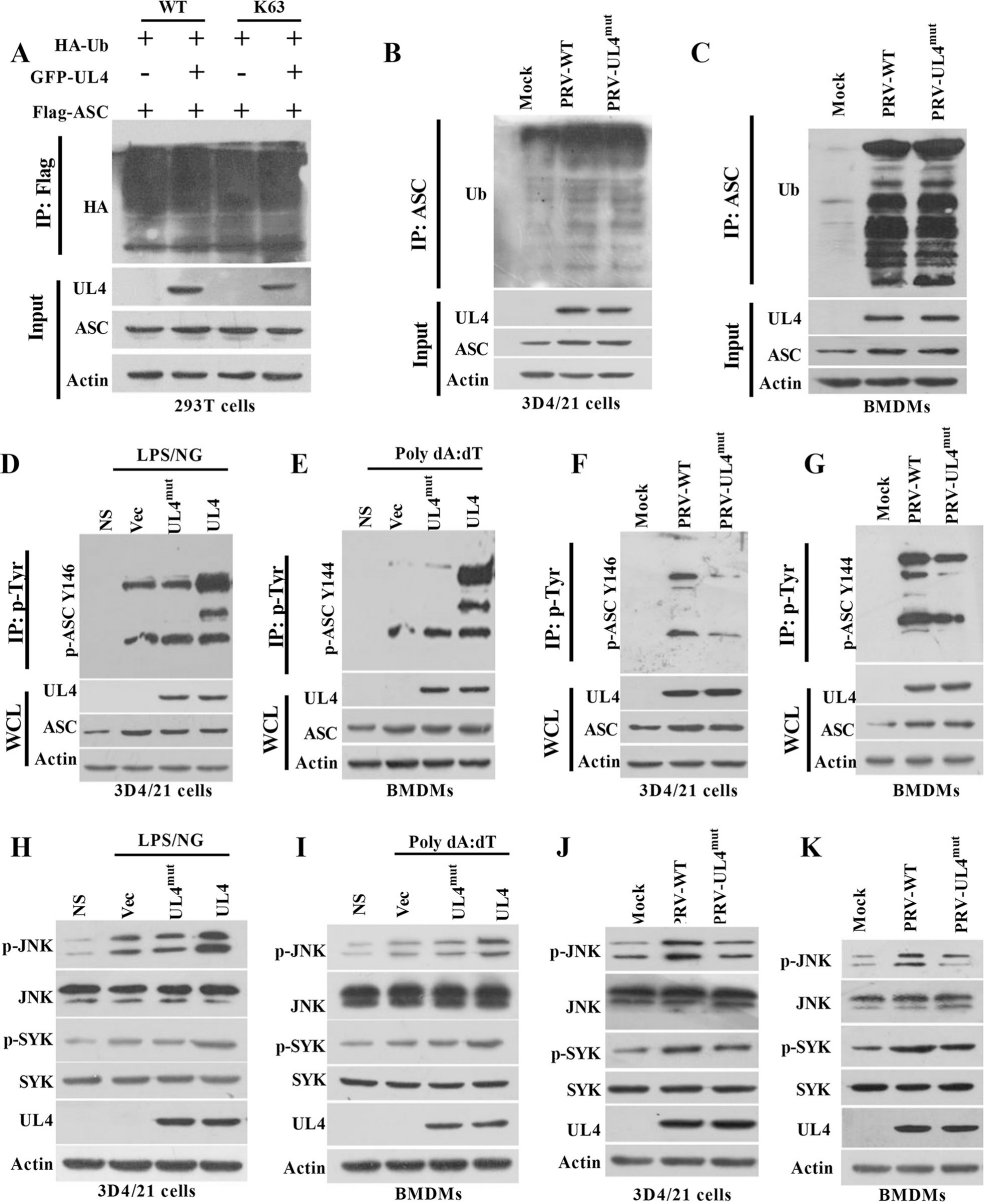
PRV UL4 protein facilitates ASC oligomerization and speck formation by promoting ASC phosphorylation. **(A-C)** The effect of PRV UL4 on the polyubiquitination level of ASC. Immunoprecipitation analysis of ASC ubiquitination in 293T cells transfected with plasmids encoding Flag-ASC, GFP-UL4, HA-tagged WT, or mutant ubiquitin harboring lysine-to-arginine mutations in all lysine residues except lysine 63 (ubiquitin K63) for 24 h (A); Immunoprecipitation analysis of ASC ubiquitination in 3D4/21 cells (B) and BMDMs (C) infected with Mock, PRV-WT, PRV-UL4^mut^ for 12 h. **(D-G)** The effect of PRV UL4 on the phosphorylation level of ASC Y144 (equivalently l to porcine Y146). Immunoprecipitation analysis of ASC Y146 phosphorylation in 3D4/21 cells or ASC Y144 phosphorylation in BMDMs, transfected with plasmids encoding UL4^mut^, UL4, or Vec for 24 h, followed by stimulation of LPS/NG (D) or Poly dA:dT (E); Immunoprecipitation analysis of ASC Y146 phosphorylation in 3D4/21 cells (F) or ASC Y144 phosphorylation in BMDMs (G), infected with Mock, PRV-WT, PRV-UL4^mut^ for 12 h. **(H-K)** The effect of PRV UL4 on the phosphorylation level of JNK and SYK. Western blotting analysis of indicated proteins in 3D4/21 cells (H) or BMDMs (I) were transfected with plasmids encoding UL4^mut^, UL4, or GFP for 24 h, followed by stimulation of LPS/NG (H) or Poly dA:dT (I). Western blotting analysis of indicated proteins in 3D4/21 cells (J) or BMDMs (K) infected with Mock, PRV-WT, PRV-UL4^mut^ for 12 h.

ASC ubiquitination and phosphorylation are essential for the oligomerization of ASC and subsequent CASP1 activation [24, 29], thus we investigate whether PRV UL4 protein may regulate the ASC at the posttranslational levels. We first examined the ASC ubiquitination after the exogenous expression of UL4. Notably, in the UL4 expressed cells, the total and K63 ubiquitination levels of ASC were all not affected (Fig 9A). Then, the ubiquitination level of ASC in Mock, PRV, or PRV-UL4^mut^ infected 3D4/21 cells and BMDMs was tested. The results showed that ASC was sightly ubiquitinated in Mock-infected cells. Though the total cellular ubiquitination levels of ASC were enhanced by PRV infection, the ubiquitination levels of ASC did not appear obvious difference between PRV- and PRV-UL4^mut^ infected cells (Fig 9B and 9C). These results suggest that the viral UL4 does not affect the ubiquitination of ASC.

Moreover, we explored the effect of UL4 on ASC phosphorylation in PRV-infected cells. Since phosphorylation of murine ASC Y144 (equal to porcine ASC Y146) is crucial for the oligomerization of ASC and subsequent CASP1 activation. To further assess whether viral UL4 affects the phosphorylation of porcine ASC Y146, phosphorylated proteins were immunoprecipitated using an anti-p-Tyr antibody and immunoblotted with an anti-p-ASC Y144 antibody. Interestingly, the phosphorylation of ASC Y146 was obviously increased in the UL4-expressed cells not in the UL4^mut^-expressed cells (Fig 9D and 9E), suggesting that UL4 can promote the phosphorylation of porcine ASC at Y146 site. On the contrary, the phosphorylation of ASC Y146 was diminished in the PRV-UL4^mut^-infected cells compared to the PRV-infected cells (Fig 9F and 9G). However, the specific mechanism by which UL4 promotes increased levels of ASC phosphorylation remains to be investigated. It has been reported that SYK and JNK are responsible for regulating the phosphorylation of murine ASC Y144 [20, 30], thus the effect of UL4 on their phosphorylation levels was further detected. It was found that the expression of UL4, not the UL4^mut^ promoted the phosphorylation of SYK and JNK in BMDMs and 3D4/21 cells (Fig 9H and 9I). In contrast, phosphorylation levels of SYK and JNK were lower in PRV-UL4^mut^-infected BMDMs and 3D4/21 cells than that in PRV-infected cells (Fig 9J and 9K). Collectively, these results suggest that UL4 plays a critical role in promoting the phosphorylation of porcine ASC Y146 through enhancing the phosphorylation of SYK-JNK.

### 3.10 The UL4 mutation alleviates inflammation and reduces the pathogenicity in vivo

**Fig 10:**
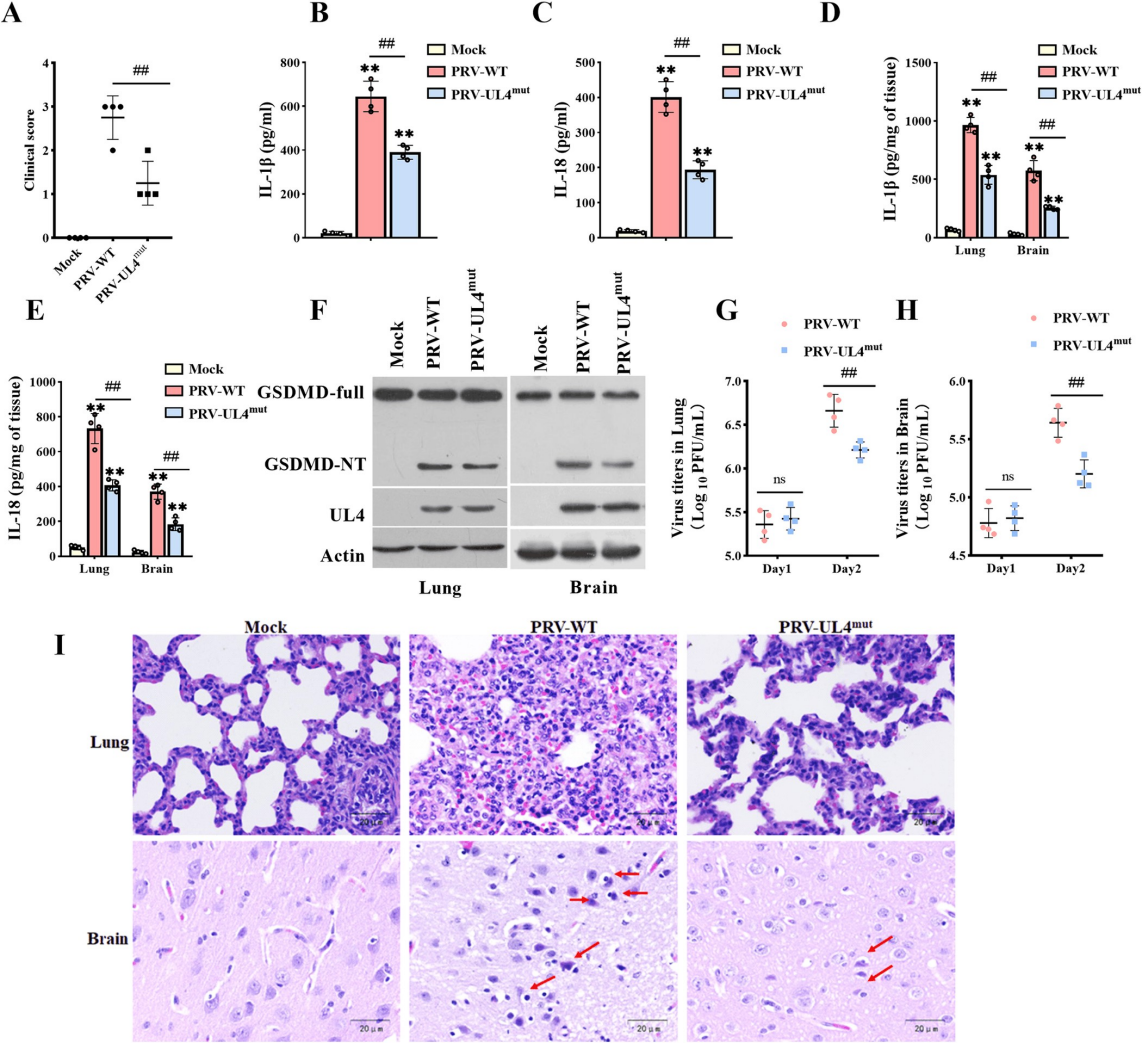
The UL4 mutation alleviates inflammation in PRV-infected pigs. pigs (3 weeks old) were nasal infections with Mock, PRV-WT, or PRV-UL4^mut^ at 10^8^ TCID_50_/pig. **(A)** clinical score of pigs infected with Mock, PRV, or PRV-UL4^mut^ at postinfection 48 h (n = 4 per group). **(B-E)** The effects of UL4 mutation on the secretion of IL-1β (B, C) or the IL-18 (D, E) in the peripheral blood (B, C) or the lung and brain tissues of mice (D, E) was detected by ELISA at postinfection 24 h. **(F)** The effects of UL4 mutation on the GSDMD-NT in lung and brain tissues were detected by western blotting at postinfection 24 h. **(G, H)** PFU in the indicated organs on indicated times. **(I)** Histopathology analysis (H&E staining) of pigs from the lungs and brain tissues was shown. ** *P* < 0.01, versus the Mock-infected group (B, C, D, E); ^##^ *P* < 0.01, compared with the PRV-WT infected group with the same treatment (A, B, C, D, E, G, H).

Next, we investigated the contribution of UL4-regulated inflammasome responses to the virulence of PRV in *vivo*. First, we used mice to compare the virulence of wild-type PRV and the mutated strain PRV-UL4^mut^. As shown in S4A Fig, all mice in the PRV-infected group died within 60 h after inoculation, while the part of mice infected with PRV-UL4^mut^ did not die till 96 h. We observed severe clinical symptoms in the PRV group at 48 h following inoculation, but slightly in PRV-UL4^mut^ infected pigs and mice (Figs 10A and S4B). Consistently, the secreted IL-1β and IL-18 levels in the serum from PRV-UL4^mut^-infected pigs and mice were significantly lower than those in PRV-infected pigs and mice at 24 h following inoculation (Figs 10B, 10C, S4C and S4D). In addition, the inflammatory factors in the lung and brain tissues of PRV-UL4^mut^-infected pigs and mice were also significantly decreased compared with that in the PRV-infected group (Figs 10D, 10E, S4E and S4F). Consistently, the amount of GSDMD-NT was also decreased in PRV-UL4^mut^-infected pigs and mice (Figs 10F, S4G). The PRV loads in lung and brain tissues from pigs and mice infected with PRV-UL4^mut^ were also similar to those of the PRV-WT-infected group at postinfection 24 h, while compared to the PRV-WT infection, the PRV loads in lung and brain tissues were decreased at postinfection 48 h (Figs 10G, 10H, S4H and S4I). Additionally, pathological examinations demonstrated that the lung tissues from PRV-UL4^mut^-infected mice and pigs exhibited less necrosis of bronchioles and alveolar epithelial cells than those from the PRV-infected group. And brain tissues from PRV-UL4^mut^-infected mice and pigs exhibited less nerve cell necrosis, vascular cuff, and microgliosis than those from PRV-infected mice and pigs (Figs 10I and 10J). The mean lung and brain histological lesion scores in the PRV-UL4^mut^-infected group were lower than those in the PRV-WT infection group (Tables 1 and 2). These results suggest that the deletion of UL4 reduces the pathogenicity of PRV, which is associated with the release of inflammatory factors or pyroptosis.

**Table 1:** Comparison of histological lesion scores of lungs and brains from PRV-WT and PRV-UL4^mut^ infected piglets.

Group^*a*^	Piglet label	Histological lesion score	
		Lungs^*b*^ Brains^*c*^	
DMEM	A	0	0
	B	0	0
	C	0	0
	D	0	0
PRV-WT	E	5	2
	F	4	2
	G	4	3
	H	3	3
PRV-UL4^mut^	I	1	1
	J	2	1
	K	1	2
	L	3	1

^***a***^DMEM; PRV-WT, pseudorabies virus; PRV-UL4^mut^, a PRV mutant.

^***b***^Score ranges from 0 (normal) to 5 (severe).

^***c***^Score ranges from 0 (normal) to 3 (severe).

**Table 2:** Comparison of histological lesion scores of lungs and brains from PRV-WT and PRV-UL4^mut^ infected mice.

Group^*a*^	Mouse label	Histological lesion score	
		Lungs^*b*^ Brains^*c*^	
DMEM	A	0	0
	B	0	0
	C	0	0
	D	0	0
	E	0	0
	F	0	0
	G	0	0
PRV-WT	H	5	2
	I	4	2
	J	4	3
	K	3	3
	L	4	3
	M	5	2
	N	4	3
PRV-UL4^mut^	O	1	1
	P	2	1
	Q	1	2
	R	3	1
	S	2	1
	T	2	1
	U	1	2

^***a***^DMEM; PRV-WT, pseudorabies virus; PRV-UL4^mut^, a PRV mutant.

^***b***^Score ranges from 0 (normal) to 5(severe).

^***c***^Score ranges from 0 (normal) to 3 (severe).

## 4 Discussion

Activated inflammasome plays an important role in host defense against pathogenic microorganisms by promoting the secretion of pro-inflammatory factors and pyroptosis, but dysregulation of inflammasome activation also contributes to the development of inflammatory diseases. It has been reported that pyroptosis is primarily responsible for the rapid death of mice infected with PRV [10]. Several studies have shown that viral proteins (including HSV-1 VP22 protein and dengue virus M protein) can regulate the activation of the inflammasome [31–38]. This study attempts to screen the key protein of PRV that regulates the activation of inflammasome, to achieve the possibility of controlling the inflammatory infection process. In the present study, a novel mechanism by which PRV UL4 protein promotes inflammasome activation to facilitate IL-1β maturation is revealed.

Firstly, ORFs corresponding to PRV were cloned and constructed into pEGFP-N1 vector to express PRV-encoded protein. Subsequently, the porcine NLRP3 inflammasome system reconstructed in 293T cells was stimulated by LPS/NG to verify the effect of PRV protein on IL-1β secretion. It was found that UL4, a non-structural protein of PRV, could significantly promote LPS/ NG-induced IL-1β secretion in the NLRP3 inflammasome system. UL4 is localized in the cytoplasm and nucleus of infected cells, and its function is largely unknown. Studies have shown that although UL4 is not necessary for PRV replication in vitro or in vivo, it may contribute to the formation of virions by facilitating viral release [6,7]. From our screening results, in addition to UL4, several other PRV proteins also bear the ability to promote IL-1β secretion, though to a much weaker extent than that with UL4. In addition, a variety of PRV proteins inhibited the activation of NLRP3 inflammasome, indicating the dual role of viral proteins in regulating inflammasome. Although more studies are required to confirm the promoting effect of these proteins on IL-1β signaling, nevertheless these results suggest that PRV may dispatch multiple proteins to regulate inflammasome signaling by multiple mechanisms, to which UL4 only partly contributes. Further studies found that IL-1β production and UL4 expression were dose-dependent, suggesting that increased UL4 might be amplifying inflammatory responses as the infection progresses. Next, the effect of UL4 on NLRP3 inflammasome activation was verified in 3D4/21 cells and BMDMs containing endogenous NLRP3 inflammasome components. After LPS/NG stimulation, UL4 expression also significantly promoted the secretion of IL-1β and IL-18 in 3D4/21 cells and BMDMs. The process of PRV infection of target cells does not only activate one inflammasome, after infecting 3D4/21 cells, PRV can activate IFI16 and NLRP3 inflammasome [11], and after infecting BMDMs, PRV can activate AIM2 and NLRP3 inflammasome [25]. Interestingly, using BMDMs and the reconstructed murine AIM2 inflammasome system in 293T cells, we found that PRV UL4 also promoted the activation of the AIM2 inflammasome, further demonstrating its importance in regulating the inflammatory response caused by PRV infection. These results indicated that the promotion effect of UL4 on inflammasome activation is common for ASC-dependent inflammasome platforms. To further elucidate the effect of UL4 on the activation of inflammasome during PRV infection, the UL4-deletion strain PRV-*Δ*UL4 was successfully constructed. Compared with PRV-WT infected cells, the amount of IL-1β and IL-18 and the protein content of p17 and p10 were obviously decreased in PRV-*Δ*UL4 infected cells [11]. Given the relationship between inflammasome and pyroptosis, the role of UL4 in pyroptosis was also tested, and it was found that UL4 expression promoted GSDMD cleavage and LDH release. However, the deletion of *UL4* of PRV resulted in the GSDMD cleavage, and LDH release was obviously reduced.

To prevent adverse tissue damage caused by overactivation of inflammasome, its activation is precisely regulated to maintain immune homeostasis [16]. The assembly and activation of the inflammasome is an extremely complex process, regulated by several different mechanisms for each of the relevant components, and finely controlled by the inter-tandem of all signals [18]. The core components of the inflammasome are pattern recognition receptors and pro-CASP1. ASC is the universal adaptor protein for multiple inflammasome, which can bridge pro-CASP1 and multiple intracellular sensors including IFI16, AIM2, Pyrin, NLRP1b, NLRP3, NLRC4, NLRP6, NLRP9, and NLRP6 [39–41]. We first explored whether there was interaction between UL4 and inflammasome components. IFA and Co-IP proved that UL4 did not interact with NLRP3 and pro-CASP1, but did interact with ASC. In the meantime, we found that UL4 132–145 aa was related to the interaction of ASC, which determines the localization of UL4 in the cytoplasm. Further verification showed that UL4 132–145 aa was associated with its promotion of the activation of the NLRP3 and AIM2 inflammasome. Since PRV can activate multiple inflammasome during infection, targeting the common adaptor ASC such as UL4 appears to be an efficient strategy for PRV to control immune responses that facilitate virus expansion in the host. To further elucidate the effect of UL4 on the activation of ASC-dependent inflammasome during PRV infection, the UL4-mutation strain PRV-UL4^mut^ was successfully constructed. The amount of IL-1β and IL-18 and the protein content of p17, p10, and GSDMD-NT were obviously decreased in PRV-UL4^mut^ infected cells.

After the receptors recognize specific stimuli, the inflammasome is assembled, which acts as a platform for activating pro-CASP1, and the activated CASP1 in turn cuts Pro-IL-1β and pro-IL-18 as well as GSDMD [42]. However, the specific mechanism by which UL4 regulates inflammasome remains to be studied. It was not clear whether specifically affected, NLRP3-ASC interaction, Pro-CASP1-ASC interaction, or ASC aggregation. At the same time, other unknown functions of UL4 cannot be ruled out. Further studies found that UL4 did not affect the interaction between NLRP3 and ASC, but the interaction between ASC and pro-CASP1 was enhanced, suggesting that UL4 might affected ASC aggregation. Inactivated ASC exists in soluble form in resting cells, during inflammasome activation, ASC oligomerization forms ASC speck [43]. Therefore, we further verified the effect of UL4 on the speckling and oligomerization of ASC. Though Spontaneous speck formation after transfection of ASC in the absence of chemical induction has also been observed after transfection of 293T cells [28], our study showed that UL4 can promote the formation of ASC specks, while the specific mechanism of the promotion of ASC spots is still unclear. Our results showed that UL4 not UL4^mut^ promoted the formation of ASC oligomers in BMDMs and 3D4/21 cells. Compared with PRV-WT infected cells, the ASC oligomers were obviously decreased in PRV-UL4^mut^ infected cells.

Phosphorylation and ubiquitination play a crucial role in the formation of ASC speck [44,45]. Many studies have shown that multiple sites of ASC can be ubiquitinated, and the ubiquitination level is closely related to the formation of specks. For example, K63 linking polyubiquitination on ASC is conducive to spot formation of ASC [46], while the K48 linking polyubiquitination on it inhibits the activation of AIM2, NLRC4, and NLRP3 inflammasome [29]. Thus, we examined the effect of UL4 on the ubiquitination level of ASC and found that UL4 did not affect the total ubiquitination level of ASC, nor did it affect the K63 ubiquitination levels.

Phosphorylation of ASC is also required for its oligomerization and inflammasome activation, and multiple sites of ASC (including ASC Y60, Y137, Y144, S16, S193, etc.) can be phosphorylated during inflammasome signaling [30,47–49]. Among them, phosphorylation of ASC Y144 (equivalent to human/porcine Y146) facilitates the formation of ASC spots and the assembly of NLRP3 and AIM2 inflammasome. Our study showed that UL4 not UL4^mut^ can promote the phosphorylation of ASC Y144 (equivalent to porcine Y146) site, and further found it can promote the phosphorylation of SYK and JNK, but the specific mechanism of promoting phosphorylation remains to be studied. However, phosphorylation of ASC itself is not sufficient to induce the formation of spots. However, in the transfection system of 293T cells, overexpression of SYK and JNK can induce the phosphorylation of ASC, but hardly cause the formation of ASC spots [20,30]. In addition to PTM levels, ASC speck formation is also regulated by transcriptional levels, variable shear, and ion channels [28,50]. How UL4 promotes the formation of ASC spots, and whether it is related to other factors besides increasing the phosphorylation level of ASC, remains to be investigated.

PRV can infect mice and pigs and cause acute and severe diseases, often manifested as fatal neurological symptoms and respiratory system damage [51]. Pyroptosis is associated with the rapid death of mice infected with PRV, and PRV infection induces a specific and lethal inflammatory response in Mice [10,52]. In vivo, studies further demonstrated that mutation of the *UL4* gene alleviated inflammatory infiltration and pathological damage in the lungs and brains and delayed the death time of mice and pigs. Though mutation of the *UL4* gene did not lead to a significant decrease in viral titers in tissues at 12 h.p.i, the levels of IL-1β and IL-18 in the lung and brain tissues, and peripheral blood were significantly lower than those of PRV-WT infected pigs and mice, suggesting that PRV-UL4^mut^ infection attenuated the pathogenicity possibly by reducing the production of pro-inflammatory factors or pyroptosis. UL4 mutation or deletion leads to reduced viral copy number at a later time point after infection in *vitro* and *vivo*, we hypothesized that UL4 might promote viral release by promoting further activation of inflammasome and pyroptosis. It remains unclear what factor determines if the inflammasome is protective or detrimental to the host downstream of IL-18 secretion. Further research is needed to obtain a more precise understanding of this question.

In conclusion, our study found a novel function of UL4 to promote the activation of inflammasome which colocalized in the cytoplasm through interaction with ASC, promoted the phosphorylation levels of JNK and SYK to enhance the phosphorylation level of ASC, led to the increase of ASC oligomerization, thus promoting the activation of NLRP3 and AIM2 inflammasome, and enhanced GSDMD-mediated pyroptosis. This study provides new insights into the inflammatory response caused by PRV infection, and the research results help to deepen the understanding of the pathogenesis of PRV and provide a new basis and control target for the research and development of attenuated PRV vaccine.

## Supporting information

S1 Fig PRV UL4 is a promotor of NLRP3 inflammasome activation. **(A)** 293T cells were transfected with the indicated plasmids for 24 h, then the expression of the viral protein was identified by western blotting. **(B)** The role of EP0 or UL4 on NLRP3 inflammasome system reconstructed in 293T cells. 293T cells were co-transfected with NLRP3 inflammasome system-associated plasmids, along with plasmids expressing GFP-EP0 or GFP-UL4 for 24 h. Then, the cells were stimulated without (NS) or with LPS/NG. **(C)** The transfection efficiency of pEGFP-UL4 in 3D4/21 cells or BMDMs.
(TIF)

S2 Fig The UL4 null mutation alleviated inflammasome activation in PRV-infected cells. **(A, B)** Identification of PRV-*Δ*UL4. PCR amplification of *UL4* using the *UL4* specific primers and *GFP* with the *GFP* specific primers in PRV-WT DNA and PRV*-Δ*UL4 DNA (A); M: Marker, 1: *UL4* of PRV-WT DNA; 2: *UL4* of PRV*-Δ*UL4; 3: *GFP* of PRV-WT DNA; 4: *GFP* of PRV*-Δ*UL4 DNA. Partial sequencing results of the constructed recombinant virus of *UL4* CDS (B). **(C, D)** 3D4/21 cells (C) or BMDMs (D) were infected with PRV-WT, or PRV-*Δ*UL4 (5 MOI), then the expression of EP0 was detected by immunoblotting. (**E, F)** Q-PCR detected the DNA copies in 3D4/21 cells (E) or BMDMs (F) infected with 5 MOI PRV-WT or PRV-*Δ*UL4 for 12 h. **(G-L)** The effects of UL4 repair mutation on the secretion and maturation of IL-1β and IL-18 in PRV-infected cells. 3D4/21 cells (G, I, J) or BMDMs **(**H, K, L) were infected with Mock, PRV-WT, or PRV-UL4^repair^ (5 MOI) for 12 h. ELISA assay for IL-1β (I, K) and IL-18 (J, L) in supernatants was measured, Sup and WCL were analyzed by immunoblotting for indicated protein (G, H). ** *P* < 0.01, compared with the Mock-infected cells.
(TIF)

S3 Fig The UL4 mutation alleviated inflammasome activation and pyroptosis in PRV-infected cells. **(A, B)** Identification of PRV-UL4^mut^. PCR amplification of *UL4* of PRV-WT DNA and PRV-UL4^mut^ DNA (A). Partial sequencing results of the constructed recombinant virus of *UL4* CDS (B). **(C, D)** Q-PCR detected the DNA copies in 3D4/21 cells (C) or BMDMs (D) infected with 5 MOI indicated PRV at 12 h and 24 h. ** *P* < 0.01, compared with the PRV-WT-infected cells. **(E, F)** western blotting was used to detect the effect of SYK/JNK inhibition on ASC phosphorylation in 3D4/21 (E) and BMDMs (F).
(TIF)

S4 Fig The UL4 mutation alleviated inflammation in PRV-infected mice. C57BL/6 genetic background mice (6 weeks old) were intramuscular injection with Mock, PRV-WT, or PRV-UL4^mut^ at 10^6^ TCID_50_/mouse. **(A, B)** Effects of UL4 mutation on mortality and clinical symptoms in mice. Survival rates of mice infected with Mock, PRV, or PRV-UL4^mut^ (n = 10 per group). **(B)** Clinical score of indicated virus-infected mice at 48 h after infection. **(C-F)** The effects of UL4 mutation on the secretion of IL-1β (C, E) or the IL-18 (D, F) in the peripheral blood (C, D) or the lung and brain tissues of mice (E, F) was detected by ELISA at 24 h after infection. **(G)** The effects of UL4 mutation on the GSDMD-NT in mouse tissues were detected by western blotting at 24 h after infection. **(H, I)** PFU in the indicated organs on indicated times. **(J)** Histopathology analysis (H&E staining) of mice from the lung and brain tissues were shown. ** *P* < 0.01, compared with the PRV-WT infected group (A, B) or versus the Mock-infected group (C, D, E, F); ^##^ *P* < 001, compared with the PRV-WT infected group with the same treatment (C, D, E, F, H, I).
(TIF)

S1 Data Data for Figs 1A–1I, 2A–2J, 3B–3H, 4A–4L, 5A–5C, 6A–6C, 6E, 6H–6M and 7B–7L. (ZIP)

S2 Data Data for Fig 5D. (ZIP)

S3 Data Data for Fig 6D. (ZIP)

S4 Data Data for Figs 8A–8H, 9A–9K, 10A–10I. (ZIP)

S5 Data Data for Figs S1A–S1C, S2A, S2C–S2L, S3C–S3F, S4A–S4J. (ZIP)
