# Supplementary figures and images for: Pseudorabies Virus UL4 protein promotes the ASC-dependent inflammasome activation and pyroptosis to exacerbate inflammation (part 1 of 6)
Source: PLoS Pathog. 2024 Sep 24;20(9):e1012546. doi: 10.1371/journal.ppat.1012546 (PMC11421794; doi:10.1371/journal.ppat.1012546)

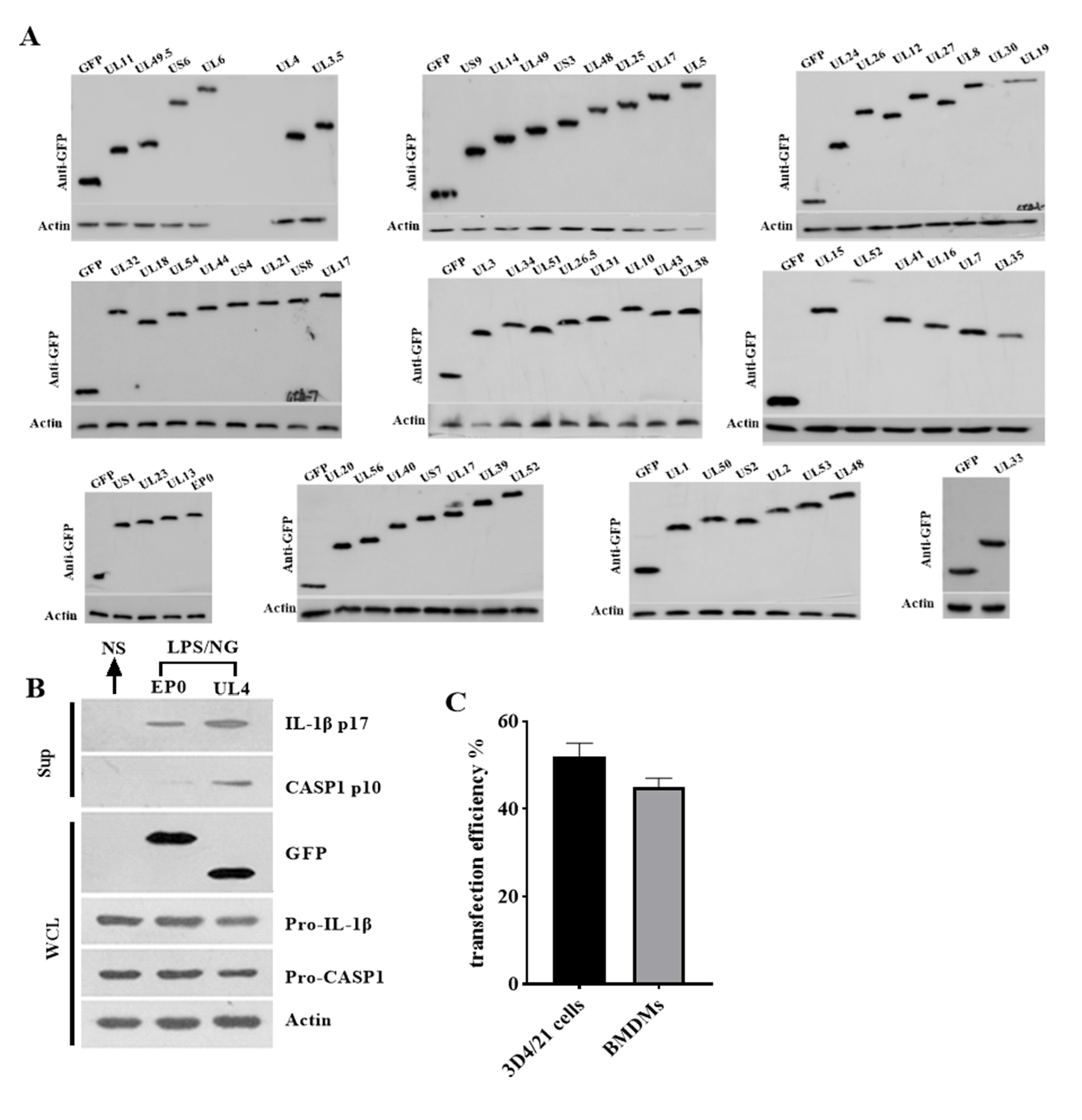

Supplement: S1 Fig — (A) 293T cells were transfected with the indicated plasmids for 24 h, then the expression of the viral protein was identified by western blotting. (B) The role of EP0 or UL4 on NLRP3 inflammasome system reconstructed in 293T cells. 293T cells were co-transfected with NLRP3 inflammasome system-associated plasmids, along with plasmids expressing GFP-EP0 or GFP-UL4 for 24 h. Then, the cells were stimulated without (NS) or with LPS/NG. (C) The transfection efficiency of pEGFP-UL4 in 3D4/21 cells or BMDMs. (TIF) [file ppat.1012546.s001.tif]

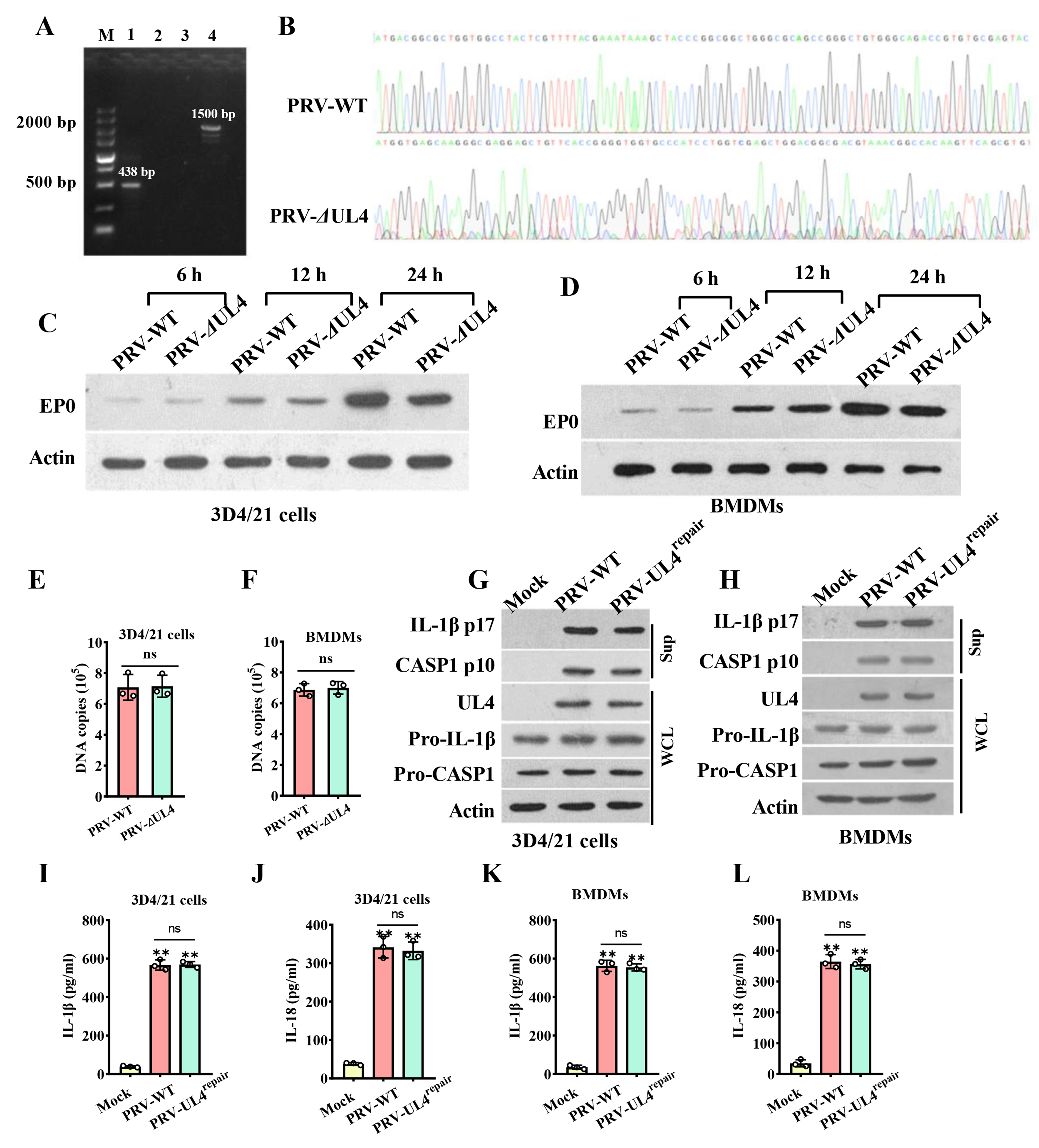

Supplement: S2 Fig — (A, B) Identification of PRV-ΔUL4. PCR amplification of UL4 using the UL4 specific primers and GFP with the GFP specific primers in PRV-WT DNA and PRV-ΔUL4 DNA (A); M: Marker, 1: UL4 of PRV-WT DNA; 2: UL4 of PRV-ΔUL4; 3: GFP of PRV-WT DNA; 4: GFP of PRV-ΔUL4 DNA. Partial sequencing results of the constructed recombinant virus of UL4 CDS (B). (C, D) 3D4/21 cells (C) or BMDMs (D) were infected with PRV-WT, or PRV-ΔUL4 (5 MOI), then the expression of EP0 was detected by immunoblotting. (E, F) Q-PCR detected the DNA copies in 3D4/21 cells (E) or BMDMs (F) infected with 5 MOI PRV-WT or PRV-ΔUL4 for 12 h. (G-L) The effects of UL4 repair mutation on the secretion and maturation of IL-1β and IL-18 in PRV-infected cells. 3D4/21 cells (G, I, J) or BMDMs (H, K, L) were infected with Mock, PRV-WT, or PRV-UL4repair (5 MOI) for 12 h. ELISA assay for IL-1β (I, K) and IL-18 (J, L) in supernatants was measured, Sup and WCL were analyzed by immunoblotting for indicated protein (G, H). ** P < 0.01, compared with the Mock-infected cells. (TIF) [file ppat.1012546.s002.tif]

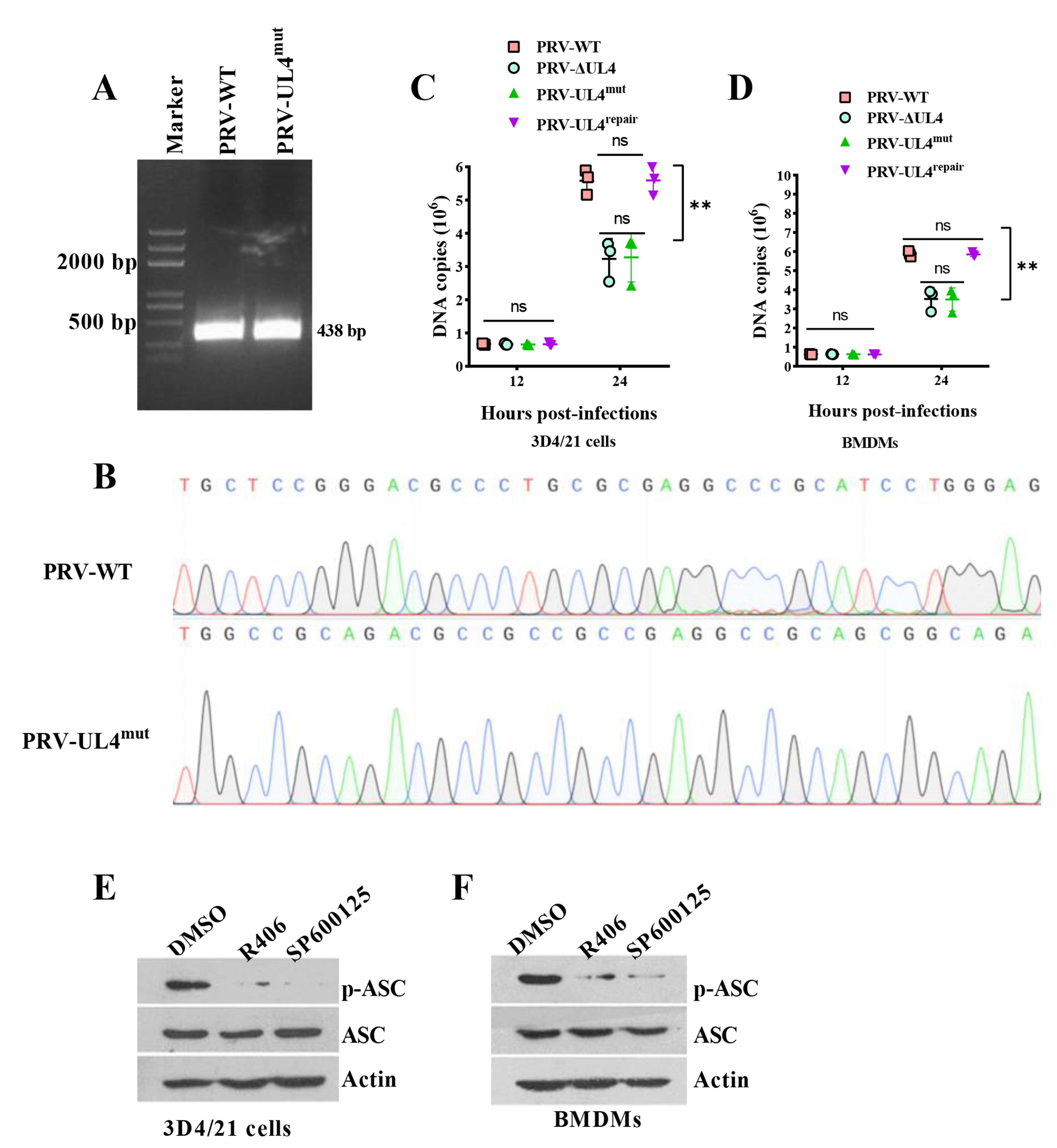

Supplement: S3 Fig — (A, B) Identification of PRV-UL4mut. PCR amplification of UL4 of PRV-WT DNA and PRV-UL4mut DNA (A). Partial sequencing results of the constructed recombinant virus of UL4 CDS (B). (C, D) Q-PCR detected the DNA copies in 3D4/21 cells (C) or BMDMs (D) infected with 5 MOI indicated PRV at 12 h and 24 h. ** P < 0.01, compared with the PRV-WT-infected cells. (E, F) western blotting was used to detect the effect of SYK/JNK inhibition on ASC phosphorylation in 3D4/21 (E) and BMDMs (F). (TIF) [file ppat.1012546.s003.tif]

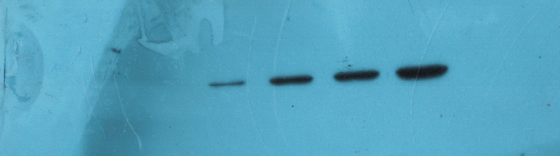

Supplement: S1 Data — (ZIP) [file ppat.1012546.s005.zip › Figure1-4, 5A-C, 6A-C, 6E, 6H-M and 7B-L. zip/Fig1/B/1/Sup-Pro-CASP1- p10.tif]

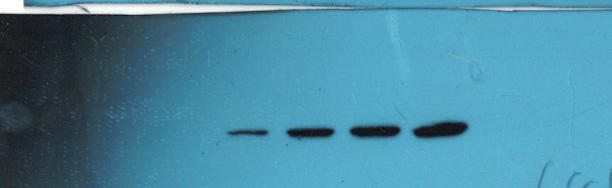

Supplement: S1 Data — (ZIP) [file ppat.1012546.s005.zip › Figure1-4, 5A-C, 6A-C, 6E, 6H-M and 7B-L. zip/Fig1/B/1/Sup-Pro-IL-1a┬ p17.tif]

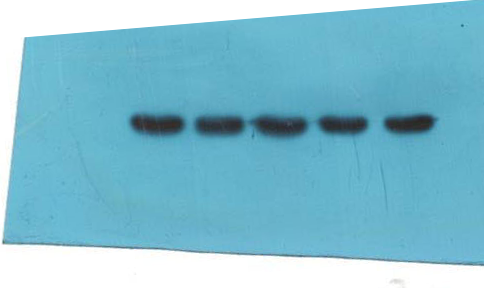

Supplement: S1 Data — (ZIP) [file ppat.1012546.s005.zip › Figure1-4, 5A-C, 6A-C, 6E, 6H-M and 7B-L. zip/Fig1/B/1/WCL-Actin.tif]

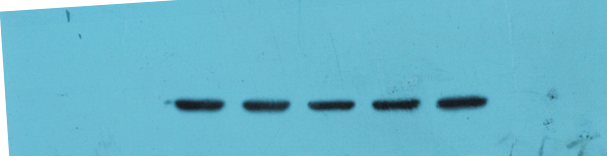

Supplement: S1 Data — (ZIP) [file ppat.1012546.s005.zip › Figure1-4, 5A-C, 6A-C, 6E, 6H-M and 7B-L. zip/Fig1/B/1/WCL-ASC.tif]

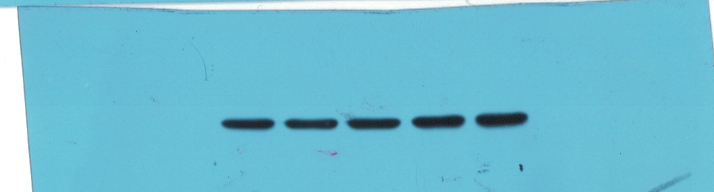

Supplement: S1 Data — (ZIP) [file ppat.1012546.s005.zip › Figure1-4, 5A-C, 6A-C, 6E, 6H-M and 7B-L. zip/Fig1/B/1/WCL-NLRP3.tif]

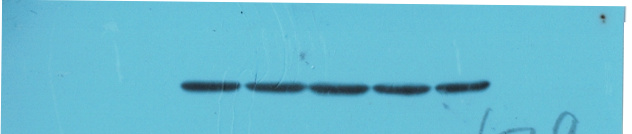

Supplement: S1 Data — (ZIP) [file ppat.1012546.s005.zip › Figure1-4, 5A-C, 6A-C, 6E, 6H-M and 7B-L. zip/Fig1/B/1/WCL-Pro-CASP1.tif]

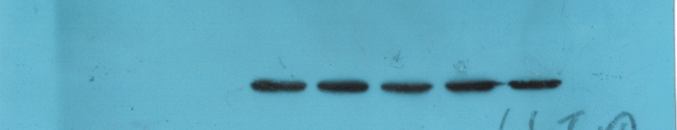

Supplement: S1 Data — (ZIP) [file ppat.1012546.s005.zip › Figure1-4, 5A-C, 6A-C, 6E, 6H-M and 7B-L. zip/Fig1/B/1/WCL-Pro-L-1a┬.tif]

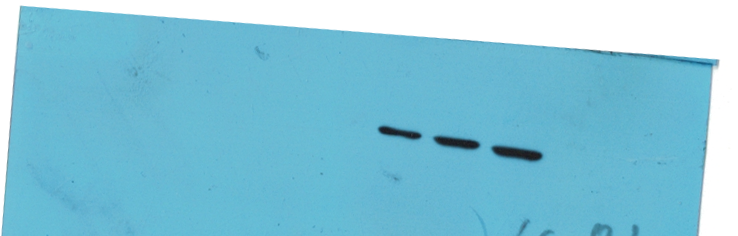

Supplement: S1 Data — (ZIP) [file ppat.1012546.s005.zip › Figure1-4, 5A-C, 6A-C, 6E, 6H-M and 7B-L. zip/Fig1/B/1/WCL-UL4.tif]

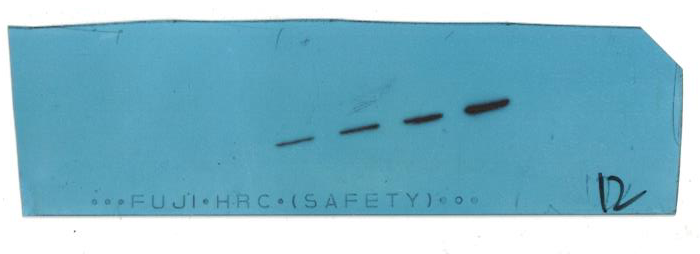

Supplement: S1 Data — (ZIP) [file ppat.1012546.s005.zip › Figure1-4, 5A-C, 6A-C, 6E, 6H-M and 7B-L. zip/Fig1/B/2/Sup CASP1 p10.tif]

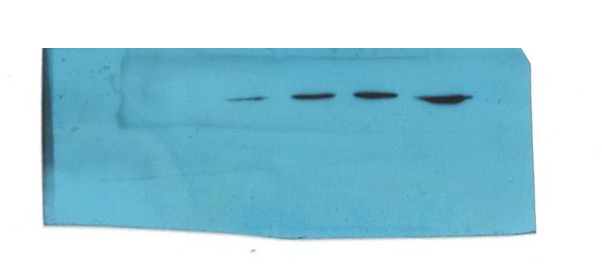

Supplement: S1 Data — (ZIP) [file ppat.1012546.s005.zip › Figure1-4, 5A-C, 6A-C, 6E, 6H-M and 7B-L. zip/Fig1/B/2/Sup-IL-1a┬ P17.tif]

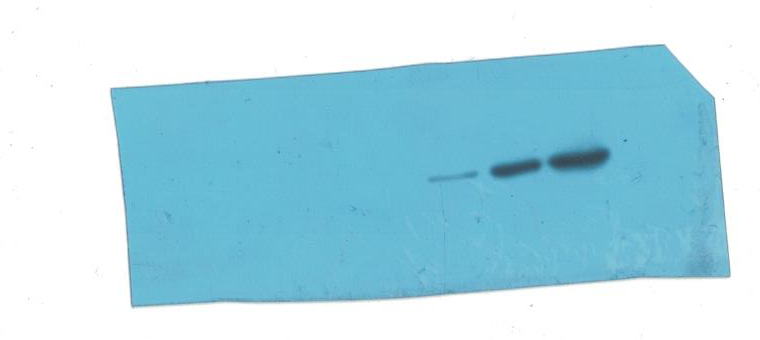

Supplement: S1 Data — (ZIP) [file ppat.1012546.s005.zip › Figure1-4, 5A-C, 6A-C, 6E, 6H-M and 7B-L. zip/Fig1/B/2/WCL UL4.tif]

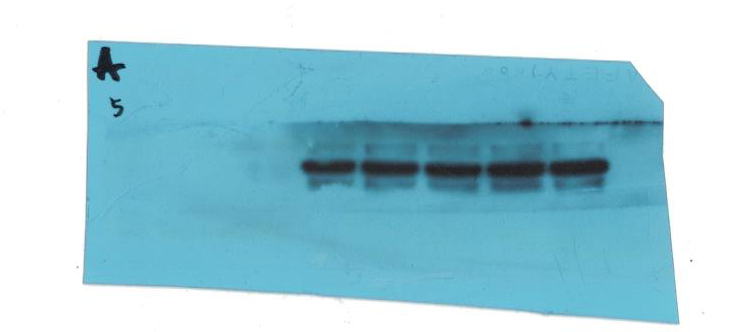

Supplement: S1 Data — (ZIP) [file ppat.1012546.s005.zip › Figure1-4, 5A-C, 6A-C, 6E, 6H-M and 7B-L. zip/Fig1/B/2/WCL-Actin.tif]

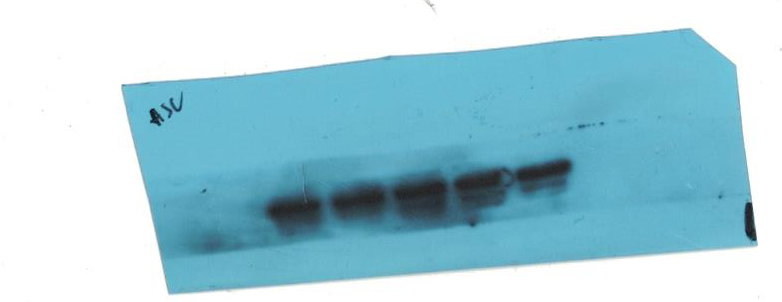

Supplement: S1 Data — (ZIP) [file ppat.1012546.s005.zip › Figure1-4, 5A-C, 6A-C, 6E, 6H-M and 7B-L. zip/Fig1/B/2/WCL-ASC.tif]

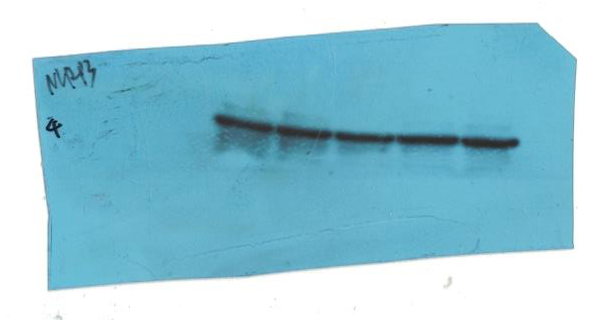

Supplement: S1 Data — (ZIP) [file ppat.1012546.s005.zip › Figure1-4, 5A-C, 6A-C, 6E, 6H-M and 7B-L. zip/Fig1/B/2/WCL-NLRP3.tif]

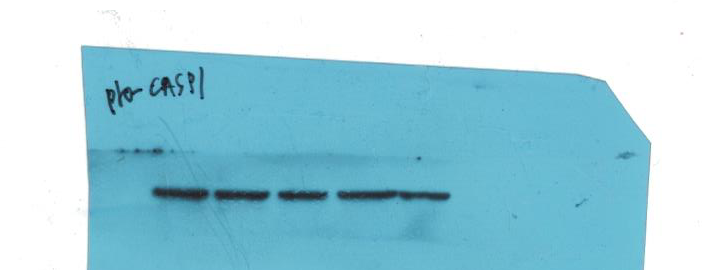

Supplement: S1 Data — (ZIP) [file ppat.1012546.s005.zip › Figure1-4, 5A-C, 6A-C, 6E, 6H-M and 7B-L. zip/Fig1/B/2/WCL-Pro-CASP1.tif]

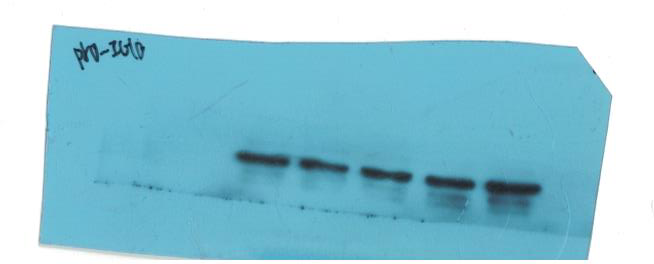

Supplement: S1 Data — (ZIP) [file ppat.1012546.s005.zip › Figure1-4, 5A-C, 6A-C, 6E, 6H-M and 7B-L. zip/Fig1/B/2/WCL-Pro-IL-1a┬.tif]

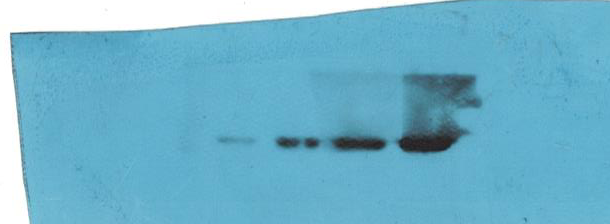

Supplement: S1 Data — (ZIP) [file ppat.1012546.s005.zip › Figure1-4, 5A-C, 6A-C, 6E, 6H-M and 7B-L. zip/Fig1/B/3/Sup-CASP1 p10.tif]

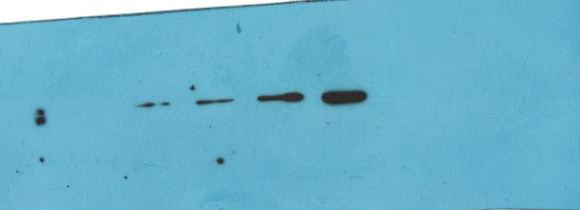

Supplement: S1 Data — (ZIP) [file ppat.1012546.s005.zip › Figure1-4, 5A-C, 6A-C, 6E, 6H-M and 7B-L. zip/Fig1/B/3/Sup-IL-1a┬ p17.tif]

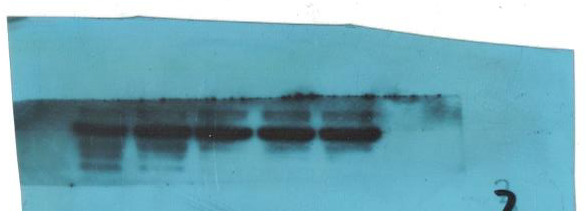

Supplement: S1 Data — (ZIP) [file ppat.1012546.s005.zip › Figure1-4, 5A-C, 6A-C, 6E, 6H-M and 7B-L. zip/Fig1/B/3/WCL-Actin.tif]

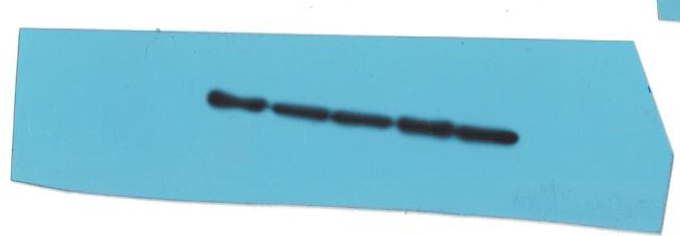

Supplement: S1 Data — (ZIP) [file ppat.1012546.s005.zip › Figure1-4, 5A-C, 6A-C, 6E, 6H-M and 7B-L. zip/Fig1/B/3/wcl-asc.tif]

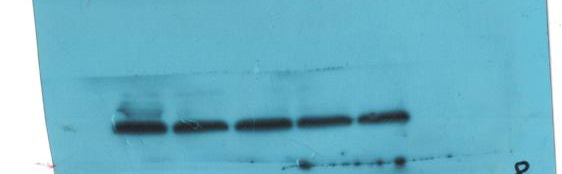

Supplement: S1 Data — (ZIP) [file ppat.1012546.s005.zip › Figure1-4, 5A-C, 6A-C, 6E, 6H-M and 7B-L. zip/Fig1/B/3/WCL-NLRP3.tif]

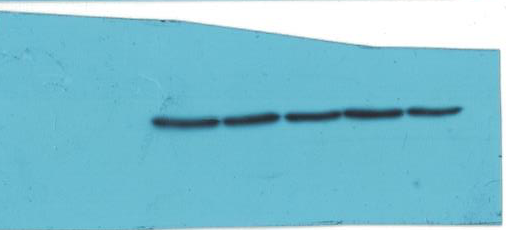

Supplement: S1 Data — (ZIP) [file ppat.1012546.s005.zip › Figure1-4, 5A-C, 6A-C, 6E, 6H-M and 7B-L. zip/Fig1/B/3/WCL-Pro-CASP1.tif]

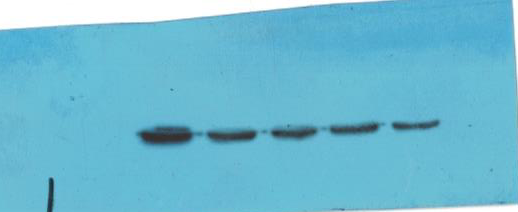

Supplement: S1 Data — (ZIP) [file ppat.1012546.s005.zip › Figure1-4, 5A-C, 6A-C, 6E, 6H-M and 7B-L. zip/Fig1/B/3/wcl-Pro-IL-1a┬.tif]

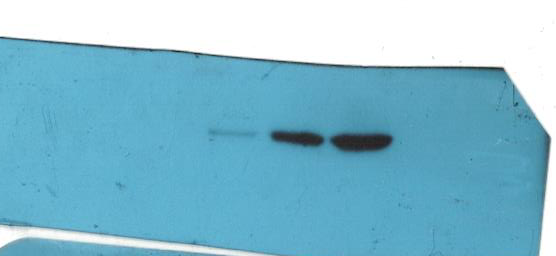

Supplement: S1 Data — (ZIP) [file ppat.1012546.s005.zip › Figure1-4, 5A-C, 6A-C, 6E, 6H-M and 7B-L. zip/Fig1/B/3/WCL-UL4.tif]

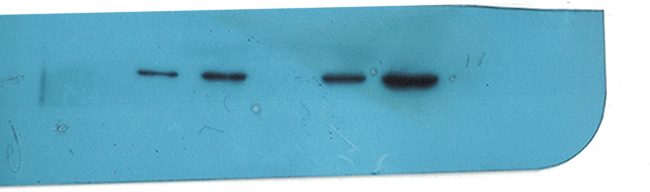

Supplement: S1 Data — (ZIP) [file ppat.1012546.s005.zip › Figure1-4, 5A-C, 6A-C, 6E, 6H-M and 7B-L. zip/Fig1/D/1/Sup-CASP1 p10.tif]

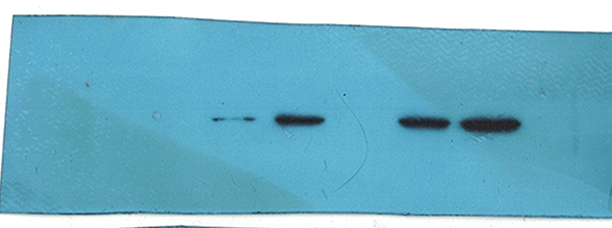

Supplement: S1 Data — (ZIP) [file ppat.1012546.s005.zip › Figure1-4, 5A-C, 6A-C, 6E, 6H-M and 7B-L. zip/Fig1/D/1/Sup-IL-1a┬ p17.tif]

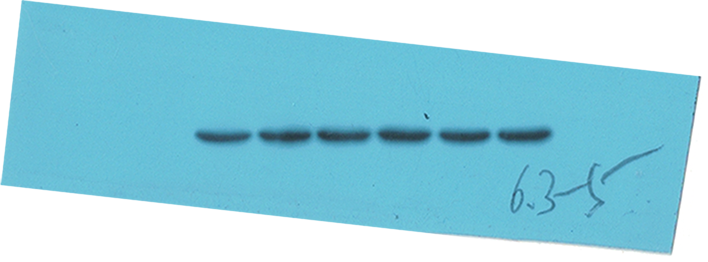

Supplement: S1 Data — (ZIP) [file ppat.1012546.s005.zip › Figure1-4, 5A-C, 6A-C, 6E, 6H-M and 7B-L. zip/Fig1/D/1/WCL-Actin.tif]

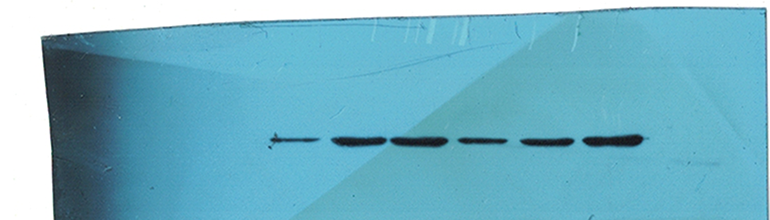

Supplement: S1 Data — (ZIP) [file ppat.1012546.s005.zip › Figure1-4, 5A-C, 6A-C, 6E, 6H-M and 7B-L. zip/Fig1/D/1/WCL-ASC.tif]

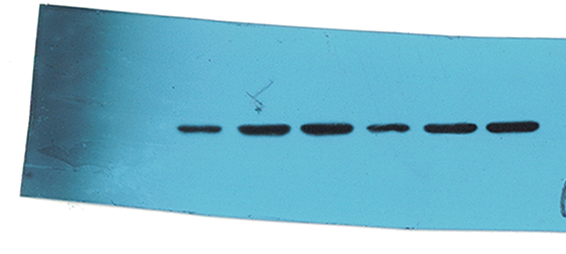

Supplement: S1 Data — (ZIP) [file ppat.1012546.s005.zip › Figure1-4, 5A-C, 6A-C, 6E, 6H-M and 7B-L. zip/Fig1/D/1/WCL-NLRP3.tif]

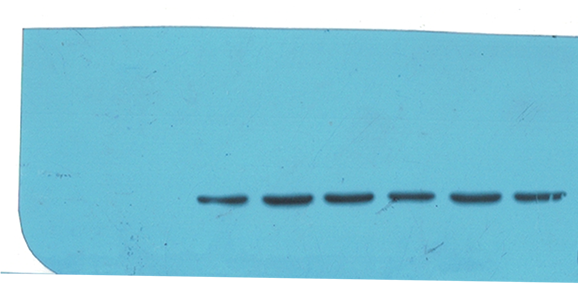

Supplement: S1 Data — (ZIP) [file ppat.1012546.s005.zip › Figure1-4, 5A-C, 6A-C, 6E, 6H-M and 7B-L. zip/Fig1/D/1/WCL-Pro-CASP1.tif]

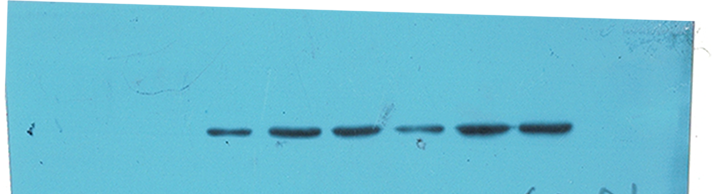

Supplement: S1 Data — (ZIP) [file ppat.1012546.s005.zip › Figure1-4, 5A-C, 6A-C, 6E, 6H-M and 7B-L. zip/Fig1/D/1/WCL-Pro-IL-1a┬.tif]

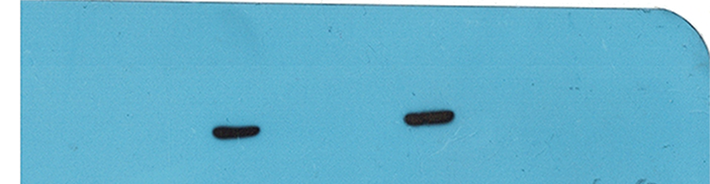

Supplement: S1 Data — (ZIP) [file ppat.1012546.s005.zip › Figure1-4, 5A-C, 6A-C, 6E, 6H-M and 7B-L. zip/Fig1/D/1/WCL-UL4.tif]

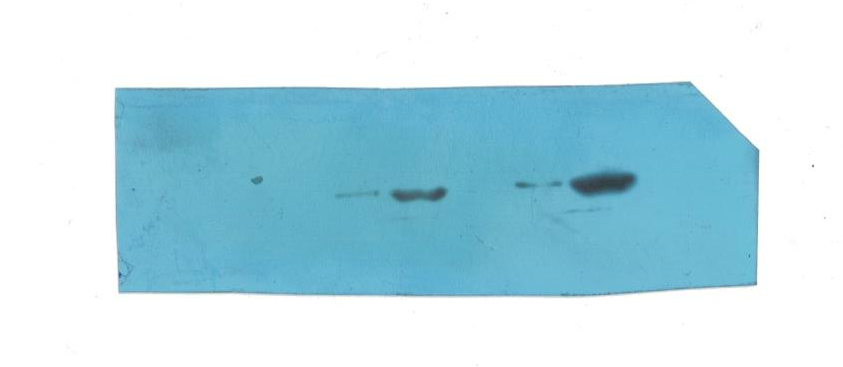

Supplement: S1 Data — (ZIP) [file ppat.1012546.s005.zip › Figure1-4, 5A-C, 6A-C, 6E, 6H-M and 7B-L. zip/Fig1/D/2/Sup-CASP1- p10.tif]

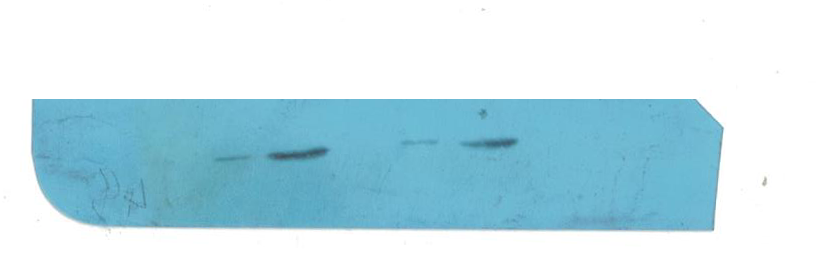

Supplement: S1 Data — (ZIP) [file ppat.1012546.s005.zip › Figure1-4, 5A-C, 6A-C, 6E, 6H-M and 7B-L. zip/Fig1/D/2/Sup-IL-1a┬ p17.tif]

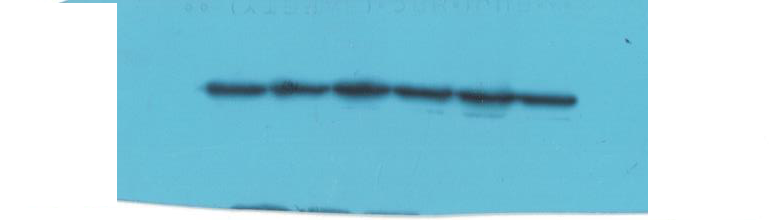

Supplement: S1 Data — (ZIP) [file ppat.1012546.s005.zip › Figure1-4, 5A-C, 6A-C, 6E, 6H-M and 7B-L. zip/Fig1/D/2/WCL-Actin.tif]

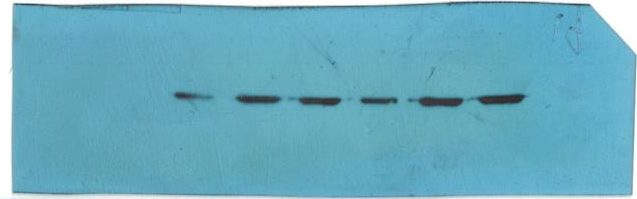

Supplement: S1 Data — (ZIP) [file ppat.1012546.s005.zip › Figure1-4, 5A-C, 6A-C, 6E, 6H-M and 7B-L. zip/Fig1/D/2/WCL-ASC.tif]

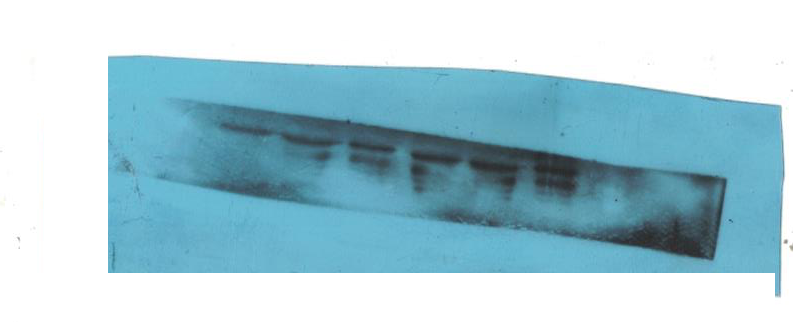

Supplement: S1 Data — (ZIP) [file ppat.1012546.s005.zip › Figure1-4, 5A-C, 6A-C, 6E, 6H-M and 7B-L. zip/Fig1/D/2/WCL-NLRP3.tif]

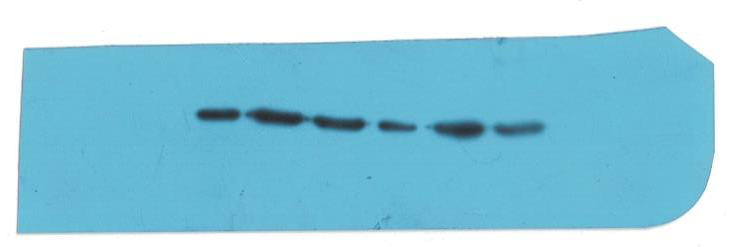

Supplement: S1 Data — (ZIP) [file ppat.1012546.s005.zip › Figure1-4, 5A-C, 6A-C, 6E, 6H-M and 7B-L. zip/Fig1/D/2/WCL-Pro-CASP1.tif]

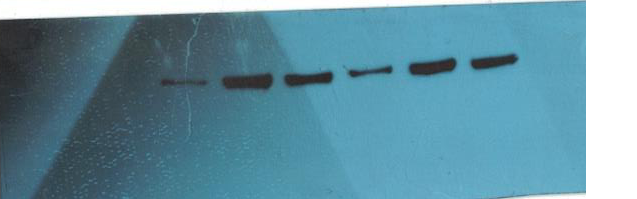

Supplement: S1 Data — (ZIP) [file ppat.1012546.s005.zip › Figure1-4, 5A-C, 6A-C, 6E, 6H-M and 7B-L. zip/Fig1/D/2/WCL-Pro-IL-1a┬.tif]

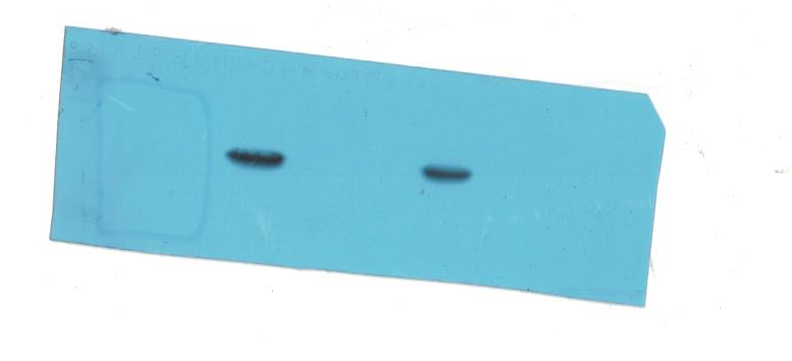

Supplement: S1 Data — (ZIP) [file ppat.1012546.s005.zip › Figure1-4, 5A-C, 6A-C, 6E, 6H-M and 7B-L. zip/Fig1/D/2/WCL-UL4.tif]

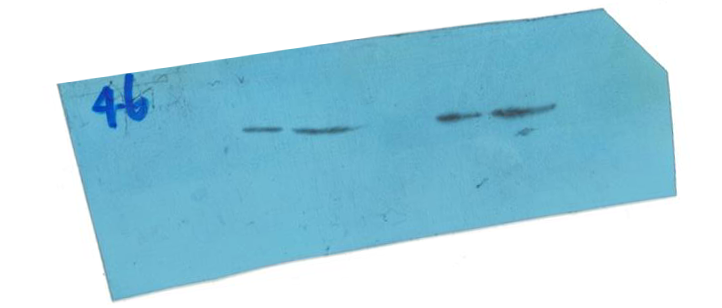

Supplement: S1 Data — (ZIP) [file ppat.1012546.s005.zip › Figure1-4, 5A-C, 6A-C, 6E, 6H-M and 7B-L. zip/Fig1/D/3/Sup-CASP1 p10.tif]

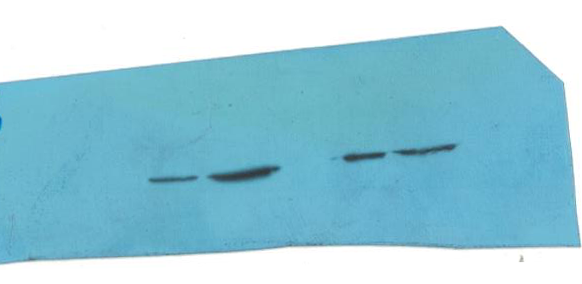

Supplement: S1 Data — (ZIP) [file ppat.1012546.s005.zip › Figure1-4, 5A-C, 6A-C, 6E, 6H-M and 7B-L. zip/Fig1/D/3/Sup-IL-1a┬ p17.tif]

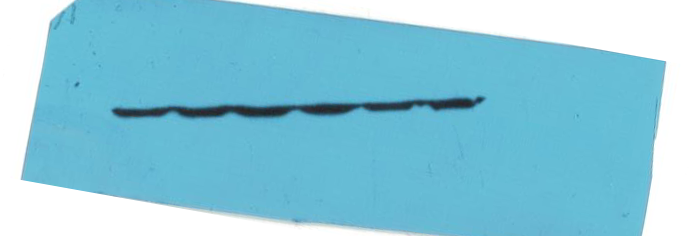

Supplement: S1 Data — (ZIP) [file ppat.1012546.s005.zip › Figure1-4, 5A-C, 6A-C, 6E, 6H-M and 7B-L. zip/Fig1/D/3/WCL-Actin.tif]

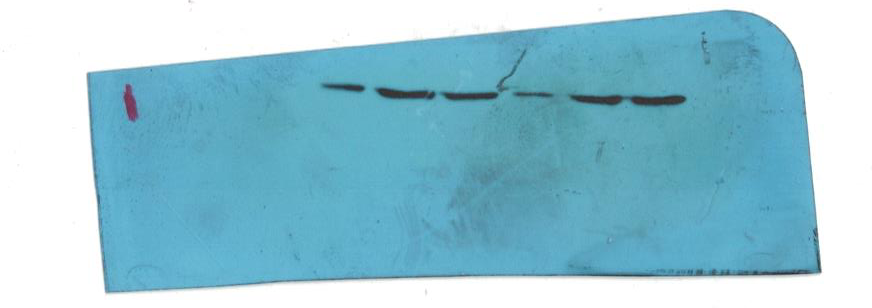

Supplement: S1 Data — (ZIP) [file ppat.1012546.s005.zip › Figure1-4, 5A-C, 6A-C, 6E, 6H-M and 7B-L. zip/Fig1/D/3/WCL-ASC.tif]

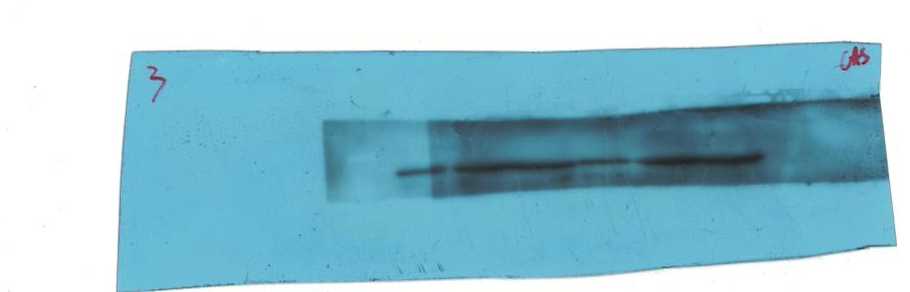

Supplement: S1 Data — (ZIP) [file ppat.1012546.s005.zip › Figure1-4, 5A-C, 6A-C, 6E, 6H-M and 7B-L. zip/Fig1/D/3/WCL-NLR3.tif]

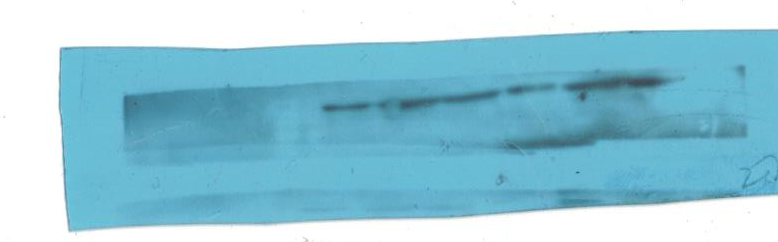

Supplement: S1 Data — (ZIP) [file ppat.1012546.s005.zip › Figure1-4, 5A-C, 6A-C, 6E, 6H-M and 7B-L. zip/Fig1/D/3/WCL-Pro-CASP1.tif]

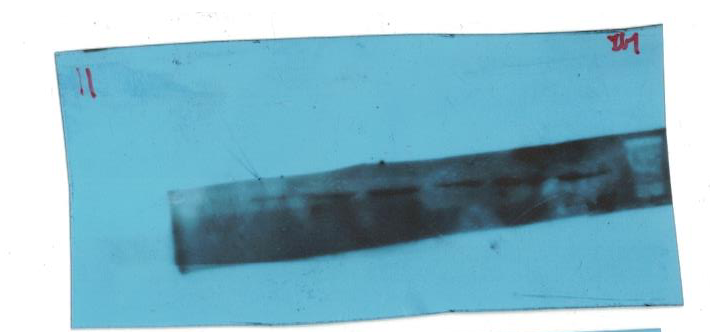

Supplement: S1 Data — (ZIP) [file ppat.1012546.s005.zip › Figure1-4, 5A-C, 6A-C, 6E, 6H-M and 7B-L. zip/Fig1/D/3/WCL-Pro-IL-1a┬.tif]

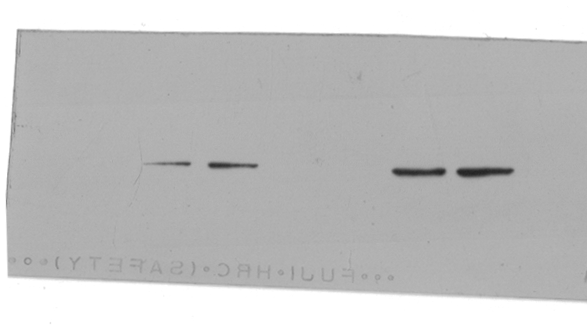

Supplement: S1 Data — (ZIP) [file ppat.1012546.s005.zip › Figure1-4, 5A-C, 6A-C, 6E, 6H-M and 7B-L. zip/Fig1/G/1/Sup-CASP1 P10.tif]

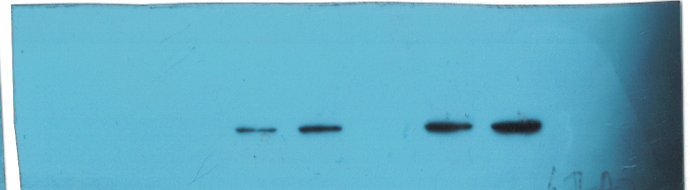

Supplement: S1 Data — (ZIP) [file ppat.1012546.s005.zip › Figure1-4, 5A-C, 6A-C, 6E, 6H-M and 7B-L. zip/Fig1/G/1/Sup-IL-1a┬ P17.tif]

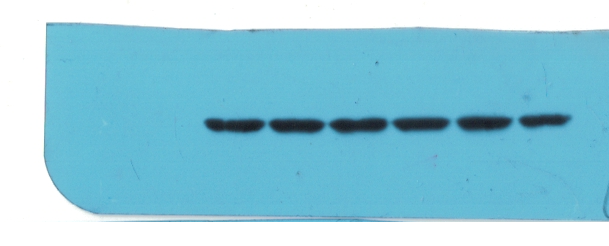

Supplement: S1 Data — (ZIP) [file ppat.1012546.s005.zip › Figure1-4, 5A-C, 6A-C, 6E, 6H-M and 7B-L. zip/Fig1/G/1/WCL-Actin.tif]

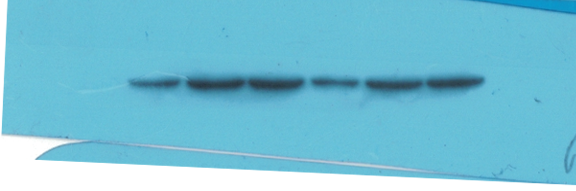

Supplement: S1 Data — (ZIP) [file ppat.1012546.s005.zip › Figure1-4, 5A-C, 6A-C, 6E, 6H-M and 7B-L. zip/Fig1/G/1/WCL-NLRP3.tif]

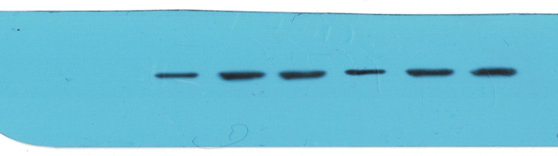

Supplement: S1 Data — (ZIP) [file ppat.1012546.s005.zip › Figure1-4, 5A-C, 6A-C, 6E, 6H-M and 7B-L. zip/Fig1/G/1/WCL-Pro-CASP1tif.tif]

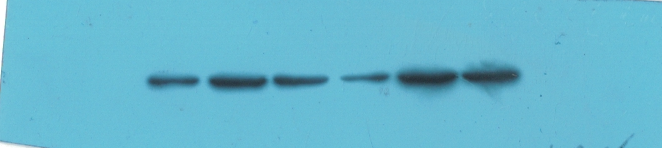

Supplement: S1 Data — (ZIP) [file ppat.1012546.s005.zip › Figure1-4, 5A-C, 6A-C, 6E, 6H-M and 7B-L. zip/Fig1/G/1/WCL-Pro-IL-1a┬.tif]

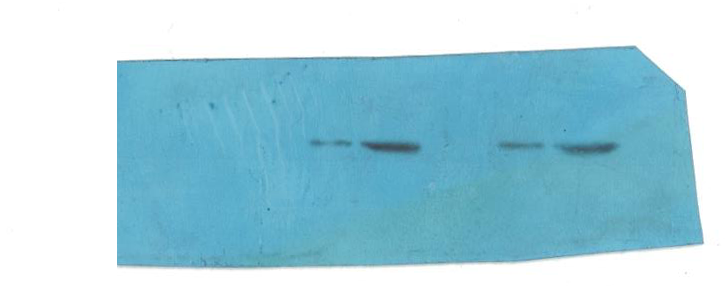

Supplement: S1 Data — (ZIP) [file ppat.1012546.s005.zip › Figure1-4, 5A-C, 6A-C, 6E, 6H-M and 7B-L. zip/Fig1/G/2/Sup-CASP1 p10.tif]

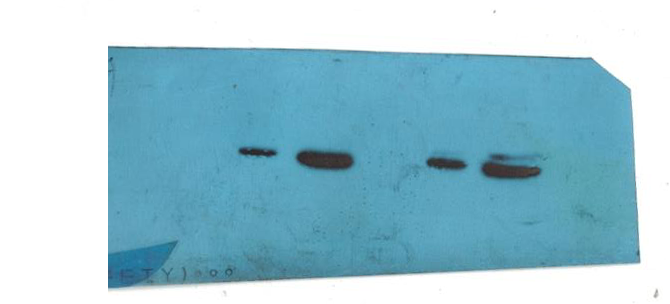

Supplement: S1 Data — (ZIP) [file ppat.1012546.s005.zip › Figure1-4, 5A-C, 6A-C, 6E, 6H-M and 7B-L. zip/Fig1/G/2/Sup-CASP1 p17.tif]

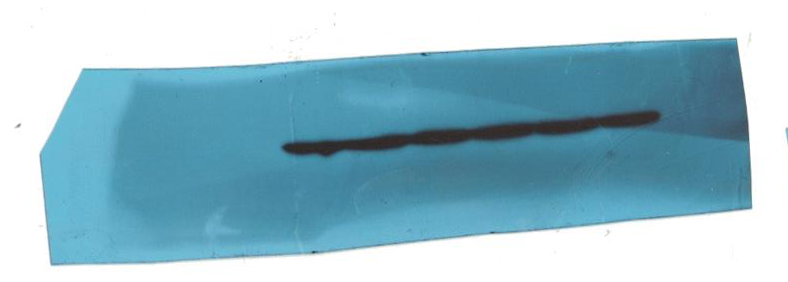

Supplement: S1 Data — (ZIP) [file ppat.1012546.s005.zip › Figure1-4, 5A-C, 6A-C, 6E, 6H-M and 7B-L. zip/Fig1/G/2/WCL-Actin.tif]

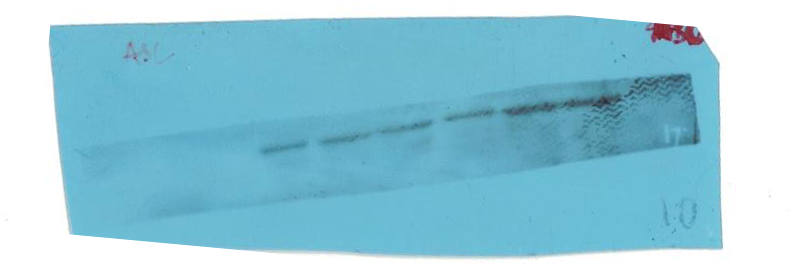

Supplement: S1 Data — (ZIP) [file ppat.1012546.s005.zip › Figure1-4, 5A-C, 6A-C, 6E, 6H-M and 7B-L. zip/Fig1/G/2/WCL-ASC.tif]

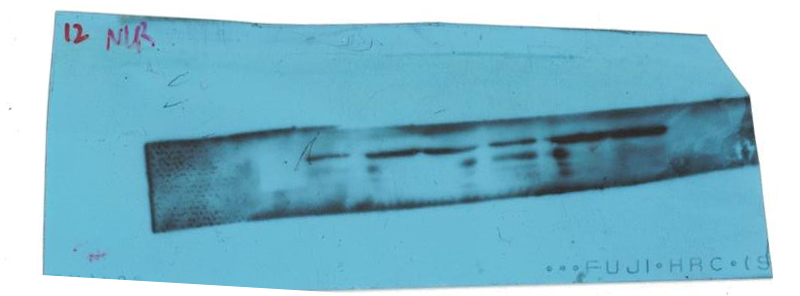

Supplement: S1 Data — (ZIP) [file ppat.1012546.s005.zip › Figure1-4, 5A-C, 6A-C, 6E, 6H-M and 7B-L. zip/Fig1/G/2/WCL-NLRP3.tif]

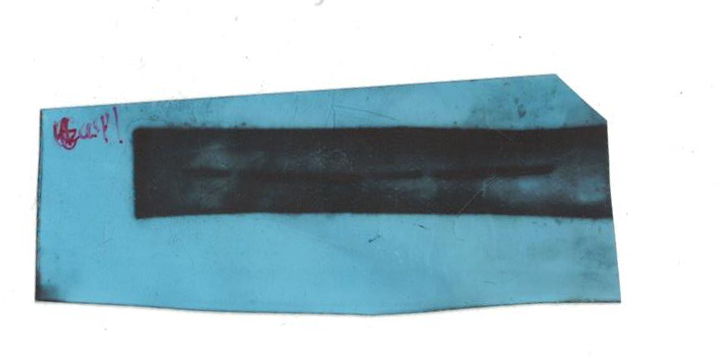

Supplement: S1 Data — (ZIP) [file ppat.1012546.s005.zip › Figure1-4, 5A-C, 6A-C, 6E, 6H-M and 7B-L. zip/Fig1/G/2/WCL-Pro-CASP1.tif]

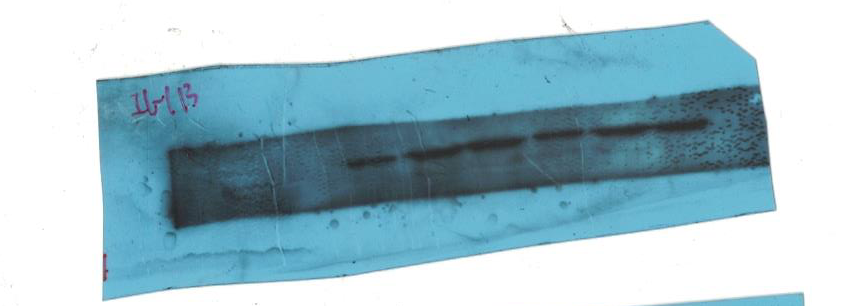

Supplement: S1 Data — (ZIP) [file ppat.1012546.s005.zip › Figure1-4, 5A-C, 6A-C, 6E, 6H-M and 7B-L. zip/Fig1/G/2/WCL-Pro-IL-1a┬.tif]

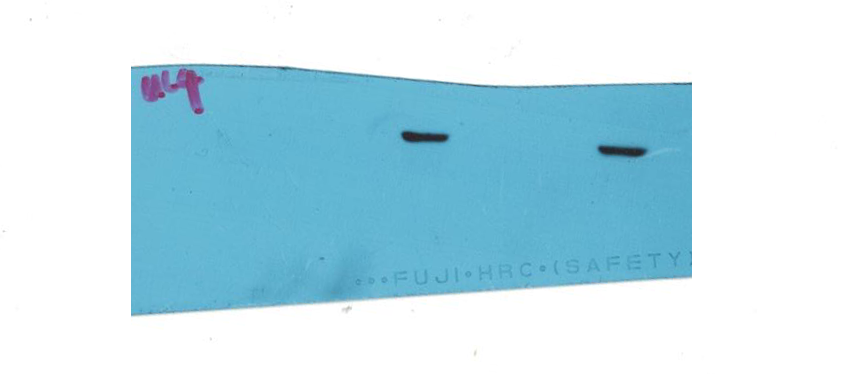

Supplement: S1 Data — (ZIP) [file ppat.1012546.s005.zip › Figure1-4, 5A-C, 6A-C, 6E, 6H-M and 7B-L. zip/Fig1/G/2/WCL-UL4.tif]

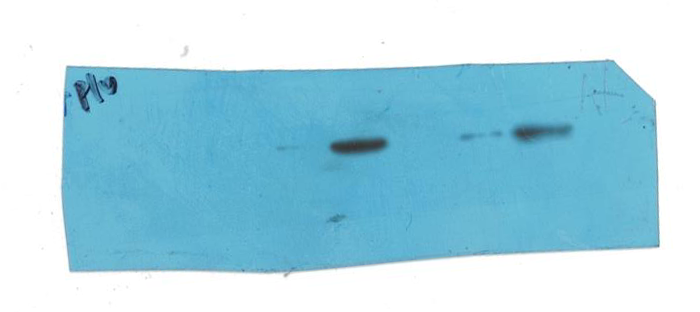

Supplement: S1 Data — (ZIP) [file ppat.1012546.s005.zip › Figure1-4, 5A-C, 6A-C, 6E, 6H-M and 7B-L. zip/Fig1/G/3/Sup CASP1 p10.tif]

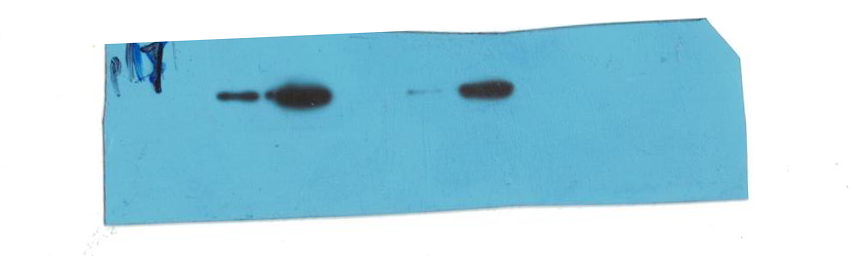

Supplement: S1 Data — (ZIP) [file ppat.1012546.s005.zip › Figure1-4, 5A-C, 6A-C, 6E, 6H-M and 7B-L. zip/Fig1/G/3/Sup IL-1a┬ p17.tif]

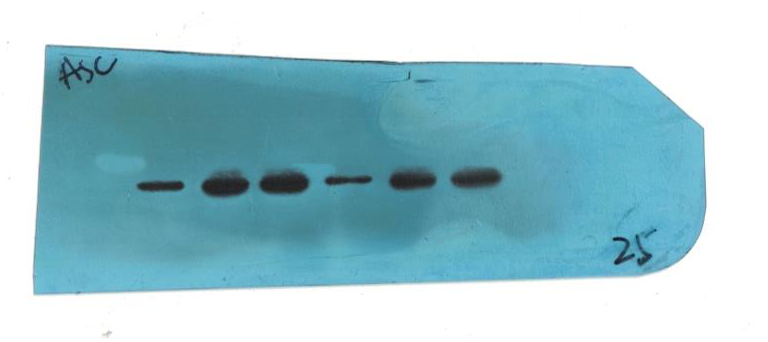

Supplement: S1 Data — (ZIP) [file ppat.1012546.s005.zip › Figure1-4, 5A-C, 6A-C, 6E, 6H-M and 7B-L. zip/Fig1/G/3/WCL-ASC.tif]

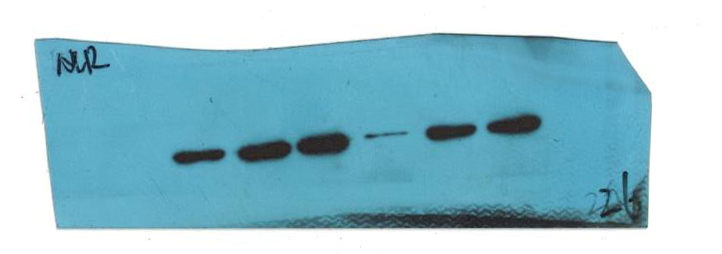

Supplement: S1 Data — (ZIP) [file ppat.1012546.s005.zip › Figure1-4, 5A-C, 6A-C, 6E, 6H-M and 7B-L. zip/Fig1/G/3/WCL-NLRP3.tif]

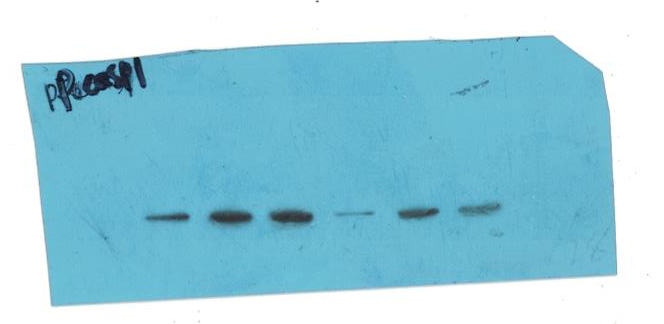

Supplement: S1 Data — (ZIP) [file ppat.1012546.s005.zip › Figure1-4, 5A-C, 6A-C, 6E, 6H-M and 7B-L. zip/Fig1/G/3/WCL-Pro-CASP1.tif]

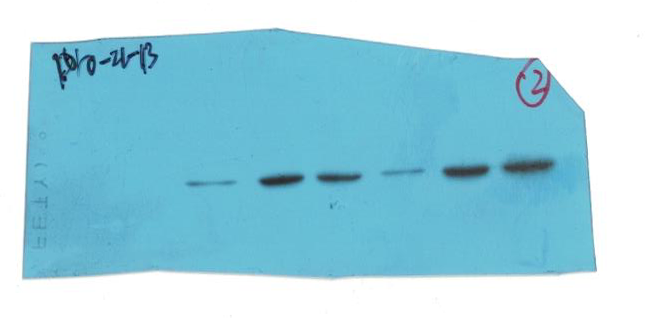

Supplement: S1 Data — (ZIP) [file ppat.1012546.s005.zip › Figure1-4, 5A-C, 6A-C, 6E, 6H-M and 7B-L. zip/Fig1/G/3/WCL-Pro-IL-1a┬.tif]

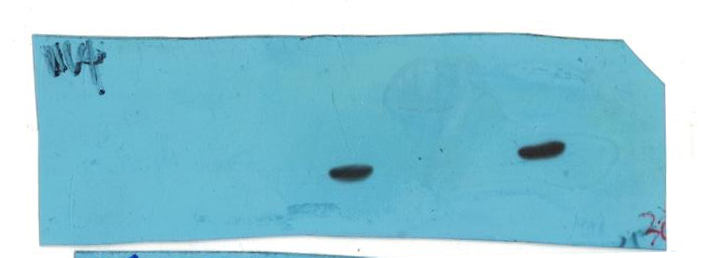

Supplement: S1 Data — (ZIP) [file ppat.1012546.s005.zip › Figure1-4, 5A-C, 6A-C, 6E, 6H-M and 7B-L. zip/Fig1/G/3/WCL-UL4.tif]

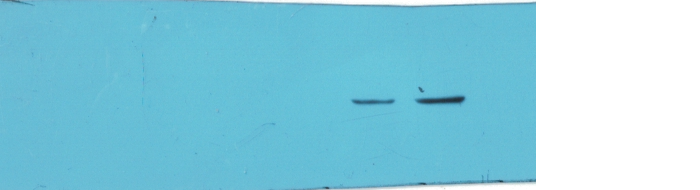

Supplement: S1 Data — (ZIP) [file ppat.1012546.s005.zip › Figure1-4, 5A-C, 6A-C, 6E, 6H-M and 7B-L. zip/Fig2/B/1/Sup-CASP1 P10.tif]

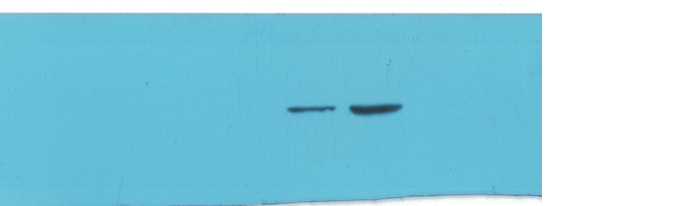

Supplement: S1 Data — (ZIP) [file ppat.1012546.s005.zip › Figure1-4, 5A-C, 6A-C, 6E, 6H-M and 7B-L. zip/Fig2/B/1/Sup-IL-1a┬ p17.tif]

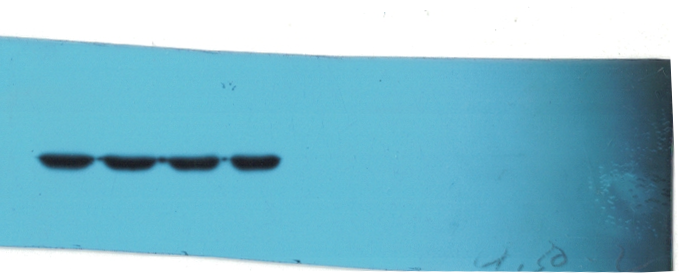

Supplement: S1 Data — (ZIP) [file ppat.1012546.s005.zip › Figure1-4, 5A-C, 6A-C, 6E, 6H-M and 7B-L. zip/Fig2/B/1/WCL-Actin.tif]

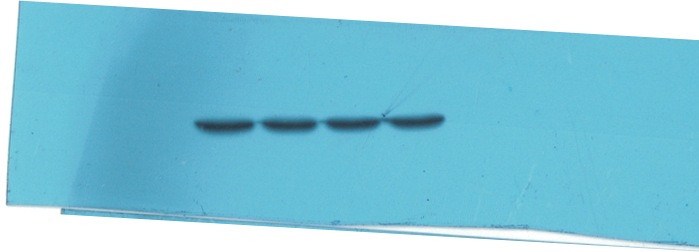

Supplement: S1 Data — (ZIP) [file ppat.1012546.s005.zip › Figure1-4, 5A-C, 6A-C, 6E, 6H-M and 7B-L. zip/Fig2/B/1/WCL-AIM2.tif]

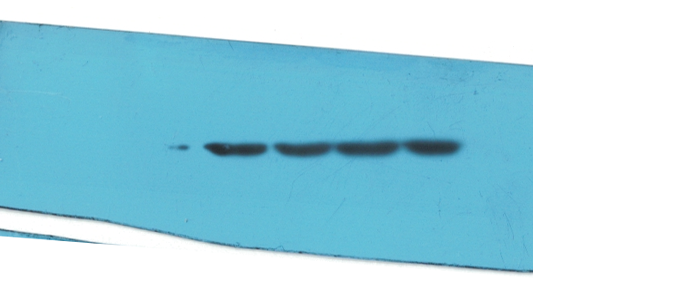

Supplement: S1 Data — (ZIP) [file ppat.1012546.s005.zip › Figure1-4, 5A-C, 6A-C, 6E, 6H-M and 7B-L. zip/Fig2/B/1/WCL-ASC.tif]

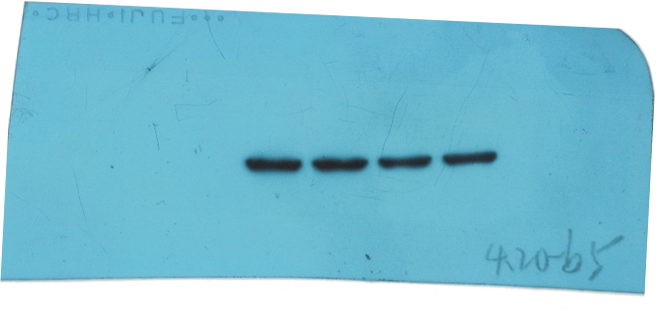

Supplement: S1 Data — (ZIP) [file ppat.1012546.s005.zip › Figure1-4, 5A-C, 6A-C, 6E, 6H-M and 7B-L. zip/Fig2/B/1/WCL-Pro-CASP1.tif]

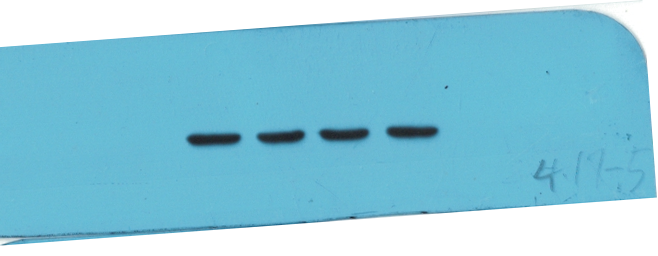

Supplement: S1 Data — (ZIP) [file ppat.1012546.s005.zip › Figure1-4, 5A-C, 6A-C, 6E, 6H-M and 7B-L. zip/Fig2/B/1/WCL-Pro-IL-1a┬.tif]

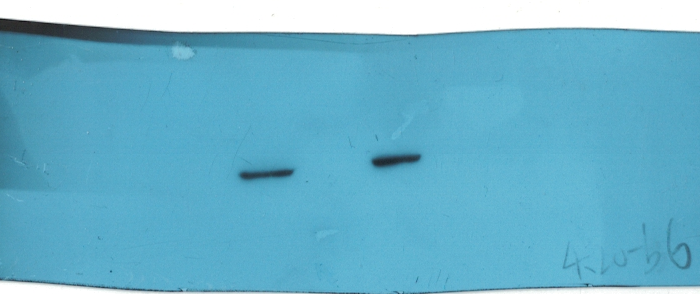

Supplement: S1 Data — (ZIP) [file ppat.1012546.s005.zip › Figure1-4, 5A-C, 6A-C, 6E, 6H-M and 7B-L. zip/Fig2/B/1/WCL-UL4.tif]

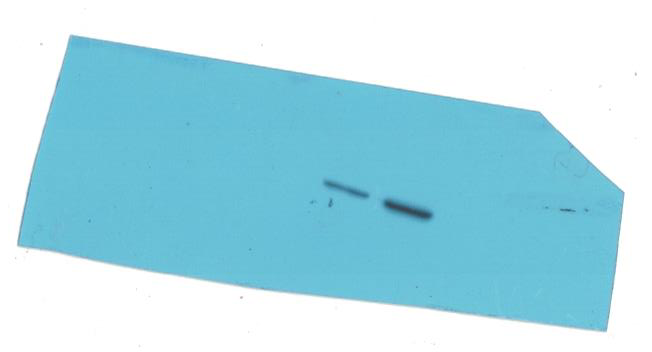

Supplement: S1 Data — (ZIP) [file ppat.1012546.s005.zip › Figure1-4, 5A-C, 6A-C, 6E, 6H-M and 7B-L. zip/Fig2/B/2/Sup-IL-1a┬ p17.tif]

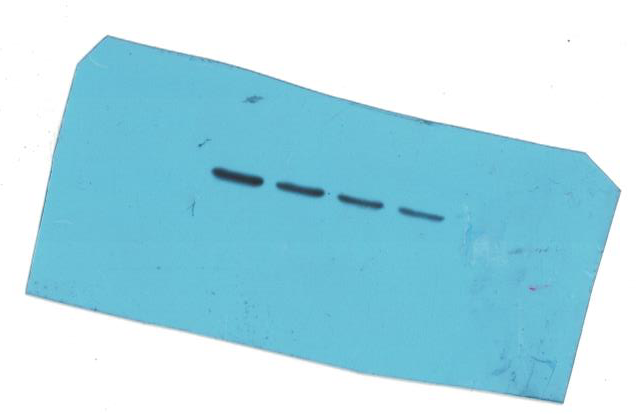

Supplement: S1 Data — (ZIP) [file ppat.1012546.s005.zip › Figure1-4, 5A-C, 6A-C, 6E, 6H-M and 7B-L. zip/Fig2/B/2/Sup-pro-IL-1a┬.tif]

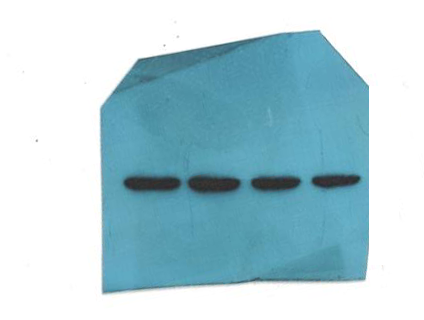

Supplement: S1 Data — (ZIP) [file ppat.1012546.s005.zip › Figure1-4, 5A-C, 6A-C, 6E, 6H-M and 7B-L. zip/Fig2/B/2/WCL-Actin.tif]

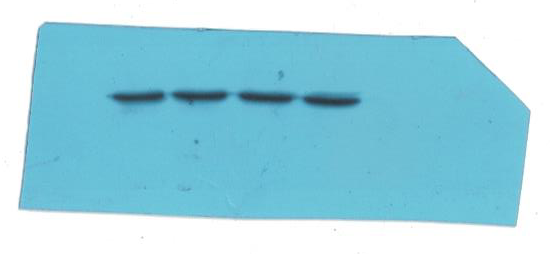

Supplement: S1 Data — (ZIP) [file ppat.1012546.s005.zip › Figure1-4, 5A-C, 6A-C, 6E, 6H-M and 7B-L. zip/Fig2/B/2/WCL-AIM2.tif]

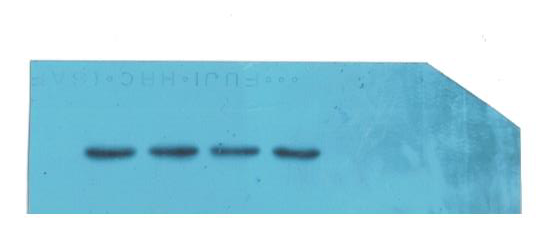

Supplement: S1 Data — (ZIP) [file ppat.1012546.s005.zip › Figure1-4, 5A-C, 6A-C, 6E, 6H-M and 7B-L. zip/Fig2/B/2/WCL-ASC.tif]

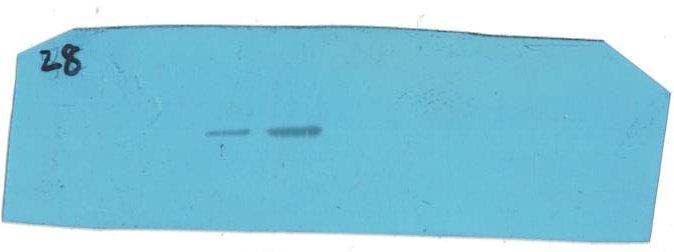

Supplement: S1 Data — (ZIP) [file ppat.1012546.s005.zip › Figure1-4, 5A-C, 6A-C, 6E, 6H-M and 7B-L. zip/Fig2/B/2/WCL-CASP1 P10.tif]

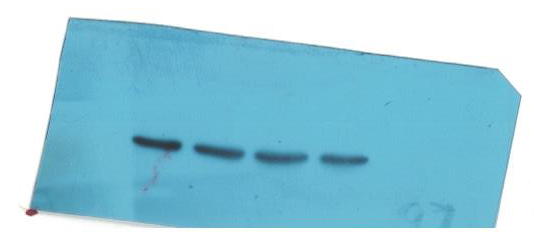

Supplement: S1 Data — (ZIP) [file ppat.1012546.s005.zip › Figure1-4, 5A-C, 6A-C, 6E, 6H-M and 7B-L. zip/Fig2/B/2/WCL-Pro-CASP1.tif]

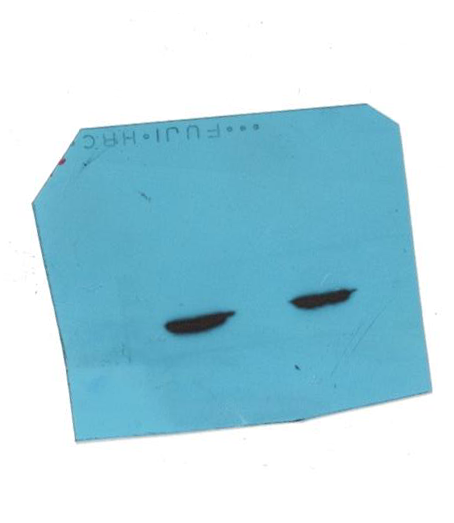

Supplement: S1 Data — (ZIP) [file ppat.1012546.s005.zip › Figure1-4, 5A-C, 6A-C, 6E, 6H-M and 7B-L. zip/Fig2/B/2/WCL-UL4.tif]

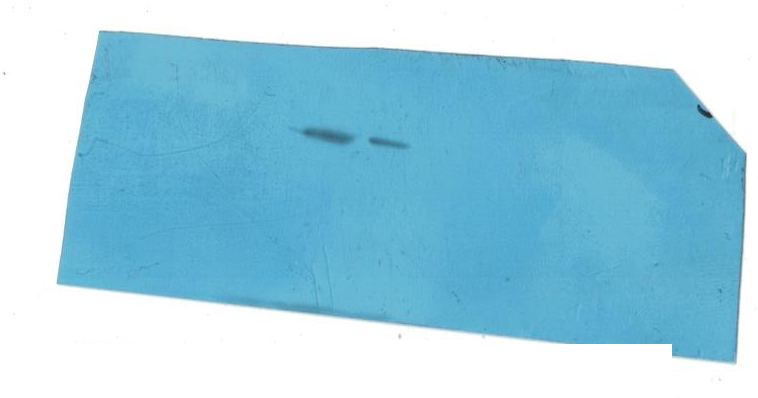

Supplement: S1 Data — (ZIP) [file ppat.1012546.s005.zip › Figure1-4, 5A-C, 6A-C, 6E, 6H-M and 7B-L. zip/Fig2/B/3/Sup-CASP1 P10.tif]

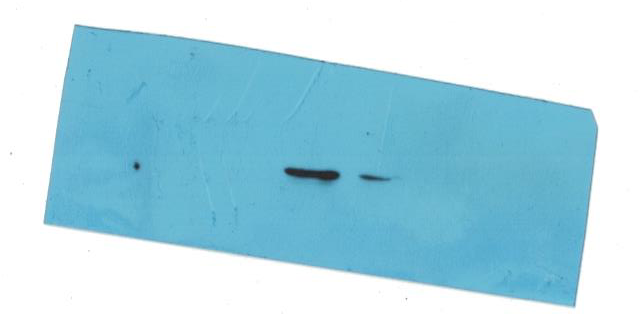

Supplement: S1 Data — (ZIP) [file ppat.1012546.s005.zip › Figure1-4, 5A-C, 6A-C, 6E, 6H-M and 7B-L. zip/Fig2/B/3/Sup-IL-1a┬ p17.tif]

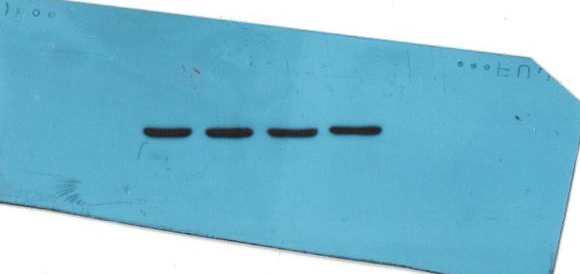

Supplement: S1 Data — (ZIP) [file ppat.1012546.s005.zip › Figure1-4, 5A-C, 6A-C, 6E, 6H-M and 7B-L. zip/Fig2/B/3/WCL-Actin.tif]

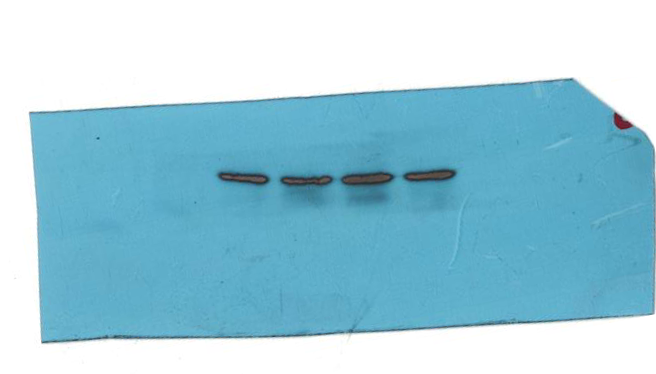

Supplement: S1 Data — (ZIP) [file ppat.1012546.s005.zip › Figure1-4, 5A-C, 6A-C, 6E, 6H-M and 7B-L. zip/Fig2/B/3/WCL-AIM2.tif]

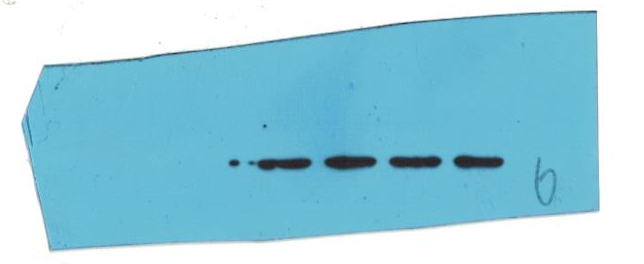

Supplement: S1 Data — (ZIP) [file ppat.1012546.s005.zip › Figure1-4, 5A-C, 6A-C, 6E, 6H-M and 7B-L. zip/Fig2/B/3/WCL-ASC.tif]

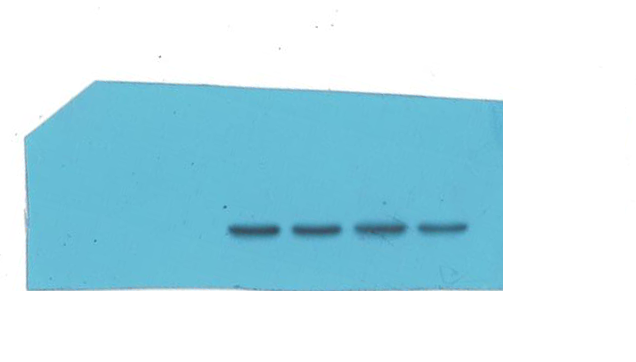

Supplement: S1 Data — (ZIP) [file ppat.1012546.s005.zip › Figure1-4, 5A-C, 6A-C, 6E, 6H-M and 7B-L. zip/Fig2/B/3/WCL-Pro-CASP1.tif]

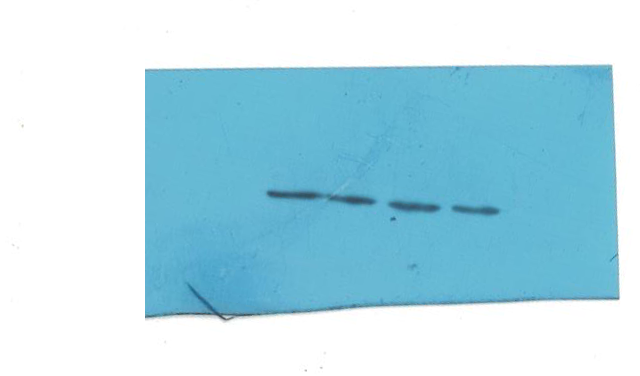

Supplement: S1 Data — (ZIP) [file ppat.1012546.s005.zip › Figure1-4, 5A-C, 6A-C, 6E, 6H-M and 7B-L. zip/Fig2/B/3/WCL-pro-IL-1a┬.tif]

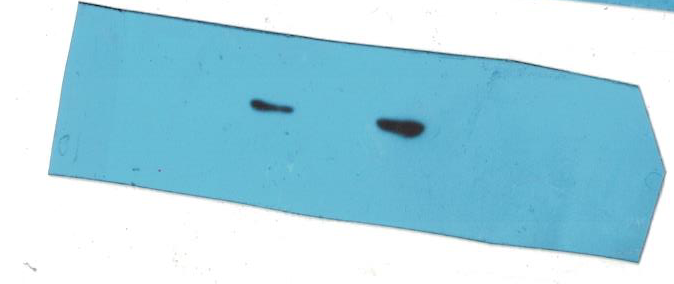

Supplement: S1 Data — (ZIP) [file ppat.1012546.s005.zip › Figure1-4, 5A-C, 6A-C, 6E, 6H-M and 7B-L. zip/Fig2/B/3/WCL-UL4.tif]

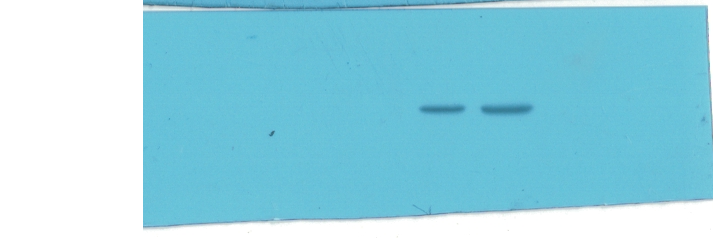

Supplement: S1 Data — (ZIP) [file ppat.1012546.s005.zip › Figure1-4, 5A-C, 6A-C, 6E, 6H-M and 7B-L. zip/Fig2/C/1/Sup-CASP1 P10.tif]

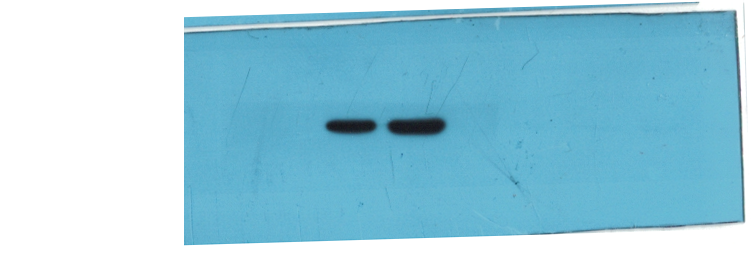

Supplement: S1 Data — (ZIP) [file ppat.1012546.s005.zip › Figure1-4, 5A-C, 6A-C, 6E, 6H-M and 7B-L. zip/Fig2/C/1/Sup-IL-1a┬ p17.tif]

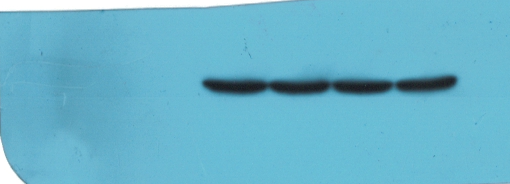

Supplement: S1 Data — (ZIP) [file ppat.1012546.s005.zip › Figure1-4, 5A-C, 6A-C, 6E, 6H-M and 7B-L. zip/Fig2/C/1/WCL-Actin.tif]

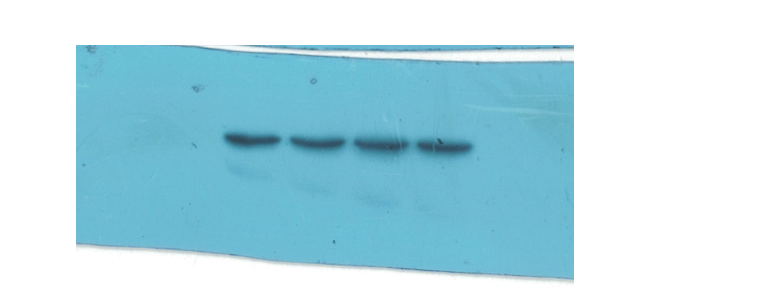

Supplement: S1 Data — (ZIP) [file ppat.1012546.s005.zip › Figure1-4, 5A-C, 6A-C, 6E, 6H-M and 7B-L. zip/Fig2/C/1/WCL-AIM2.tif]

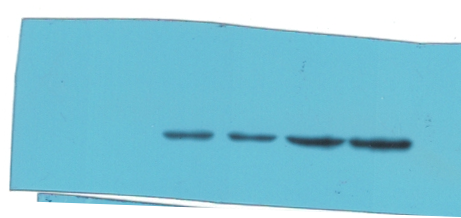

Supplement: S1 Data — (ZIP) [file ppat.1012546.s005.zip › Figure1-4, 5A-C, 6A-C, 6E, 6H-M and 7B-L. zip/Fig2/C/1/WCL-ASC.tif]
