# Supplementary figures and images for: Pseudorabies Virus UL4 protein promotes the ASC-dependent inflammasome activation and pyroptosis to exacerbate inflammation (part 2 of 6)
Source: PLoS Pathog. 2024 Sep 24;20(9):e1012546. doi: 10.1371/journal.ppat.1012546 (PMC11421794; doi:10.1371/journal.ppat.1012546)

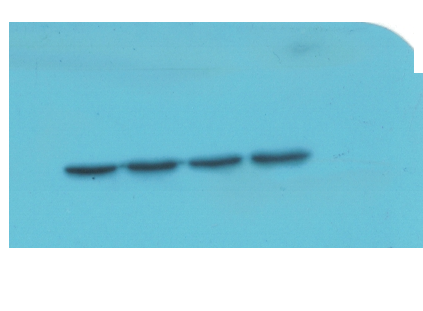

Supplement: S1 Data — (ZIP) [file ppat.1012546.s005.zip › Figure1-4, 5A-C, 6A-C, 6E, 6H-M and 7B-L. zip/Fig2/C/1/WCL-Pro-CASP1.tif]

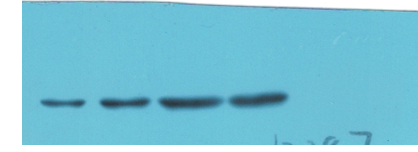

Supplement: S1 Data — (ZIP) [file ppat.1012546.s005.zip › Figure1-4, 5A-C, 6A-C, 6E, 6H-M and 7B-L. zip/Fig2/C/1/WCL-Pro-IL-1a┬.tif]

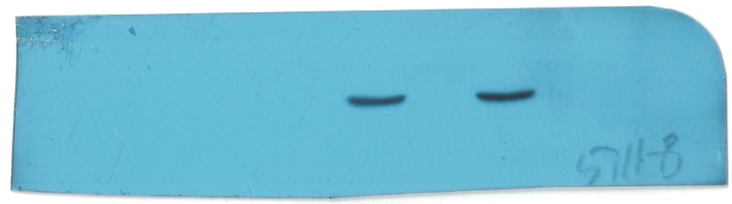

Supplement: S1 Data — (ZIP) [file ppat.1012546.s005.zip › Figure1-4, 5A-C, 6A-C, 6E, 6H-M and 7B-L. zip/Fig2/C/1/WCL-UL4.tif]

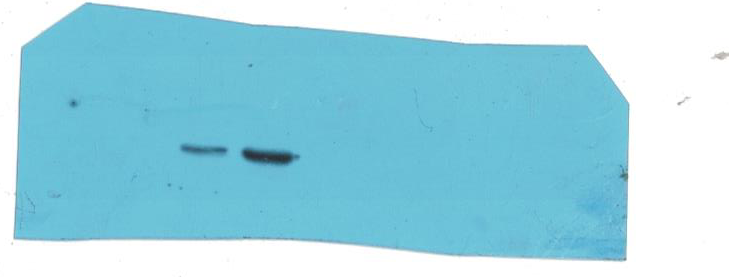

Supplement: S1 Data — (ZIP) [file ppat.1012546.s005.zip › Figure1-4, 5A-C, 6A-C, 6E, 6H-M and 7B-L. zip/Fig2/C/2/Sup-CASP1 p10.tif]

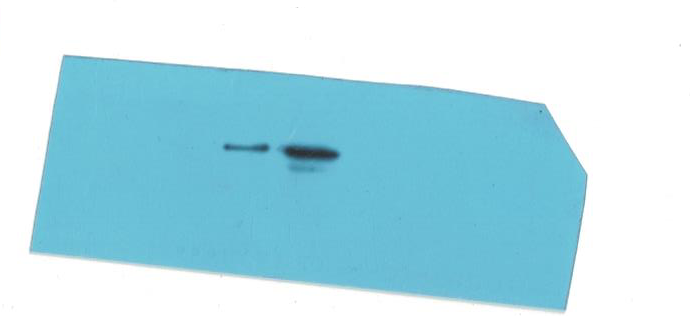

Supplement: S1 Data — (ZIP) [file ppat.1012546.s005.zip › Figure1-4, 5A-C, 6A-C, 6E, 6H-M and 7B-L. zip/Fig2/C/2/Sup-IL-1a┬ p17.tif]

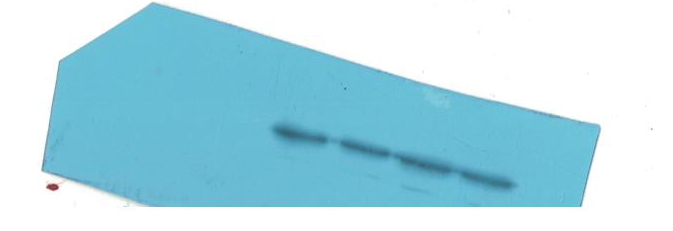

Supplement: S1 Data — (ZIP) [file ppat.1012546.s005.zip › Figure1-4, 5A-C, 6A-C, 6E, 6H-M and 7B-L. zip/Fig2/C/2/WCL-Actin.tif]

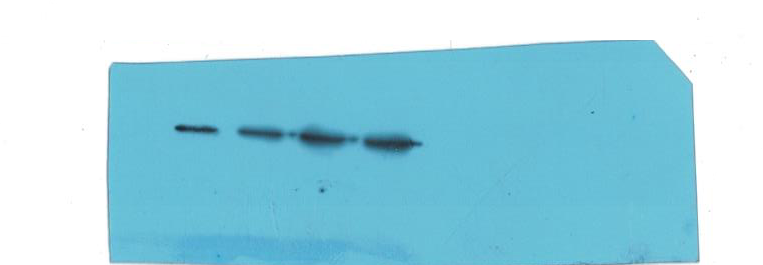

Supplement: S1 Data — (ZIP) [file ppat.1012546.s005.zip › Figure1-4, 5A-C, 6A-C, 6E, 6H-M and 7B-L. zip/Fig2/C/2/WCL-AIM2.tif]

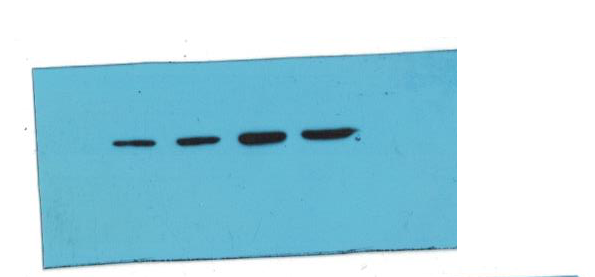

Supplement: S1 Data — (ZIP) [file ppat.1012546.s005.zip › Figure1-4, 5A-C, 6A-C, 6E, 6H-M and 7B-L. zip/Fig2/C/2/WCL-ASC.tif]

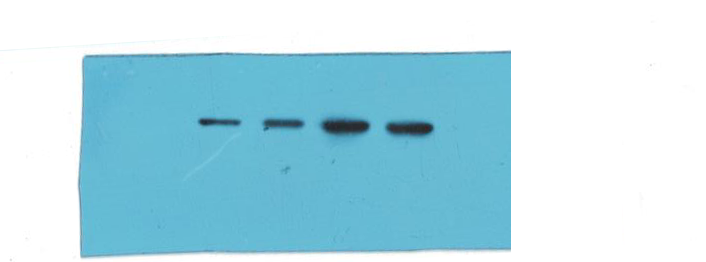

Supplement: S1 Data — (ZIP) [file ppat.1012546.s005.zip › Figure1-4, 5A-C, 6A-C, 6E, 6H-M and 7B-L. zip/Fig2/C/2/WCL-Pro-CASP1.tif]

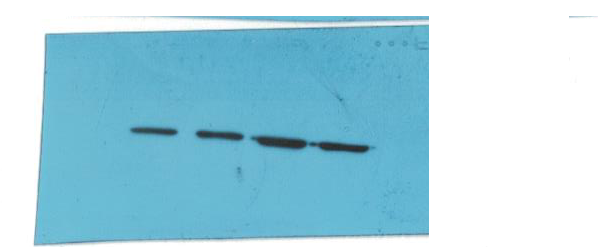

Supplement: S1 Data — (ZIP) [file ppat.1012546.s005.zip › Figure1-4, 5A-C, 6A-C, 6E, 6H-M and 7B-L. zip/Fig2/C/2/WCL-Pro-IL-1a┬.tif]

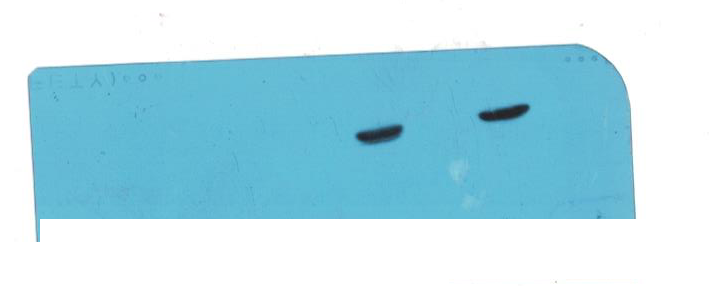

Supplement: S1 Data — (ZIP) [file ppat.1012546.s005.zip › Figure1-4, 5A-C, 6A-C, 6E, 6H-M and 7B-L. zip/Fig2/C/2/WCL-UL4.tif]

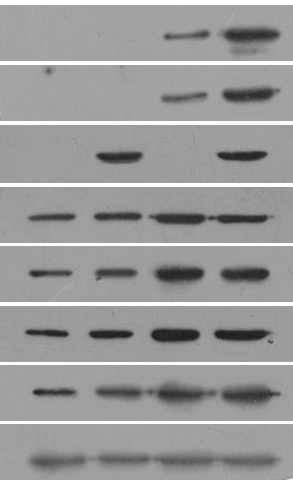

Supplement: S1 Data — (ZIP) [file ppat.1012546.s005.zip › Figure1-4, 5A-C, 6A-C, 6E, 6H-M and 7B-L. zip/Fig2/C/2/╬┤▒Ω╠Γ-1.tif]

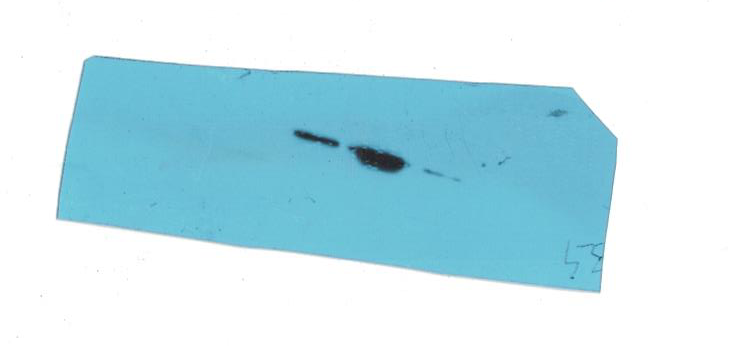

Supplement: S1 Data — (ZIP) [file ppat.1012546.s005.zip › Figure1-4, 5A-C, 6A-C, 6E, 6H-M and 7B-L. zip/Fig2/C/3/Sup-CASP1 p10.tif]

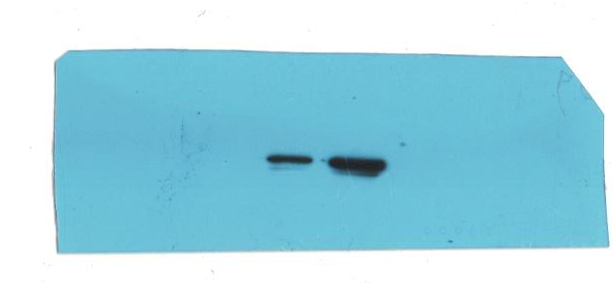

Supplement: S1 Data — (ZIP) [file ppat.1012546.s005.zip › Figure1-4, 5A-C, 6A-C, 6E, 6H-M and 7B-L. zip/Fig2/C/3/Sup-IL-1a┬ p17.tif]

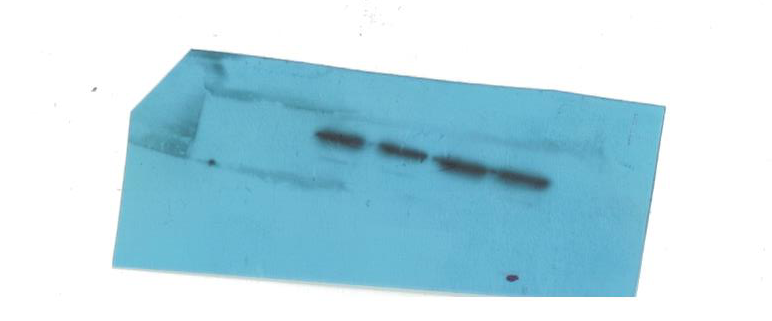

Supplement: S1 Data — (ZIP) [file ppat.1012546.s005.zip › Figure1-4, 5A-C, 6A-C, 6E, 6H-M and 7B-L. zip/Fig2/C/3/WCL-Actin.tif]

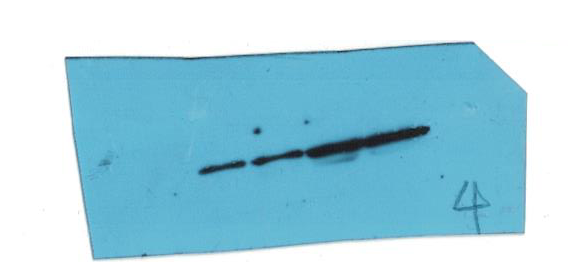

Supplement: S1 Data — (ZIP) [file ppat.1012546.s005.zip › Figure1-4, 5A-C, 6A-C, 6E, 6H-M and 7B-L. zip/Fig2/C/3/WCL-AIM2.tif]

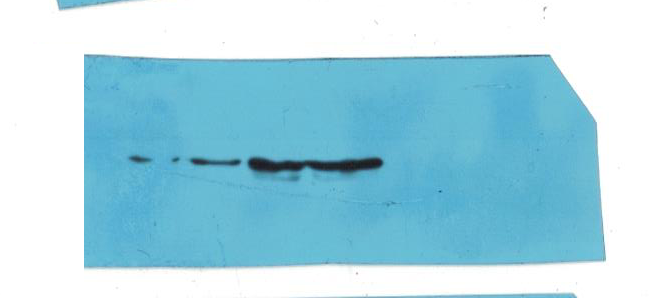

Supplement: S1 Data — (ZIP) [file ppat.1012546.s005.zip › Figure1-4, 5A-C, 6A-C, 6E, 6H-M and 7B-L. zip/Fig2/C/3/WCL-ASC.tif]

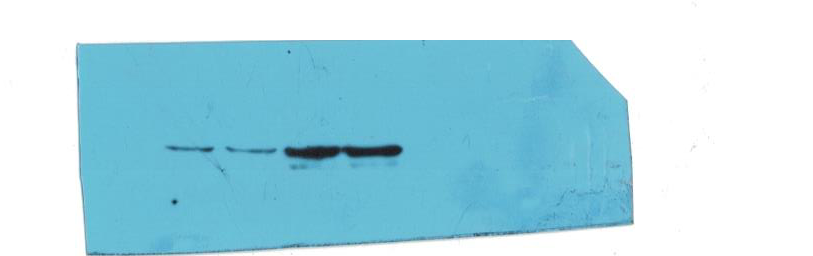

Supplement: S1 Data — (ZIP) [file ppat.1012546.s005.zip › Figure1-4, 5A-C, 6A-C, 6E, 6H-M and 7B-L. zip/Fig2/C/3/WCL-Pro-CASP1.tif]

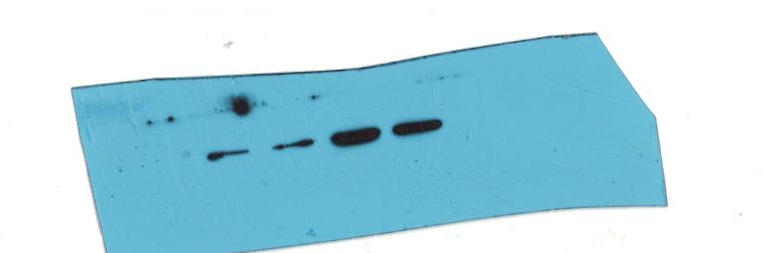

Supplement: S1 Data — (ZIP) [file ppat.1012546.s005.zip › Figure1-4, 5A-C, 6A-C, 6E, 6H-M and 7B-L. zip/Fig2/C/3/WCL-Pro-IL-1a┬.tif]

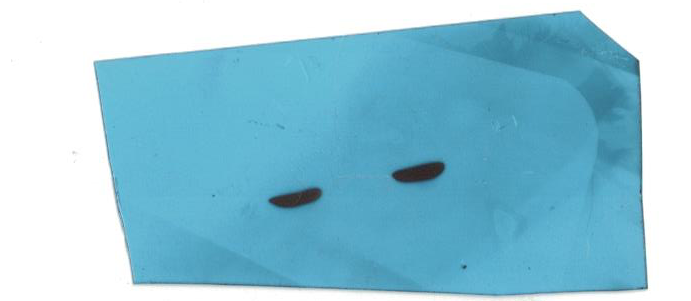

Supplement: S1 Data — (ZIP) [file ppat.1012546.s005.zip › Figure1-4, 5A-C, 6A-C, 6E, 6H-M and 7B-L. zip/Fig2/C/3/WCL-UL4.tif]

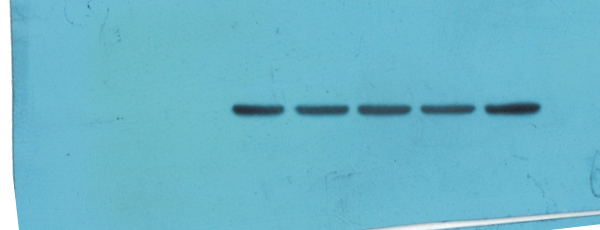

Supplement: S1 Data — (ZIP) [file ppat.1012546.s005.zip › Figure1-4, 5A-C, 6A-C, 6E, 6H-M and 7B-L. zip/Fig2/F/1/Actin.tif]

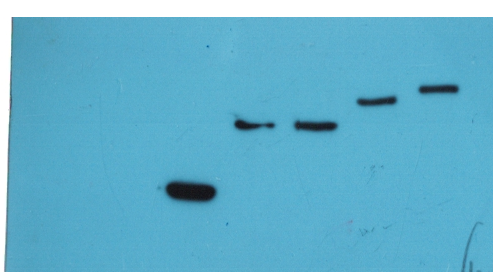

Supplement: S1 Data — (ZIP) [file ppat.1012546.s005.zip › Figure1-4, 5A-C, 6A-C, 6E, 6H-M and 7B-L. zip/Fig2/F/1/GFP.tif]

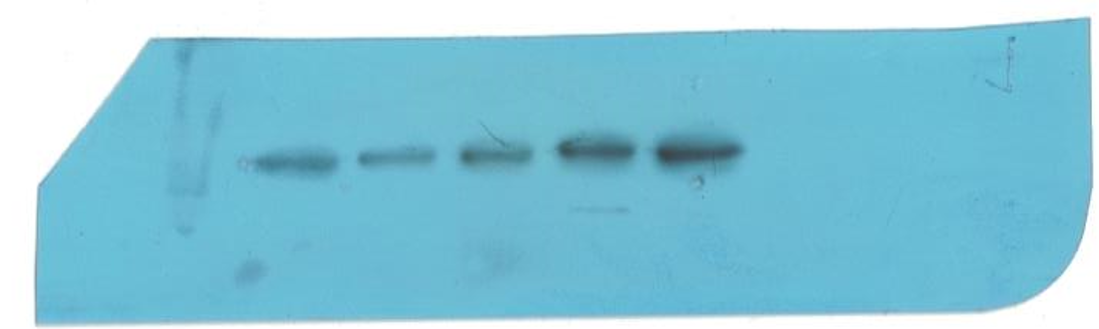

Supplement: S1 Data — (ZIP) [file ppat.1012546.s005.zip › Figure1-4, 5A-C, 6A-C, 6E, 6H-M and 7B-L. zip/Fig2/F/2/Actin.tiff]

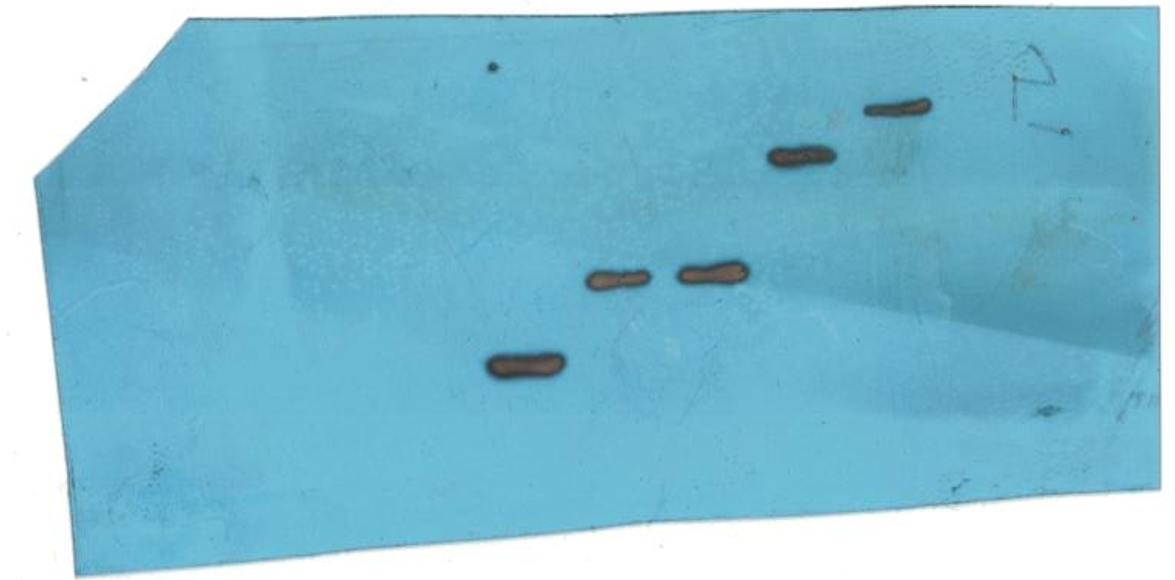

Supplement: S1 Data — (ZIP) [file ppat.1012546.s005.zip › Figure1-4, 5A-C, 6A-C, 6E, 6H-M and 7B-L. zip/Fig2/F/2/GFP .tiff]

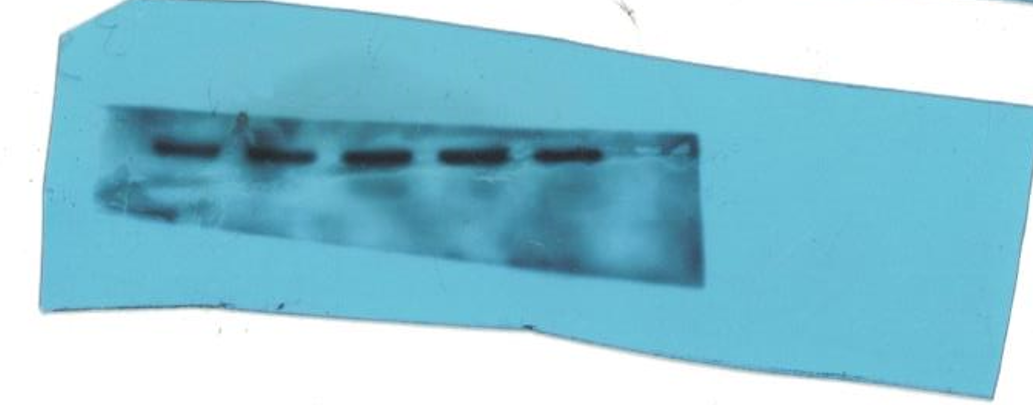

Supplement: S1 Data — (ZIP) [file ppat.1012546.s005.zip › Figure1-4, 5A-C, 6A-C, 6E, 6H-M and 7B-L. zip/Fig2/F/3/Actin.tiff]

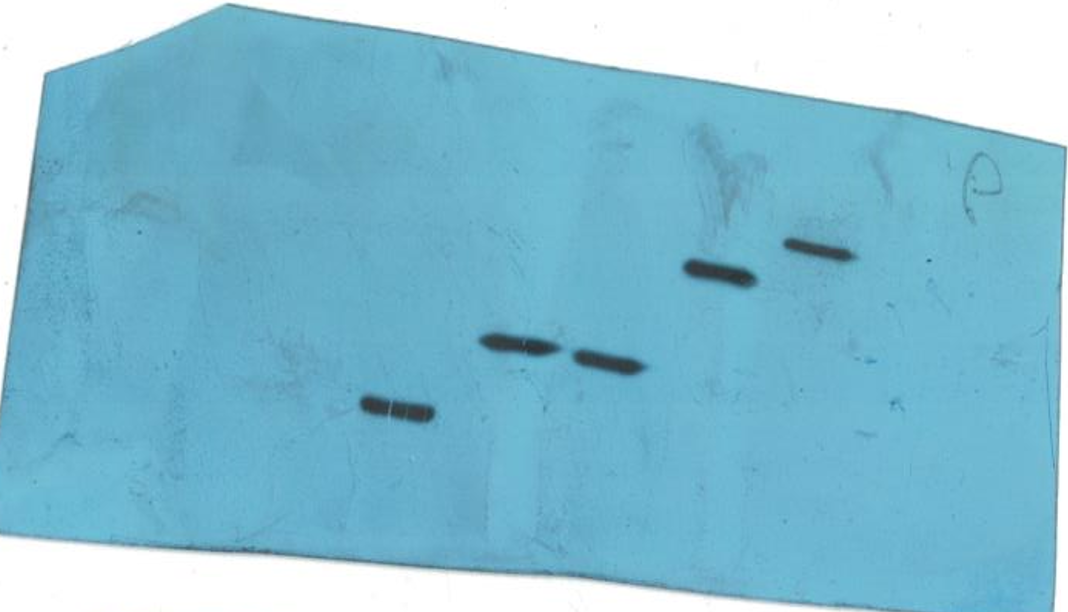

Supplement: S1 Data — (ZIP) [file ppat.1012546.s005.zip › Figure1-4, 5A-C, 6A-C, 6E, 6H-M and 7B-L. zip/Fig2/F/3/GFP .tiff]

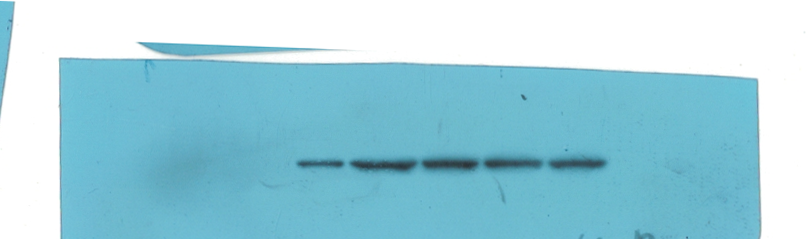

Supplement: S1 Data — (ZIP) [file ppat.1012546.s005.zip › Figure1-4, 5A-C, 6A-C, 6E, 6H-M and 7B-L. zip/Fig2/I/1/Sup-CASP1 P10.tif]

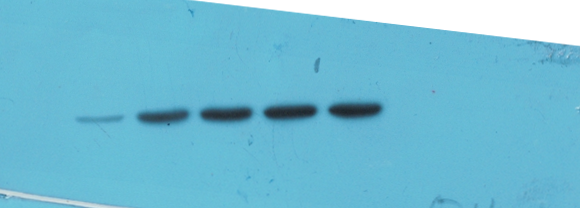

Supplement: S1 Data — (ZIP) [file ppat.1012546.s005.zip › Figure1-4, 5A-C, 6A-C, 6E, 6H-M and 7B-L. zip/Fig2/I/1/Sup-IL-1a┬ P17.tif]

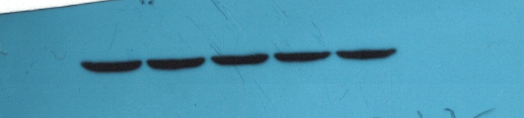

Supplement: S1 Data — (ZIP) [file ppat.1012546.s005.zip › Figure1-4, 5A-C, 6A-C, 6E, 6H-M and 7B-L. zip/Fig2/I/1/wcl-Actin.tif]

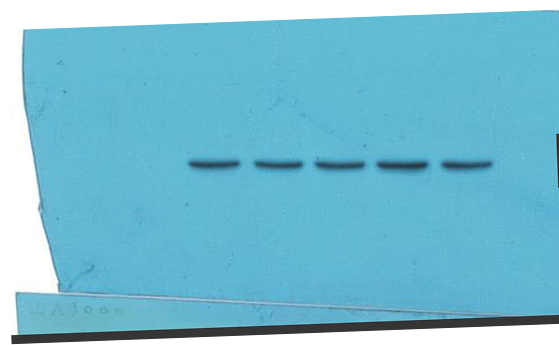

Supplement: S1 Data — (ZIP) [file ppat.1012546.s005.zip › Figure1-4, 5A-C, 6A-C, 6E, 6H-M and 7B-L. zip/Fig2/I/1/WCL-Pro-CASP1 .tif]

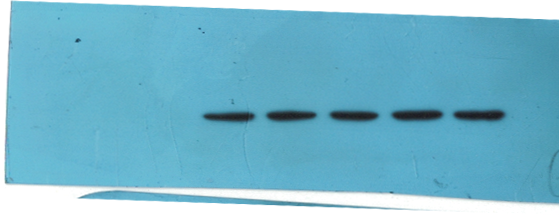

Supplement: S1 Data — (ZIP) [file ppat.1012546.s005.zip › Figure1-4, 5A-C, 6A-C, 6E, 6H-M and 7B-L. zip/Fig2/I/1/WCL-Pro-IL-1a┬ .tif]

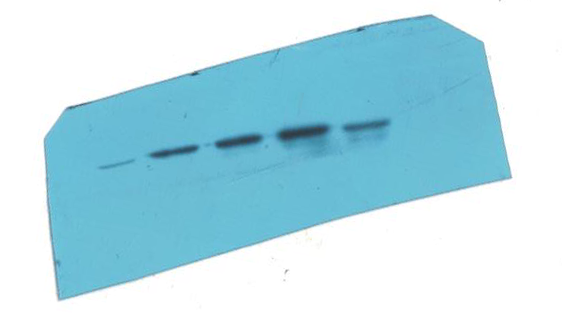

Supplement: S1 Data — (ZIP) [file ppat.1012546.s005.zip › Figure1-4, 5A-C, 6A-C, 6E, 6H-M and 7B-L. zip/Fig2/I/2/Sup-CASP1 p10.tiff]

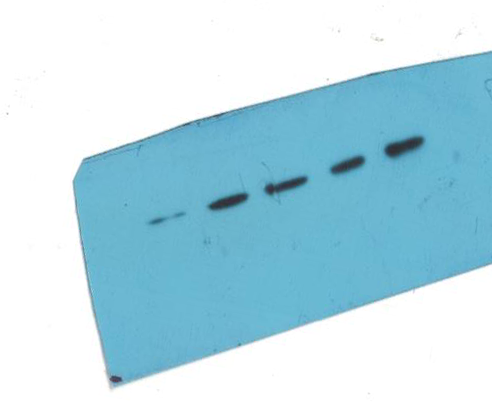

Supplement: S1 Data — (ZIP) [file ppat.1012546.s005.zip › Figure1-4, 5A-C, 6A-C, 6E, 6H-M and 7B-L. zip/Fig2/I/2/Sup-IL-1a┬ p17.tiff]

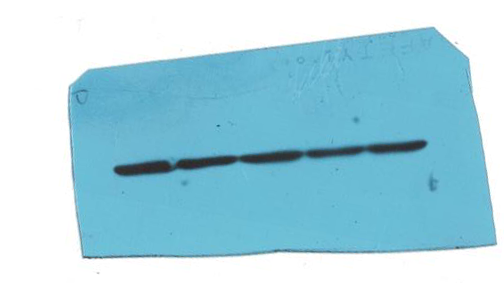

Supplement: S1 Data — (ZIP) [file ppat.1012546.s005.zip › Figure1-4, 5A-C, 6A-C, 6E, 6H-M and 7B-L. zip/Fig2/I/2/WCL-Actin.tiff]

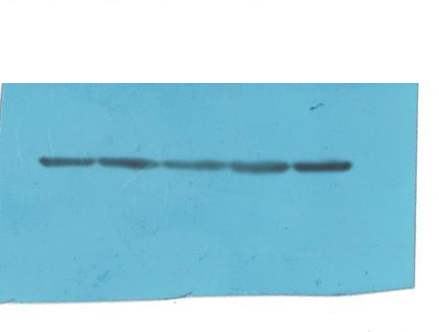

Supplement: S1 Data — (ZIP) [file ppat.1012546.s005.zip › Figure1-4, 5A-C, 6A-C, 6E, 6H-M and 7B-L. zip/Fig2/I/2/WCL-Pro-CASP1 .tiff]

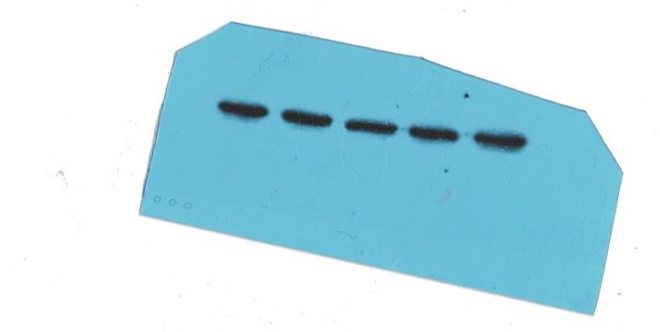

Supplement: S1 Data — (ZIP) [file ppat.1012546.s005.zip › Figure1-4, 5A-C, 6A-C, 6E, 6H-M and 7B-L. zip/Fig2/I/2/WCL-Pro-IL-1a┬.tiff]

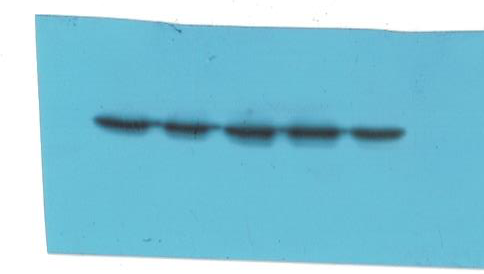

Supplement: S1 Data — (ZIP) [file ppat.1012546.s005.zip › Figure1-4, 5A-C, 6A-C, 6E, 6H-M and 7B-L. zip/Fig2/I/3/WCL-Actin.tif]

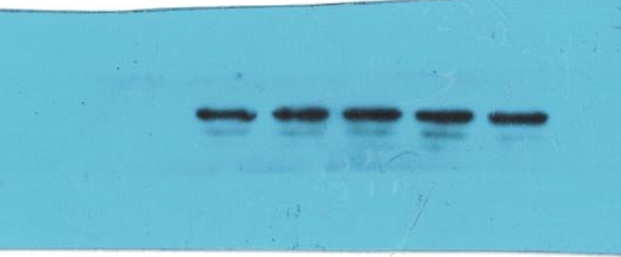

Supplement: S1 Data — (ZIP) [file ppat.1012546.s005.zip › Figure1-4, 5A-C, 6A-C, 6E, 6H-M and 7B-L. zip/Fig2/I/3/WCL-Pro-CASP1.tiff]

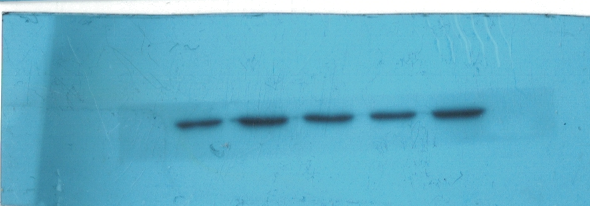

Supplement: S1 Data — (ZIP) [file ppat.1012546.s005.zip › Figure1-4, 5A-C, 6A-C, 6E, 6H-M and 7B-L. zip/Fig2/J/1/Sup-CASP1 p10.tif]

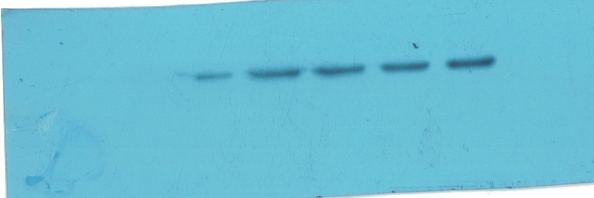

Supplement: S1 Data — (ZIP) [file ppat.1012546.s005.zip › Figure1-4, 5A-C, 6A-C, 6E, 6H-M and 7B-L. zip/Fig2/J/1/Sup-IL-1a┬ p17.tif]

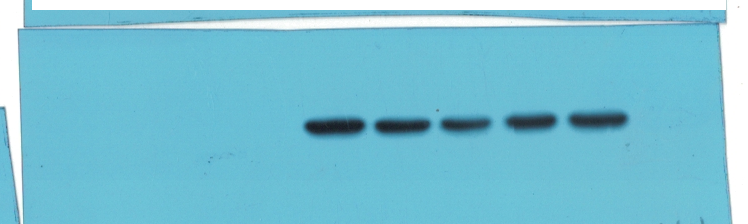

Supplement: S1 Data — (ZIP) [file ppat.1012546.s005.zip › Figure1-4, 5A-C, 6A-C, 6E, 6H-M and 7B-L. zip/Fig2/J/1/wcl-Actin.tif]

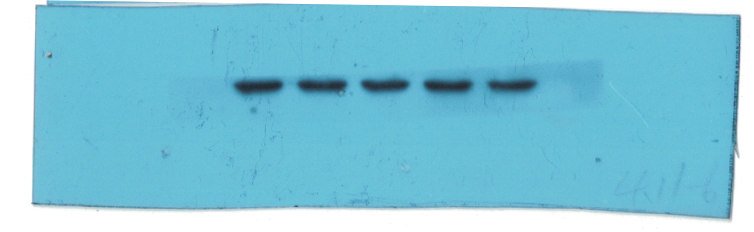

Supplement: S1 Data — (ZIP) [file ppat.1012546.s005.zip › Figure1-4, 5A-C, 6A-C, 6E, 6H-M and 7B-L. zip/Fig2/J/1/wcl-pRO-CASP1.tif]

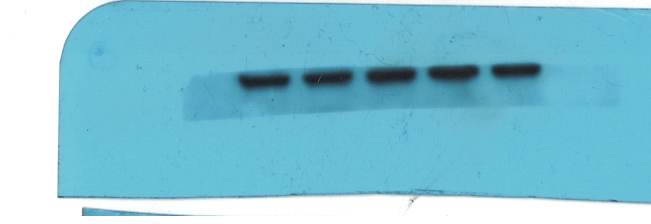

Supplement: S1 Data — (ZIP) [file ppat.1012546.s005.zip › Figure1-4, 5A-C, 6A-C, 6E, 6H-M and 7B-L. zip/Fig2/J/1/wcl-pro-IL-1a┬.tif]

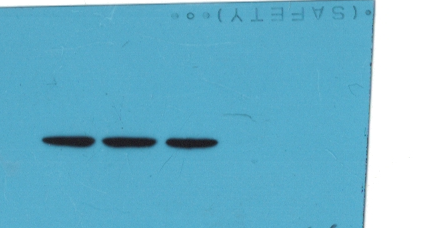

Supplement: S1 Data — (ZIP) [file ppat.1012546.s005.zip › Figure1-4, 5A-C, 6A-C, 6E, 6H-M and 7B-L. zip/Fig3/B/1/Actin.tif]

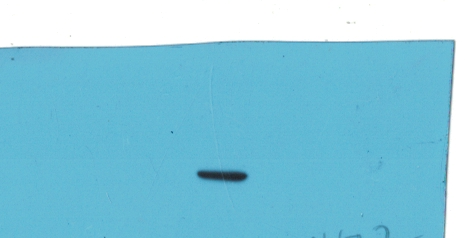

Supplement: S1 Data — (ZIP) [file ppat.1012546.s005.zip › Figure1-4, 5A-C, 6A-C, 6E, 6H-M and 7B-L. zip/Fig3/B/1/GFP.tif]

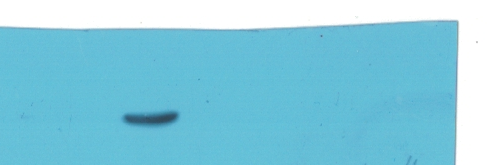

Supplement: S1 Data — (ZIP) [file ppat.1012546.s005.zip › Figure1-4, 5A-C, 6A-C, 6E, 6H-M and 7B-L. zip/Fig3/B/1/UL4.tif]

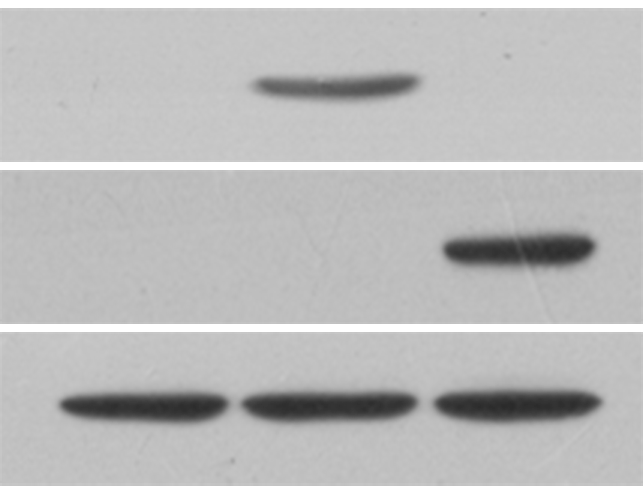

Supplement: S1 Data — (ZIP) [file ppat.1012546.s005.zip › Figure1-4, 5A-C, 6A-C, 6E, 6H-M and 7B-L. zip/Fig3/B/1/UL4╟├│2╢╛╓Ω╕╨╚╛╧╕░√WB═╝-1.tif]

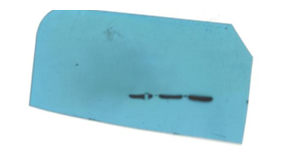

Supplement: S1 Data — (ZIP) [file ppat.1012546.s005.zip › Figure1-4, 5A-C, 6A-C, 6E, 6H-M and 7B-L. zip/Fig3/B/2/Actin.tif]

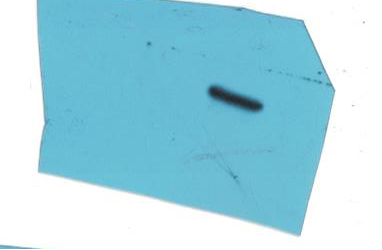

Supplement: S1 Data — (ZIP) [file ppat.1012546.s005.zip › Figure1-4, 5A-C, 6A-C, 6E, 6H-M and 7B-L. zip/Fig3/B/2/GFP.tif]

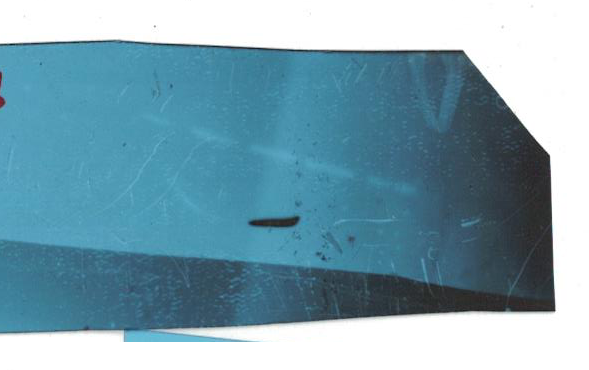

Supplement: S1 Data — (ZIP) [file ppat.1012546.s005.zip › Figure1-4, 5A-C, 6A-C, 6E, 6H-M and 7B-L. zip/Fig3/B/2/UL4.tif]

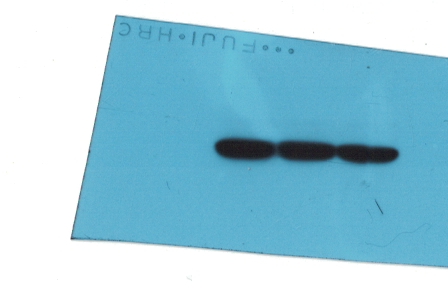

Supplement: S1 Data — (ZIP) [file ppat.1012546.s005.zip › Figure1-4, 5A-C, 6A-C, 6E, 6H-M and 7B-L. zip/Fig3/B/3/Actin.tif]

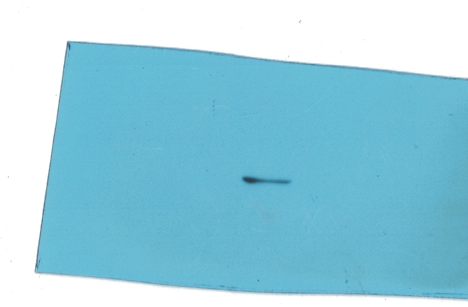

Supplement: S1 Data — (ZIP) [file ppat.1012546.s005.zip › Figure1-4, 5A-C, 6A-C, 6E, 6H-M and 7B-L. zip/Fig3/B/3/GFP.tif]

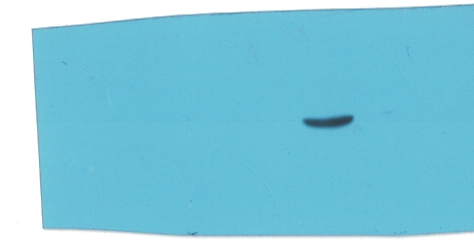

Supplement: S1 Data — (ZIP) [file ppat.1012546.s005.zip › Figure1-4, 5A-C, 6A-C, 6E, 6H-M and 7B-L. zip/Fig3/B/3/UL4.tif]

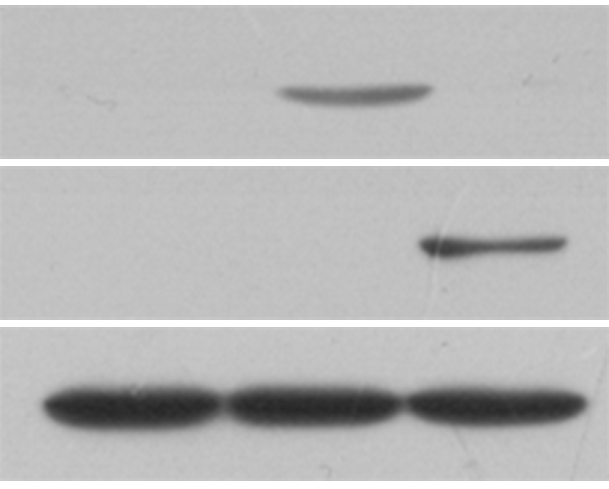

Supplement: S1 Data — (ZIP) [file ppat.1012546.s005.zip › Figure1-4, 5A-C, 6A-C, 6E, 6H-M and 7B-L. zip/Fig3/B/3/UL4╟├│2╢╛╓Ω╕╨╚╛╧╕░√WB═╝-2.tif]

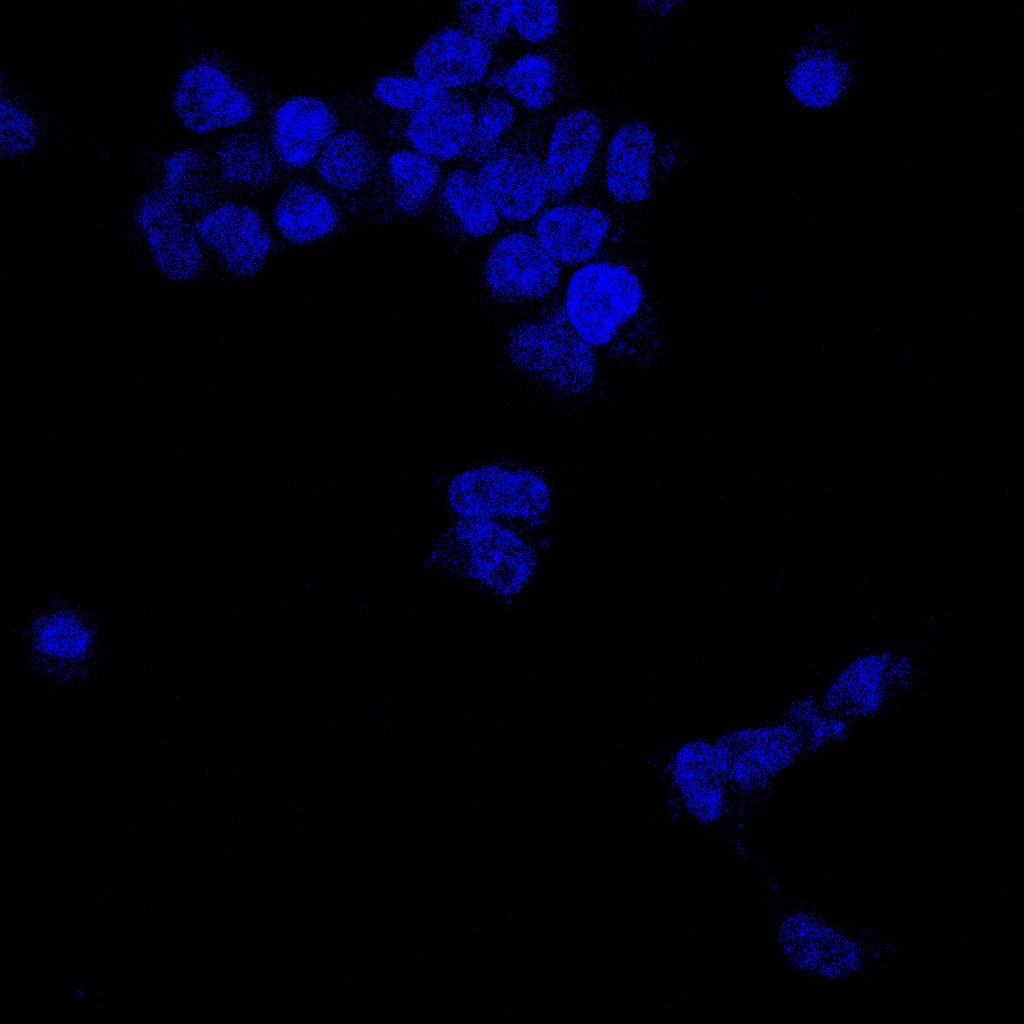

Supplement: S2 Data — (ZIP) [file ppat.1012546.s006.zip › Figure 5D/1/Flag-ASC/DAPI.tif]

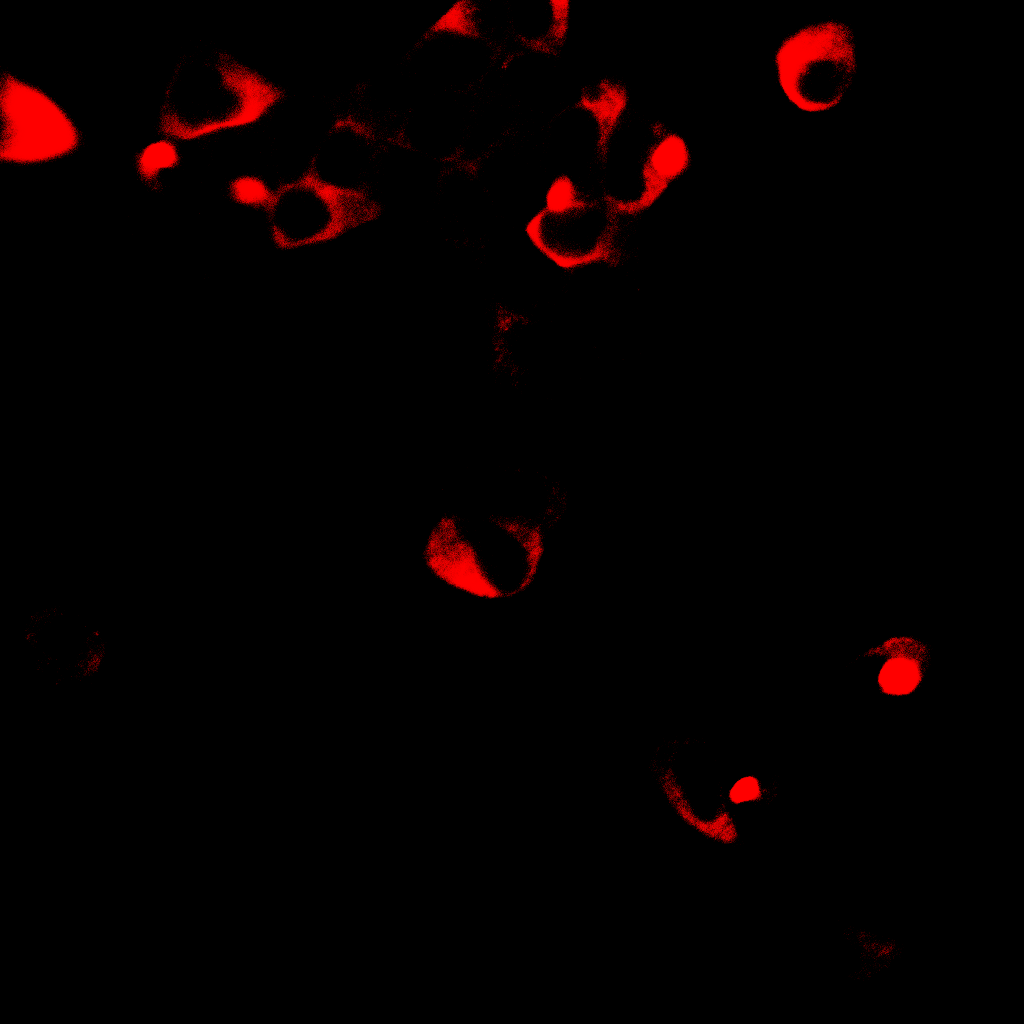

Supplement: S2 Data — (ZIP) [file ppat.1012546.s006.zip › Figure 5D/1/Flag-ASC/Flag-ASC .tif]

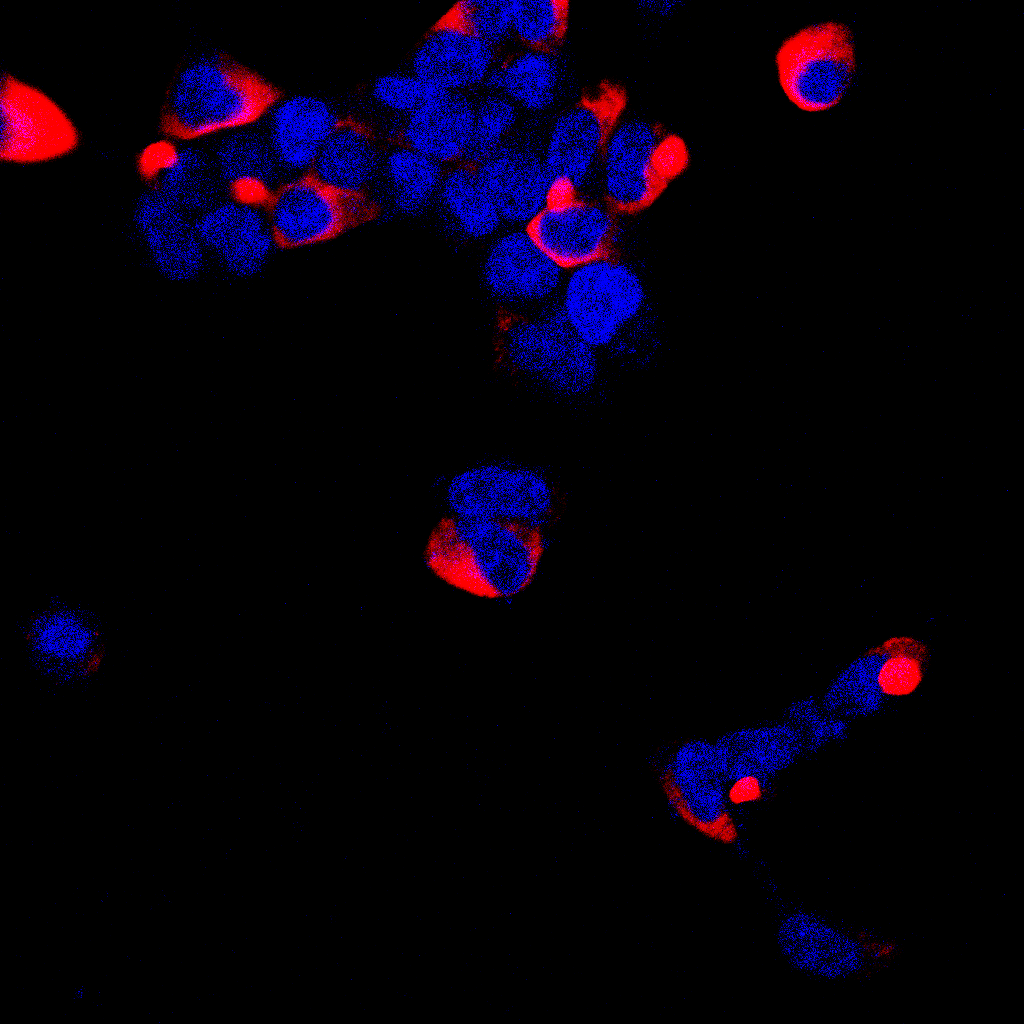

Supplement: S2 Data — (ZIP) [file ppat.1012546.s006.zip › Figure 5D/1/Flag-ASC/Merge.tif]

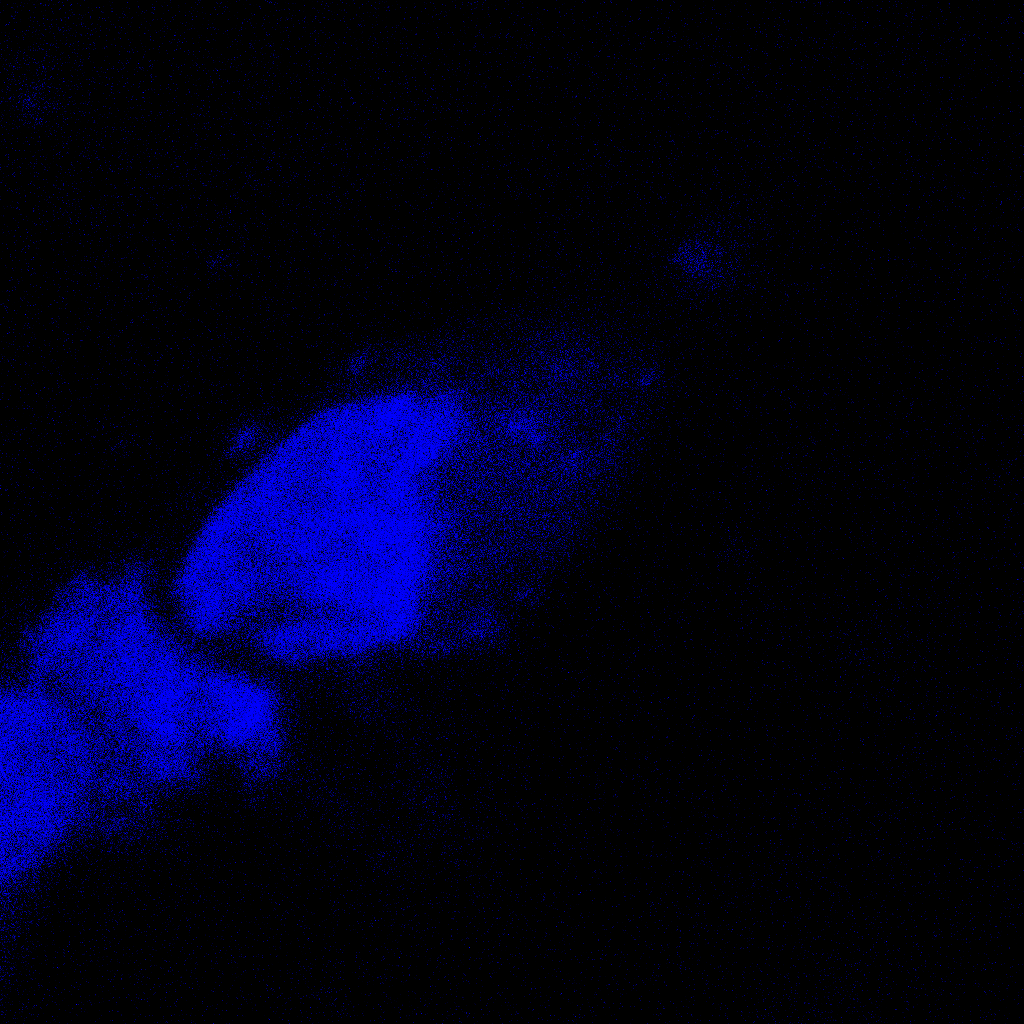

Supplement: S2 Data — (ZIP) [file ppat.1012546.s006.zip › Figure 5D/1/Flag-ASC+GFP-UL4/1/DAPI.tif]

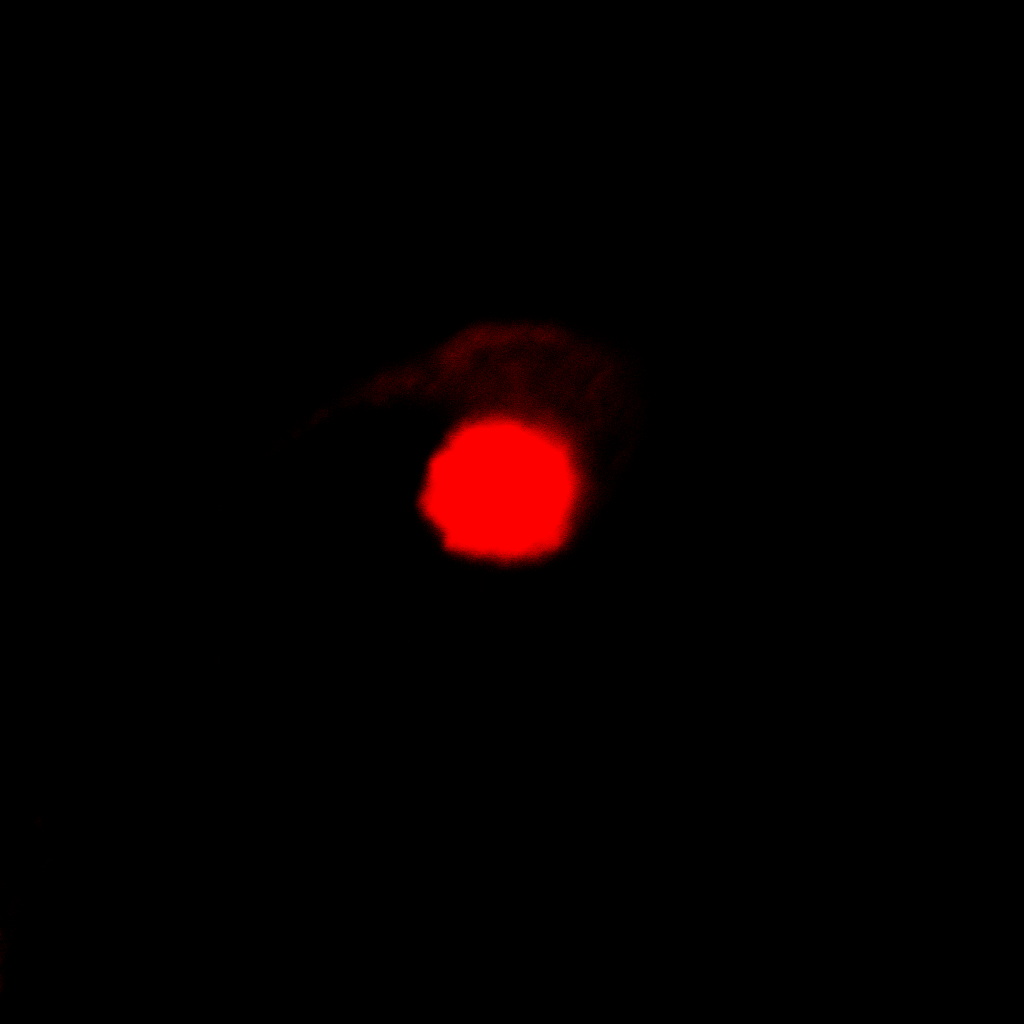

Supplement: S2 Data — (ZIP) [file ppat.1012546.s006.zip › Figure 5D/1/Flag-ASC+GFP-UL4/1/flag-asc.tif]

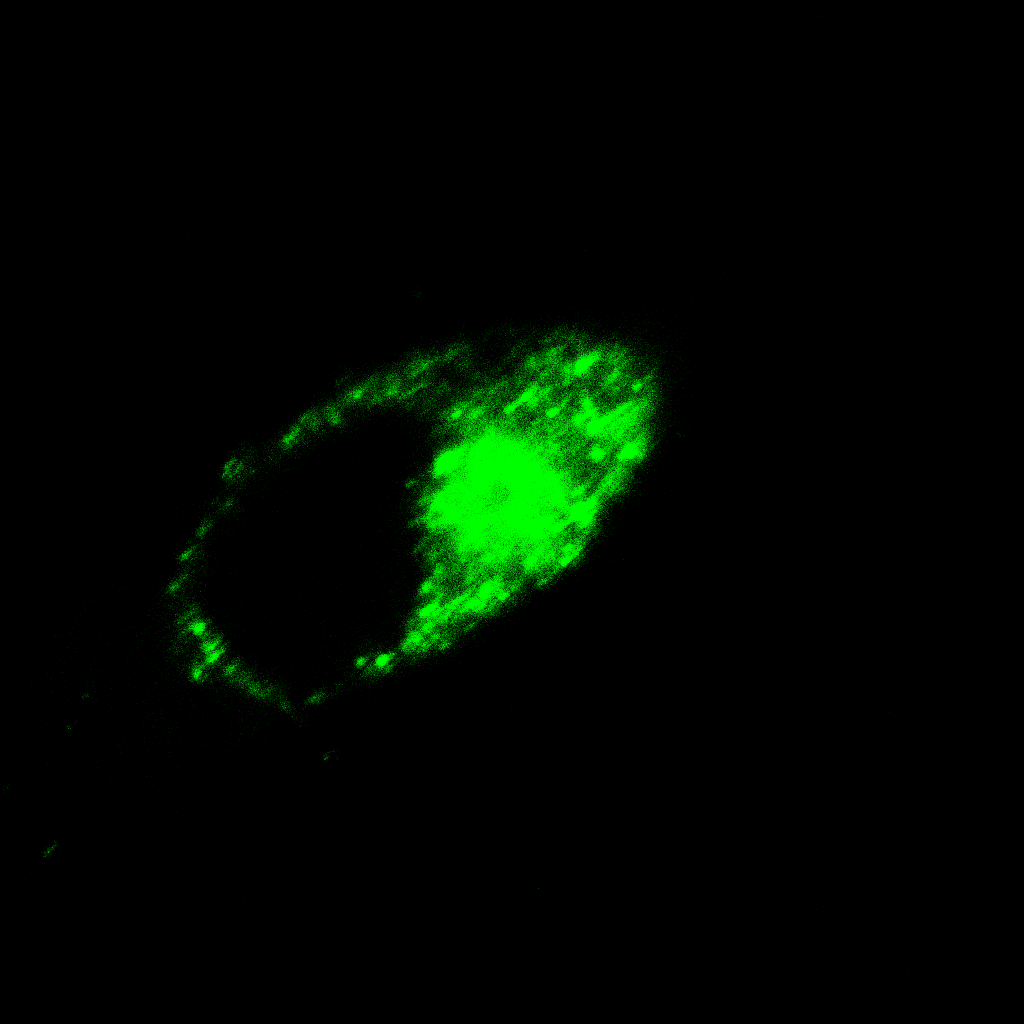

Supplement: S2 Data — (ZIP) [file ppat.1012546.s006.zip › Figure 5D/1/Flag-ASC+GFP-UL4/1/GFP-UL4.tif]

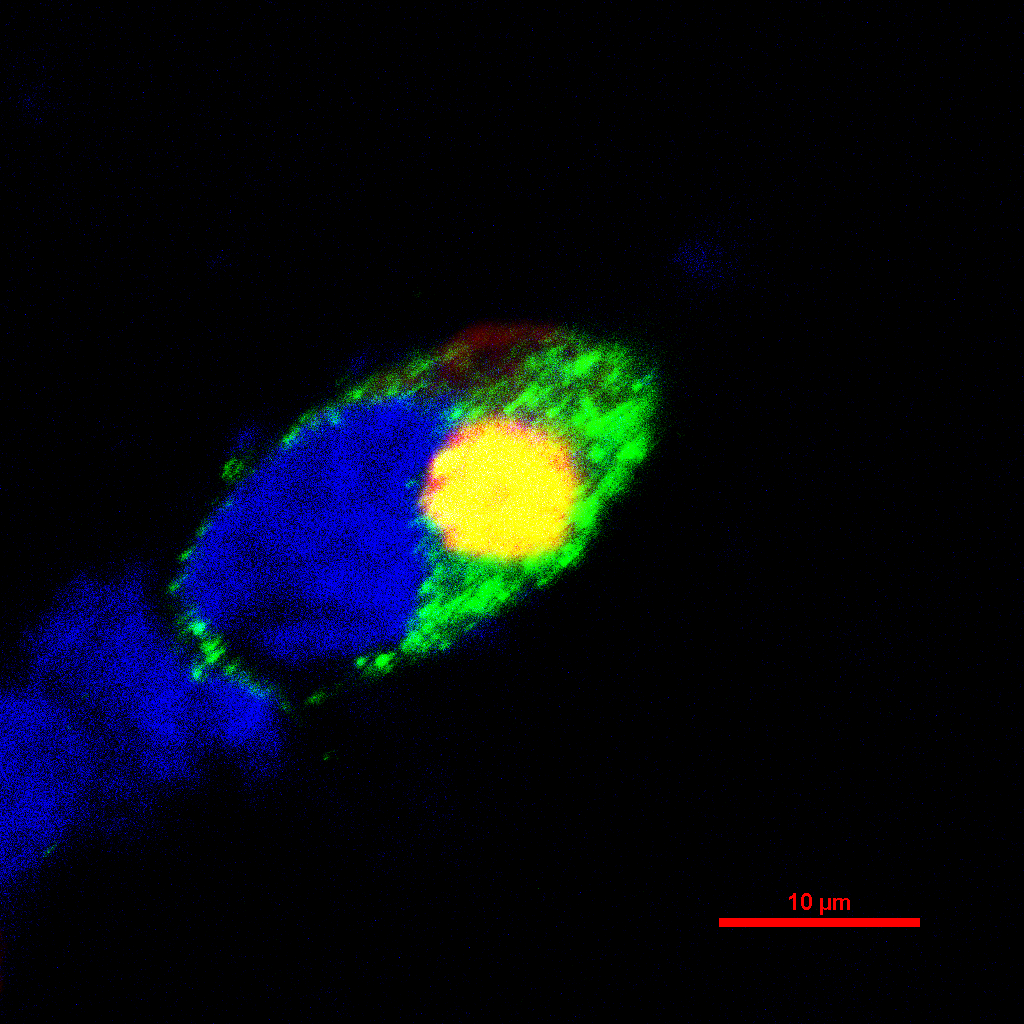

Supplement: S2 Data — (ZIP) [file ppat.1012546.s006.zip › Figure 5D/1/Flag-ASC+GFP-UL4/1/Merge.tif]

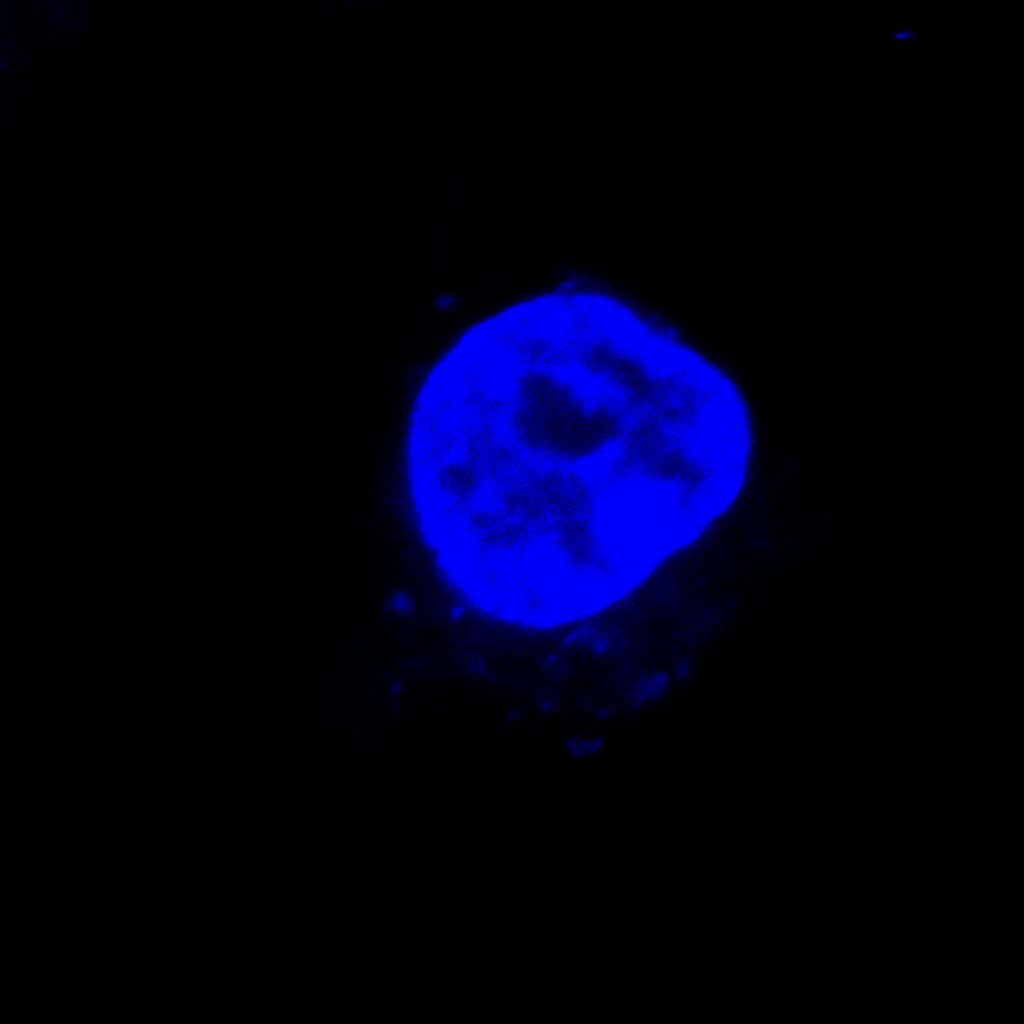

Supplement: S2 Data — (ZIP) [file ppat.1012546.s006.zip › Figure 5D/1/Flag-ASC+GFP-UL4/2/DAPI.tif]

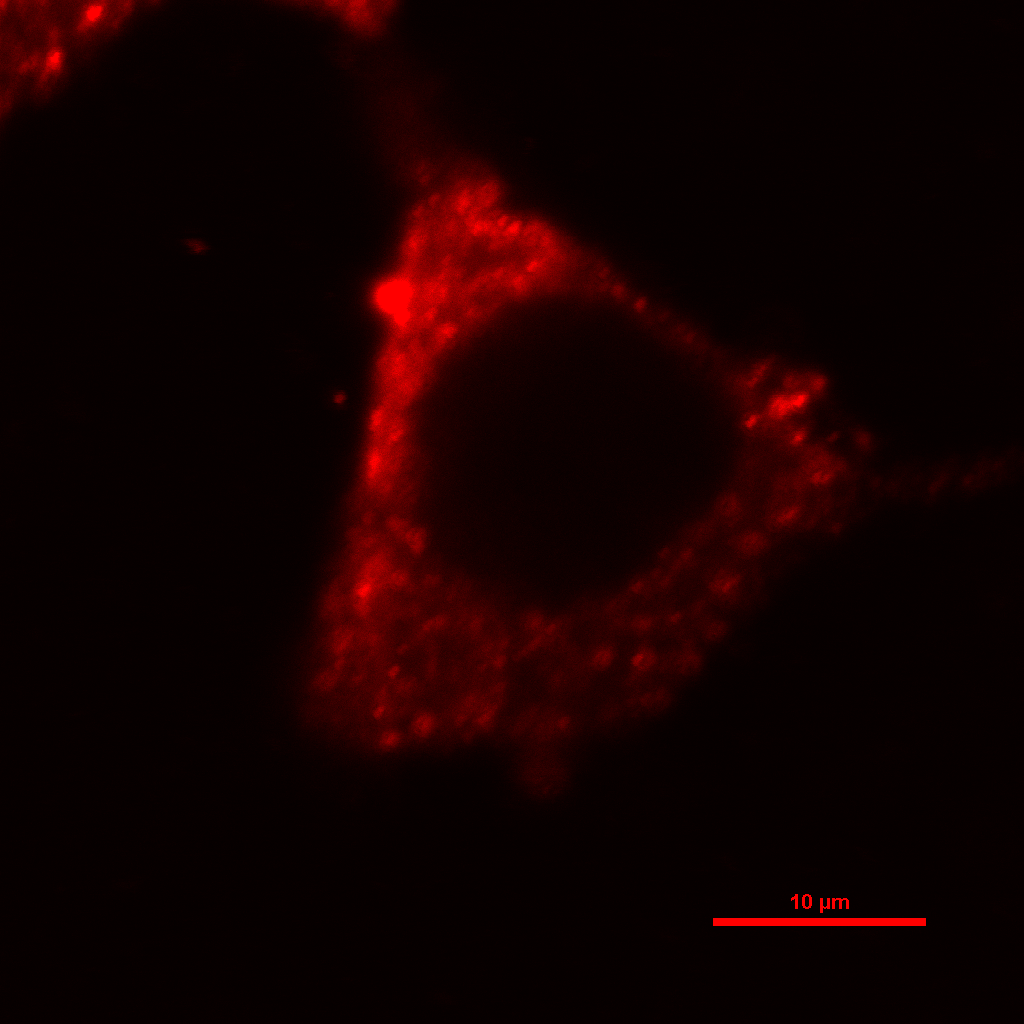

Supplement: S2 Data — (ZIP) [file ppat.1012546.s006.zip › Figure 5D/1/Flag-ASC+GFP-UL4/2/Flag-ASC.tif]

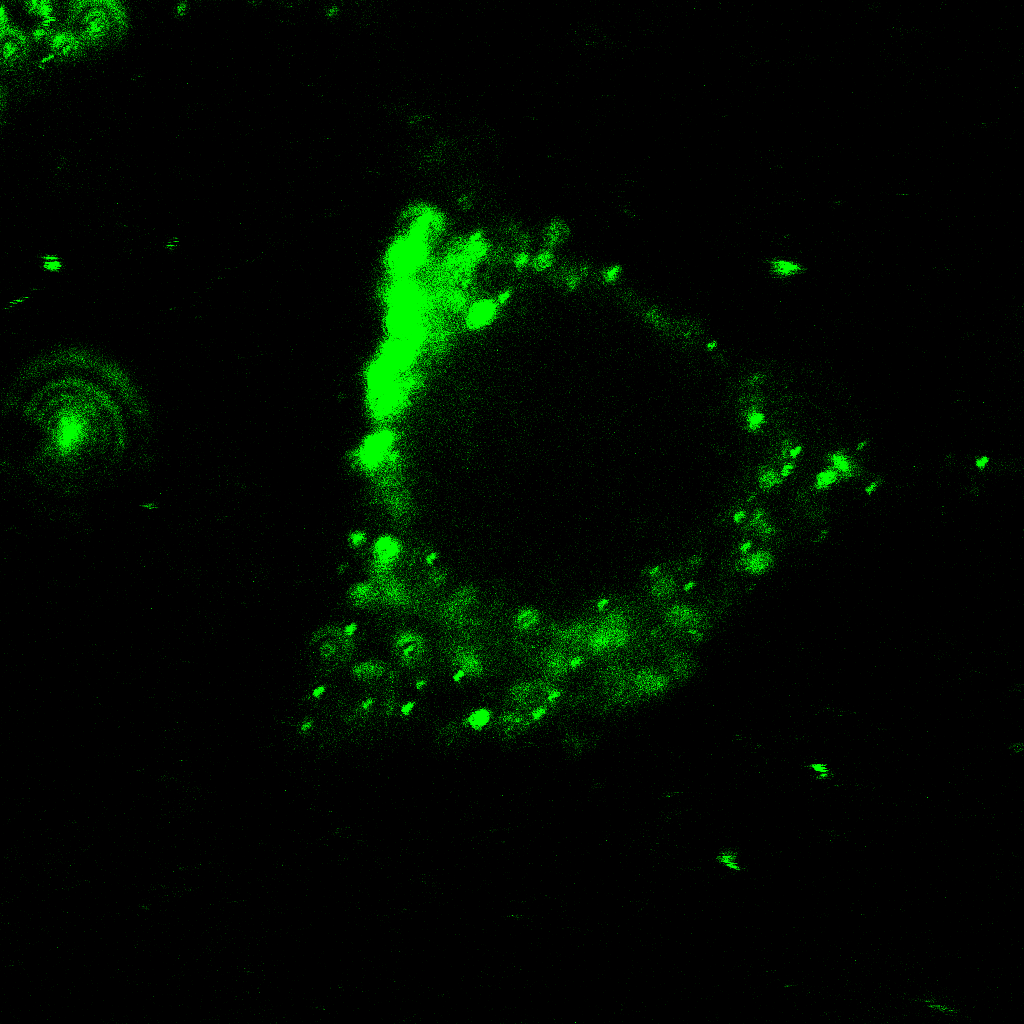

Supplement: S2 Data — (ZIP) [file ppat.1012546.s006.zip › Figure 5D/1/Flag-ASC+GFP-UL4/2/GFP-UL4.tif]

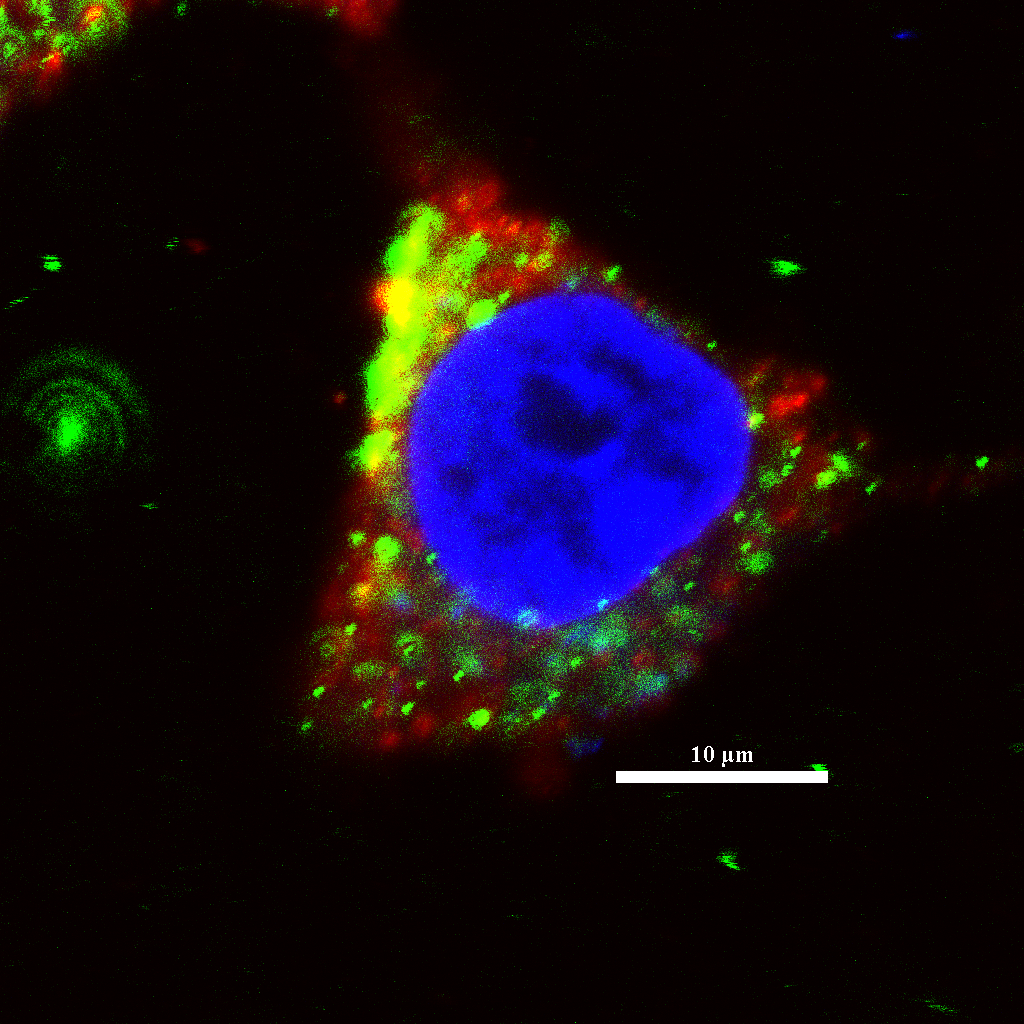

Supplement: S2 Data — (ZIP) [file ppat.1012546.s006.zip › Figure 5D/1/Flag-ASC+GFP-UL4/2/Merge.tif]

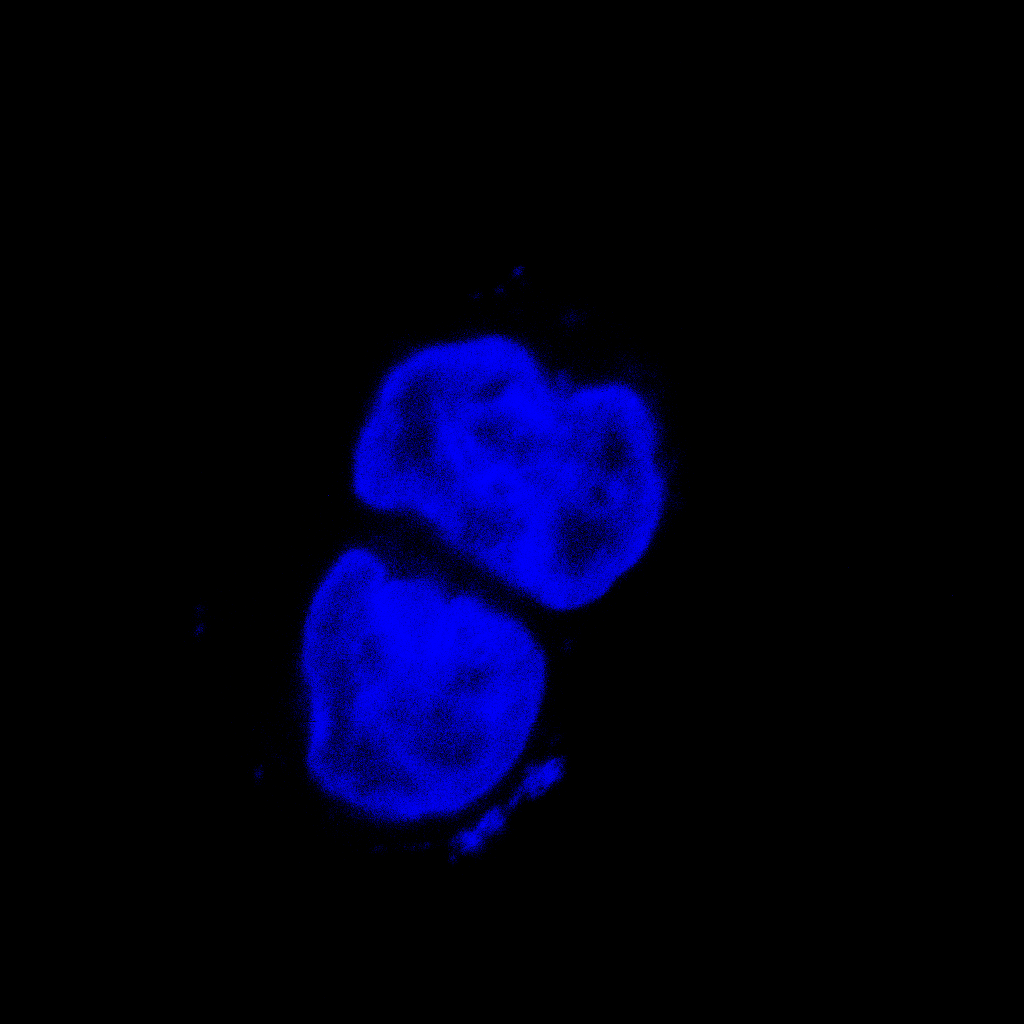

Supplement: S2 Data — (ZIP) [file ppat.1012546.s006.zip › Figure 5D/1/Flag-CASP1/DAPI.tif]

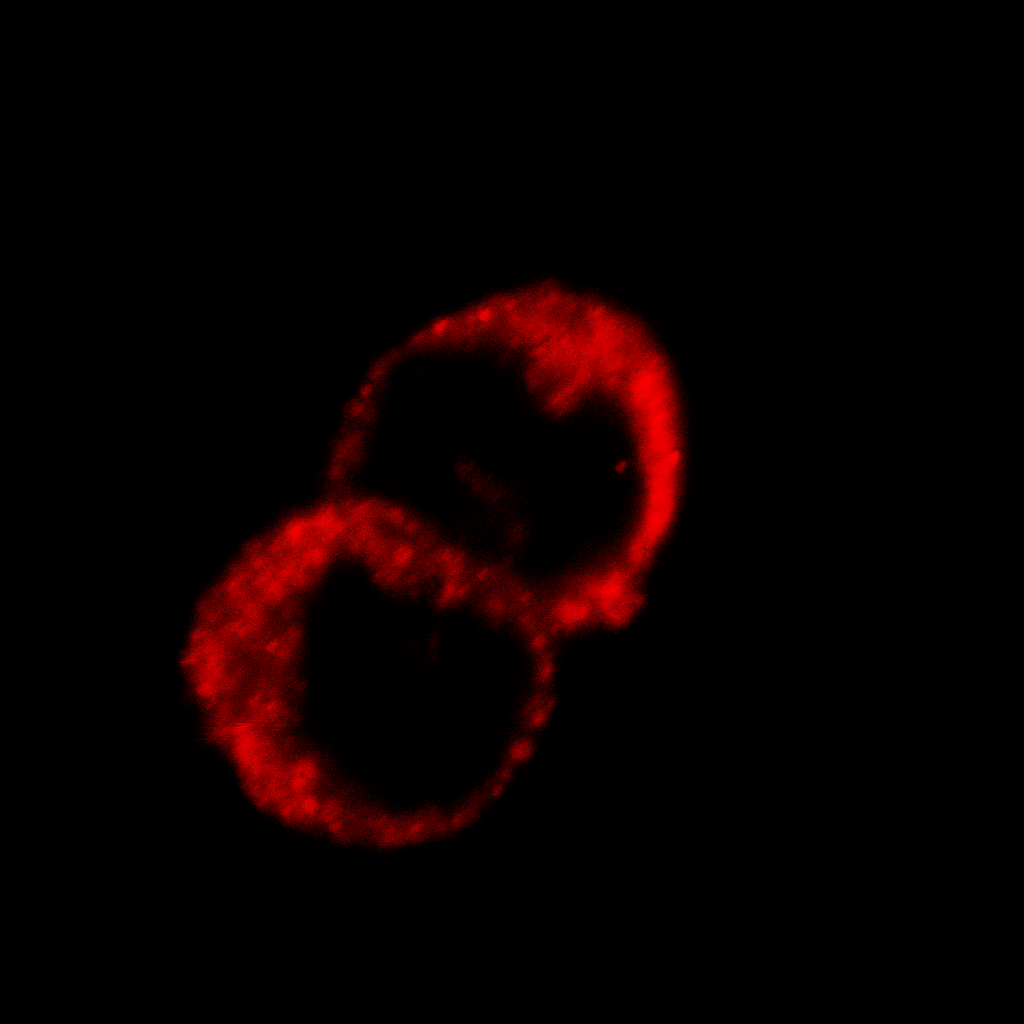

Supplement: S2 Data — (ZIP) [file ppat.1012546.s006.zip › Figure 5D/1/Flag-CASP1/Flag-CASP1.tif]

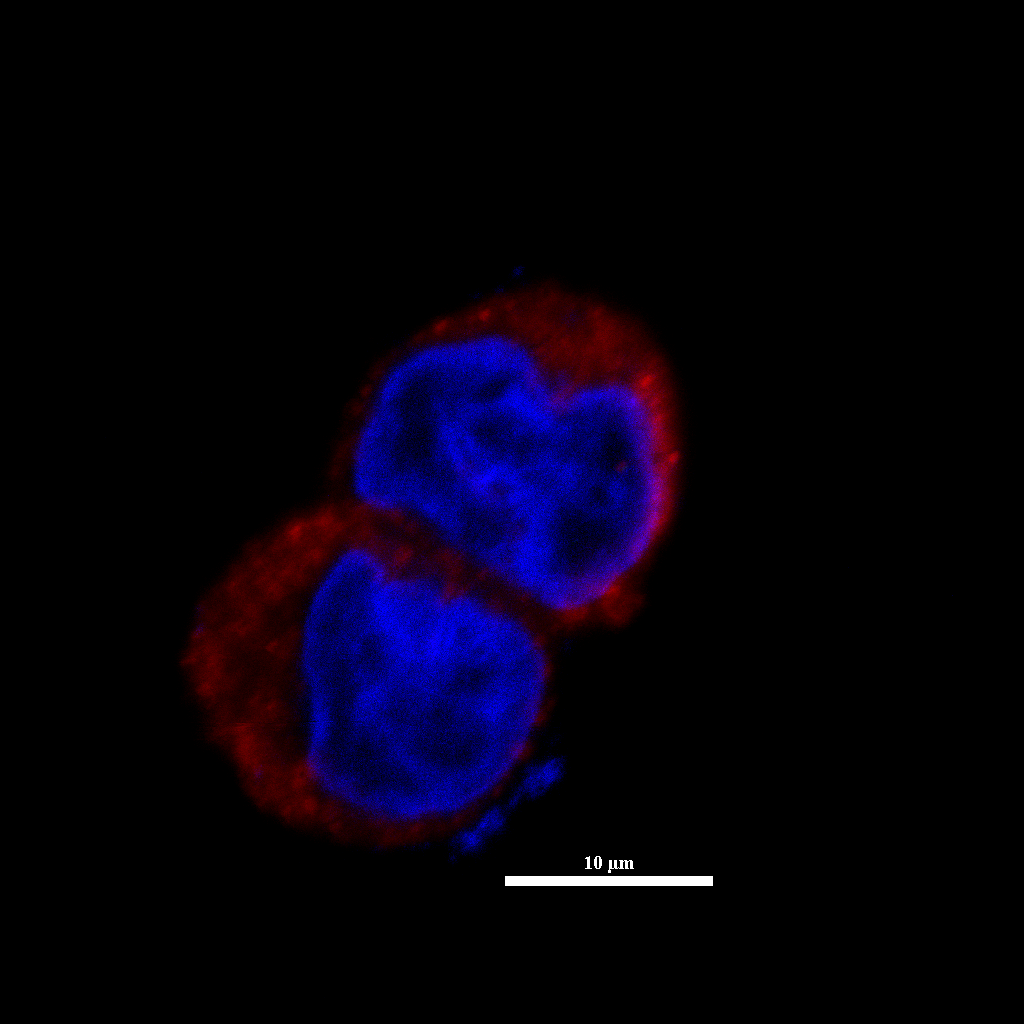

Supplement: S2 Data — (ZIP) [file ppat.1012546.s006.zip › Figure 5D/1/Flag-CASP1/Merge.tif]

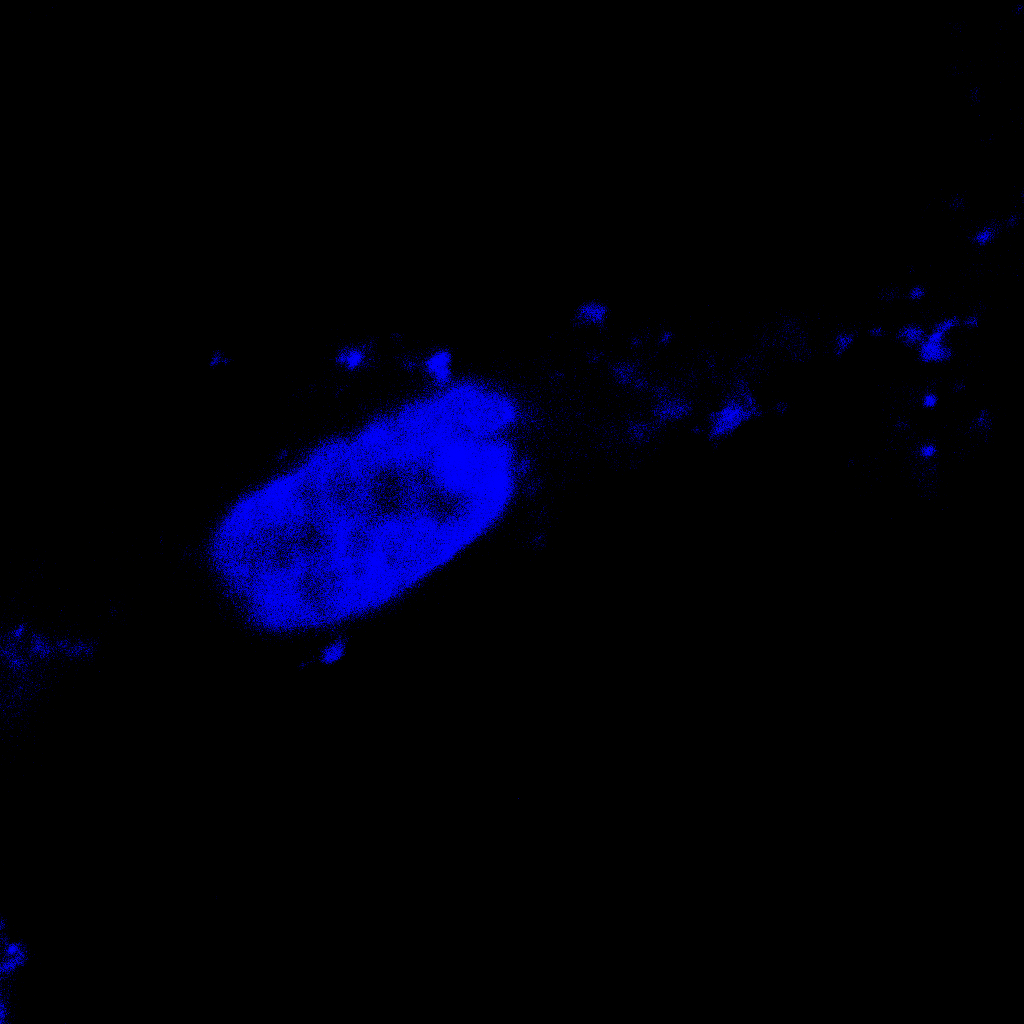

Supplement: S2 Data — (ZIP) [file ppat.1012546.s006.zip › Figure 5D/1/Flag-CASP1+GFP-UL4/DAPI.tif]

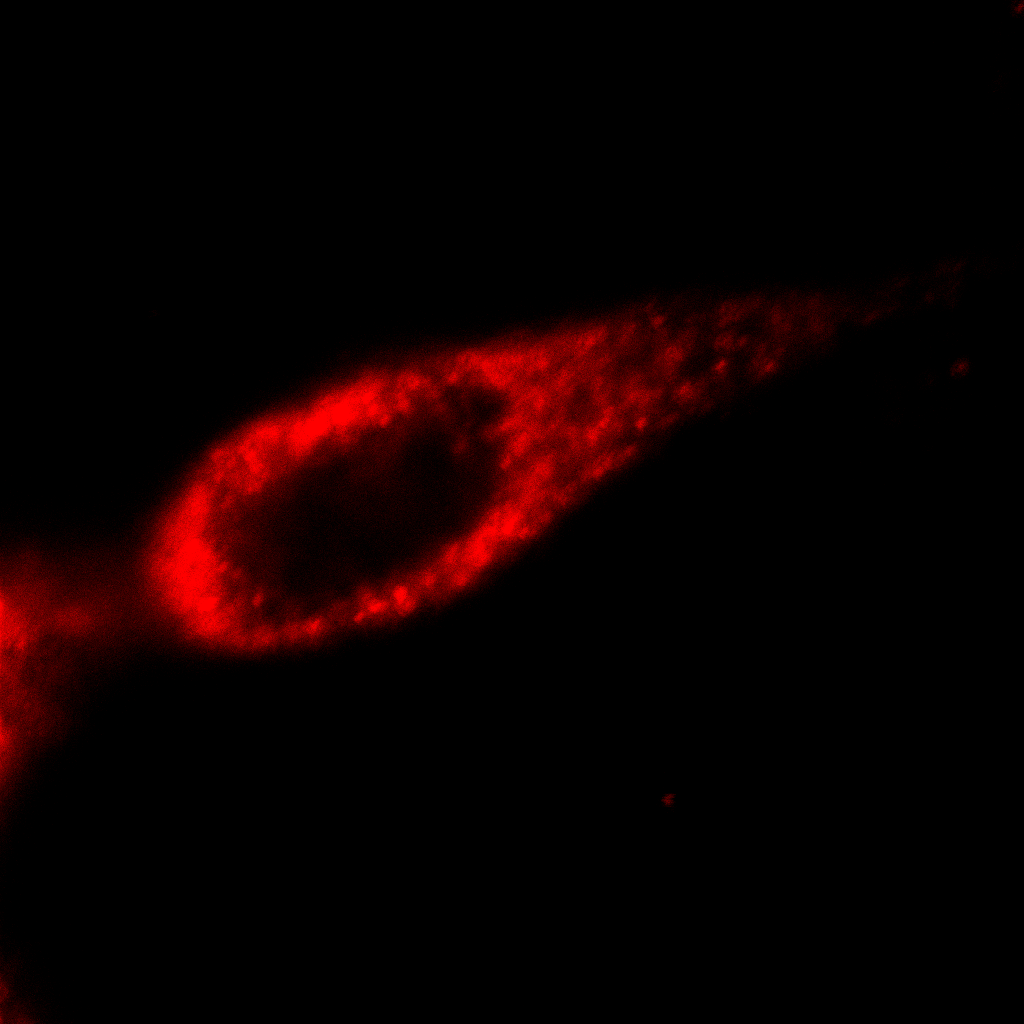

Supplement: S2 Data — (ZIP) [file ppat.1012546.s006.zip › Figure 5D/1/Flag-CASP1+GFP-UL4/Flag-ASC.tif]

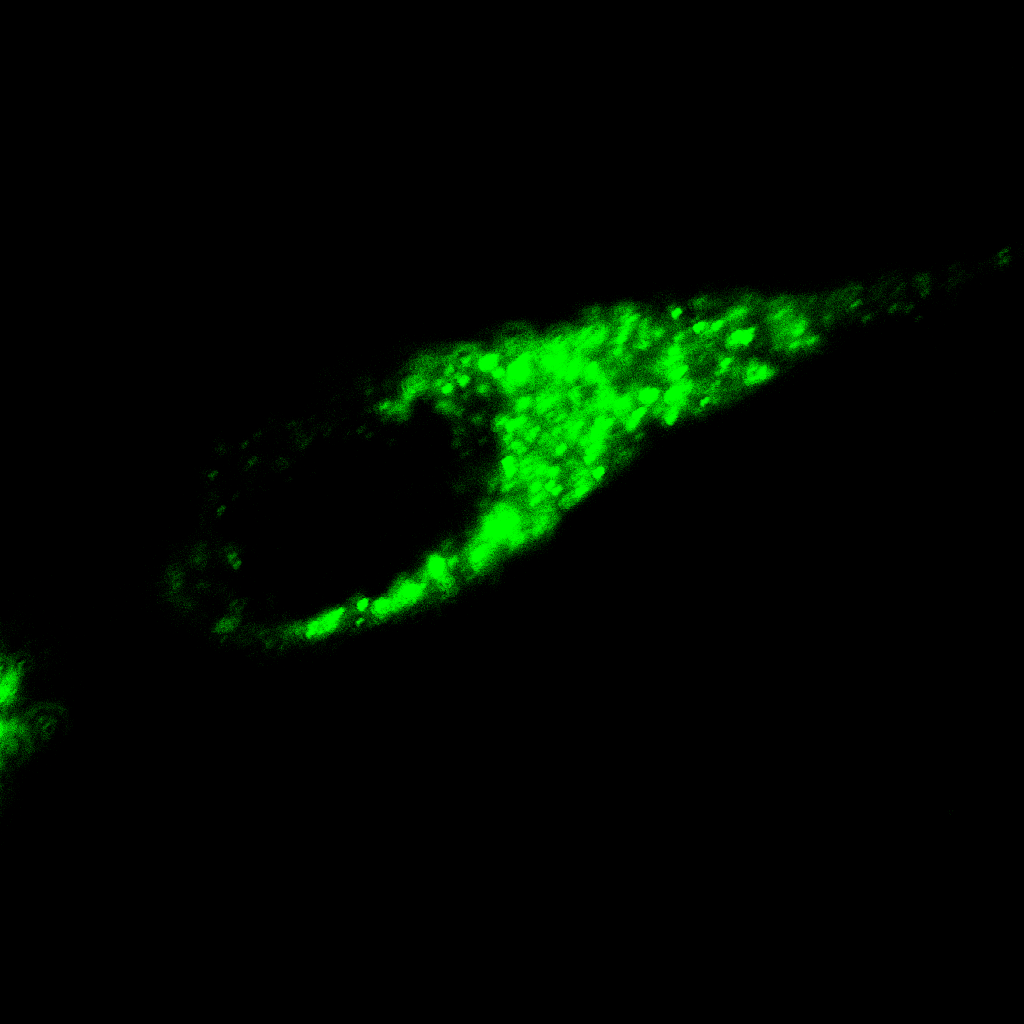

Supplement: S2 Data — (ZIP) [file ppat.1012546.s006.zip › Figure 5D/1/Flag-CASP1+GFP-UL4/GFP-UL4.tif]

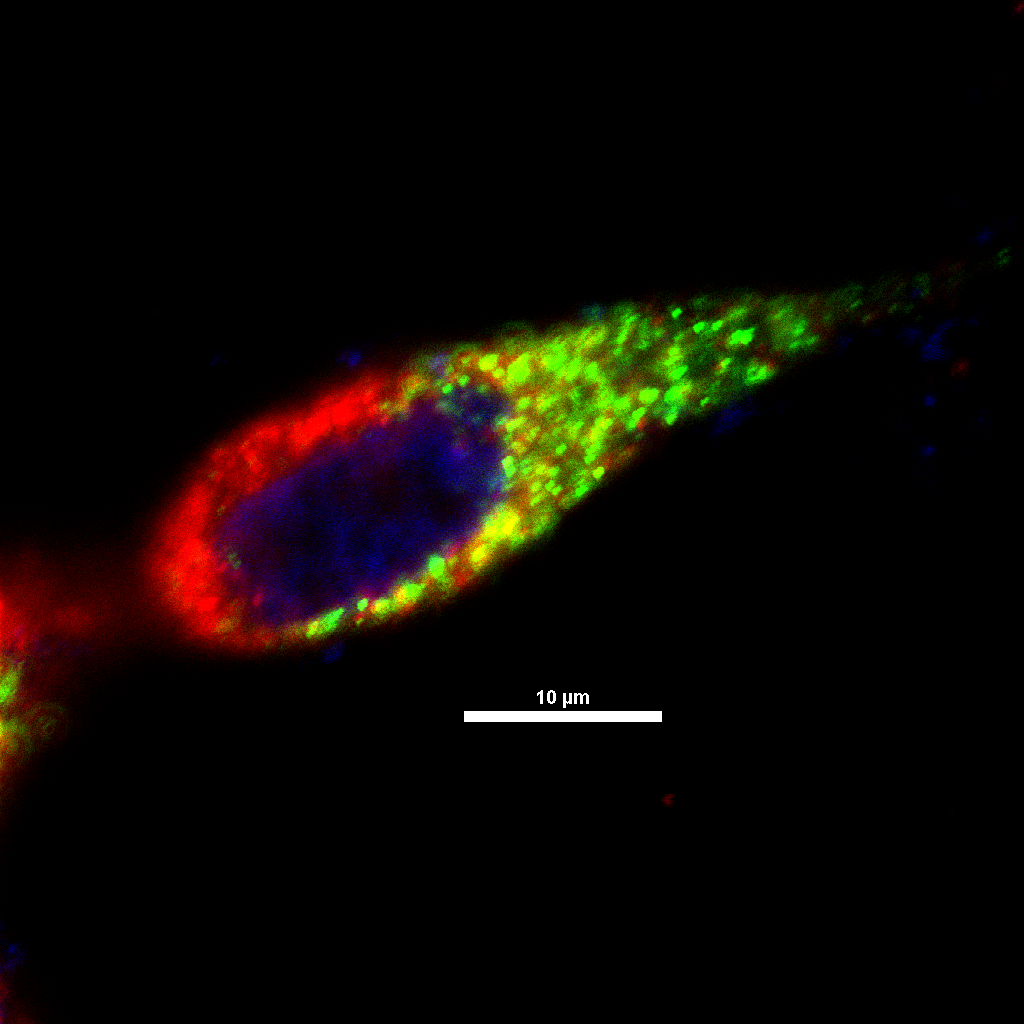

Supplement: S2 Data — (ZIP) [file ppat.1012546.s006.zip › Figure 5D/1/Flag-CASP1+GFP-UL4/Merge.tif]

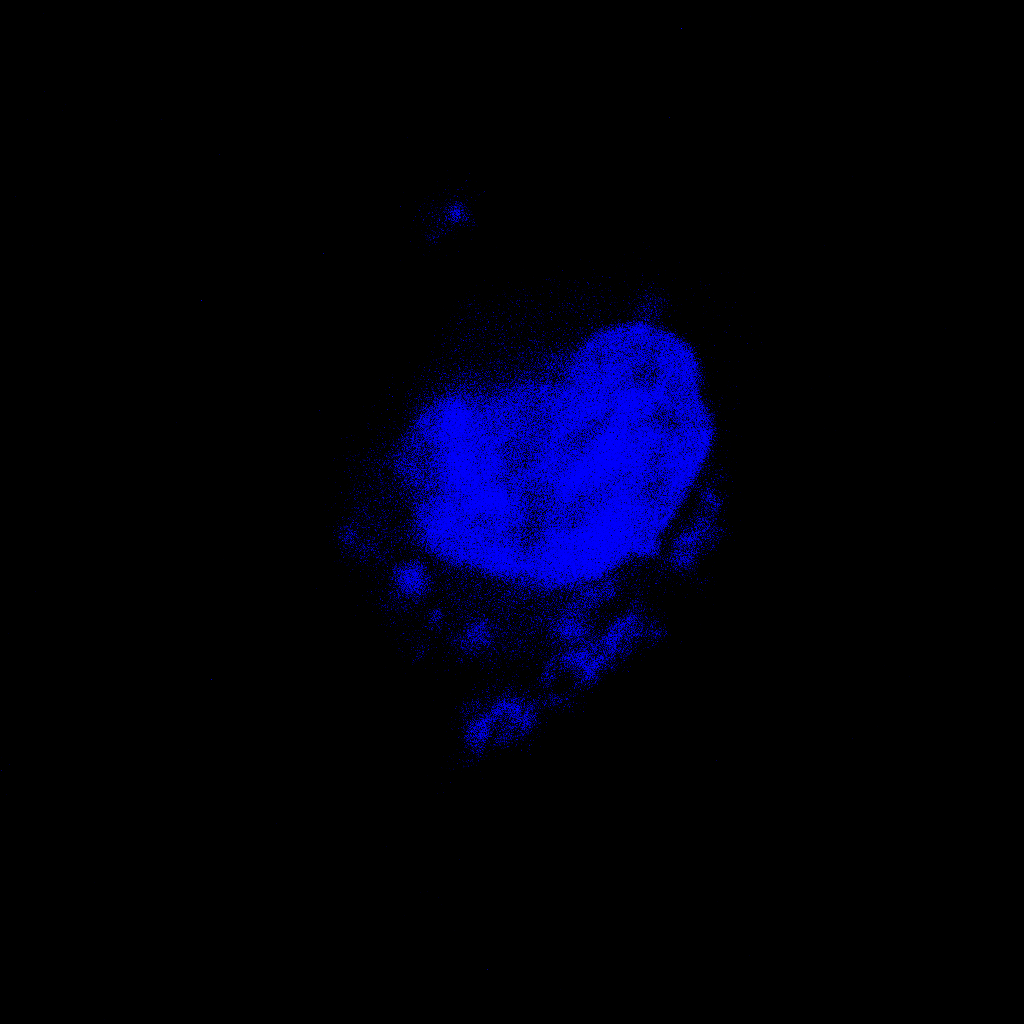

Supplement: S2 Data — (ZIP) [file ppat.1012546.s006.zip › Figure 5D/1/Flag-NLRP3/DAPI.tif]

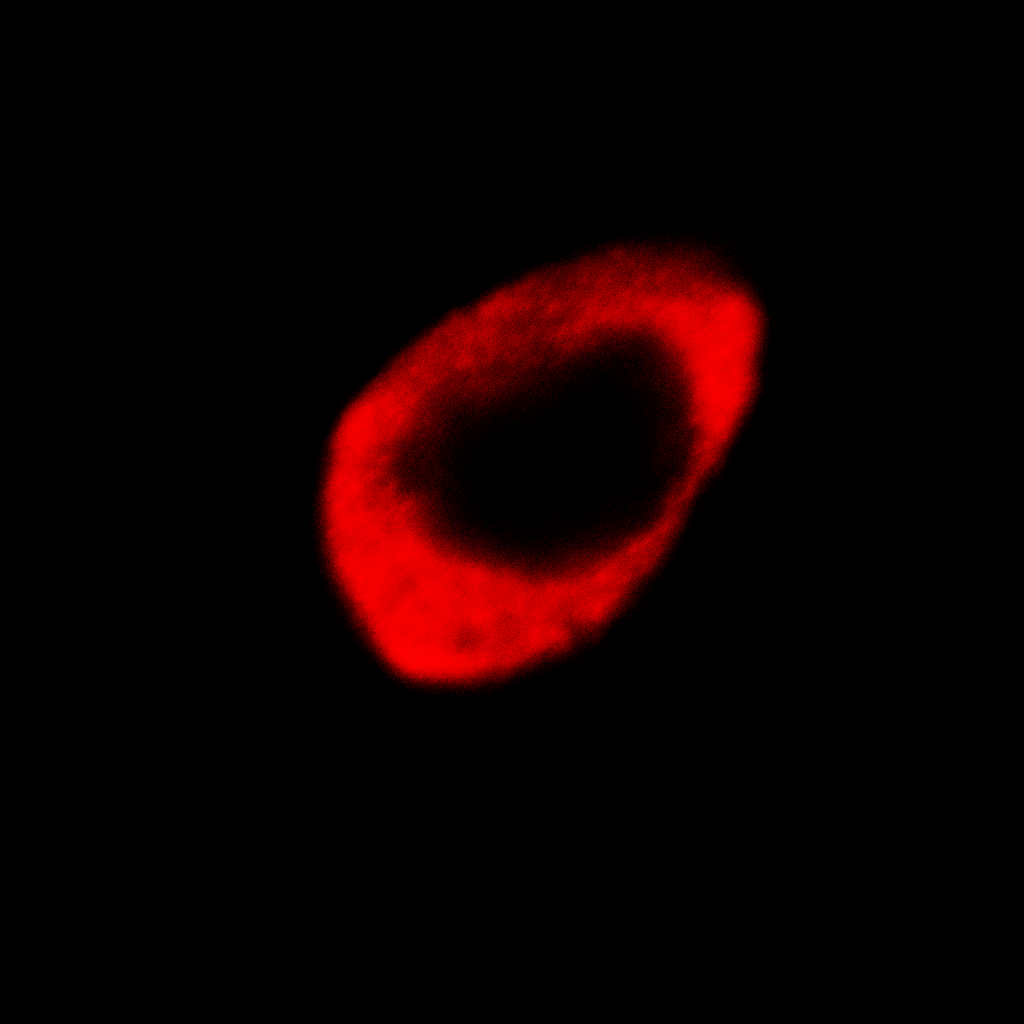

Supplement: S2 Data — (ZIP) [file ppat.1012546.s006.zip › Figure 5D/1/Flag-NLRP3/Flag-NLRP3 .tif]

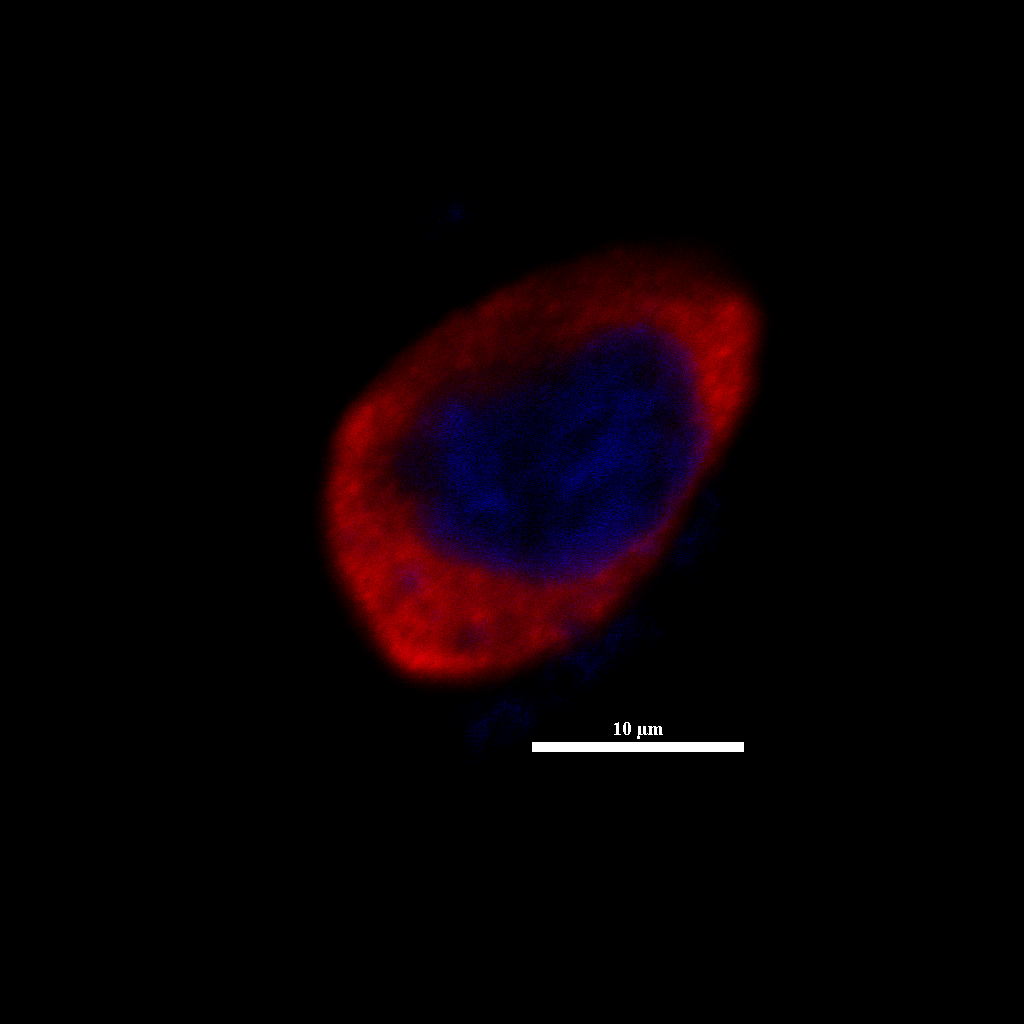

Supplement: S2 Data — (ZIP) [file ppat.1012546.s006.zip › Figure 5D/1/Flag-NLRP3/Merge.tif]

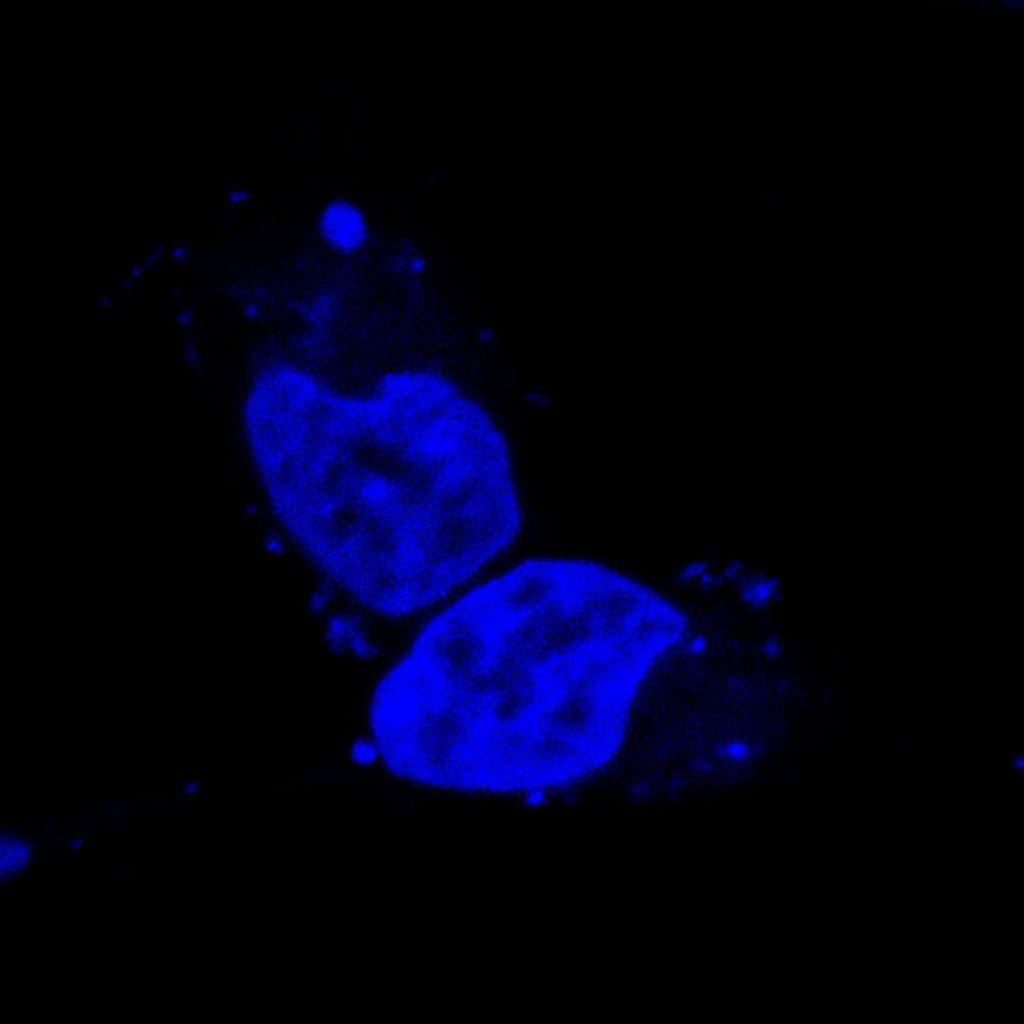

Supplement: S2 Data — (ZIP) [file ppat.1012546.s006.zip › Figure 5D/1/Flag-NLRP3+GFP-UL4/DAPI.tif]

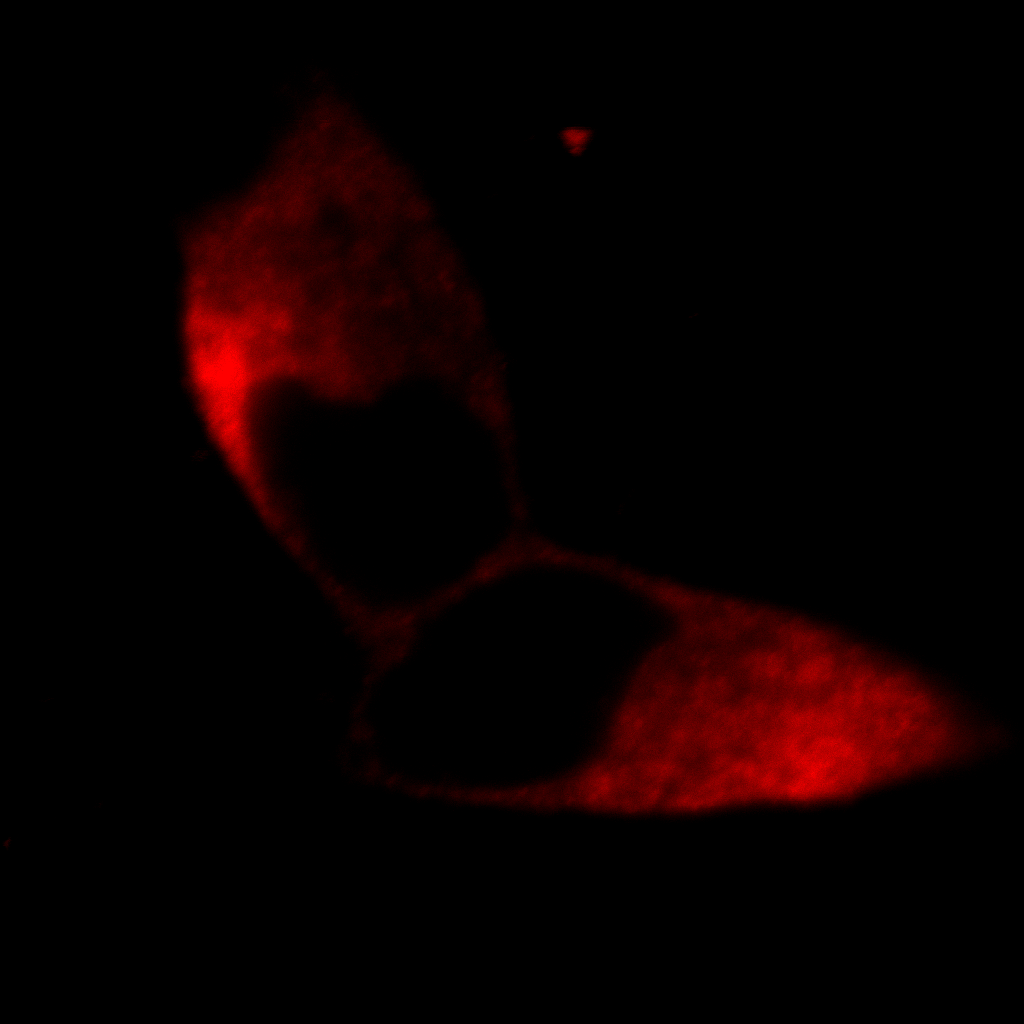

Supplement: S2 Data — (ZIP) [file ppat.1012546.s006.zip › Figure 5D/1/Flag-NLRP3+GFP-UL4/Flag-NLRP3.tif]

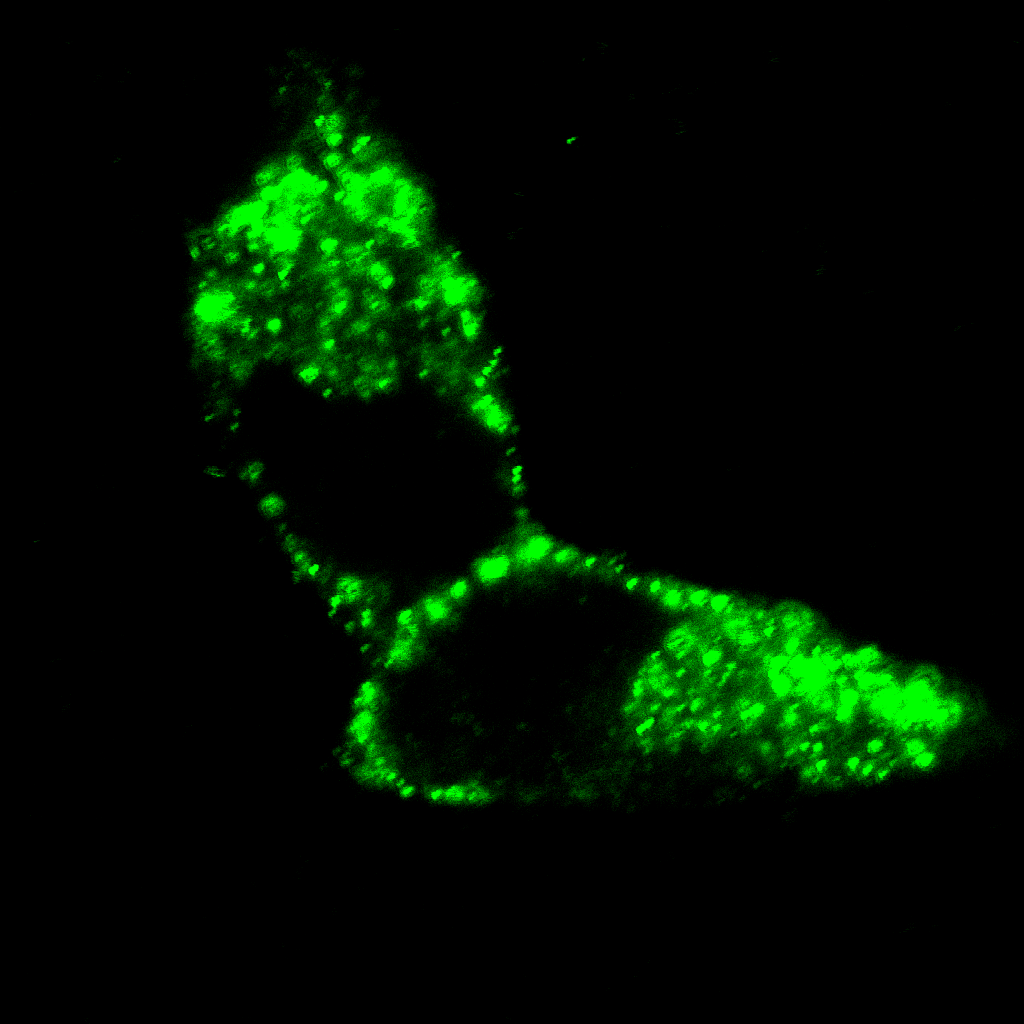

Supplement: S2 Data — (ZIP) [file ppat.1012546.s006.zip › Figure 5D/1/Flag-NLRP3+GFP-UL4/GFP-UL4.tif]

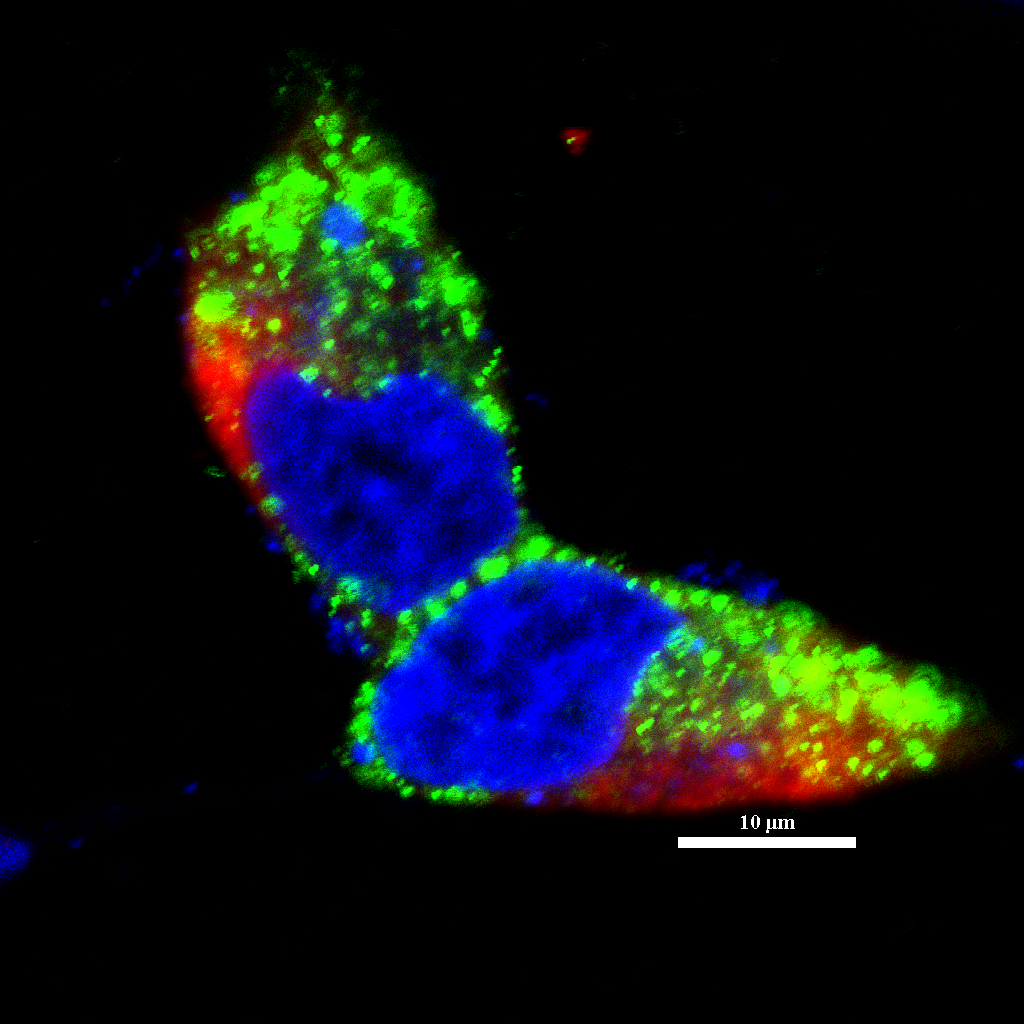

Supplement: S2 Data — (ZIP) [file ppat.1012546.s006.zip › Figure 5D/1/Flag-NLRP3+GFP-UL4/Merge.tif]

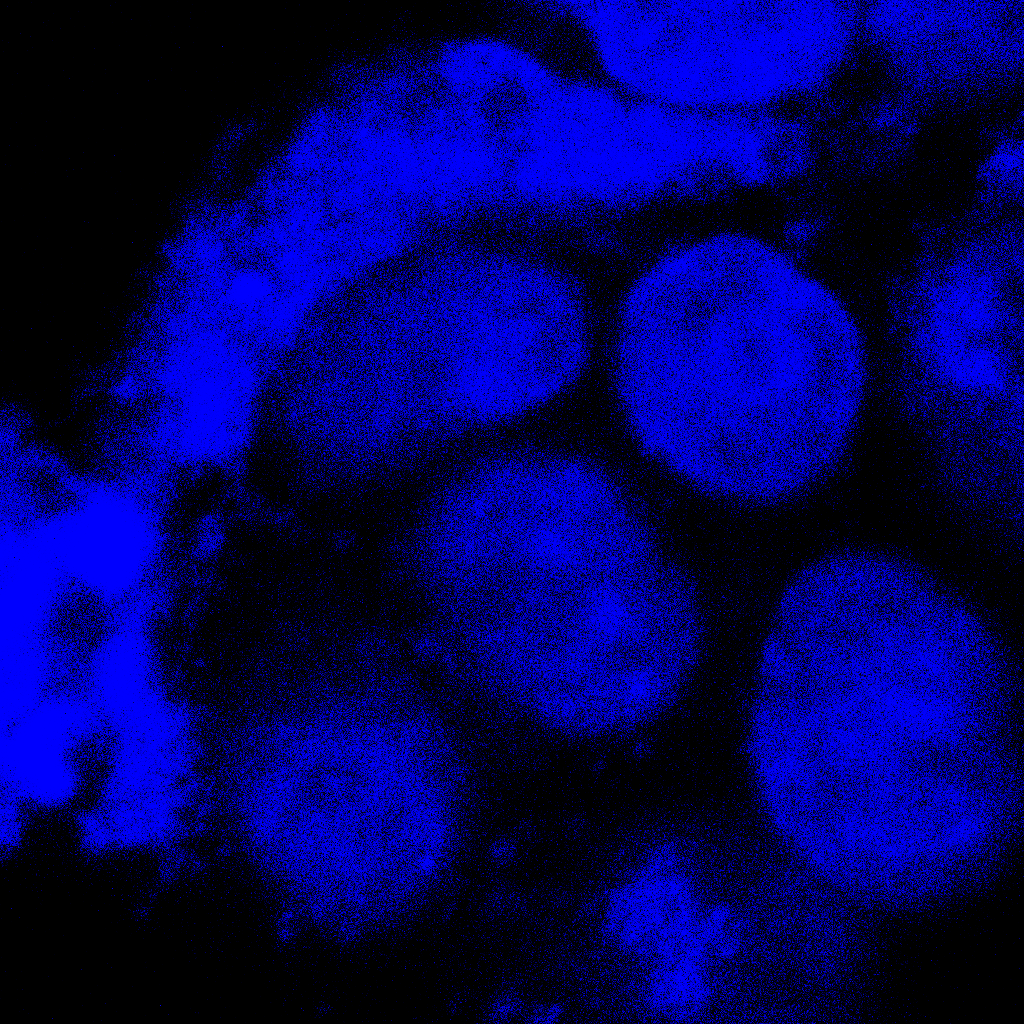

Supplement: S2 Data — (ZIP) [file ppat.1012546.s006.zip › Figure 5D/1/GFP-UL4/DAPI.tif]

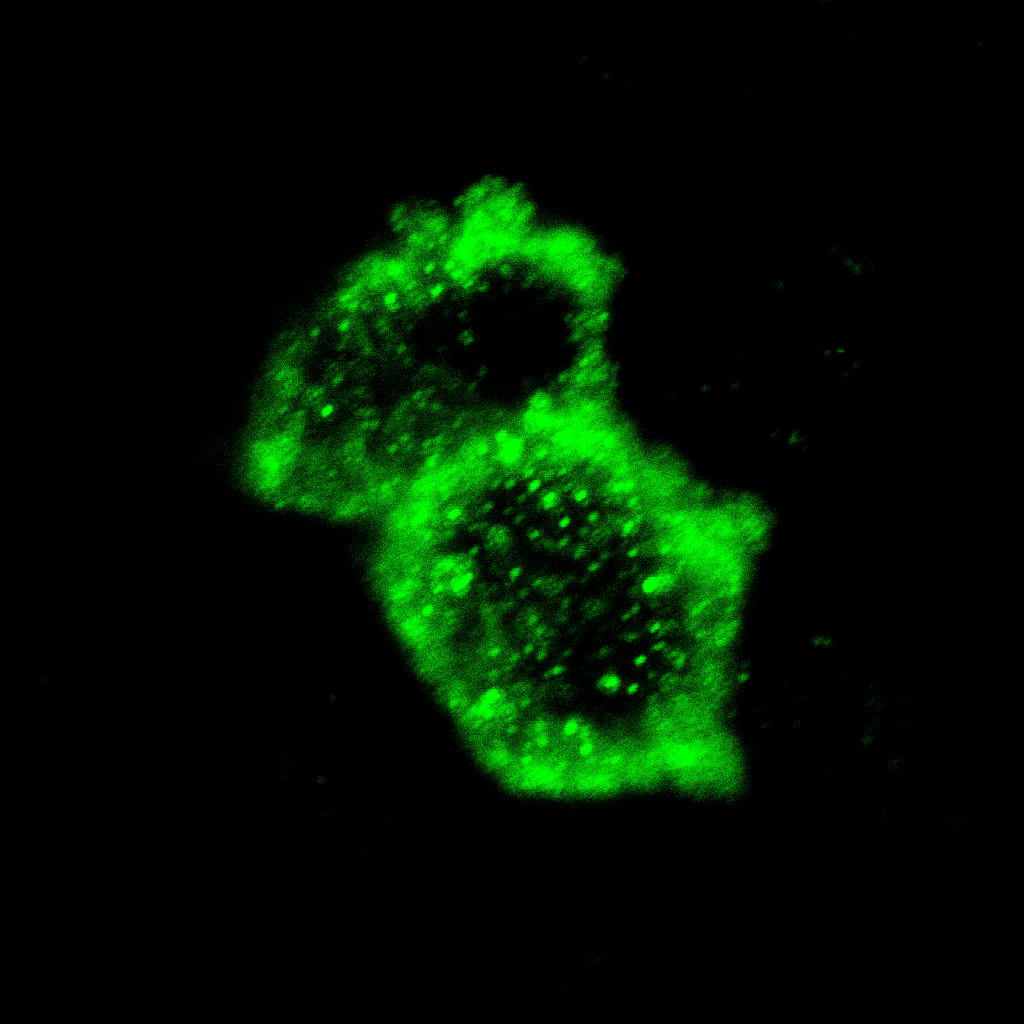

Supplement: S2 Data — (ZIP) [file ppat.1012546.s006.zip › Figure 5D/1/GFP-UL4/GFP-UL4.tif]

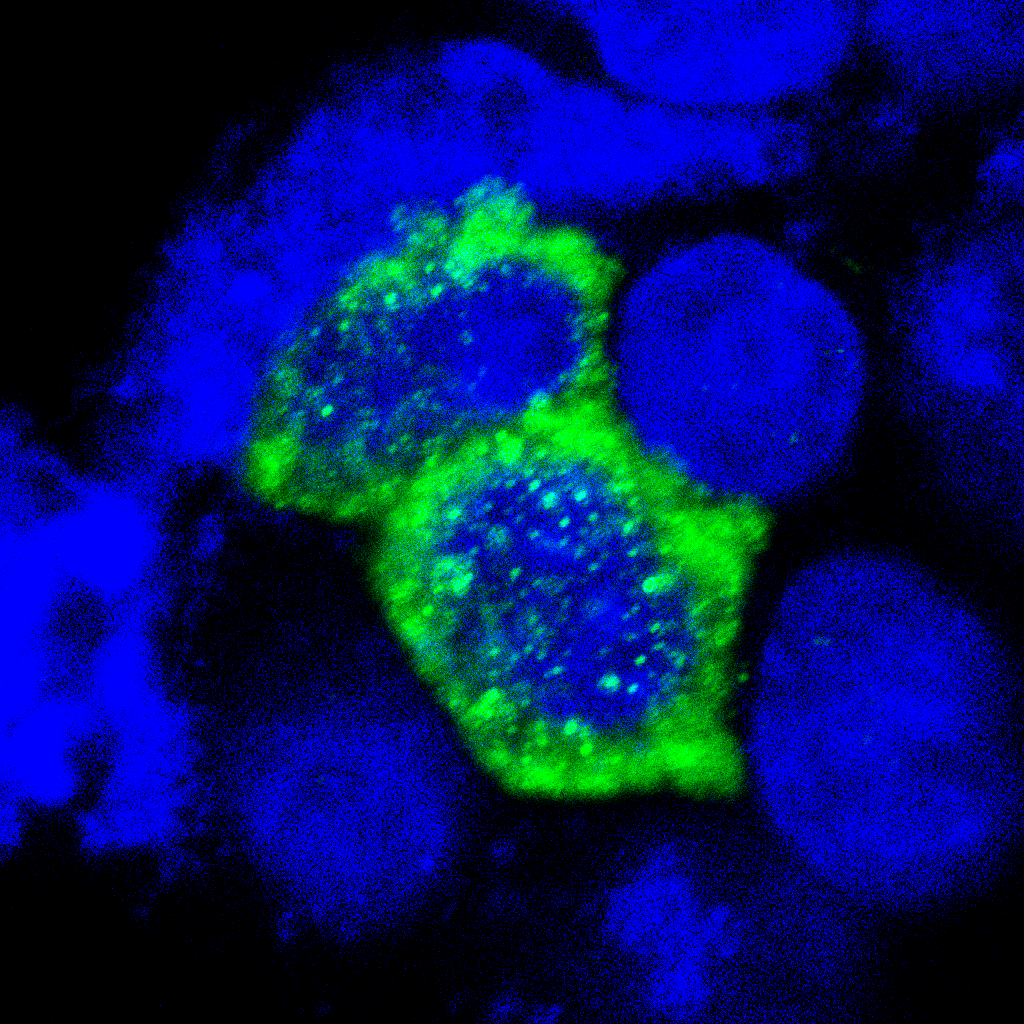

Supplement: S2 Data — (ZIP) [file ppat.1012546.s006.zip › Figure 5D/1/GFP-UL4/Merge.tif]

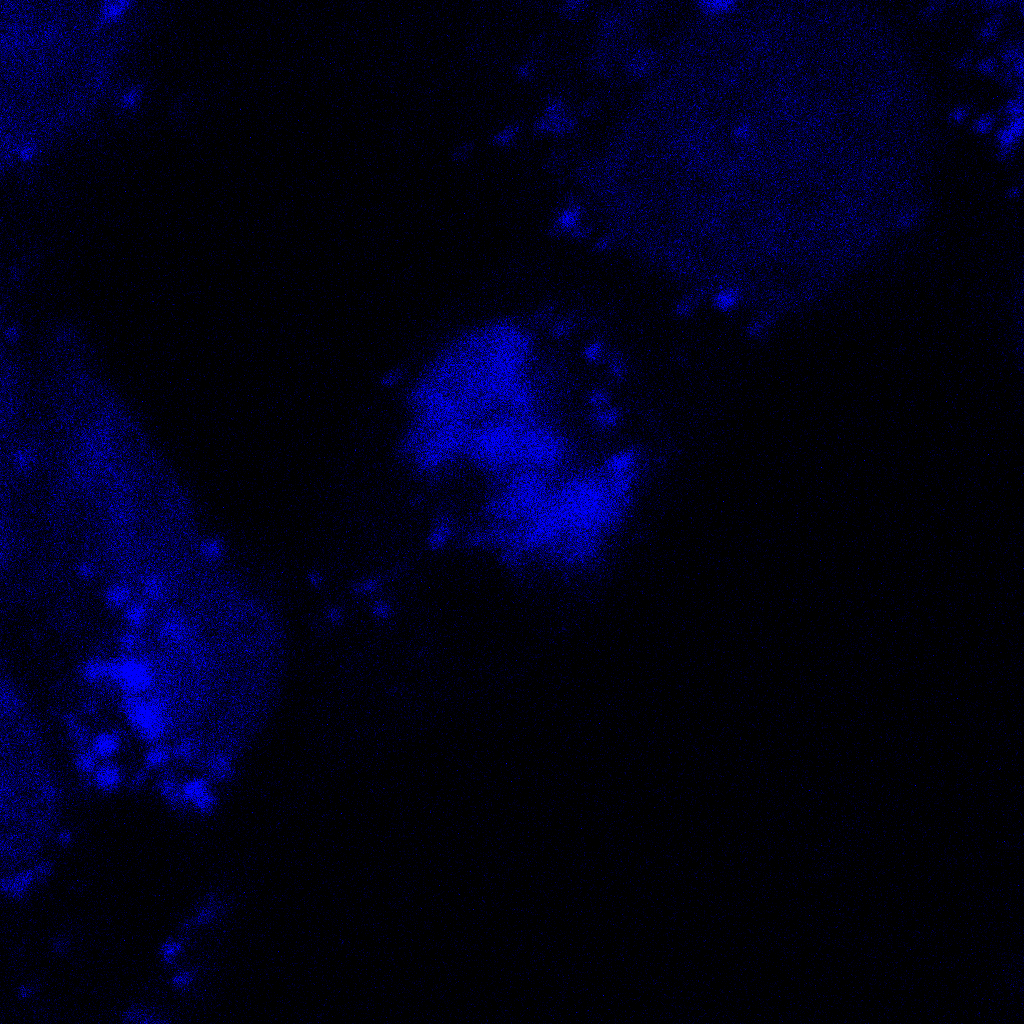

Supplement: S2 Data — (ZIP) [file ppat.1012546.s006.zip › Figure 5D/2/Flag-ASC/DAPI.tif]

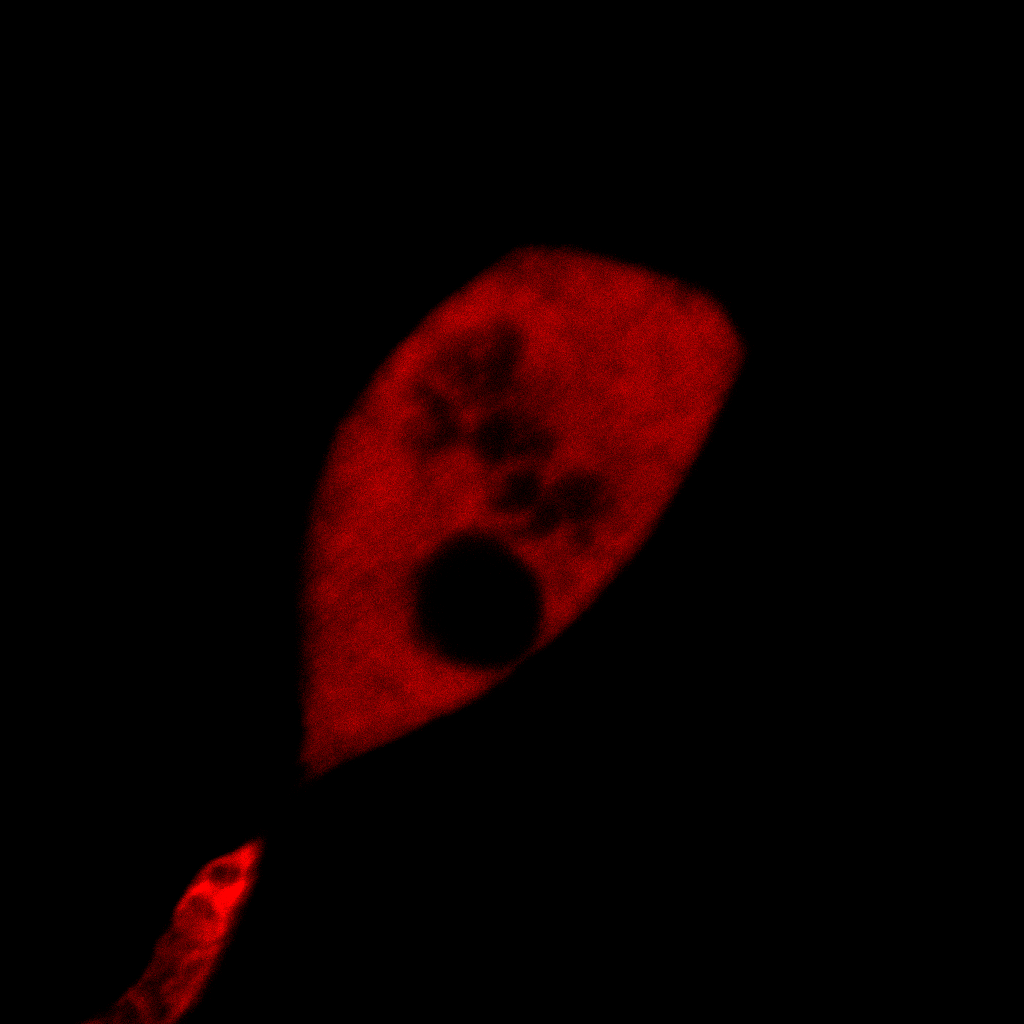

Supplement: S2 Data — (ZIP) [file ppat.1012546.s006.zip › Figure 5D/2/Flag-ASC/flag-asc.tif]

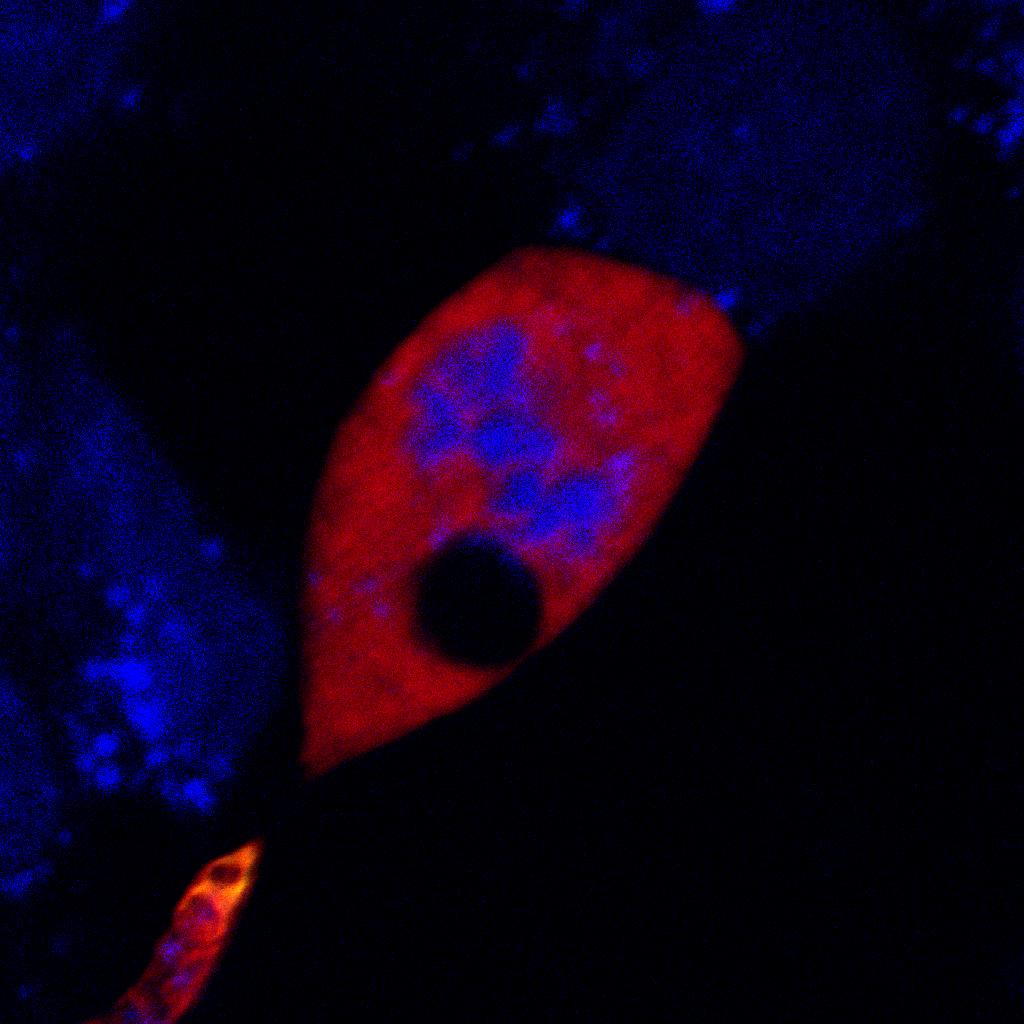

Supplement: S2 Data — (ZIP) [file ppat.1012546.s006.zip › Figure 5D/2/Flag-ASC/Merge.tif]

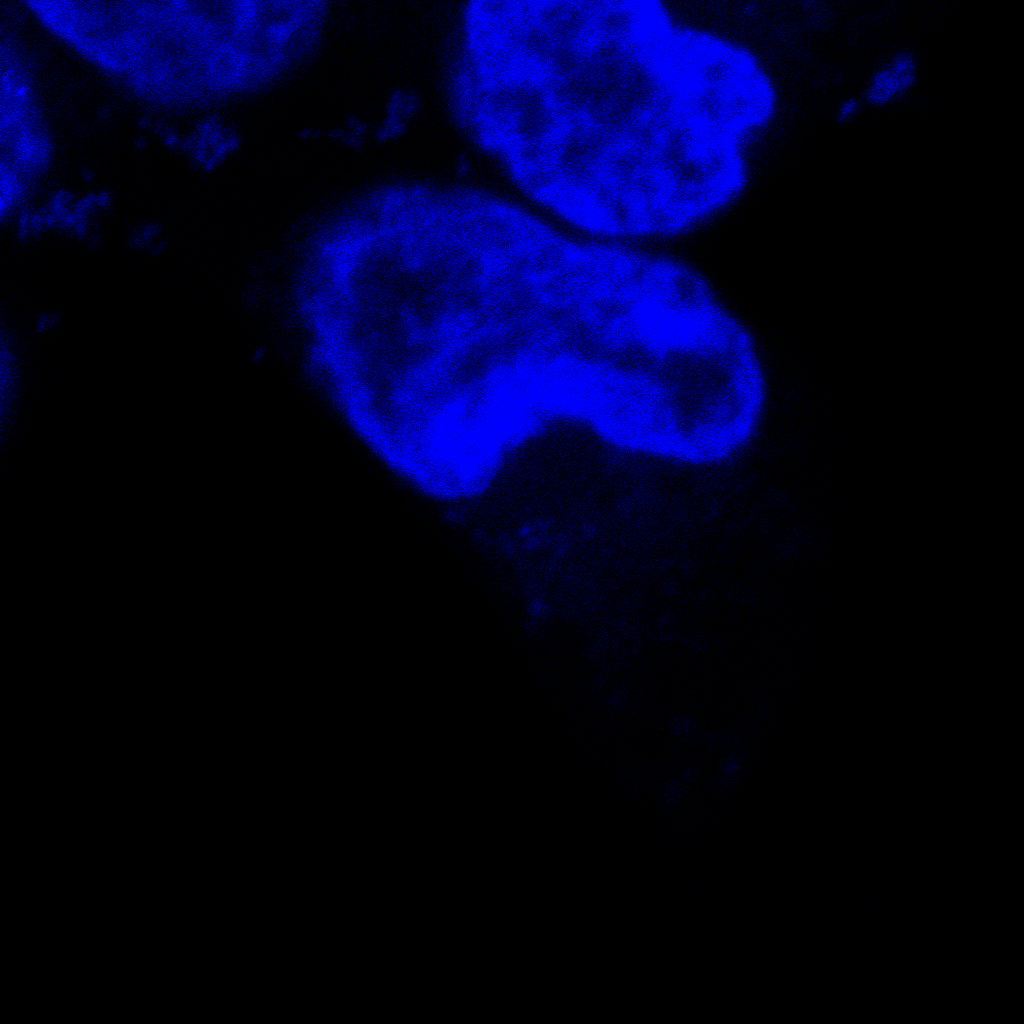

Supplement: S2 Data — (ZIP) [file ppat.1012546.s006.zip › Figure 5D/2/Flag-ASC+GFP-UL4/1/DAPI.tif]

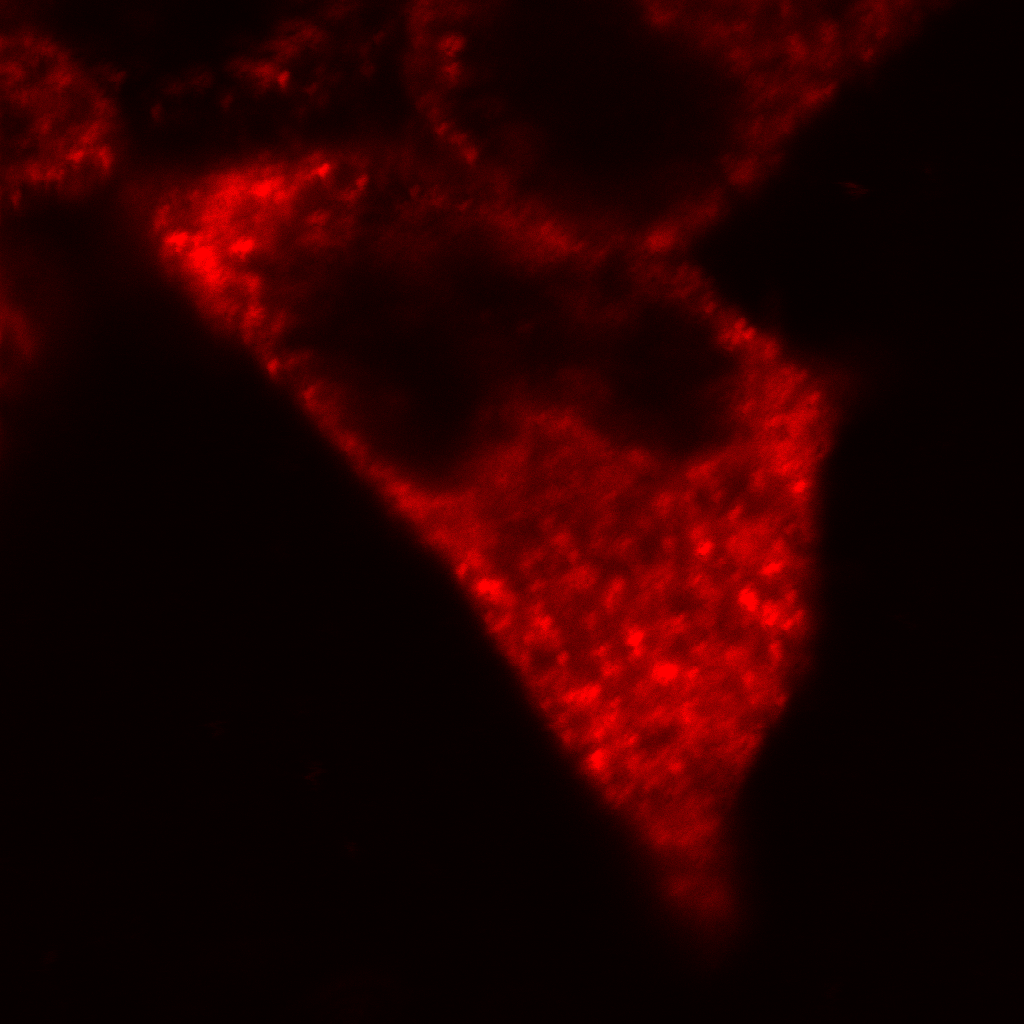

Supplement: S2 Data — (ZIP) [file ppat.1012546.s006.zip › Figure 5D/2/Flag-ASC+GFP-UL4/1/flag-asc.tif]

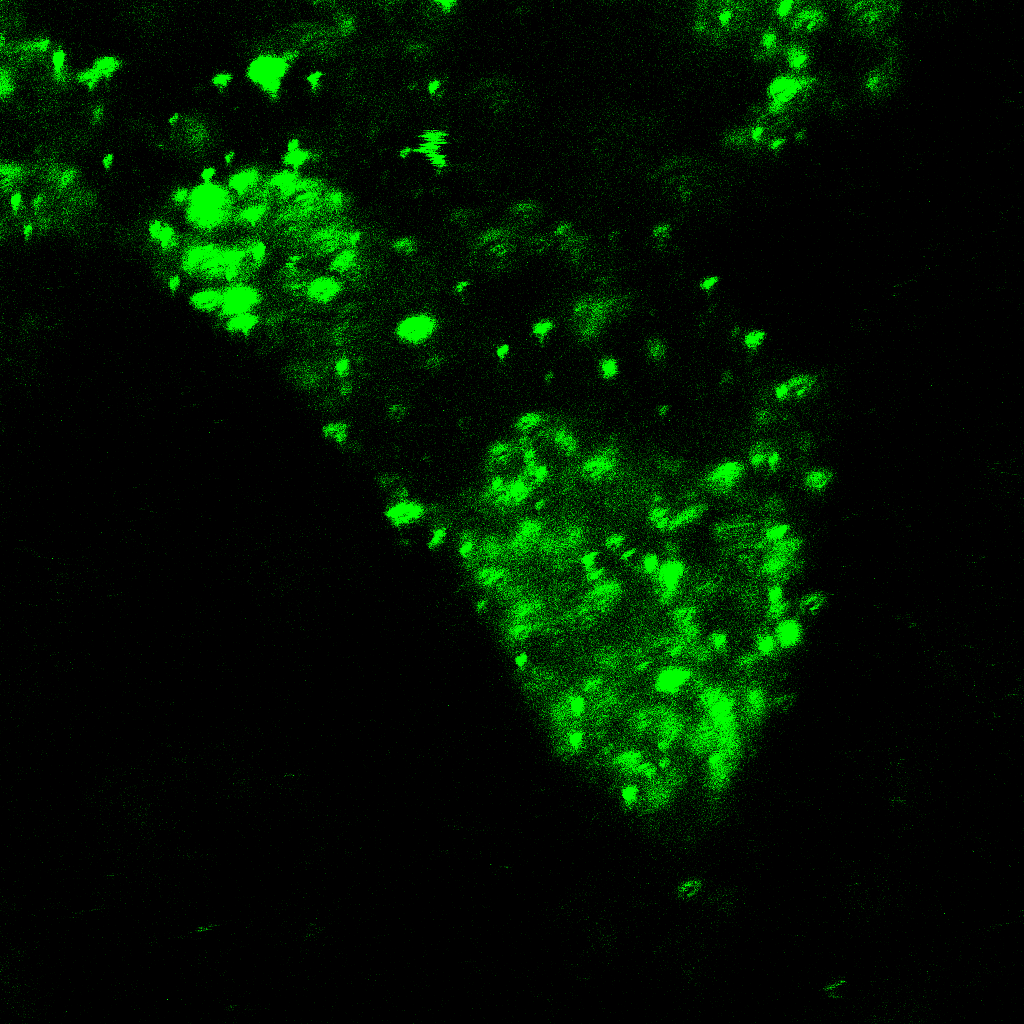

Supplement: S2 Data — (ZIP) [file ppat.1012546.s006.zip › Figure 5D/2/Flag-ASC+GFP-UL4/1/GFP-UL4.tif]

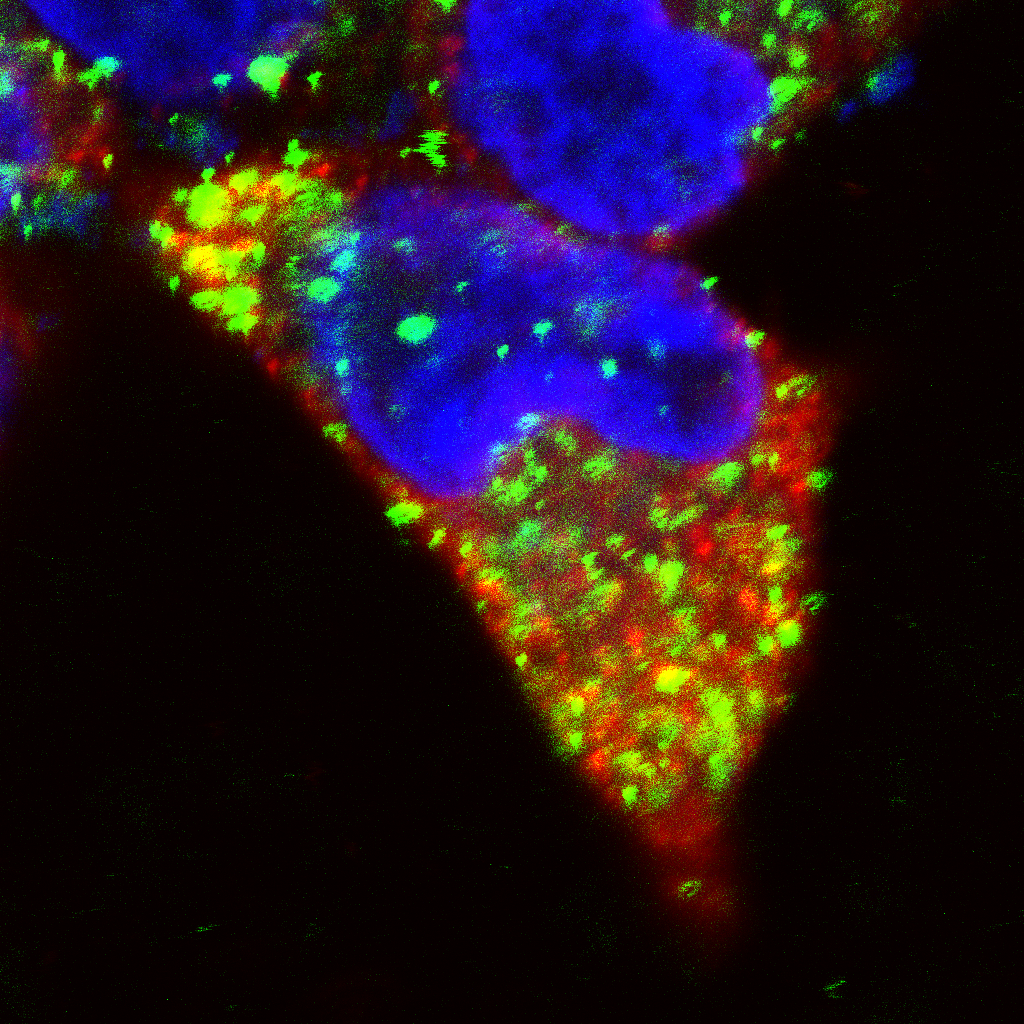

Supplement: S2 Data — (ZIP) [file ppat.1012546.s006.zip › Figure 5D/2/Flag-ASC+GFP-UL4/1/Merge.tif]

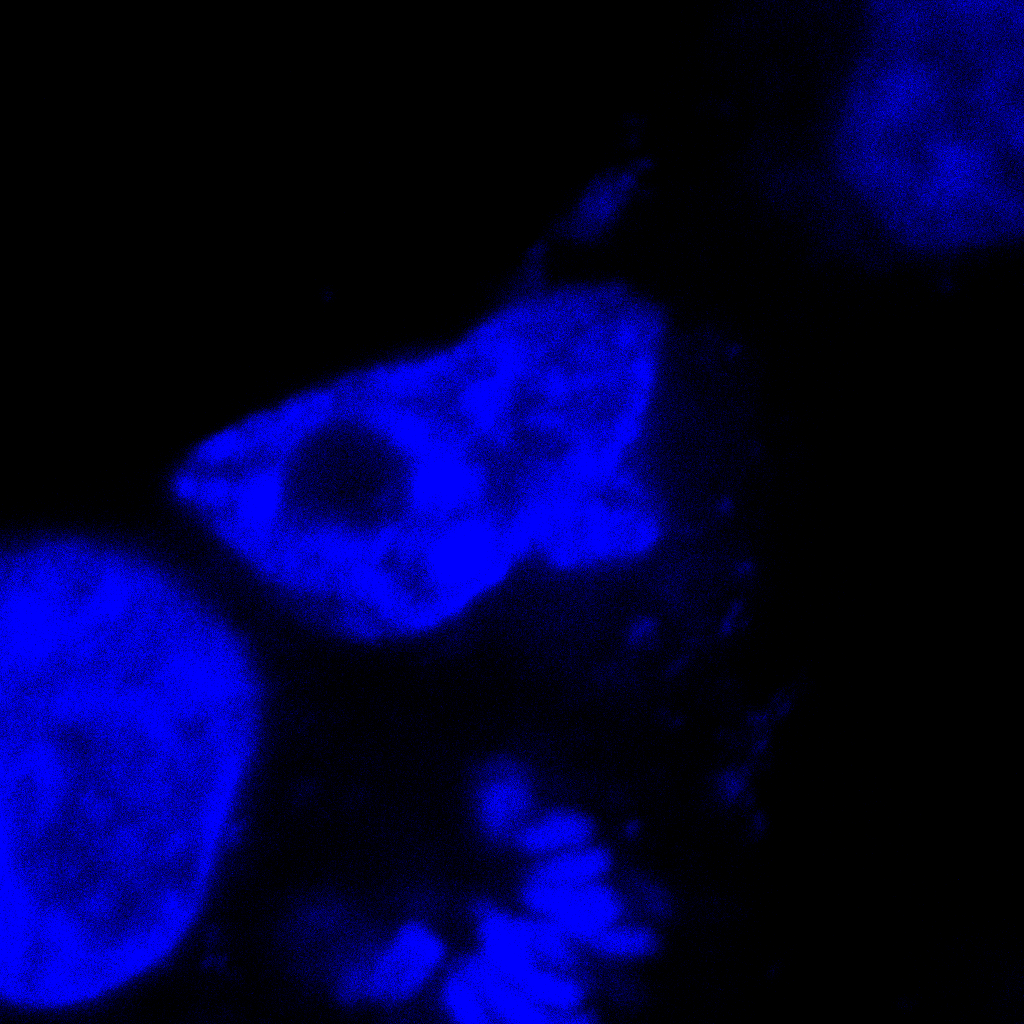

Supplement: S2 Data — (ZIP) [file ppat.1012546.s006.zip › Figure 5D/2/Flag-ASC+GFP-UL4/2/DAPI.tif]

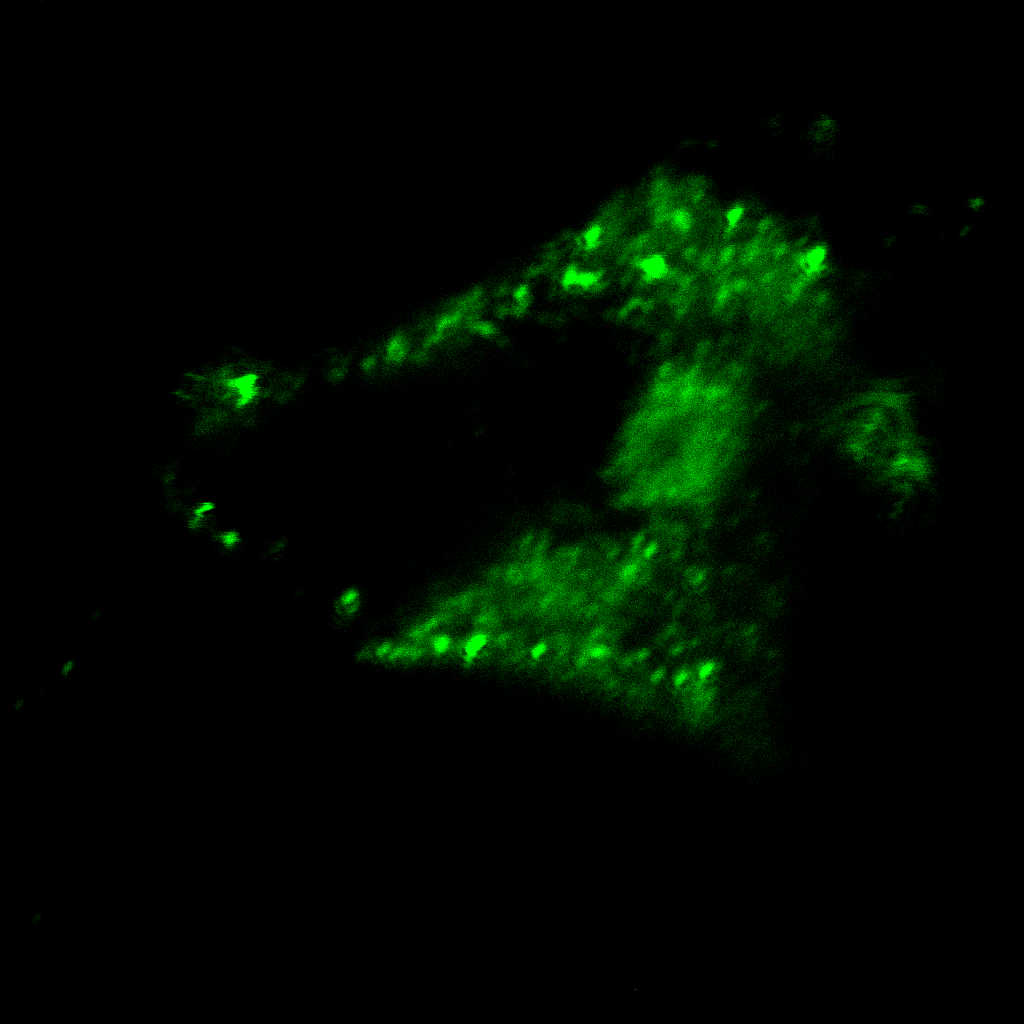

Supplement: S2 Data — (ZIP) [file ppat.1012546.s006.zip › Figure 5D/2/Flag-ASC+GFP-UL4/2/GFP-UL4.tif]

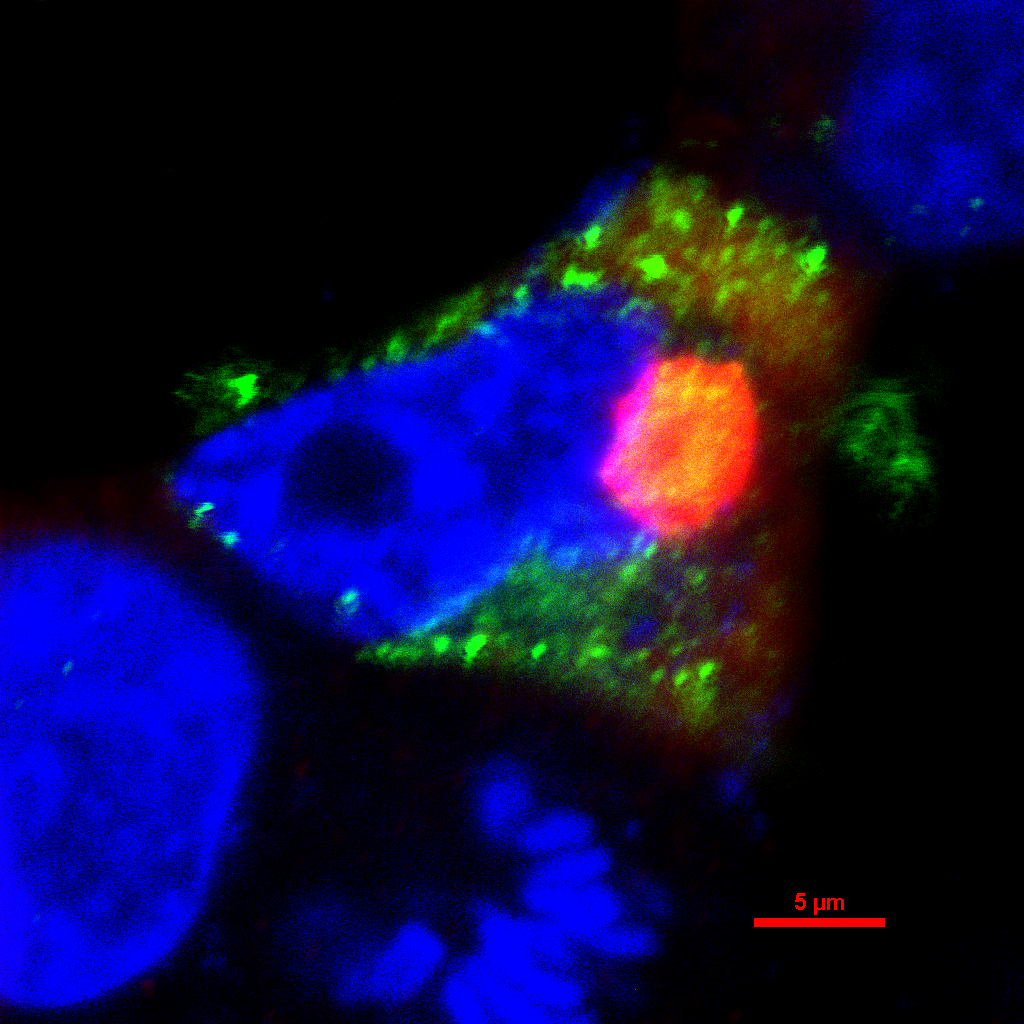

Supplement: S2 Data — (ZIP) [file ppat.1012546.s006.zip › Figure 5D/2/Flag-ASC+GFP-UL4/2/Merge.tif]

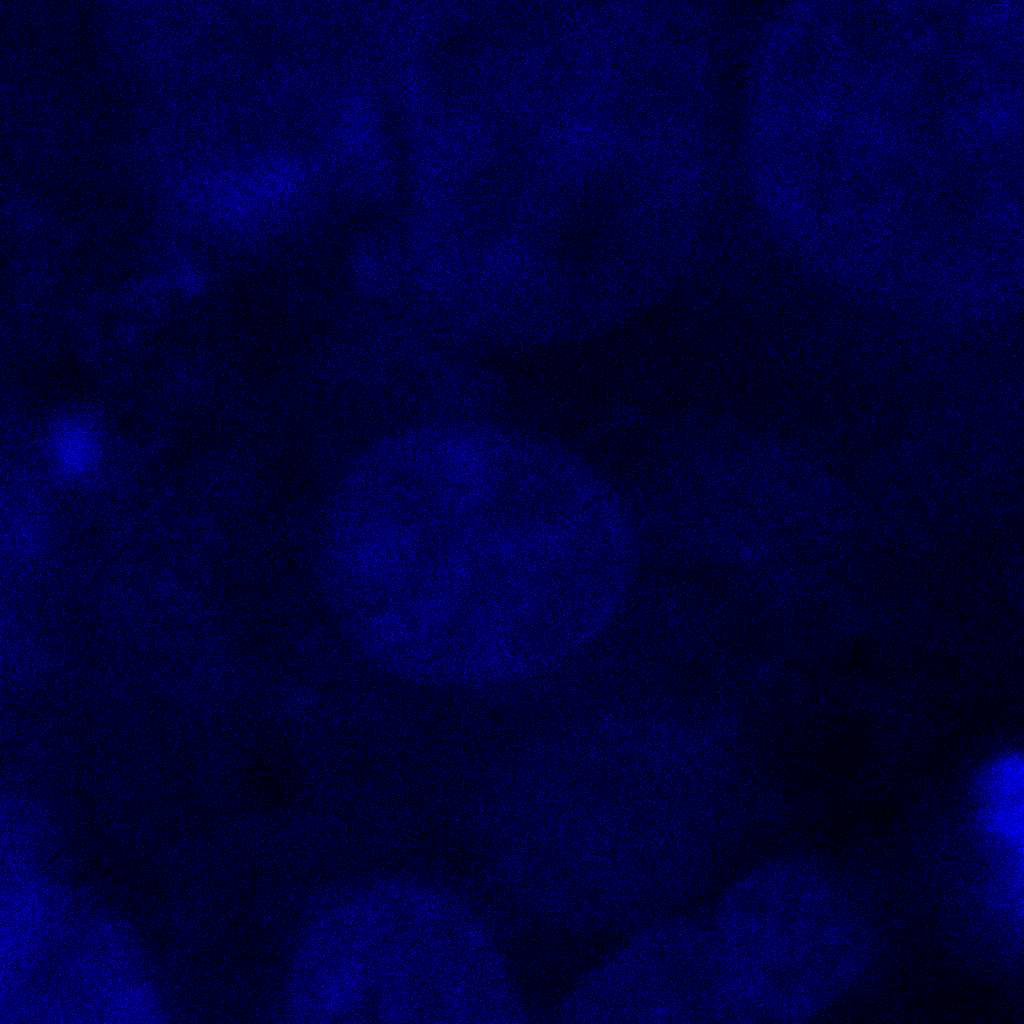

Supplement: S2 Data — (ZIP) [file ppat.1012546.s006.zip › Figure 5D/2/Flag-ASC-2/DAPI.tif]

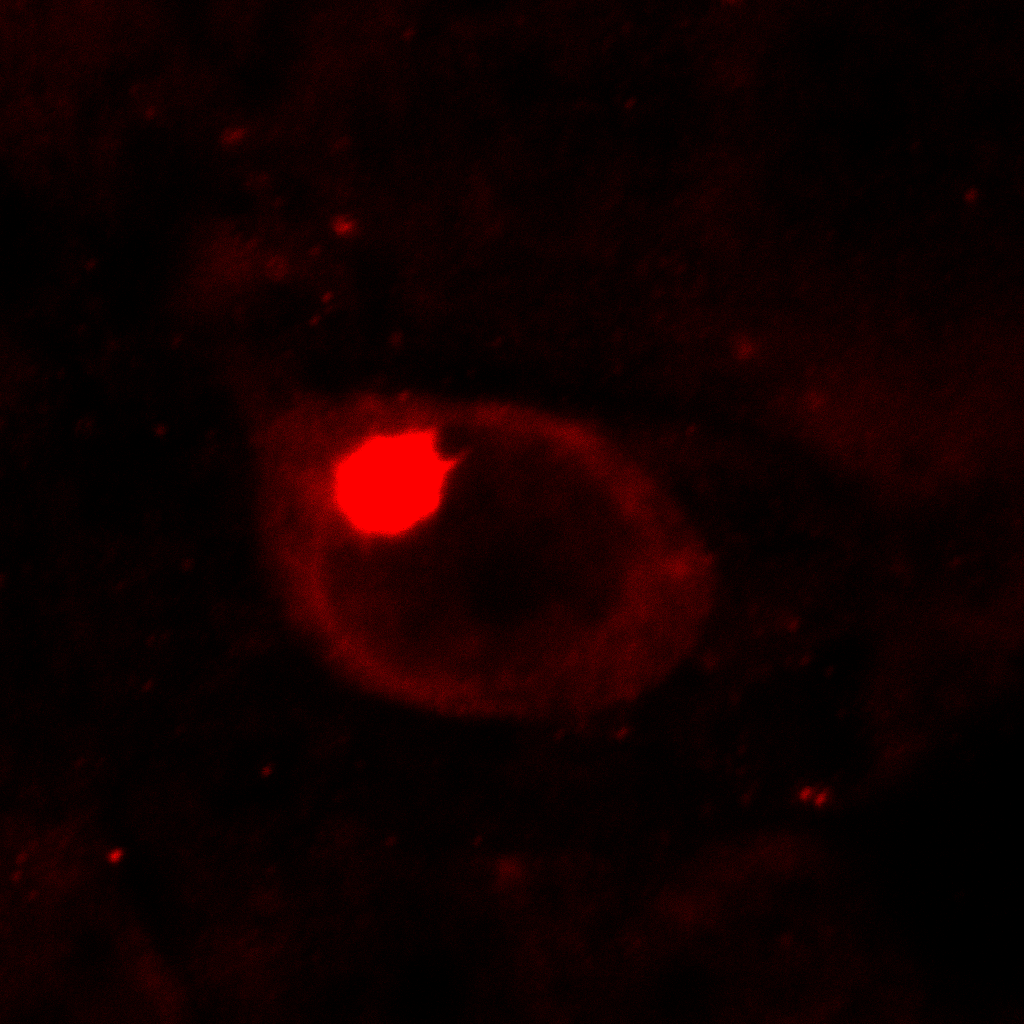

Supplement: S2 Data — (ZIP) [file ppat.1012546.s006.zip › Figure 5D/2/Flag-ASC-2/flag-asc.tif]

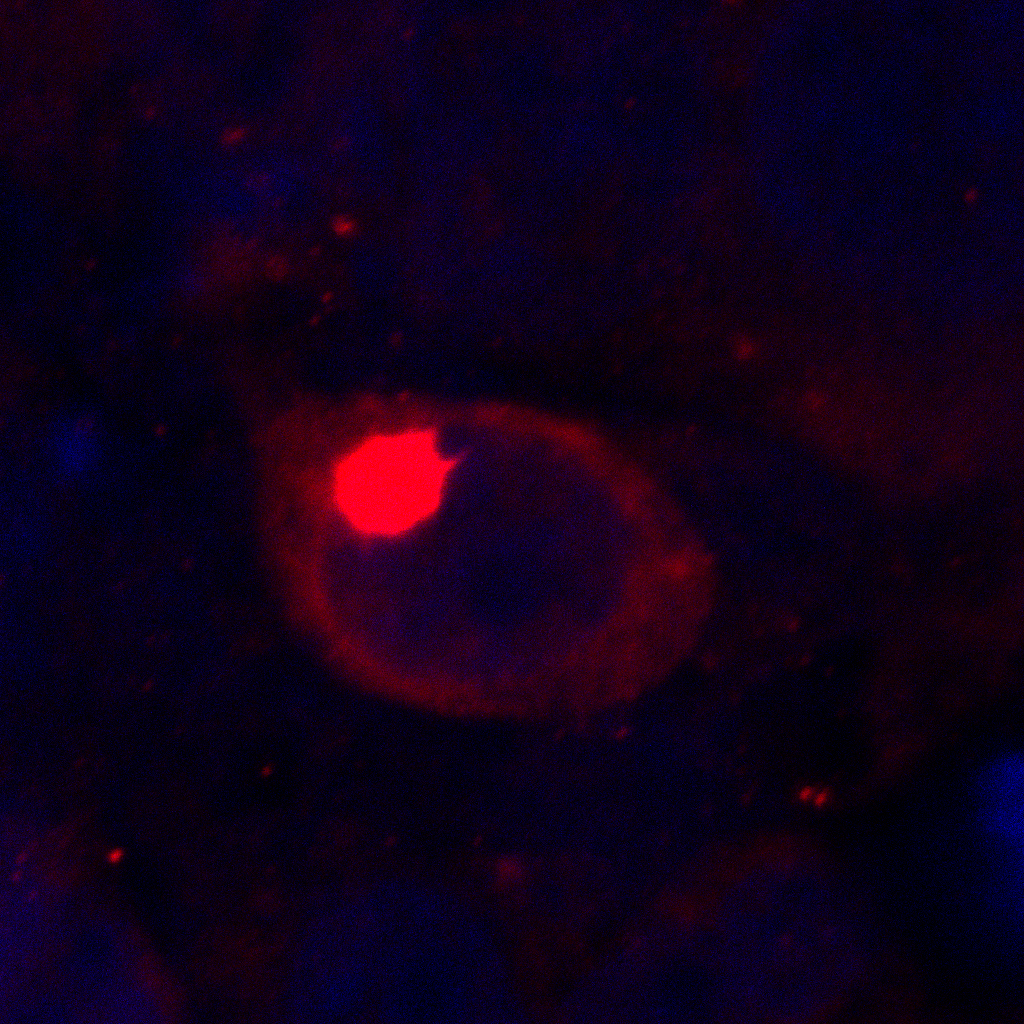

Supplement: S2 Data — (ZIP) [file ppat.1012546.s006.zip › Figure 5D/2/Flag-ASC-2/Merge.tif]

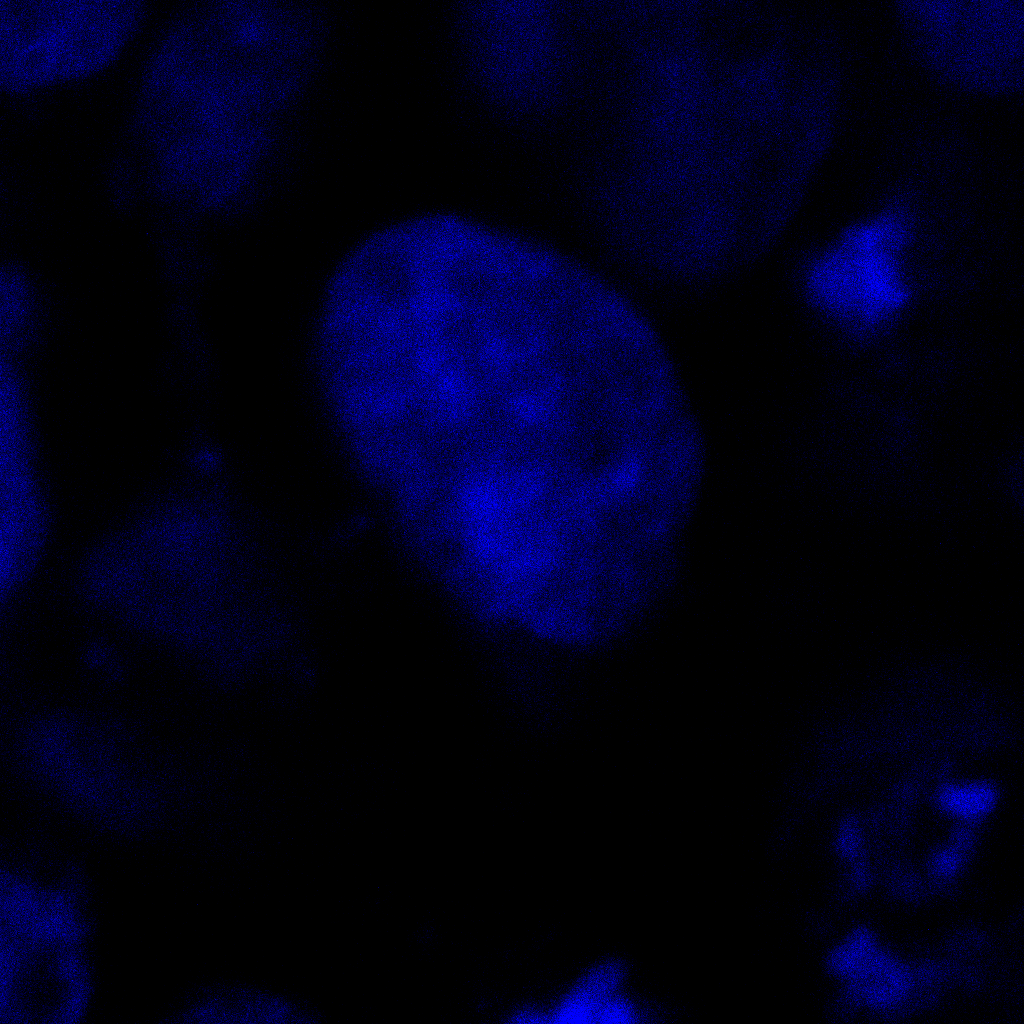

Supplement: S2 Data — (ZIP) [file ppat.1012546.s006.zip › Figure 5D/2/Flag-CASP1/DAPI.tif]

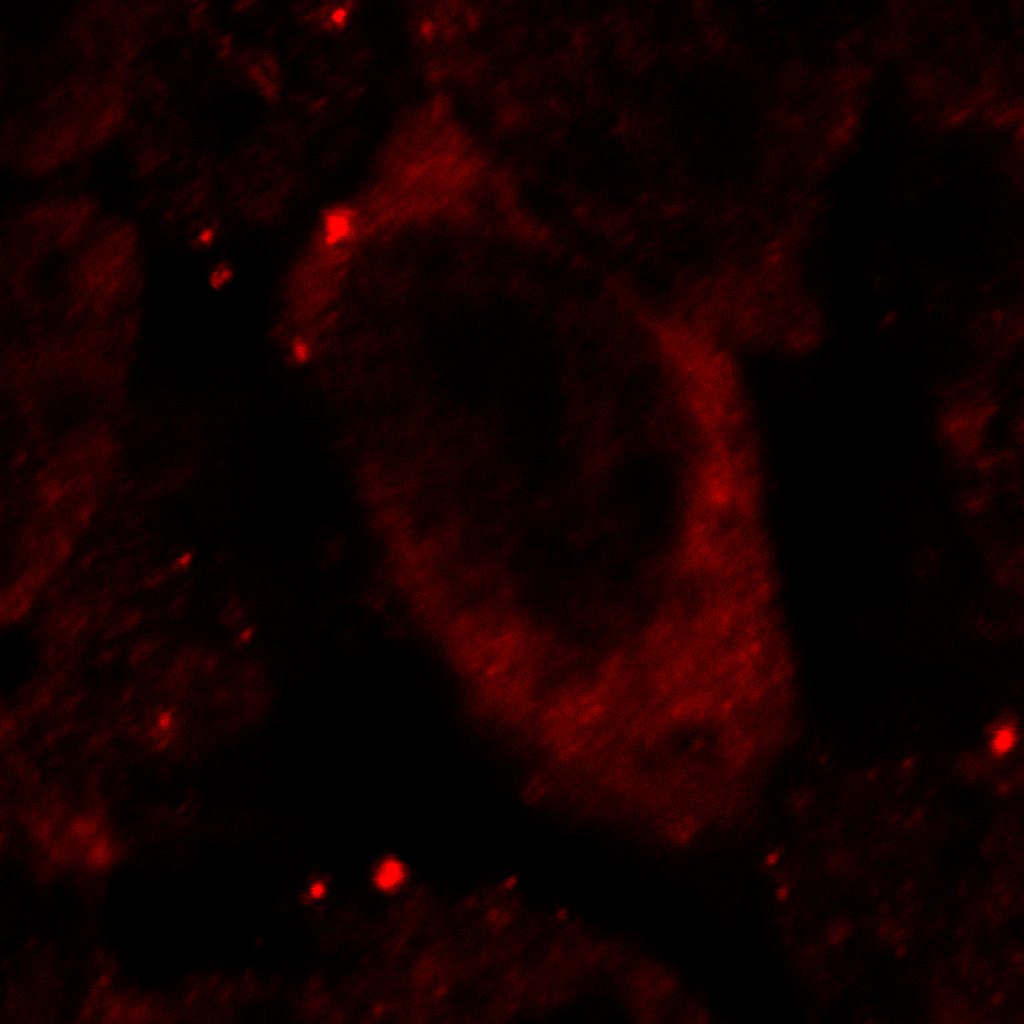

Supplement: S2 Data — (ZIP) [file ppat.1012546.s006.zip › Figure 5D/2/Flag-CASP1/flag-casp1.tif]

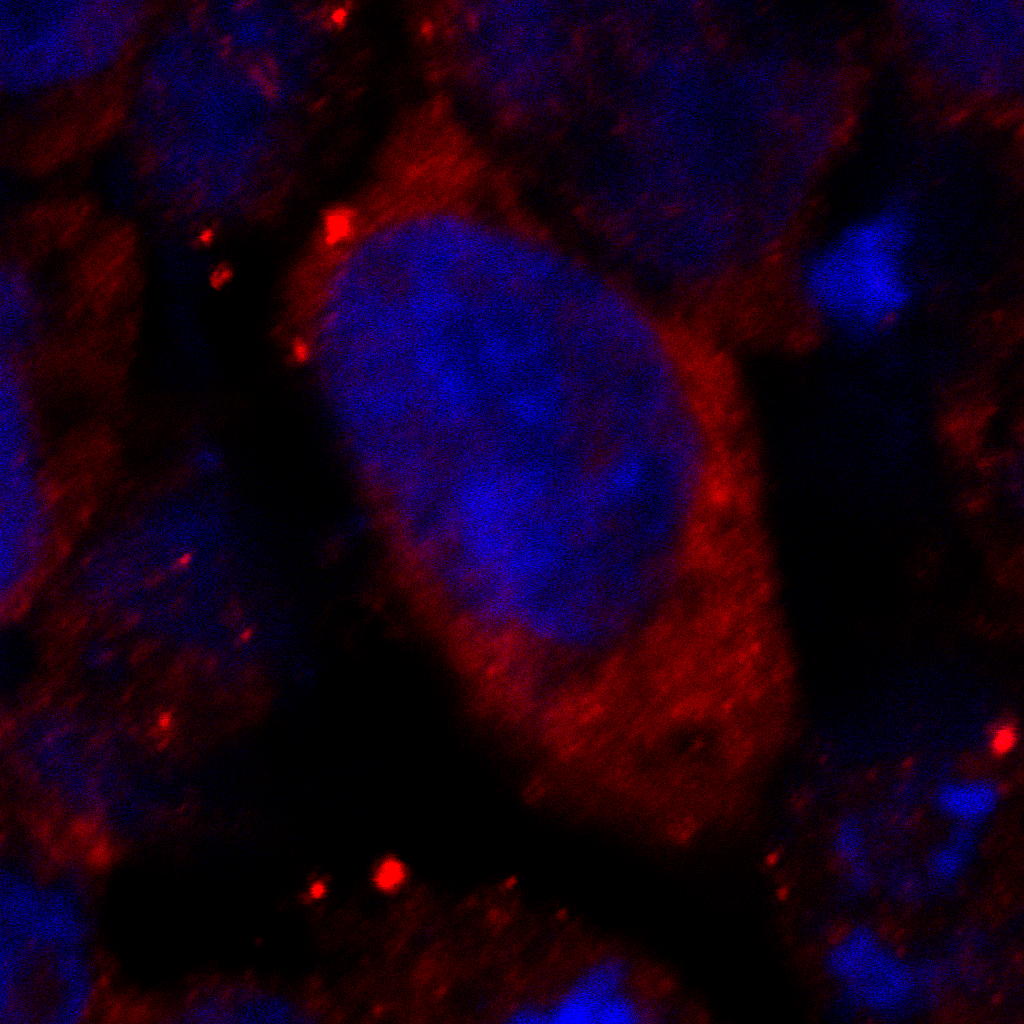

Supplement: S2 Data — (ZIP) [file ppat.1012546.s006.zip › Figure 5D/2/Flag-CASP1/Merge.tif]

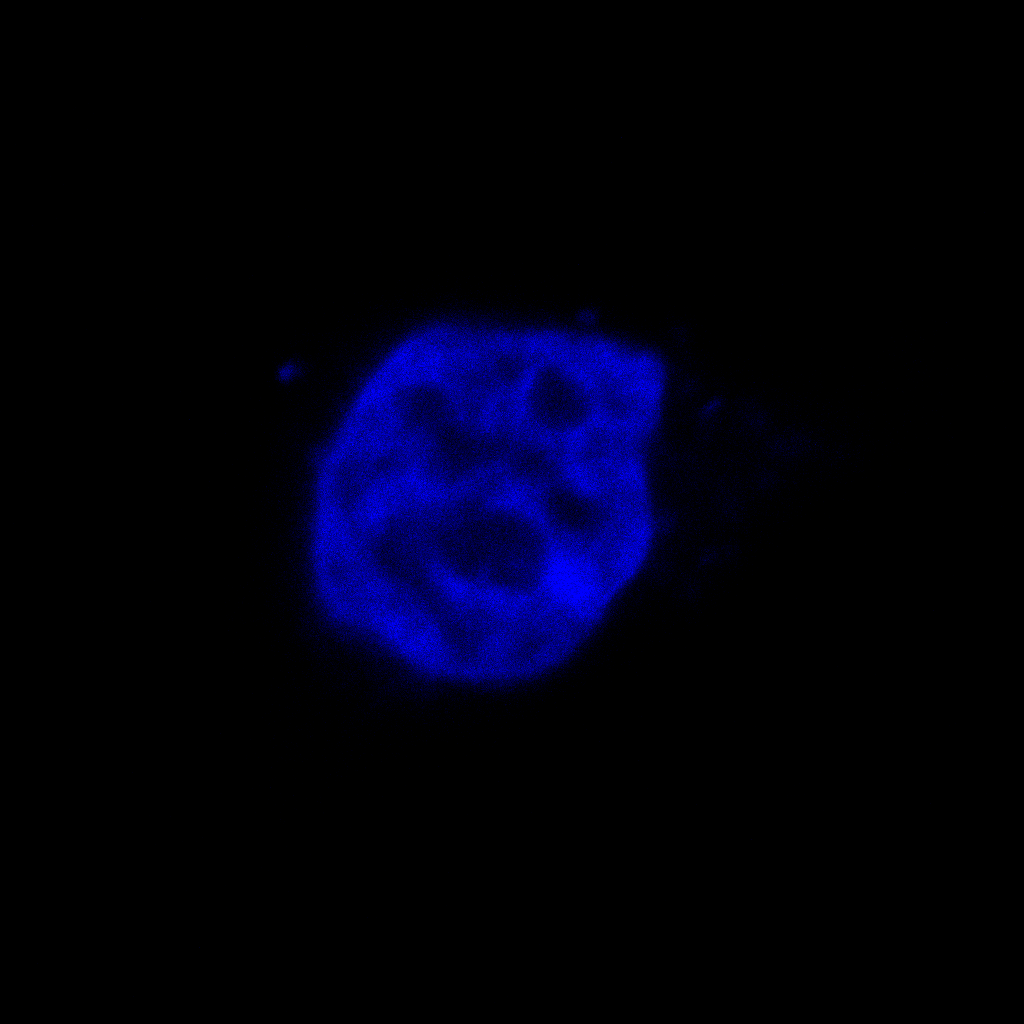

Supplement: S2 Data — (ZIP) [file ppat.1012546.s006.zip › Figure 5D/2/Flag-CASP1+GFP-UL4/DAPI.tif]

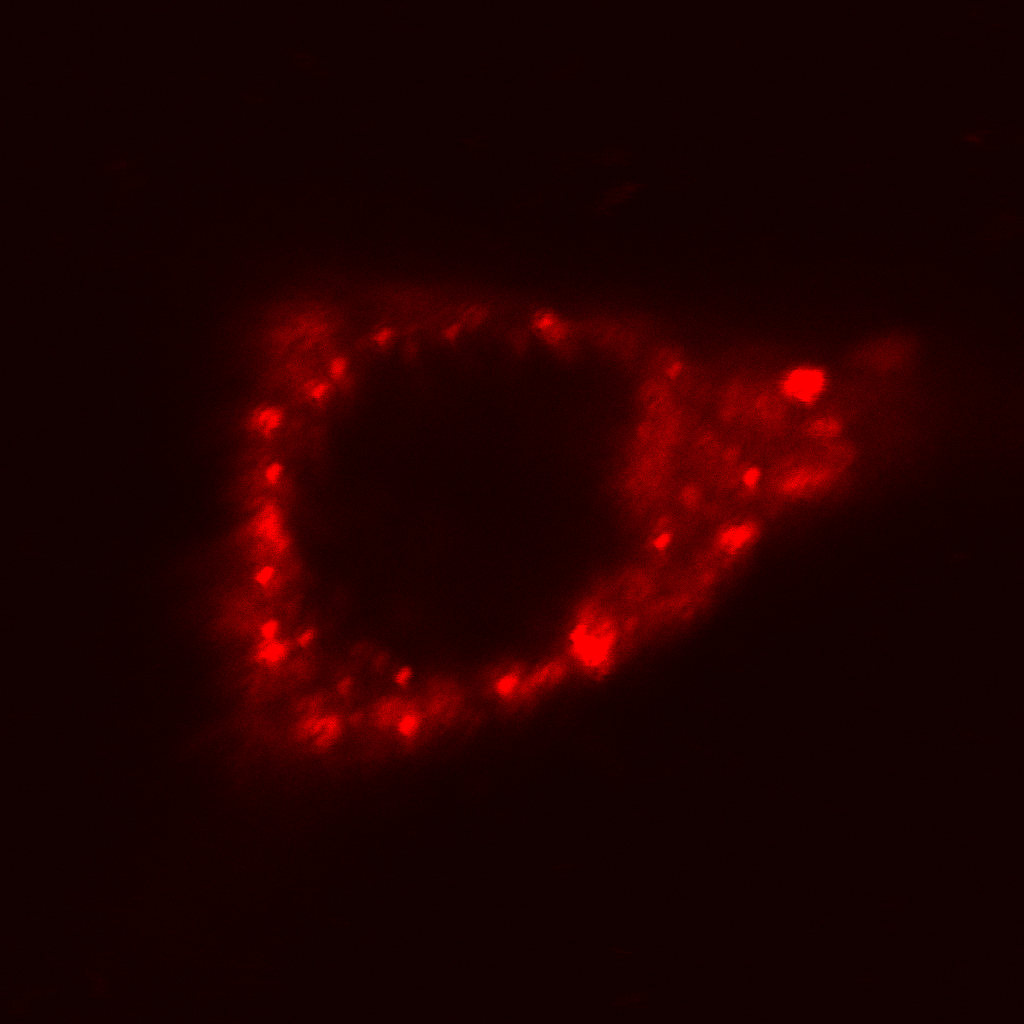

Supplement: S2 Data — (ZIP) [file ppat.1012546.s006.zip › Figure 5D/2/Flag-CASP1+GFP-UL4/flag-casp1.tif]
