# Supplementary figures and images for: Pseudorabies Virus UL4 protein promotes the ASC-dependent inflammasome activation and pyroptosis to exacerbate inflammation (part 3 of 6)
Source: PLoS Pathog. 2024 Sep 24;20(9):e1012546. doi: 10.1371/journal.ppat.1012546 (PMC11421794; doi:10.1371/journal.ppat.1012546)

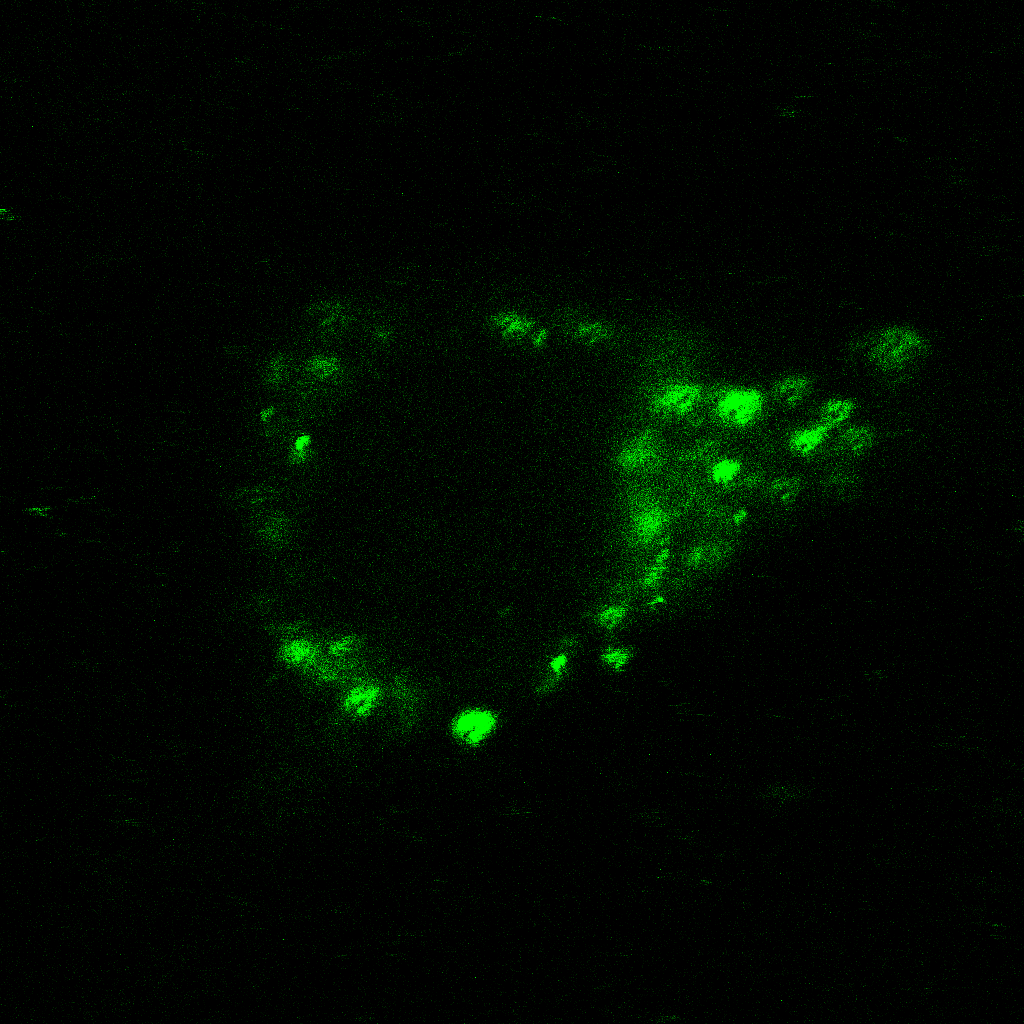

Supplement: S2 Data — (ZIP) [file ppat.1012546.s006.zip › Figure 5D/2/Flag-CASP1+GFP-UL4/gfp-ul4.tif]

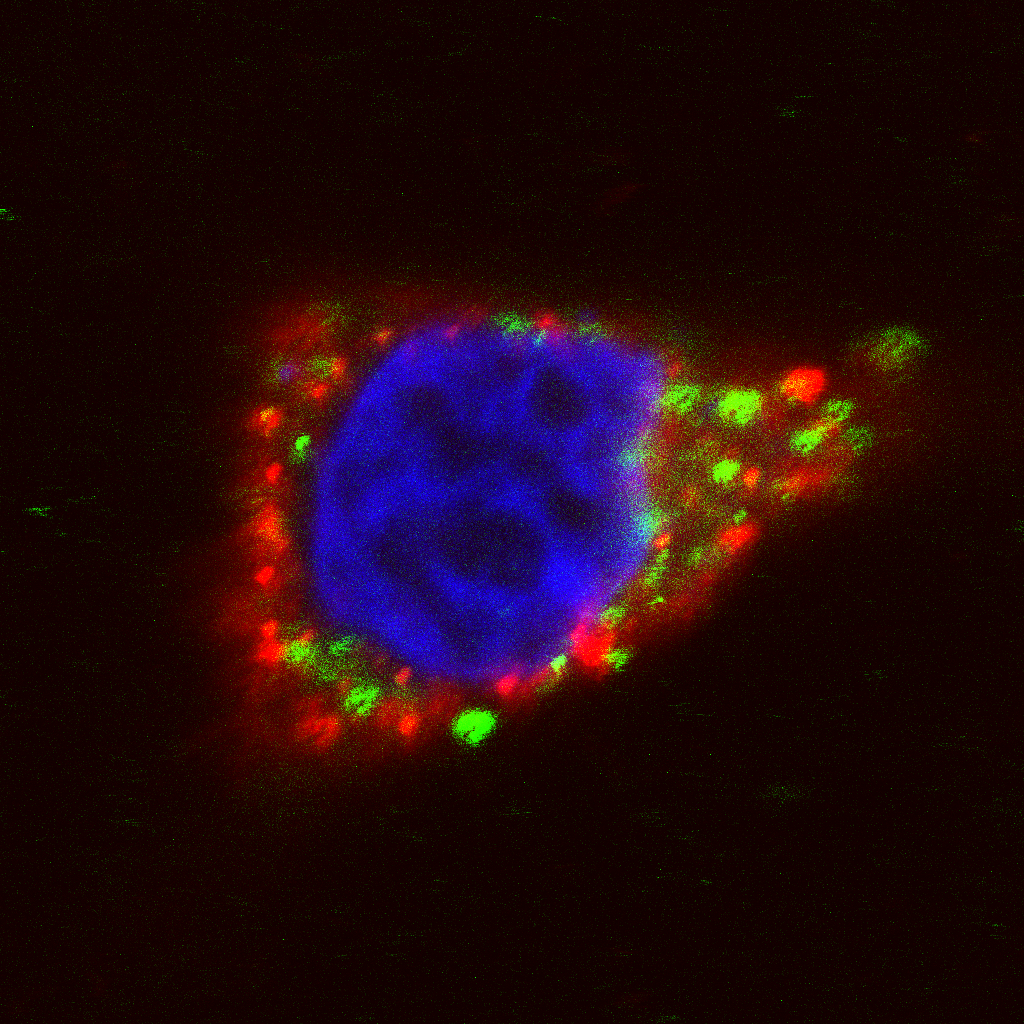

Supplement: S2 Data — (ZIP) [file ppat.1012546.s006.zip › Figure 5D/2/Flag-CASP1+GFP-UL4/Merge.tif]

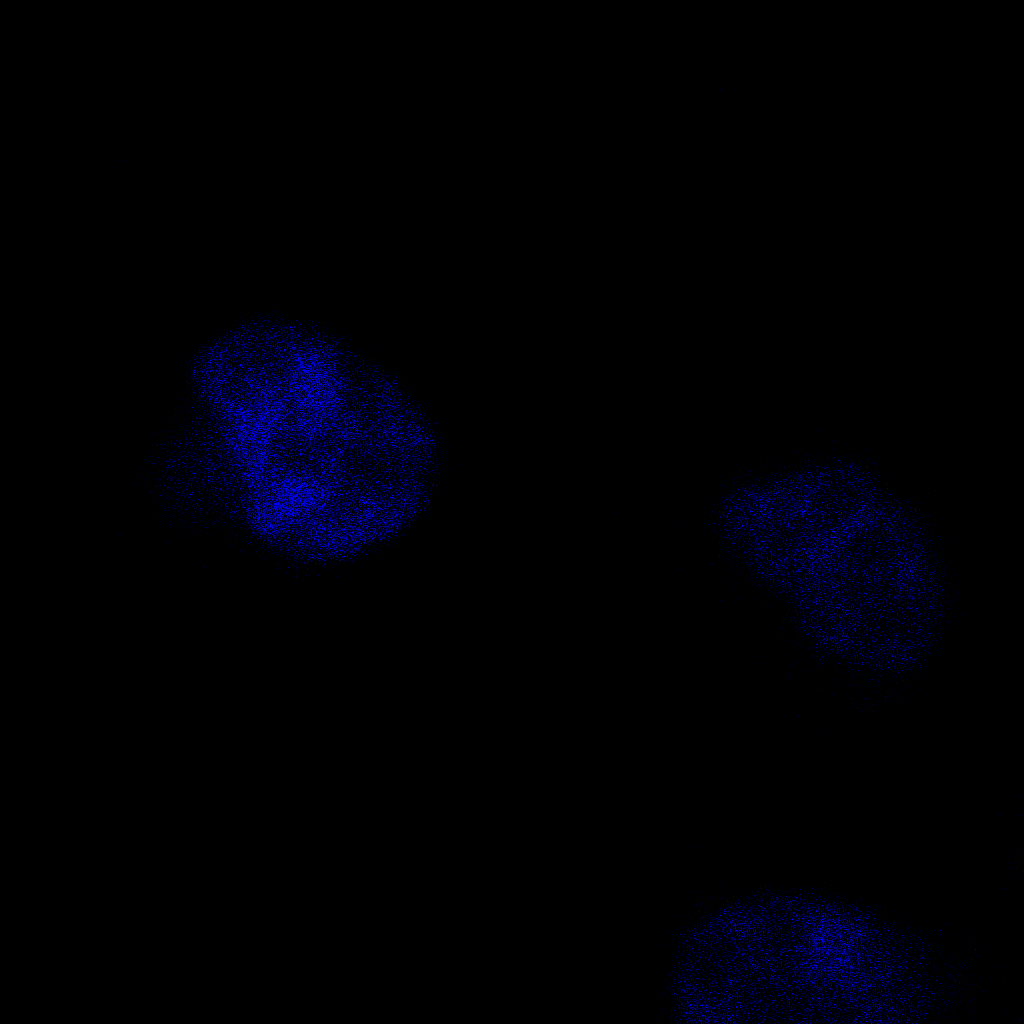

Supplement: S2 Data — (ZIP) [file ppat.1012546.s006.zip › Figure 5D/2/Flag-NLRP3/DAPI.tif]

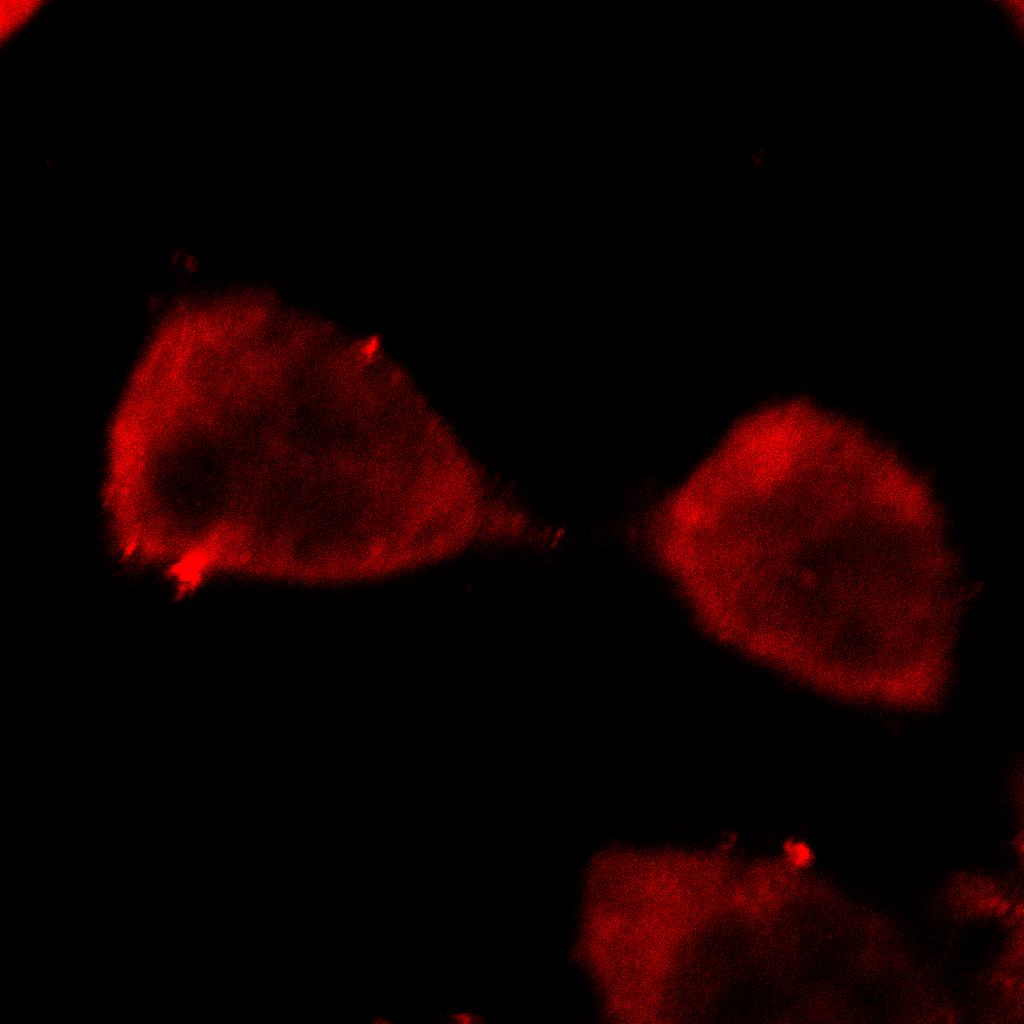

Supplement: S2 Data — (ZIP) [file ppat.1012546.s006.zip › Figure 5D/2/Flag-NLRP3/Flag-NLRP3 .tif]

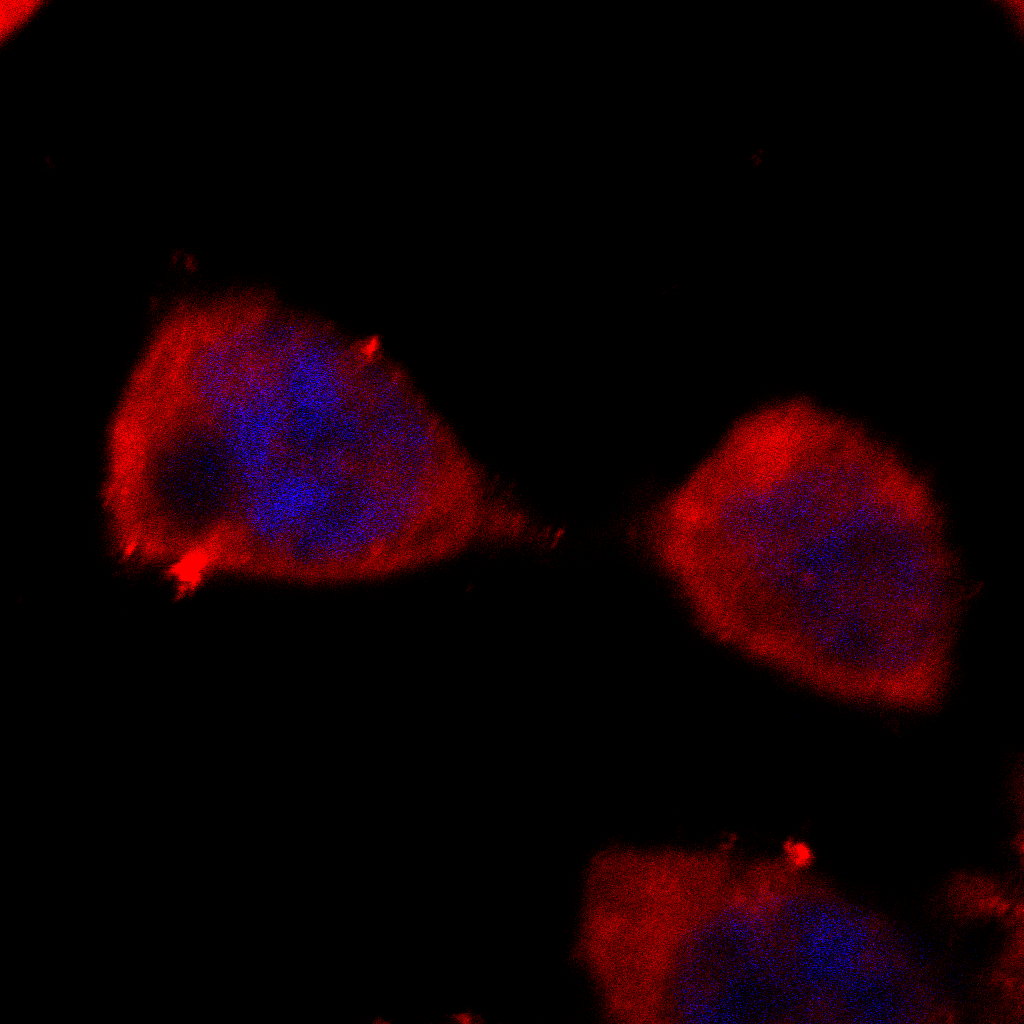

Supplement: S2 Data — (ZIP) [file ppat.1012546.s006.zip › Figure 5D/2/Flag-NLRP3/Merge.tif]

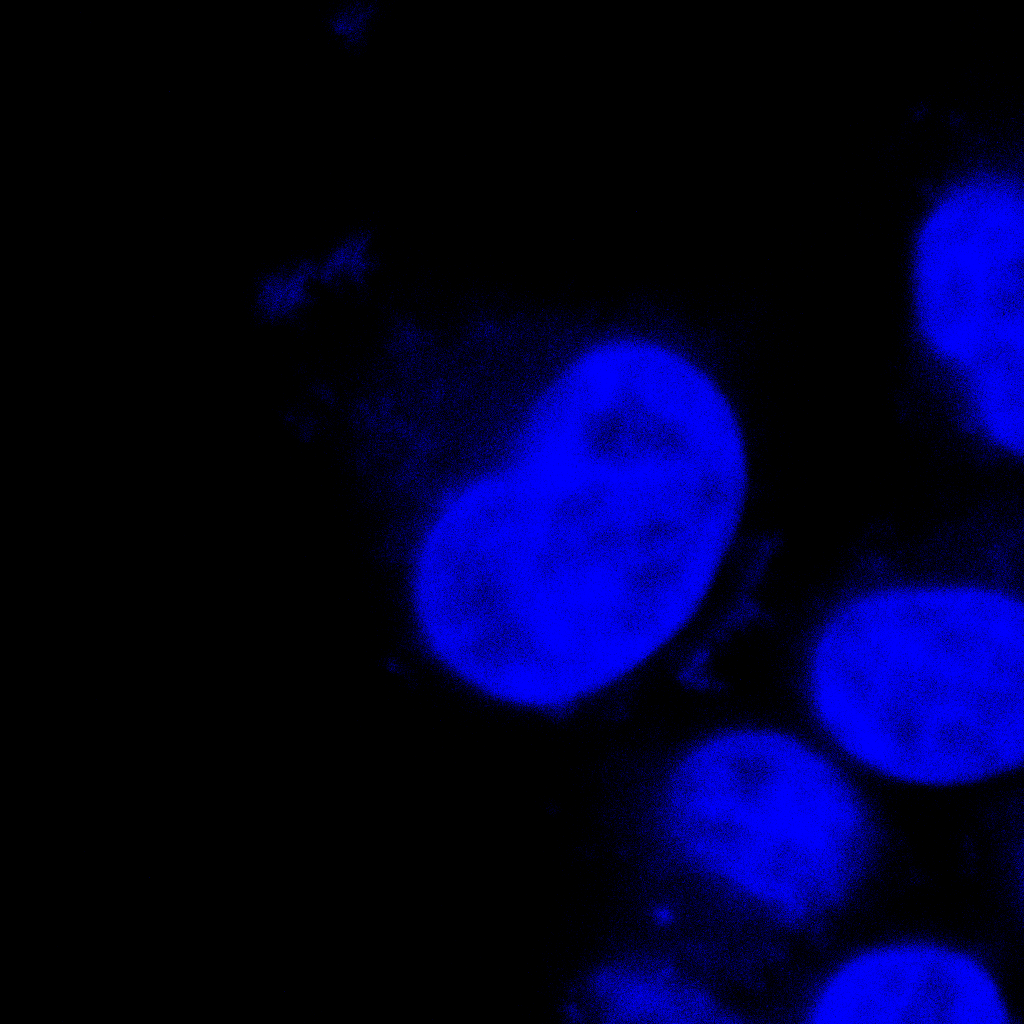

Supplement: S2 Data — (ZIP) [file ppat.1012546.s006.zip › Figure 5D/2/Flag-NLRP3+GFP-UL4/DAPI.tif]

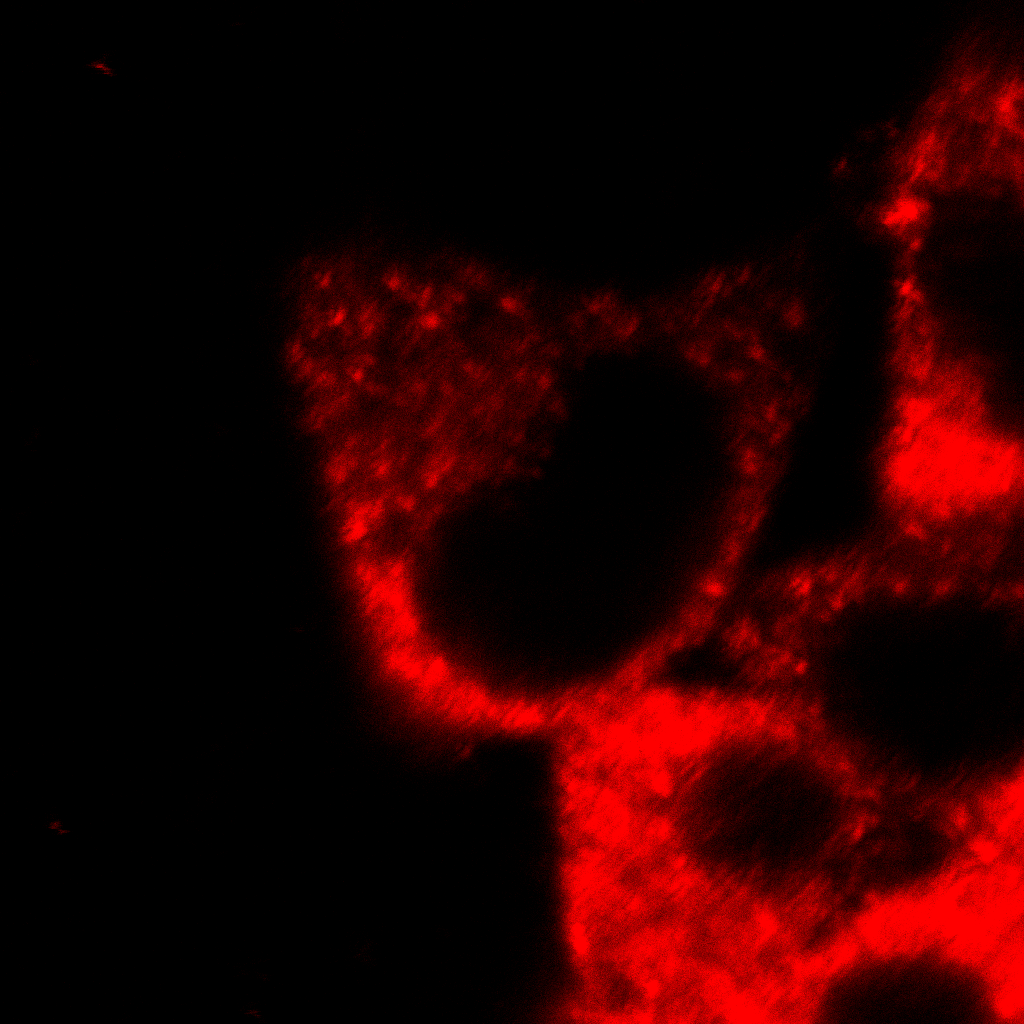

Supplement: S2 Data — (ZIP) [file ppat.1012546.s006.zip › Figure 5D/2/Flag-NLRP3+GFP-UL4/Flag-NLRP3.tif]

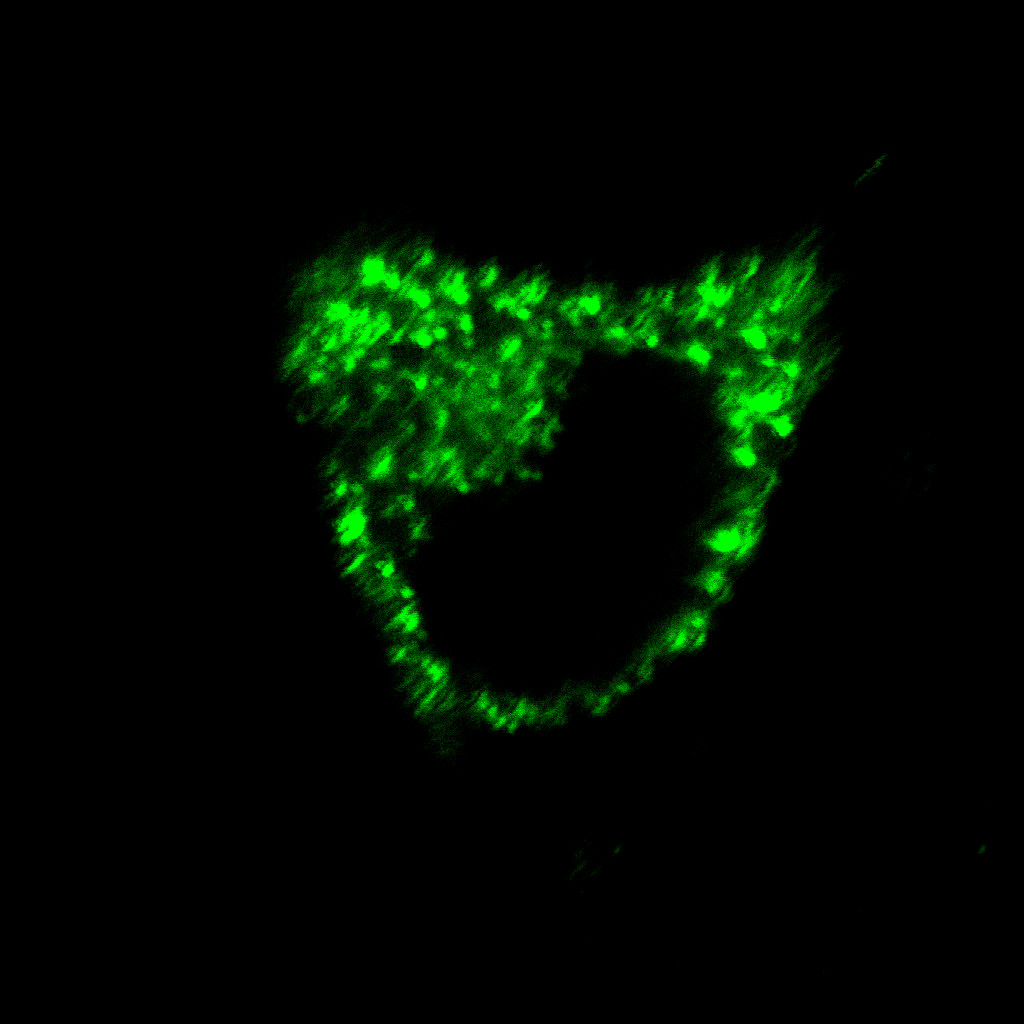

Supplement: S2 Data — (ZIP) [file ppat.1012546.s006.zip › Figure 5D/2/Flag-NLRP3+GFP-UL4/GFP-UL4.tif]

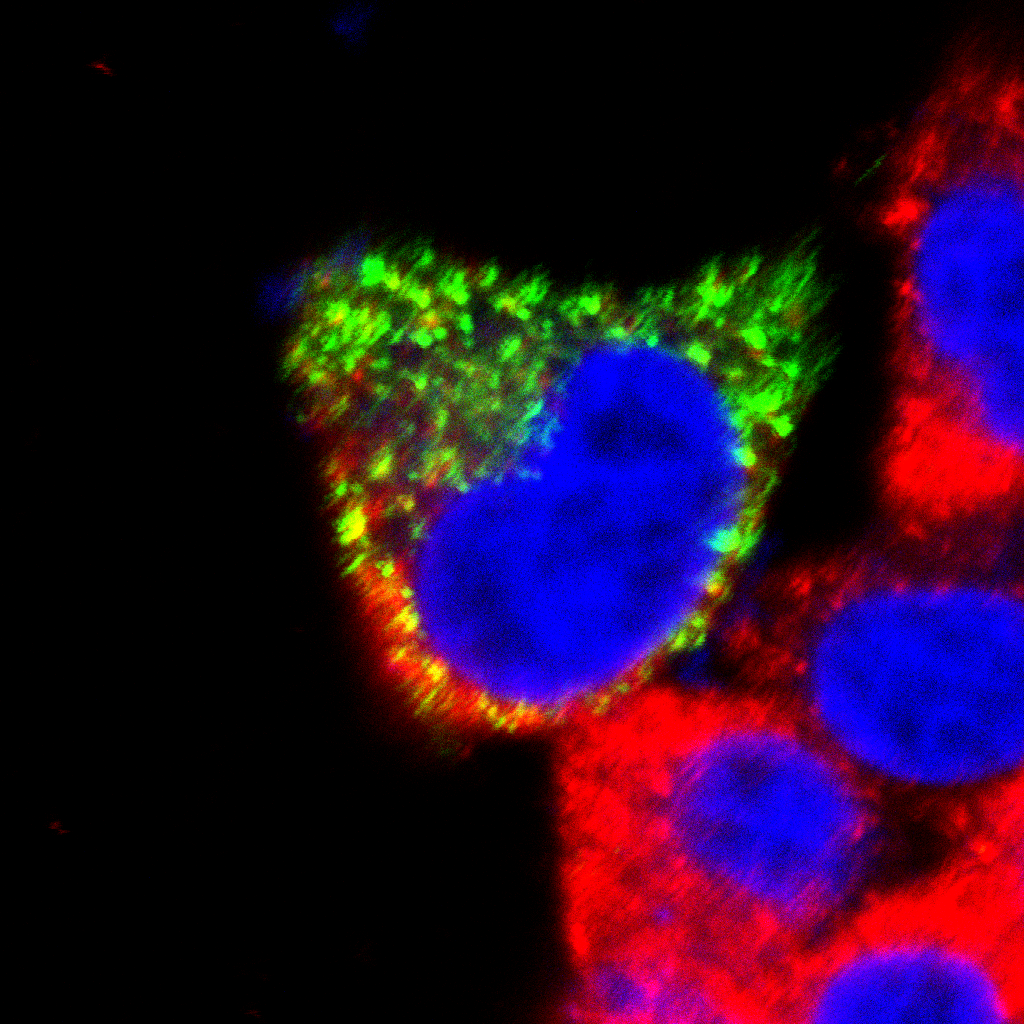

Supplement: S2 Data — (ZIP) [file ppat.1012546.s006.zip › Figure 5D/2/Flag-NLRP3+GFP-UL4/Merge.tif]

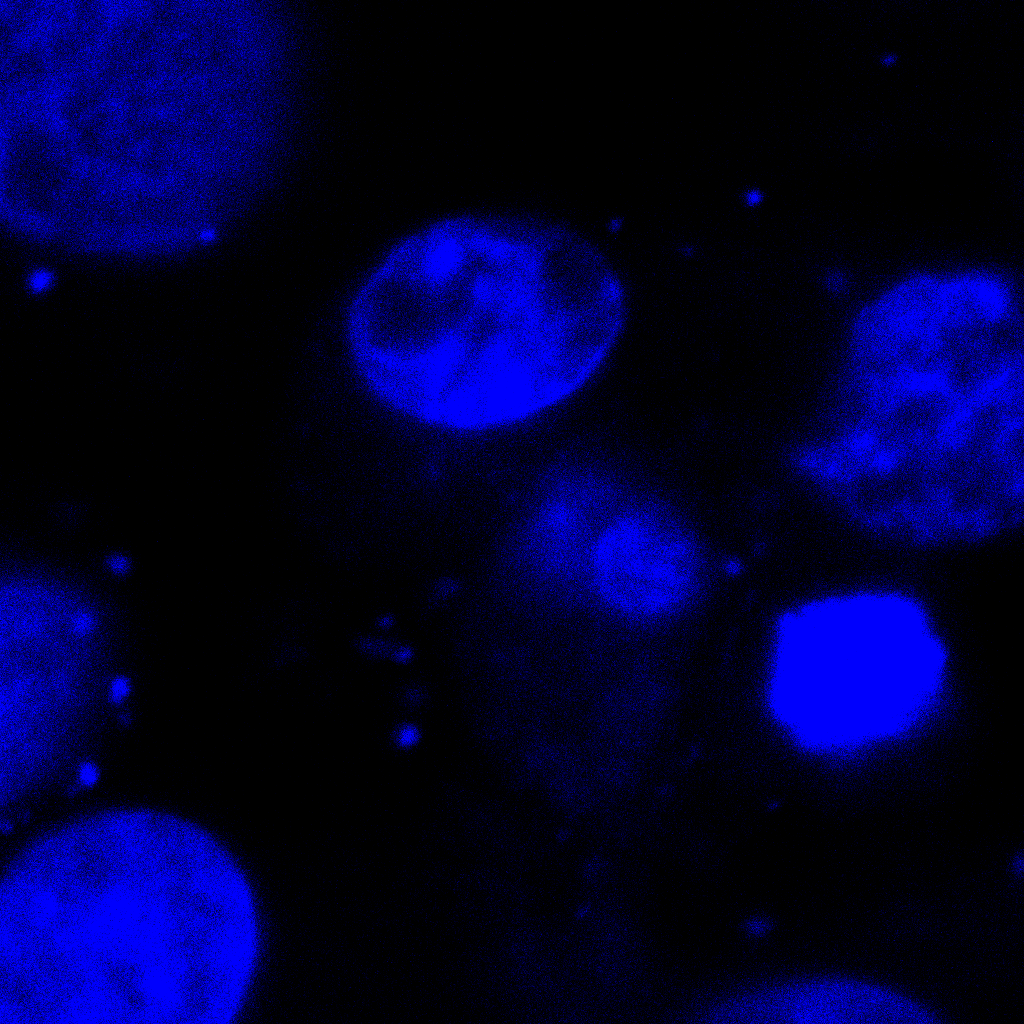

Supplement: S2 Data — (ZIP) [file ppat.1012546.s006.zip › Figure 5D/2/GFP-UL4/DAPI.tif]

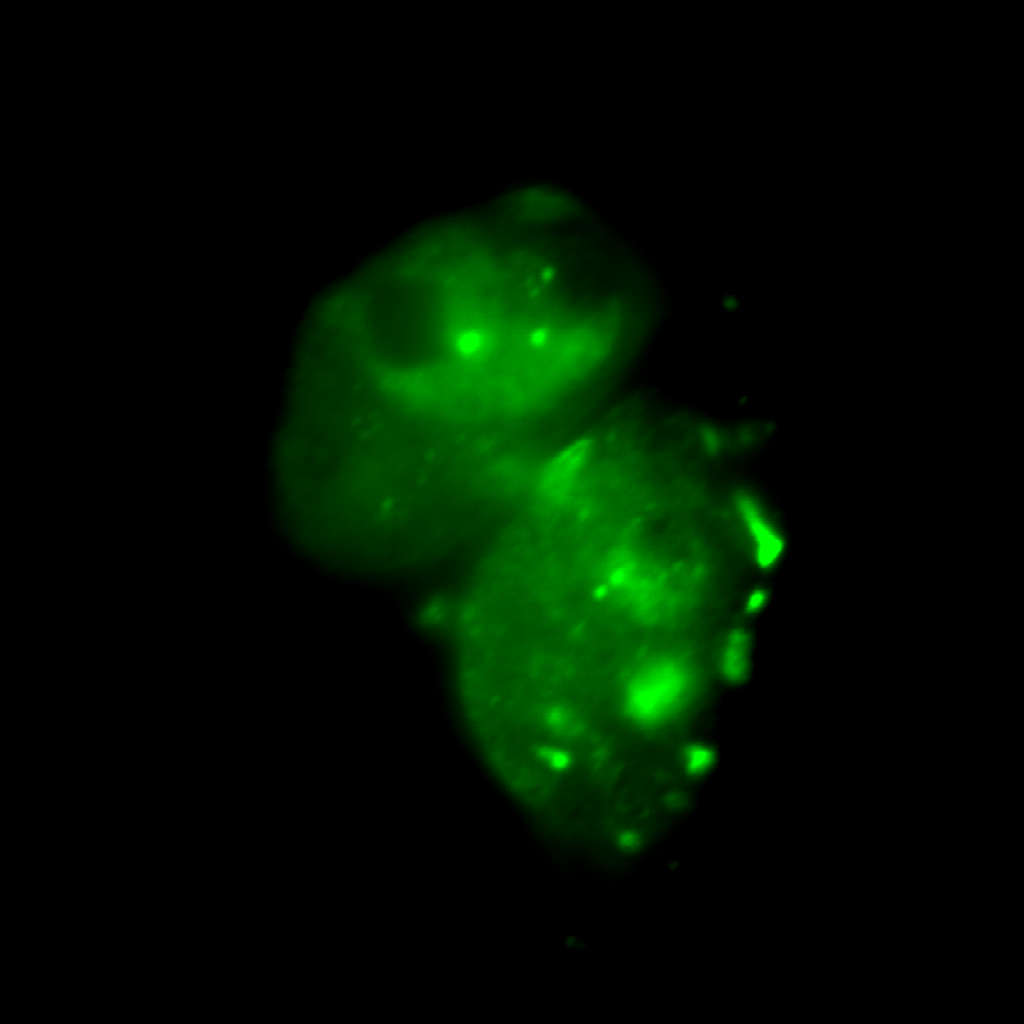

Supplement: S2 Data — (ZIP) [file ppat.1012546.s006.zip › Figure 5D/2/GFP-UL4/GFP-UL4.tif]

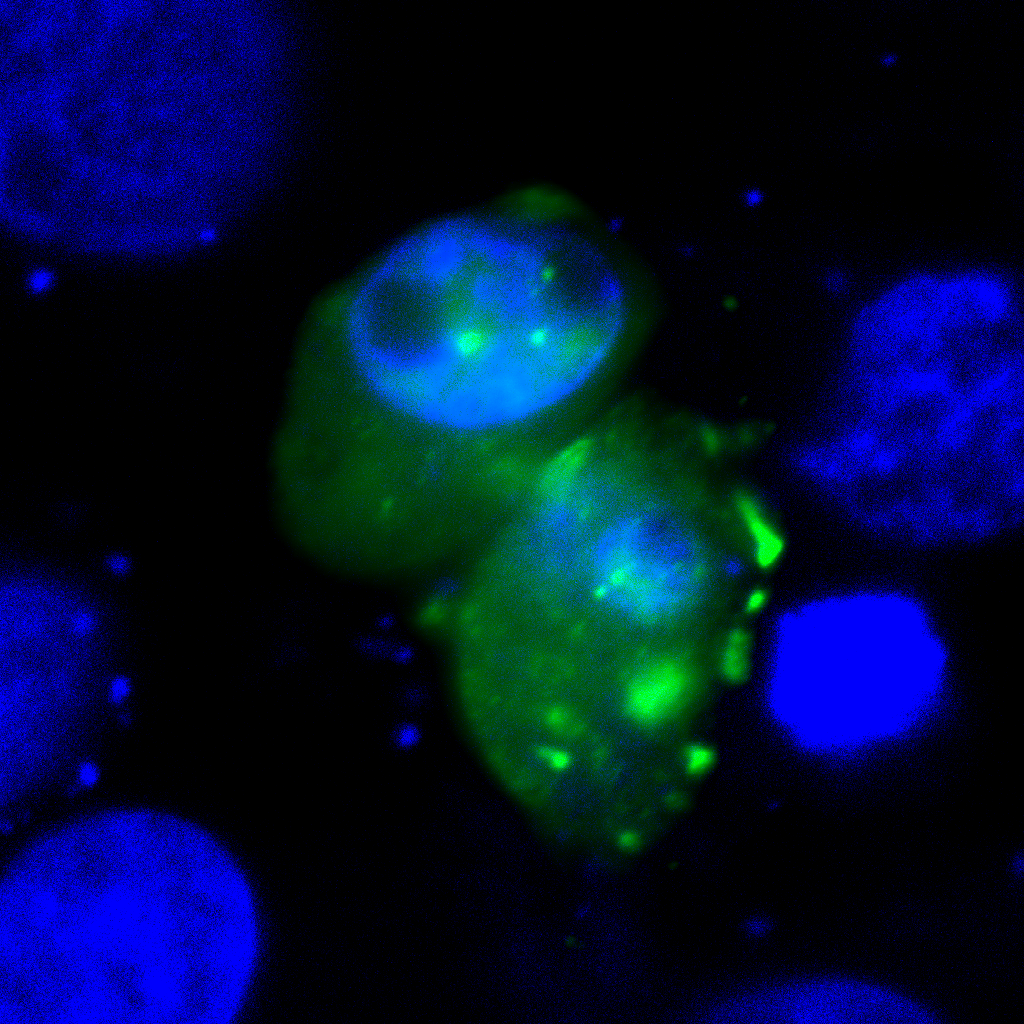

Supplement: S2 Data — (ZIP) [file ppat.1012546.s006.zip › Figure 5D/2/GFP-UL4/Merge.tif]

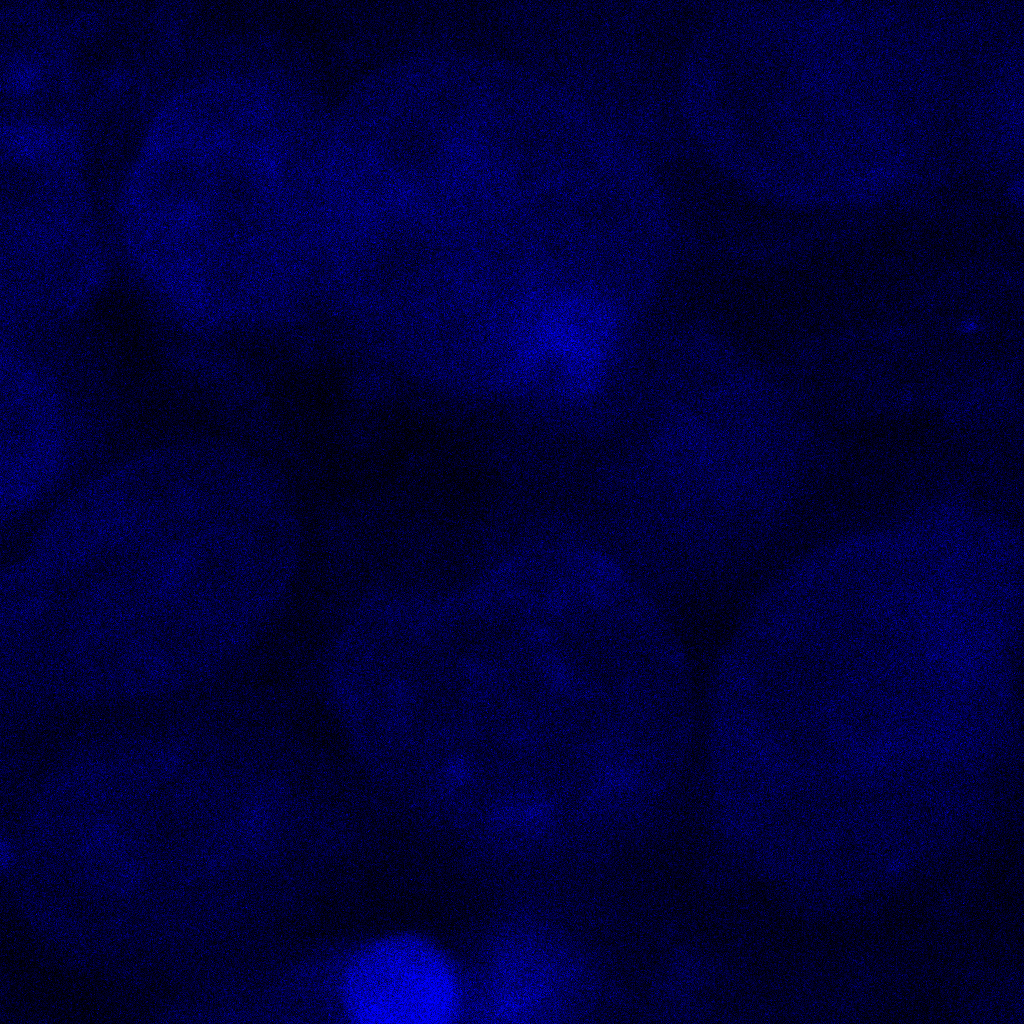

Supplement: S2 Data — (ZIP) [file ppat.1012546.s006.zip › Figure 5D/3/Flag-ASC/DAPI-1.tif]

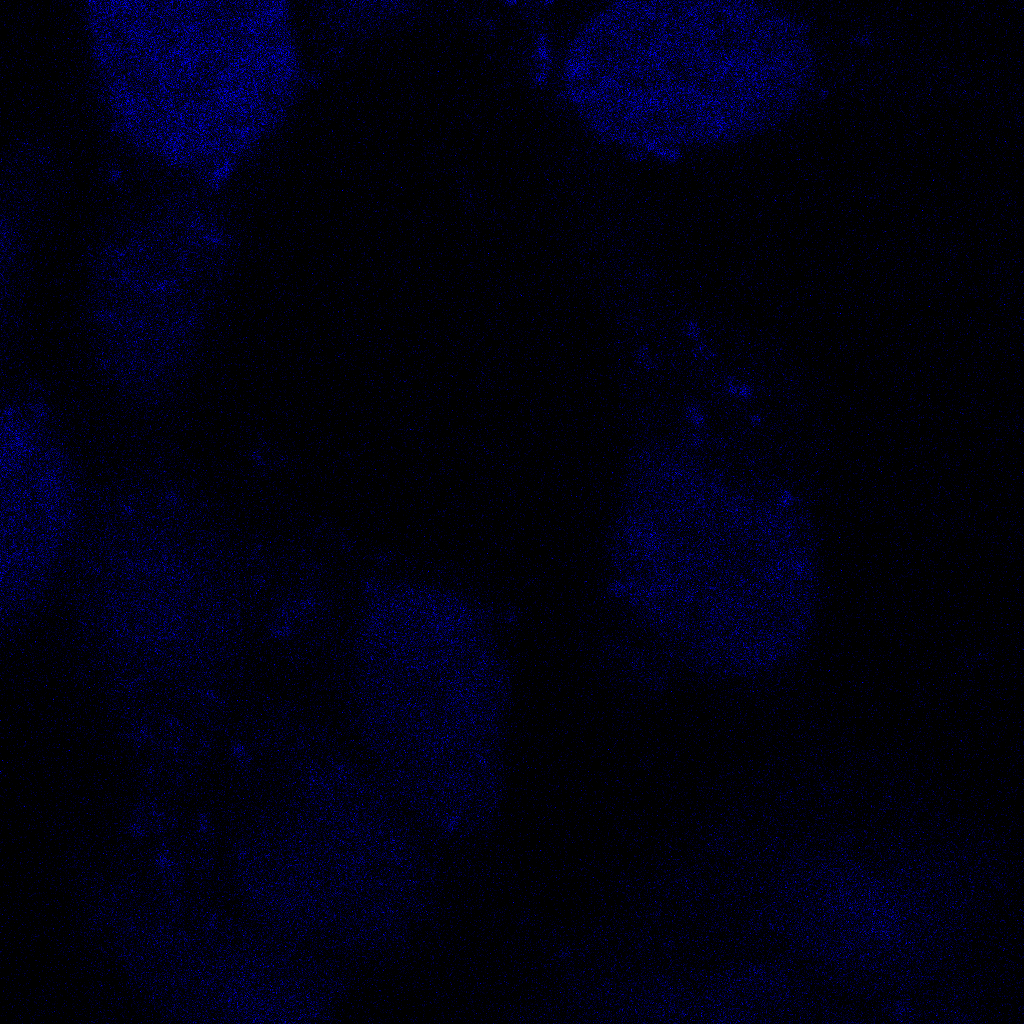

Supplement: S2 Data — (ZIP) [file ppat.1012546.s006.zip › Figure 5D/3/Flag-ASC/DAPI-2.tif]

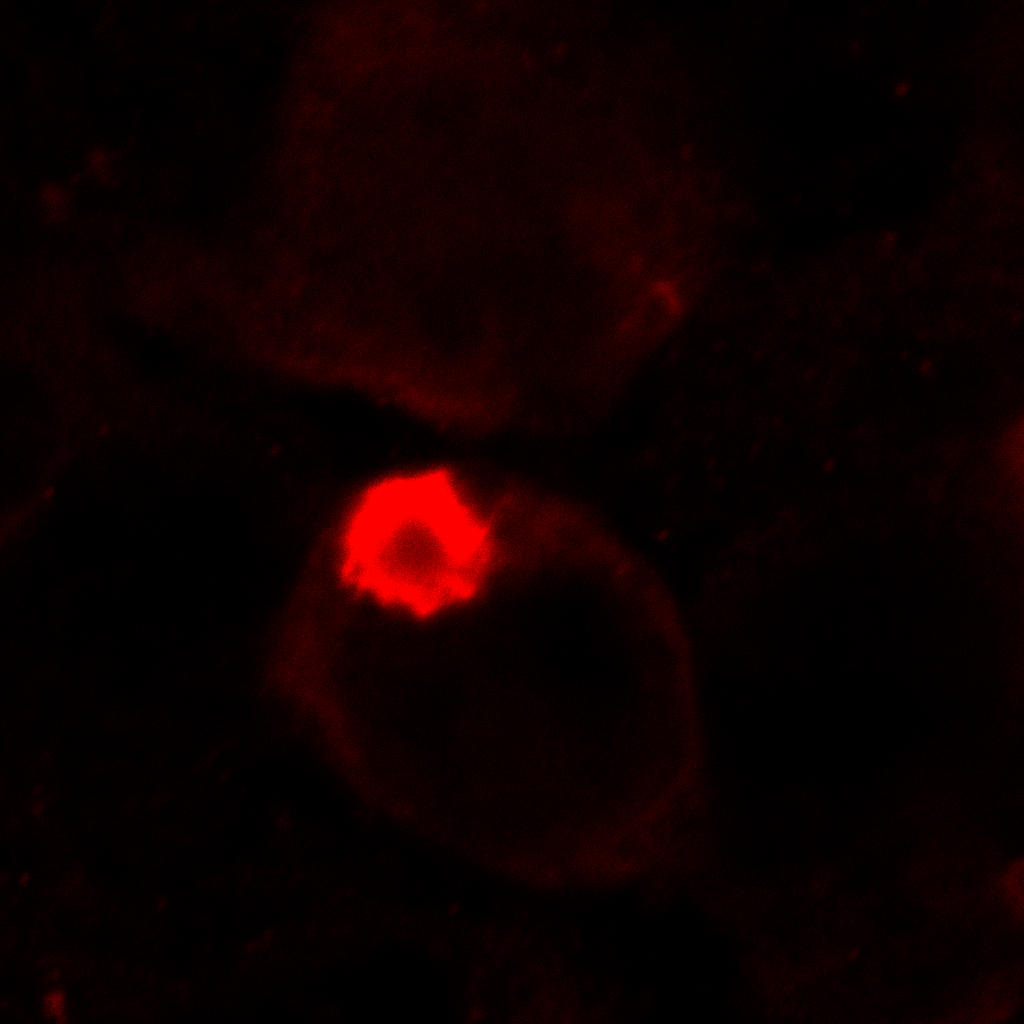

Supplement: S2 Data — (ZIP) [file ppat.1012546.s006.zip › Figure 5D/3/Flag-ASC/flag-asc-1.tif]

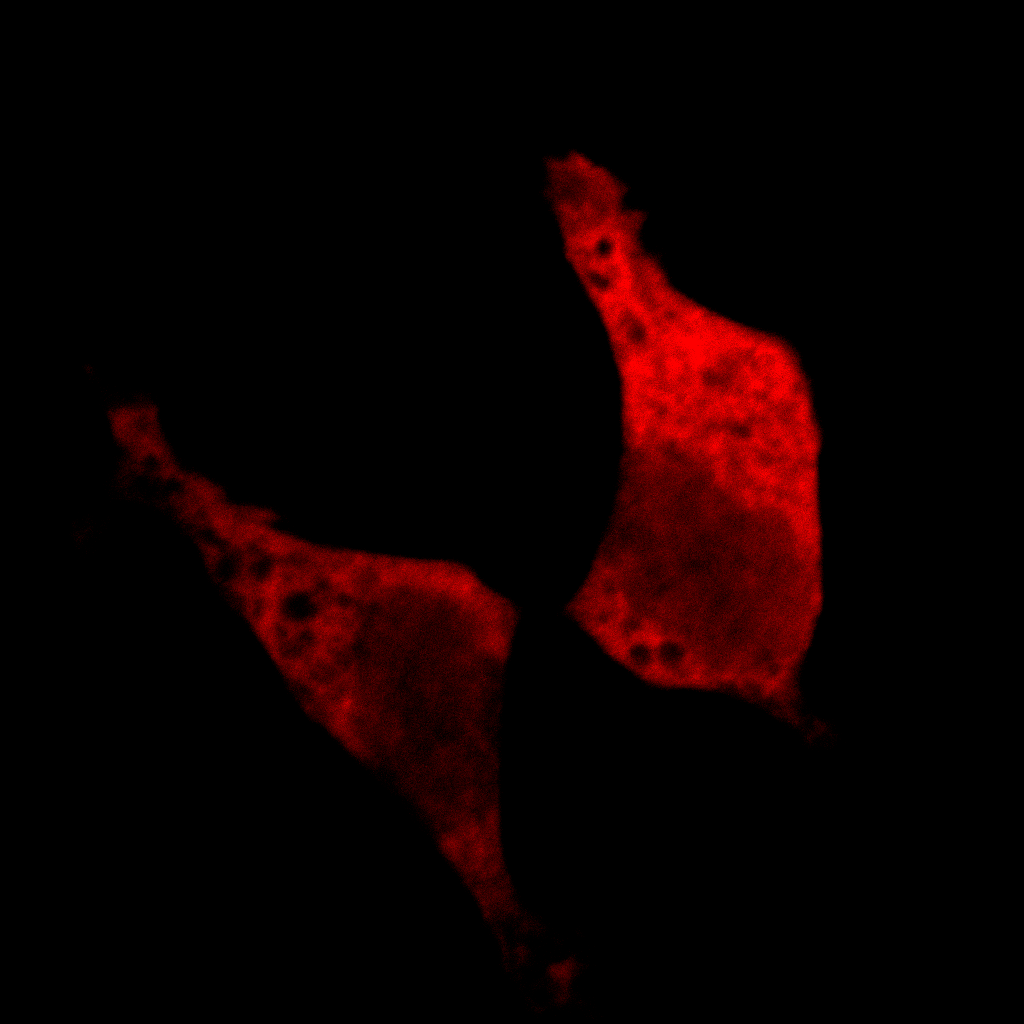

Supplement: S2 Data — (ZIP) [file ppat.1012546.s006.zip › Figure 5D/3/Flag-ASC/flag-asc-2.tif]

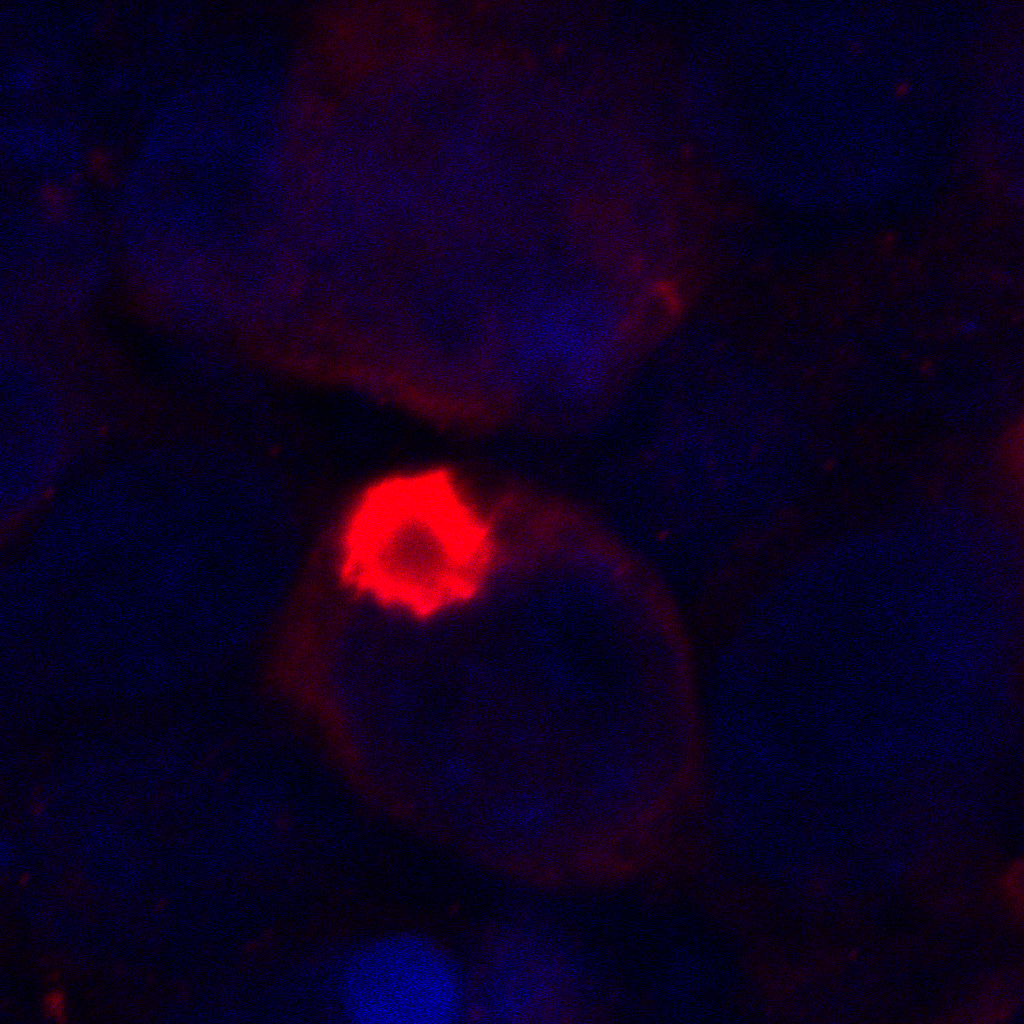

Supplement: S2 Data — (ZIP) [file ppat.1012546.s006.zip › Figure 5D/3/Flag-ASC/Merge.tif]

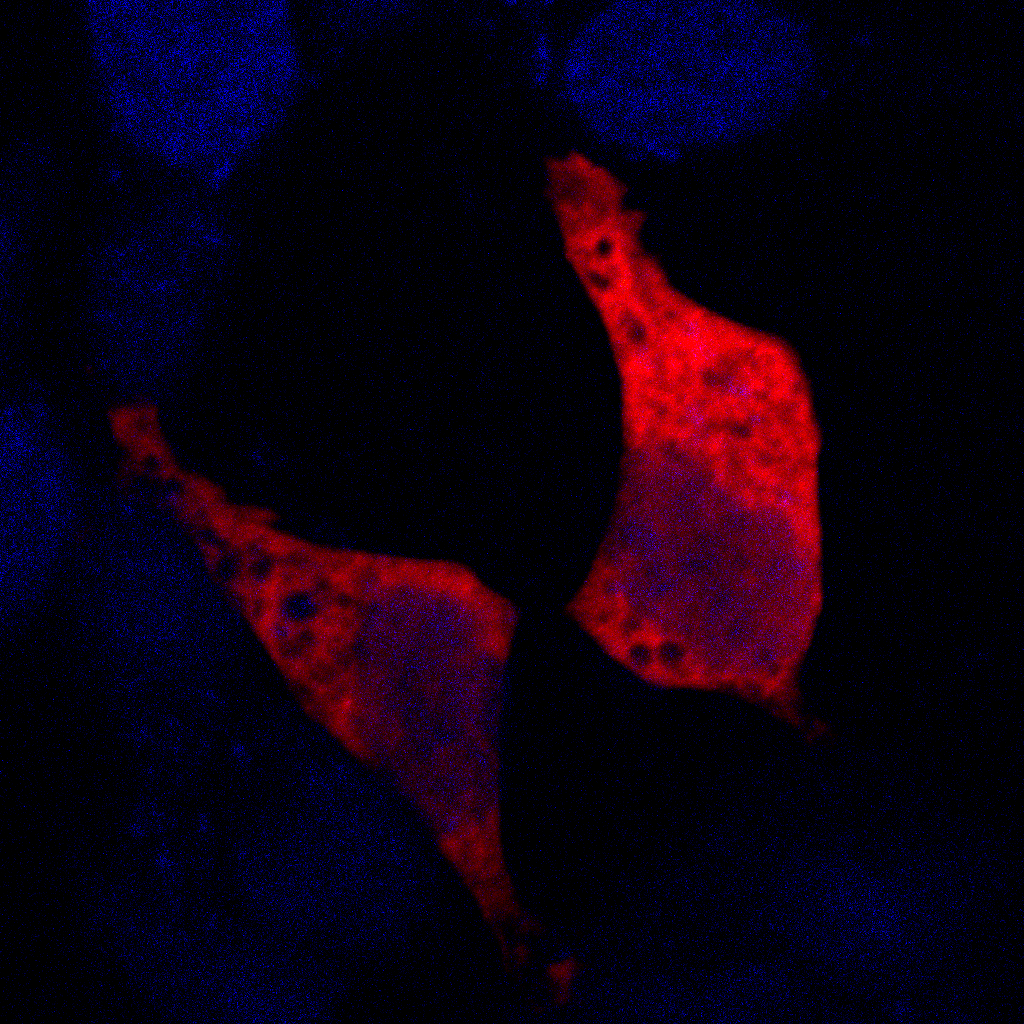

Supplement: S2 Data — (ZIP) [file ppat.1012546.s006.zip › Figure 5D/3/Flag-ASC/Merge-2.tif]

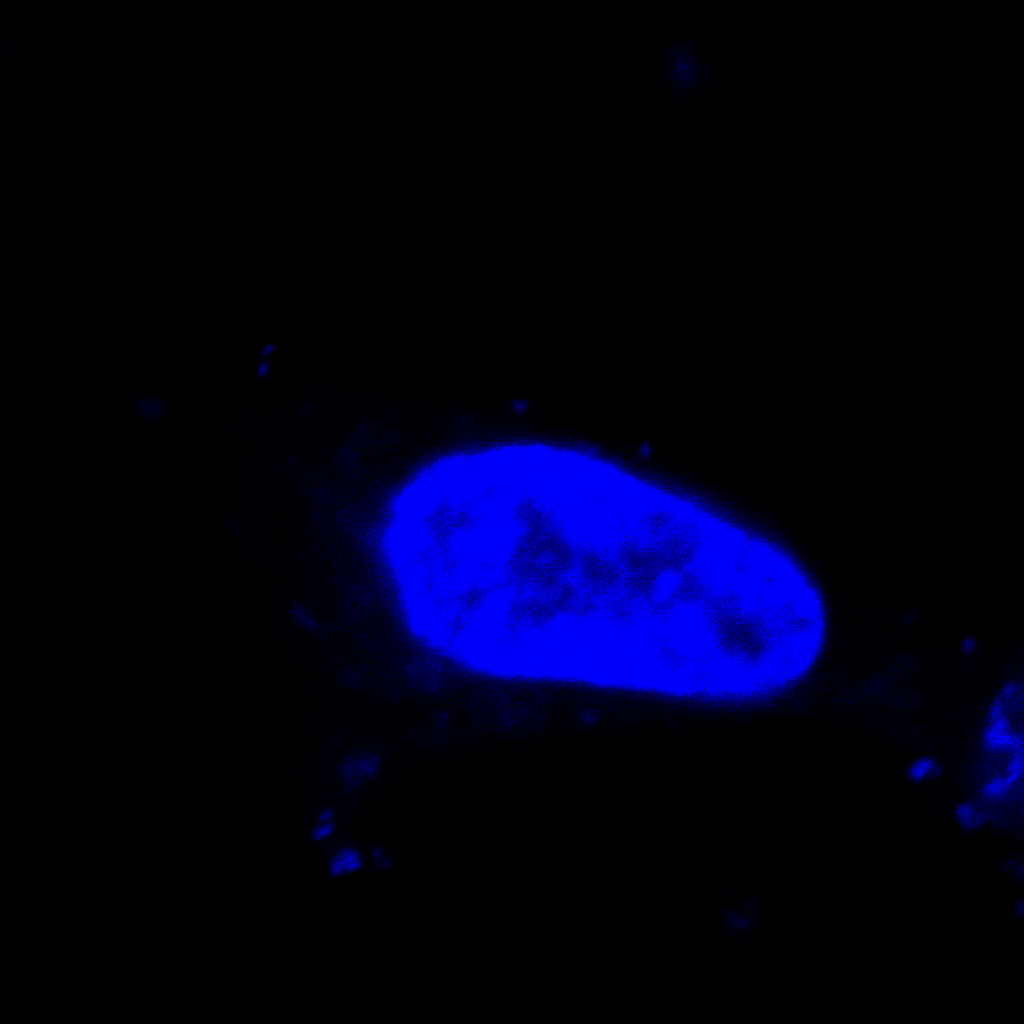

Supplement: S2 Data — (ZIP) [file ppat.1012546.s006.zip › Figure 5D/3/Flag-ASC+GFP-UL4/1/DAPI.tif]

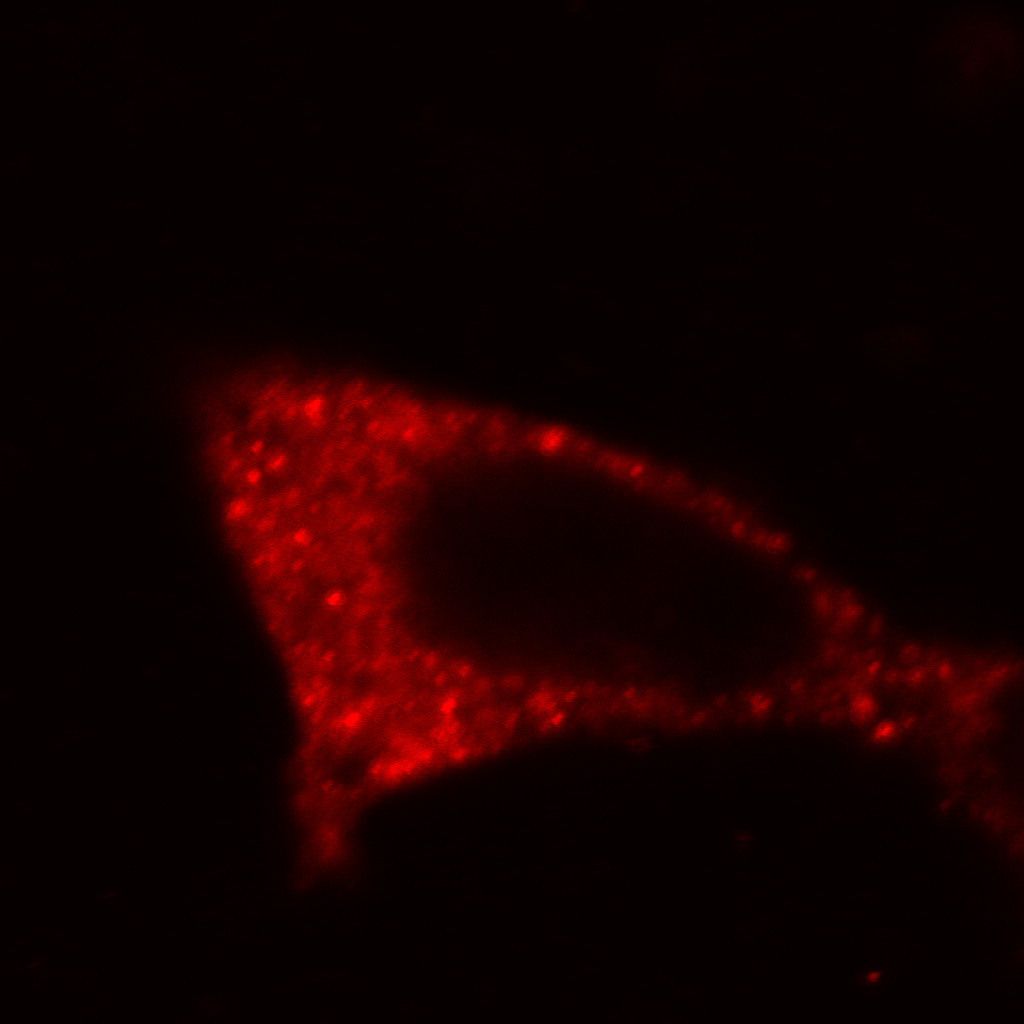

Supplement: S2 Data — (ZIP) [file ppat.1012546.s006.zip › Figure 5D/3/Flag-ASC+GFP-UL4/1/flag-asc.tif]

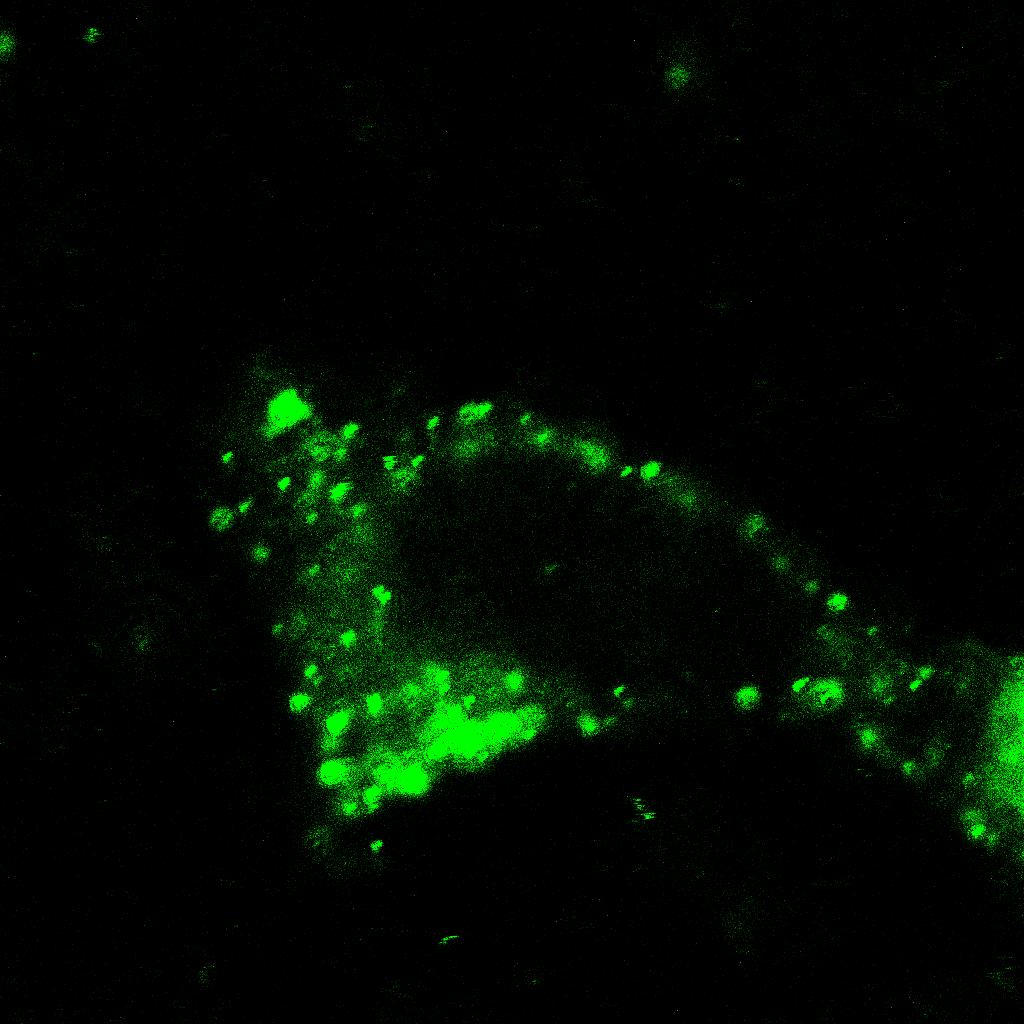

Supplement: S2 Data — (ZIP) [file ppat.1012546.s006.zip › Figure 5D/3/Flag-ASC+GFP-UL4/1/gfp-ul4.tif]

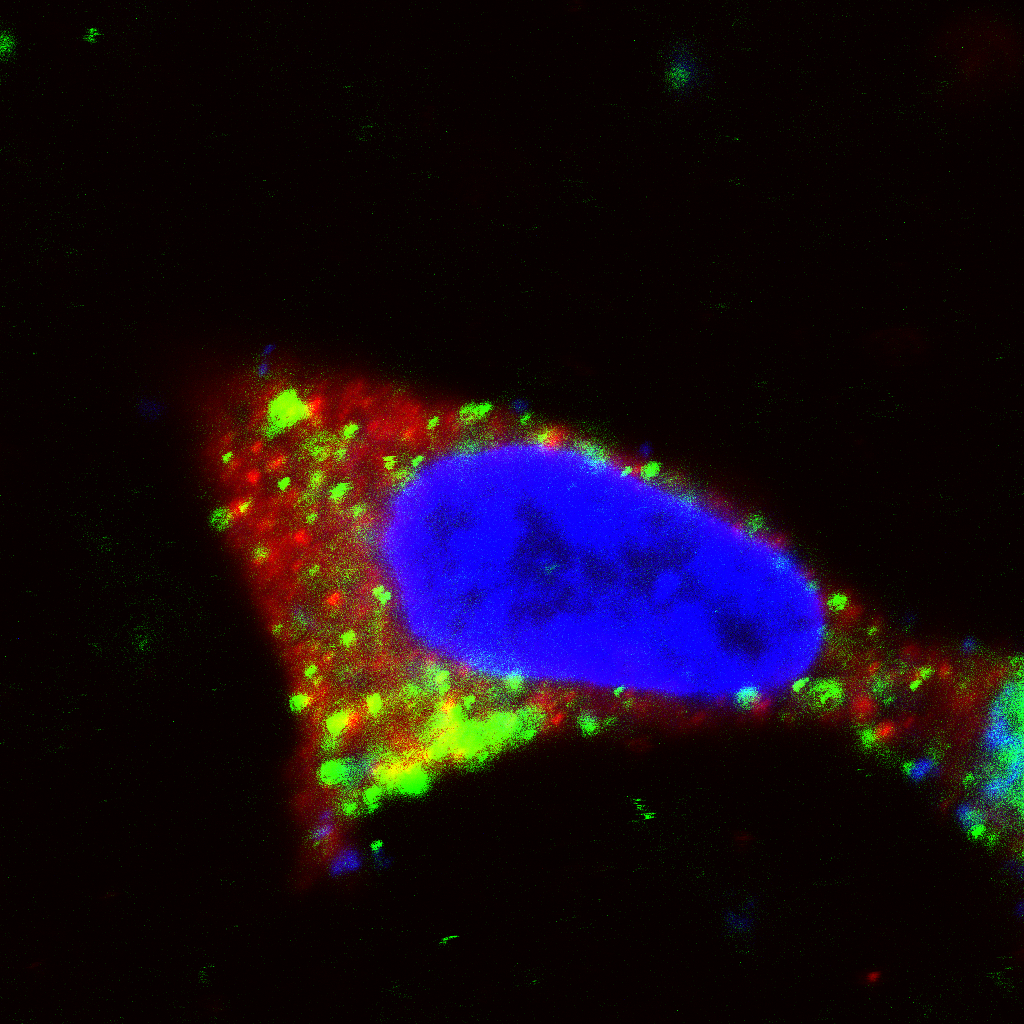

Supplement: S2 Data — (ZIP) [file ppat.1012546.s006.zip › Figure 5D/3/Flag-ASC+GFP-UL4/1/Merge.tif]

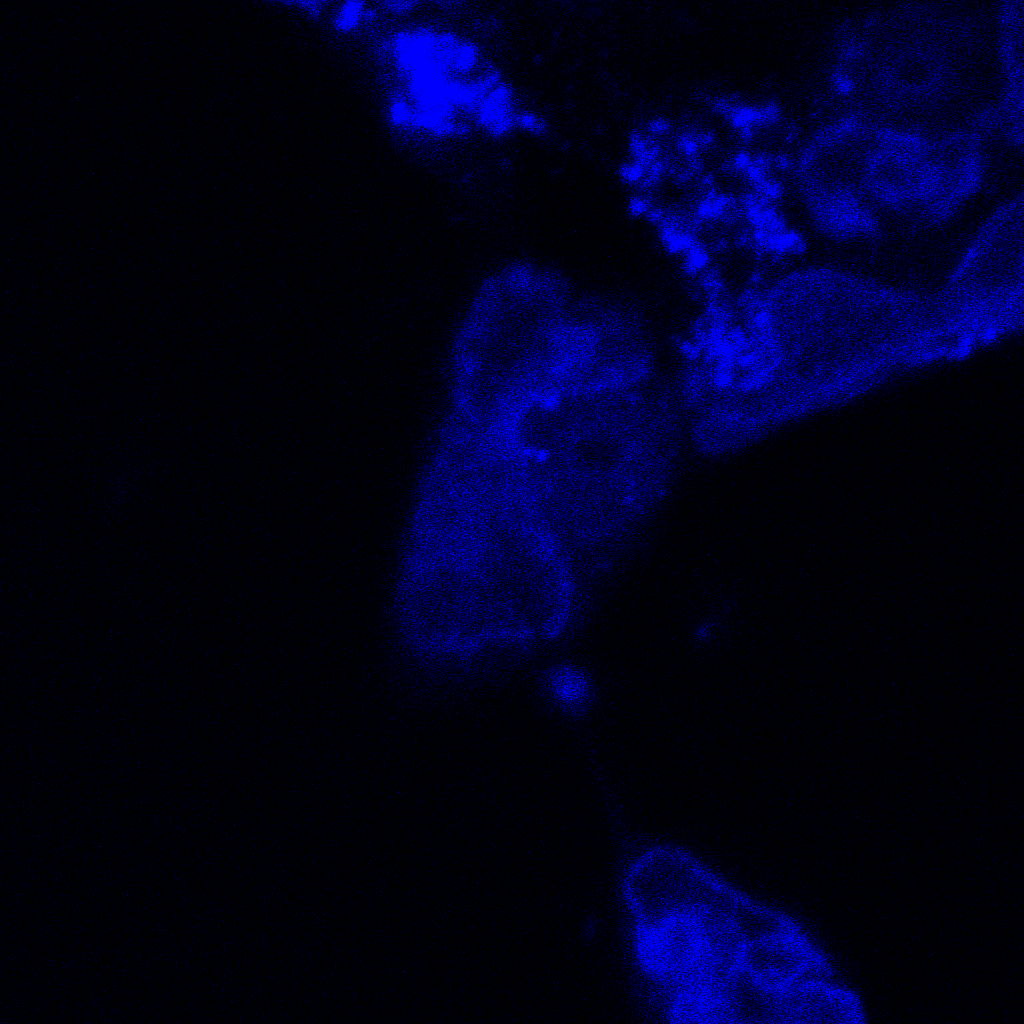

Supplement: S2 Data — (ZIP) [file ppat.1012546.s006.zip › Figure 5D/3/Flag-ASC+GFP-UL4/2/DAPI.tif]

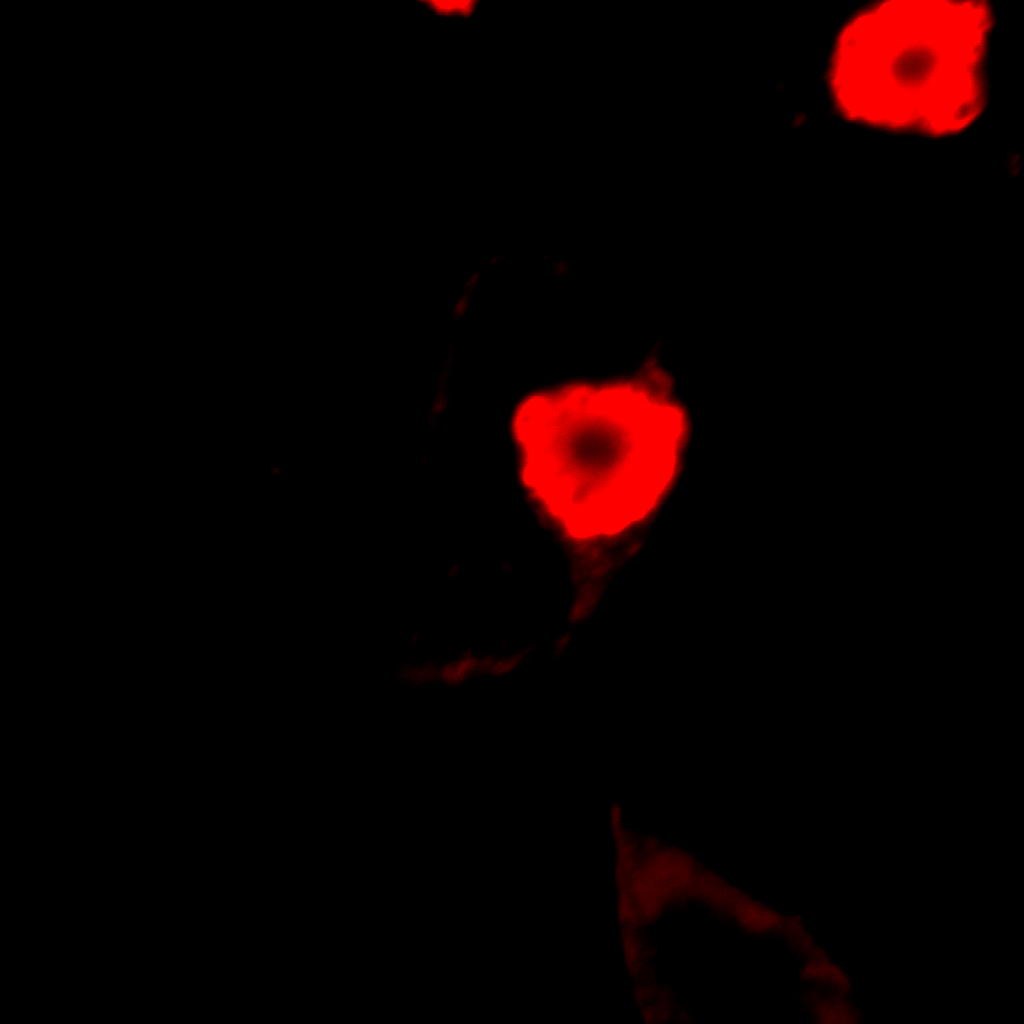

Supplement: S2 Data — (ZIP) [file ppat.1012546.s006.zip › Figure 5D/3/Flag-ASC+GFP-UL4/2/Flag-ASC.tif]

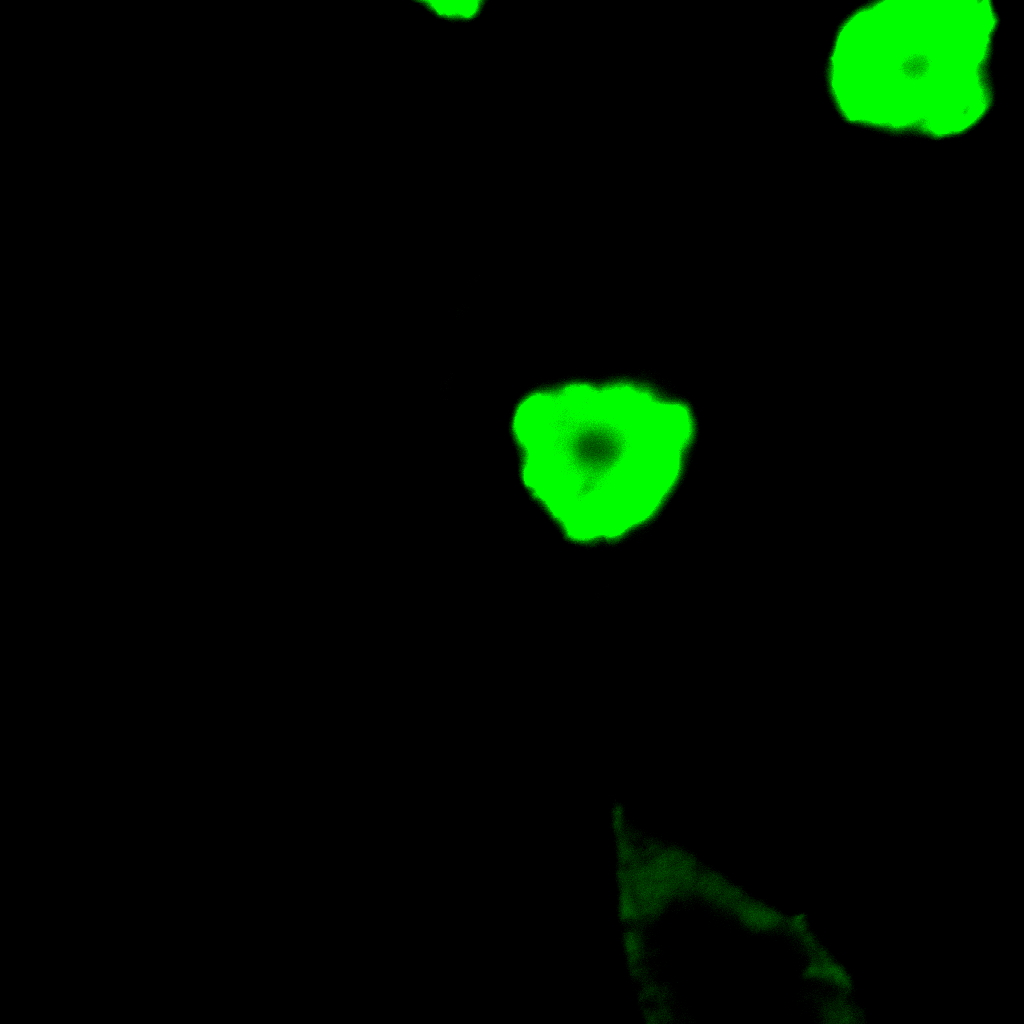

Supplement: S2 Data — (ZIP) [file ppat.1012546.s006.zip › Figure 5D/3/Flag-ASC+GFP-UL4/2/GFP-UL4.tif]

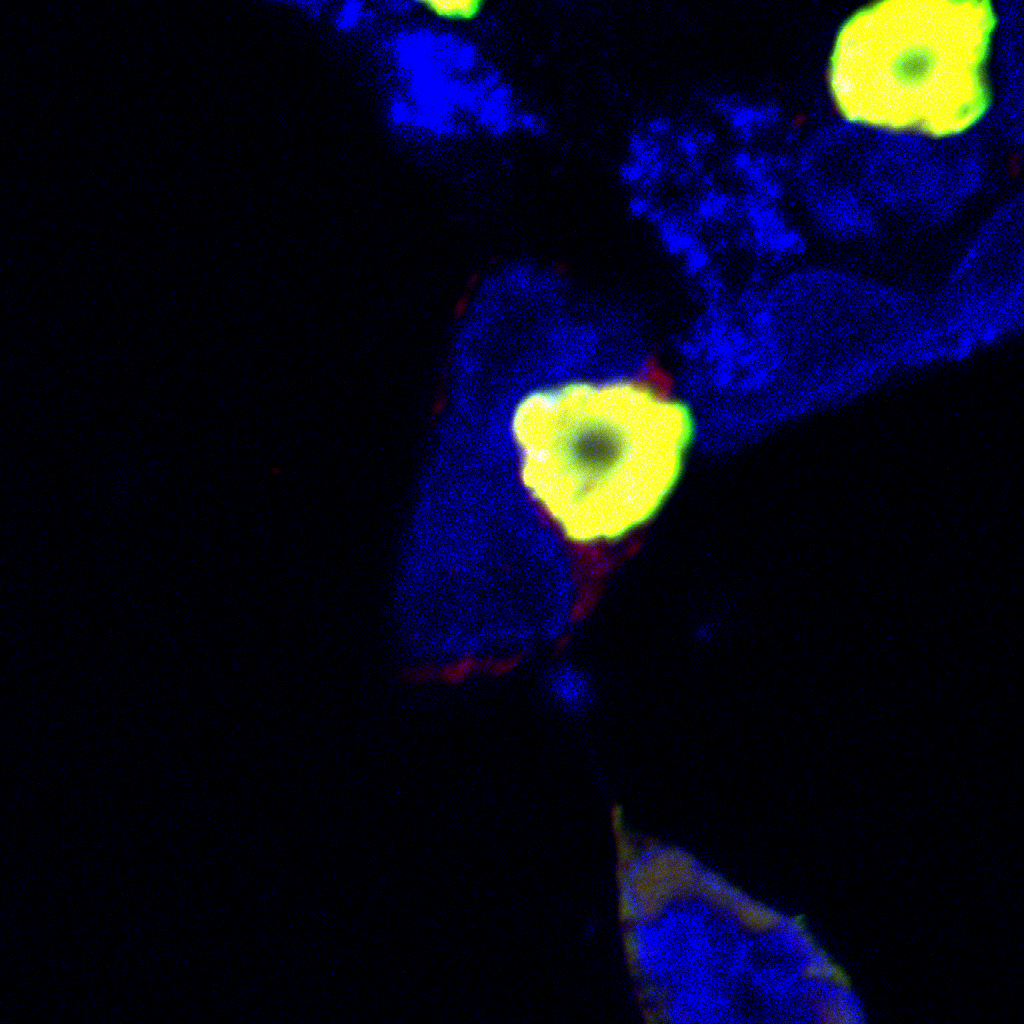

Supplement: S2 Data — (ZIP) [file ppat.1012546.s006.zip › Figure 5D/3/Flag-ASC+GFP-UL4/2/Merge.tif]

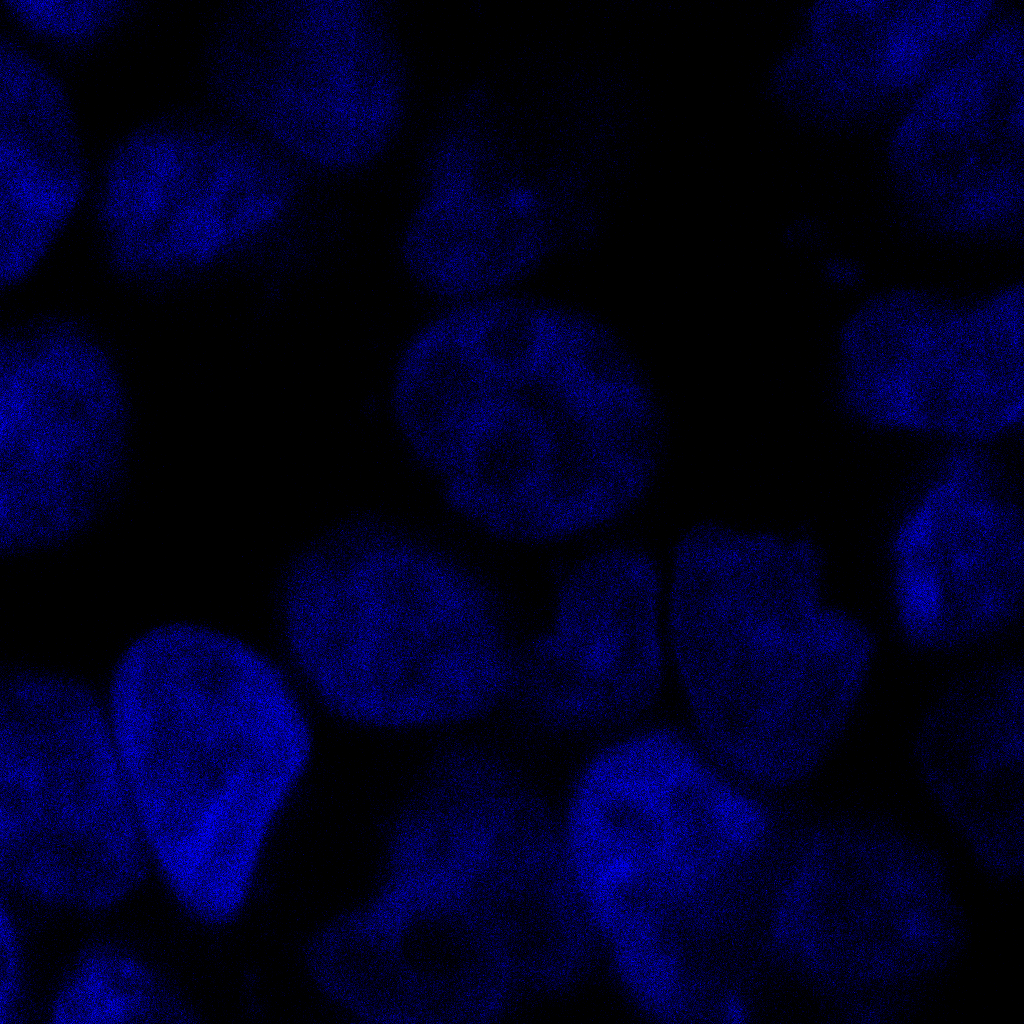

Supplement: S2 Data — (ZIP) [file ppat.1012546.s006.zip › Figure 5D/3/Flag-CASP1/DAPI.tif]

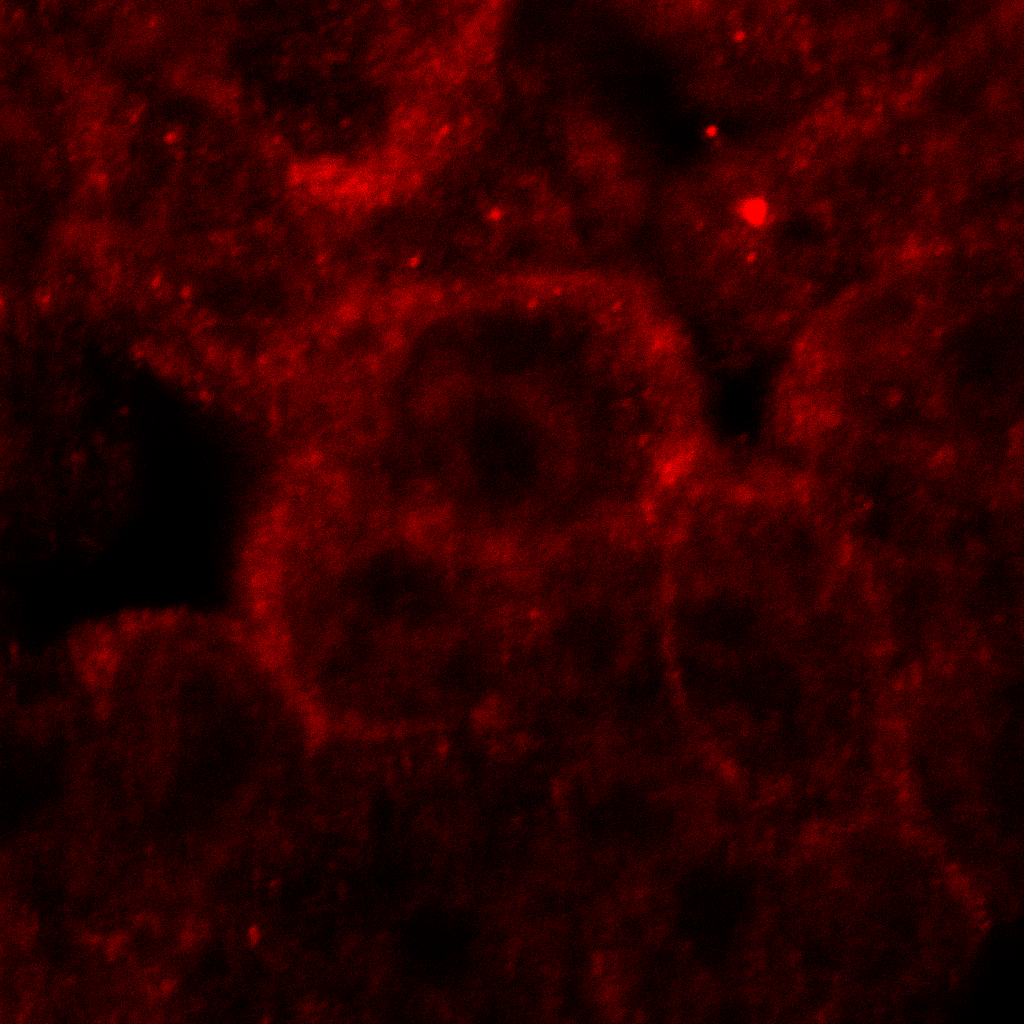

Supplement: S2 Data — (ZIP) [file ppat.1012546.s006.zip › Figure 5D/3/Flag-CASP1/flag-casp1.tif]

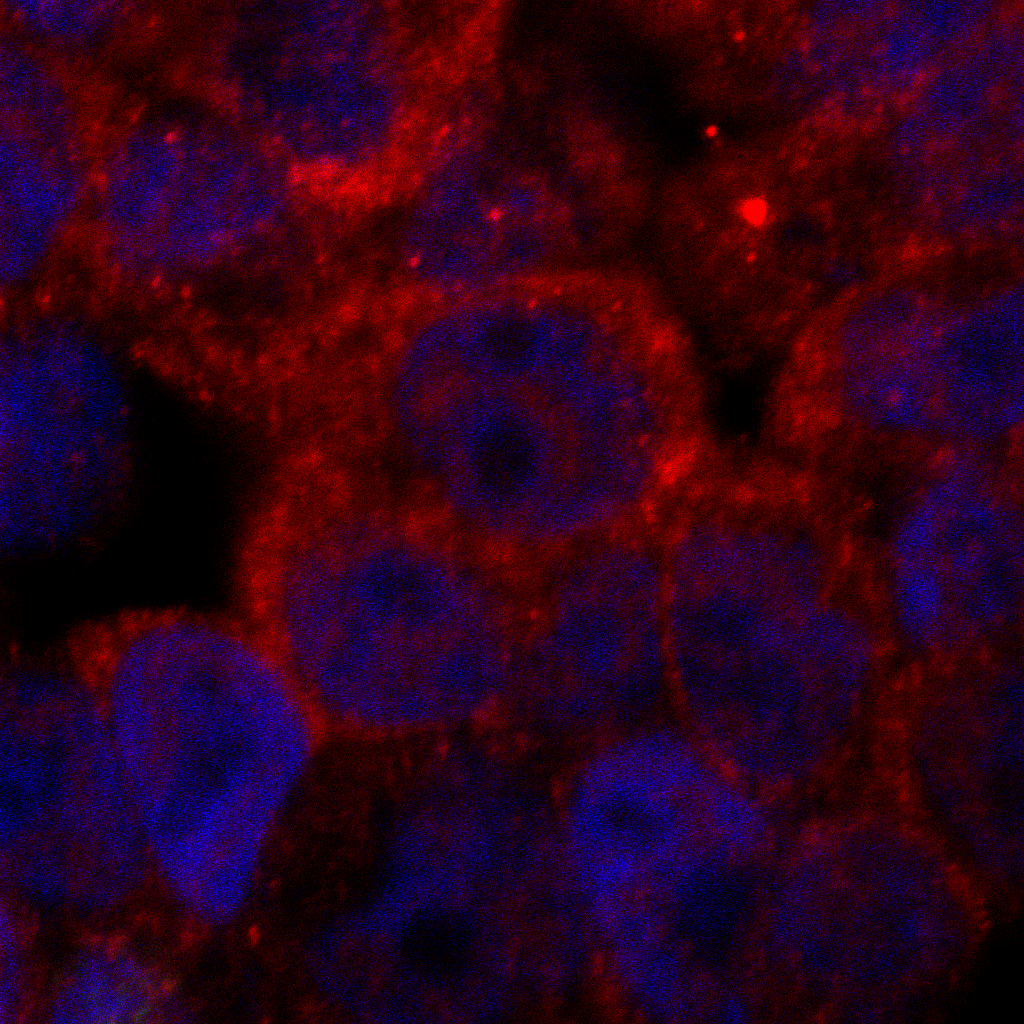

Supplement: S2 Data — (ZIP) [file ppat.1012546.s006.zip › Figure 5D/3/Flag-CASP1/Merge.tif]

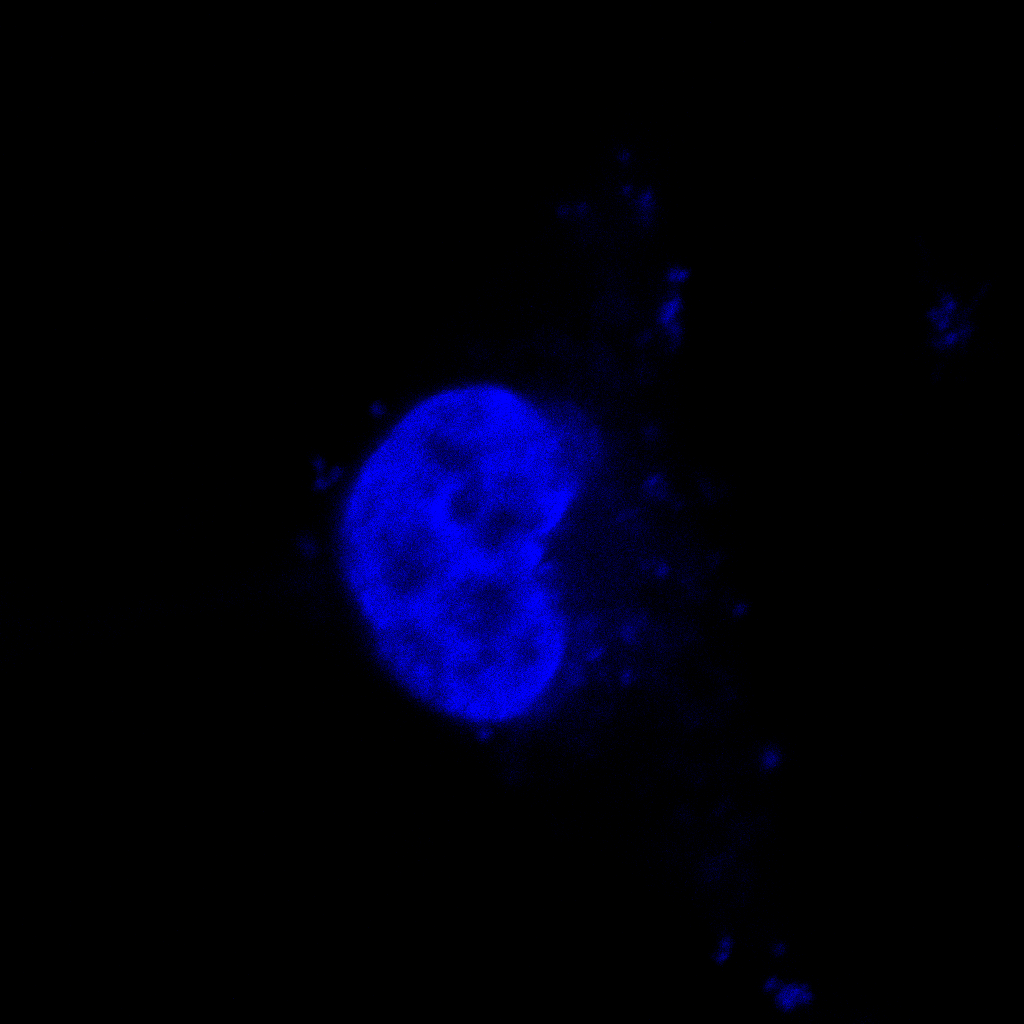

Supplement: S2 Data — (ZIP) [file ppat.1012546.s006.zip › Figure 5D/3/Flag-CASP1+GFP-UL4/DAPI.tif]

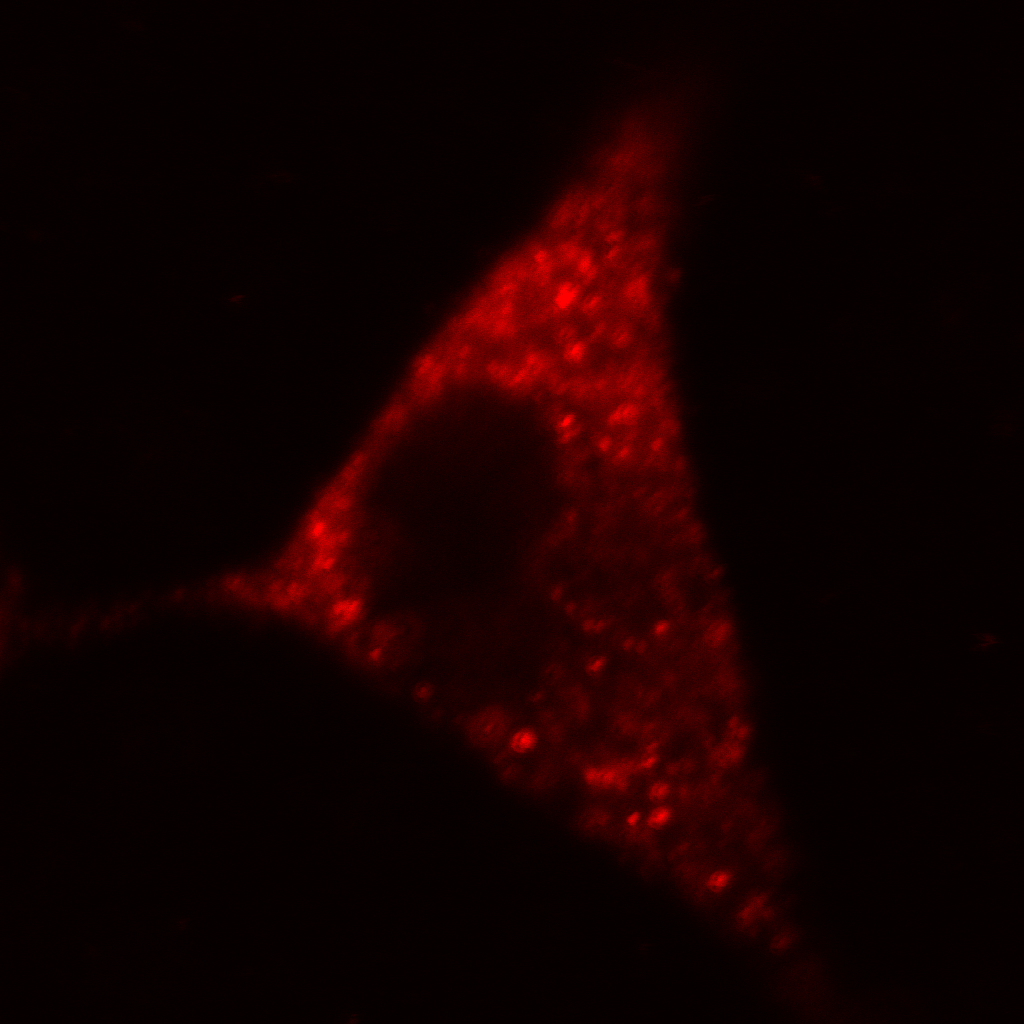

Supplement: S2 Data — (ZIP) [file ppat.1012546.s006.zip › Figure 5D/3/Flag-CASP1+GFP-UL4/flag-casp1.tif]

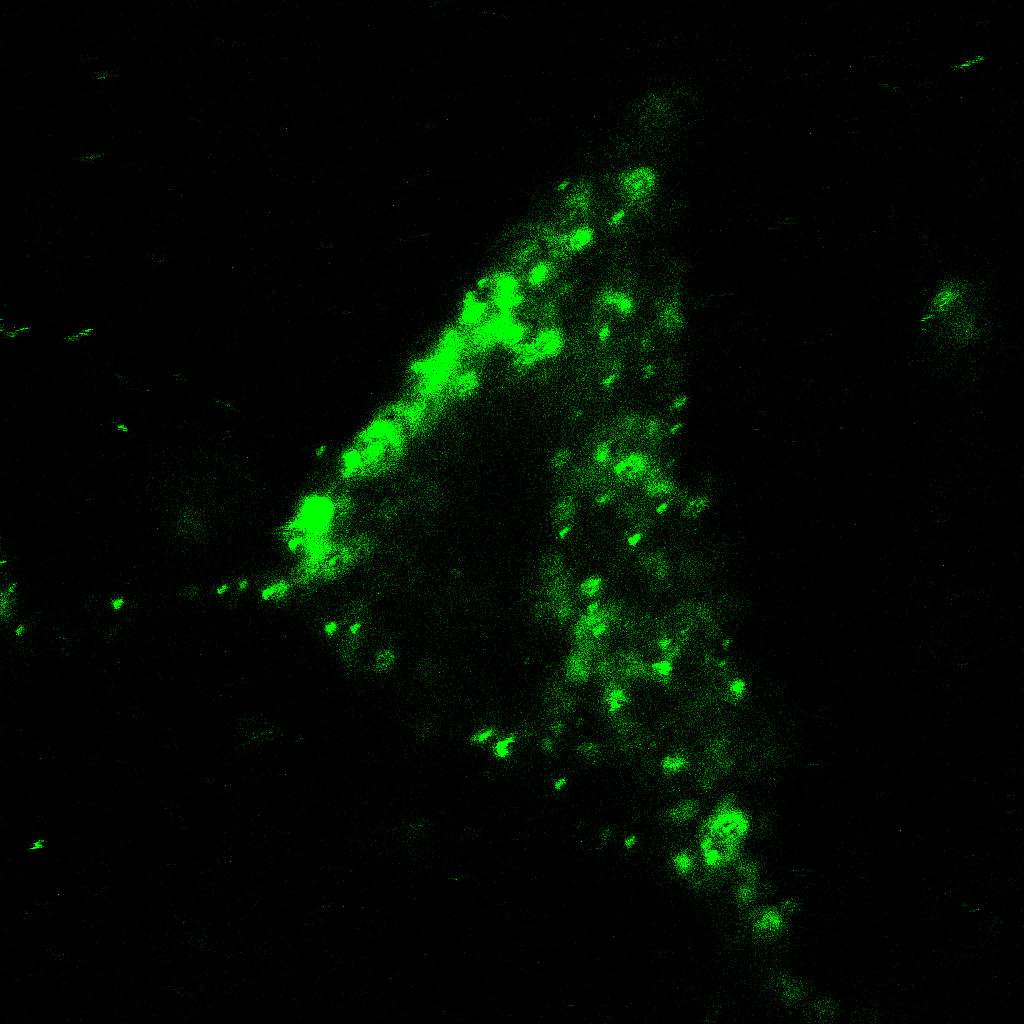

Supplement: S2 Data — (ZIP) [file ppat.1012546.s006.zip › Figure 5D/3/Flag-CASP1+GFP-UL4/GFP-UL4.tif]

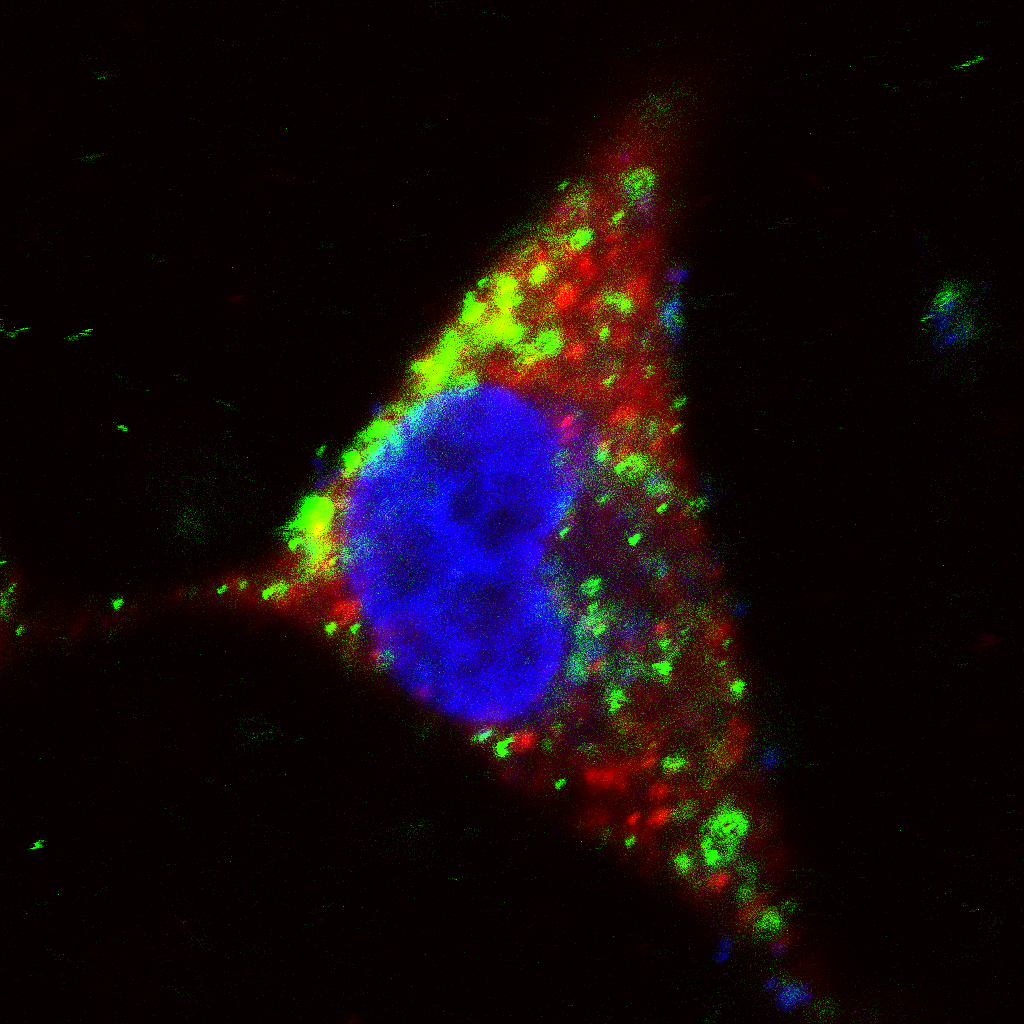

Supplement: S2 Data — (ZIP) [file ppat.1012546.s006.zip › Figure 5D/3/Flag-CASP1+GFP-UL4/Merge.tif]

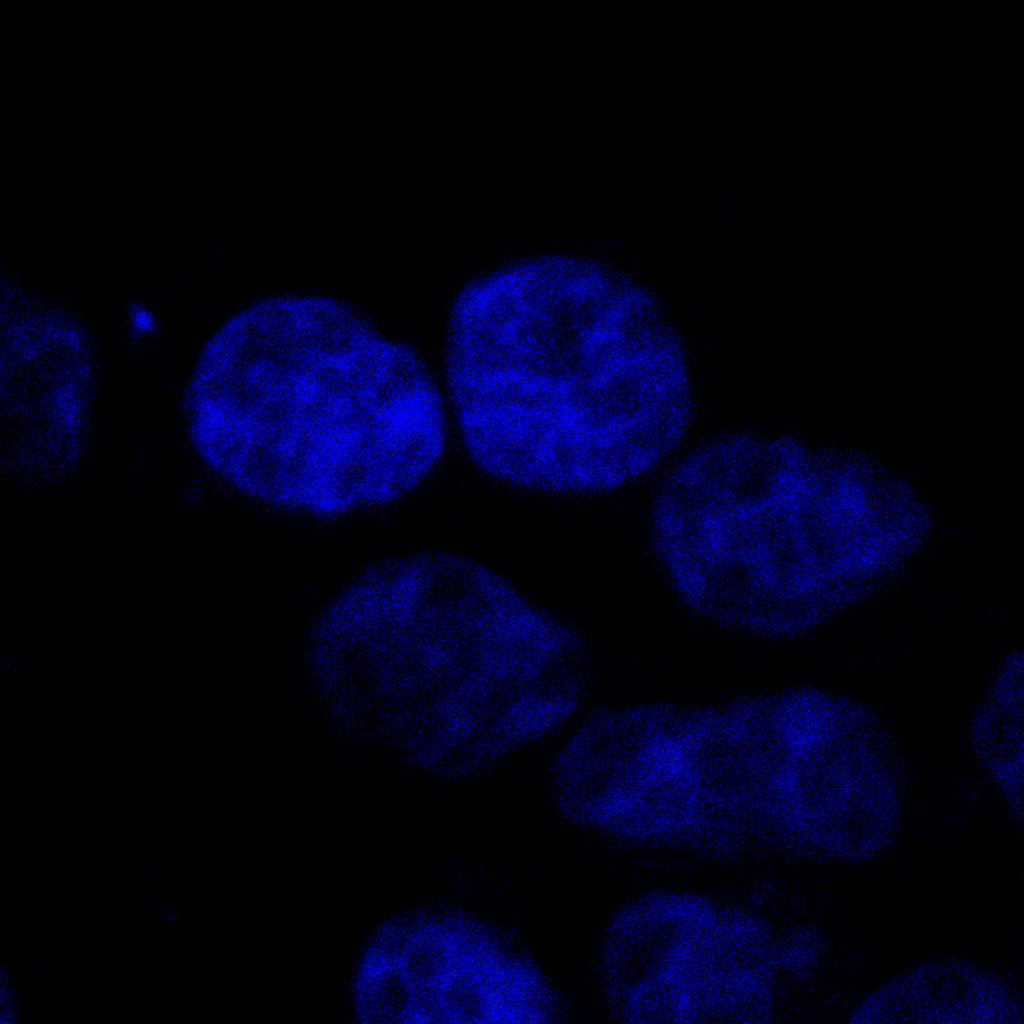

Supplement: S2 Data — (ZIP) [file ppat.1012546.s006.zip › Figure 5D/3/Flag-NLRP3/DAPI.tif]

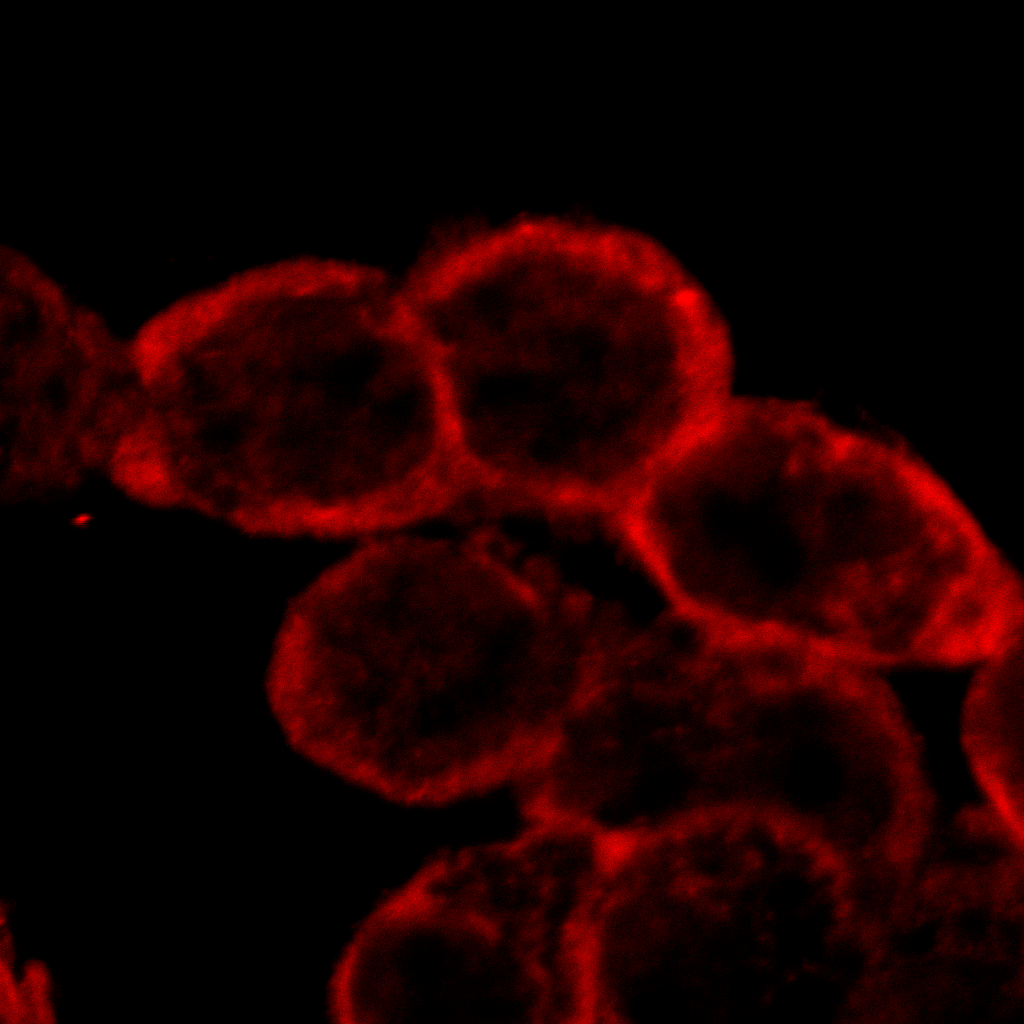

Supplement: S2 Data — (ZIP) [file ppat.1012546.s006.zip › Figure 5D/3/Flag-NLRP3/flag-nlrp3.tif]

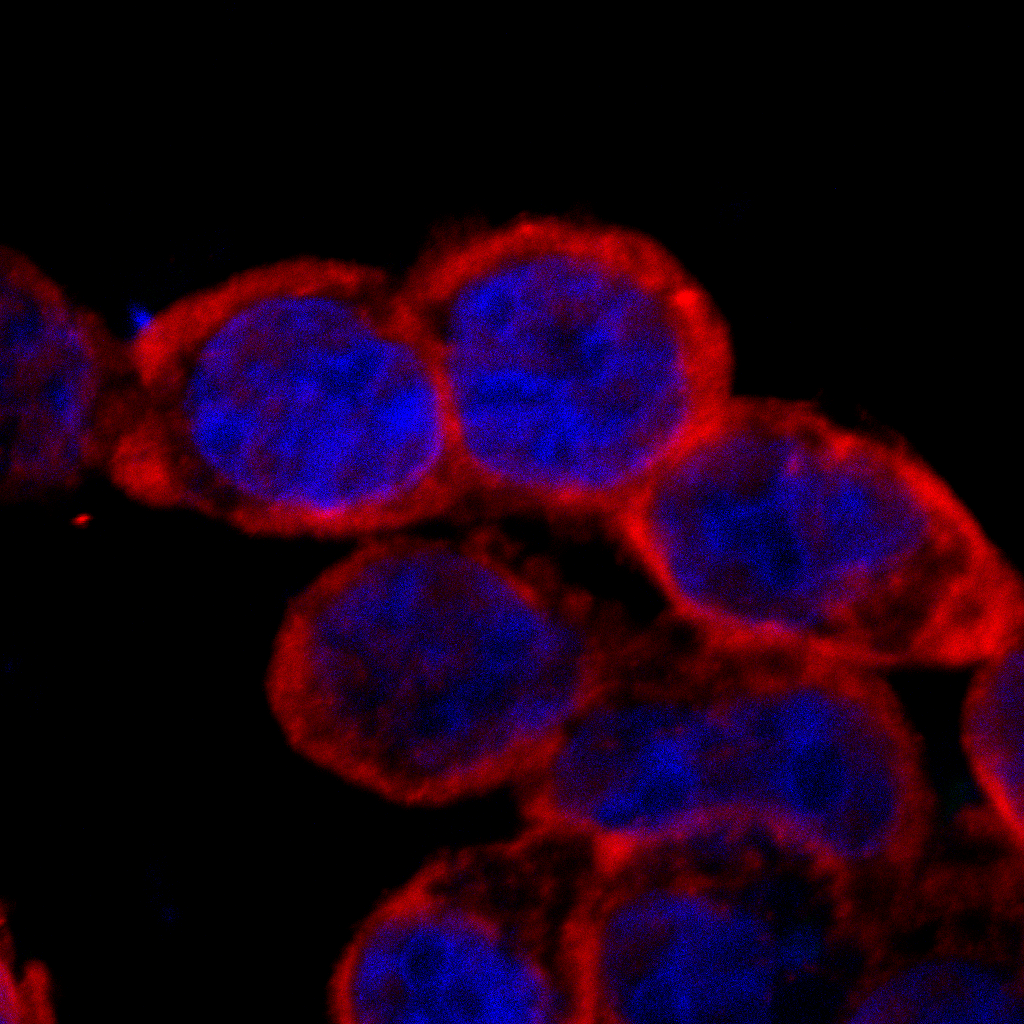

Supplement: S2 Data — (ZIP) [file ppat.1012546.s006.zip › Figure 5D/3/Flag-NLRP3/Merge.tif]

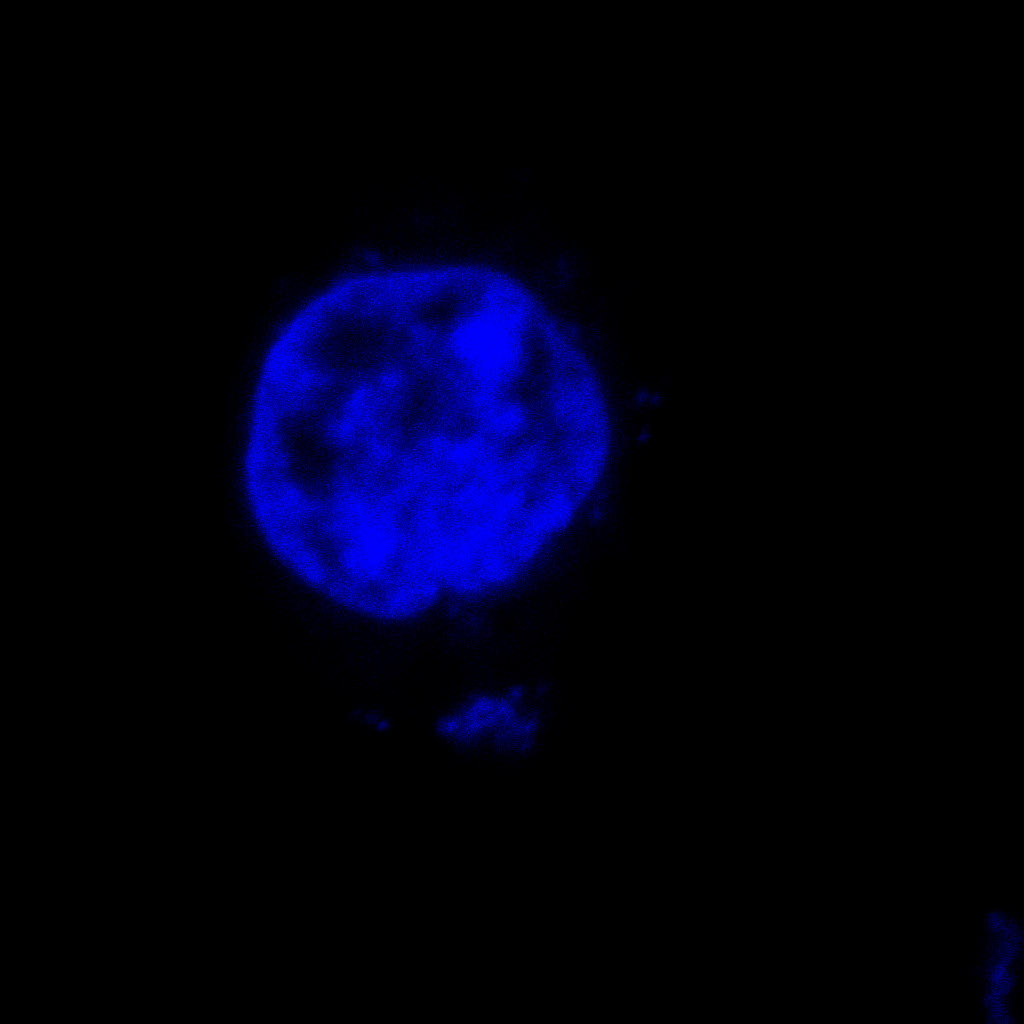

Supplement: S2 Data — (ZIP) [file ppat.1012546.s006.zip › Figure 5D/3/Flag-NLRP3+GFP-UL4/DAPI.tif]

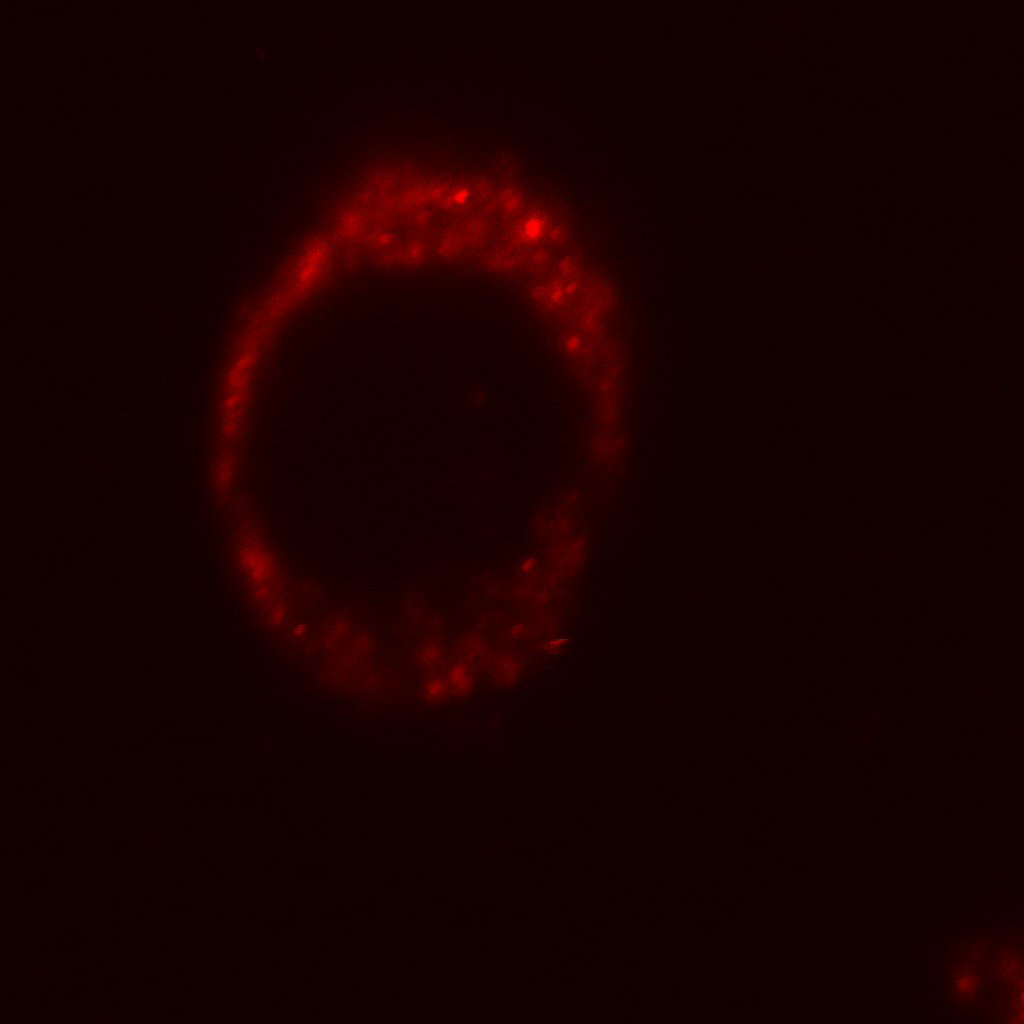

Supplement: S2 Data — (ZIP) [file ppat.1012546.s006.zip › Figure 5D/3/Flag-NLRP3+GFP-UL4/flag-nlrp3.tif]

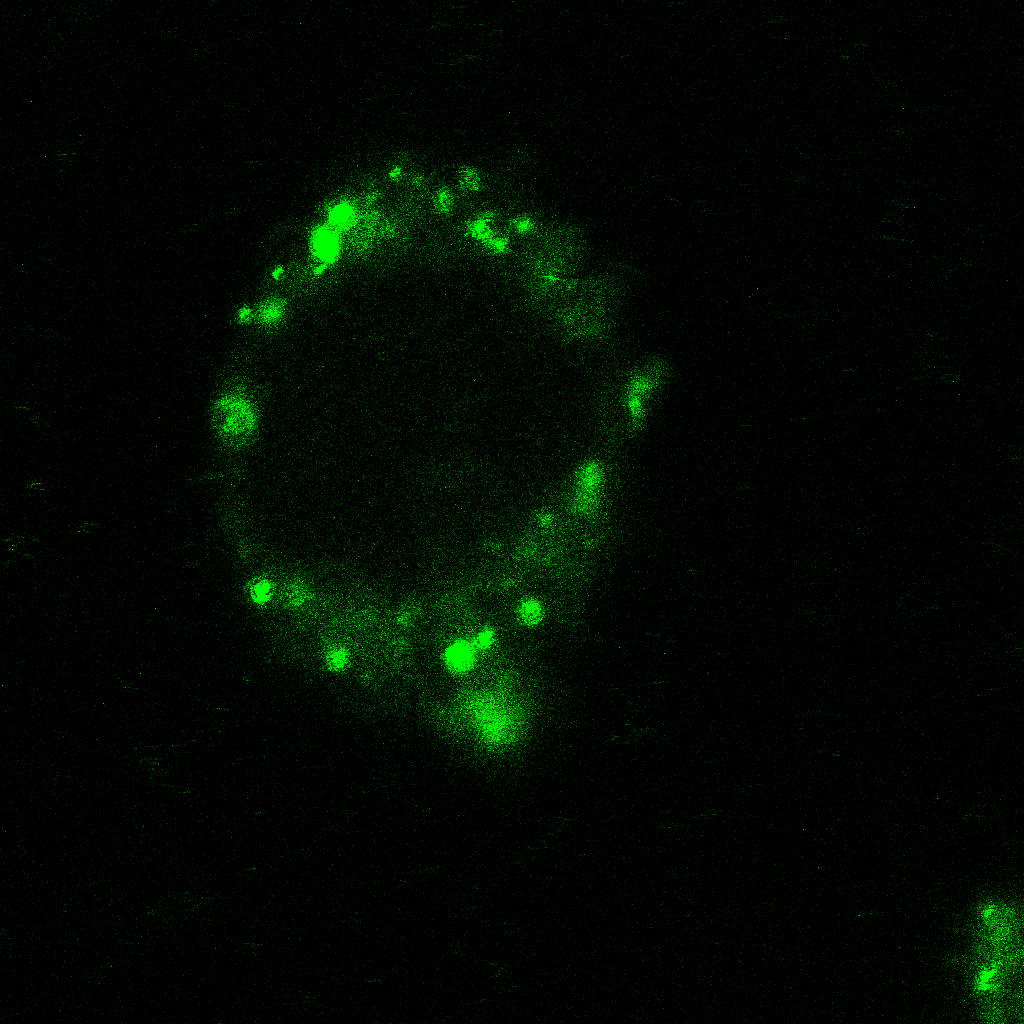

Supplement: S2 Data — (ZIP) [file ppat.1012546.s006.zip › Figure 5D/3/Flag-NLRP3+GFP-UL4/gfp-ul4.tif]

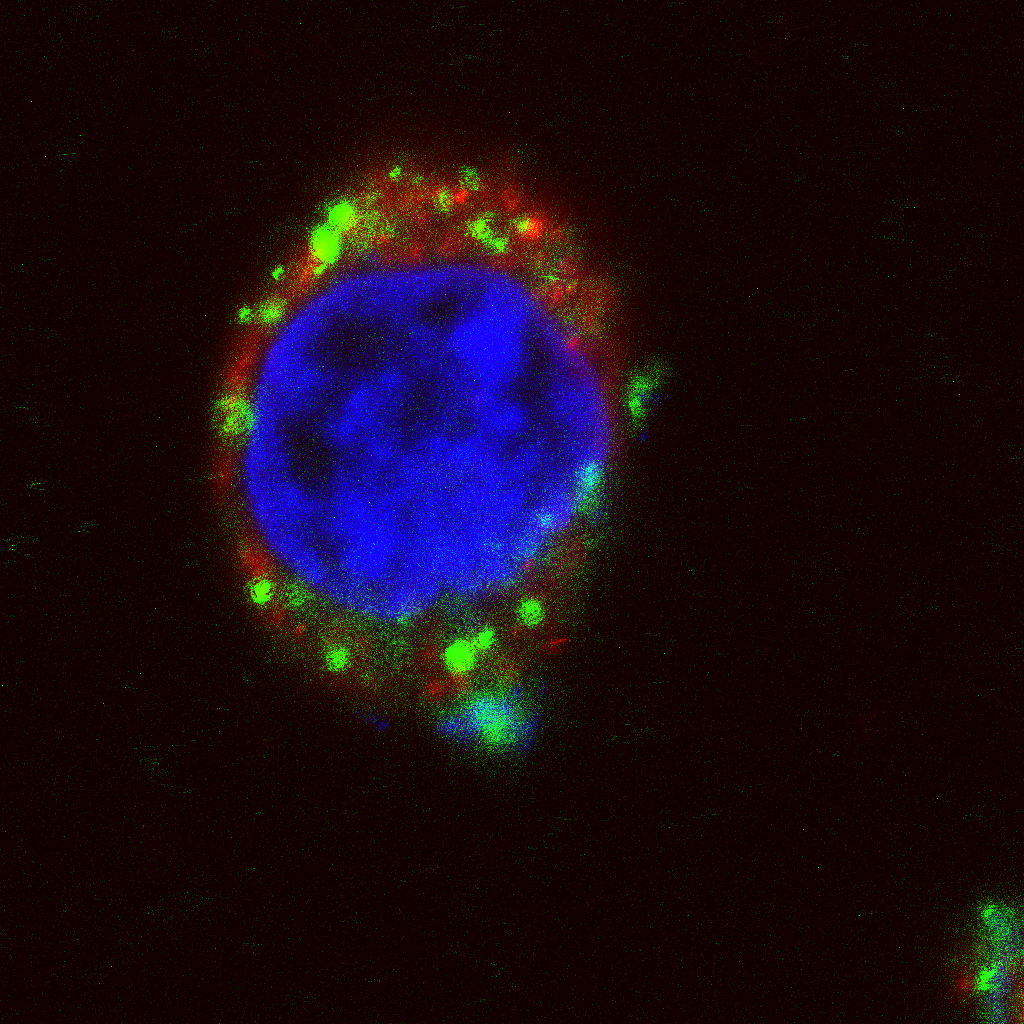

Supplement: S2 Data — (ZIP) [file ppat.1012546.s006.zip › Figure 5D/3/Flag-NLRP3+GFP-UL4/Merge.tif]

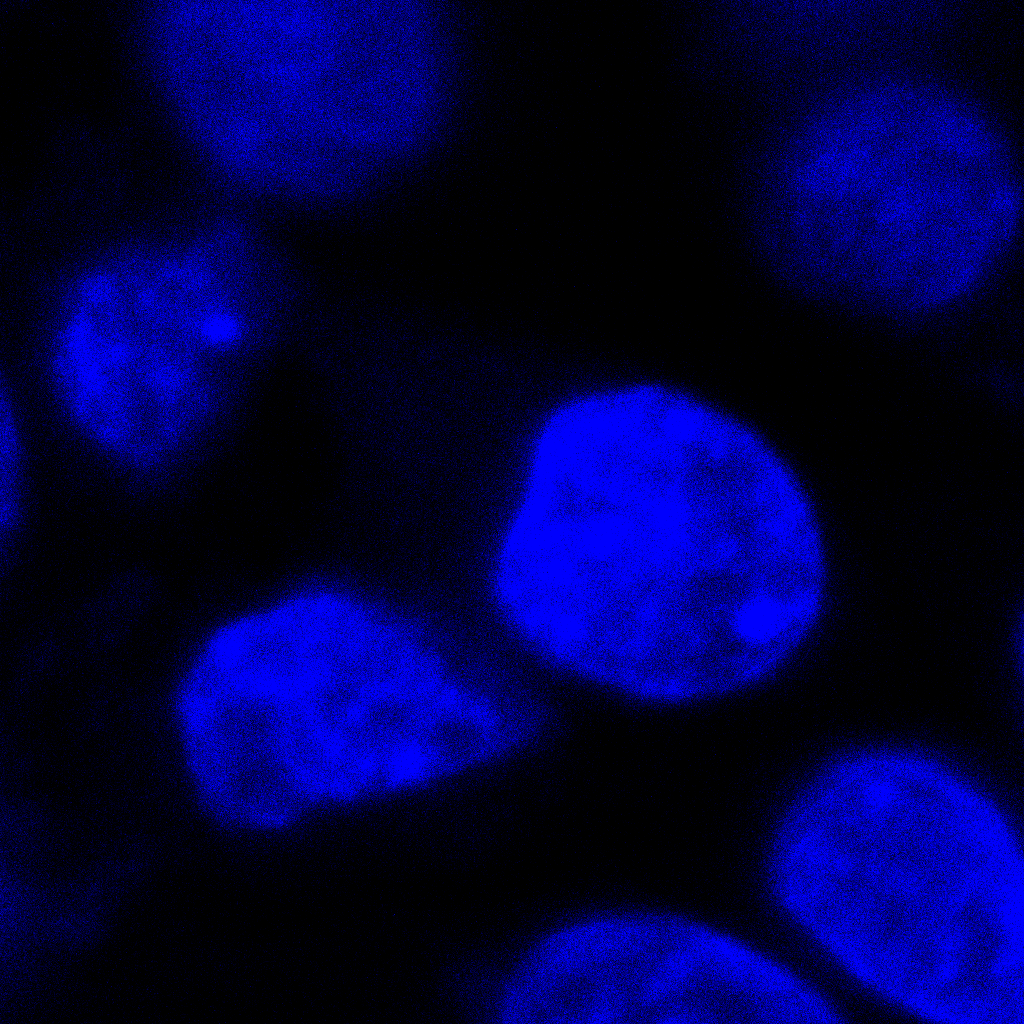

Supplement: S2 Data — (ZIP) [file ppat.1012546.s006.zip › Figure 5D/3/GFP-UL4/DAPI.tif]

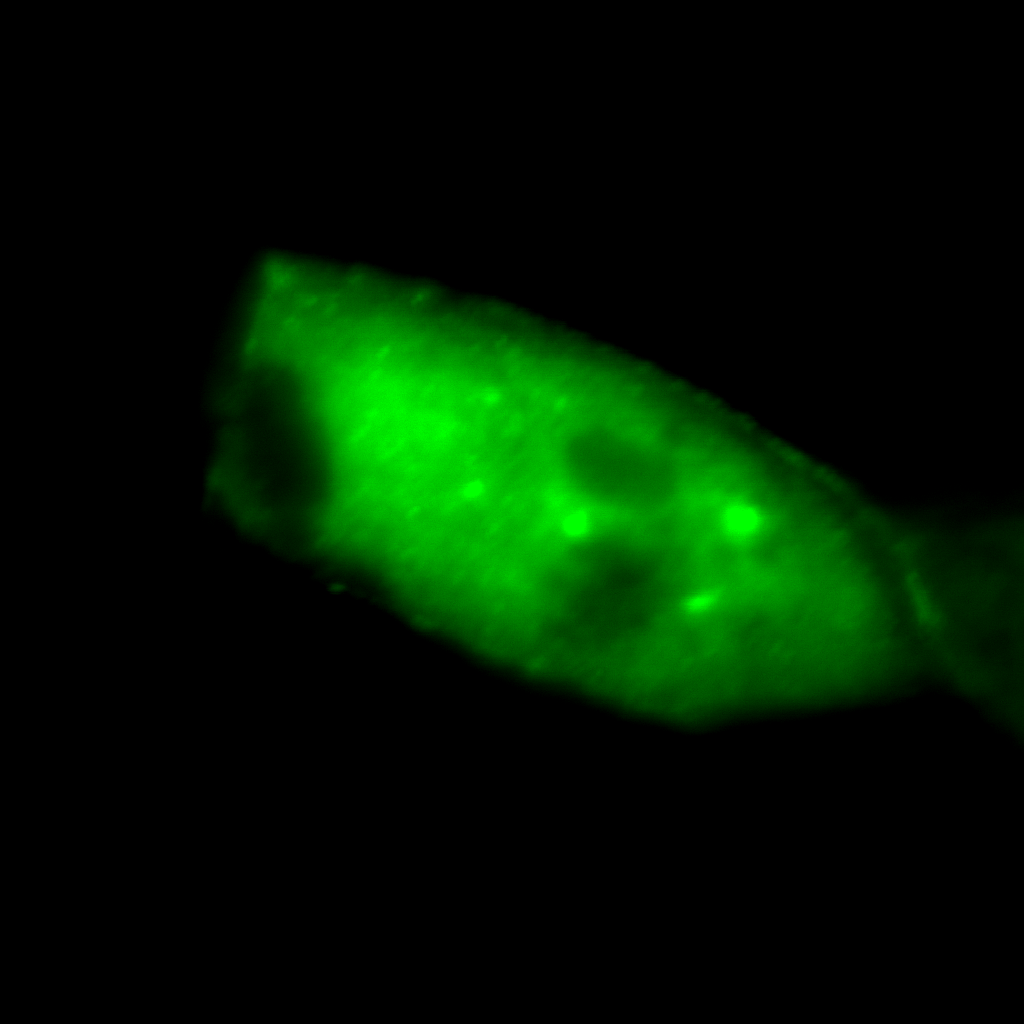

Supplement: S2 Data — (ZIP) [file ppat.1012546.s006.zip › Figure 5D/3/GFP-UL4/gfp-ul4.tif]

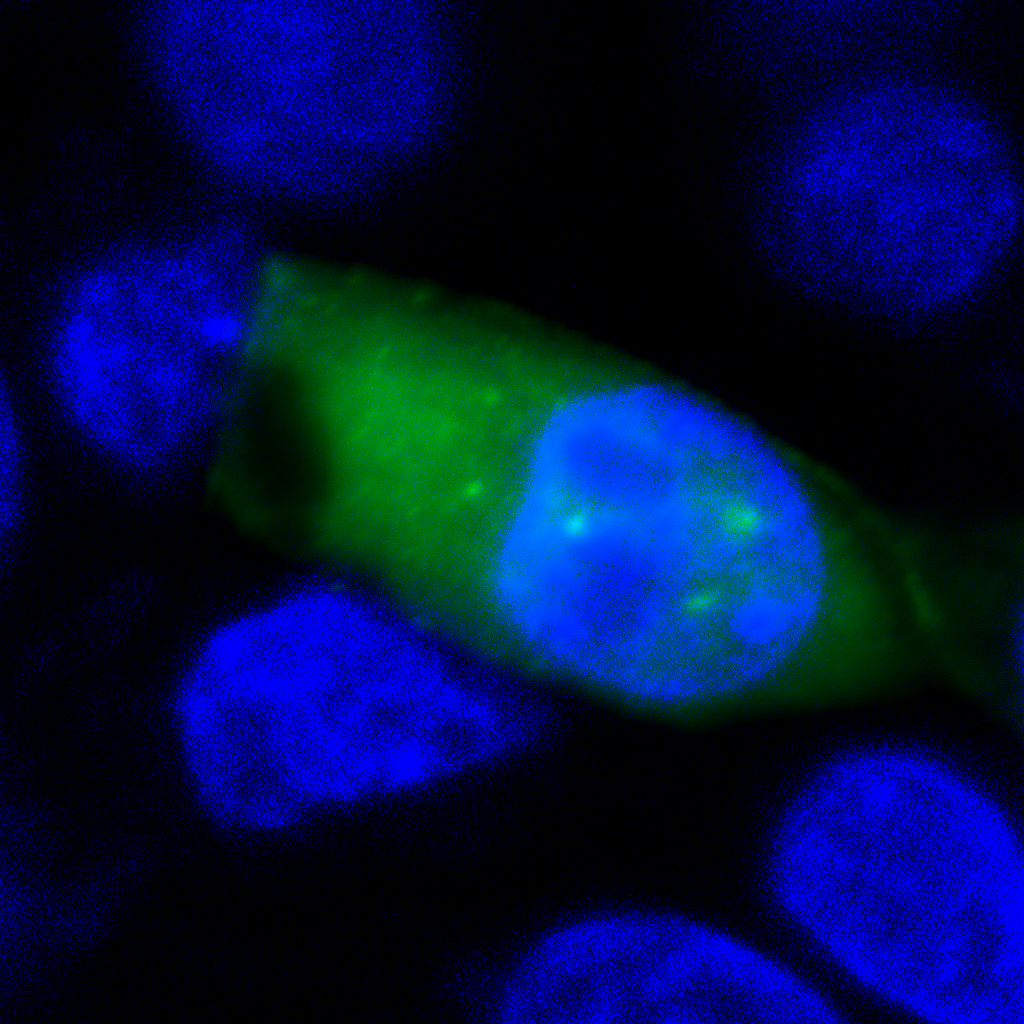

Supplement: S2 Data — (ZIP) [file ppat.1012546.s006.zip › Figure 5D/3/GFP-UL4/Merge.tif]

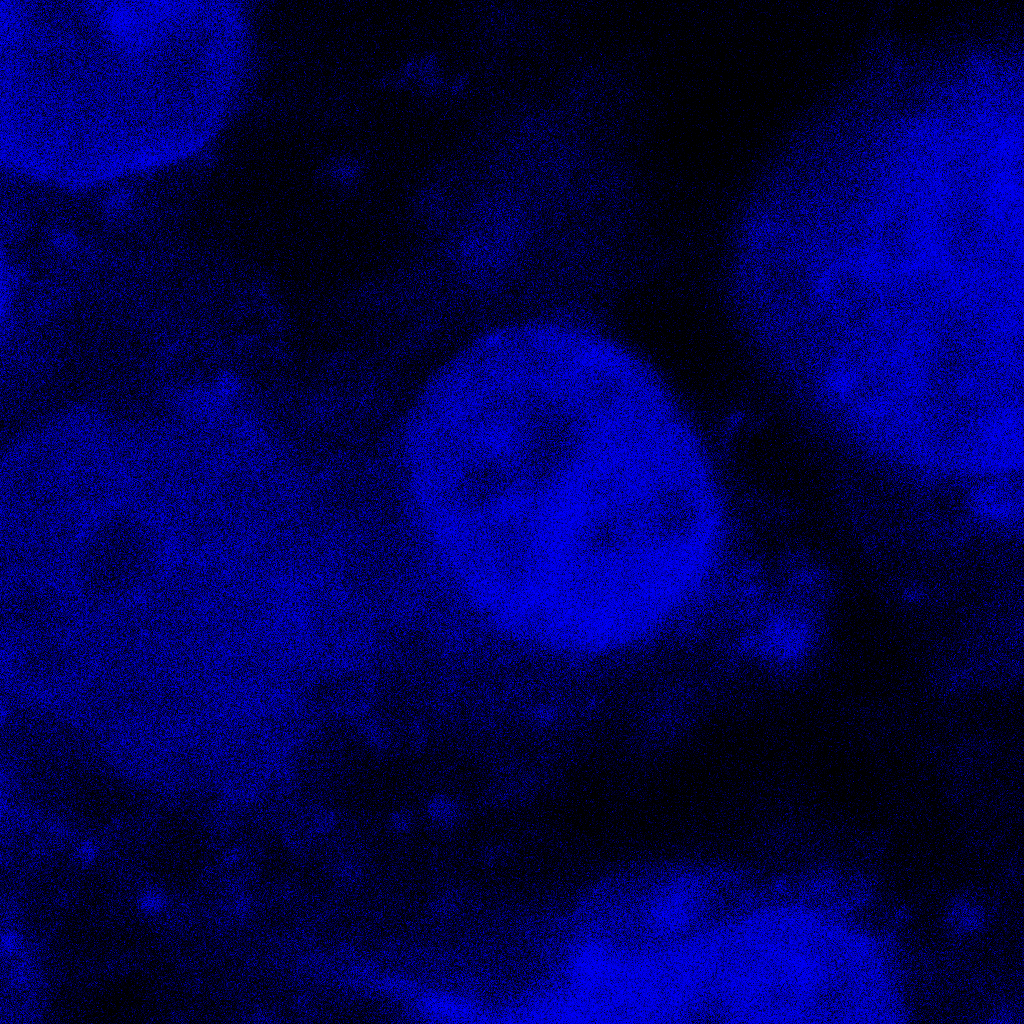

Supplement: S3 Data — (ZIP) [file ppat.1012546.s007.zip › Figure6D/1/flag-ASC+GFP-UL4(1-73)/DAPI.tif]

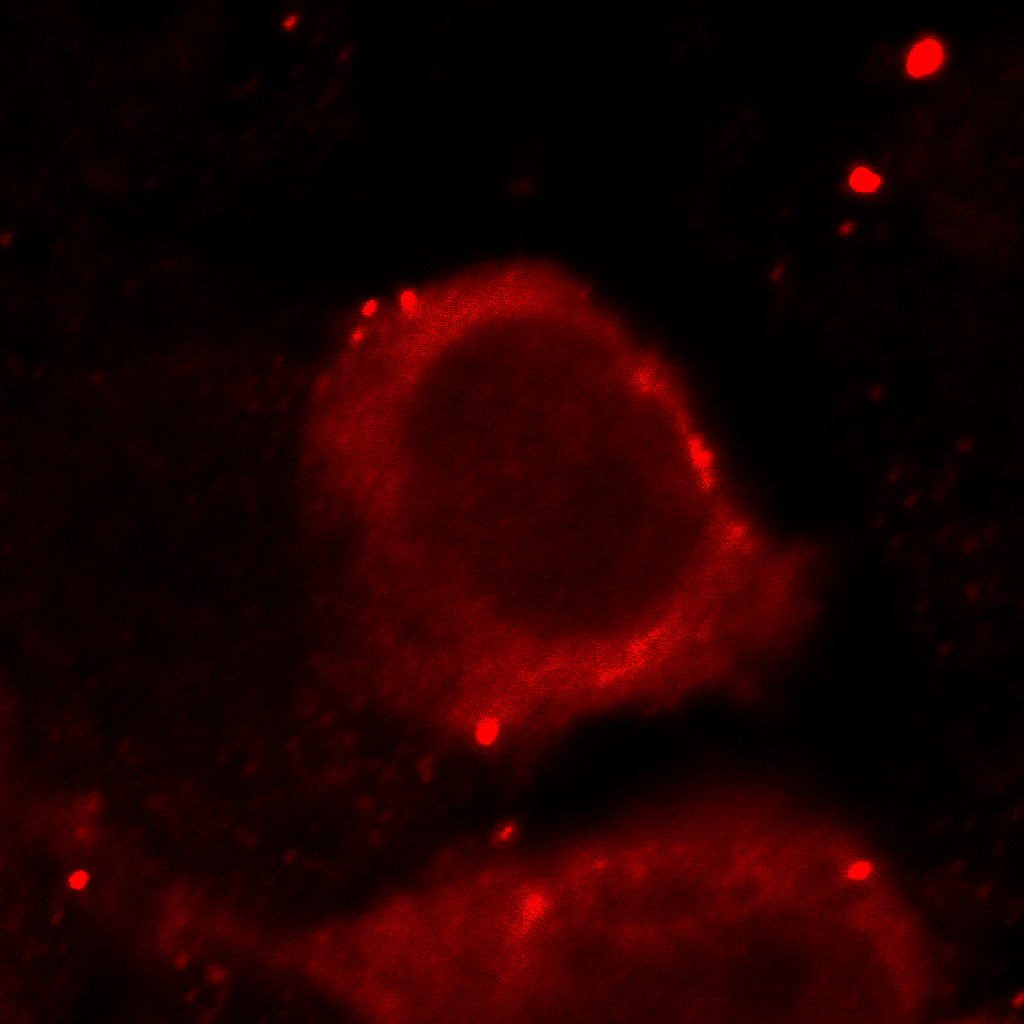

Supplement: S3 Data — (ZIP) [file ppat.1012546.s007.zip › Figure6D/1/flag-ASC+GFP-UL4(1-73)/Flag-ASC.tif]

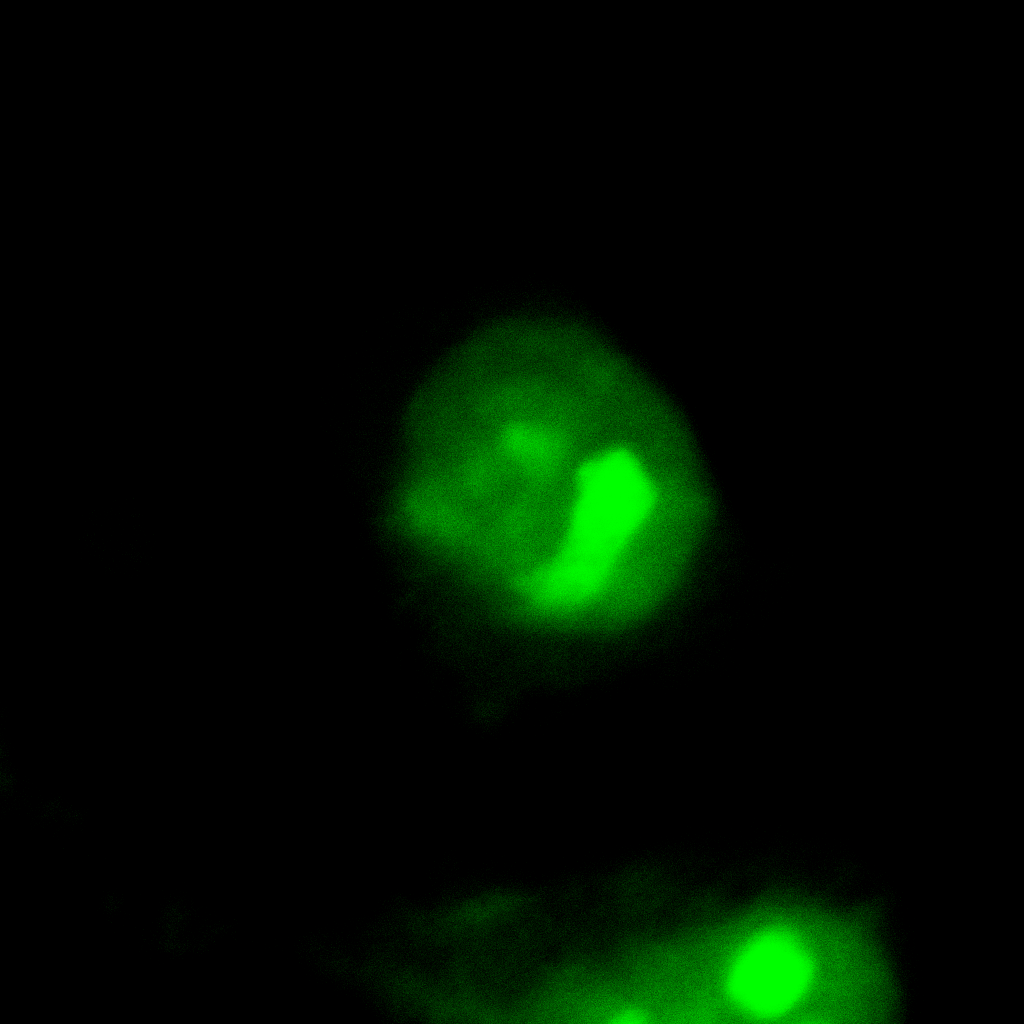

Supplement: S3 Data — (ZIP) [file ppat.1012546.s007.zip › Figure6D/1/flag-ASC+GFP-UL4(1-73)/GFP-UL4(1-73).tif]

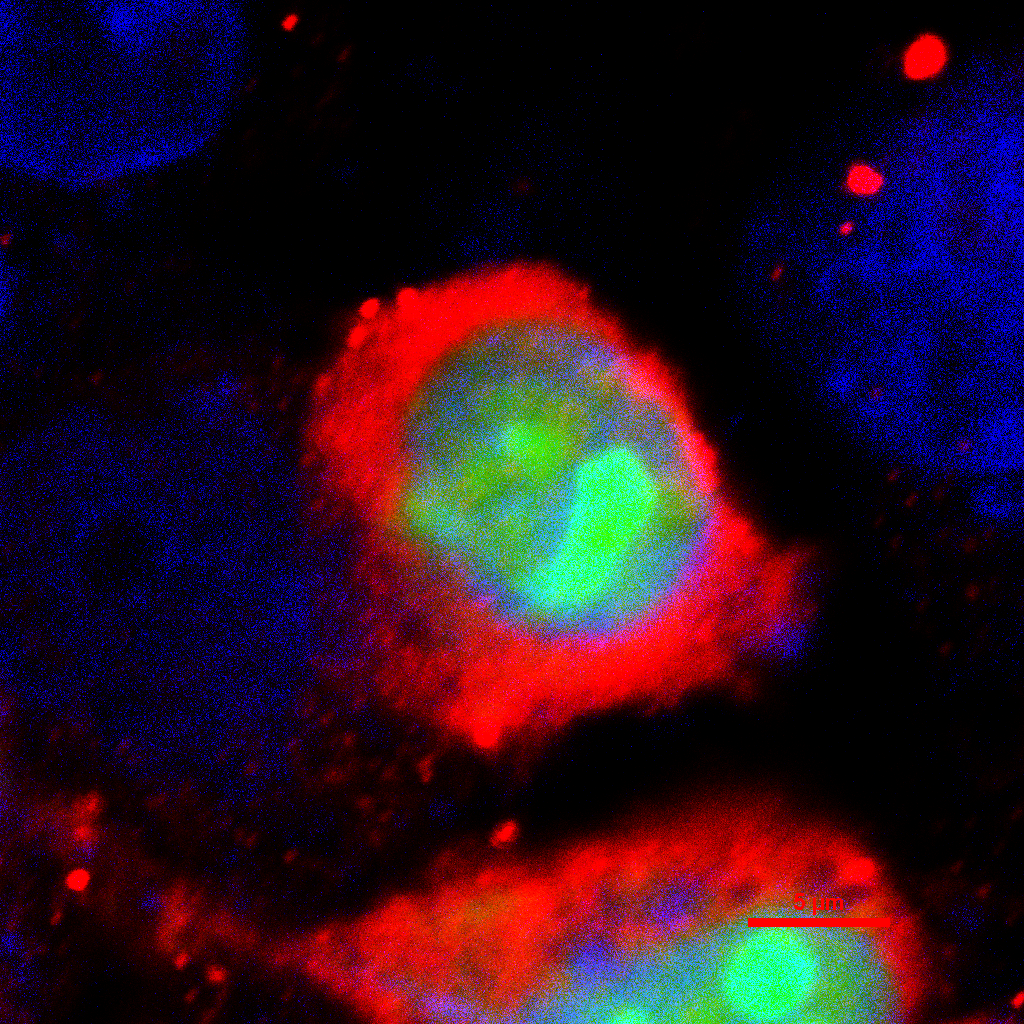

Supplement: S3 Data — (ZIP) [file ppat.1012546.s007.zip › Figure6D/1/flag-ASC+GFP-UL4(1-73)/Merge.tif]

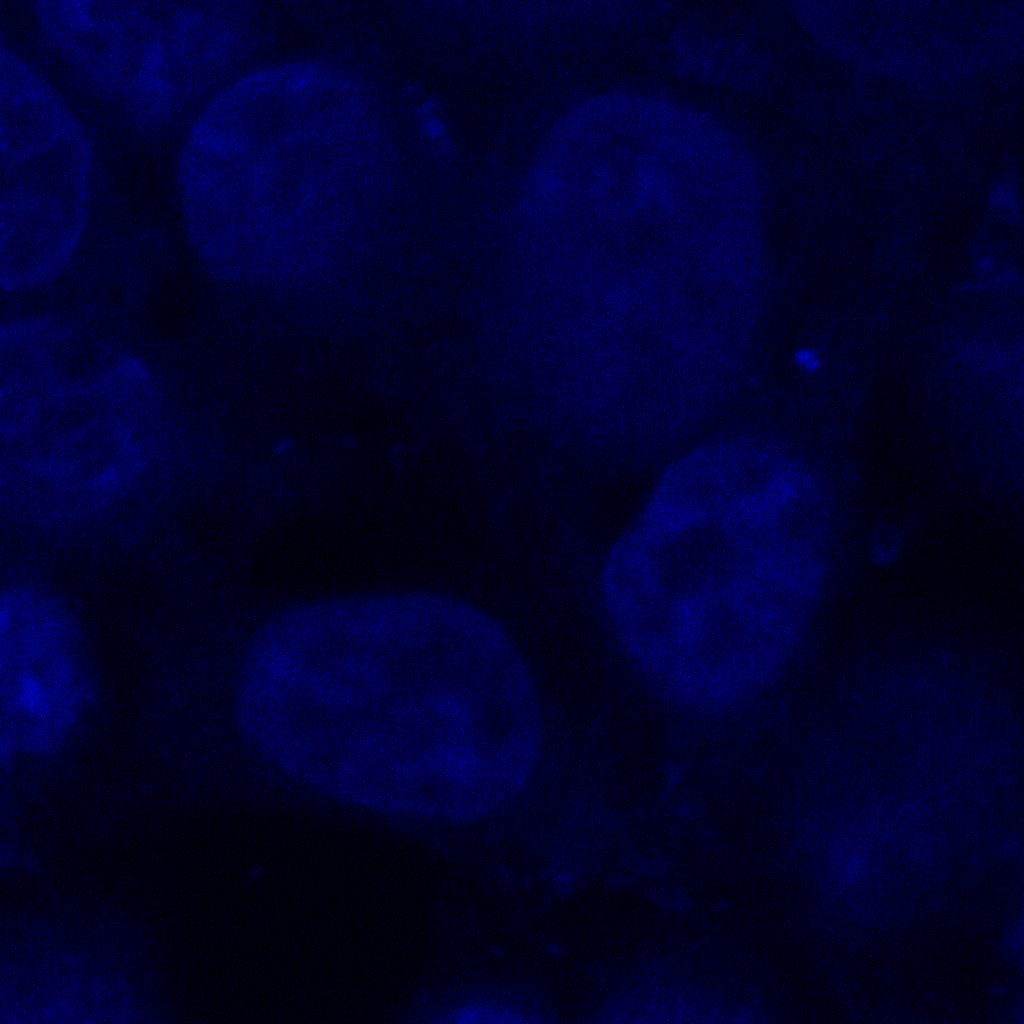

Supplement: S3 Data — (ZIP) [file ppat.1012546.s007.zip › Figure6D/1/flag-ASC+GFP-UL4(50-130)/DAPI.tif]

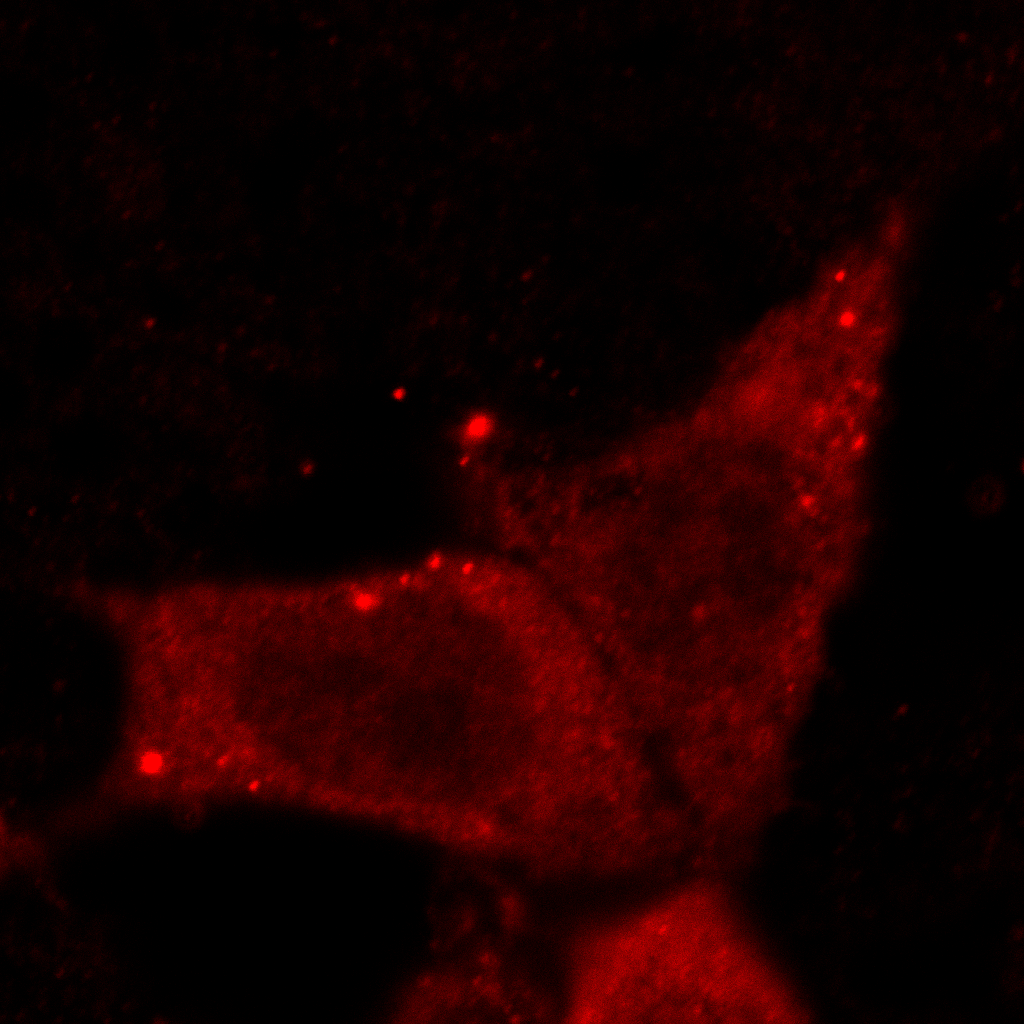

Supplement: S3 Data — (ZIP) [file ppat.1012546.s007.zip › Figure6D/1/flag-ASC+GFP-UL4(50-130)/flag-asc.tif]

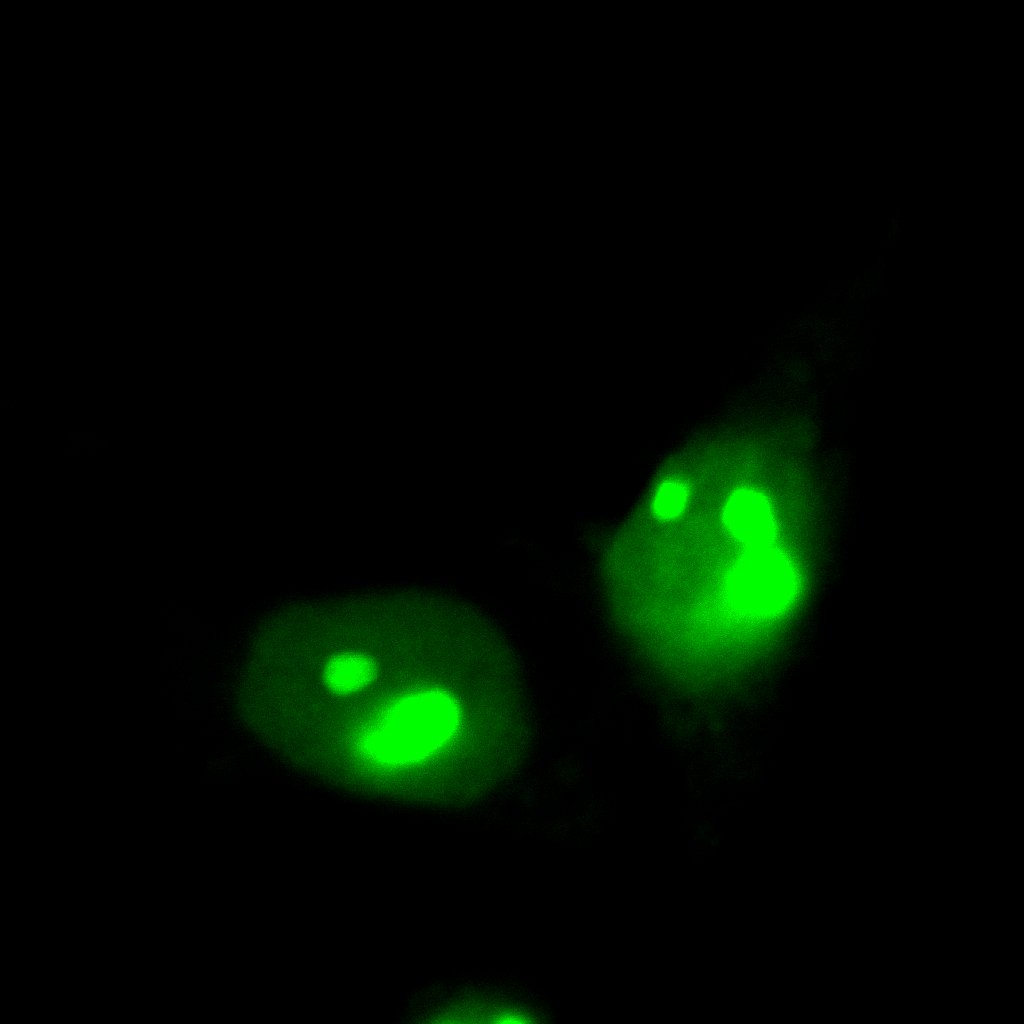

Supplement: S3 Data — (ZIP) [file ppat.1012546.s007.zip › Figure6D/1/flag-ASC+GFP-UL4(50-130)/gfp-ul4 ú¿50-130ú⌐.tif]

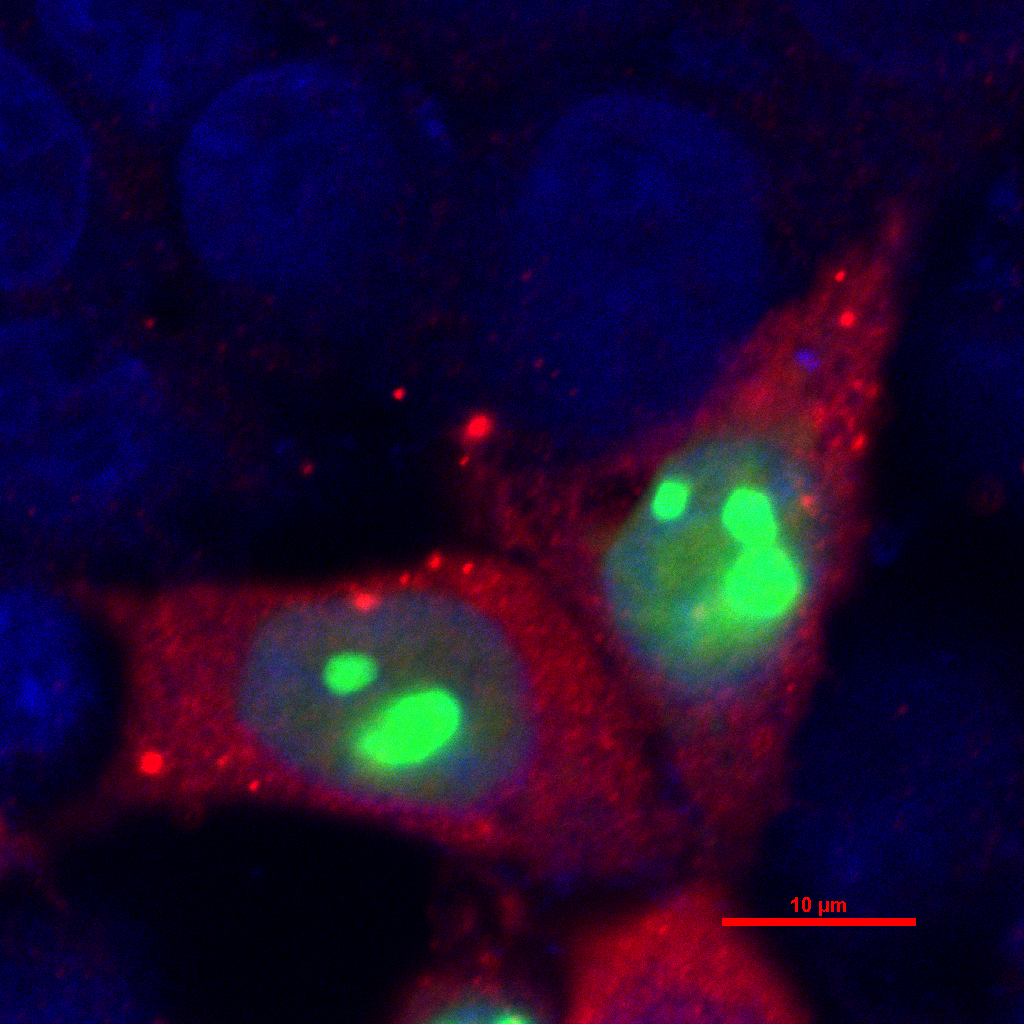

Supplement: S3 Data — (ZIP) [file ppat.1012546.s007.zip › Figure6D/1/flag-ASC+GFP-UL4(50-130)/Merge.tif]

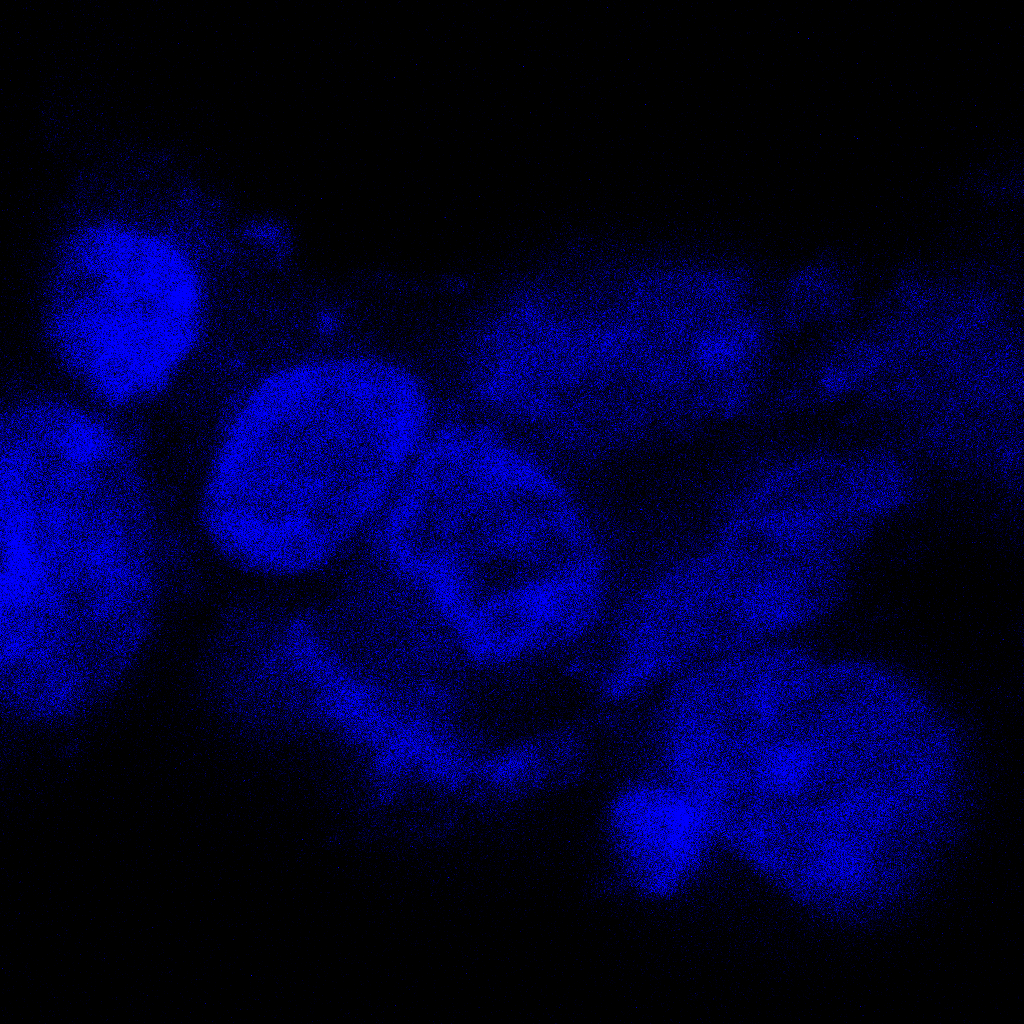

Supplement: S3 Data — (ZIP) [file ppat.1012546.s007.zip › Figure6D/1/flag-ASC+GFP-UL4(73-146)/DAPI.tif]

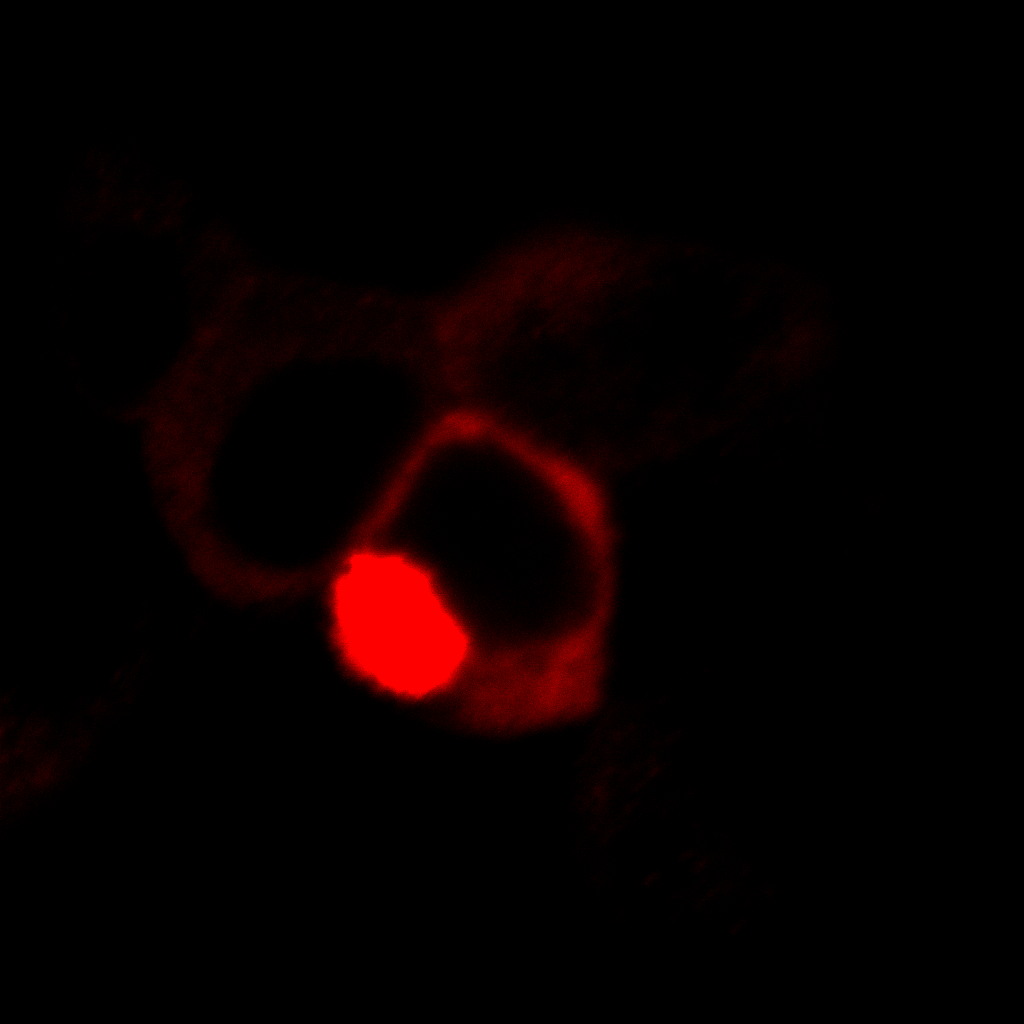

Supplement: S3 Data — (ZIP) [file ppat.1012546.s007.zip › Figure6D/1/flag-ASC+GFP-UL4(73-146)/Flag-ASC.tif]

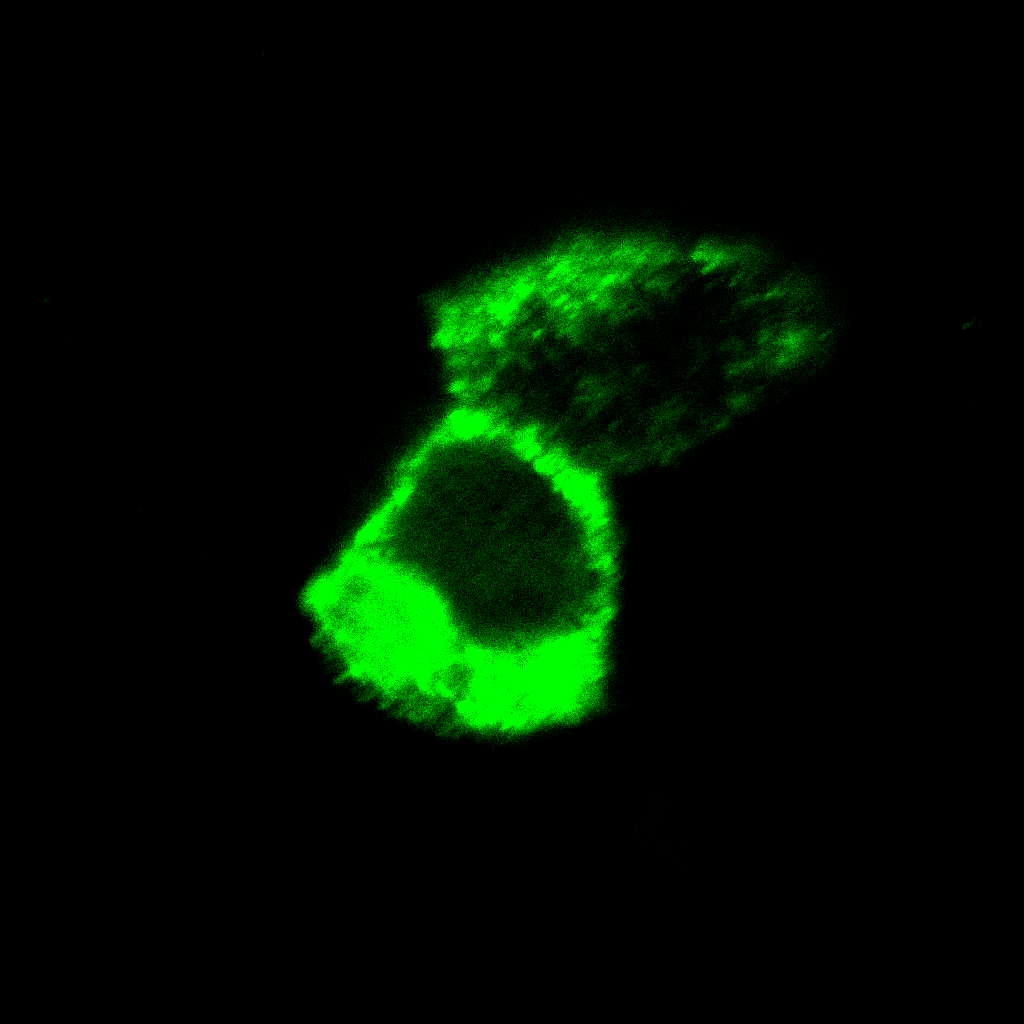

Supplement: S3 Data — (ZIP) [file ppat.1012546.s007.zip › Figure6D/1/flag-ASC+GFP-UL4(73-146)/GFP-UL4ú¿73-146).tif]

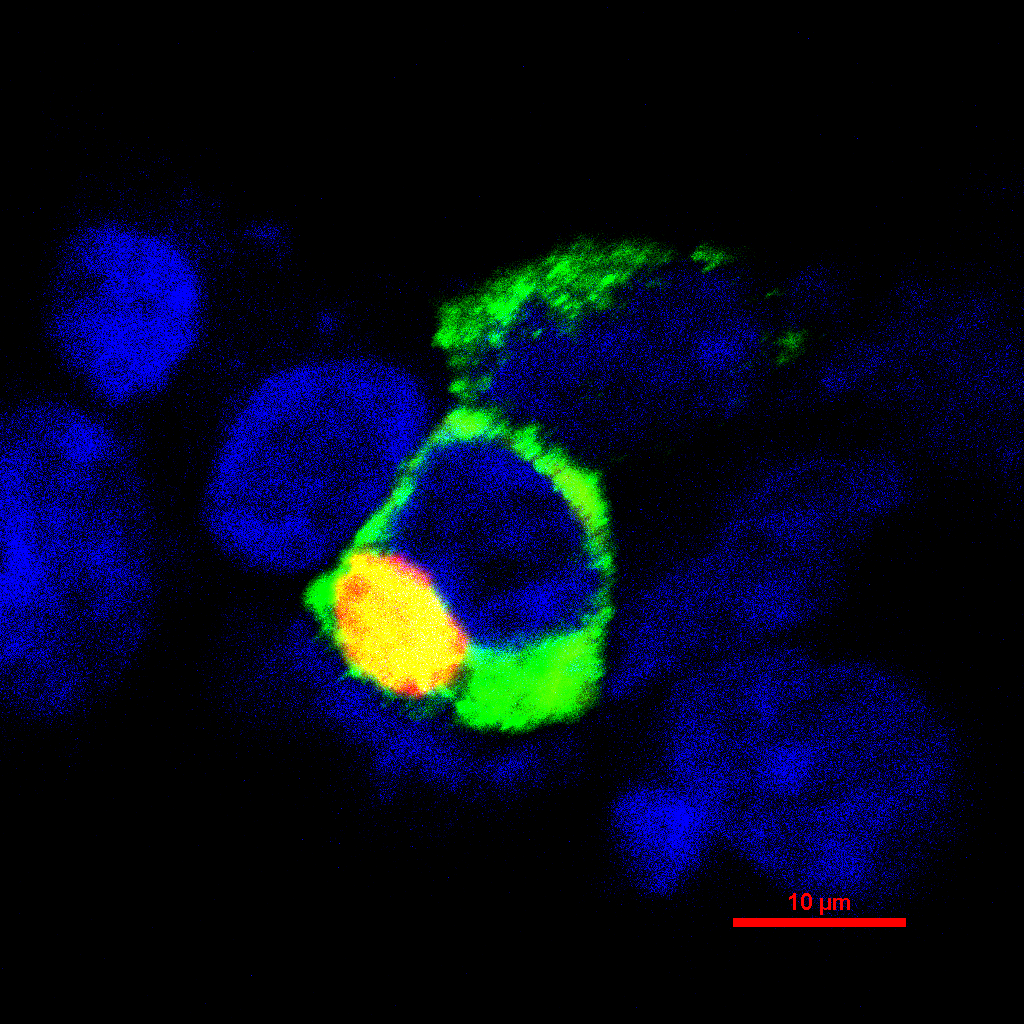

Supplement: S3 Data — (ZIP) [file ppat.1012546.s007.zip › Figure6D/1/flag-ASC+GFP-UL4(73-146)/Merge.tif]

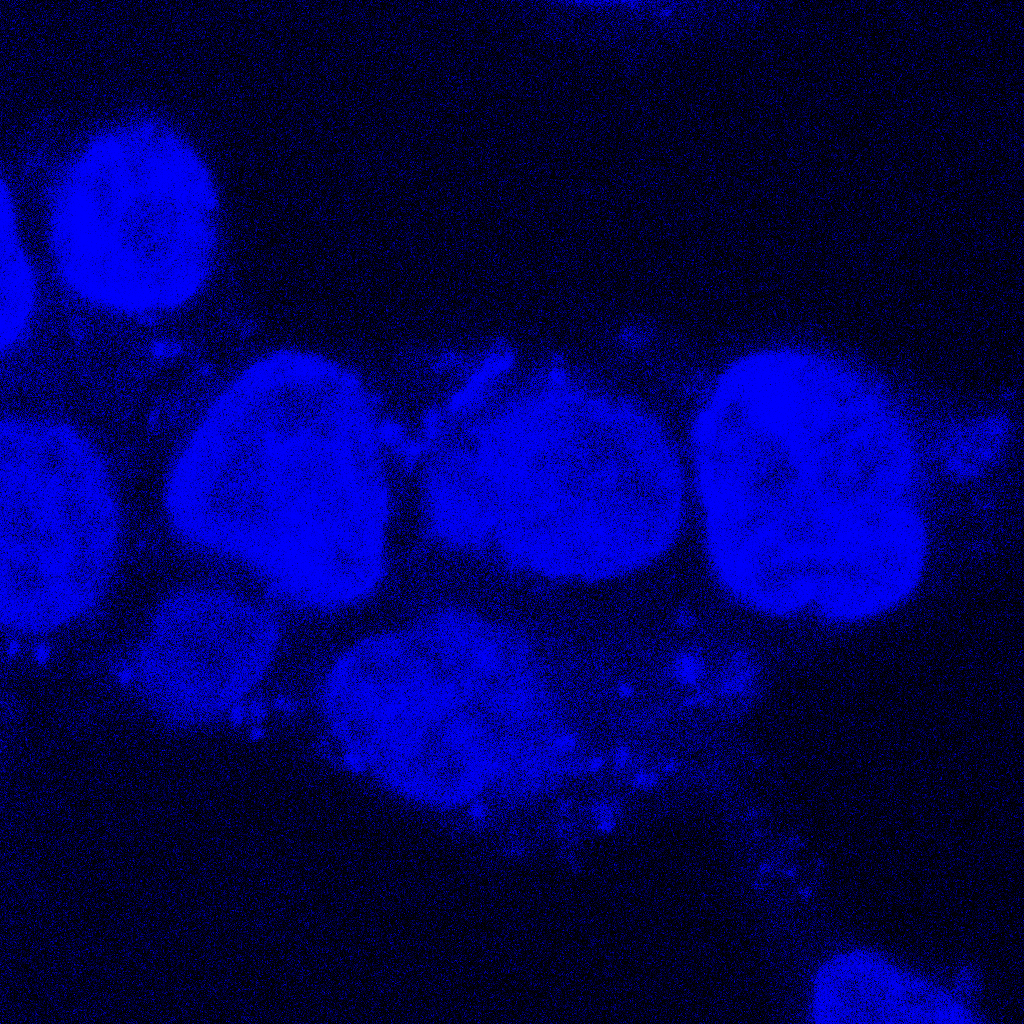

Supplement: S3 Data — (ZIP) [file ppat.1012546.s007.zip › Figure6D/1/GFP-UL4(1-73)/DAPI.tif]

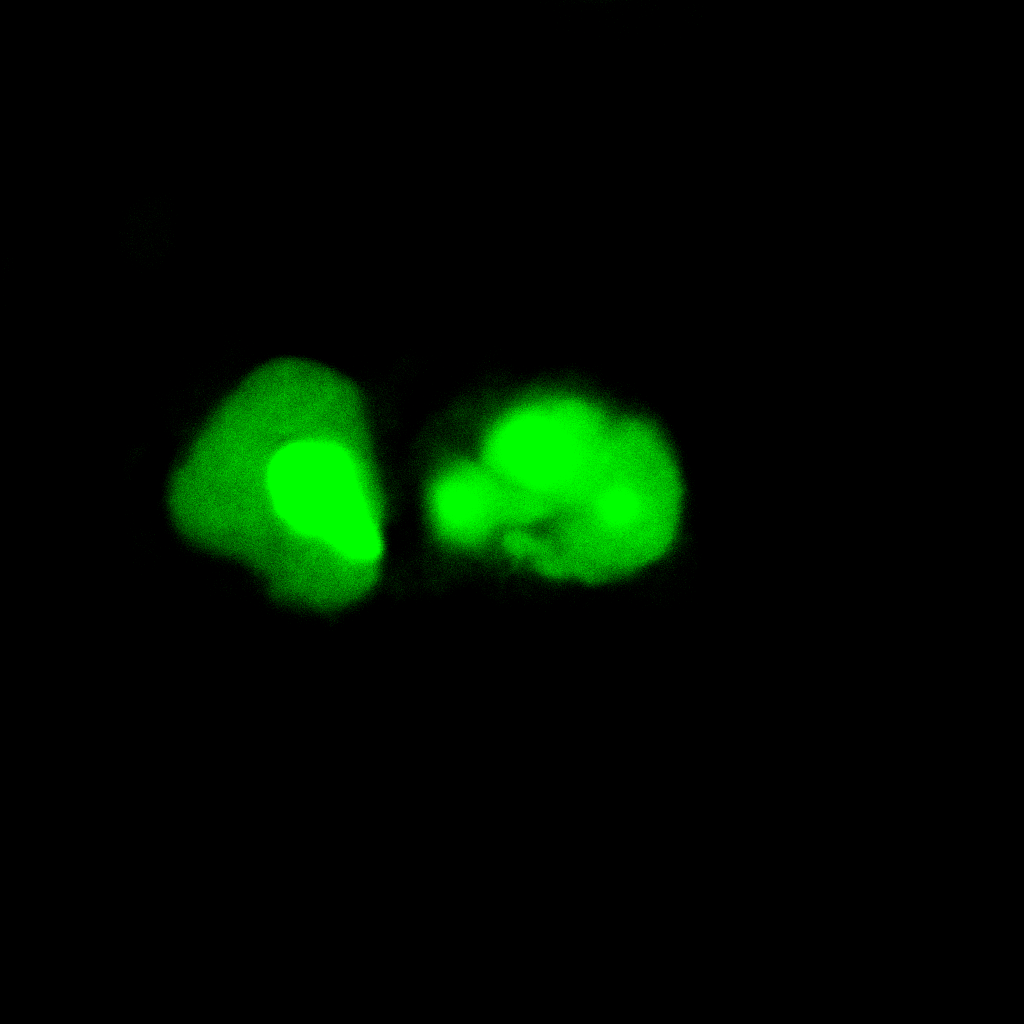

Supplement: S3 Data — (ZIP) [file ppat.1012546.s007.zip › Figure6D/1/GFP-UL4(1-73)/GFP-UL4(1-73).tif]

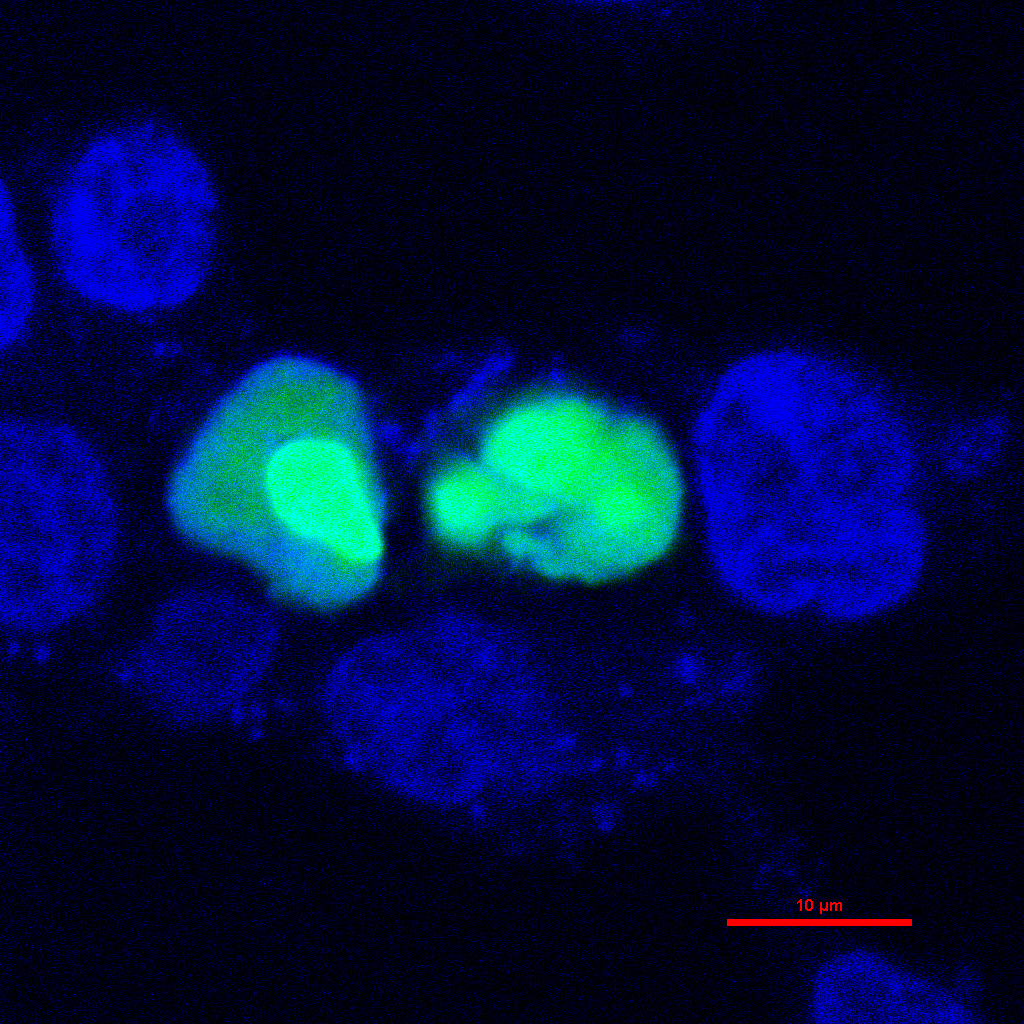

Supplement: S3 Data — (ZIP) [file ppat.1012546.s007.zip › Figure6D/1/GFP-UL4(1-73)/Merge.tif]

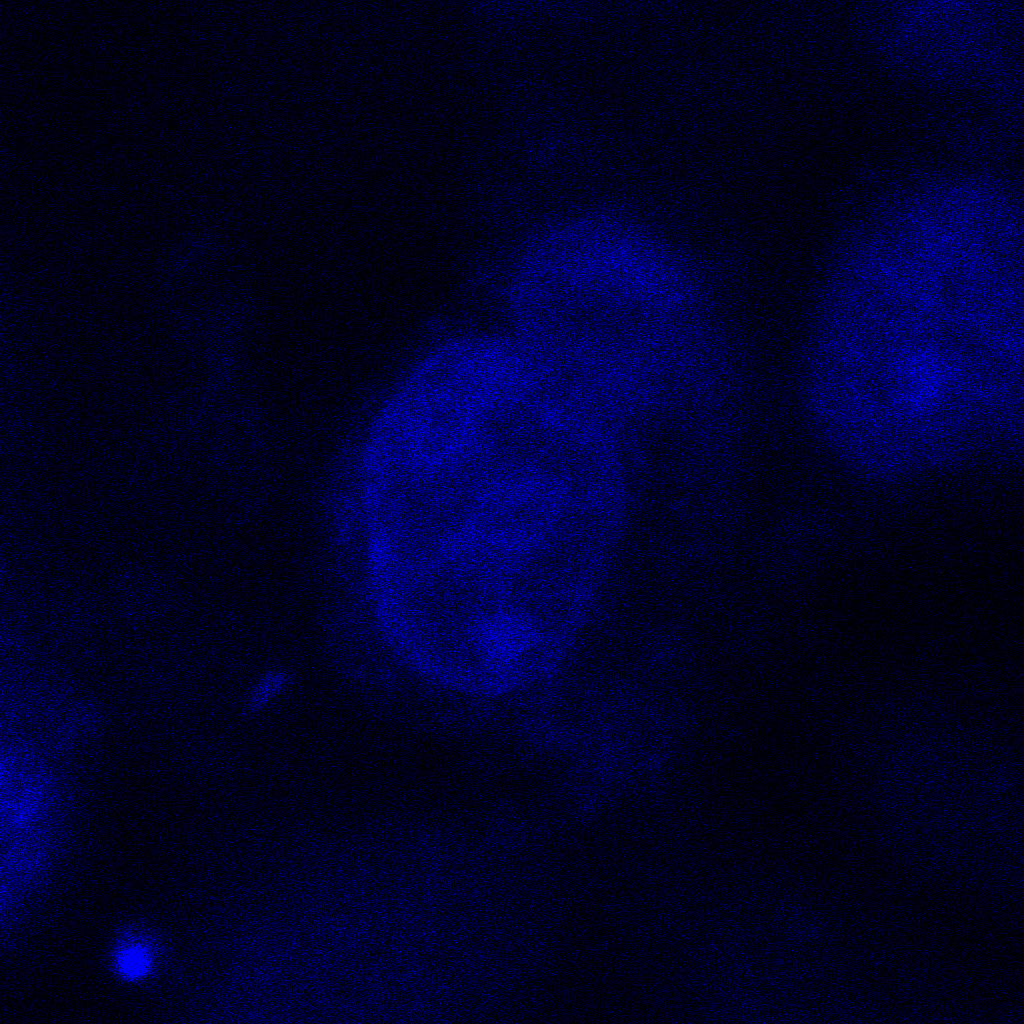

Supplement: S3 Data — (ZIP) [file ppat.1012546.s007.zip › Figure6D/1/GFP-UL4(50-130)/DAPI.tif]

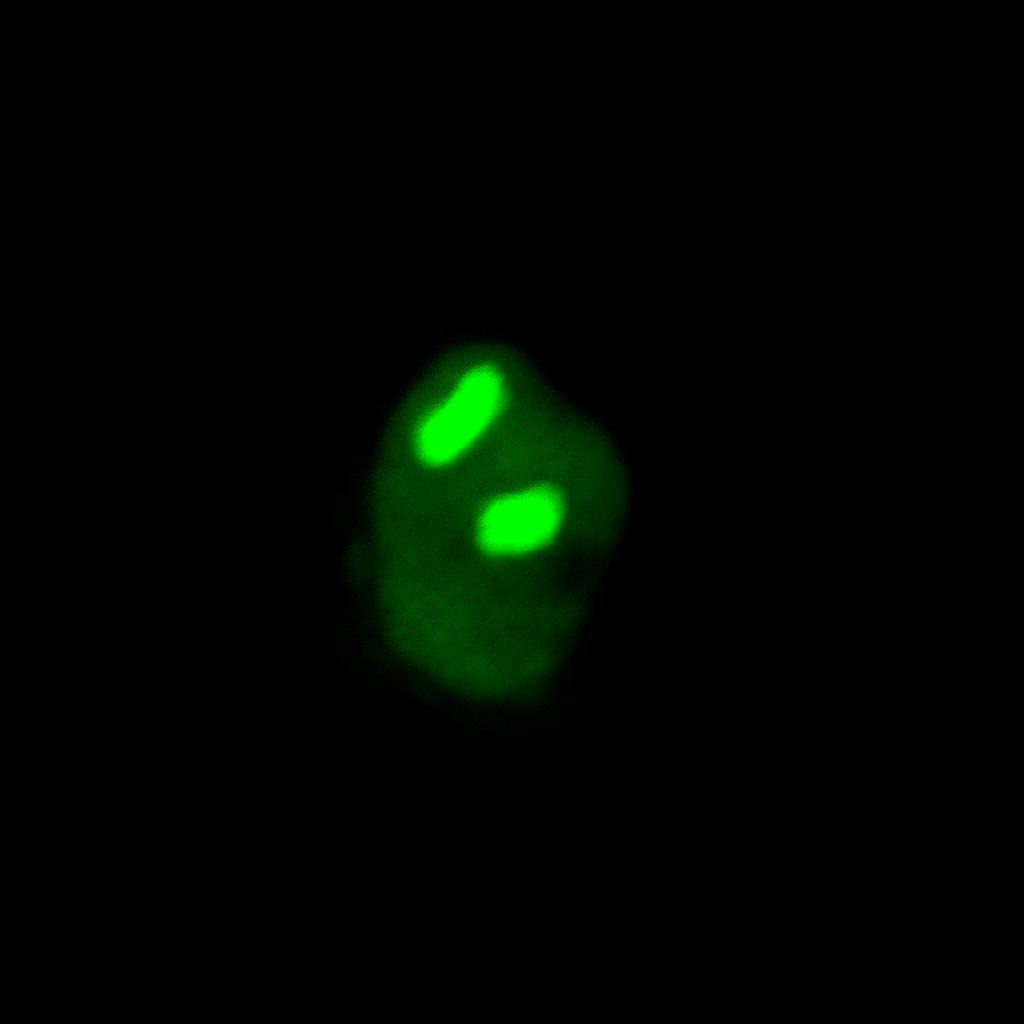

Supplement: S3 Data — (ZIP) [file ppat.1012546.s007.zip › Figure6D/1/GFP-UL4(50-130)/GFP-ul4(50-130).tif]

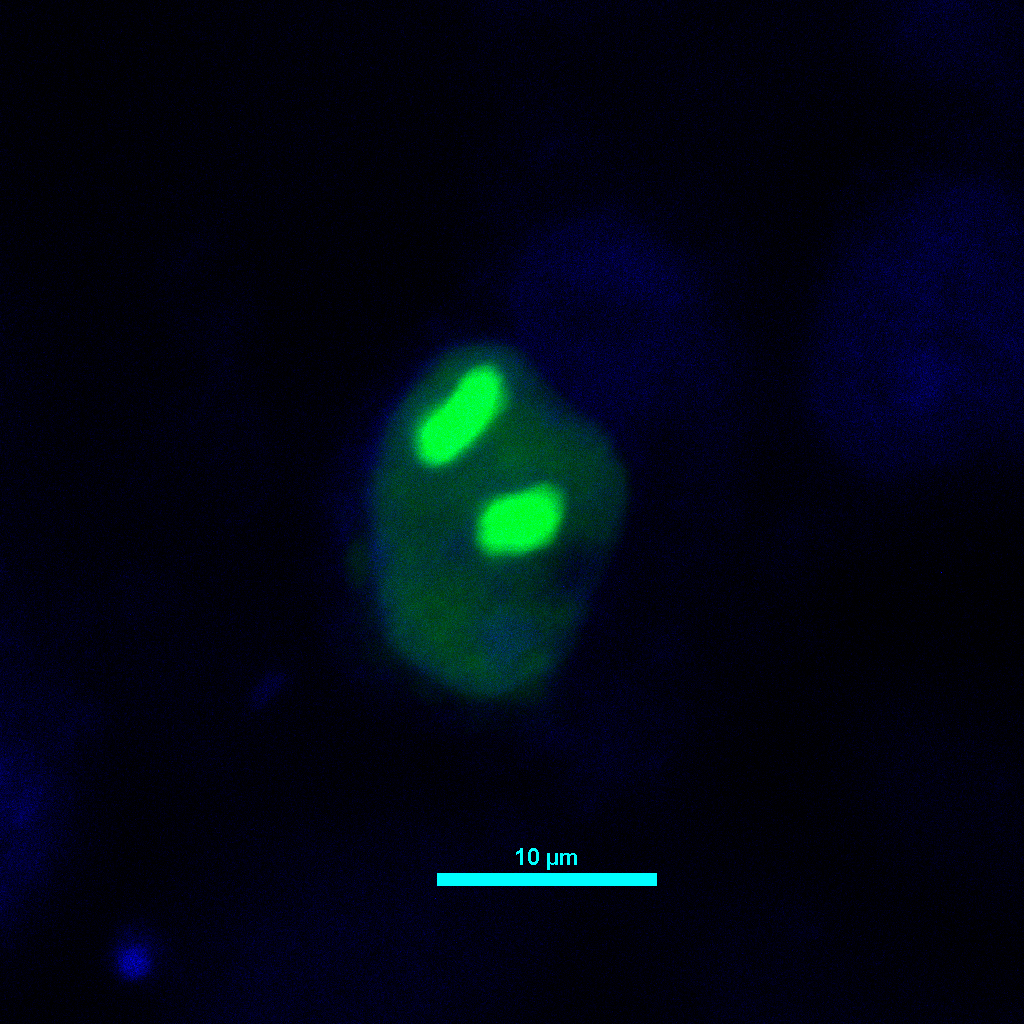

Supplement: S3 Data — (ZIP) [file ppat.1012546.s007.zip › Figure6D/1/GFP-UL4(50-130)/Merge.tif]

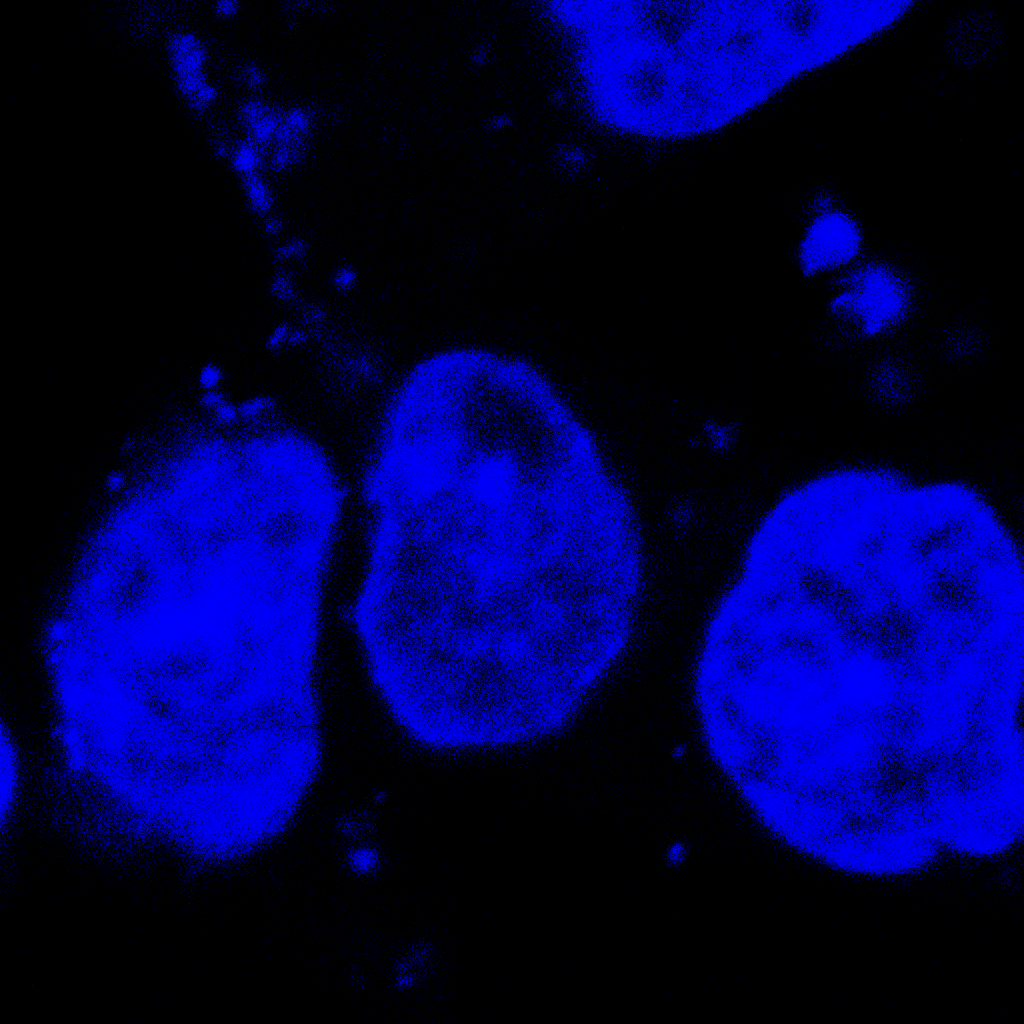

Supplement: S3 Data — (ZIP) [file ppat.1012546.s007.zip › Figure6D/1/GFP-UL4(73-146)/DAPI.tif]

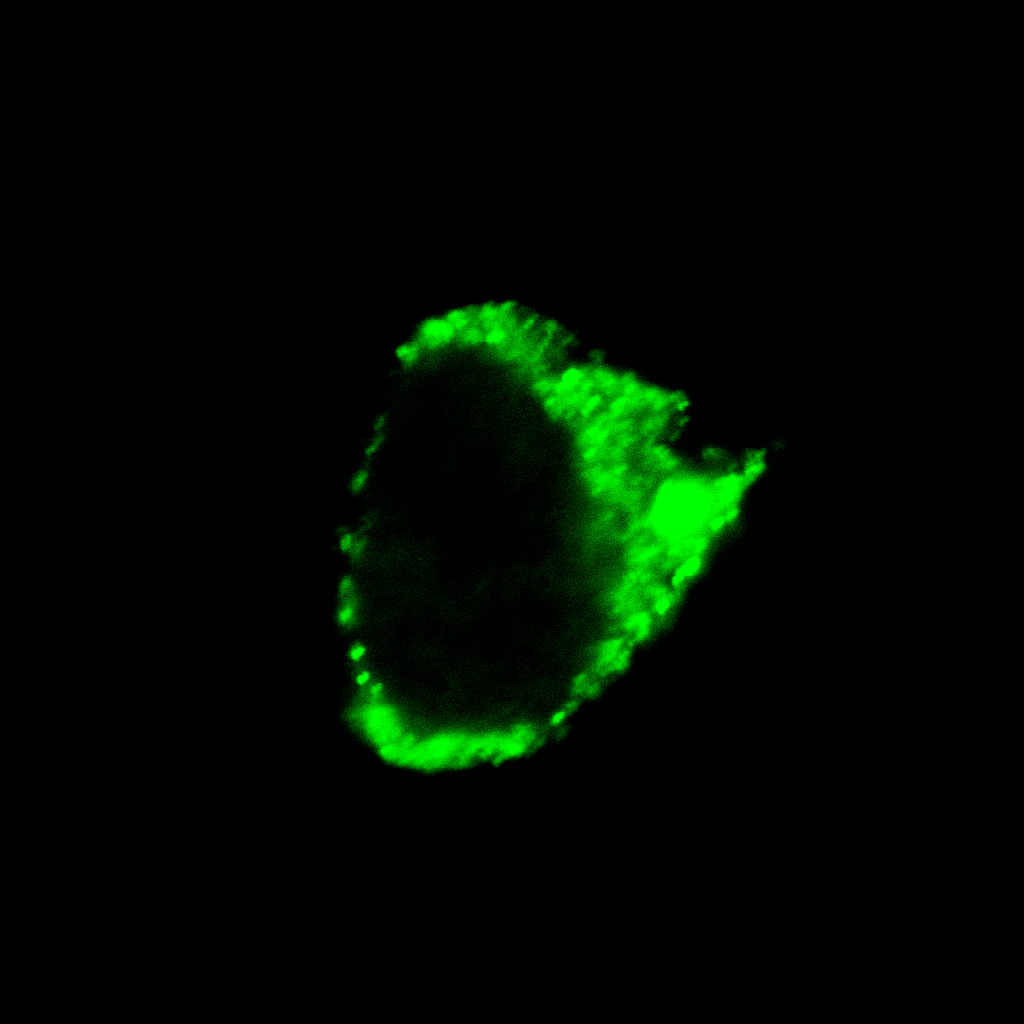

Supplement: S3 Data — (ZIP) [file ppat.1012546.s007.zip › Figure6D/1/GFP-UL4(73-146)/GFP-UL4(73-146).tif]

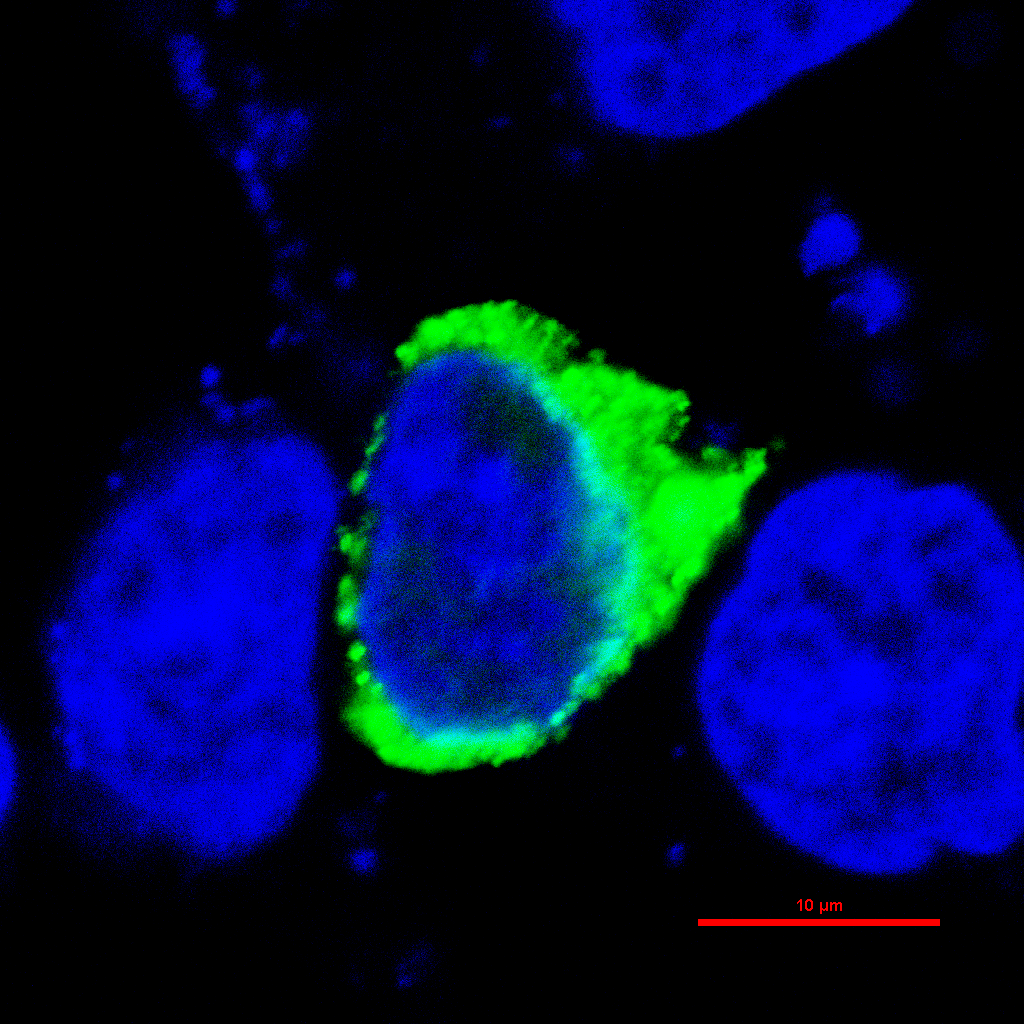

Supplement: S3 Data — (ZIP) [file ppat.1012546.s007.zip › Figure6D/1/GFP-UL4(73-146)/Merge.tif]

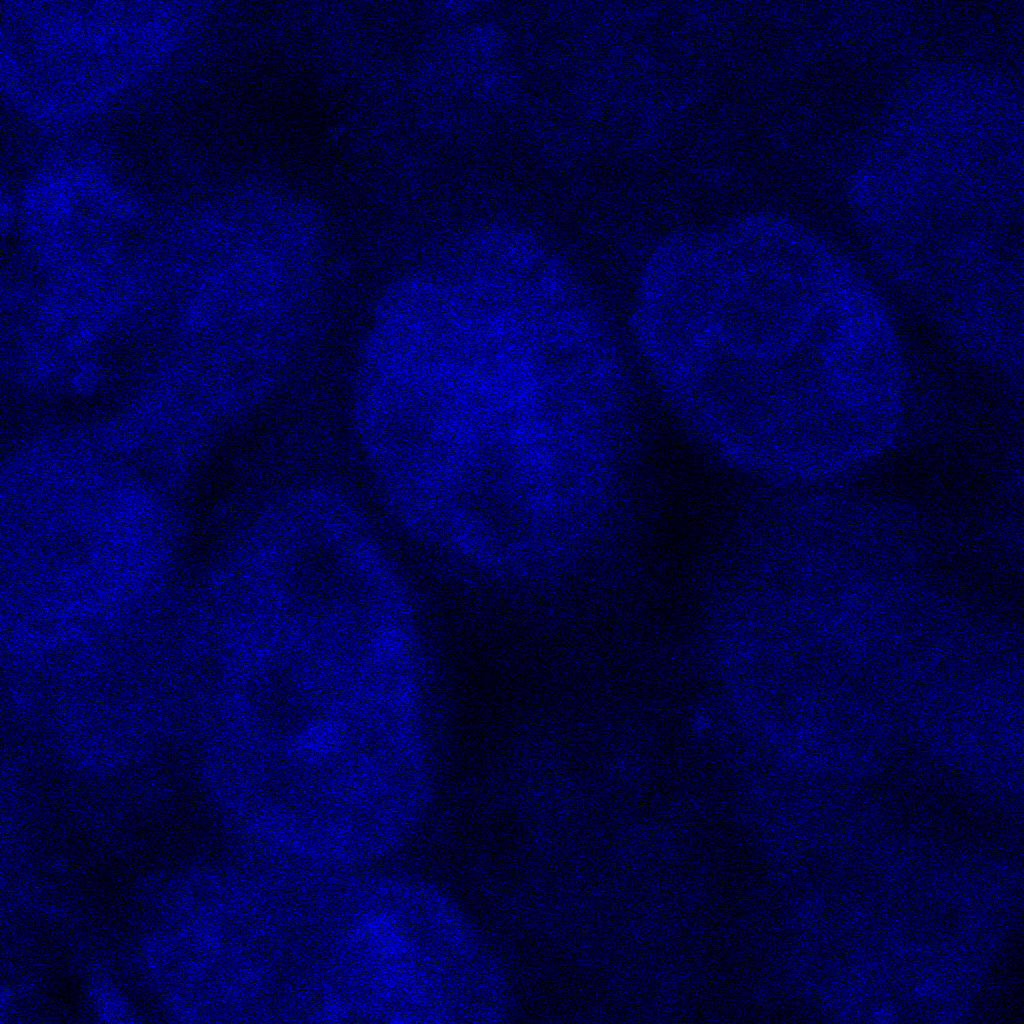

Supplement: S3 Data — (ZIP) [file ppat.1012546.s007.zip › Figure6D/2/flag-ASC+GFP-UL4(1-73)/DAPI.tif]

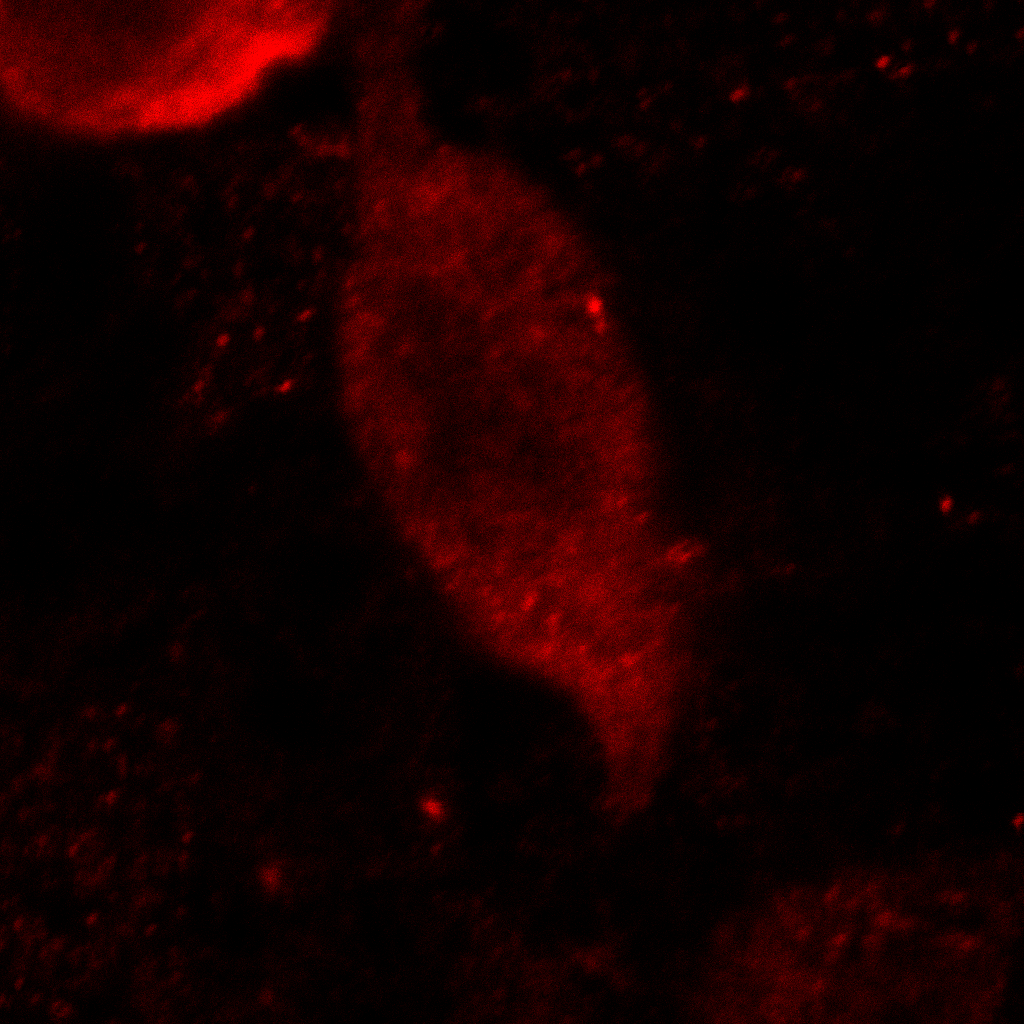

Supplement: S3 Data — (ZIP) [file ppat.1012546.s007.zip › Figure6D/2/flag-ASC+GFP-UL4(1-73)/flag-asc.tif]

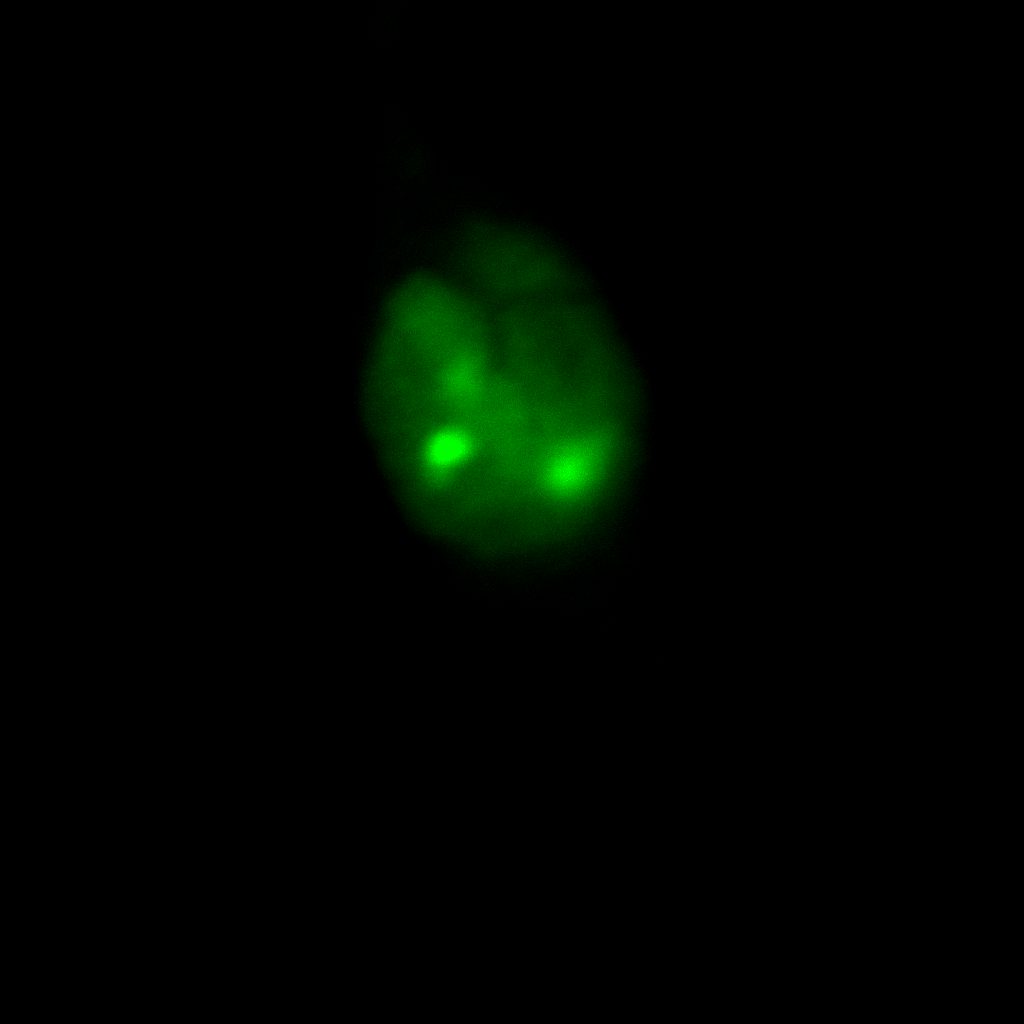

Supplement: S3 Data — (ZIP) [file ppat.1012546.s007.zip › Figure6D/2/flag-ASC+GFP-UL4(1-73)/gfp-ul4 (1-73ú⌐.tif]

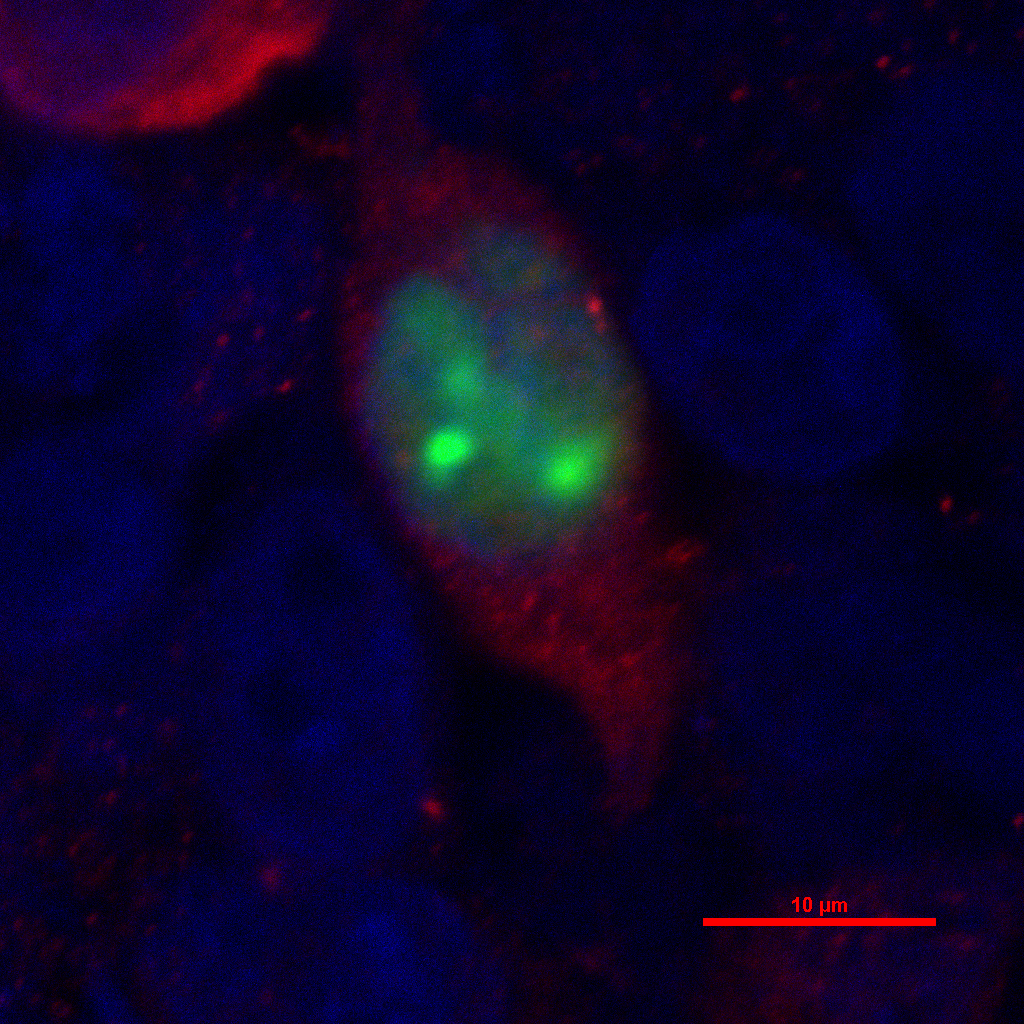

Supplement: S3 Data — (ZIP) [file ppat.1012546.s007.zip › Figure6D/2/flag-ASC+GFP-UL4(1-73)/Merge.tif]

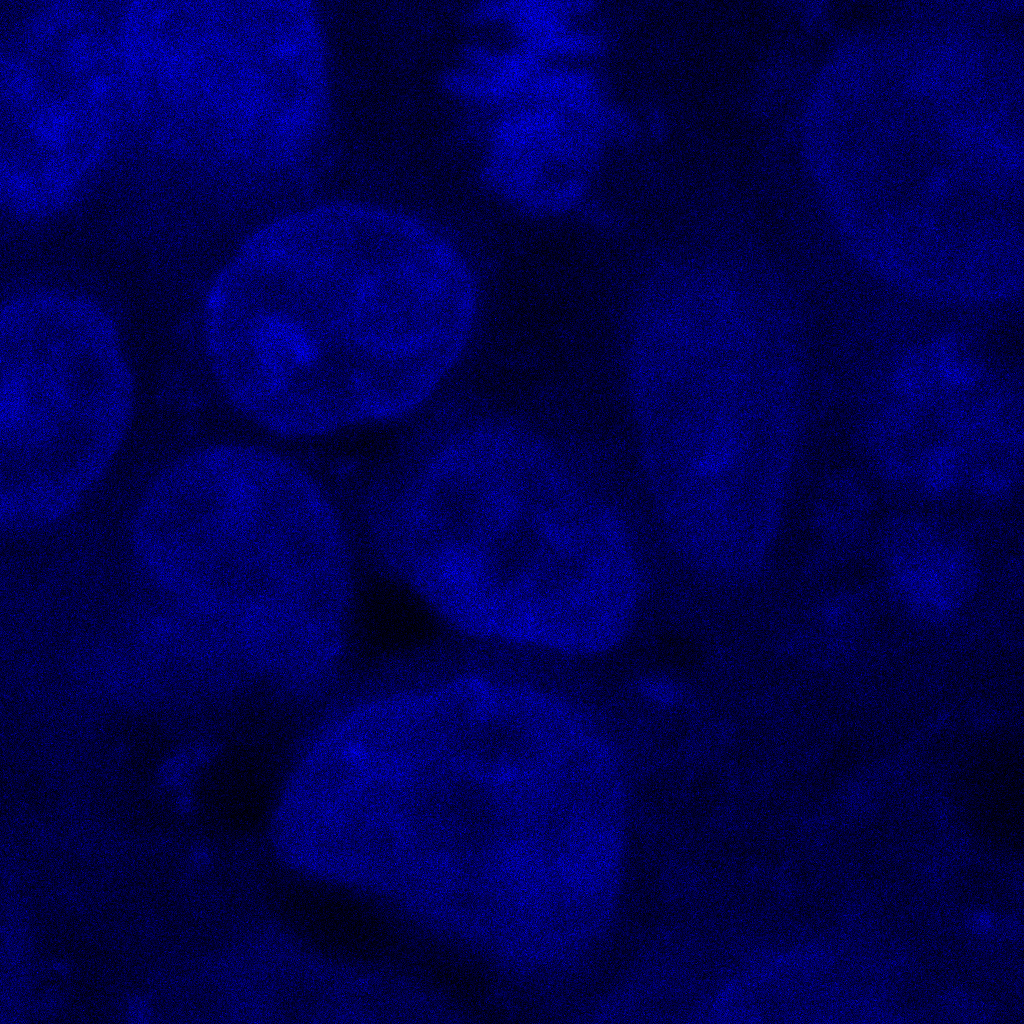

Supplement: S3 Data — (ZIP) [file ppat.1012546.s007.zip › Figure6D/2/flag-ASC+GFP-UL4(50-130)/DAPI.tif]

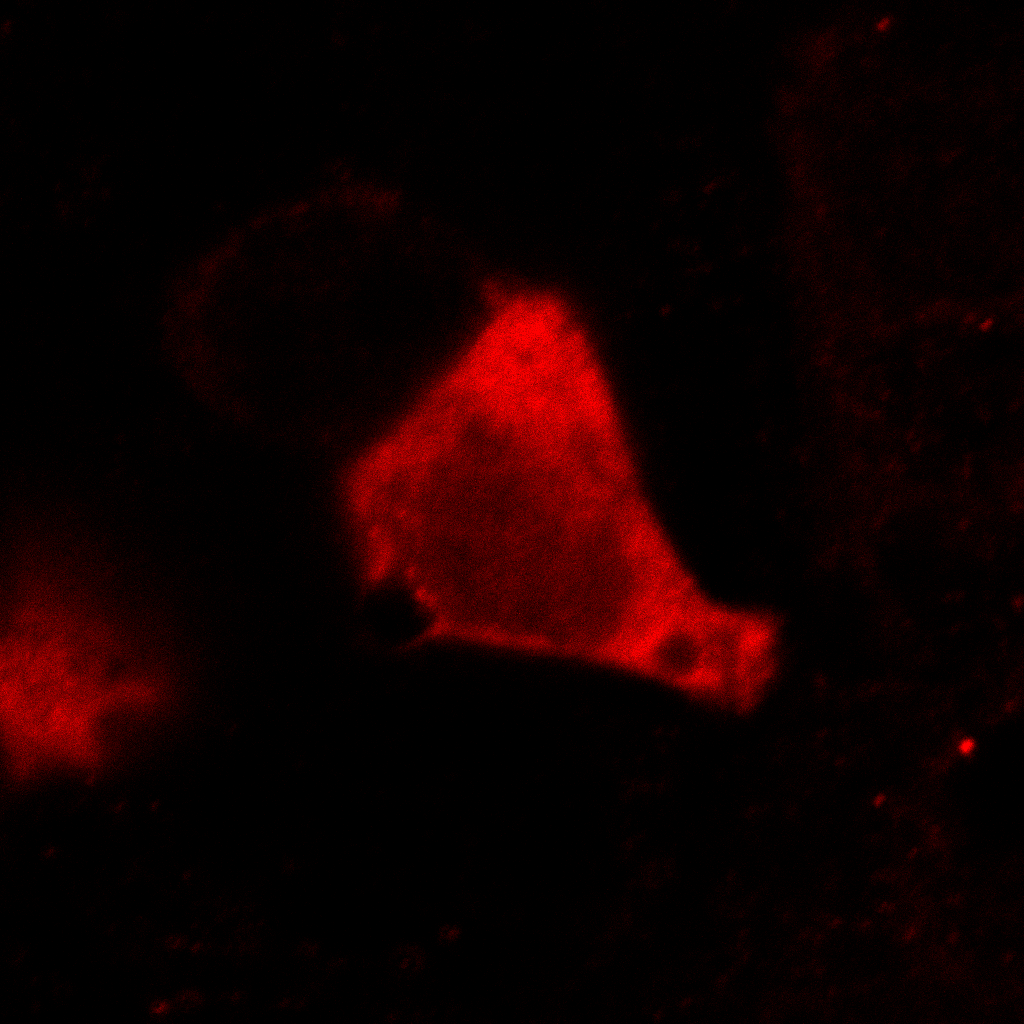

Supplement: S3 Data — (ZIP) [file ppat.1012546.s007.zip › Figure6D/2/flag-ASC+GFP-UL4(50-130)/Flag-asc.tif]

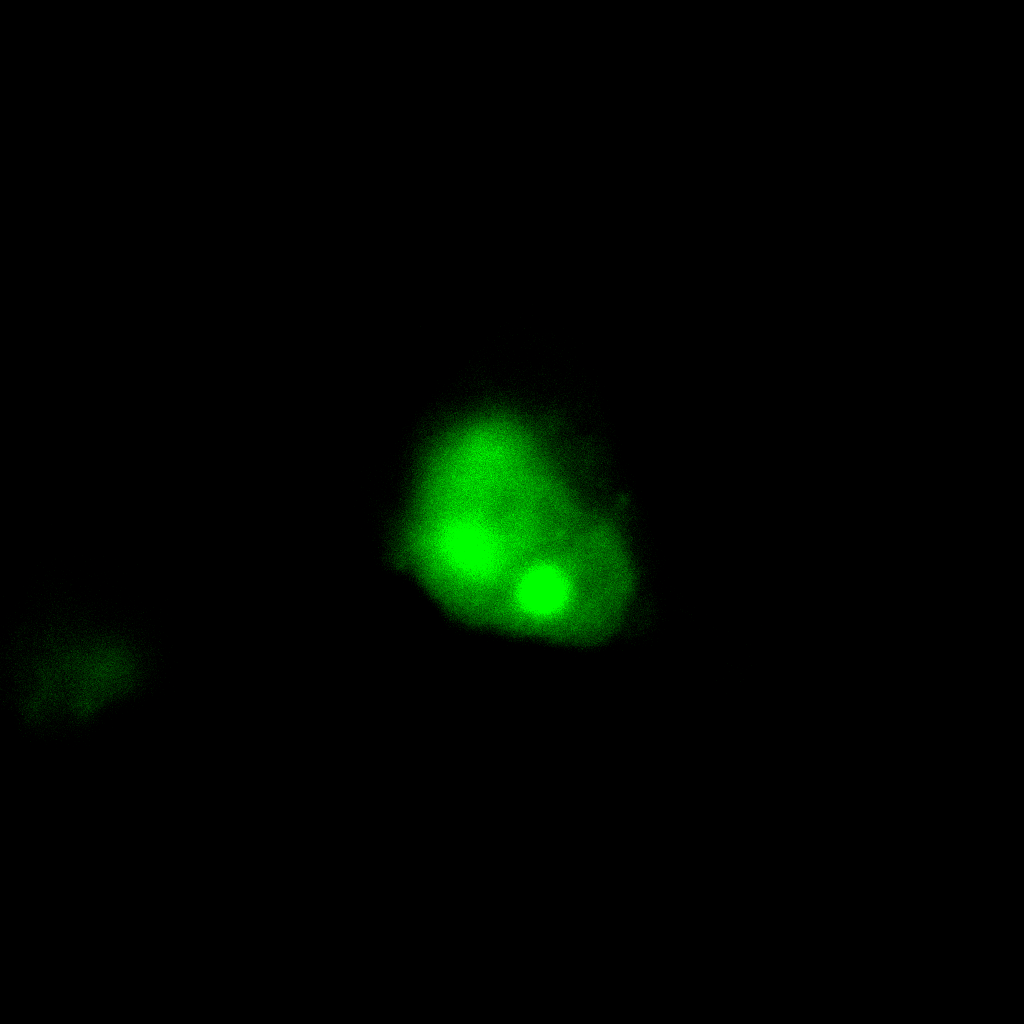

Supplement: S3 Data — (ZIP) [file ppat.1012546.s007.zip › Figure6D/2/flag-ASC+GFP-UL4(50-130)/gfp-ul4 (50-130).tif]

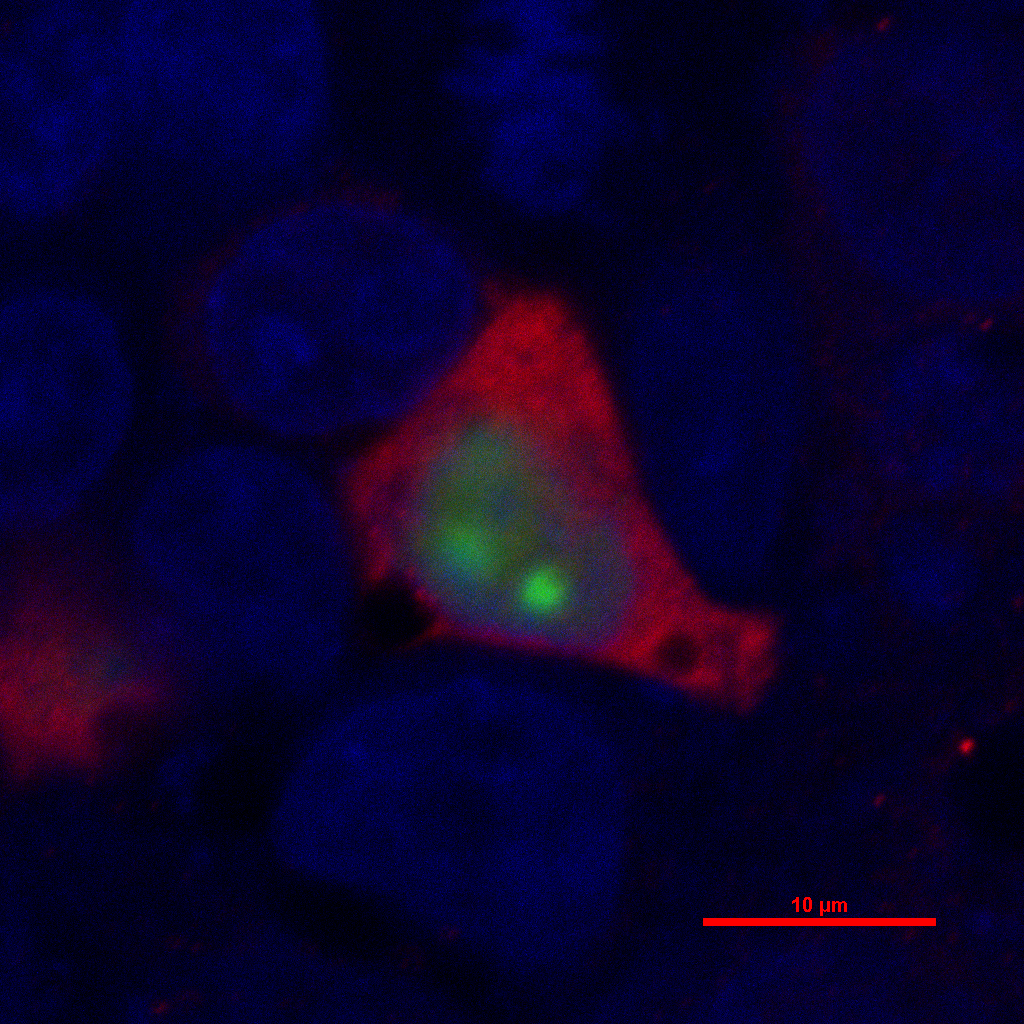

Supplement: S3 Data — (ZIP) [file ppat.1012546.s007.zip › Figure6D/2/flag-ASC+GFP-UL4(50-130)/Merge.tif]

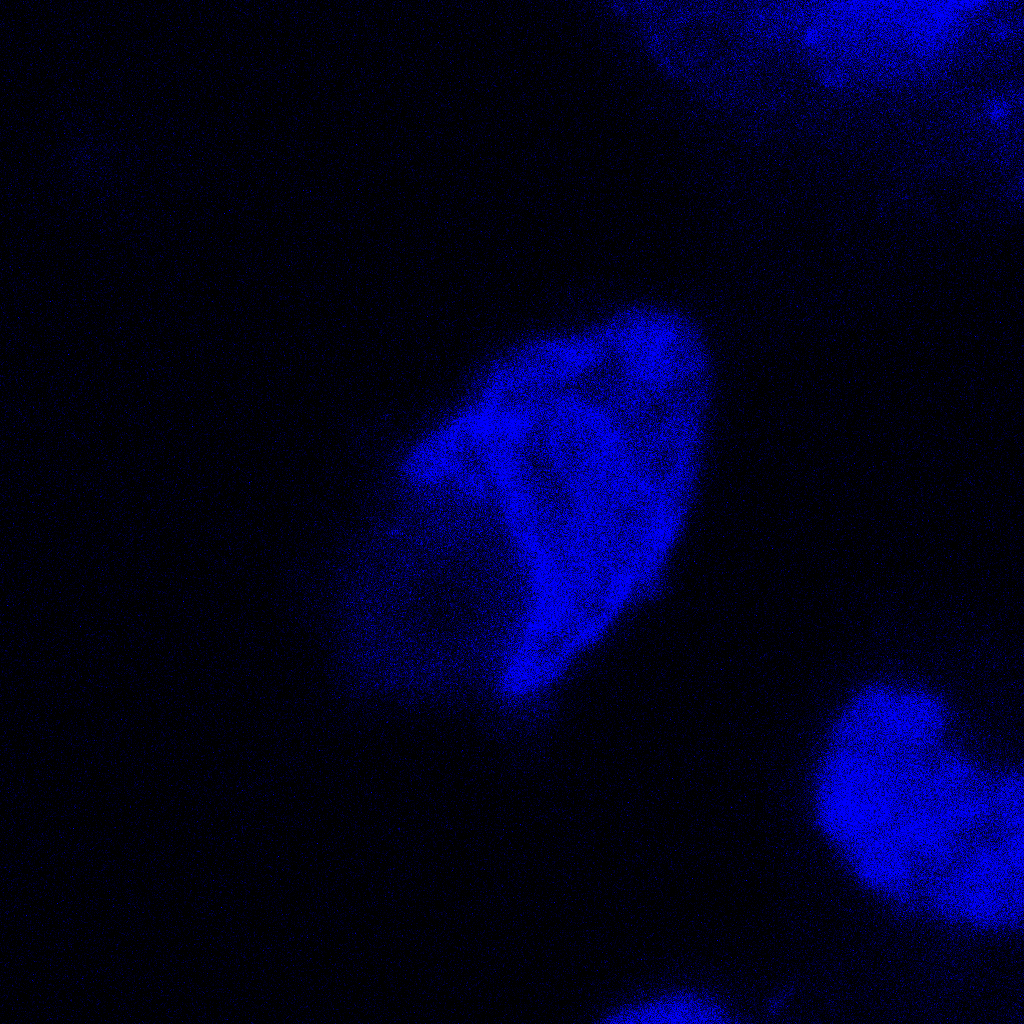

Supplement: S3 Data — (ZIP) [file ppat.1012546.s007.zip › Figure6D/2/flag-ASC+GFP-UL4(73-146)/DAPI.tif]

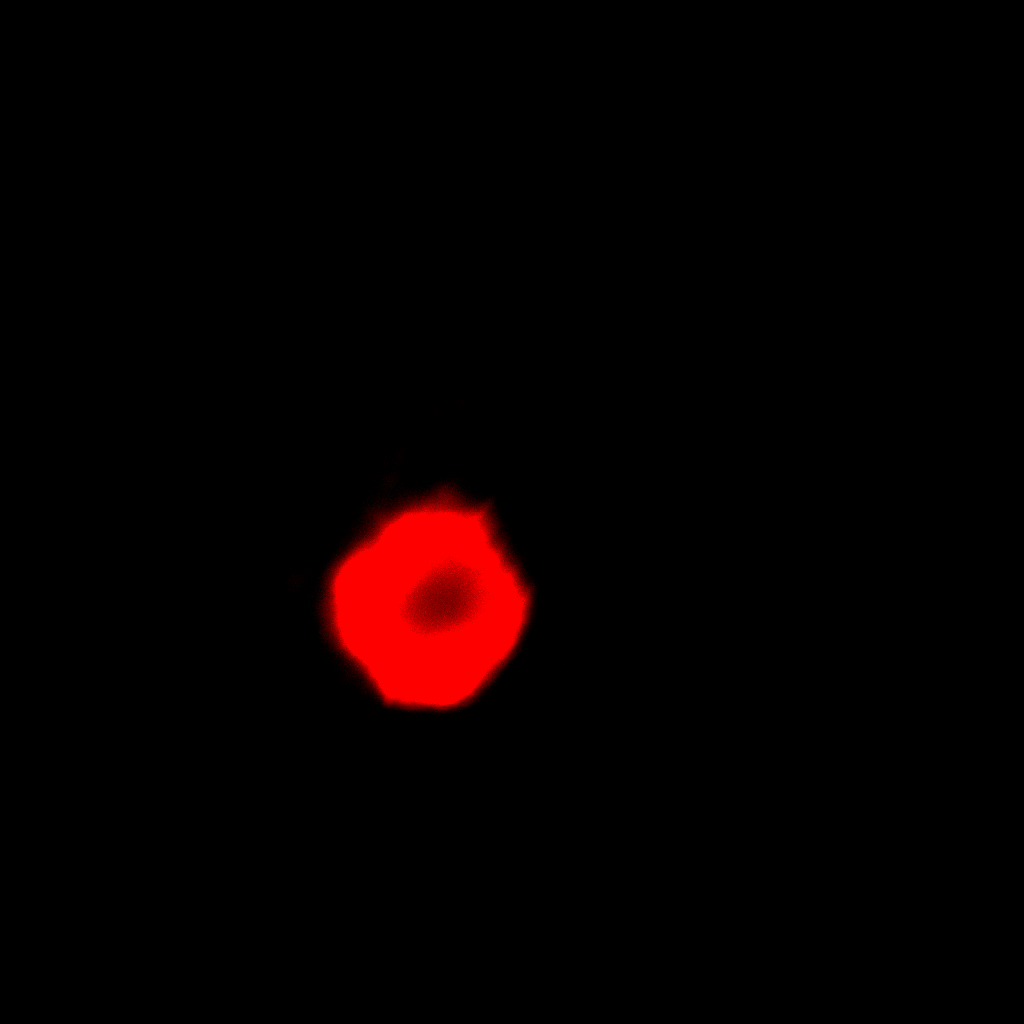

Supplement: S3 Data — (ZIP) [file ppat.1012546.s007.zip › Figure6D/2/flag-ASC+GFP-UL4(73-146)/flag-asc.tif]

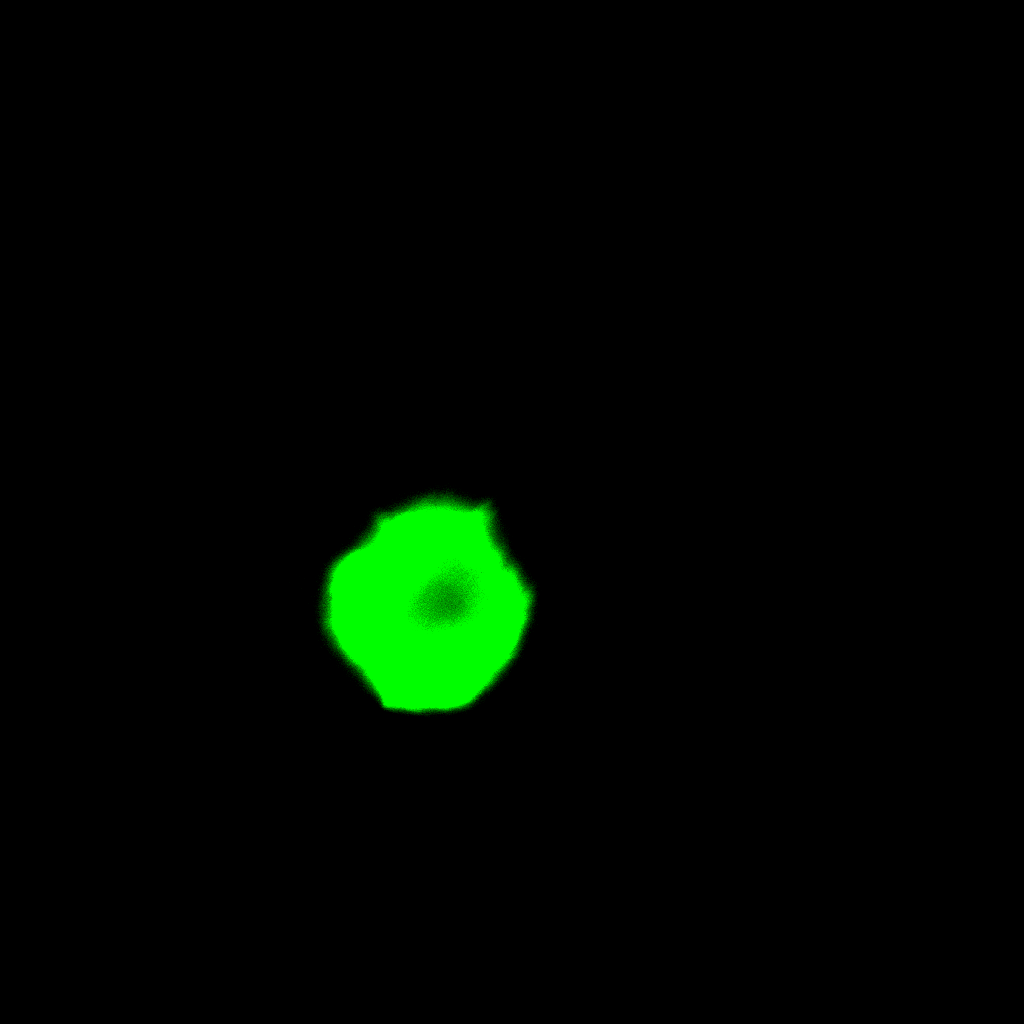

Supplement: S3 Data — (ZIP) [file ppat.1012546.s007.zip › Figure6D/2/flag-ASC+GFP-UL4(73-146)/gfp-ul4(73-146).tif]

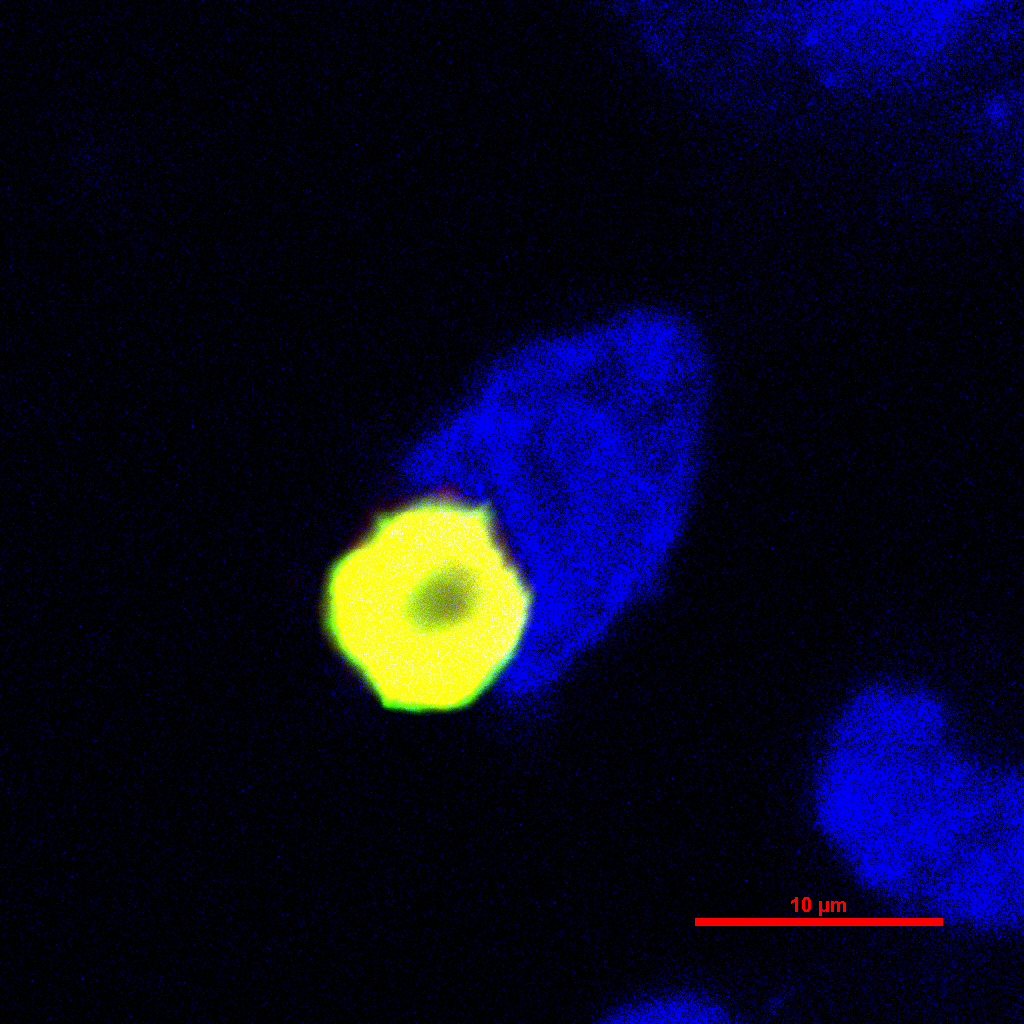

Supplement: S3 Data — (ZIP) [file ppat.1012546.s007.zip › Figure6D/2/flag-ASC+GFP-UL4(73-146)/Merge.tif]

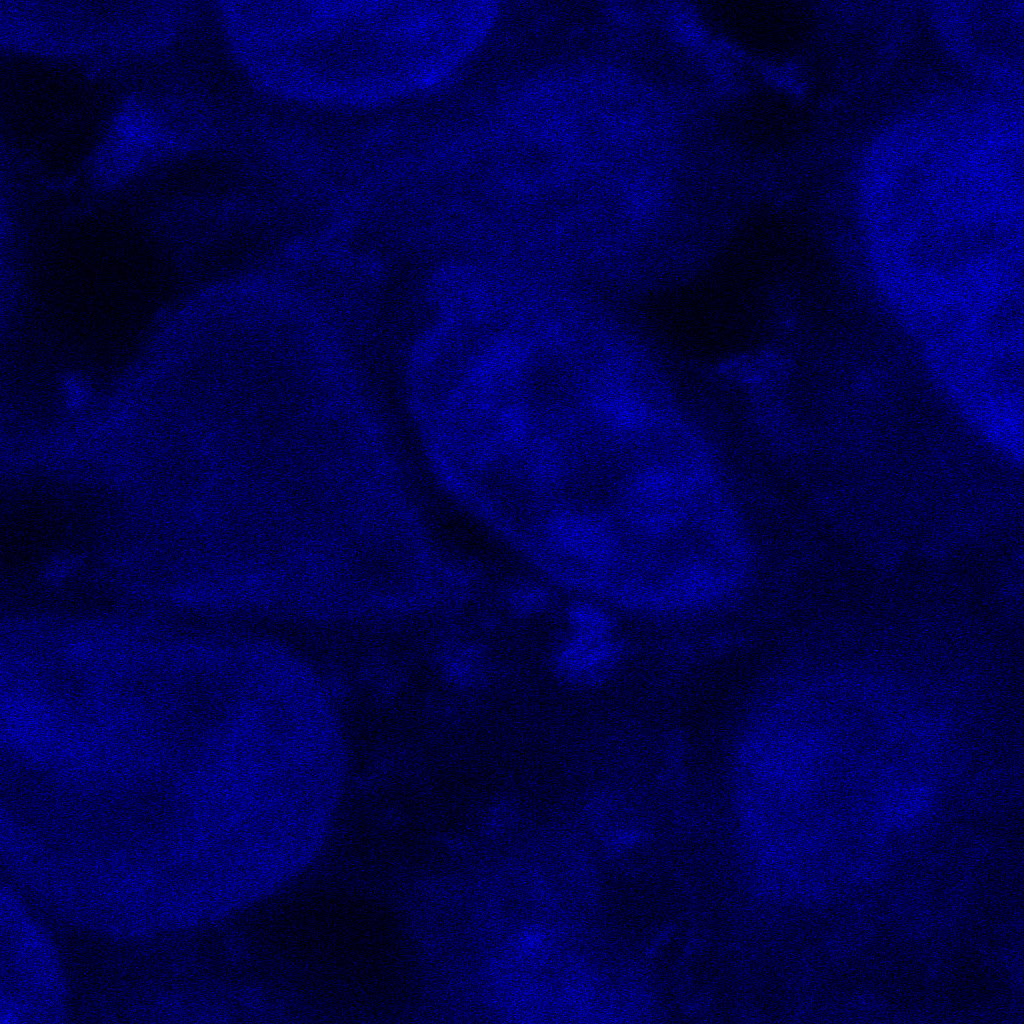

Supplement: S3 Data — (ZIP) [file ppat.1012546.s007.zip › Figure6D/2/GFP-UL4(1-73)/DAPI.tif]

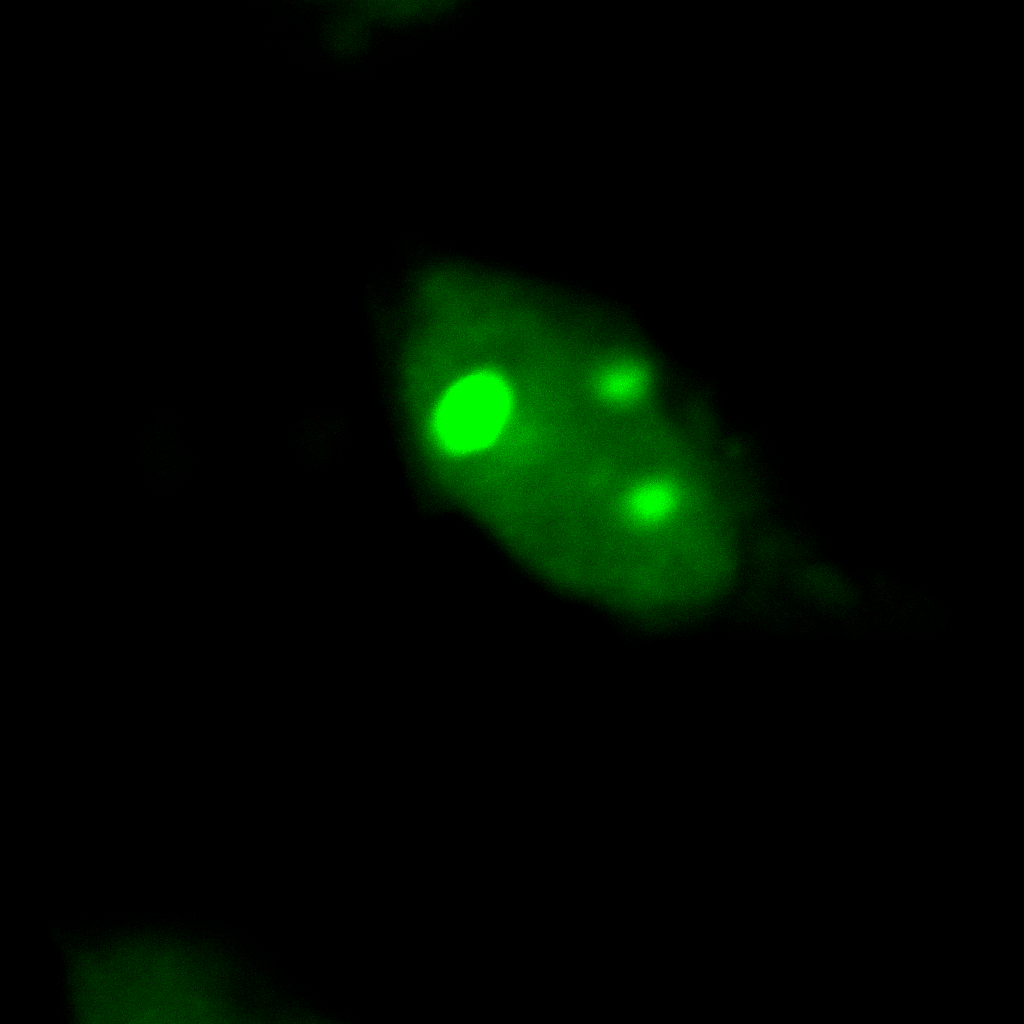

Supplement: S3 Data — (ZIP) [file ppat.1012546.s007.zip › Figure6D/2/GFP-UL4(1-73)/GFP-UL4(1-73).tif]

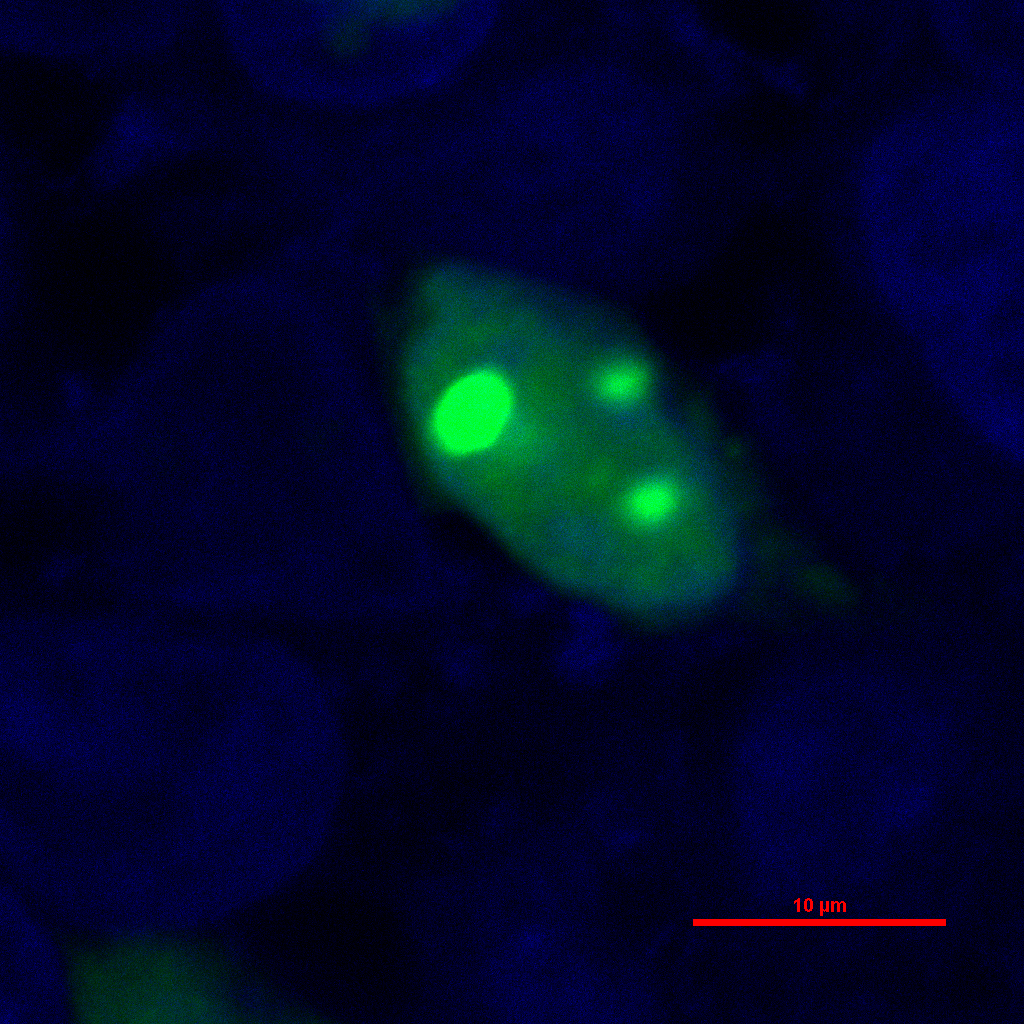

Supplement: S3 Data — (ZIP) [file ppat.1012546.s007.zip › Figure6D/2/GFP-UL4(1-73)/Merge.tif]

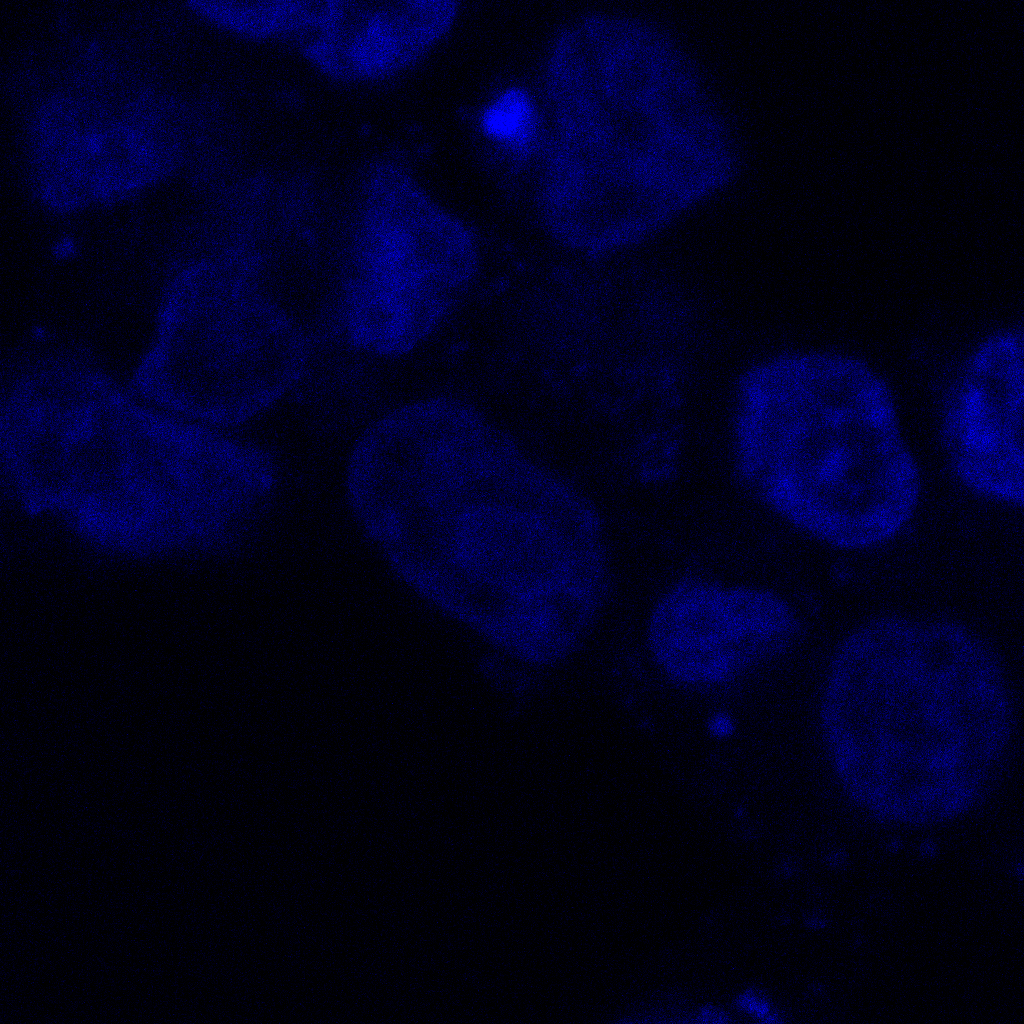

Supplement: S3 Data — (ZIP) [file ppat.1012546.s007.zip › Figure6D/2/GFP-UL4(50-130)/DAPI.tif]

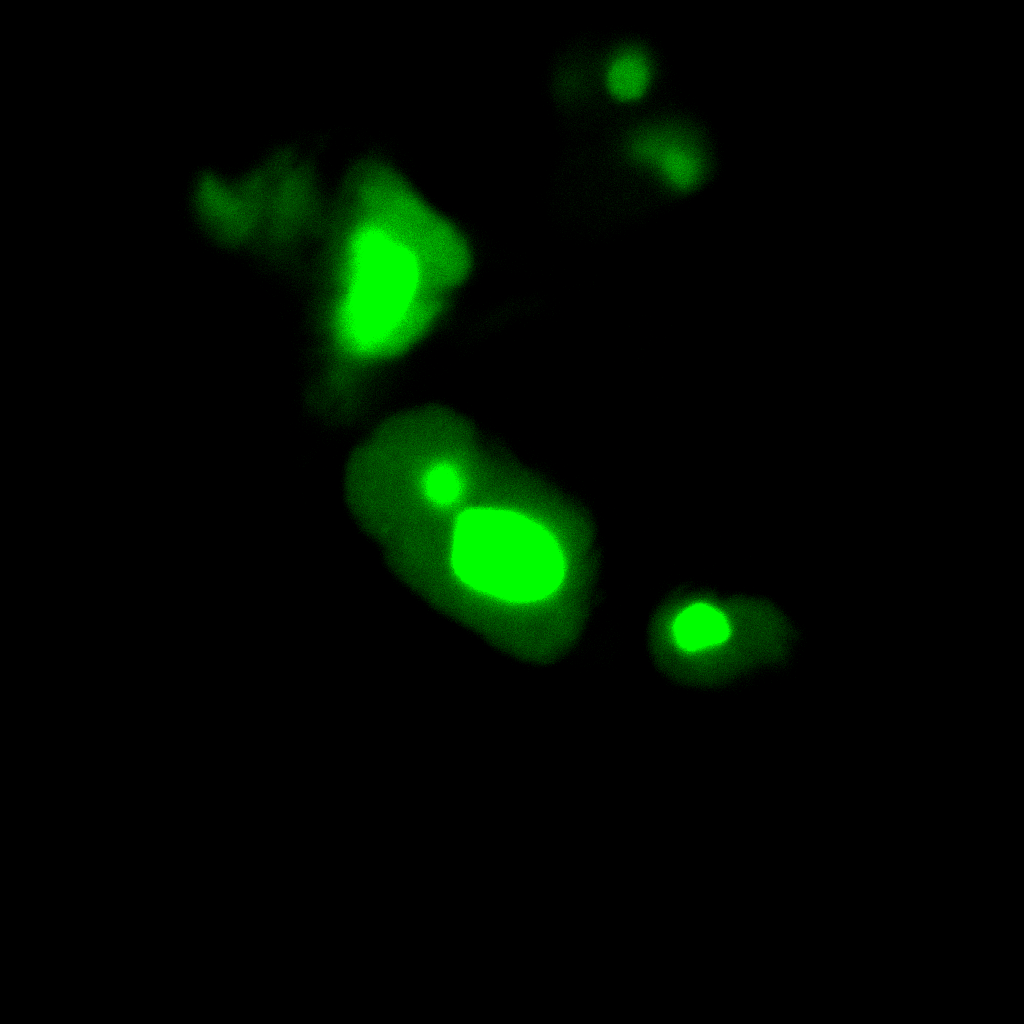

Supplement: S3 Data — (ZIP) [file ppat.1012546.s007.zip › Figure6D/2/GFP-UL4(50-130)/gfp-ul4(50-130).tif]

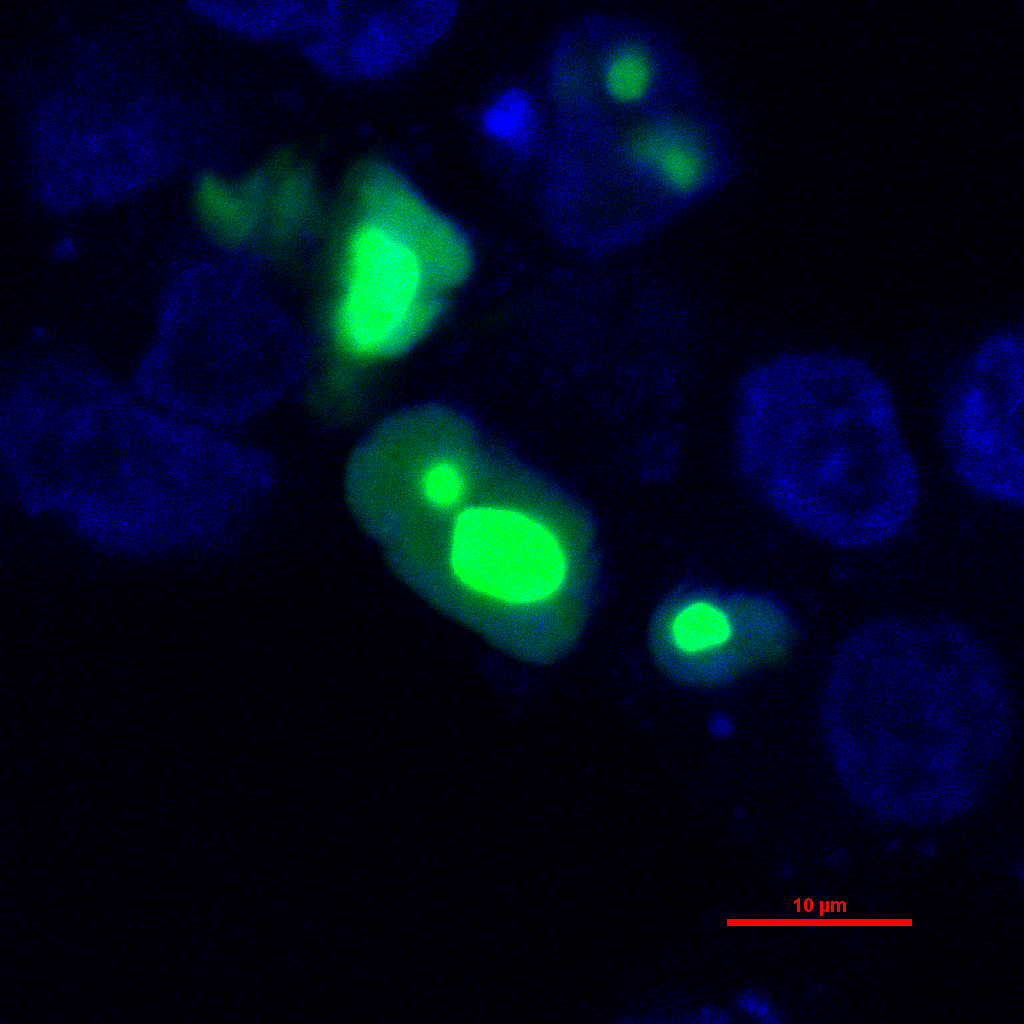

Supplement: S3 Data — (ZIP) [file ppat.1012546.s007.zip › Figure6D/2/GFP-UL4(50-130)/Merge.tif]

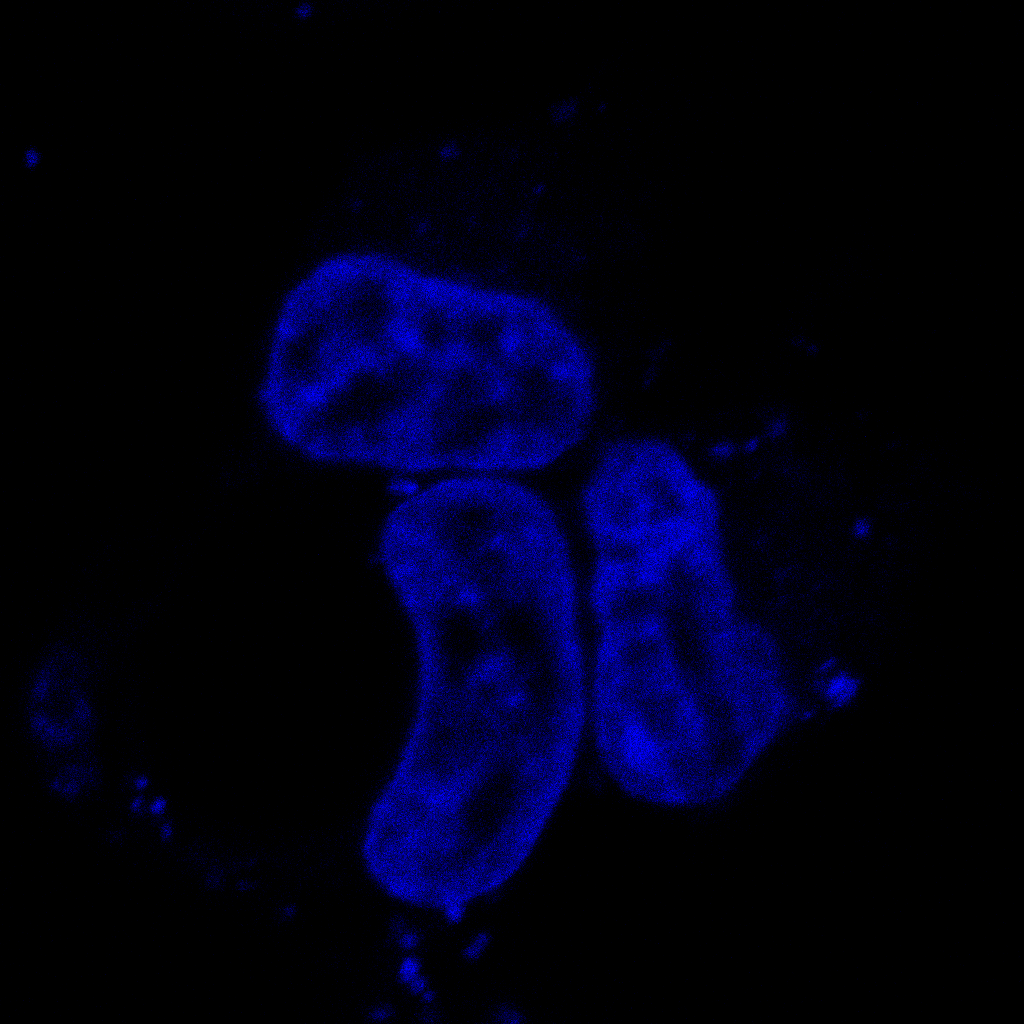

Supplement: S3 Data — (ZIP) [file ppat.1012546.s007.zip › Figure6D/2/GFP-UL4(73-146)/DAPI.tif]

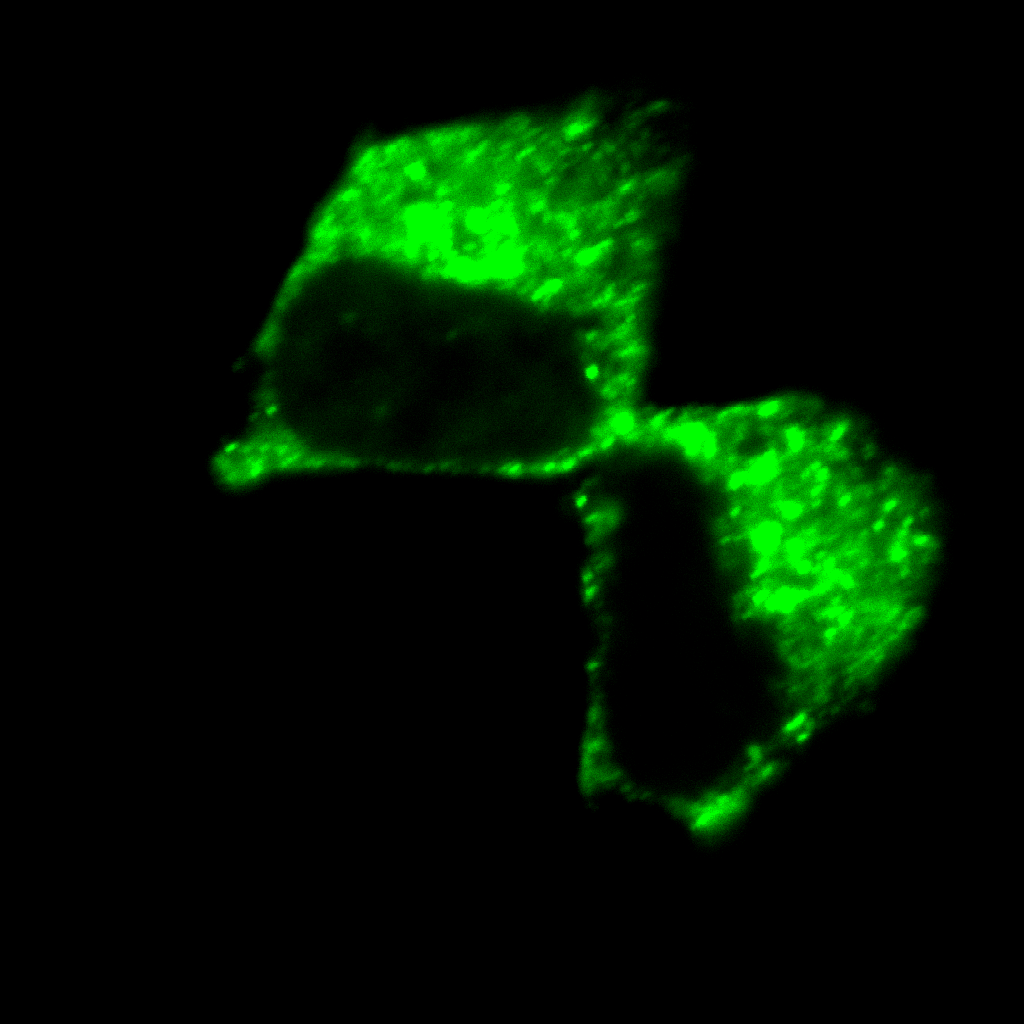

Supplement: S3 Data — (ZIP) [file ppat.1012546.s007.zip › Figure6D/2/GFP-UL4(73-146)/gfp-ul4(73-146).tif]

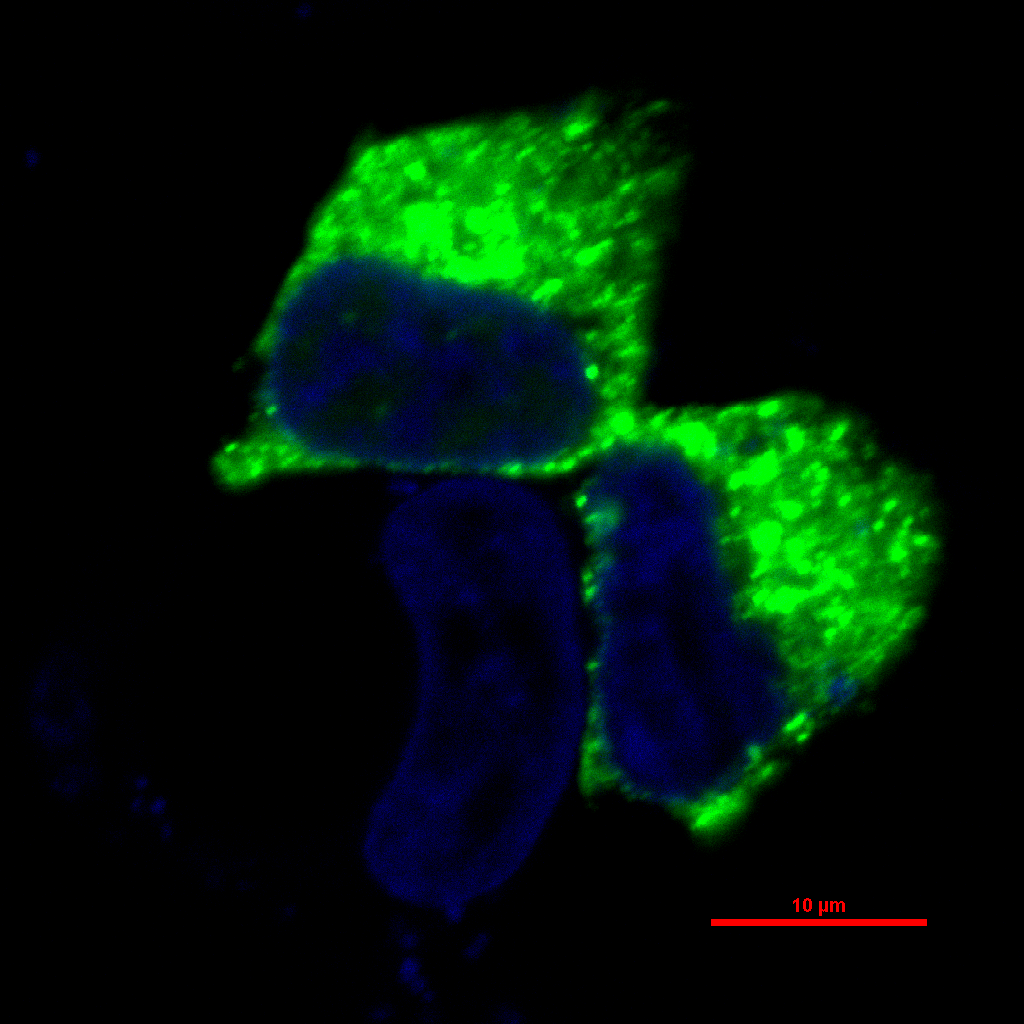

Supplement: S3 Data — (ZIP) [file ppat.1012546.s007.zip › Figure6D/2/GFP-UL4(73-146)/Merge.tif]

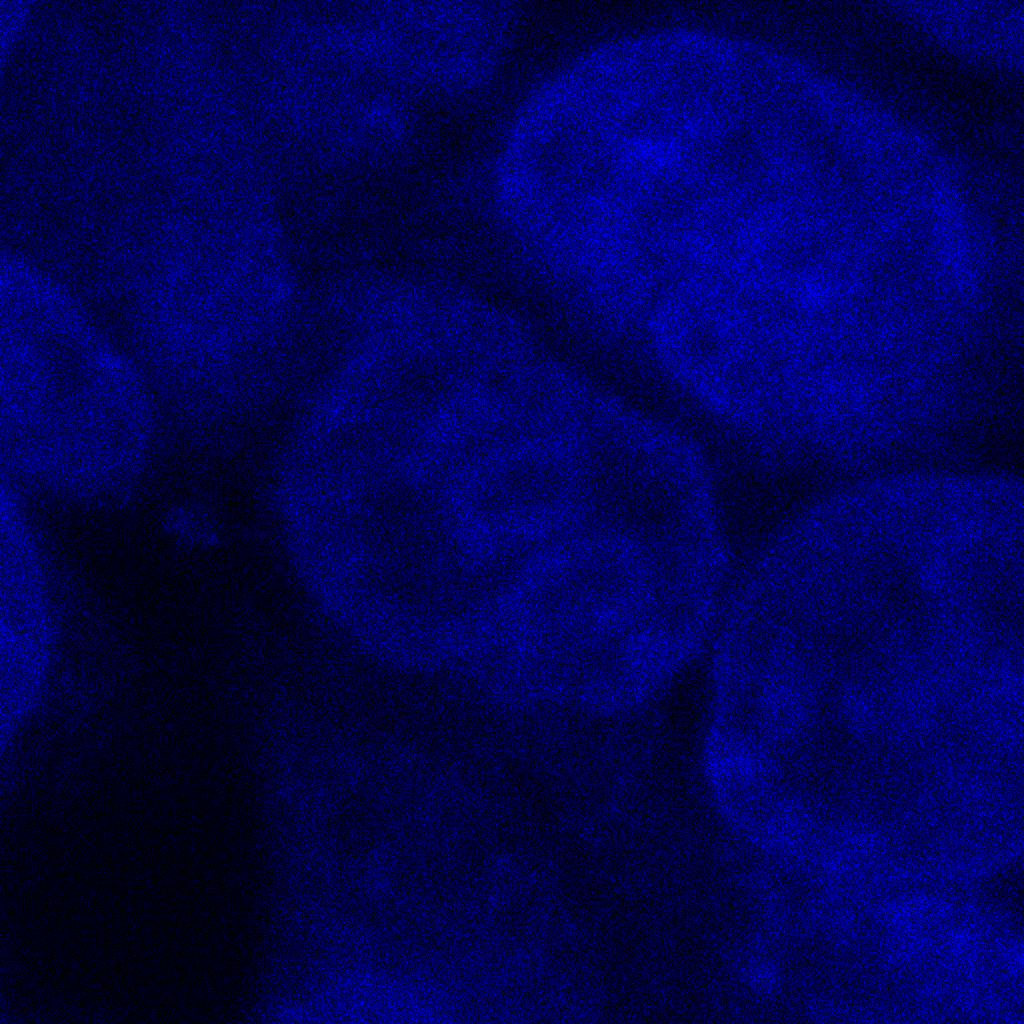

Supplement: S3 Data — (ZIP) [file ppat.1012546.s007.zip › Figure6D/3/flag-ASC+GFP-UL4(1-73)/DAPI.tif]

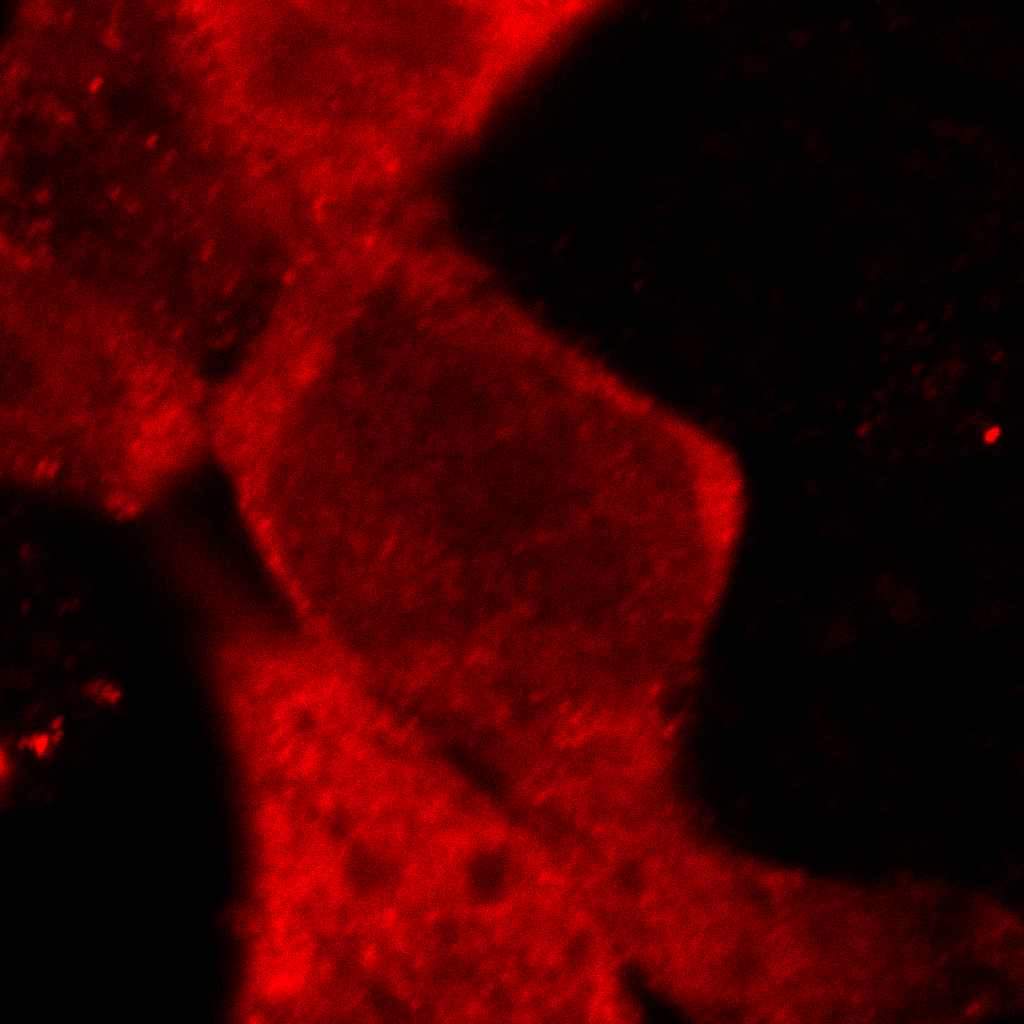

Supplement: S3 Data — (ZIP) [file ppat.1012546.s007.zip › Figure6D/3/flag-ASC+GFP-UL4(1-73)/flag-asc.tif]

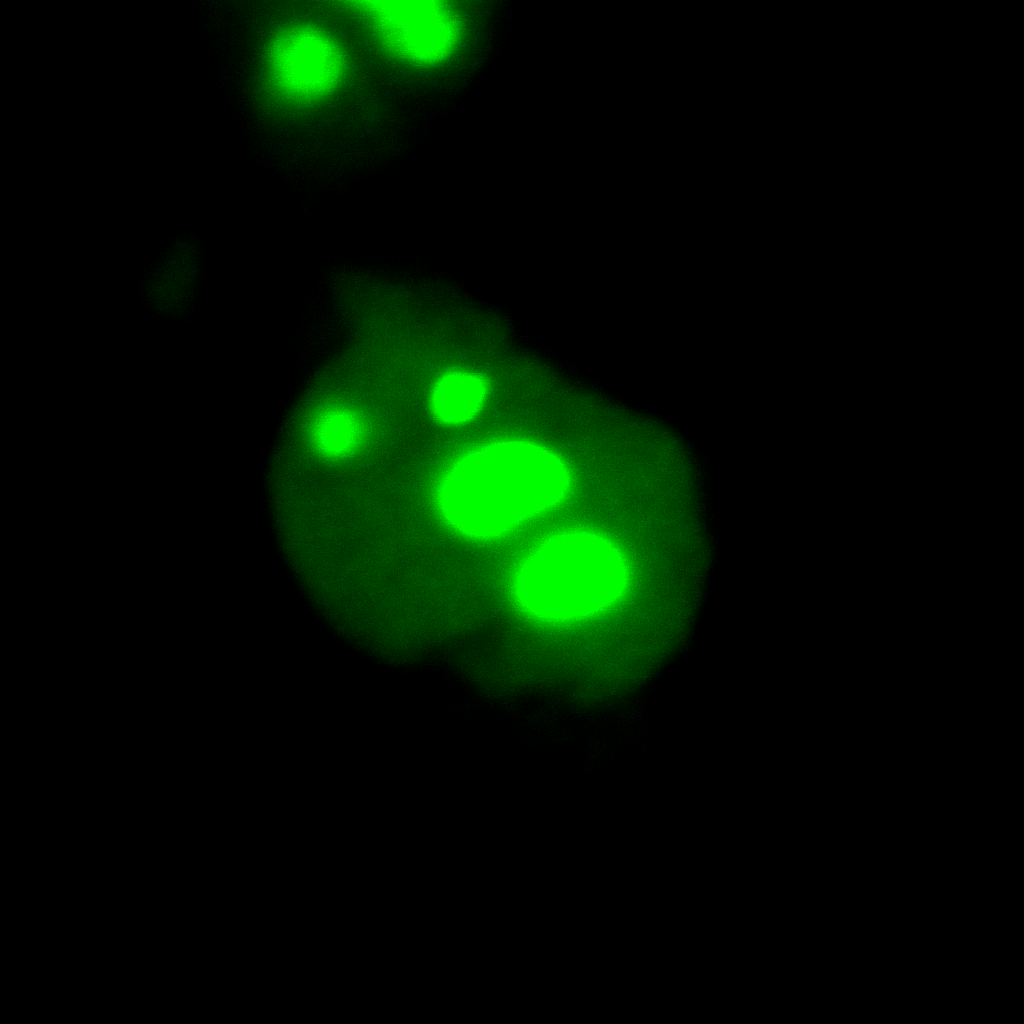

Supplement: S3 Data — (ZIP) [file ppat.1012546.s007.zip › Figure6D/3/flag-ASC+GFP-UL4(1-73)/gfp-ul4 ú¿1-73ú⌐.tif]

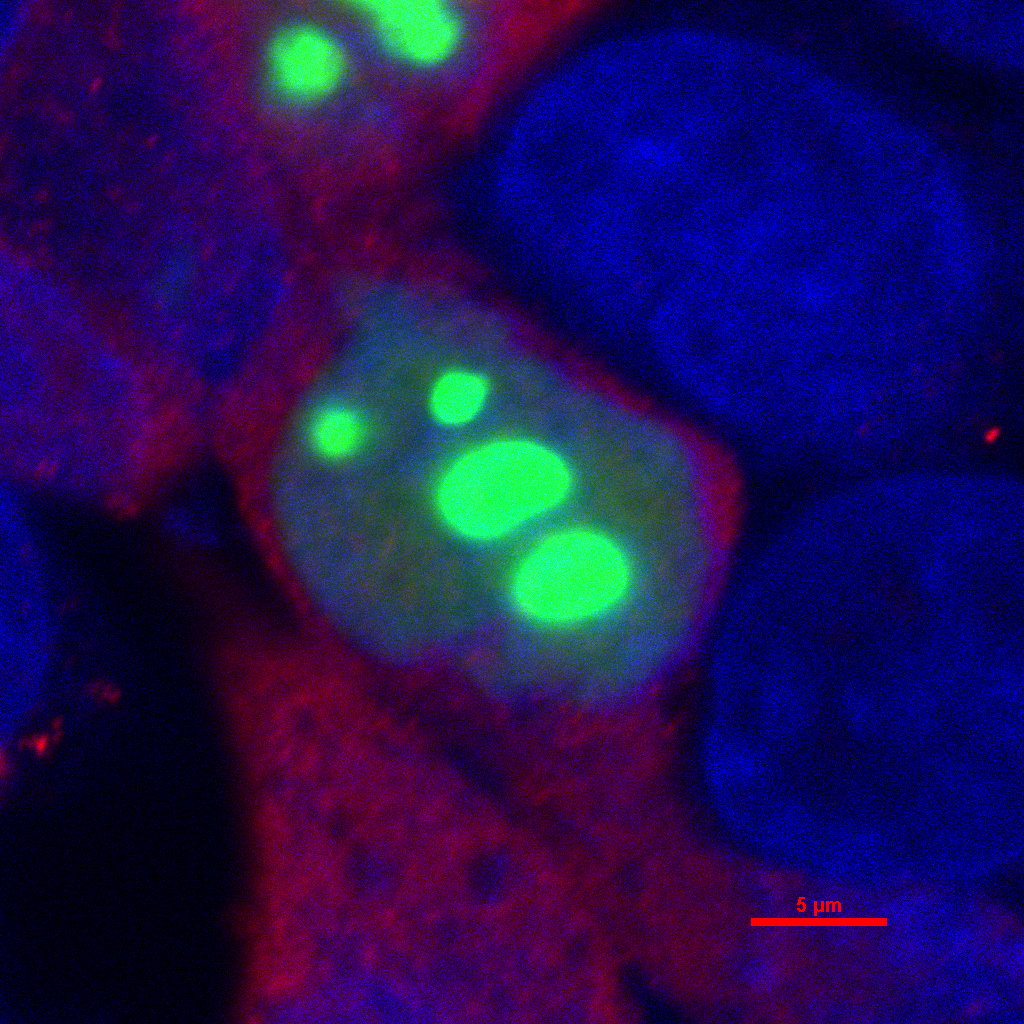

Supplement: S3 Data — (ZIP) [file ppat.1012546.s007.zip › Figure6D/3/flag-ASC+GFP-UL4(1-73)/Merge.tif]

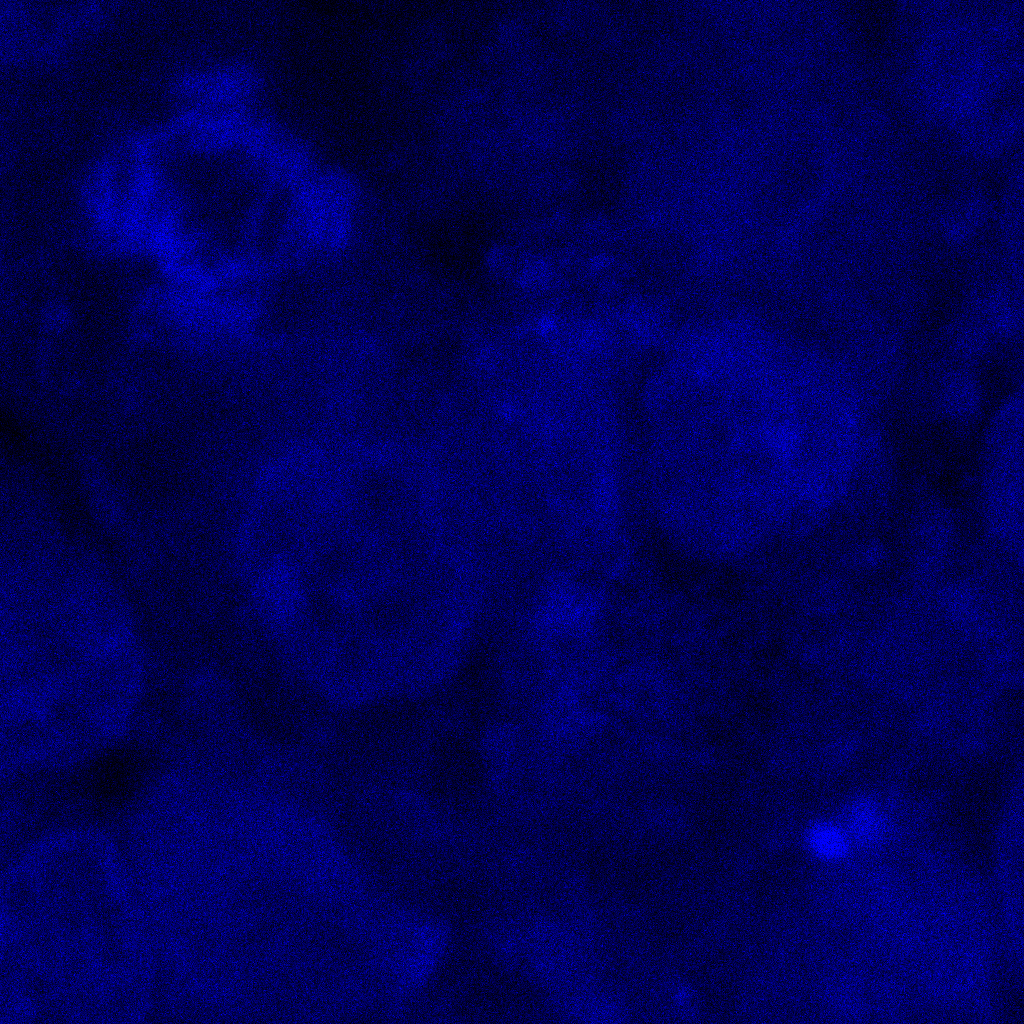

Supplement: S3 Data — (ZIP) [file ppat.1012546.s007.zip › Figure6D/3/flag-ASC+GFP-UL4(50-130)/DAPI.tif]

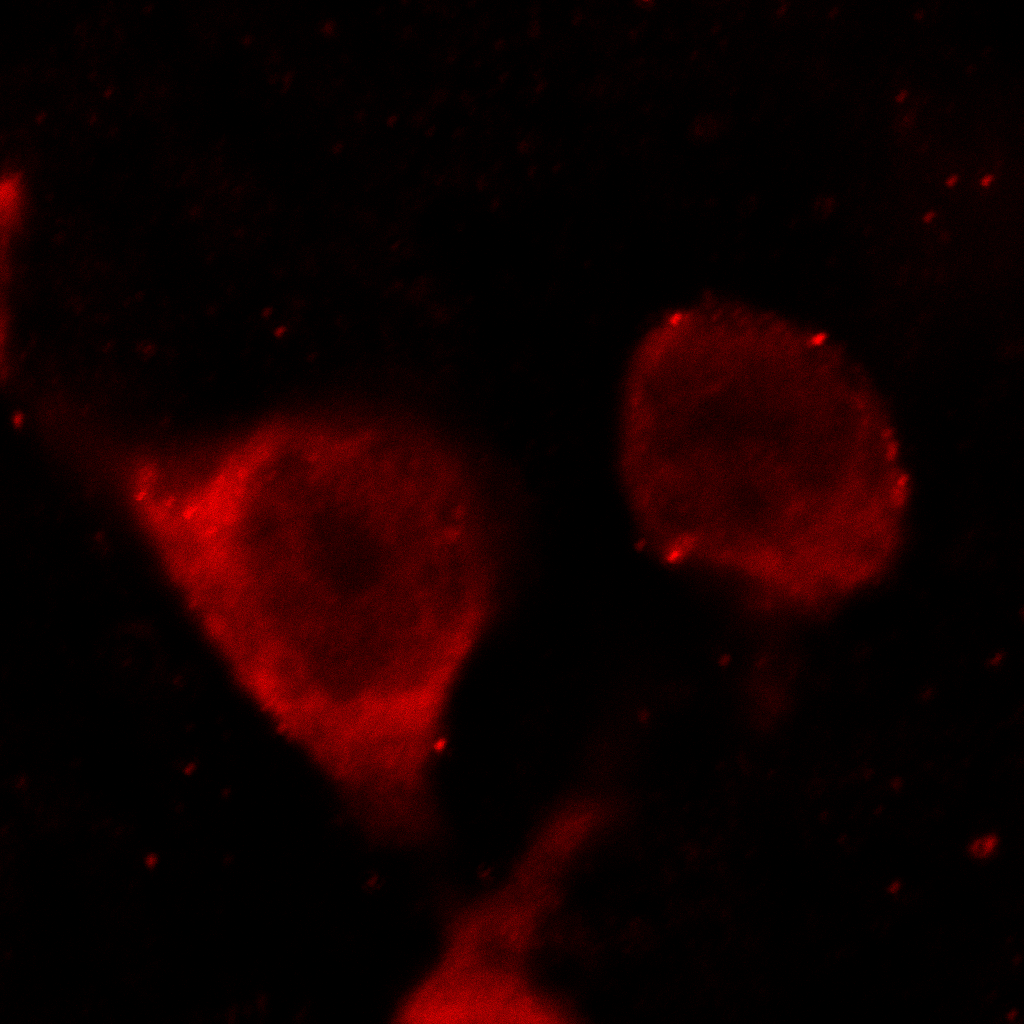

Supplement: S3 Data — (ZIP) [file ppat.1012546.s007.zip › Figure6D/3/flag-ASC+GFP-UL4(50-130)/flag-asc.tif]

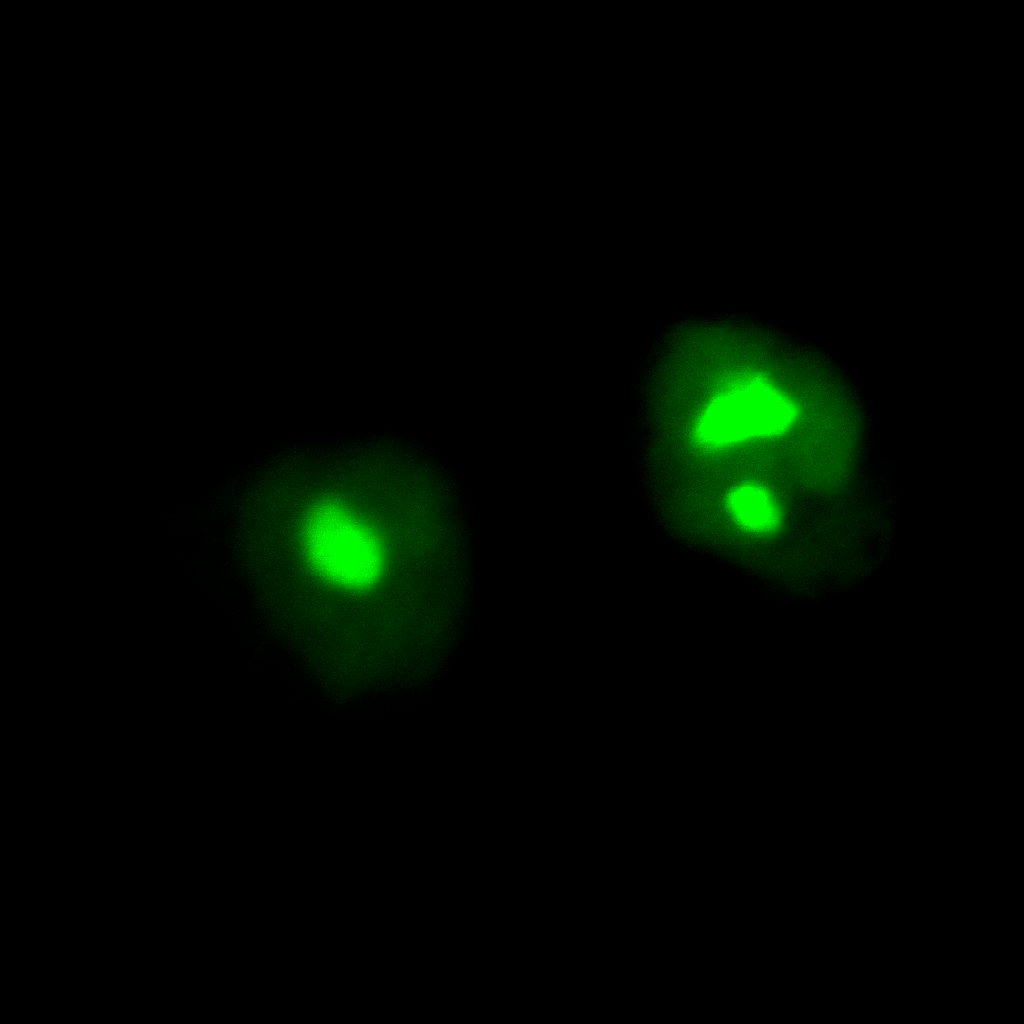

Supplement: S3 Data — (ZIP) [file ppat.1012546.s007.zip › Figure6D/3/flag-ASC+GFP-UL4(50-130)/gfp-ul4 (50-130).tif]

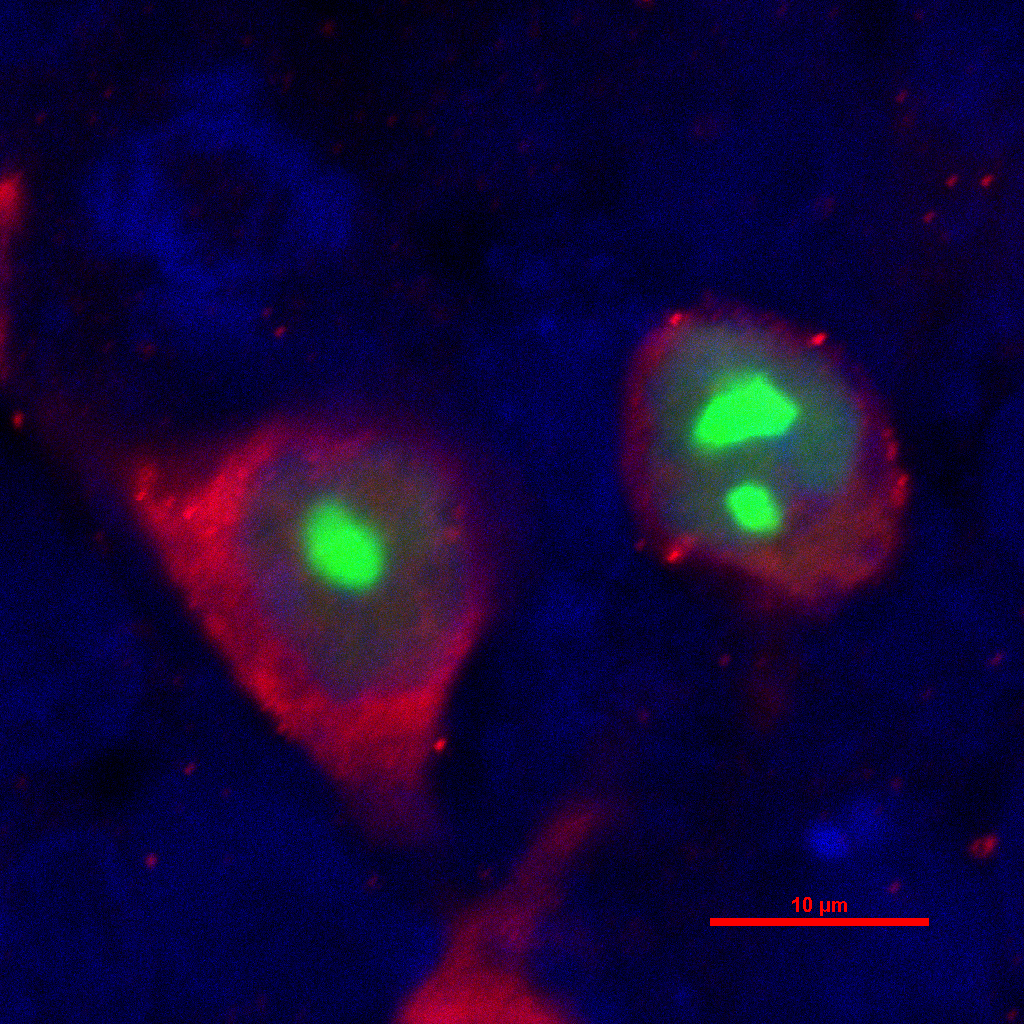

Supplement: S3 Data — (ZIP) [file ppat.1012546.s007.zip › Figure6D/3/flag-ASC+GFP-UL4(50-130)/Merge.tif]

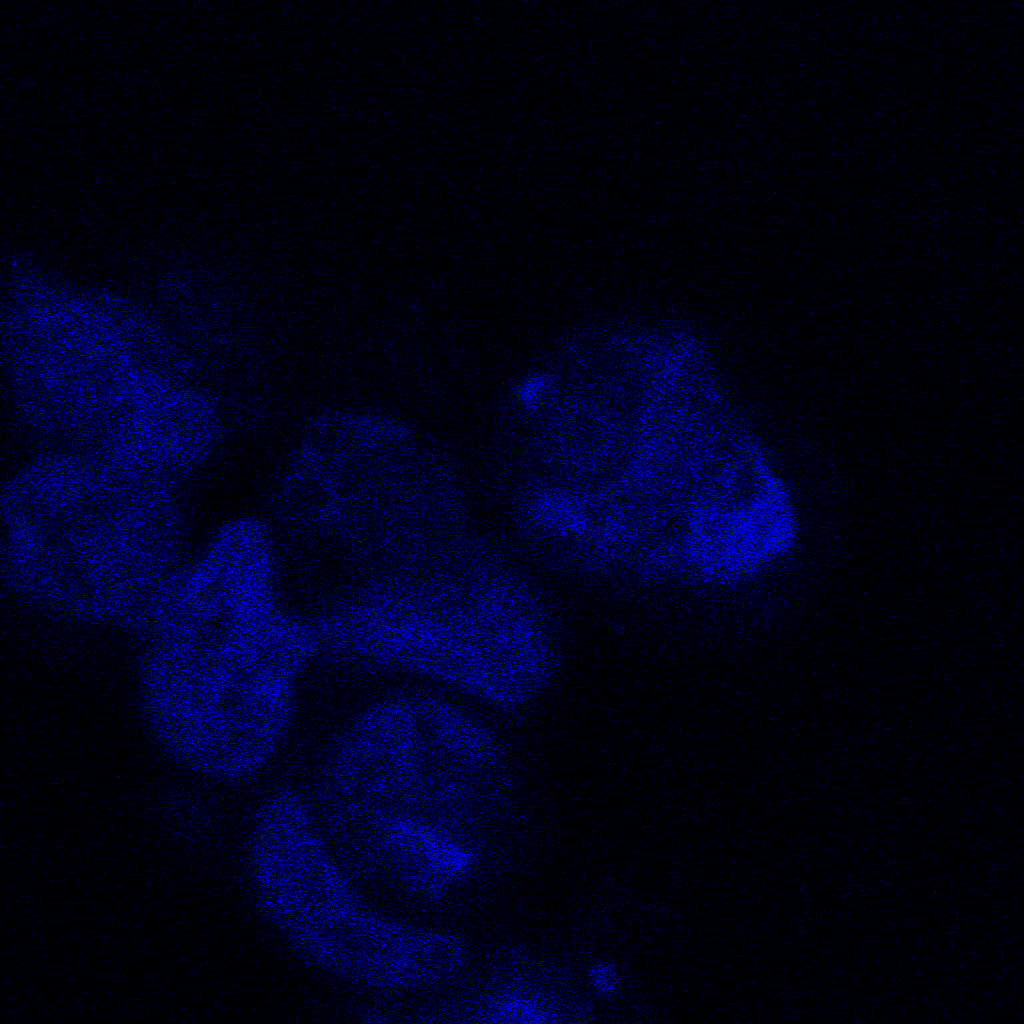

Supplement: S3 Data — (ZIP) [file ppat.1012546.s007.zip › Figure6D/3/flag-ASC+GFP-UL4(73-146)/DAPI.tif]

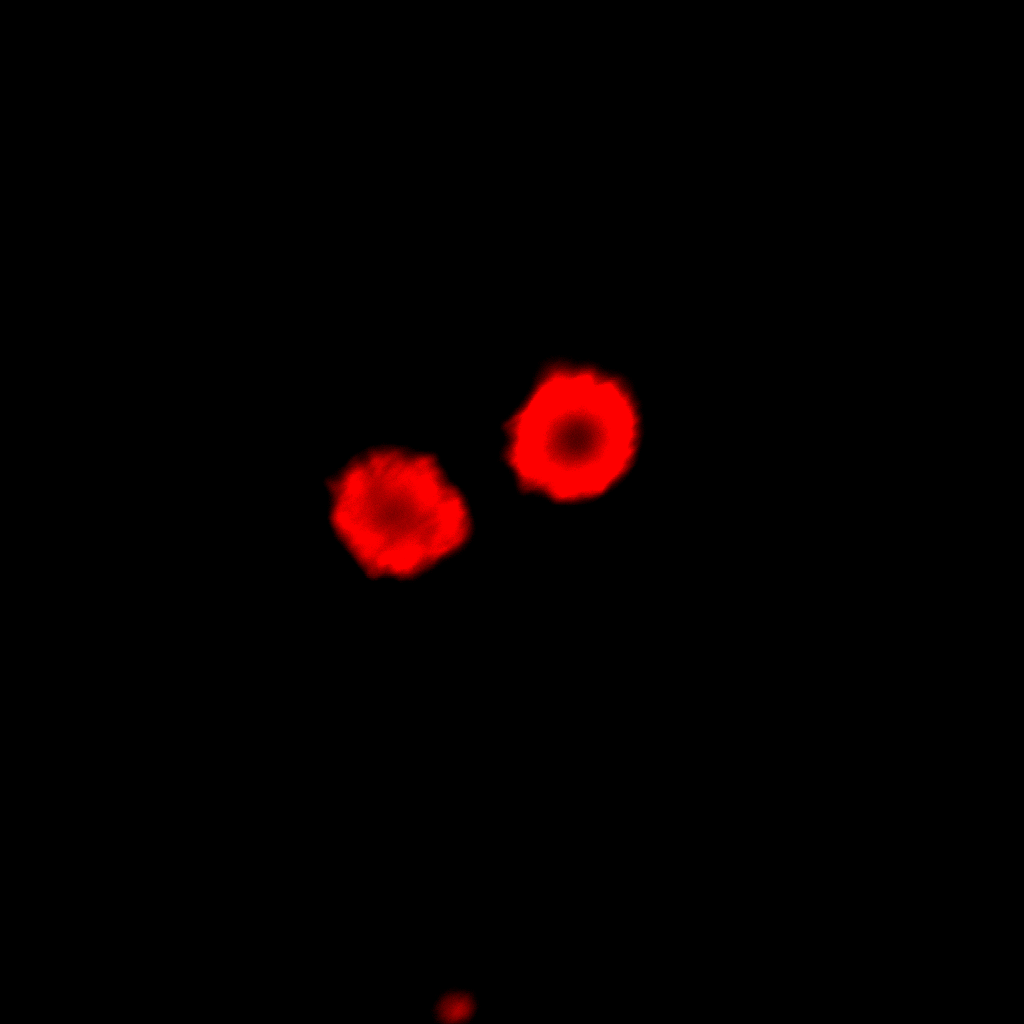

Supplement: S3 Data — (ZIP) [file ppat.1012546.s007.zip › Figure6D/3/flag-ASC+GFP-UL4(73-146)/flag-asc-3c3.tif]

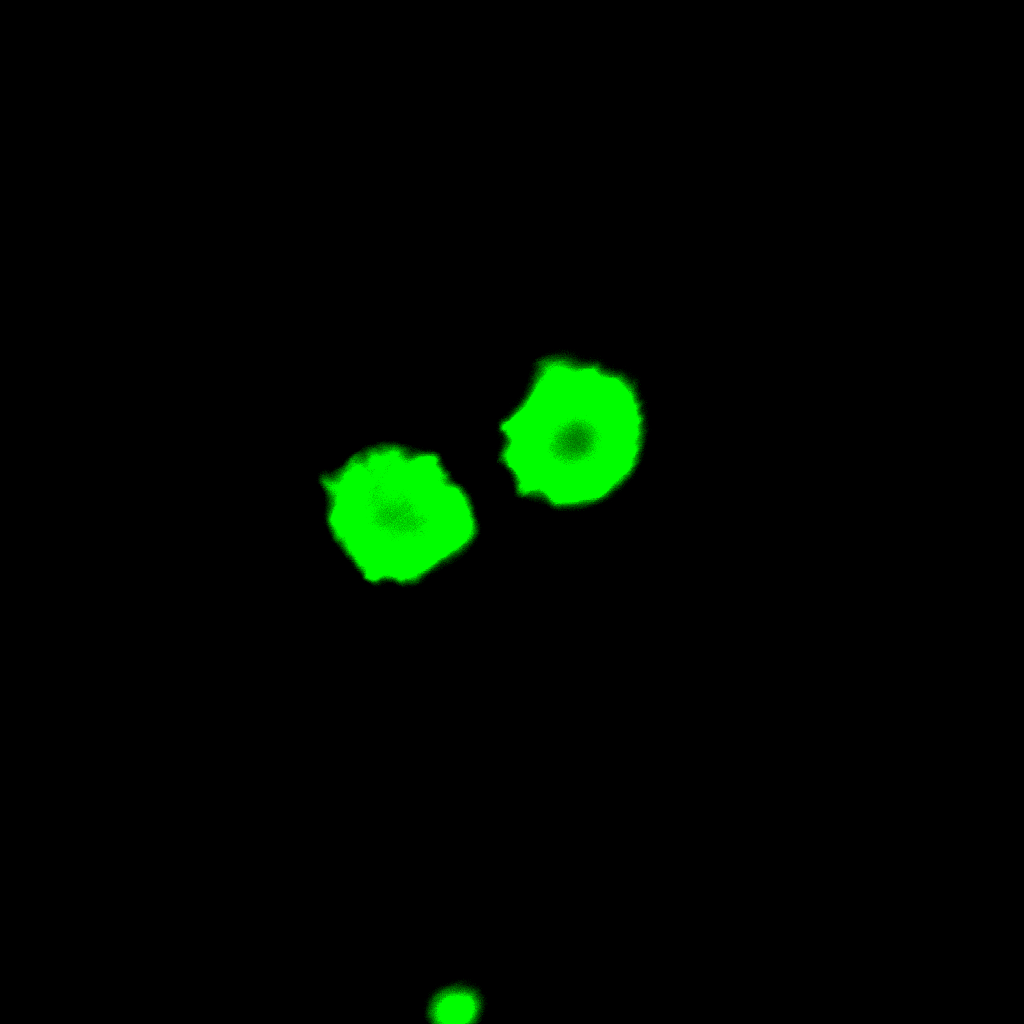

Supplement: S3 Data — (ZIP) [file ppat.1012546.s007.zip › Figure6D/3/flag-ASC+GFP-UL4(73-146)/gfp-ul4(73-146).tif]

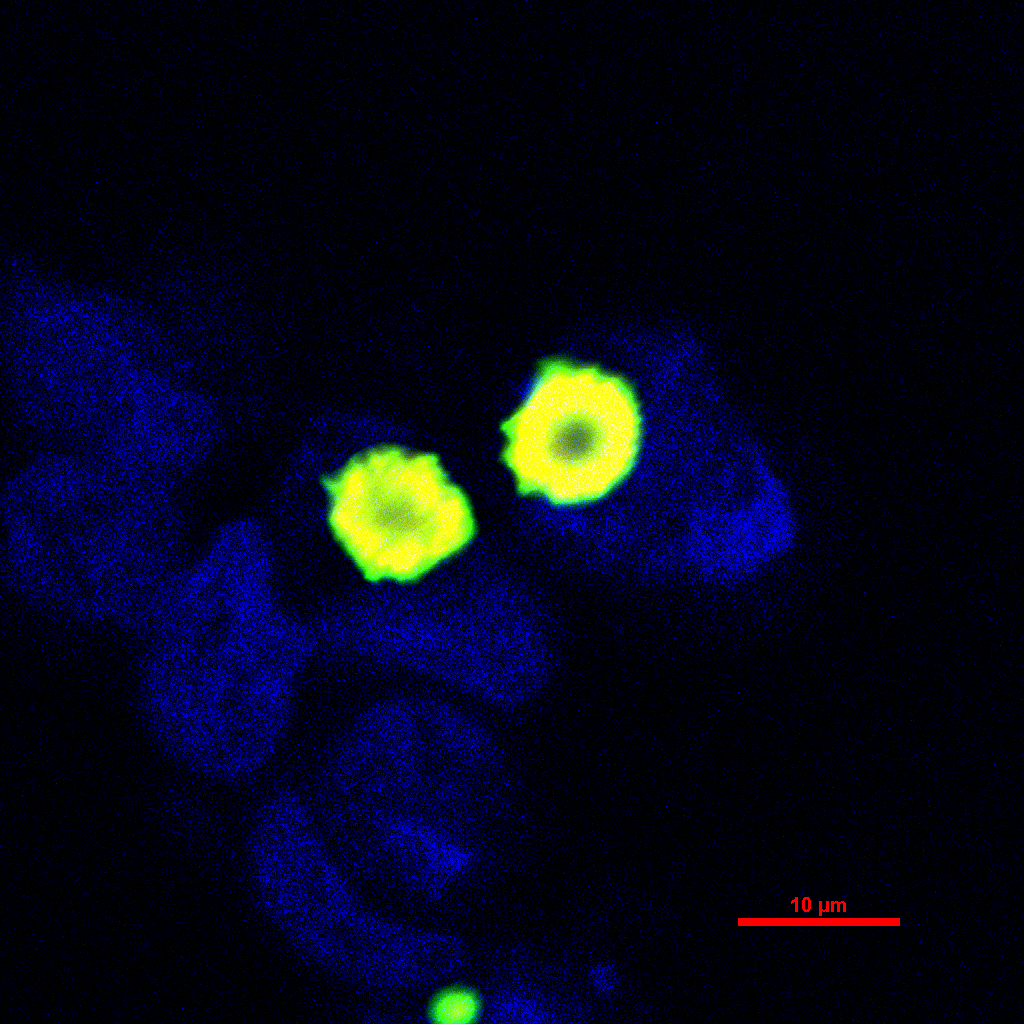

Supplement: S3 Data — (ZIP) [file ppat.1012546.s007.zip › Figure6D/3/flag-ASC+GFP-UL4(73-146)/Merge.tif]

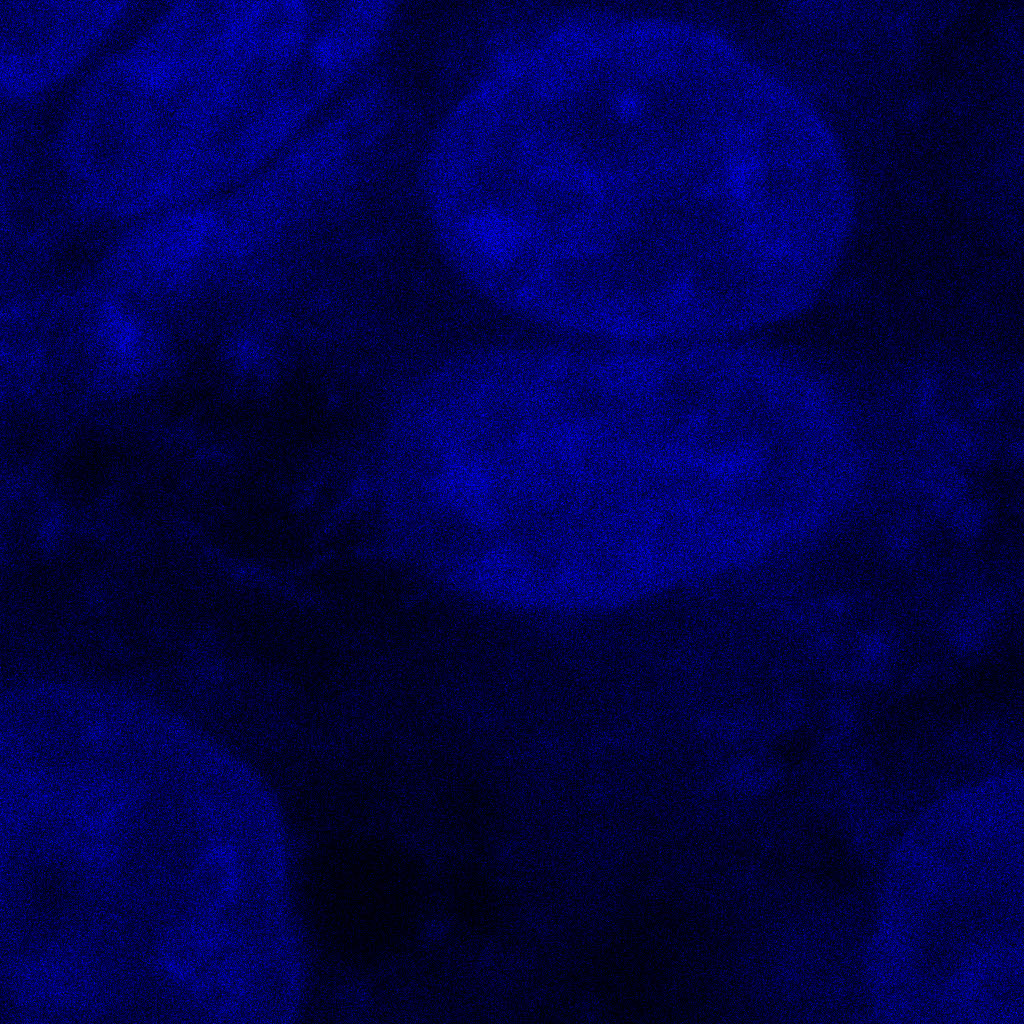

Supplement: S3 Data — (ZIP) [file ppat.1012546.s007.zip › Figure6D/3/GFP-UL4(1-73)/DAPI.tif]

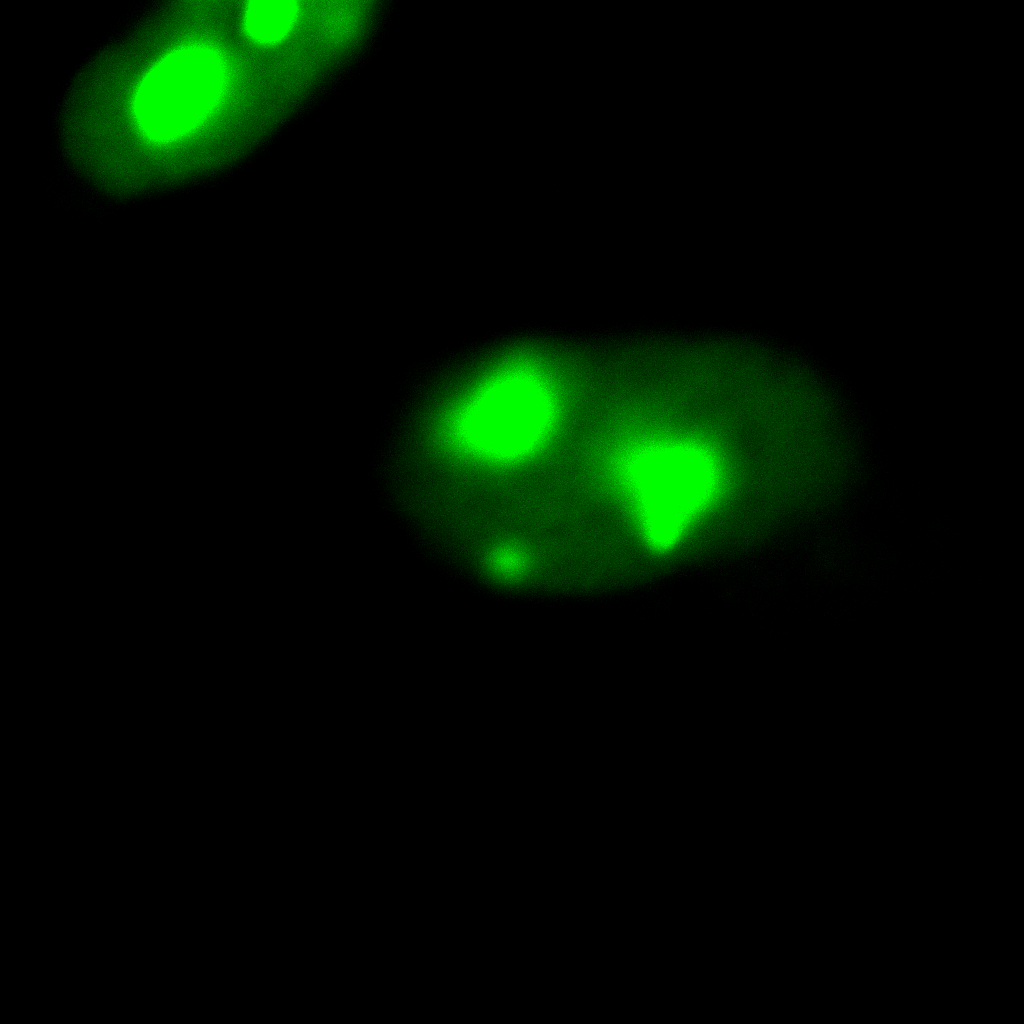

Supplement: S3 Data — (ZIP) [file ppat.1012546.s007.zip › Figure6D/3/GFP-UL4(1-73)/gfp-ul4 ú¿1-73ú⌐.tif]

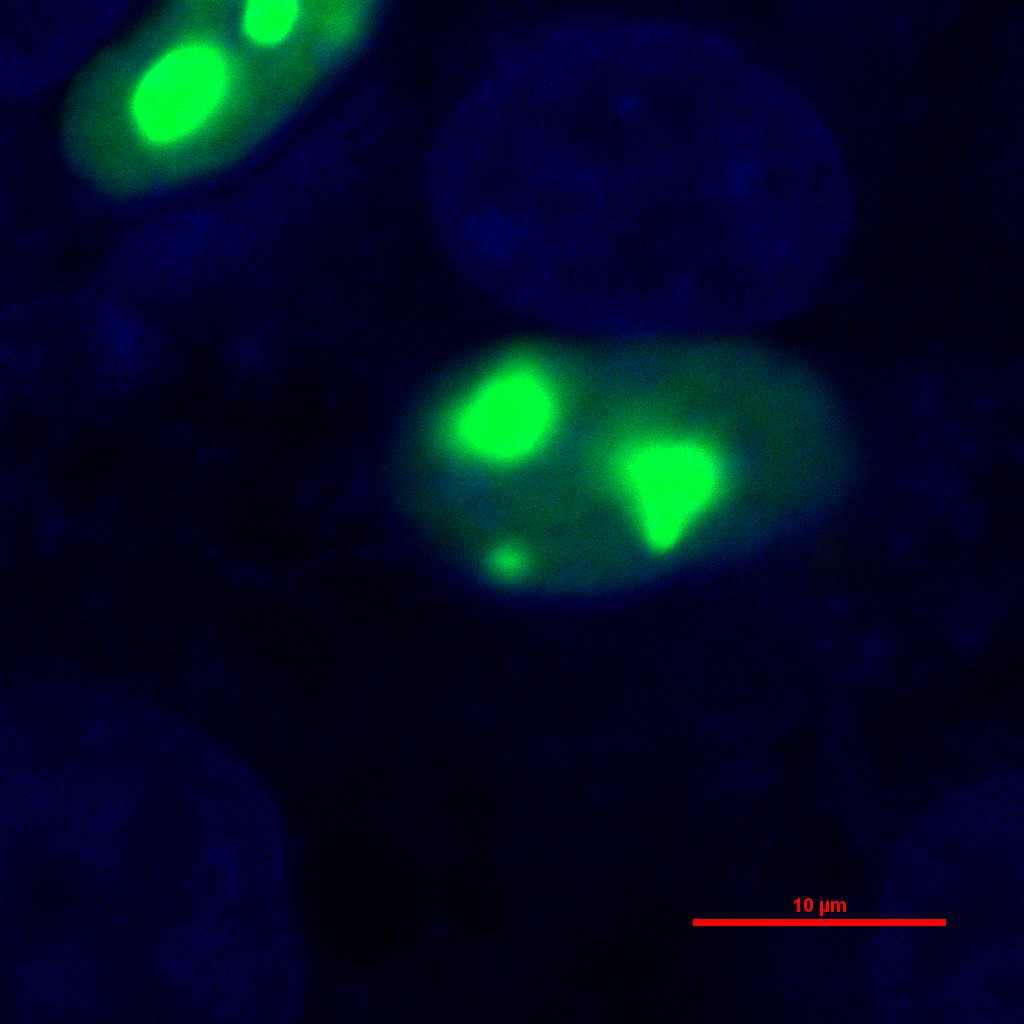

Supplement: S3 Data — (ZIP) [file ppat.1012546.s007.zip › Figure6D/3/GFP-UL4(1-73)/Merge.tif]
