# Supplementary figures and images for: Pseudorabies Virus UL4 protein promotes the ASC-dependent inflammasome activation and pyroptosis to exacerbate inflammation (part 4 of 6)
Source: PLoS Pathog. 2024 Sep 24;20(9):e1012546. doi: 10.1371/journal.ppat.1012546 (PMC11421794; doi:10.1371/journal.ppat.1012546)

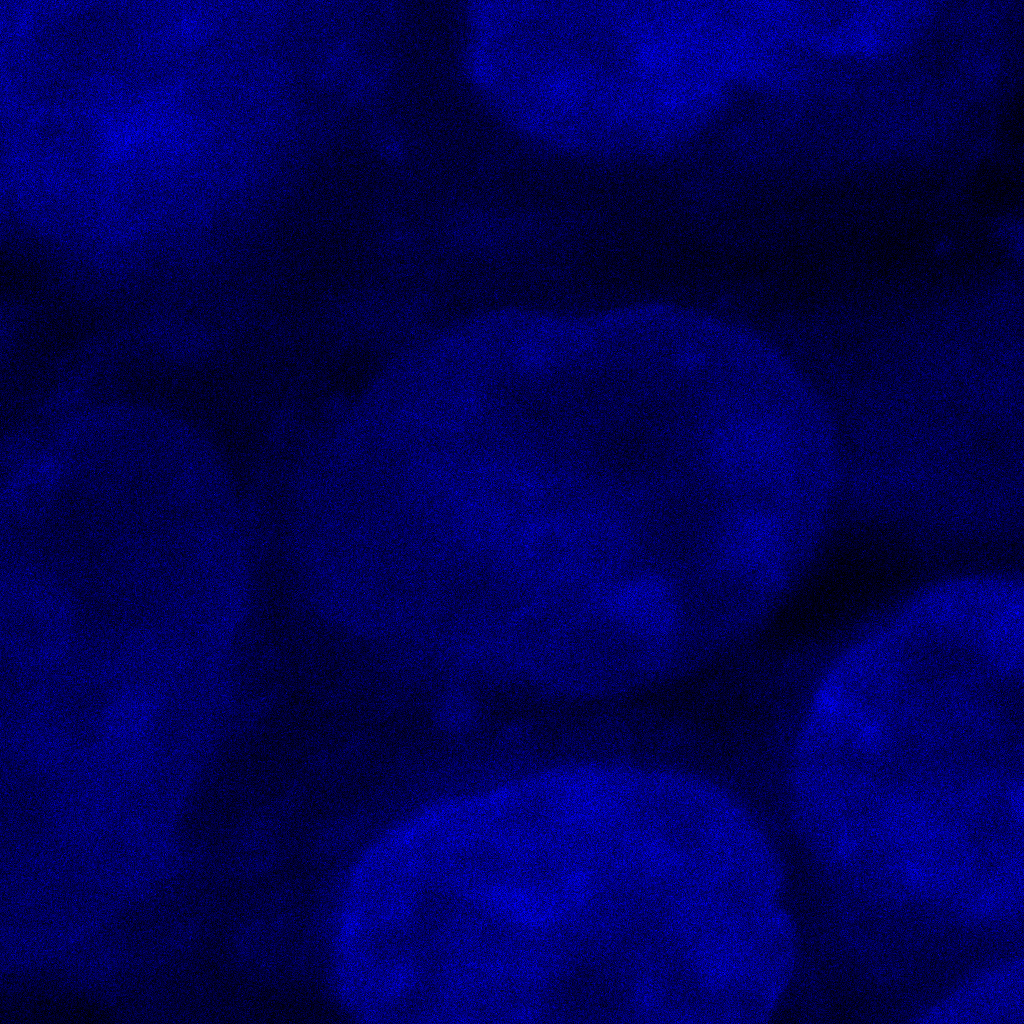

Supplement: S3 Data — (ZIP) [file ppat.1012546.s007.zip › Figure6D/3/GFP-UL4(50-130)/DAPI.tif]

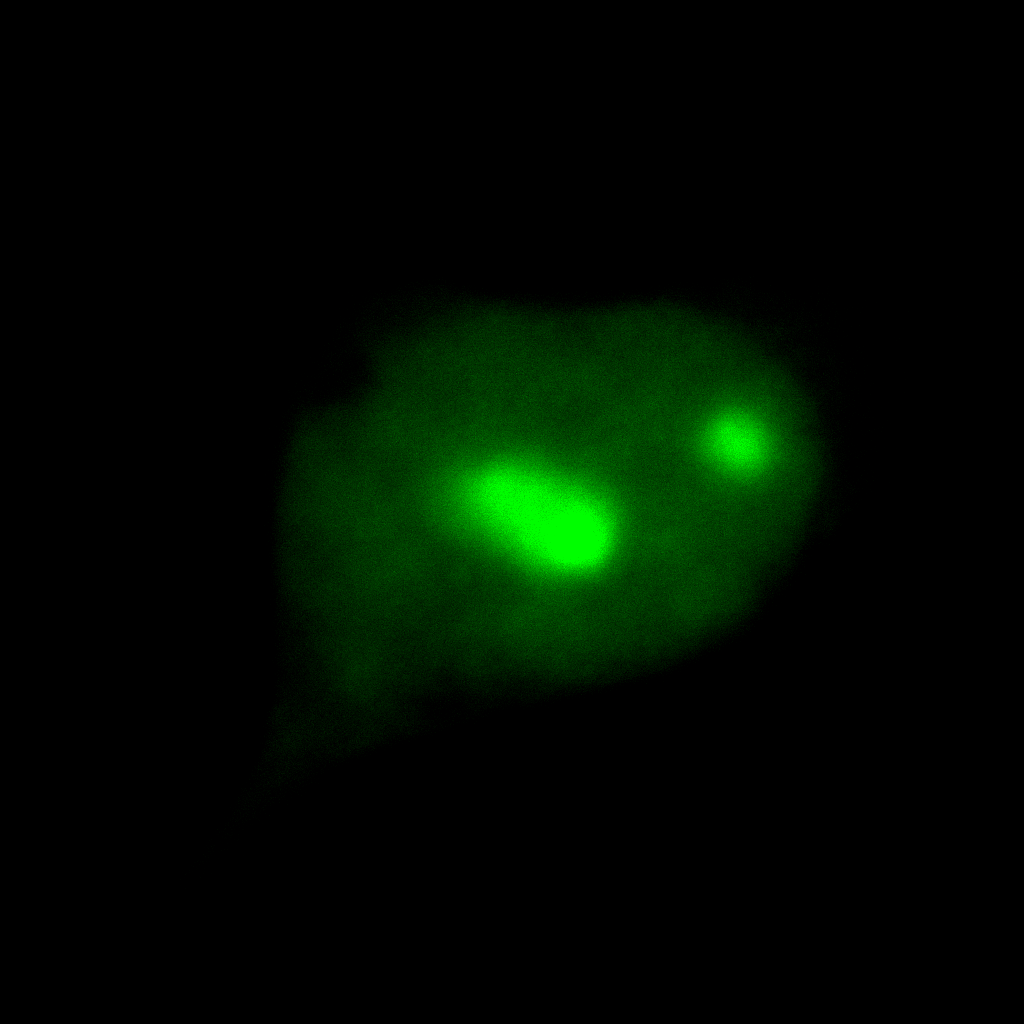

Supplement: S3 Data — (ZIP) [file ppat.1012546.s007.zip › Figure6D/3/GFP-UL4(50-130)/gfp-ul4 (50-130).tif]

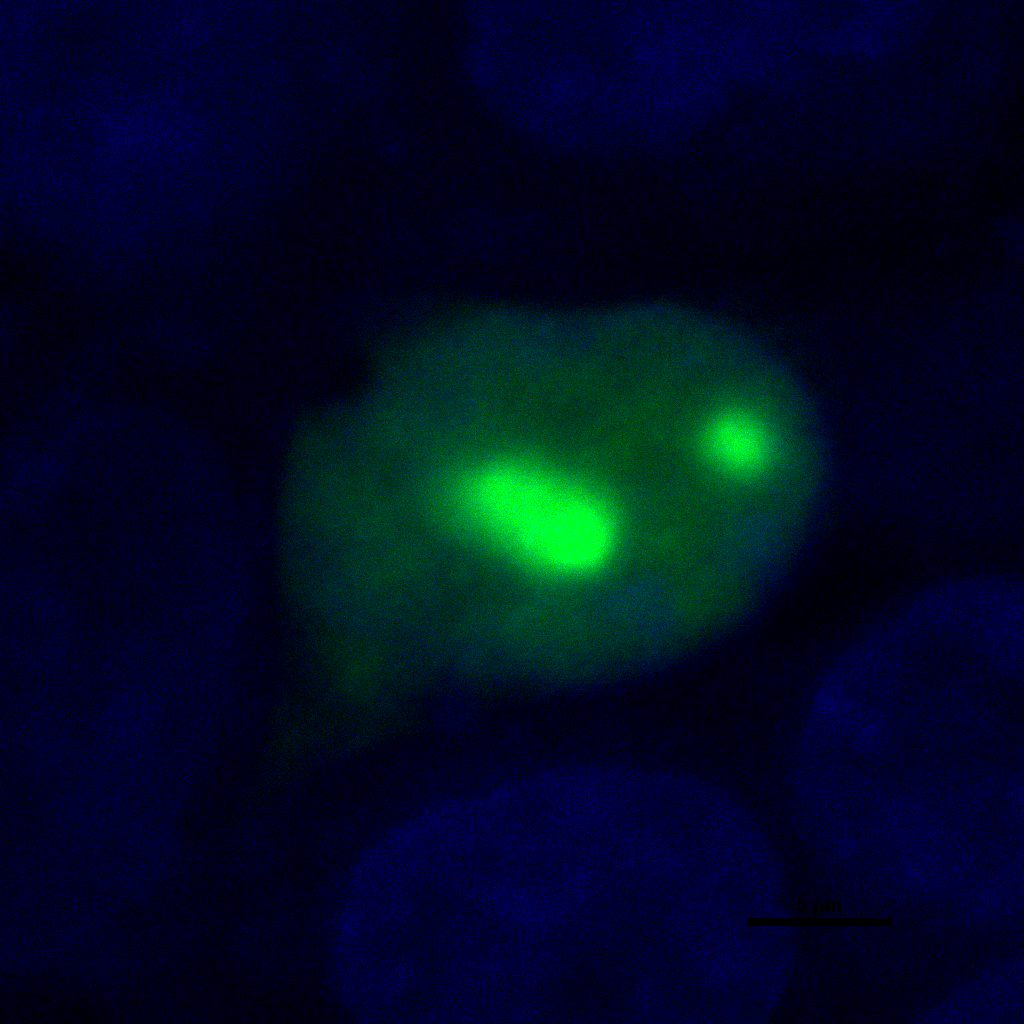

Supplement: S3 Data — (ZIP) [file ppat.1012546.s007.zip › Figure6D/3/GFP-UL4(50-130)/Merge.tif]

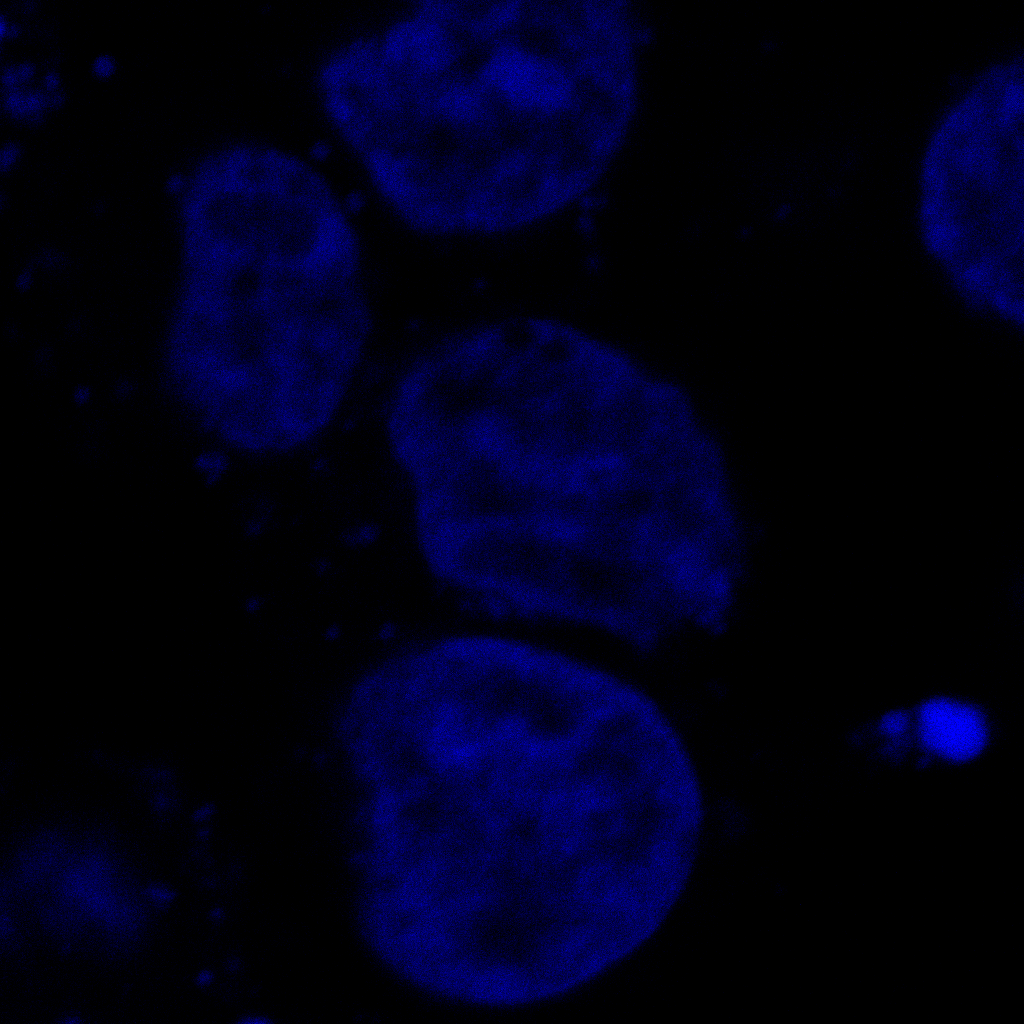

Supplement: S3 Data — (ZIP) [file ppat.1012546.s007.zip › Figure6D/3/GFP-UL4(73-146)/DAPI.tif]

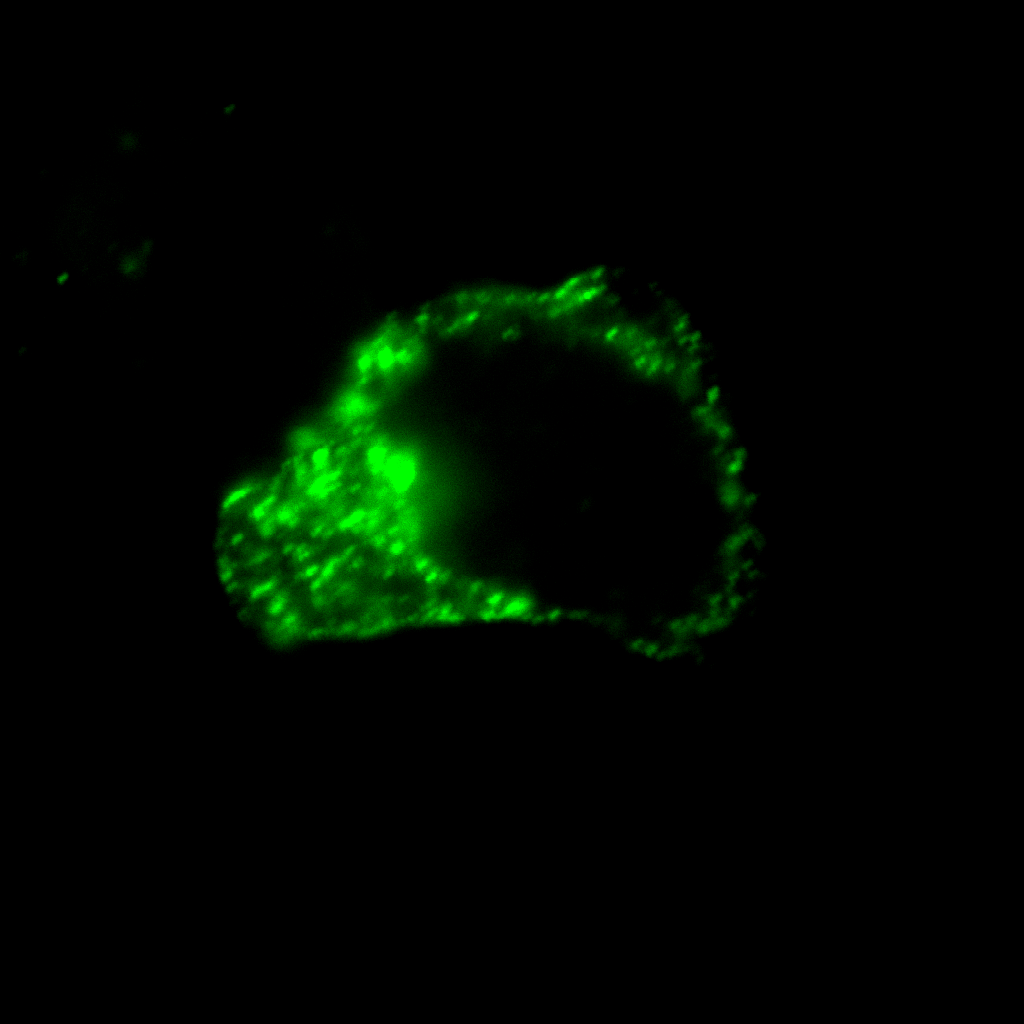

Supplement: S3 Data — (ZIP) [file ppat.1012546.s007.zip › Figure6D/3/GFP-UL4(73-146)/gfp-ul4(73-146).tif]

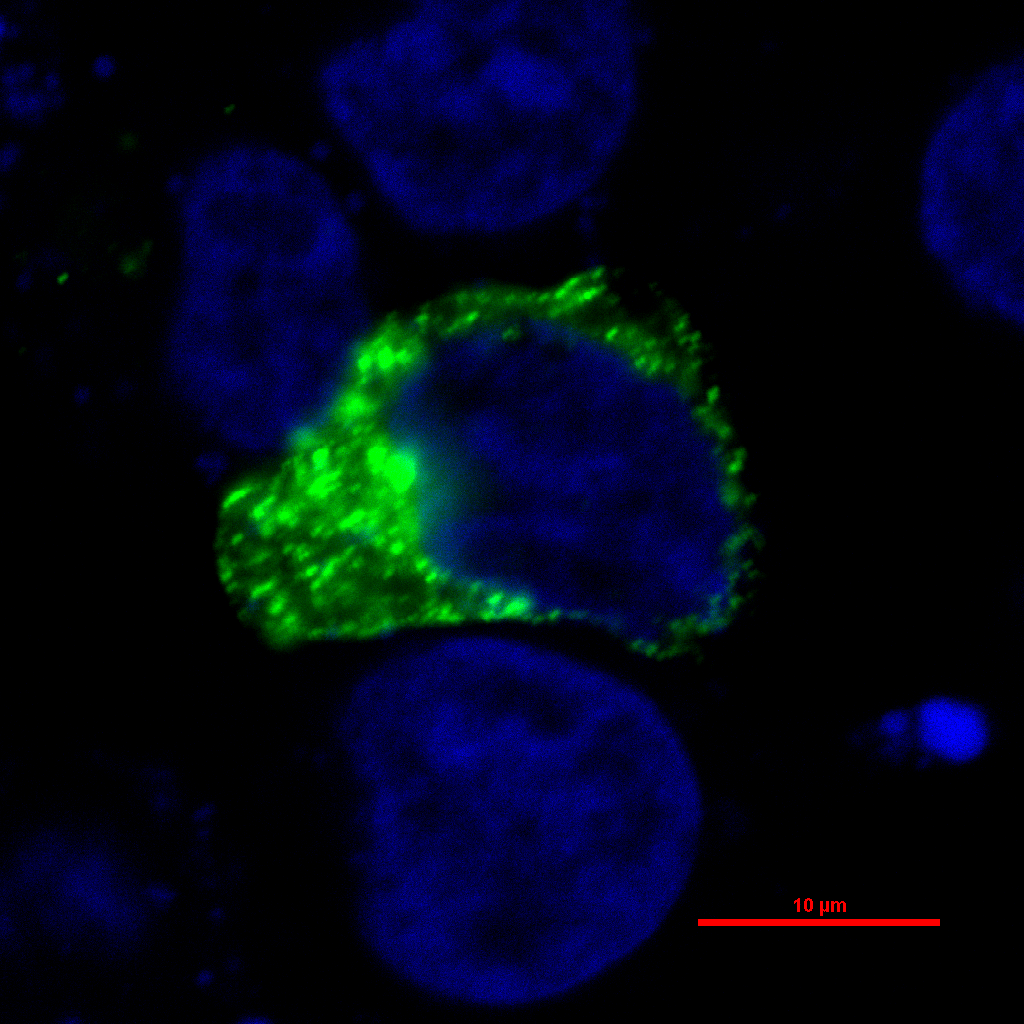

Supplement: S3 Data — (ZIP) [file ppat.1012546.s007.zip › Figure6D/3/GFP-UL4(73-146)/Merge.tif]

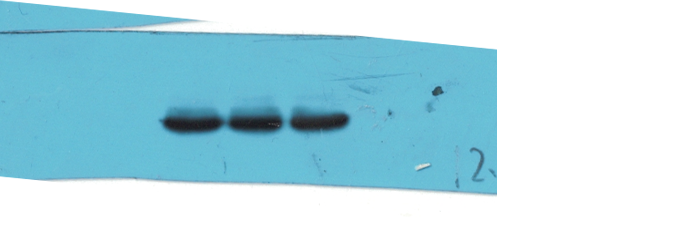

Supplement: S4 Data — (ZIP) [file ppat.1012546.s008.zip › Figure 8-10/Fig10/F/1/Brain-Actin.tif]

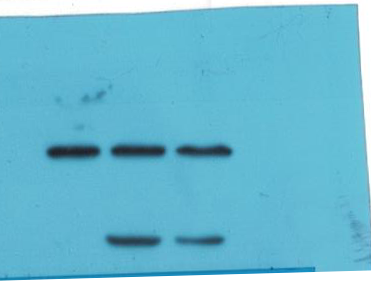

Supplement: S4 Data — (ZIP) [file ppat.1012546.s008.zip › Figure 8-10/Fig10/F/1/Brain-GSDMD-Full+GSDMD-NT.tif]

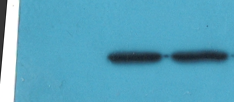

Supplement: S4 Data — (ZIP) [file ppat.1012546.s008.zip › Figure 8-10/Fig10/F/1/Brain-UL4.tif]

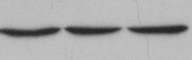

Supplement: S4 Data — (ZIP) [file ppat.1012546.s008.zip › Figure 8-10/Fig10/F/1/Lung-Actin.tif]

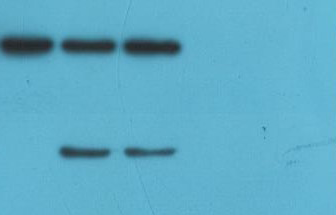

Supplement: S4 Data — (ZIP) [file ppat.1012546.s008.zip › Figure 8-10/Fig10/F/1/Lung-GSDMD-Full+GSDMD-nt.tif]

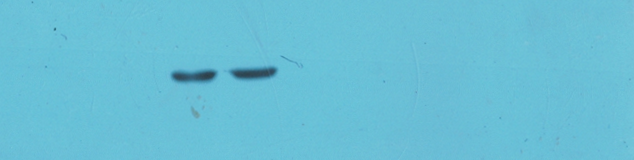

Supplement: S4 Data — (ZIP) [file ppat.1012546.s008.zip › Figure 8-10/Fig10/F/1/Lung-UL4.tif]

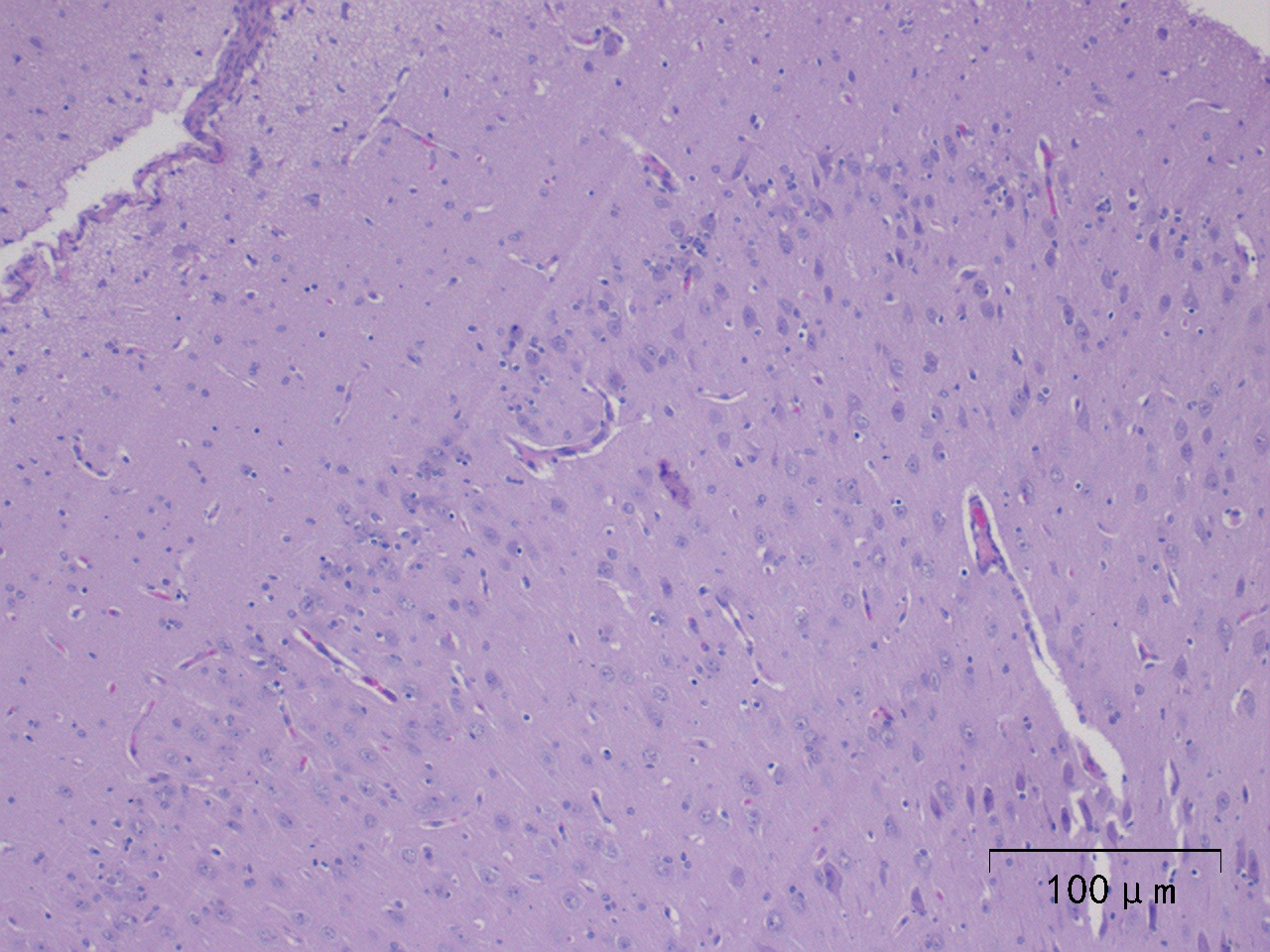

Supplement: S4 Data — (ZIP) [file ppat.1012546.s008.zip › Figure 8-10/Fig10/I/pig/Mock-Brain/19.jpg]

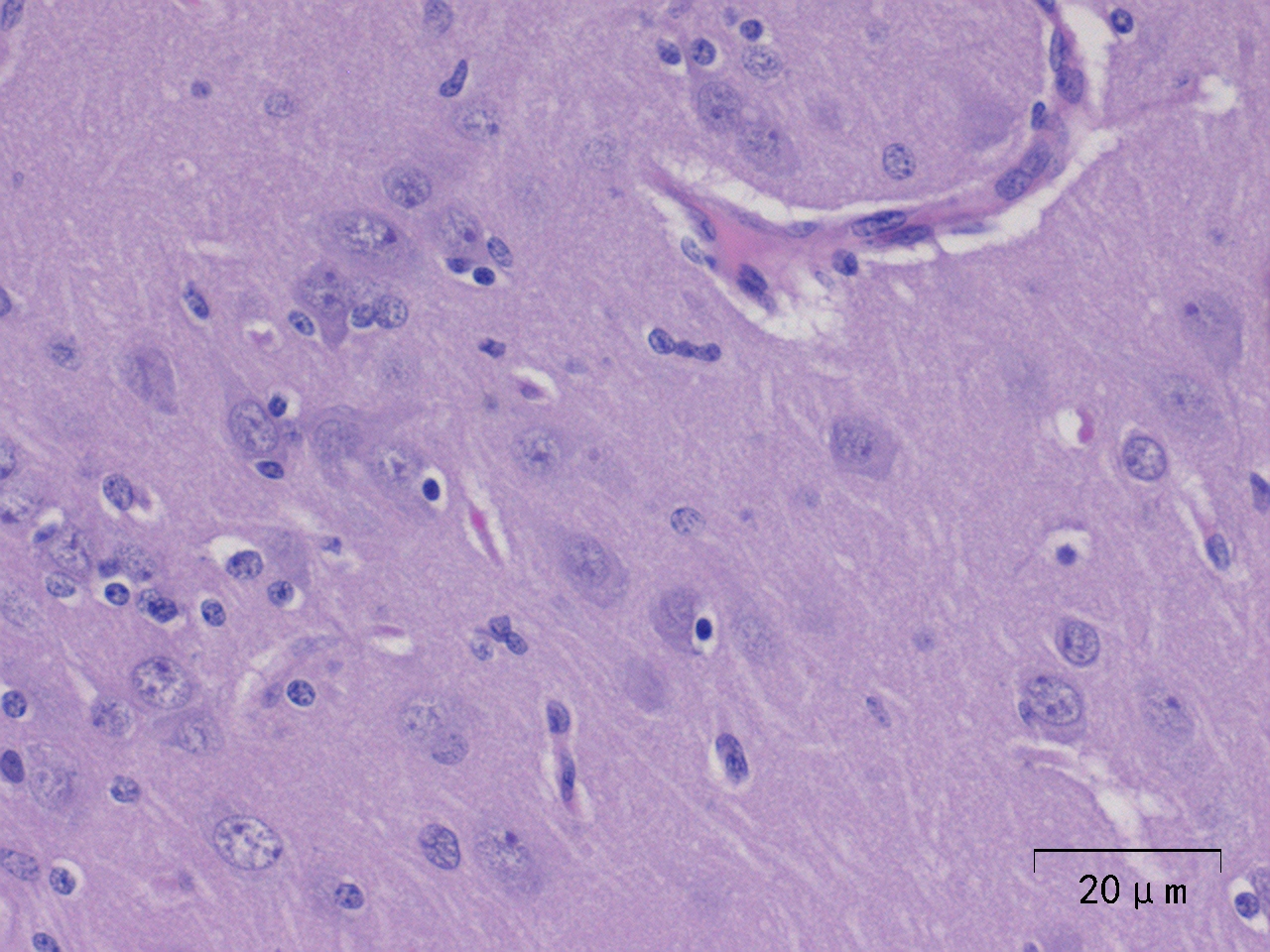

Supplement: S4 Data — (ZIP) [file ppat.1012546.s008.zip › Figure 8-10/Fig10/I/pig/Mock-Brain/20.jpg]

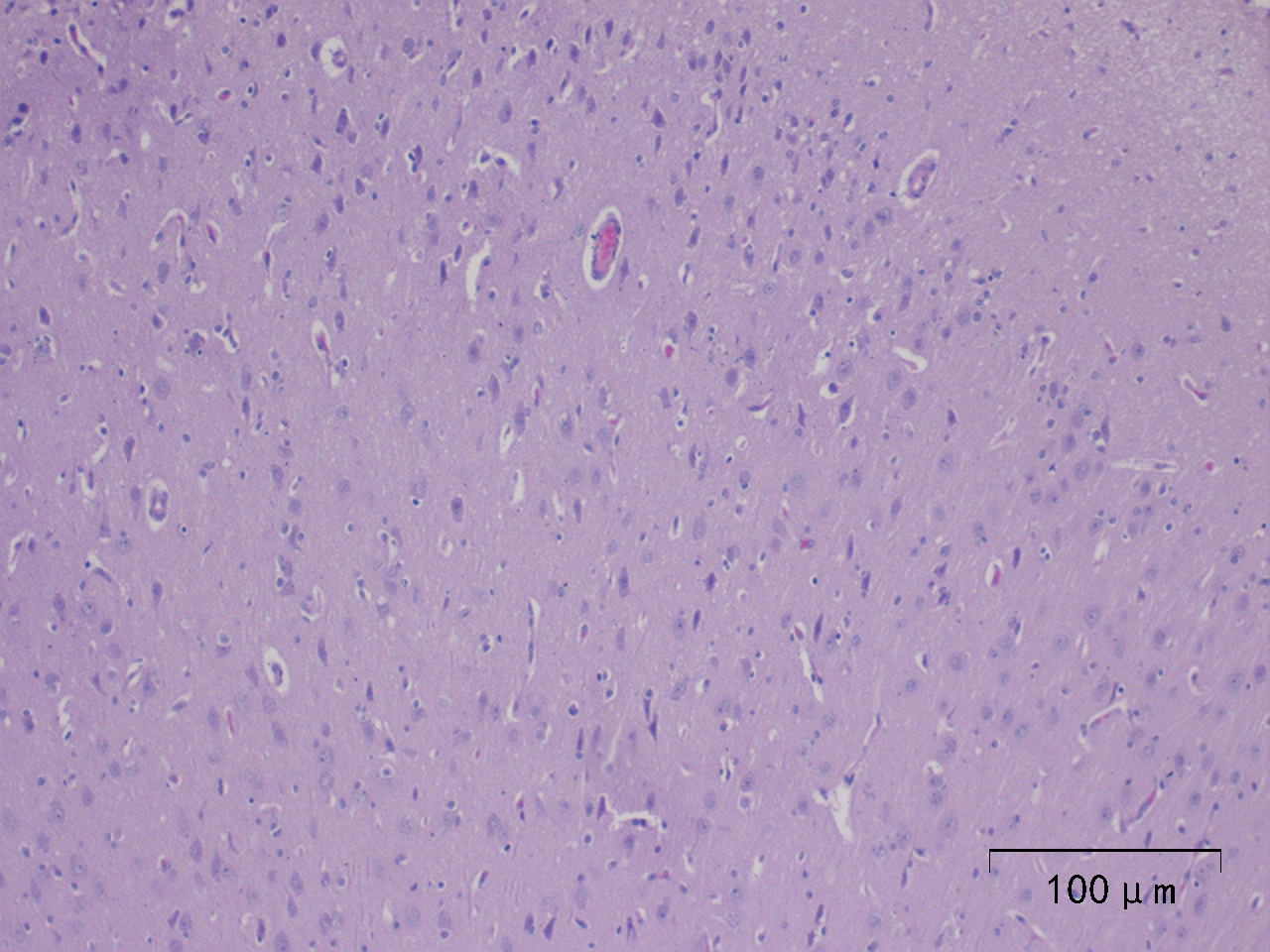

Supplement: S4 Data — (ZIP) [file ppat.1012546.s008.zip › Figure 8-10/Fig10/I/pig/Mock-Brain/21.jpg]

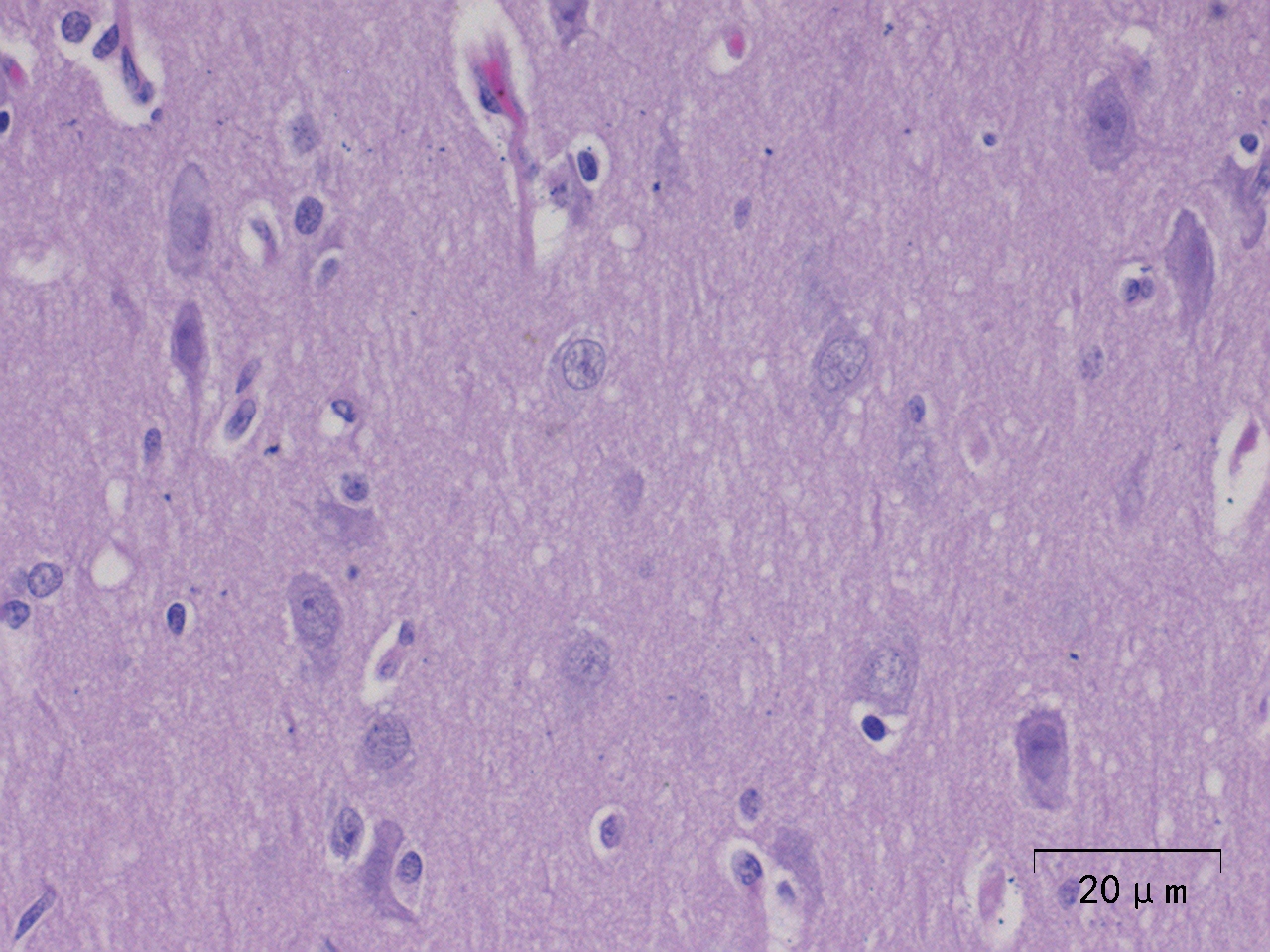

Supplement: S4 Data — (ZIP) [file ppat.1012546.s008.zip › Figure 8-10/Fig10/I/pig/Mock-Brain/22.jpg]

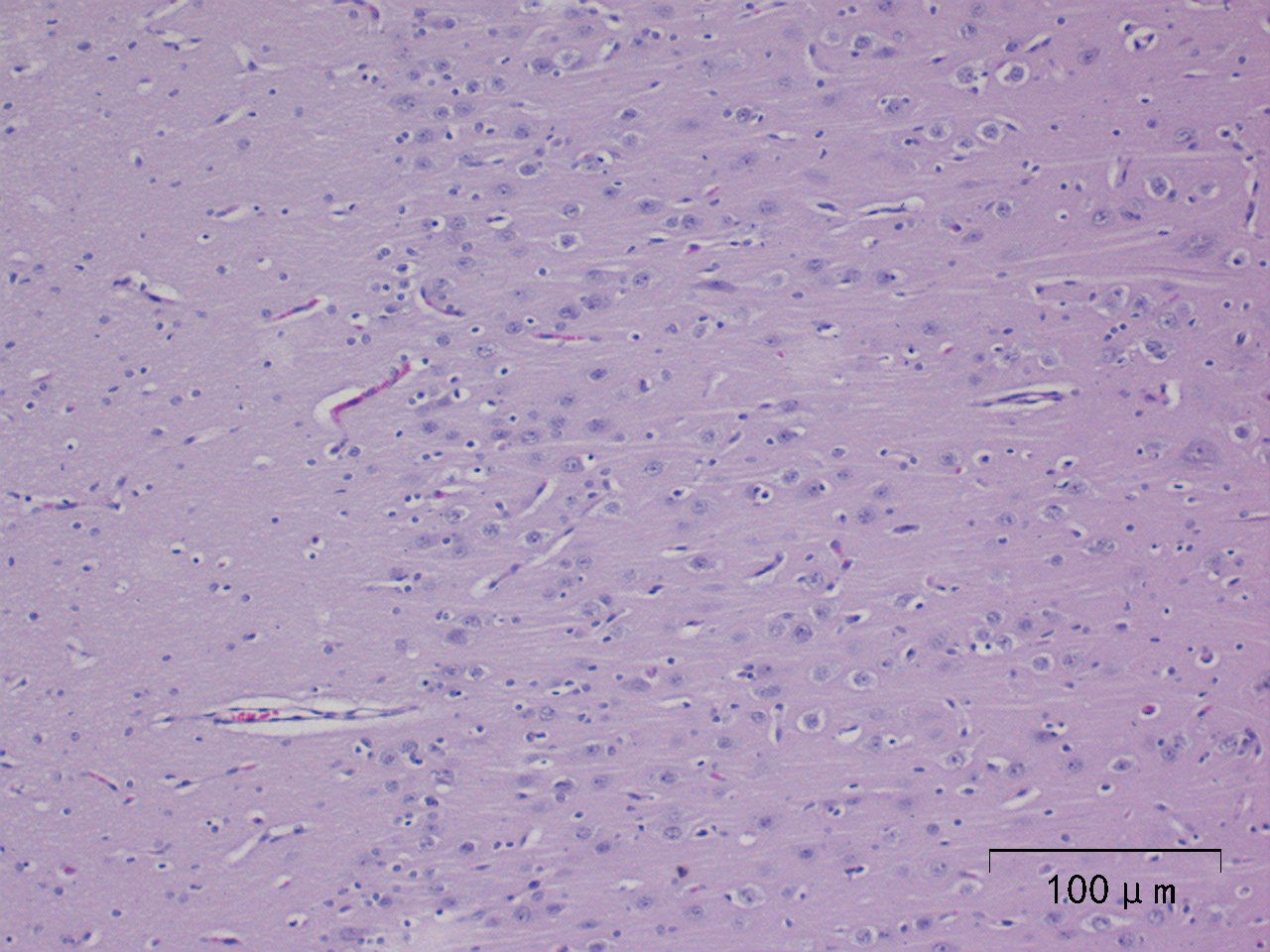

Supplement: S4 Data — (ZIP) [file ppat.1012546.s008.zip › Figure 8-10/Fig10/I/pig/Mock-Brain/23.jpg]

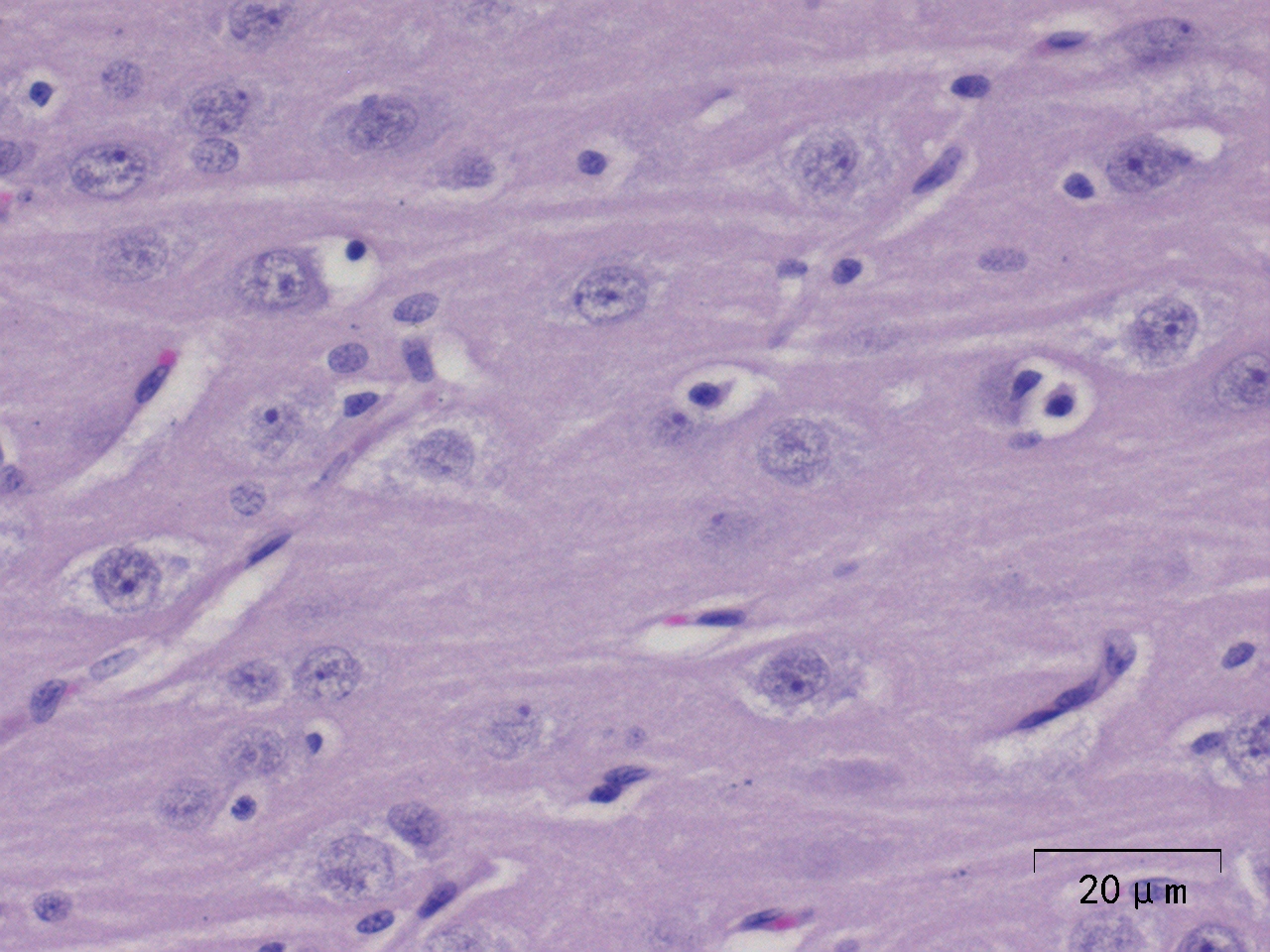

Supplement: S4 Data — (ZIP) [file ppat.1012546.s008.zip › Figure 8-10/Fig10/I/pig/Mock-Brain/24.jpg]

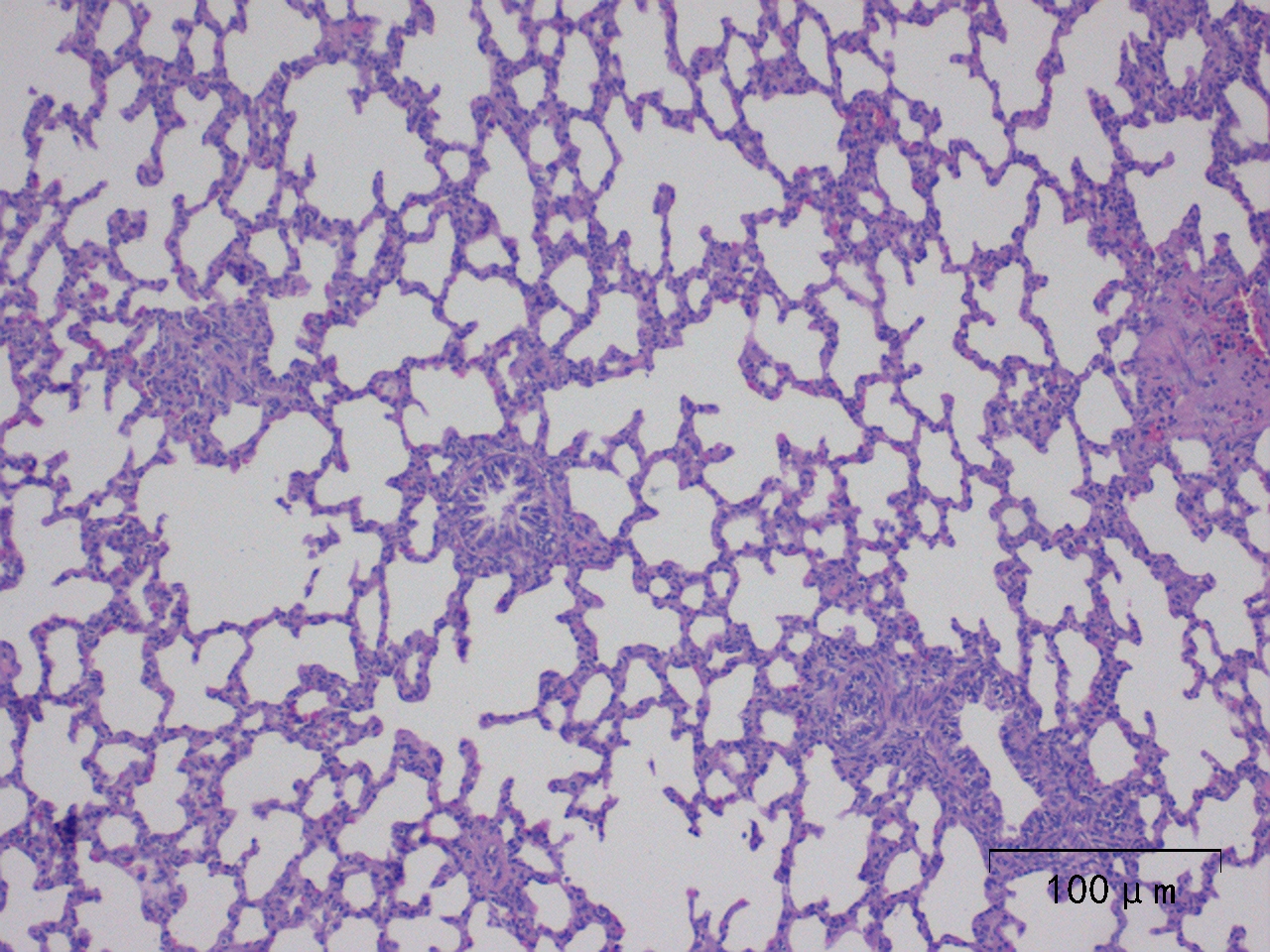

Supplement: S4 Data — (ZIP) [file ppat.1012546.s008.zip › Figure 8-10/Fig10/I/pig/Mock-lung/3.jpg]

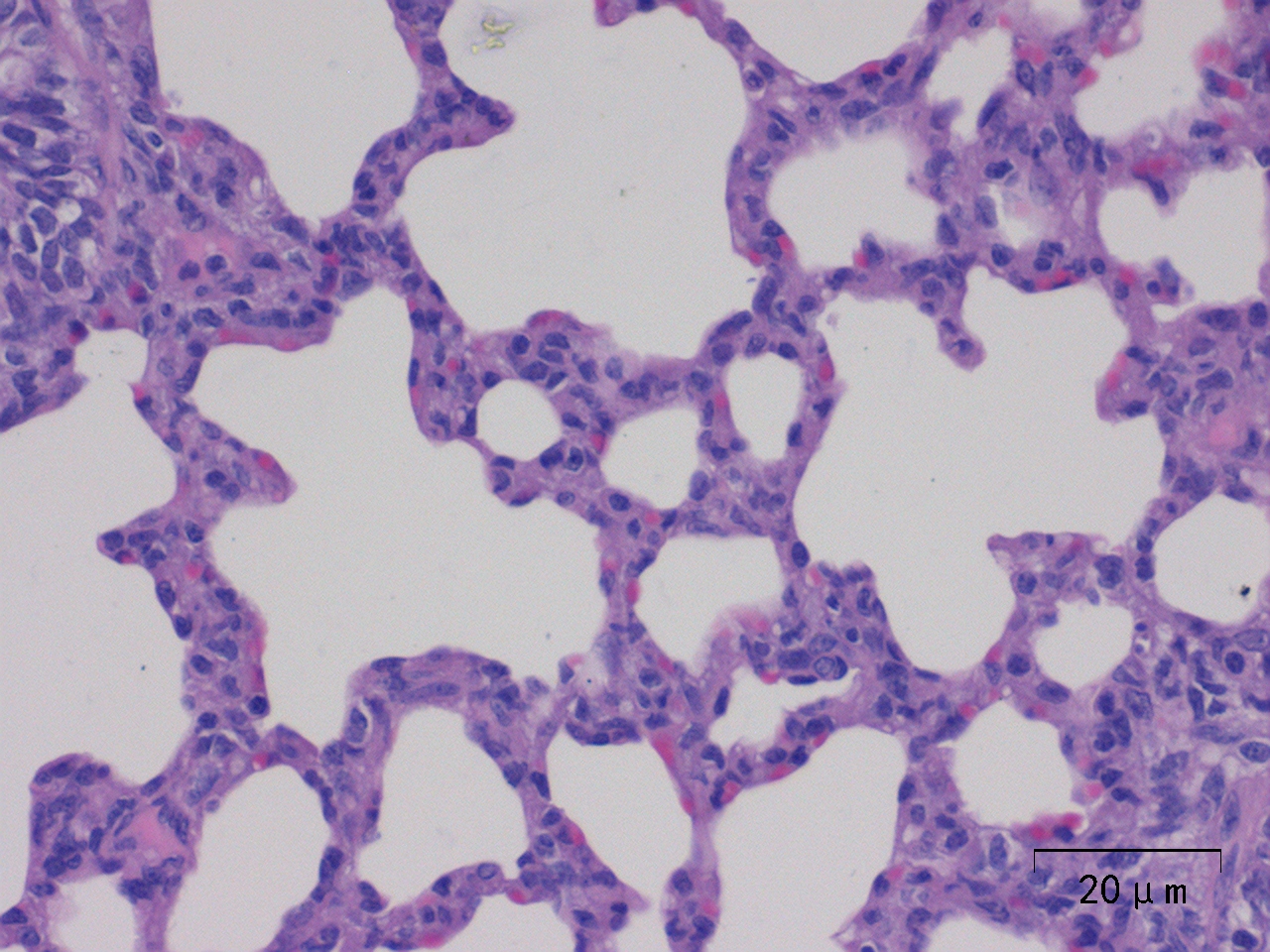

Supplement: S4 Data — (ZIP) [file ppat.1012546.s008.zip › Figure 8-10/Fig10/I/pig/Mock-lung/4.jpg]

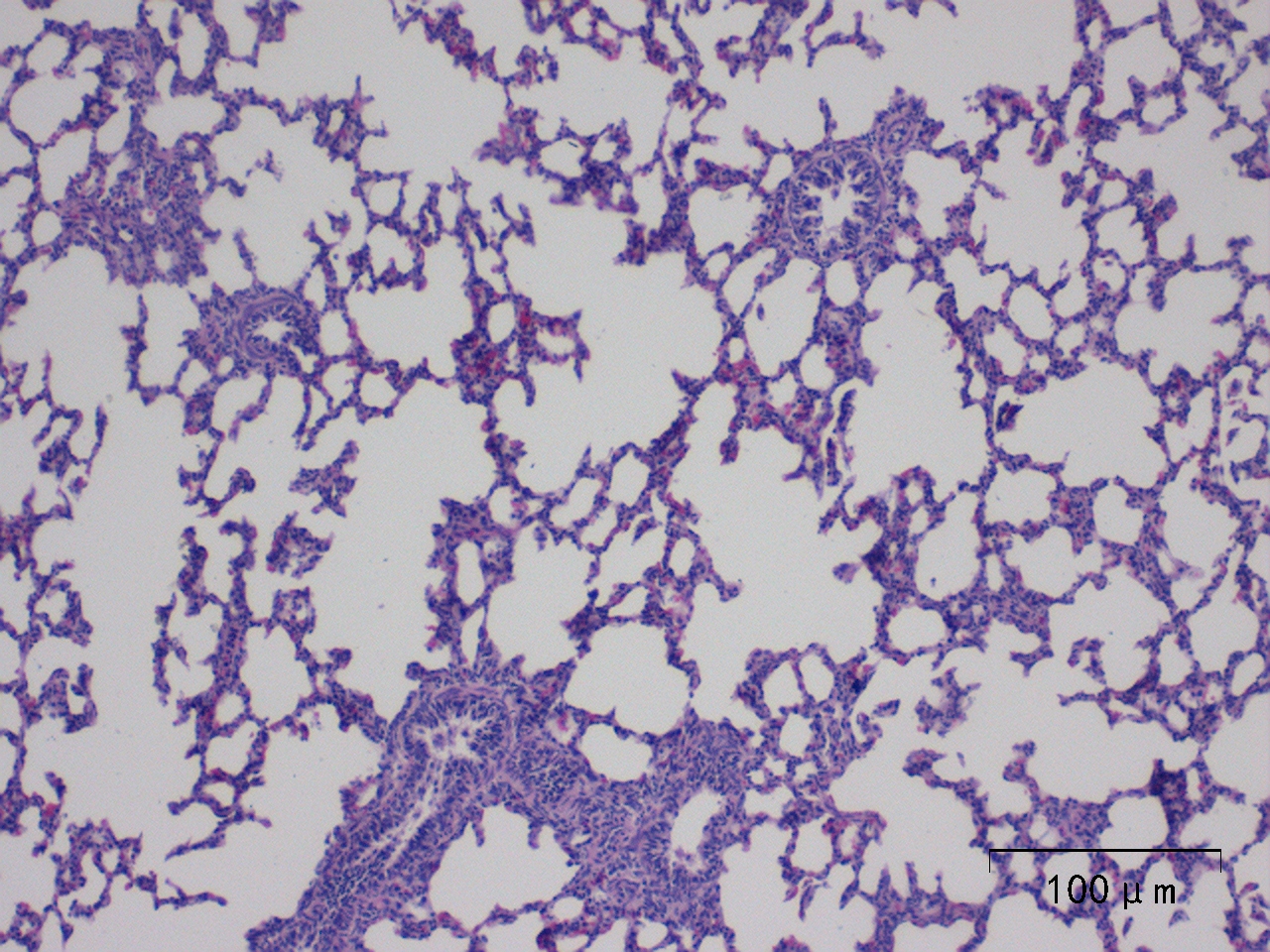

Supplement: S4 Data — (ZIP) [file ppat.1012546.s008.zip › Figure 8-10/Fig10/I/pig/Mock-lung/5.jpg]

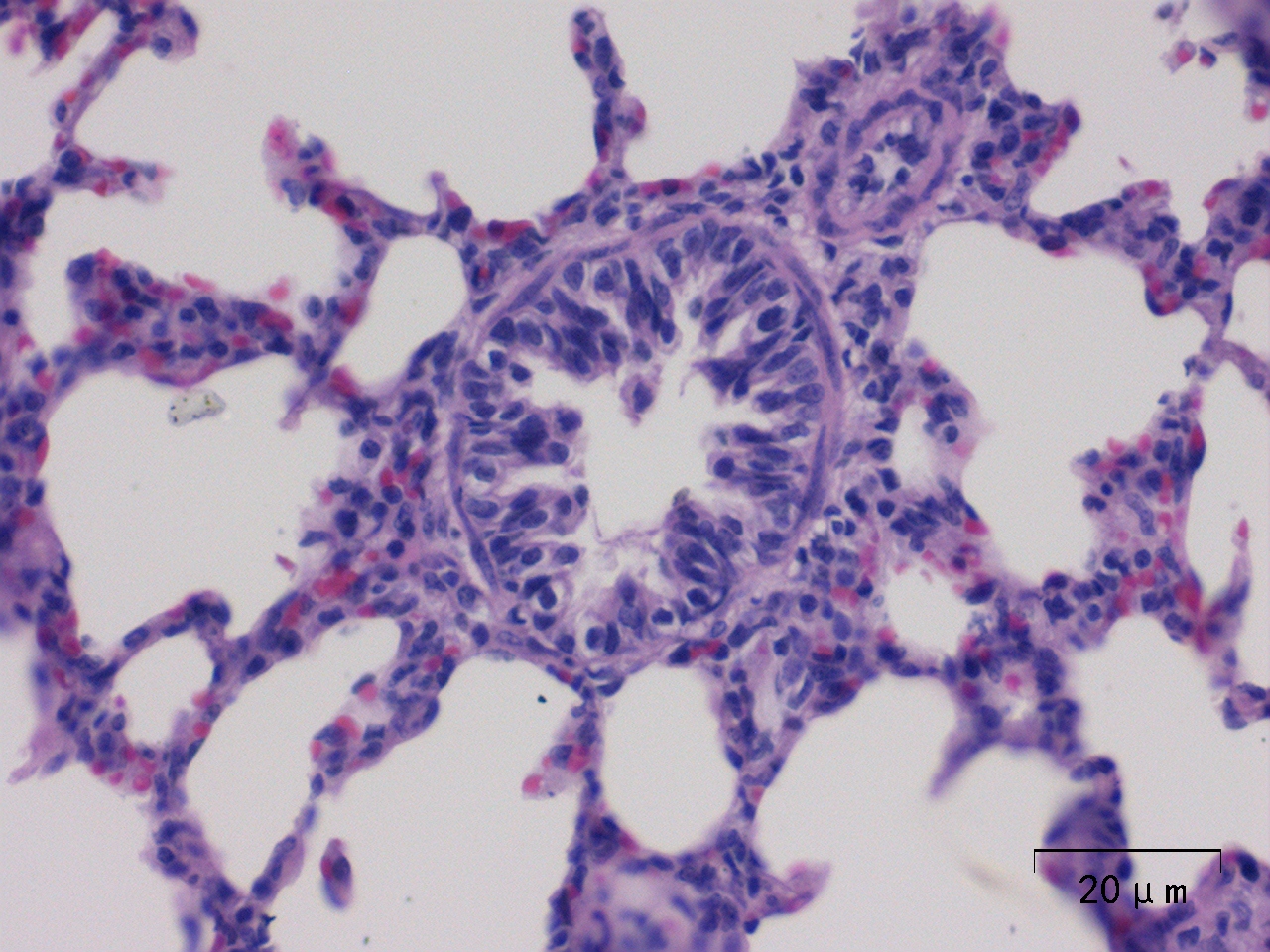

Supplement: S4 Data — (ZIP) [file ppat.1012546.s008.zip › Figure 8-10/Fig10/I/pig/Mock-lung/6.jpg]

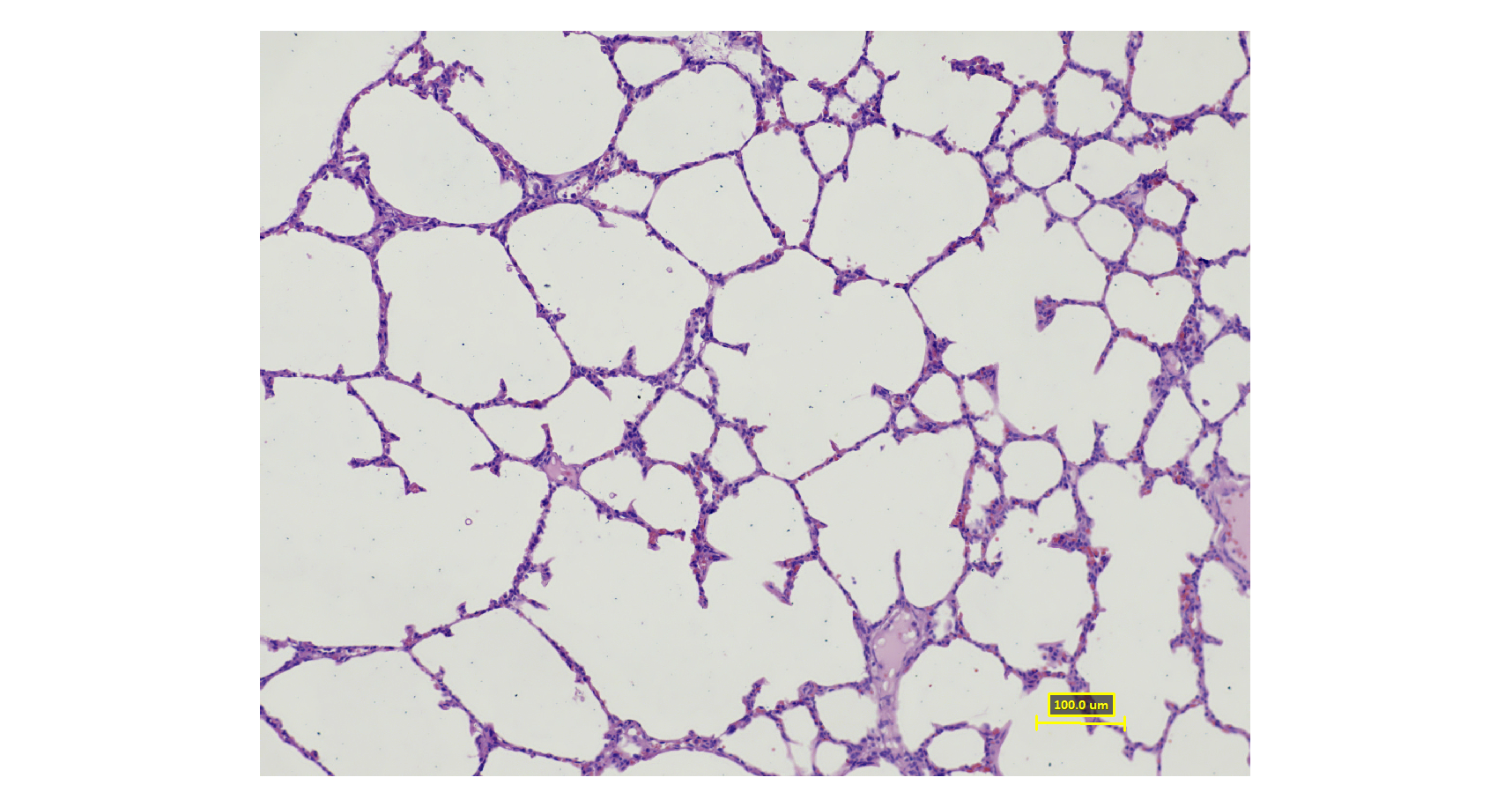

Supplement: S4 Data — (ZIP) [file ppat.1012546.s008.zip › Figure 8-10/Fig10/I/pig/Mock-lung/Mock-f1-10x-1.tif]

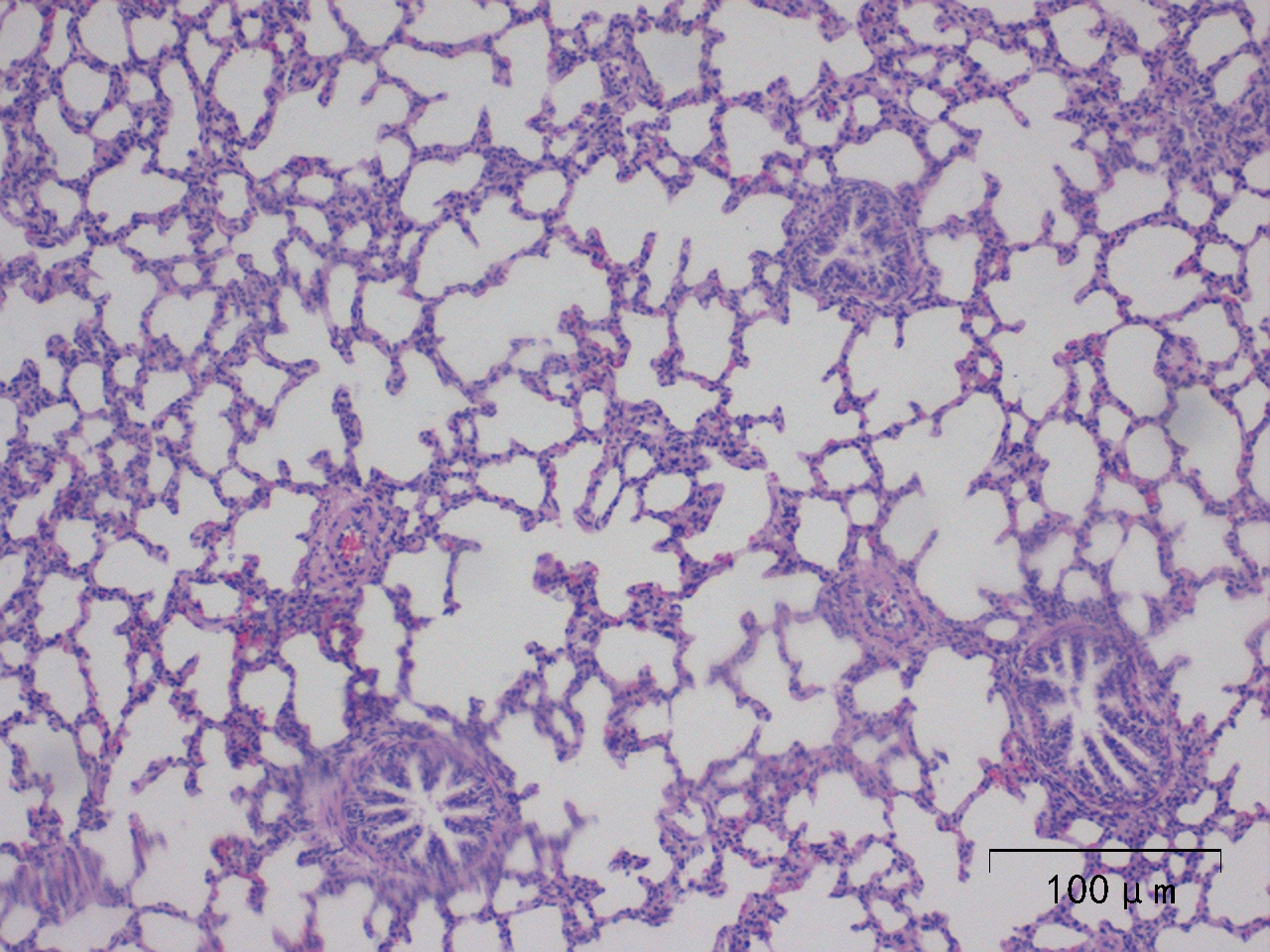

Supplement: S4 Data — (ZIP) [file ppat.1012546.s008.zip › Figure 8-10/Fig10/I/pig/Mock-lung/Mock-lung-1.jpg]

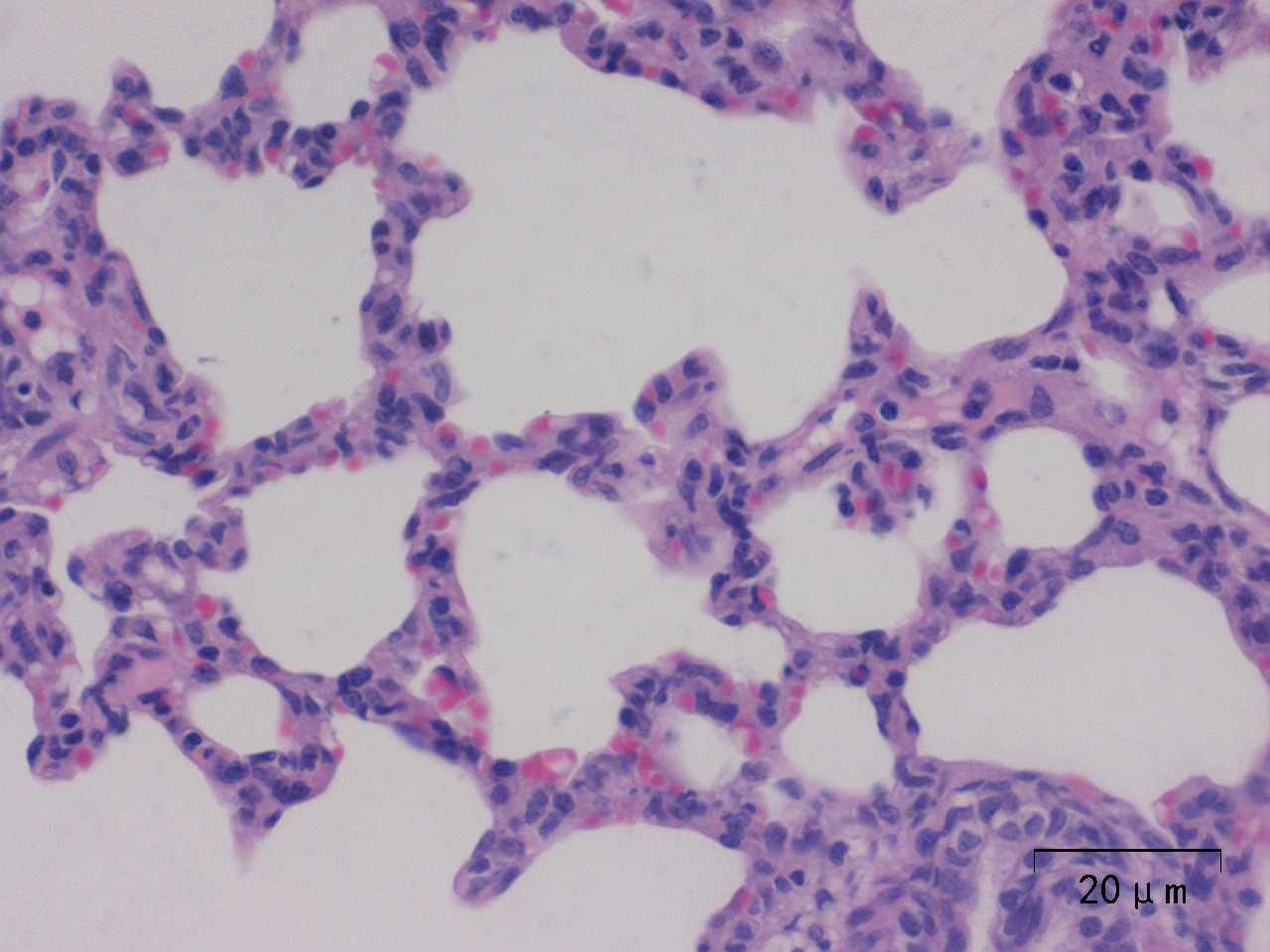

Supplement: S4 Data — (ZIP) [file ppat.1012546.s008.zip › Figure 8-10/Fig10/I/pig/Mock-lung/Mock-lung-1-1.jpg]

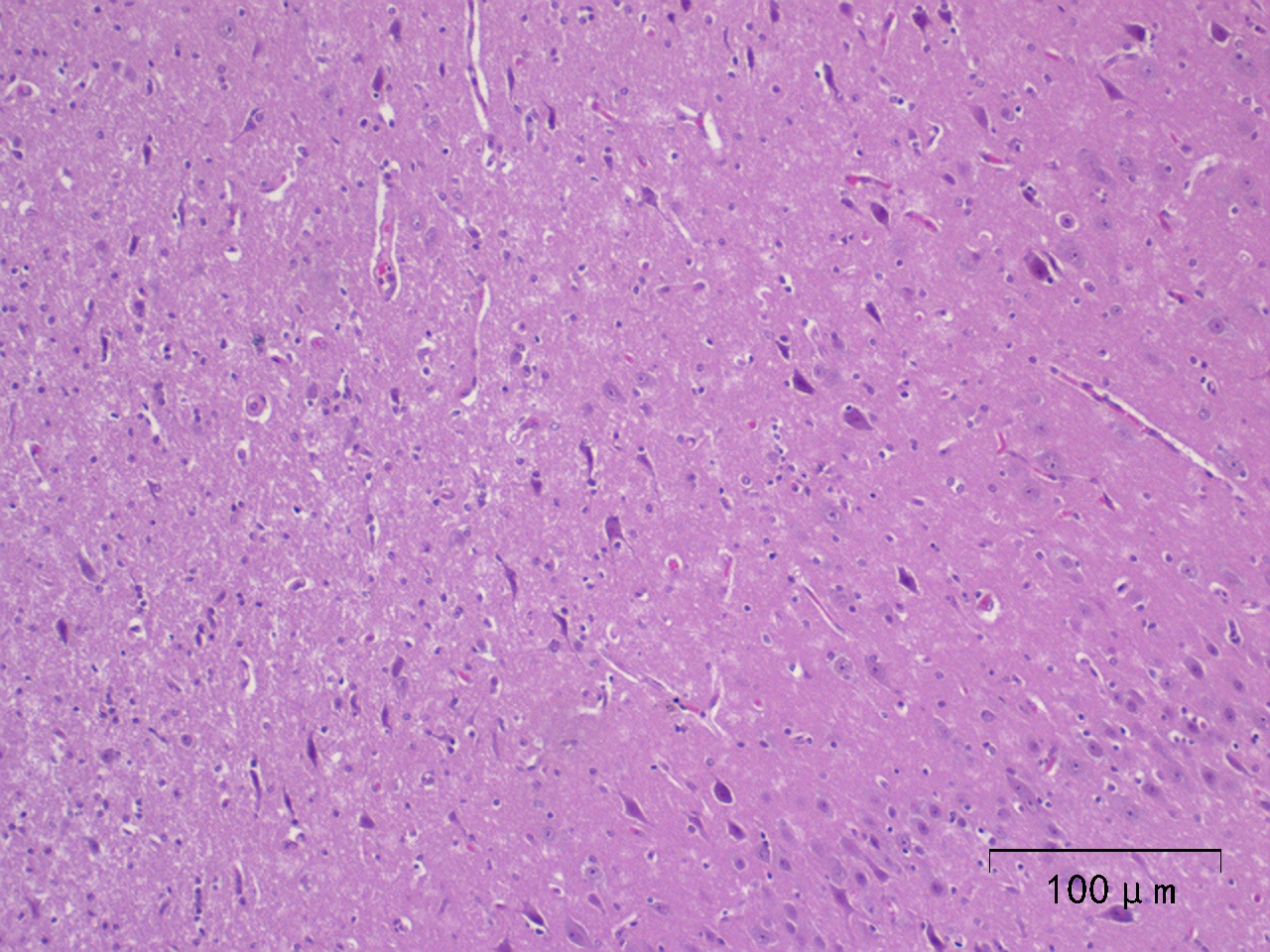

Supplement: S4 Data — (ZIP) [file ppat.1012546.s008.zip › Figure 8-10/Fig10/I/pig/PRV-Brain/25.jpg]

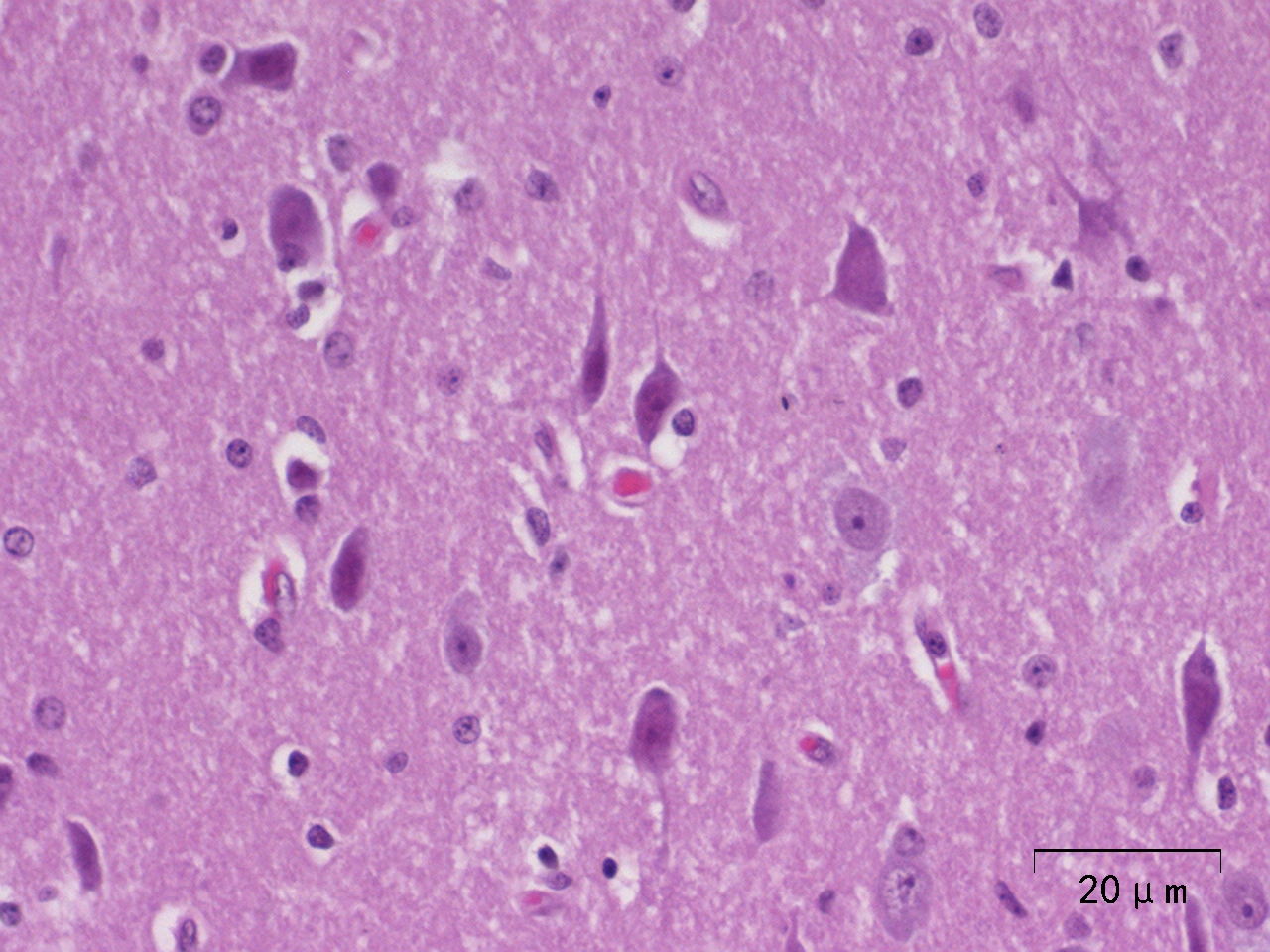

Supplement: S4 Data — (ZIP) [file ppat.1012546.s008.zip › Figure 8-10/Fig10/I/pig/PRV-Brain/26.jpg]

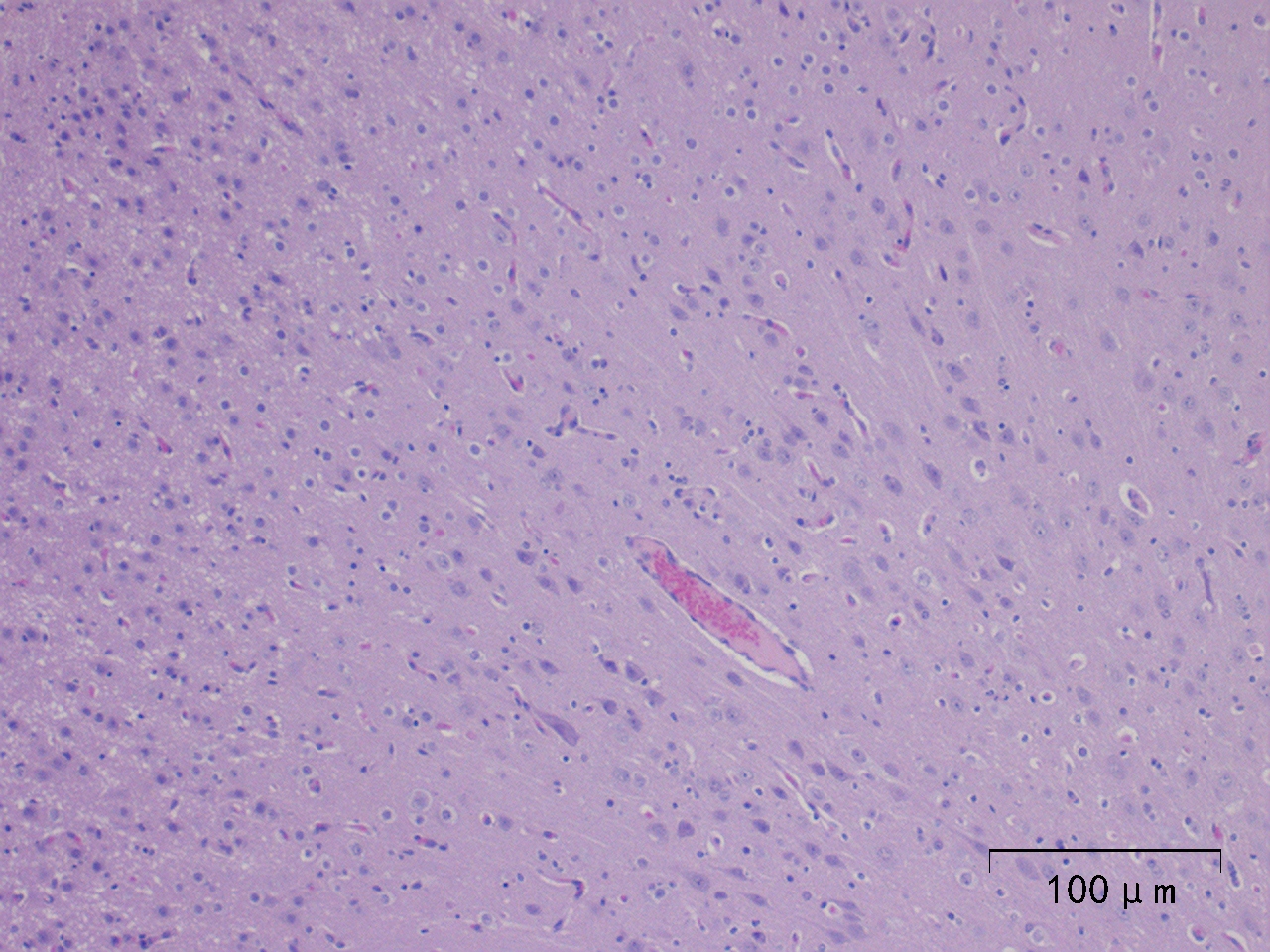

Supplement: S4 Data — (ZIP) [file ppat.1012546.s008.zip › Figure 8-10/Fig10/I/pig/PRV-Brain/27.jpg]

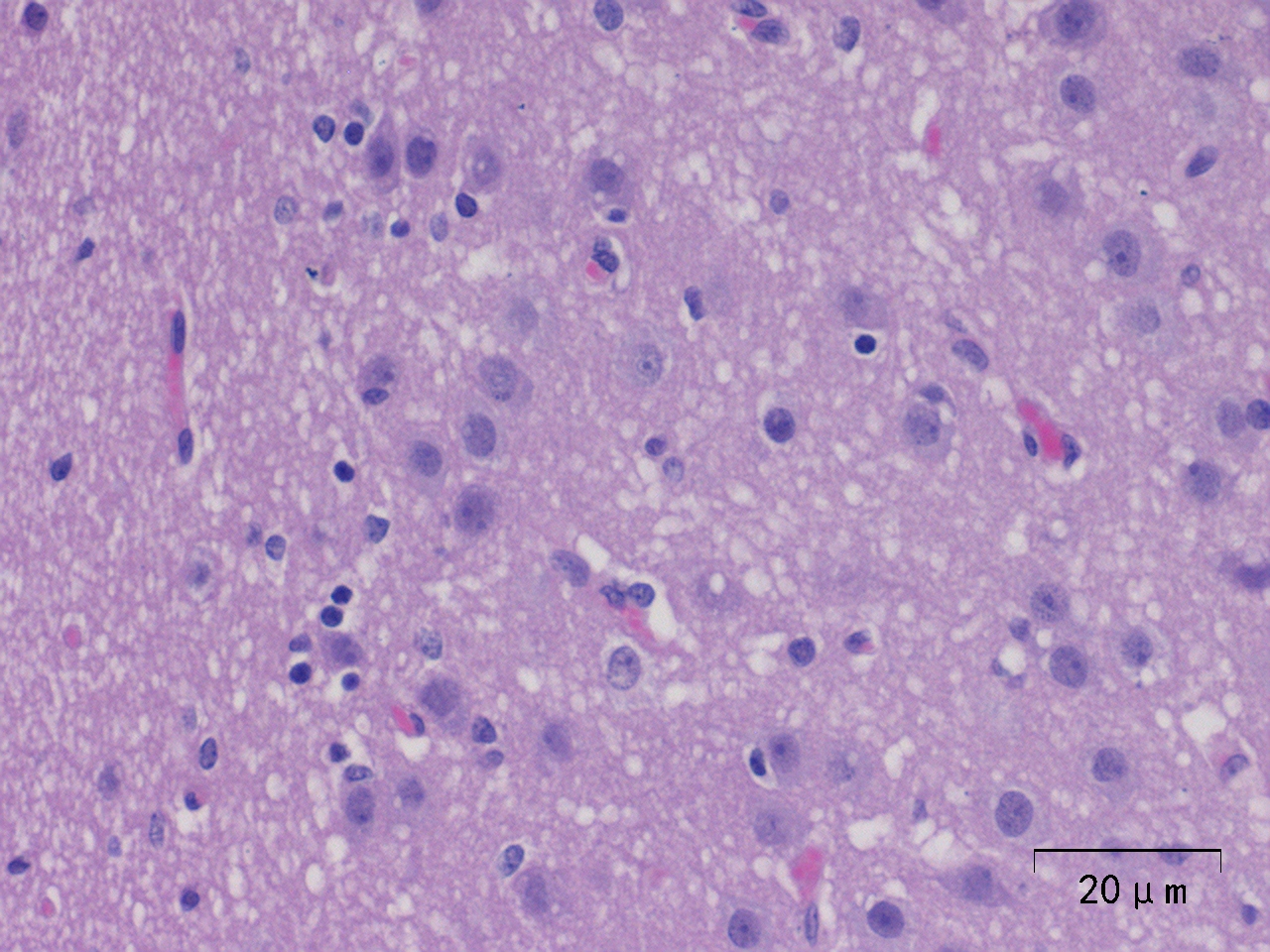

Supplement: S4 Data — (ZIP) [file ppat.1012546.s008.zip › Figure 8-10/Fig10/I/pig/PRV-Brain/28.jpg]

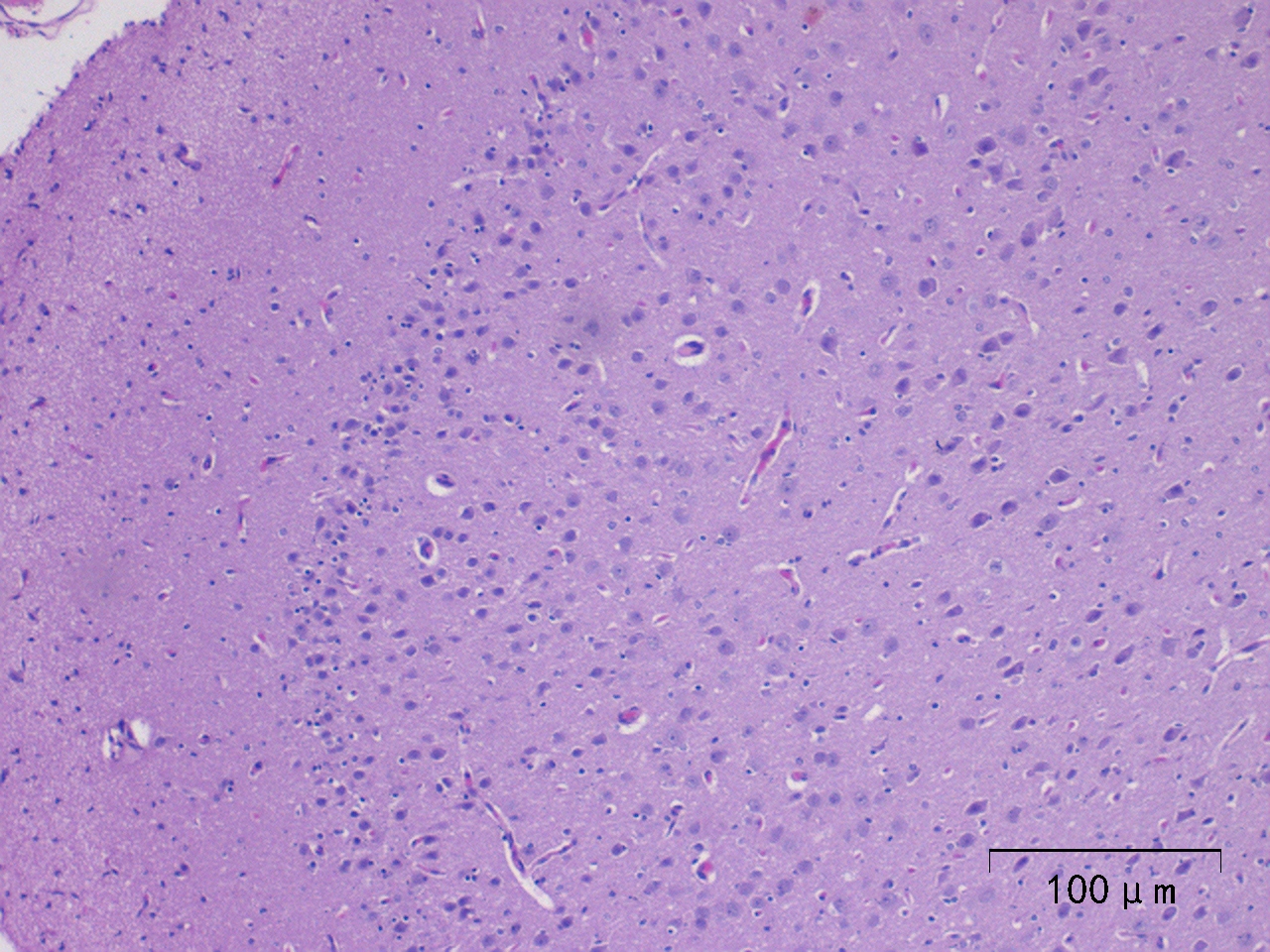

Supplement: S4 Data — (ZIP) [file ppat.1012546.s008.zip › Figure 8-10/Fig10/I/pig/PRV-Brain/29.jpg]

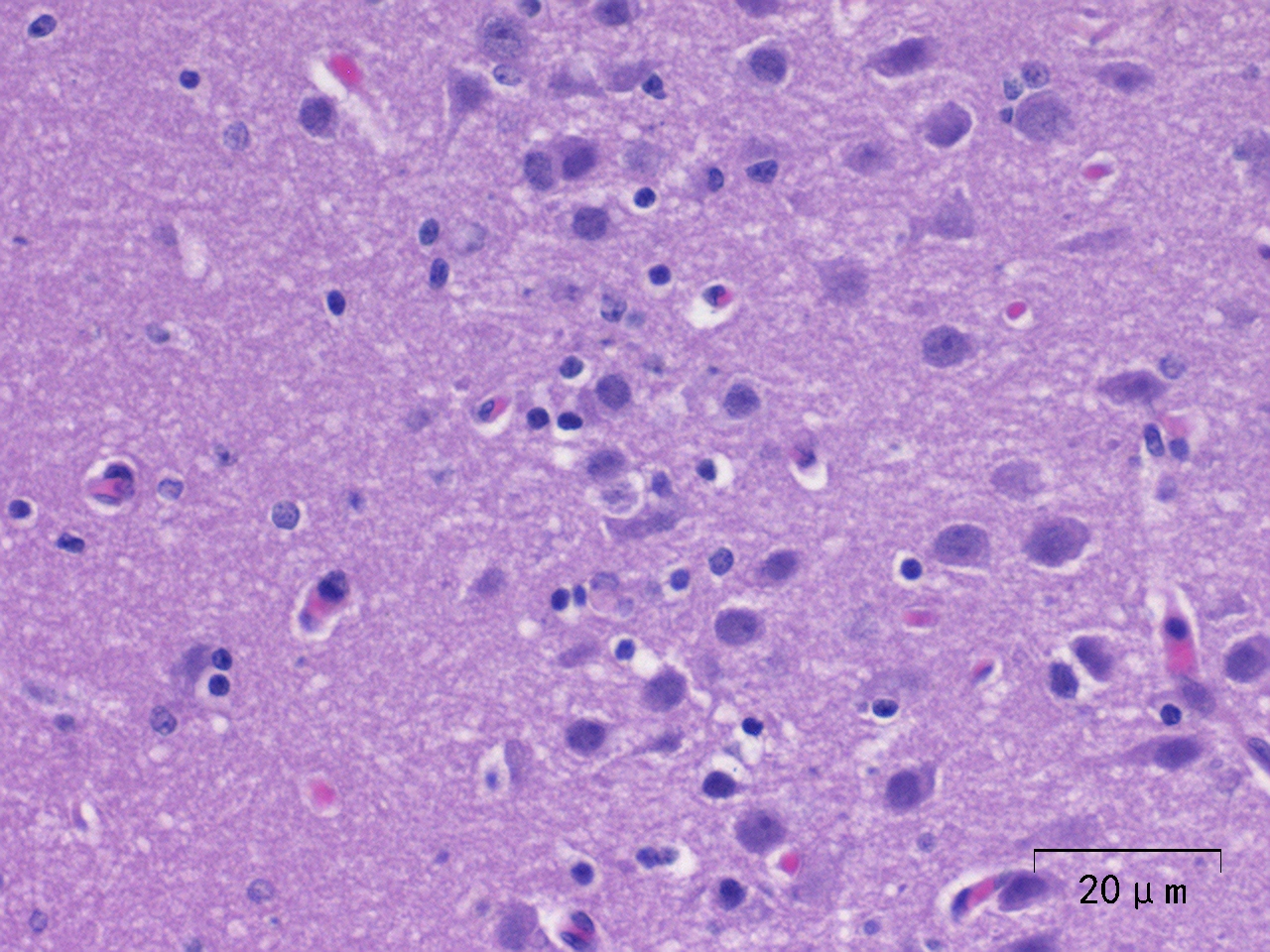

Supplement: S4 Data — (ZIP) [file ppat.1012546.s008.zip › Figure 8-10/Fig10/I/pig/PRV-Brain/30.jpg]

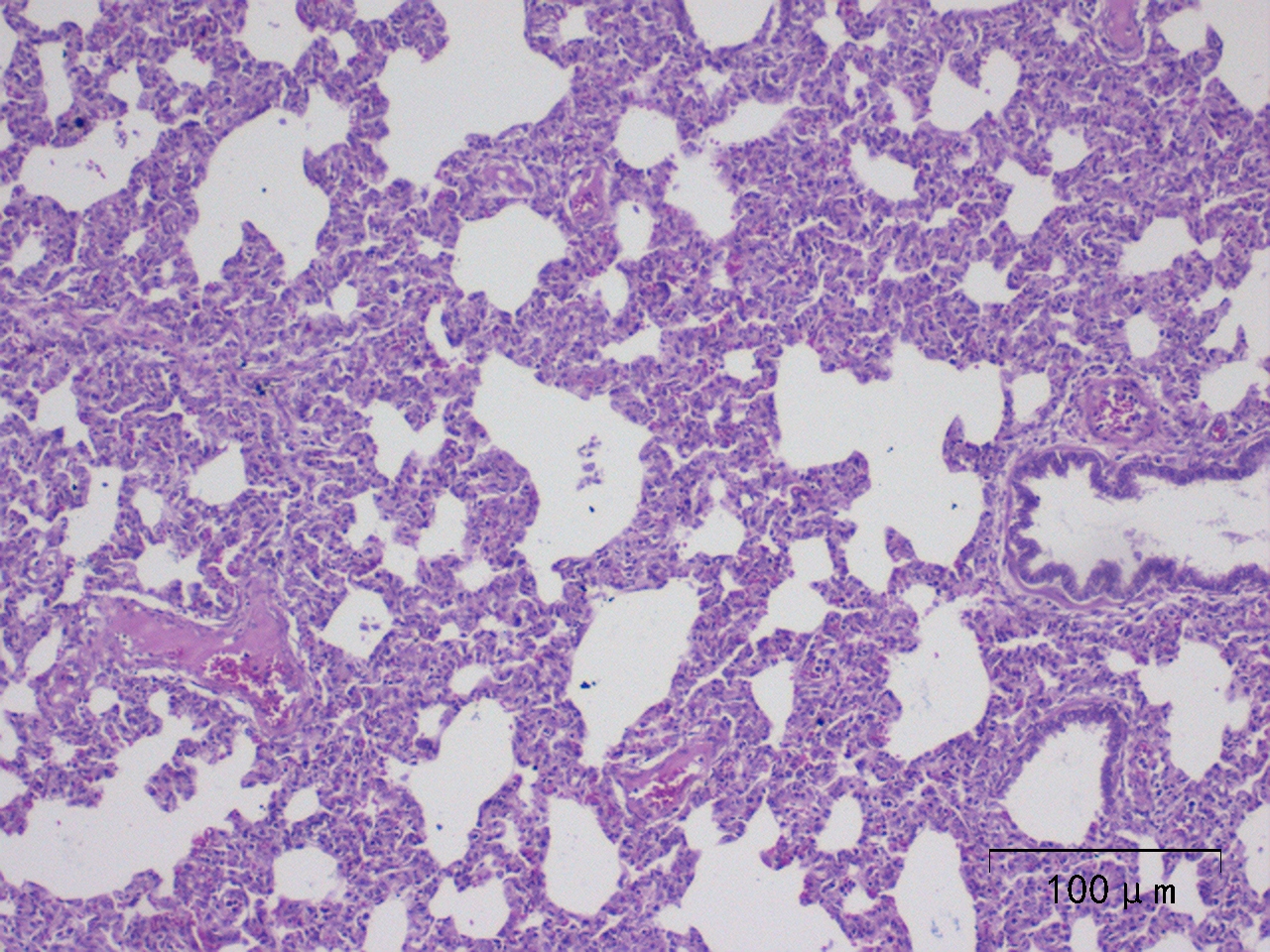

Supplement: S4 Data — (ZIP) [file ppat.1012546.s008.zip › Figure 8-10/Fig10/I/pig/PRV-lung/13.jpg]

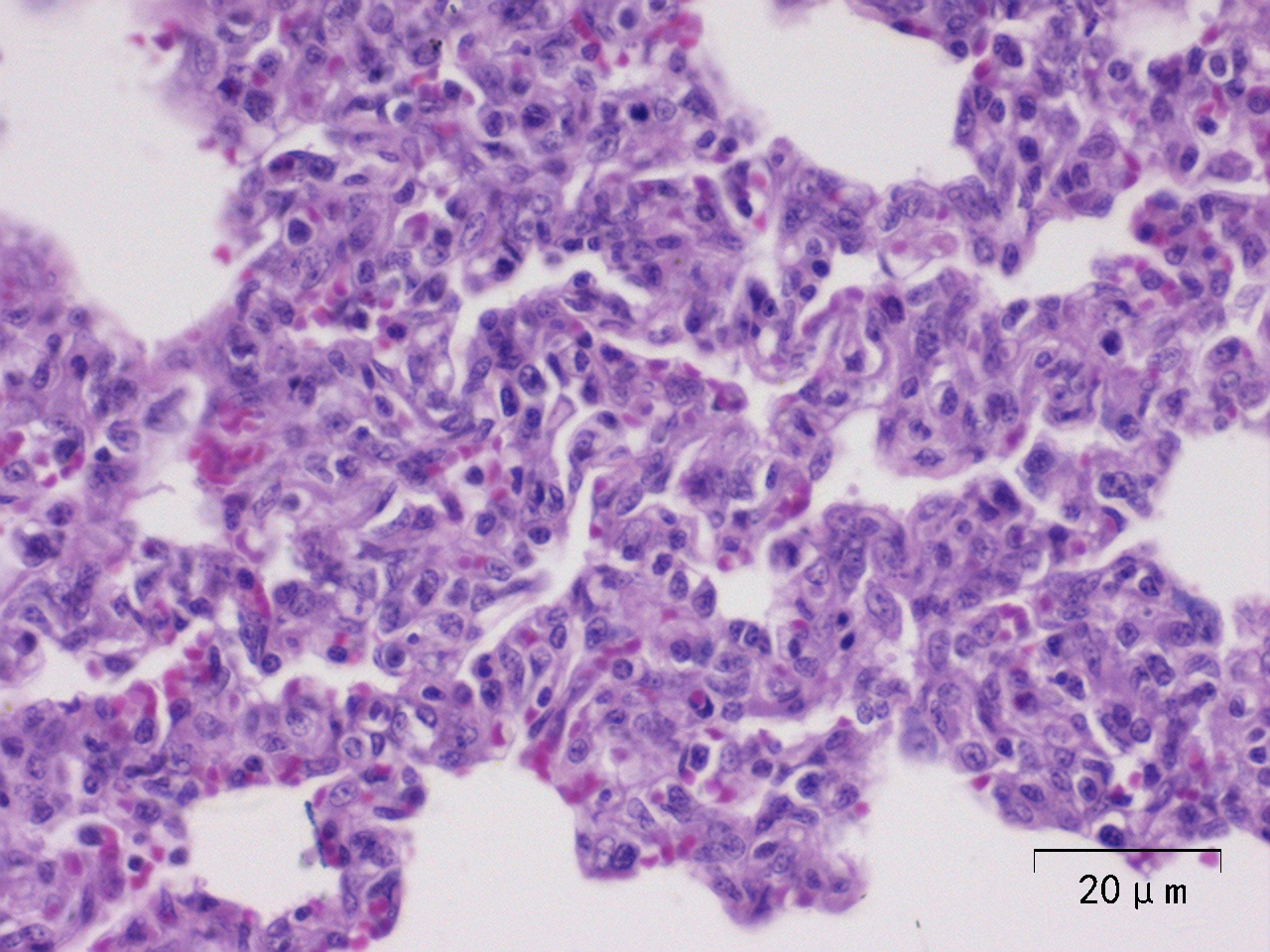

Supplement: S4 Data — (ZIP) [file ppat.1012546.s008.zip › Figure 8-10/Fig10/I/pig/PRV-lung/14.jpg]

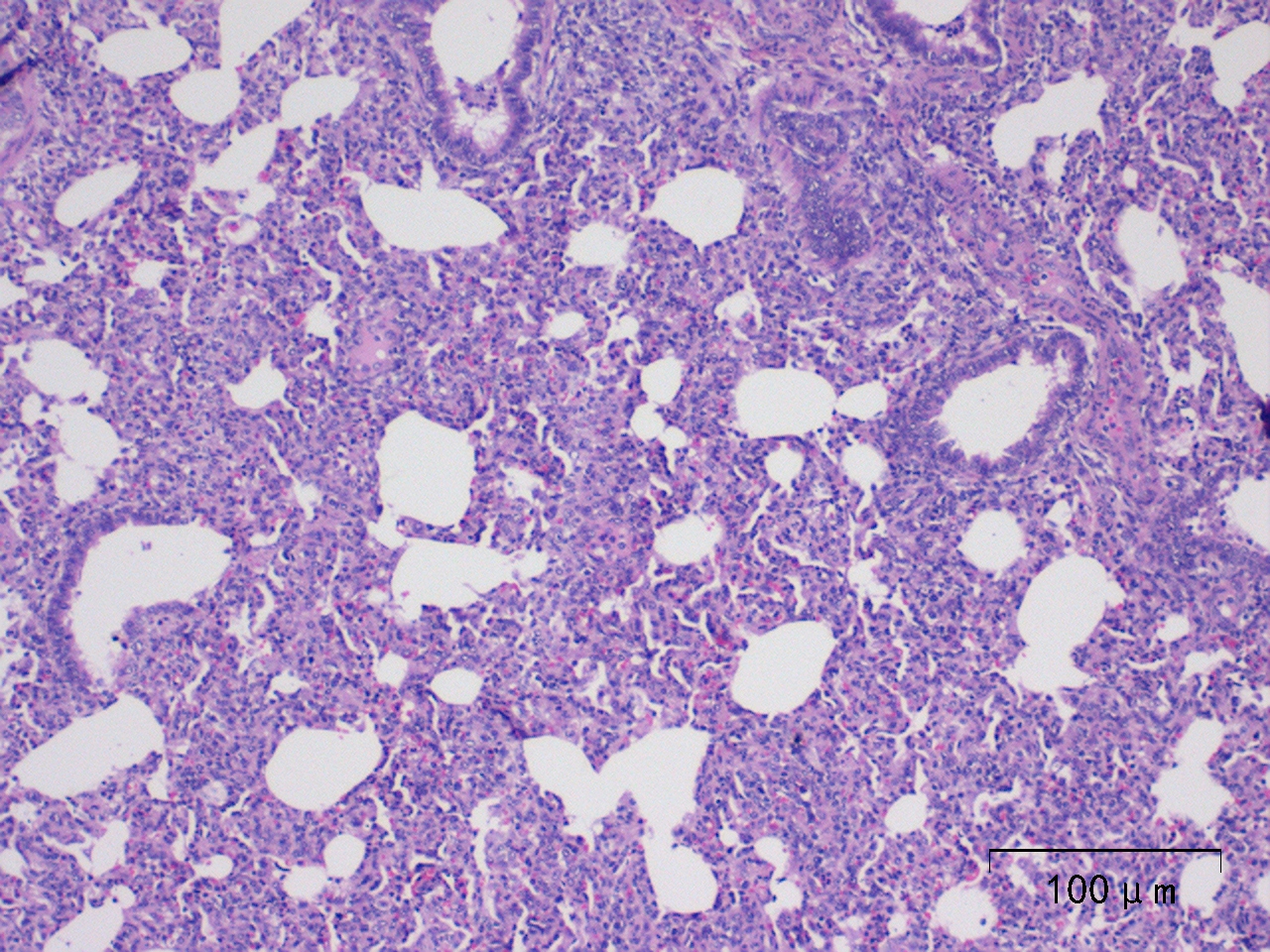

Supplement: S4 Data — (ZIP) [file ppat.1012546.s008.zip › Figure 8-10/Fig10/I/pig/PRV-lung/15.jpg]

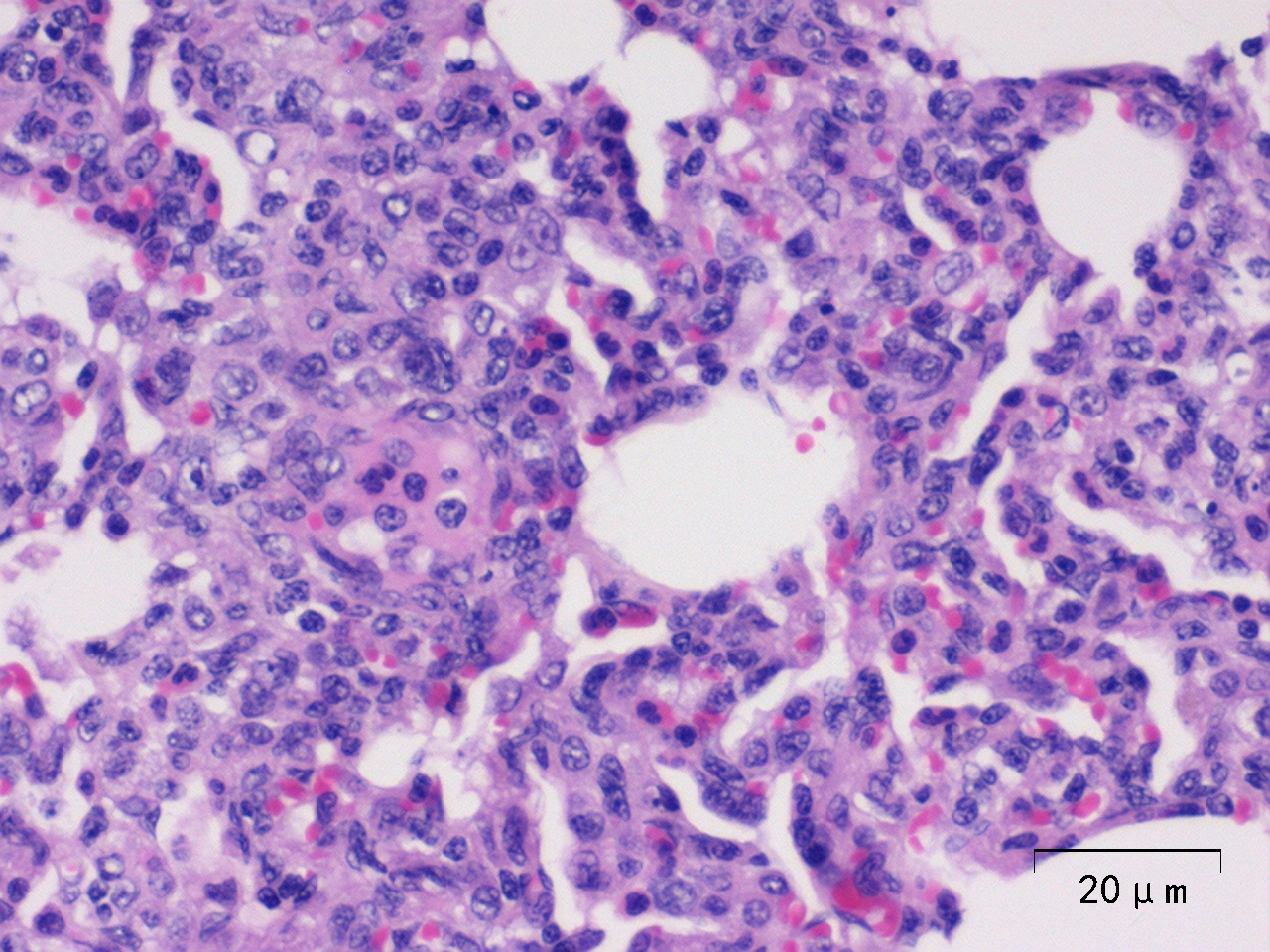

Supplement: S4 Data — (ZIP) [file ppat.1012546.s008.zip › Figure 8-10/Fig10/I/pig/PRV-lung/16.jpg]

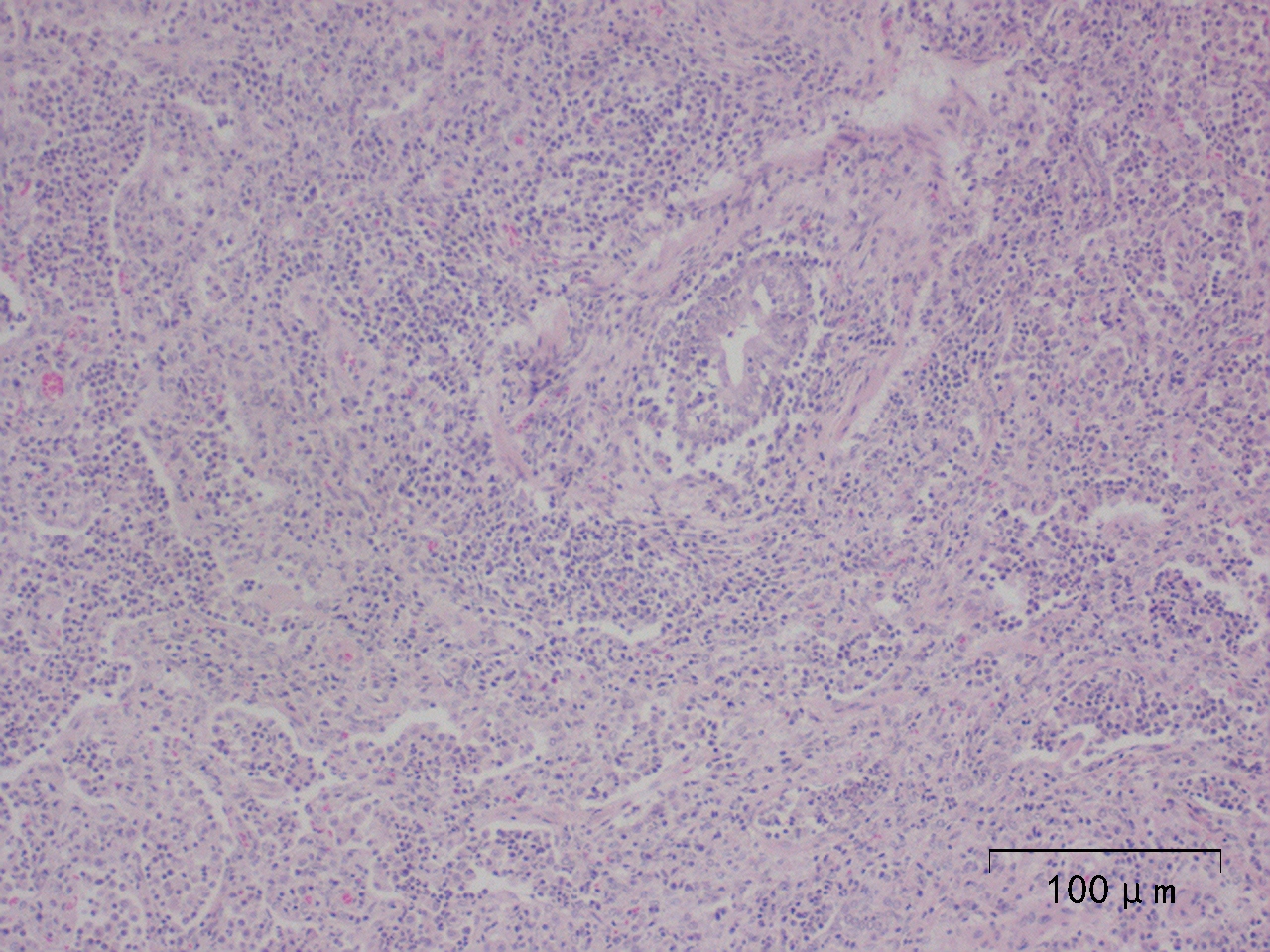

Supplement: S4 Data — (ZIP) [file ppat.1012546.s008.zip › Figure 8-10/Fig10/I/pig/PRV-lung/17.jpg]

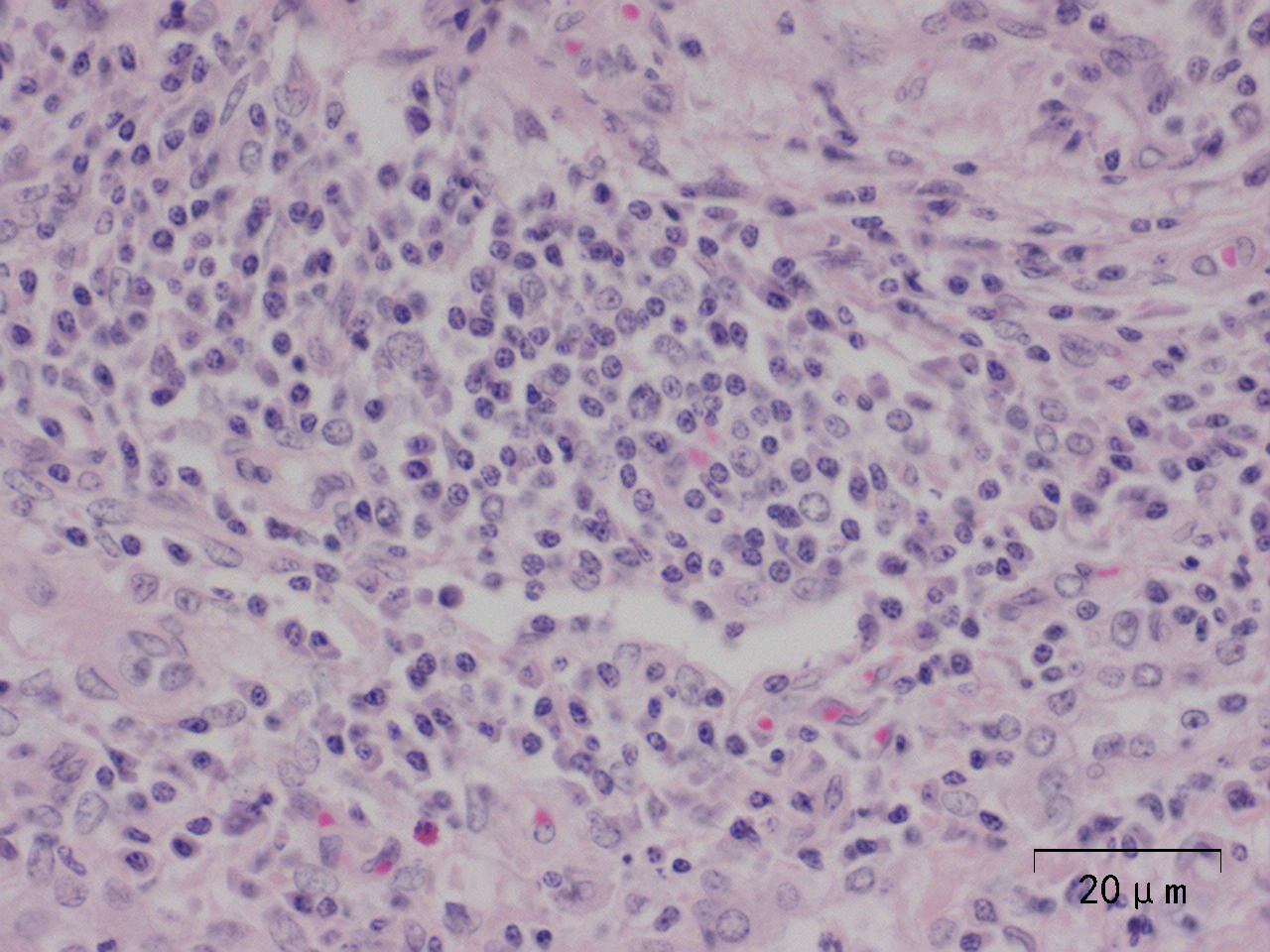

Supplement: S4 Data — (ZIP) [file ppat.1012546.s008.zip › Figure 8-10/Fig10/I/pig/PRV-lung/18.jpg]

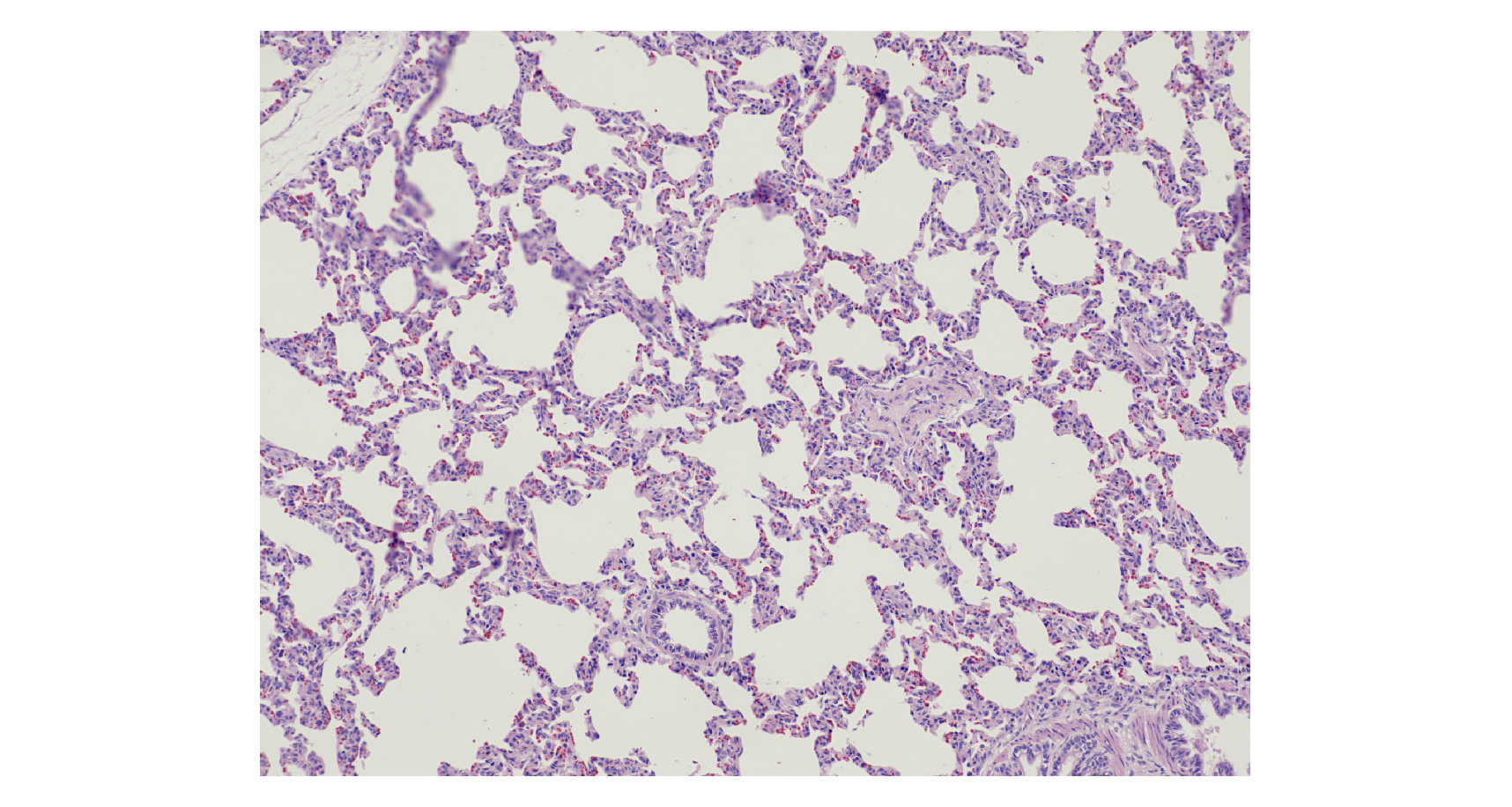

Supplement: S4 Data — (ZIP) [file ppat.1012546.s008.zip › Figure 8-10/Fig10/I/pig/PRV-lung/PRV-f1-10x-1.tif]

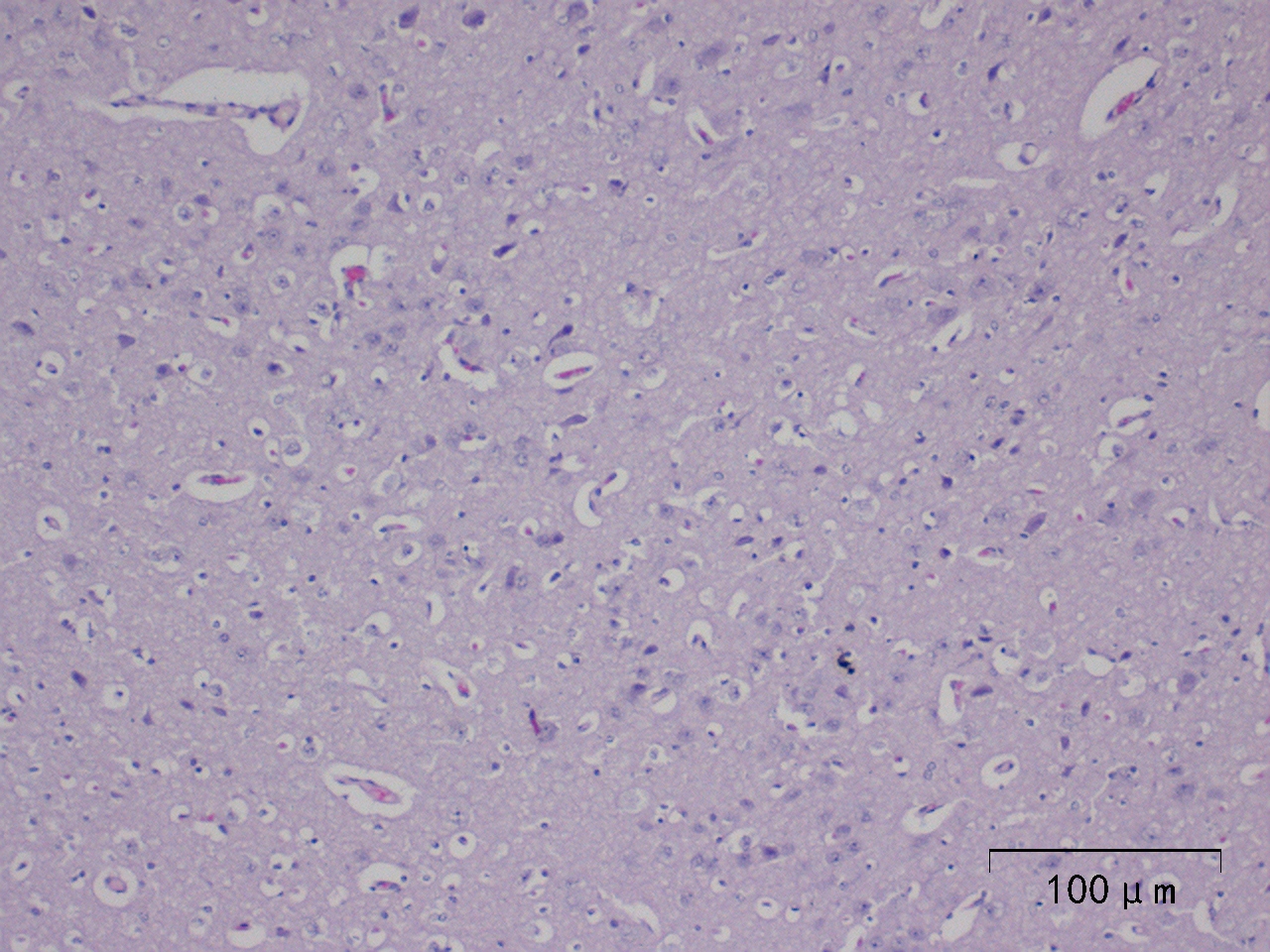

Supplement: S4 Data — (ZIP) [file ppat.1012546.s008.zip › Figure 8-10/Fig10/I/pig/PRV-UL4mut-Brain/31.jpg]

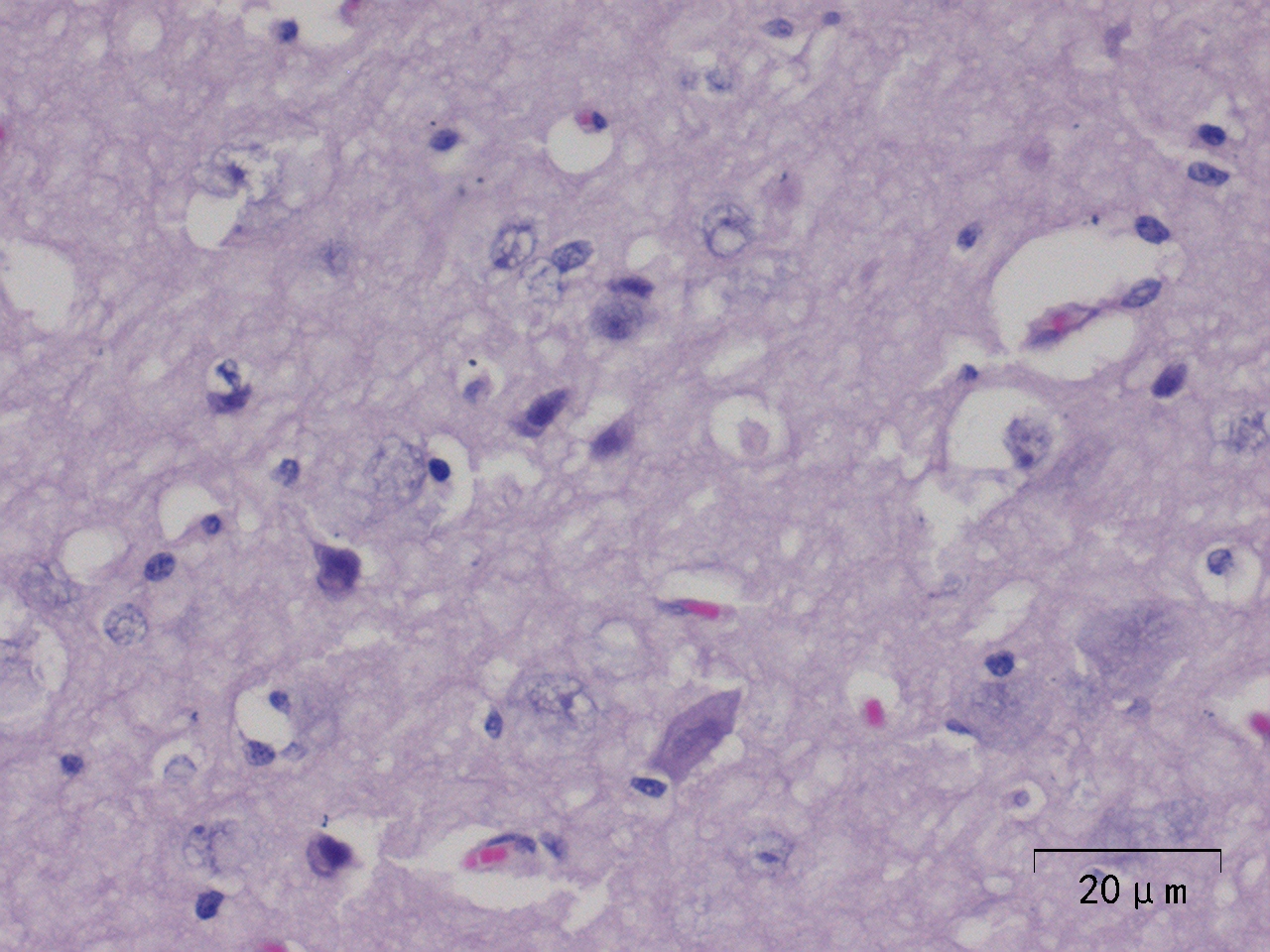

Supplement: S4 Data — (ZIP) [file ppat.1012546.s008.zip › Figure 8-10/Fig10/I/pig/PRV-UL4mut-Brain/32.jpg]

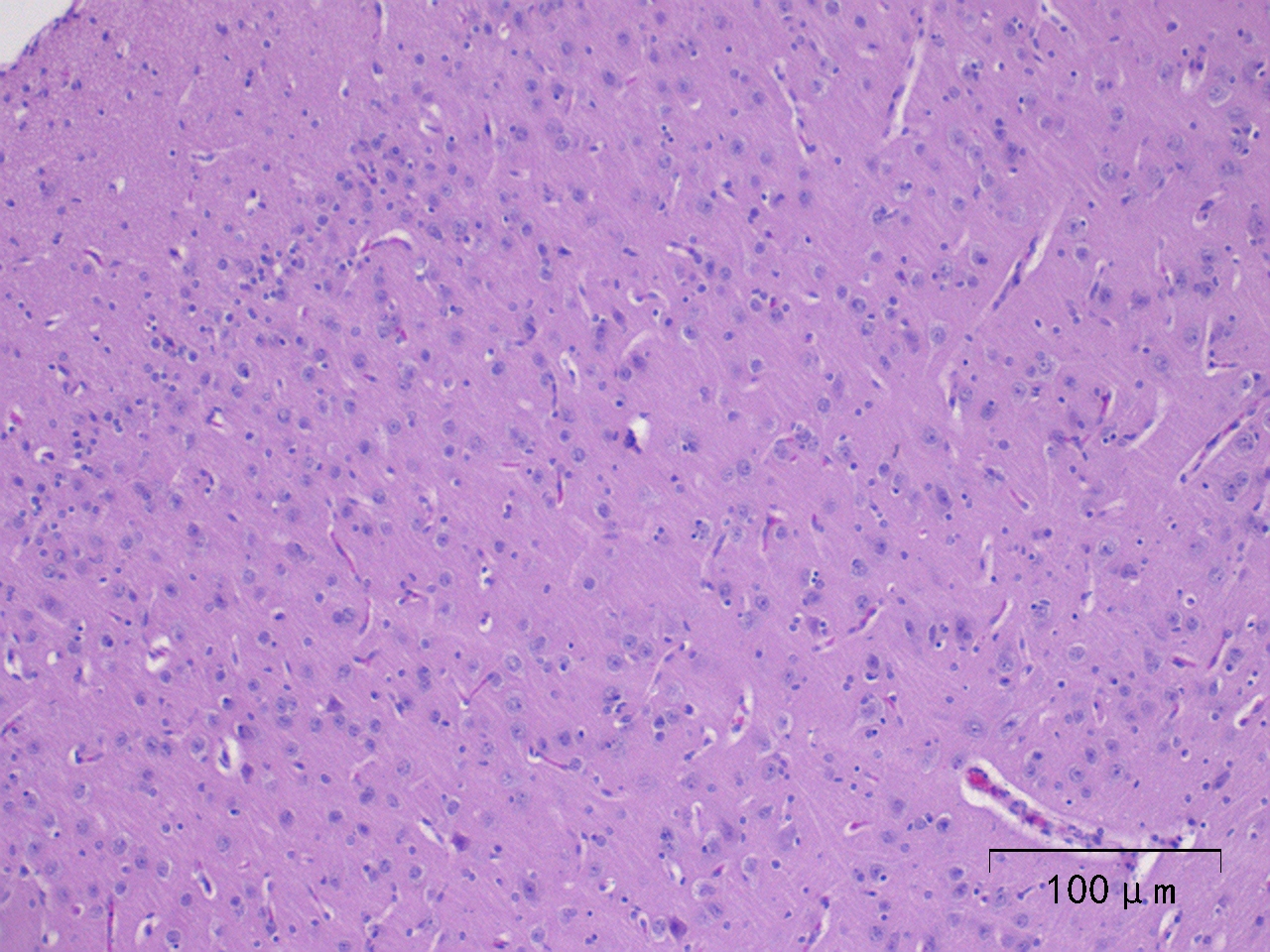

Supplement: S4 Data — (ZIP) [file ppat.1012546.s008.zip › Figure 8-10/Fig10/I/pig/PRV-UL4mut-Brain/33.jpg]

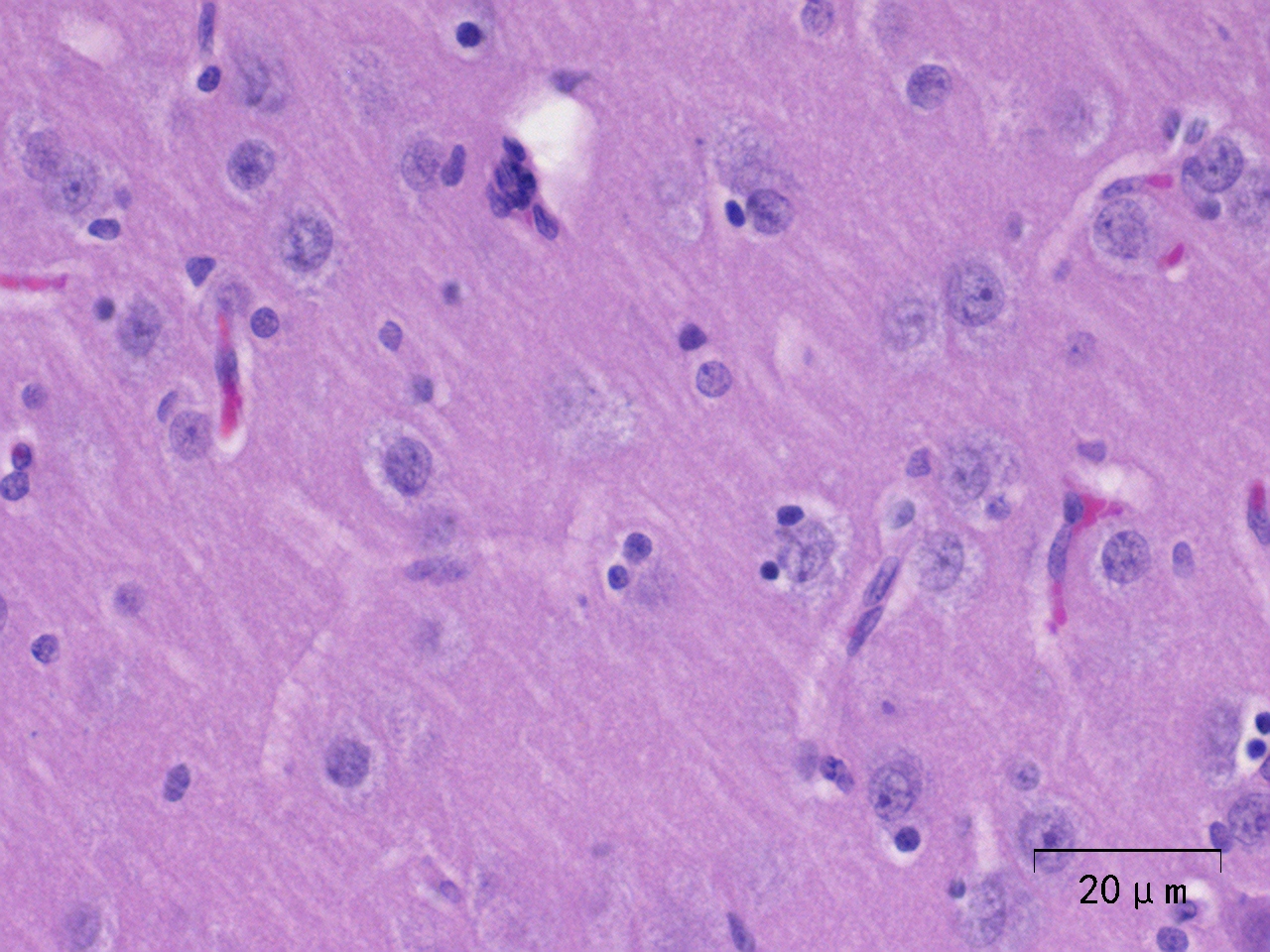

Supplement: S4 Data — (ZIP) [file ppat.1012546.s008.zip › Figure 8-10/Fig10/I/pig/PRV-UL4mut-Brain/34.jpg]

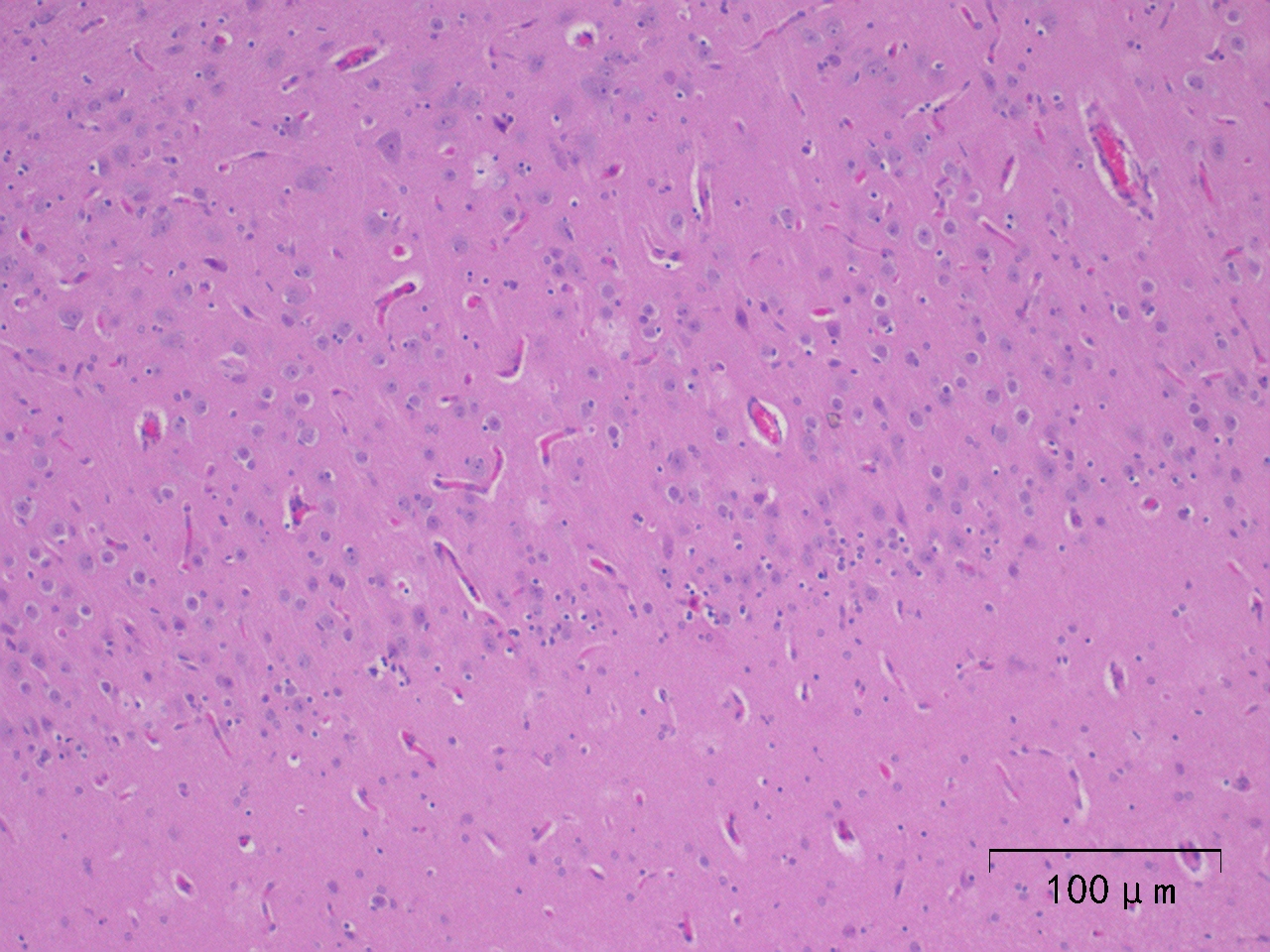

Supplement: S4 Data — (ZIP) [file ppat.1012546.s008.zip › Figure 8-10/Fig10/I/pig/PRV-UL4mut-Brain/35.jpg]

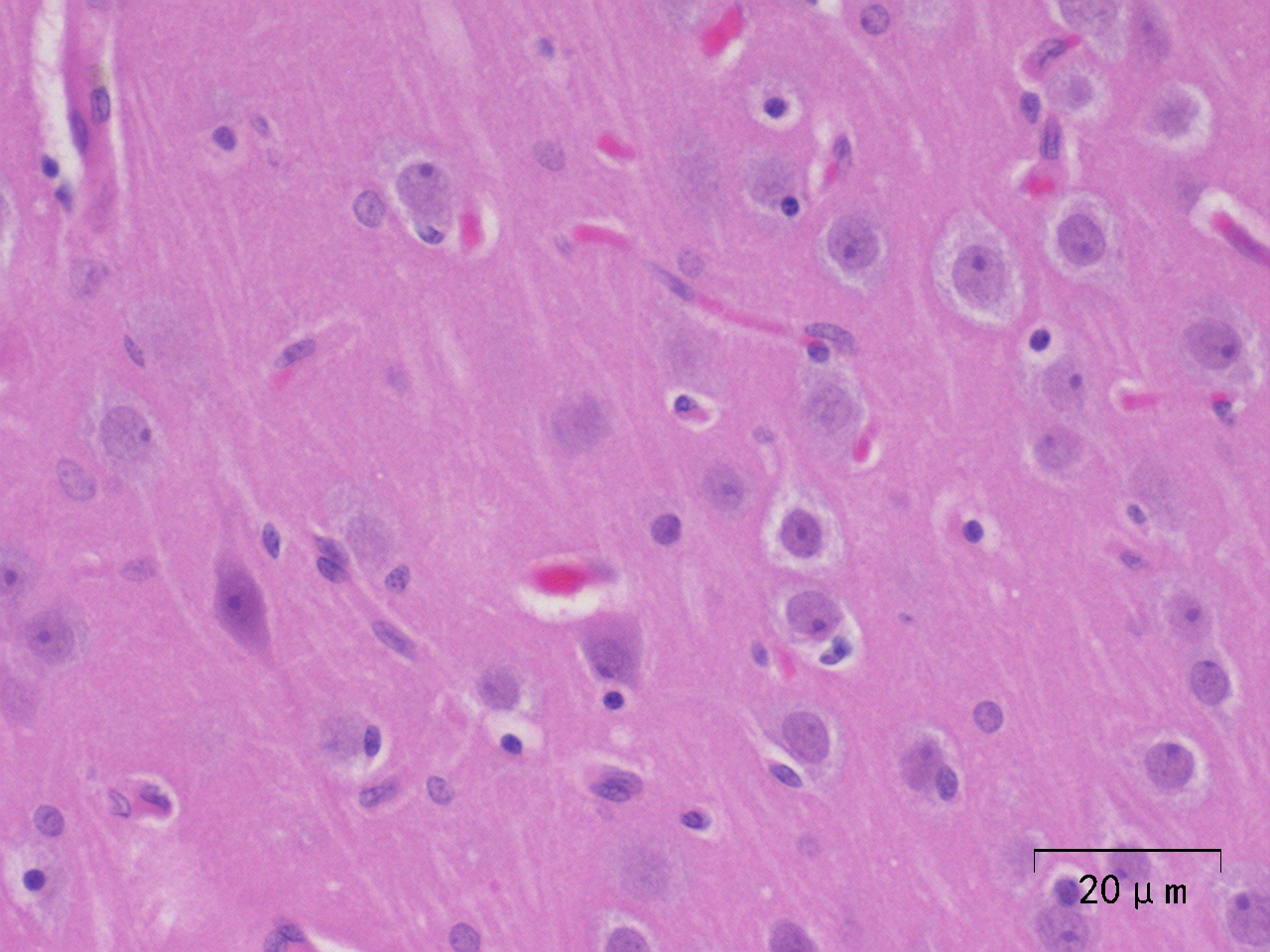

Supplement: S4 Data — (ZIP) [file ppat.1012546.s008.zip › Figure 8-10/Fig10/I/pig/PRV-UL4mut-Brain/36.jpg]

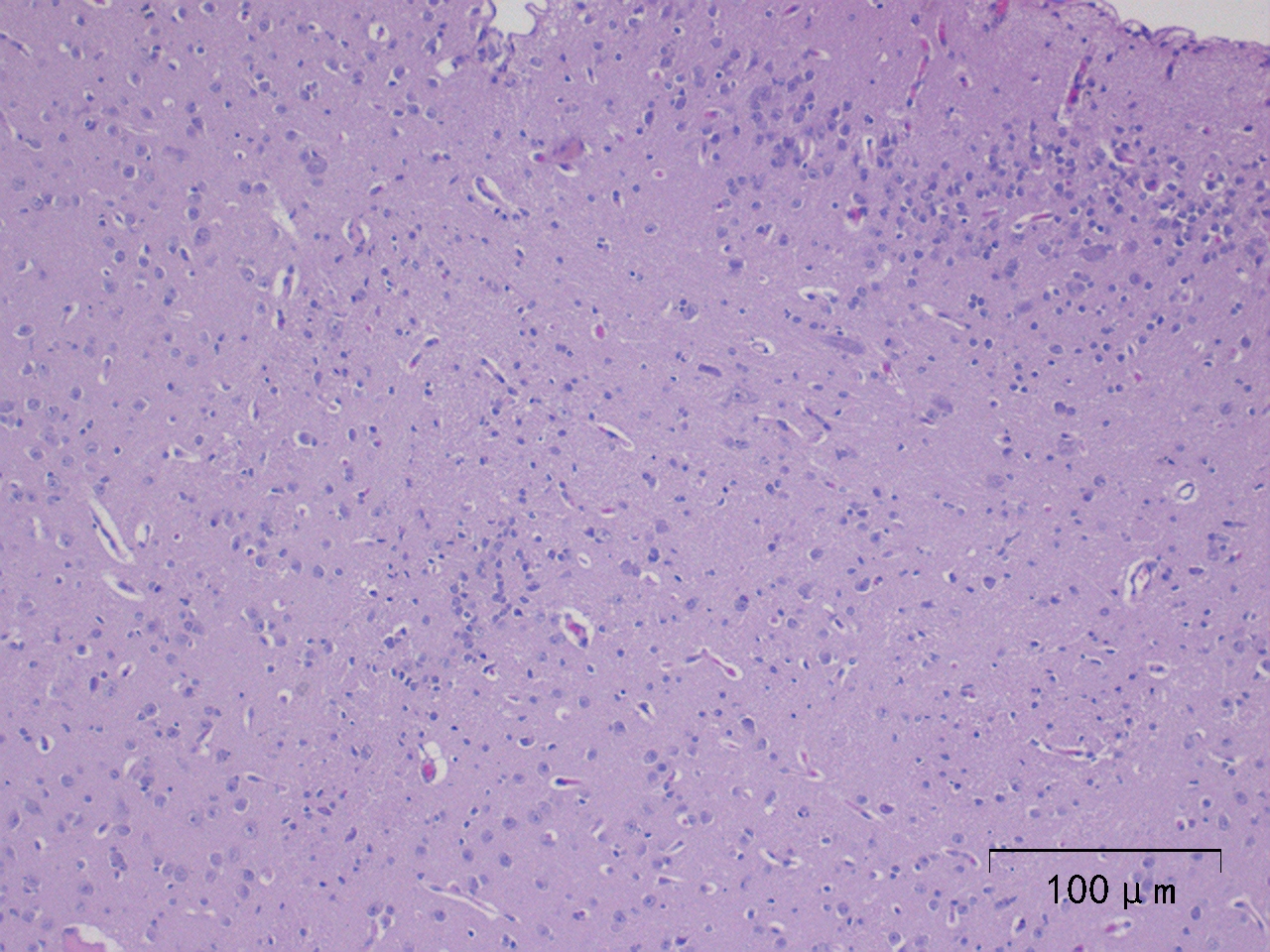

Supplement: S4 Data — (ZIP) [file ppat.1012546.s008.zip › Figure 8-10/Fig10/I/pig/PRV-UL4mut-Brain/37.jpg]

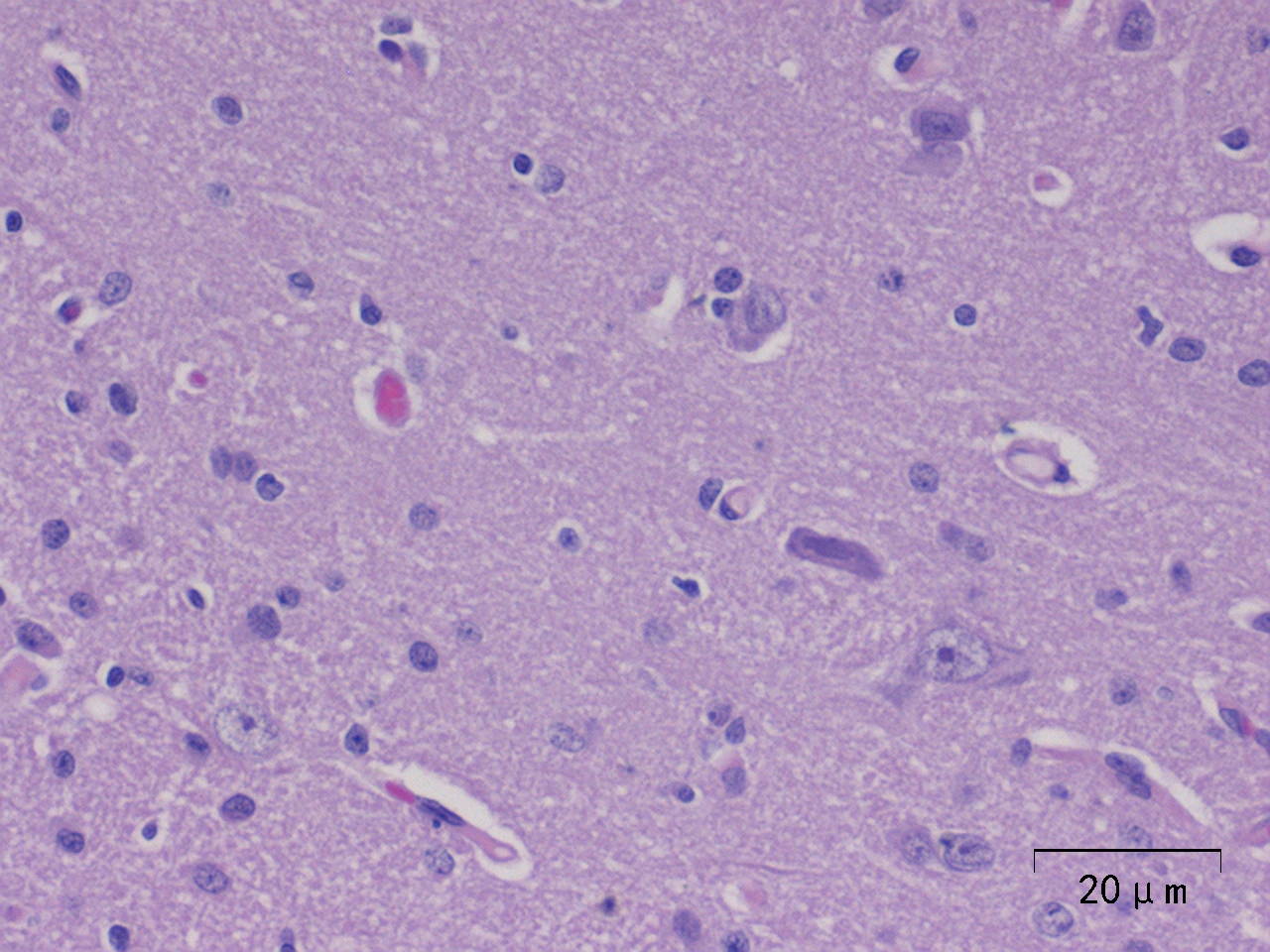

Supplement: S4 Data — (ZIP) [file ppat.1012546.s008.zip › Figure 8-10/Fig10/I/pig/PRV-UL4mut-Brain/38.jpg]

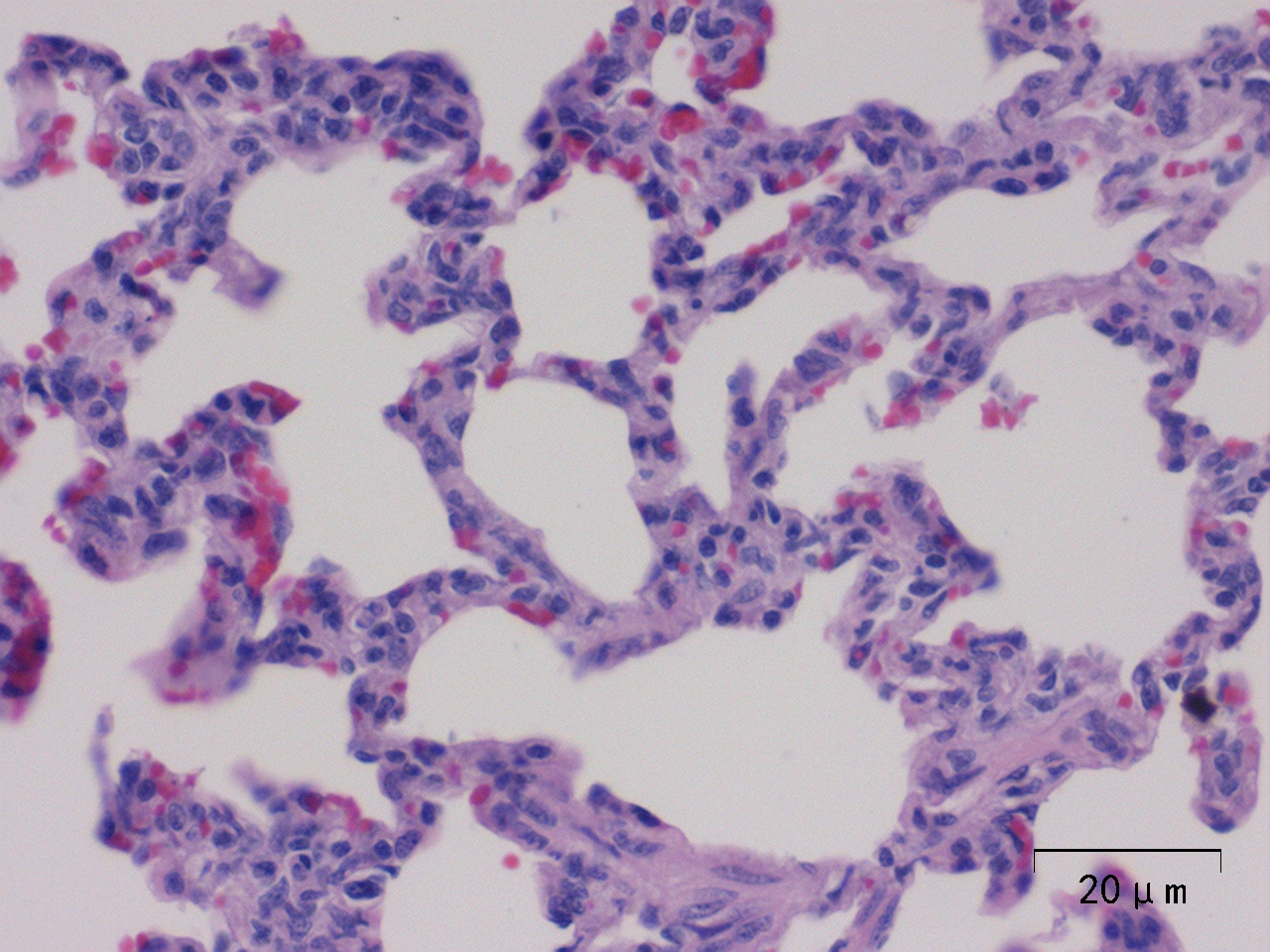

Supplement: S4 Data — (ZIP) [file ppat.1012546.s008.zip › Figure 8-10/Fig10/I/pig/PRV-UL4mut-lung/10.jpg]

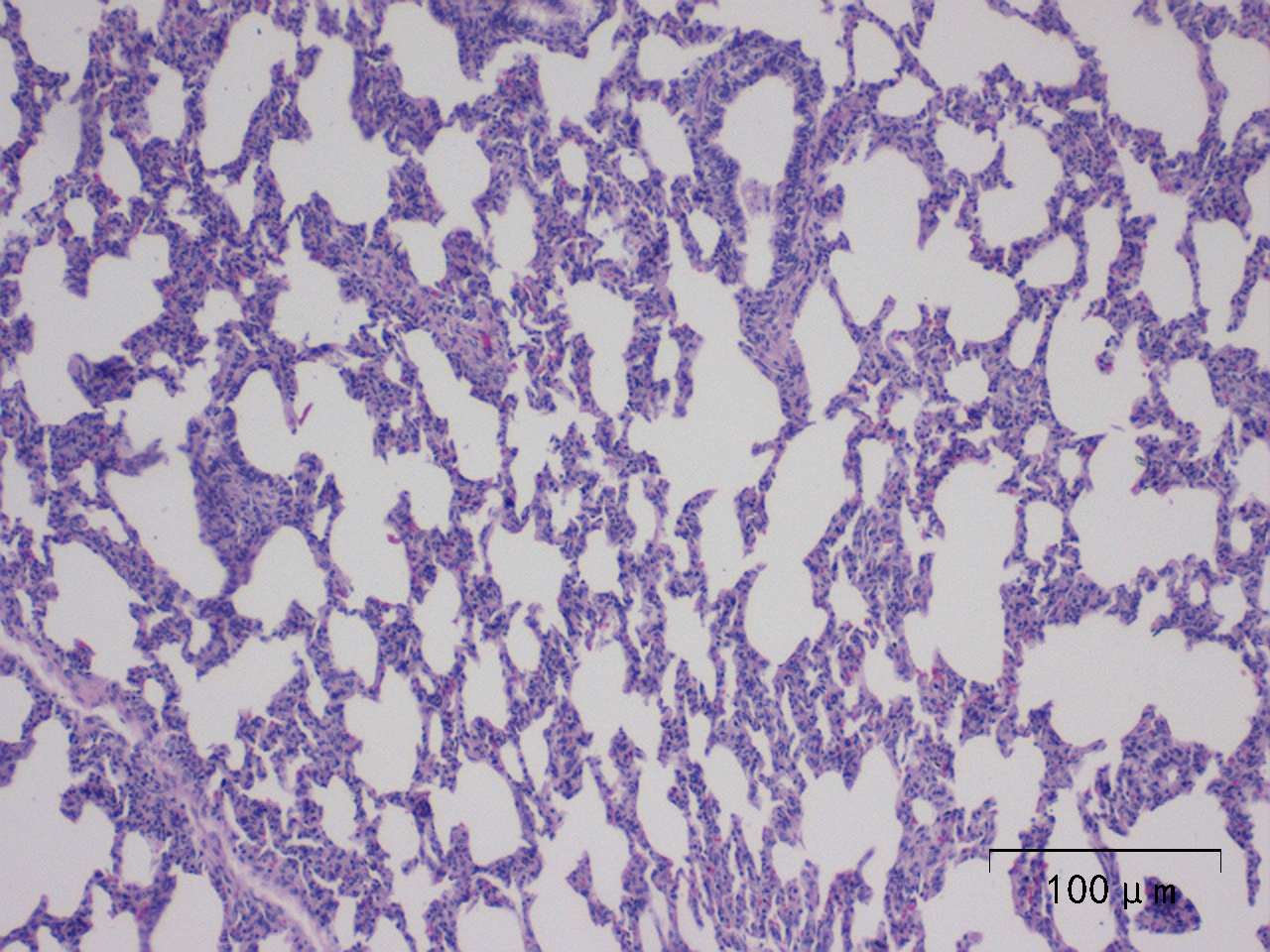

Supplement: S4 Data — (ZIP) [file ppat.1012546.s008.zip › Figure 8-10/Fig10/I/pig/PRV-UL4mut-lung/11.jpg]

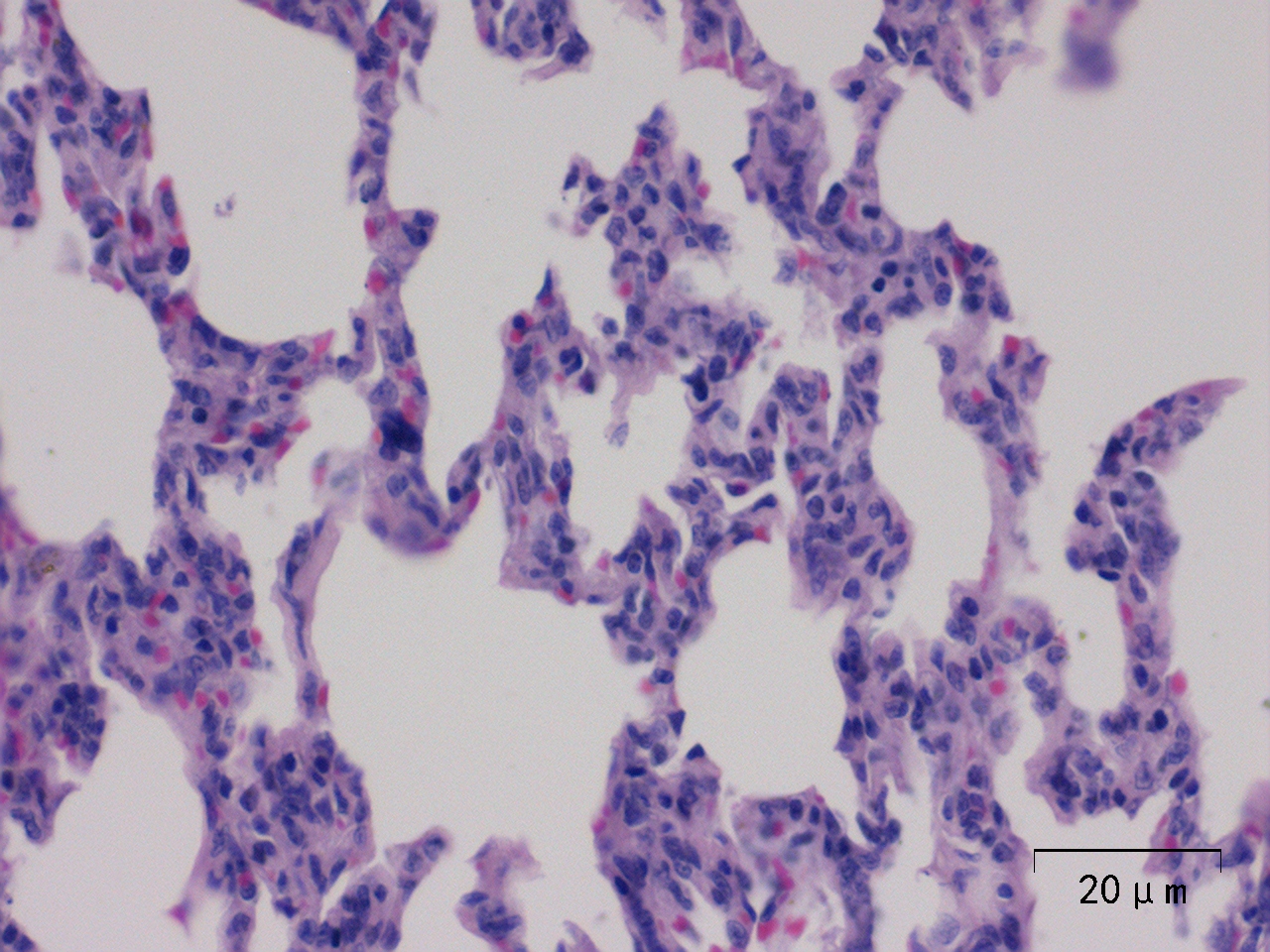

Supplement: S4 Data — (ZIP) [file ppat.1012546.s008.zip › Figure 8-10/Fig10/I/pig/PRV-UL4mut-lung/12.jpg]

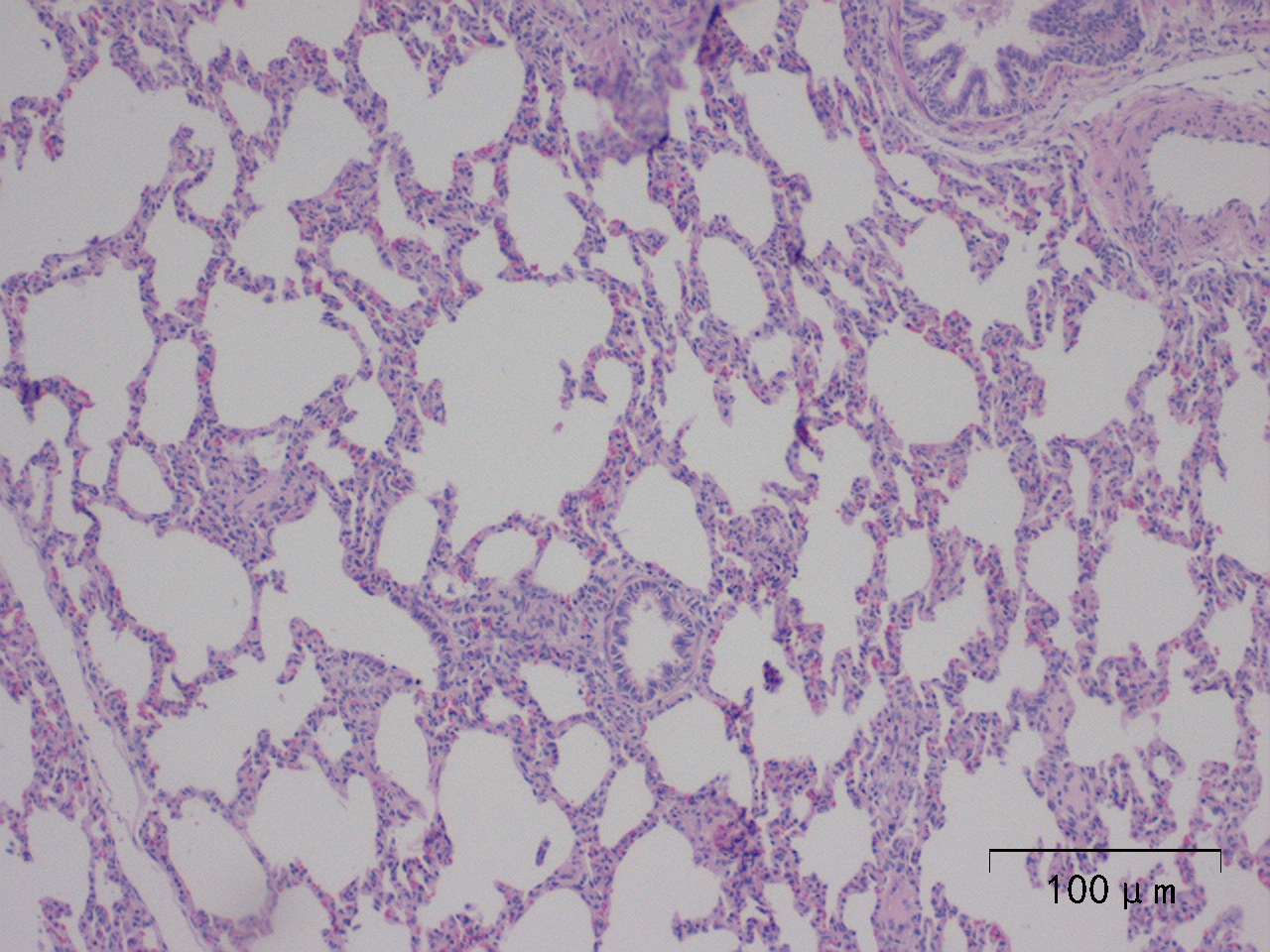

Supplement: S4 Data — (ZIP) [file ppat.1012546.s008.zip › Figure 8-10/Fig10/I/pig/PRV-UL4mut-lung/7.jpg]

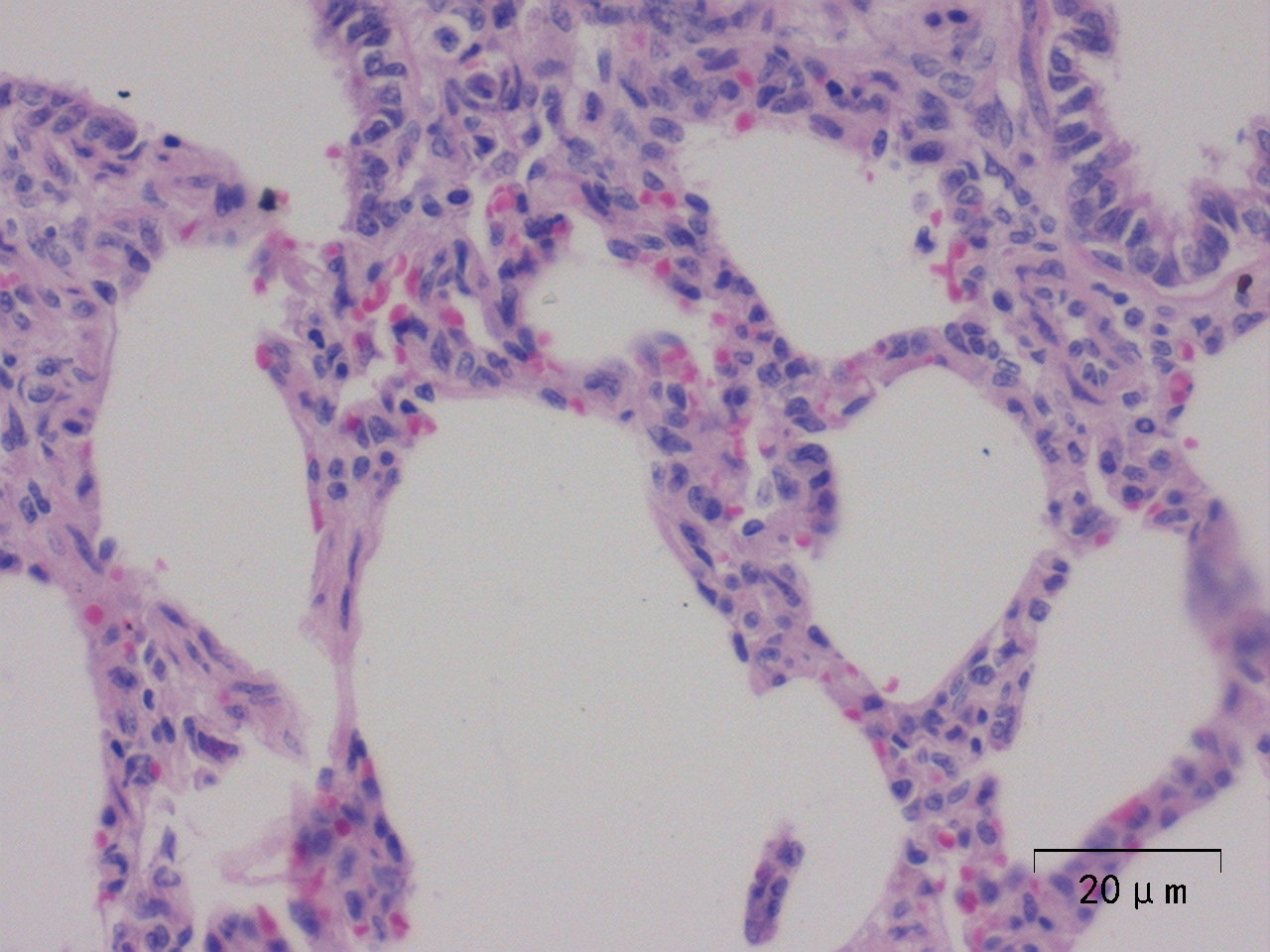

Supplement: S4 Data — (ZIP) [file ppat.1012546.s008.zip › Figure 8-10/Fig10/I/pig/PRV-UL4mut-lung/8.jpg]

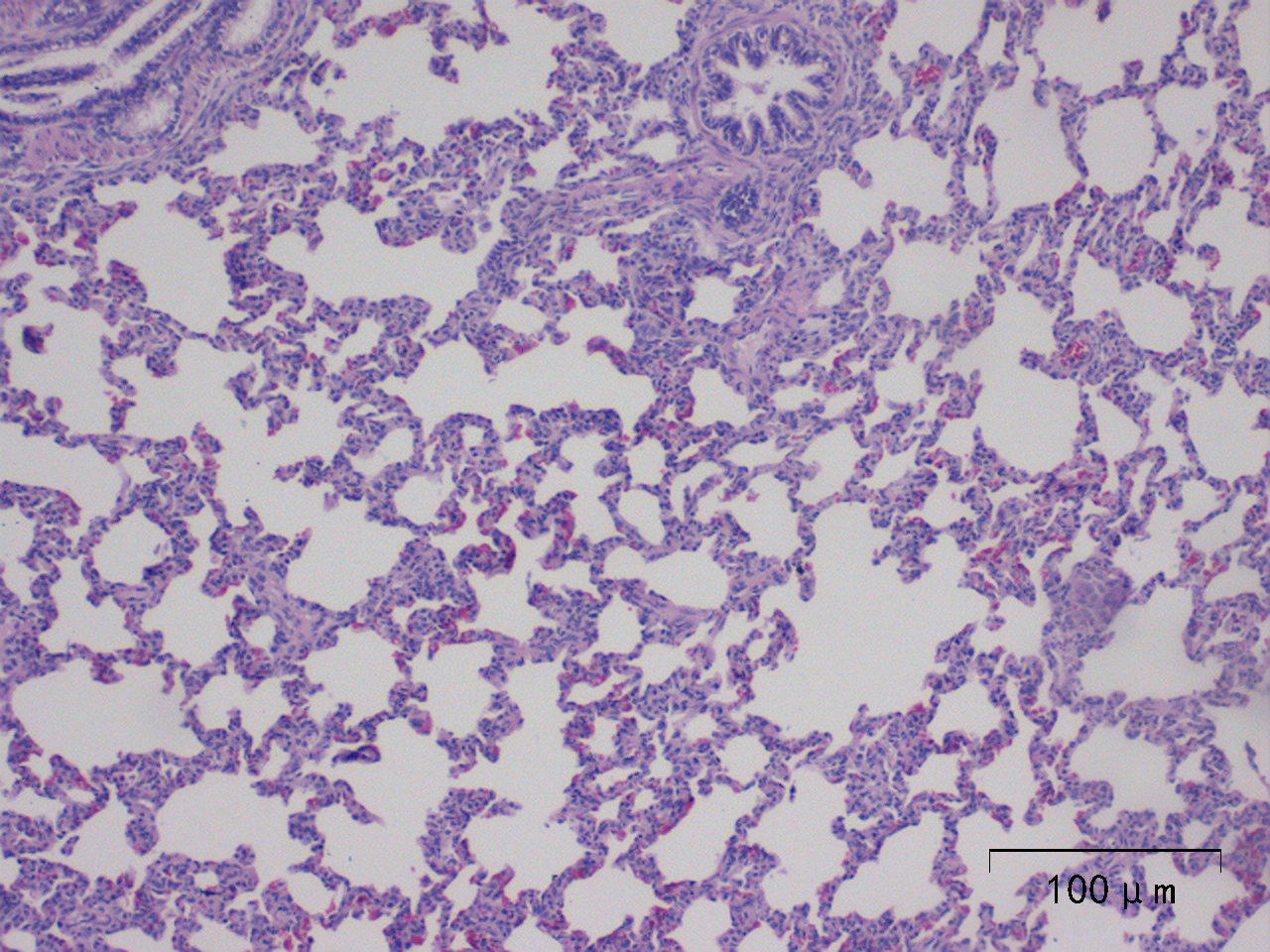

Supplement: S4 Data — (ZIP) [file ppat.1012546.s008.zip › Figure 8-10/Fig10/I/pig/PRV-UL4mut-lung/9.jpg]

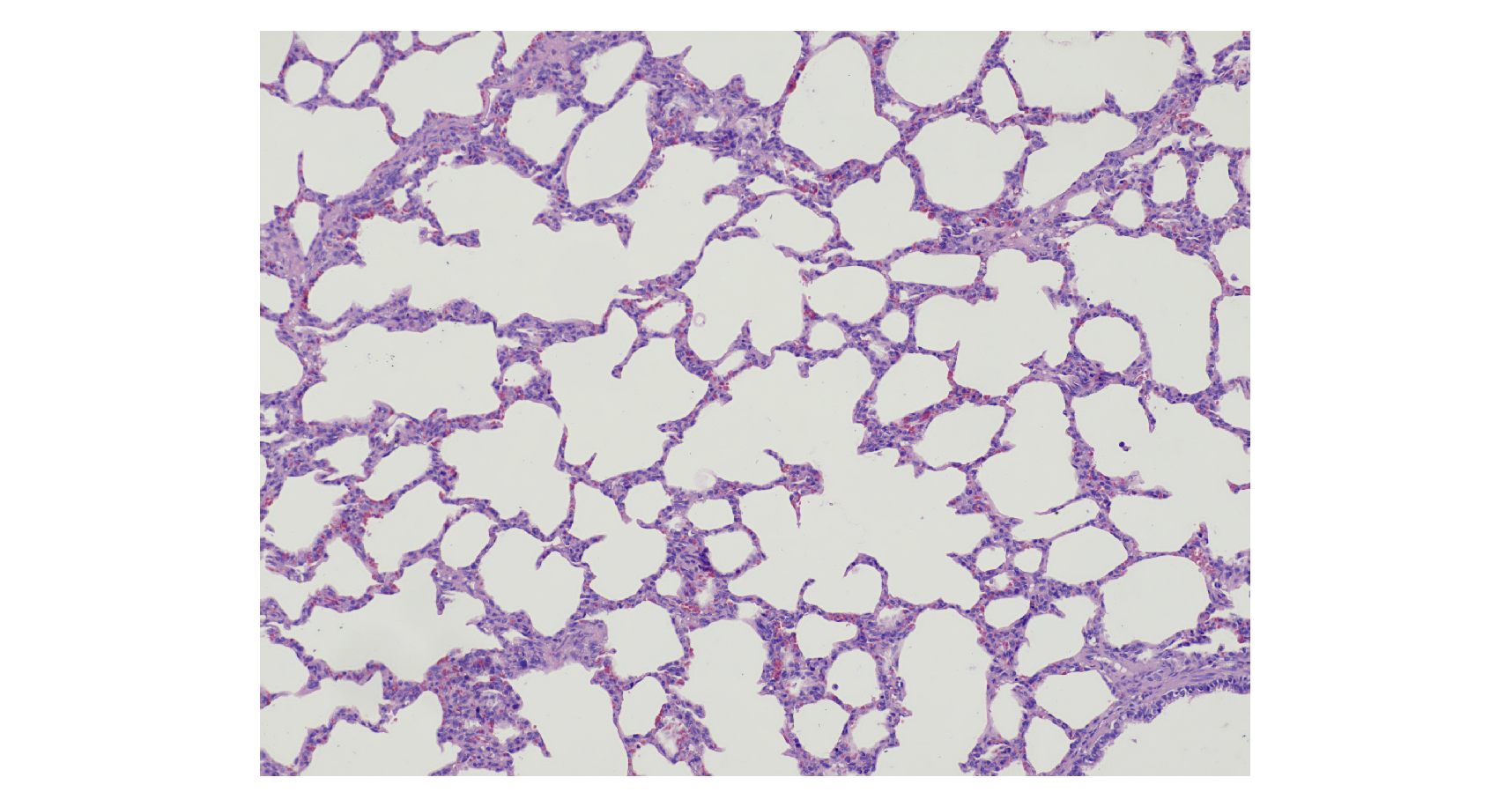

Supplement: S4 Data — (ZIP) [file ppat.1012546.s008.zip › Figure 8-10/Fig10/I/pig/PRV-UL4mut-lung/UL4-f1-10x-3.tif]

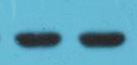

Supplement: S4 Data — (ZIP) [file ppat.1012546.s008.zip › Figure 8-10/Fig8/A/A-1/Actin.tif]

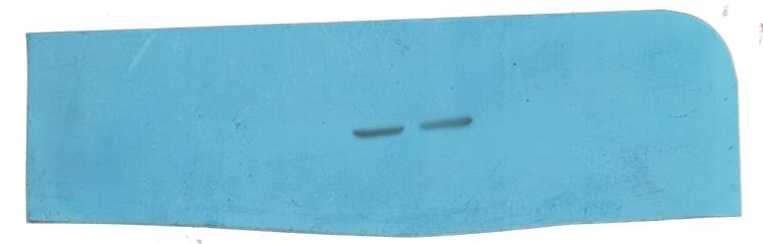

Supplement: S4 Data — (ZIP) [file ppat.1012546.s008.zip › Figure 8-10/Fig8/A/A-1/CASP1.tif]

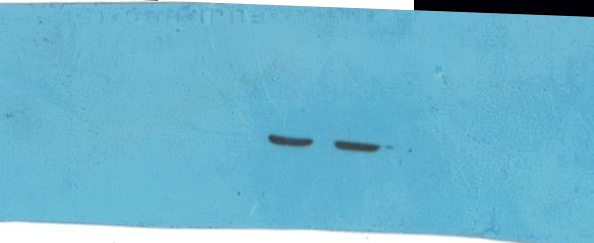

Supplement: S4 Data — (ZIP) [file ppat.1012546.s008.zip › Figure 8-10/Fig8/A/A-1/NLRP3.tif]

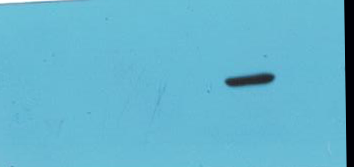

Supplement: S4 Data — (ZIP) [file ppat.1012546.s008.zip › Figure 8-10/Fig8/A/A-1/UL4.tif]

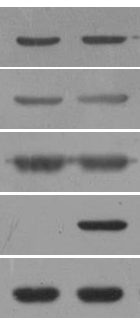

Supplement: S4 Data — (ZIP) [file ppat.1012546.s008.zip › Figure 8-10/Fig8/A/A-1/╬┤▒Ω╠Γ-1.tif]

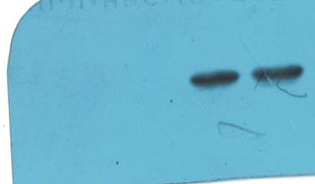

Supplement: S4 Data — (ZIP) [file ppat.1012546.s008.zip › Figure 8-10/Fig8/A/A-2/Actin.tif]

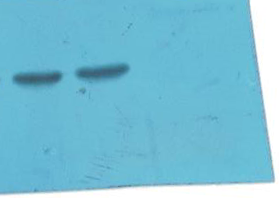

Supplement: S4 Data — (ZIP) [file ppat.1012546.s008.zip › Figure 8-10/Fig8/A/A-2/CASP1.tif]

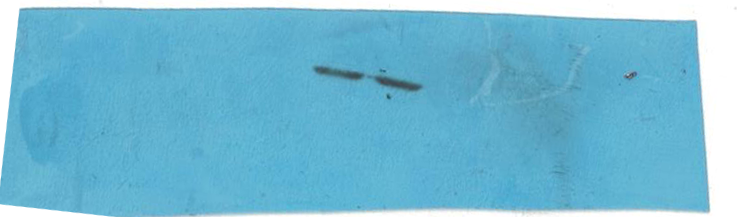

Supplement: S4 Data — (ZIP) [file ppat.1012546.s008.zip › Figure 8-10/Fig8/A/A-2/NLRP3.tif]

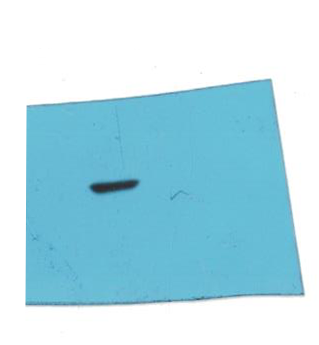

Supplement: S4 Data — (ZIP) [file ppat.1012546.s008.zip › Figure 8-10/Fig8/A/A-2/UL4.tif]

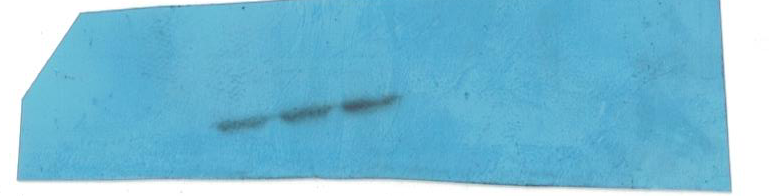

Supplement: S4 Data — (ZIP) [file ppat.1012546.s008.zip › Figure 8-10/Fig8/B/B-1/IP-ASC.tif]

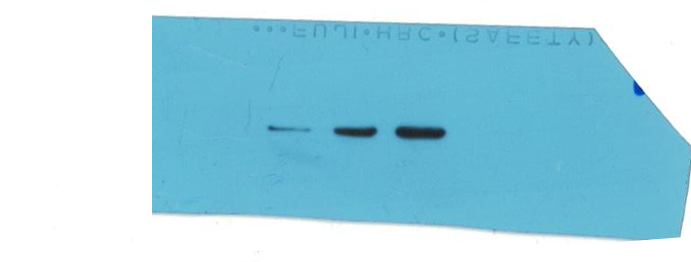

Supplement: S4 Data — (ZIP) [file ppat.1012546.s008.zip › Figure 8-10/Fig8/B/B-1/IP-CASP1.tif]

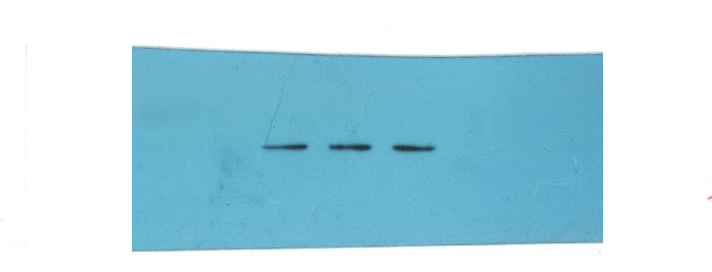

Supplement: S4 Data — (ZIP) [file ppat.1012546.s008.zip › Figure 8-10/Fig8/B/B-1/IP-NLRP3.tif]

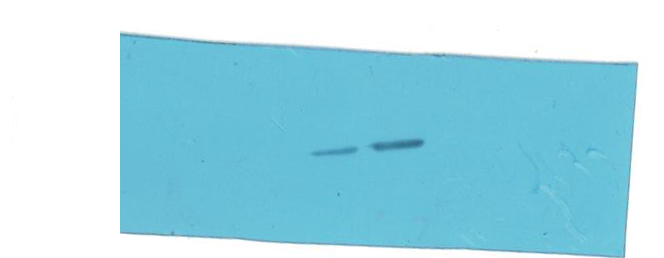

Supplement: S4 Data — (ZIP) [file ppat.1012546.s008.zip › Figure 8-10/Fig8/B/B-1/IP-UL4.tif]

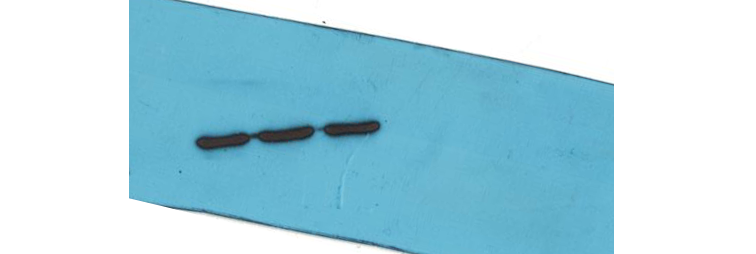

Supplement: S4 Data — (ZIP) [file ppat.1012546.s008.zip › Figure 8-10/Fig8/B/B-1/WCL-Actin.tif]

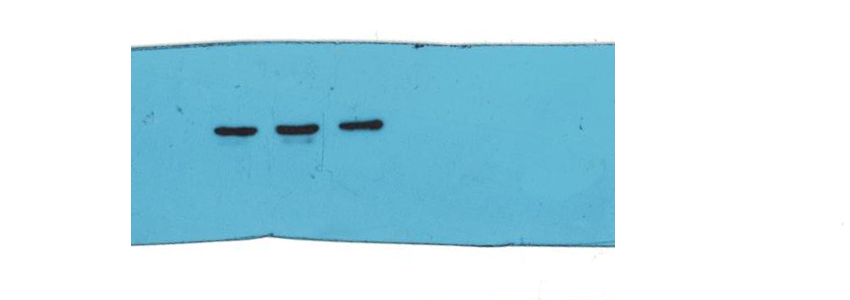

Supplement: S4 Data — (ZIP) [file ppat.1012546.s008.zip › Figure 8-10/Fig8/B/B-1/WCL-ASC.tif]

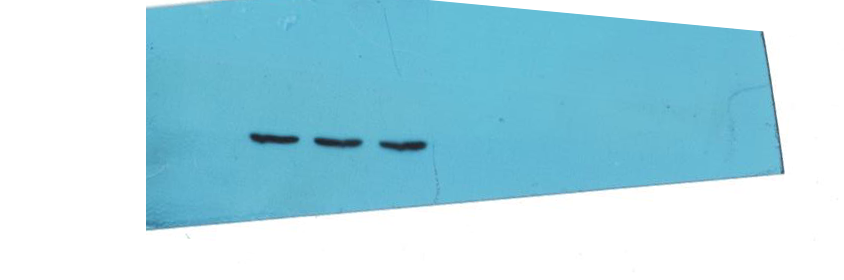

Supplement: S4 Data — (ZIP) [file ppat.1012546.s008.zip › Figure 8-10/Fig8/B/B-1/WCL-CASP1.tif]

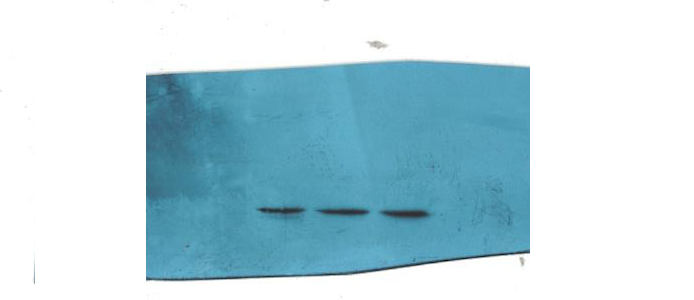

Supplement: S4 Data — (ZIP) [file ppat.1012546.s008.zip › Figure 8-10/Fig8/B/B-1/WCL-NLRP3.tif]

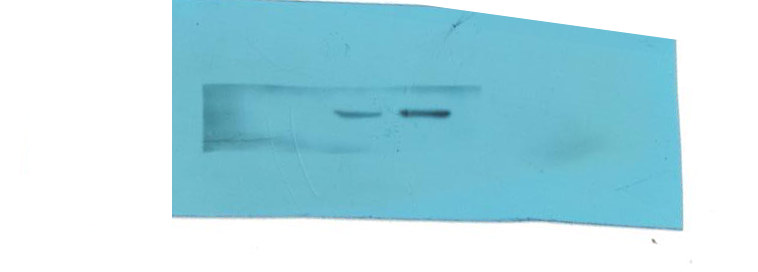

Supplement: S4 Data — (ZIP) [file ppat.1012546.s008.zip › Figure 8-10/Fig8/B/B-1/WCL-UL4.tif]

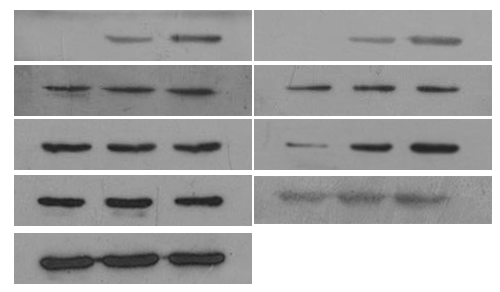

Supplement: S4 Data — (ZIP) [file ppat.1012546.s008.zip › Figure 8-10/Fig8/B/B-1/╬┤▒Ω╠Γ-3.tif]

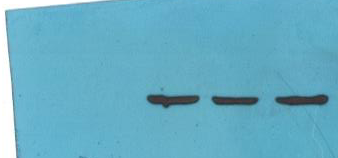

Supplement: S4 Data — (ZIP) [file ppat.1012546.s008.zip › Figure 8-10/Fig8/B/B-2/IP-ASC.tif]

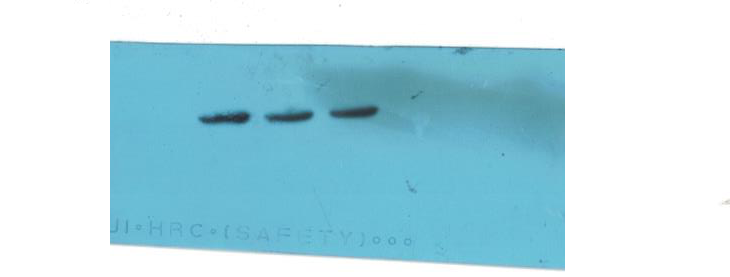

Supplement: S4 Data — (ZIP) [file ppat.1012546.s008.zip › Figure 8-10/Fig8/B/B-2/IP-NLRP3.tif]

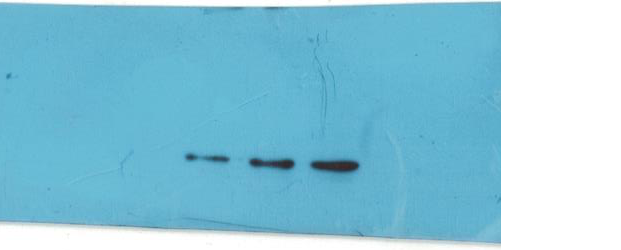

Supplement: S4 Data — (ZIP) [file ppat.1012546.s008.zip › Figure 8-10/Fig8/B/B-2/IP-pro-CASP1.tif]

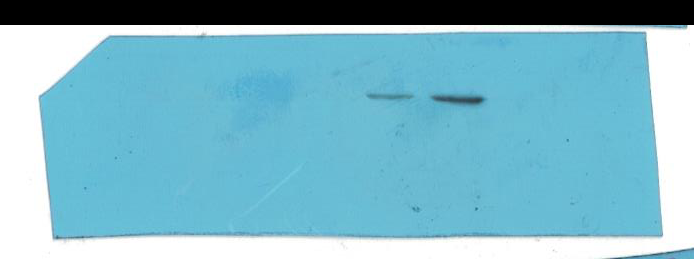

Supplement: S4 Data — (ZIP) [file ppat.1012546.s008.zip › Figure 8-10/Fig8/B/B-2/IP-UL4.tif]

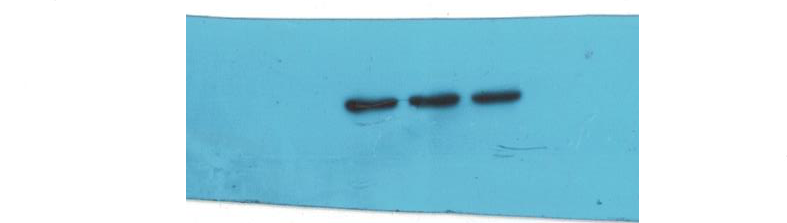

Supplement: S4 Data — (ZIP) [file ppat.1012546.s008.zip › Figure 8-10/Fig8/B/B-2/WCL-Actin.tif]

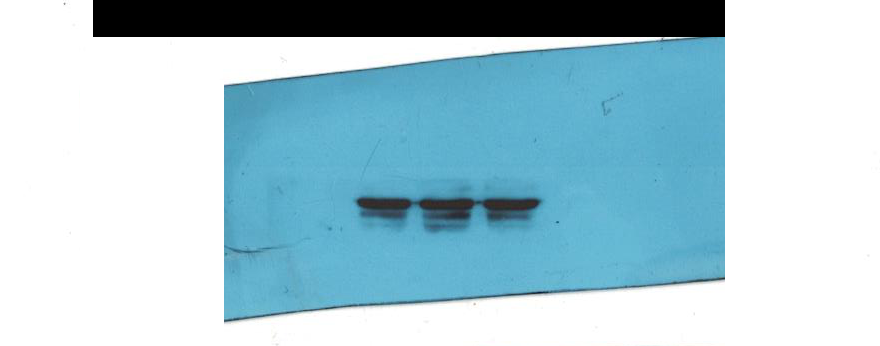

Supplement: S4 Data — (ZIP) [file ppat.1012546.s008.zip › Figure 8-10/Fig8/B/B-2/WCL-ASC.tif]

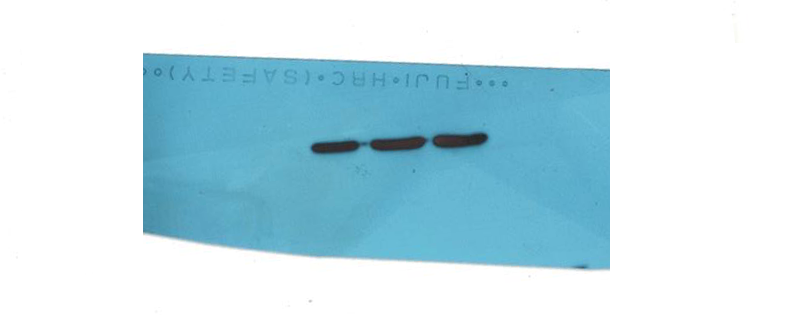

Supplement: S4 Data — (ZIP) [file ppat.1012546.s008.zip › Figure 8-10/Fig8/B/B-2/WCL-NLRP3.tif]

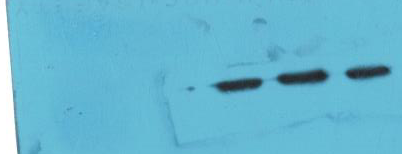

Supplement: S4 Data — (ZIP) [file ppat.1012546.s008.zip › Figure 8-10/Fig8/B/B-2/WCL-Pro-CASP1.tif]

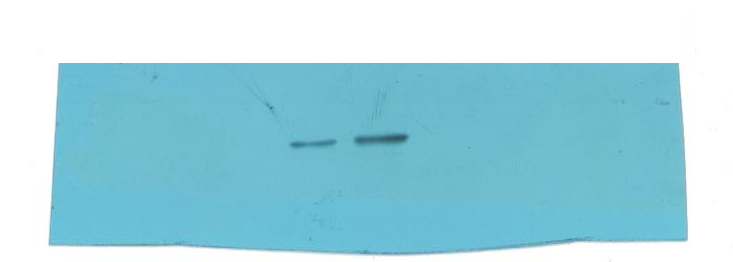

Supplement: S4 Data — (ZIP) [file ppat.1012546.s008.zip › Figure 8-10/Fig8/B/B-2/WCL-UL4.tif]

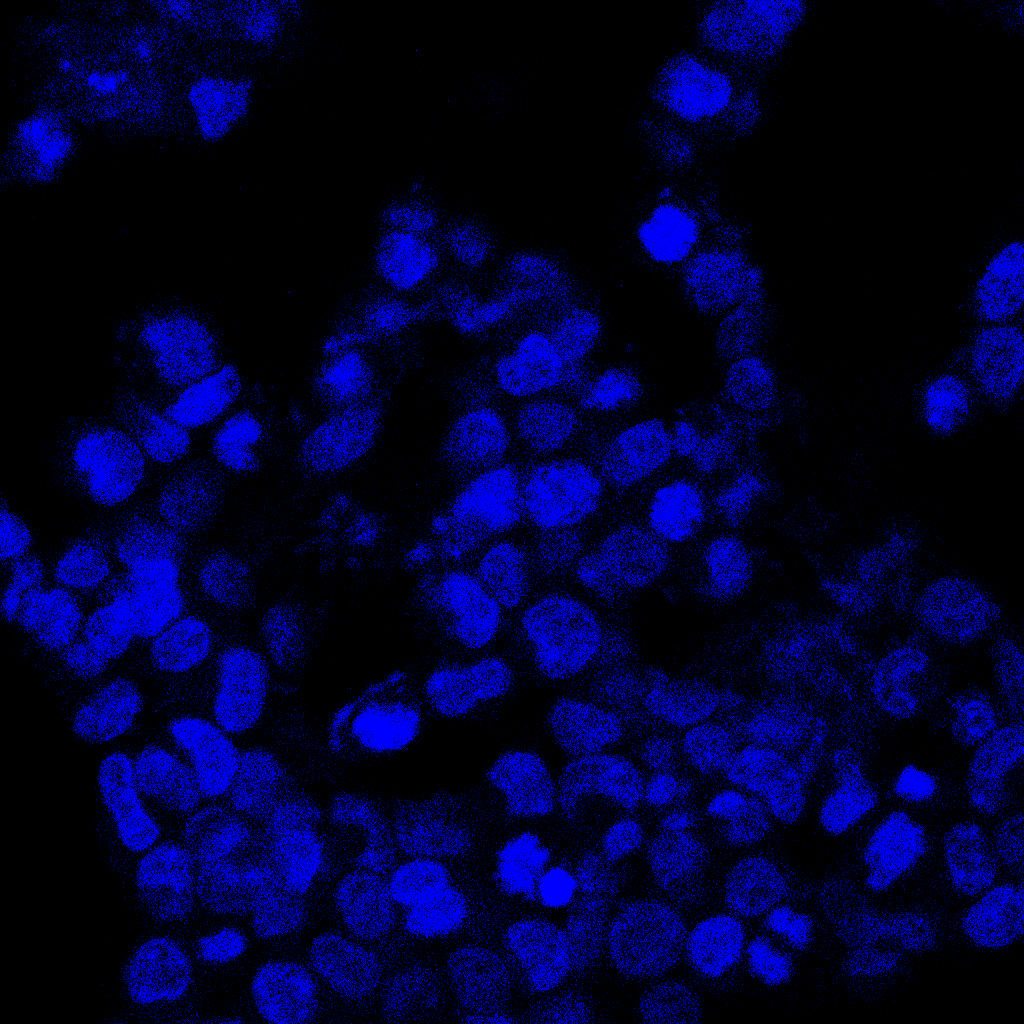

Supplement: S4 Data — (ZIP) [file ppat.1012546.s008.zip › Figure 8-10/Fig8/C/1/HA-UL4+Flag-ASC/DAPI.tif]

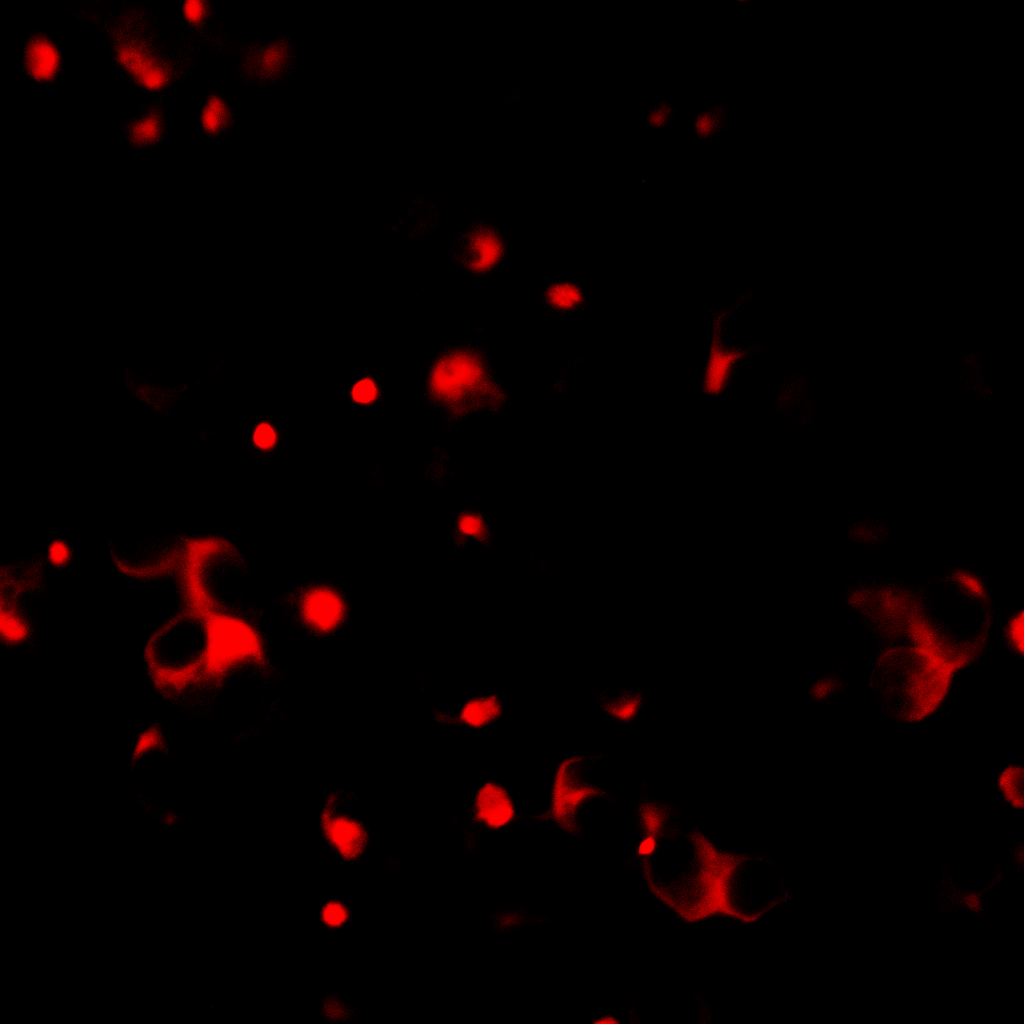

Supplement: S4 Data — (ZIP) [file ppat.1012546.s008.zip › Figure 8-10/Fig8/C/1/HA-UL4+Flag-ASC/Flag-ASC.tif]

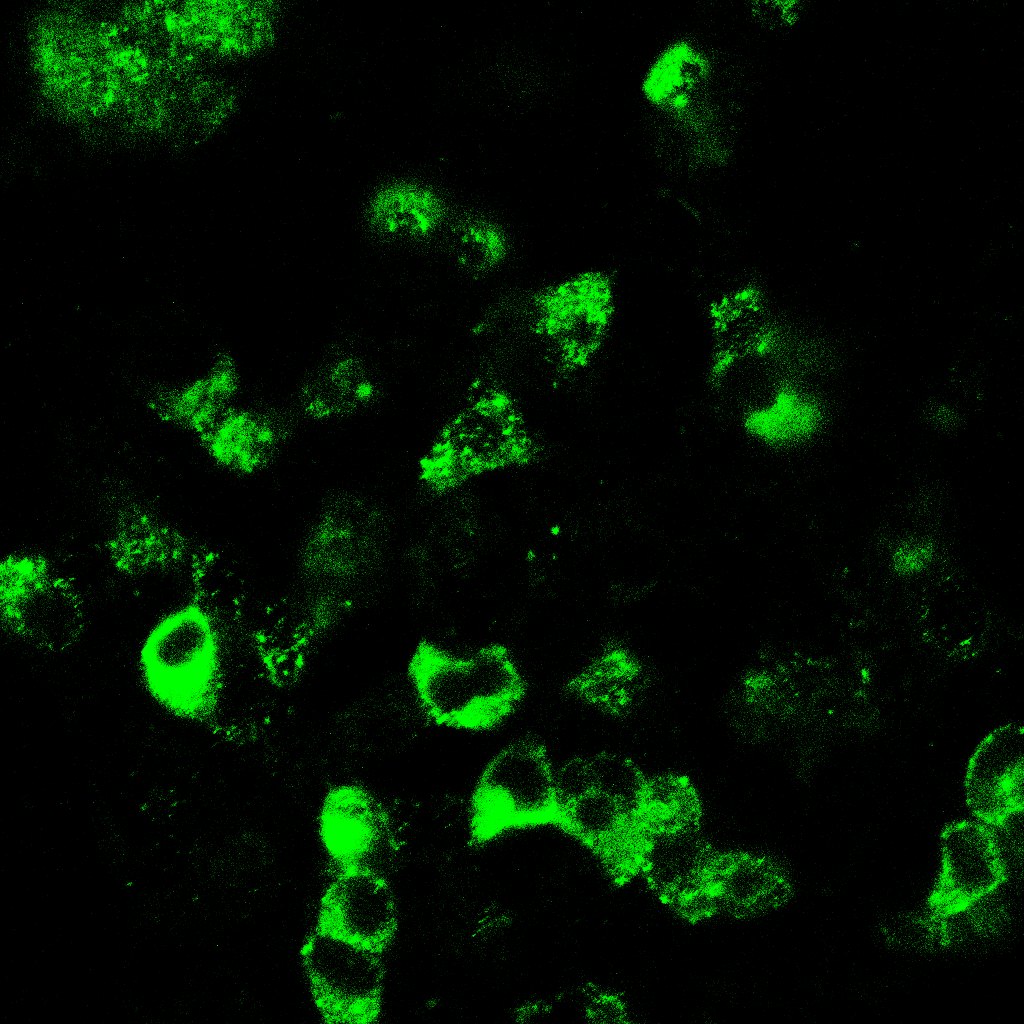

Supplement: S4 Data — (ZIP) [file ppat.1012546.s008.zip › Figure 8-10/Fig8/C/1/HA-UL4+Flag-ASC/HA-UL4.tif]

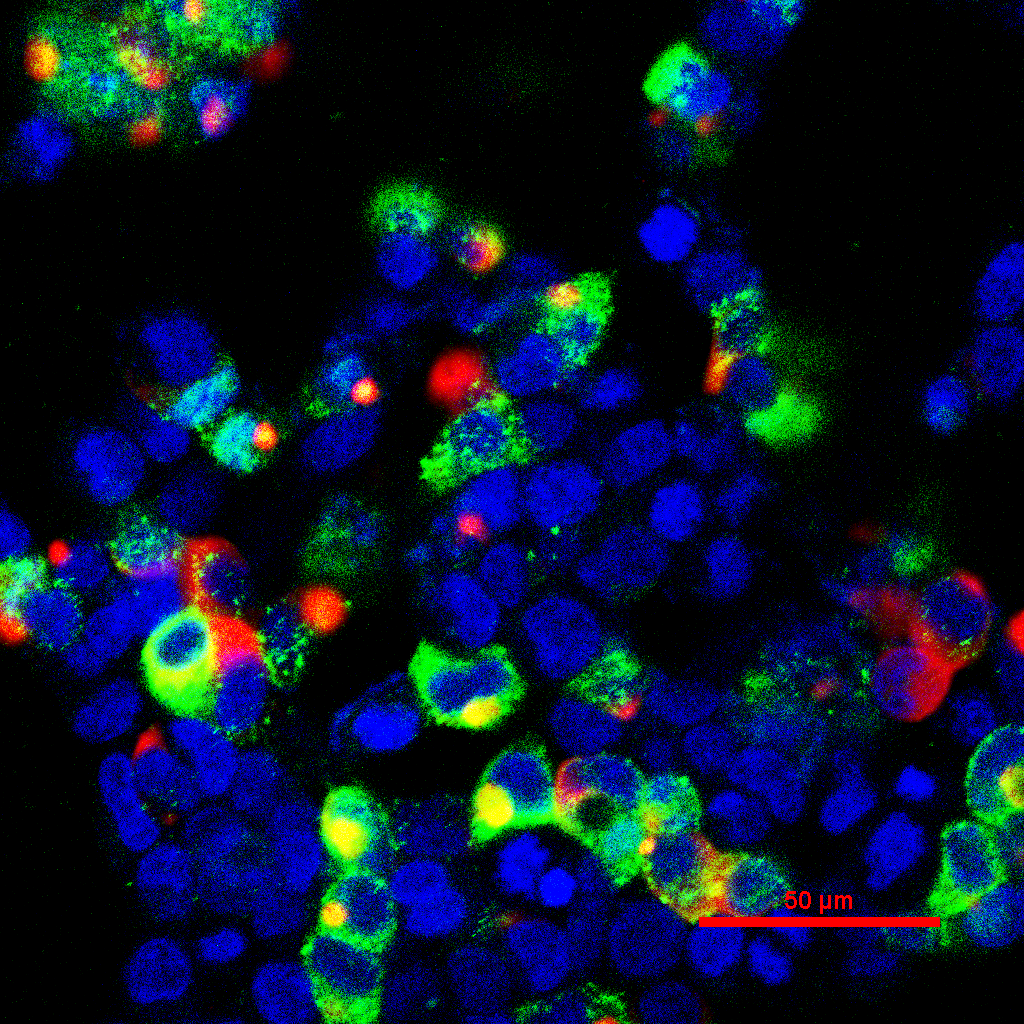

Supplement: S4 Data — (ZIP) [file ppat.1012546.s008.zip › Figure 8-10/Fig8/C/1/HA-UL4+Flag-ASC/Merge.tif]

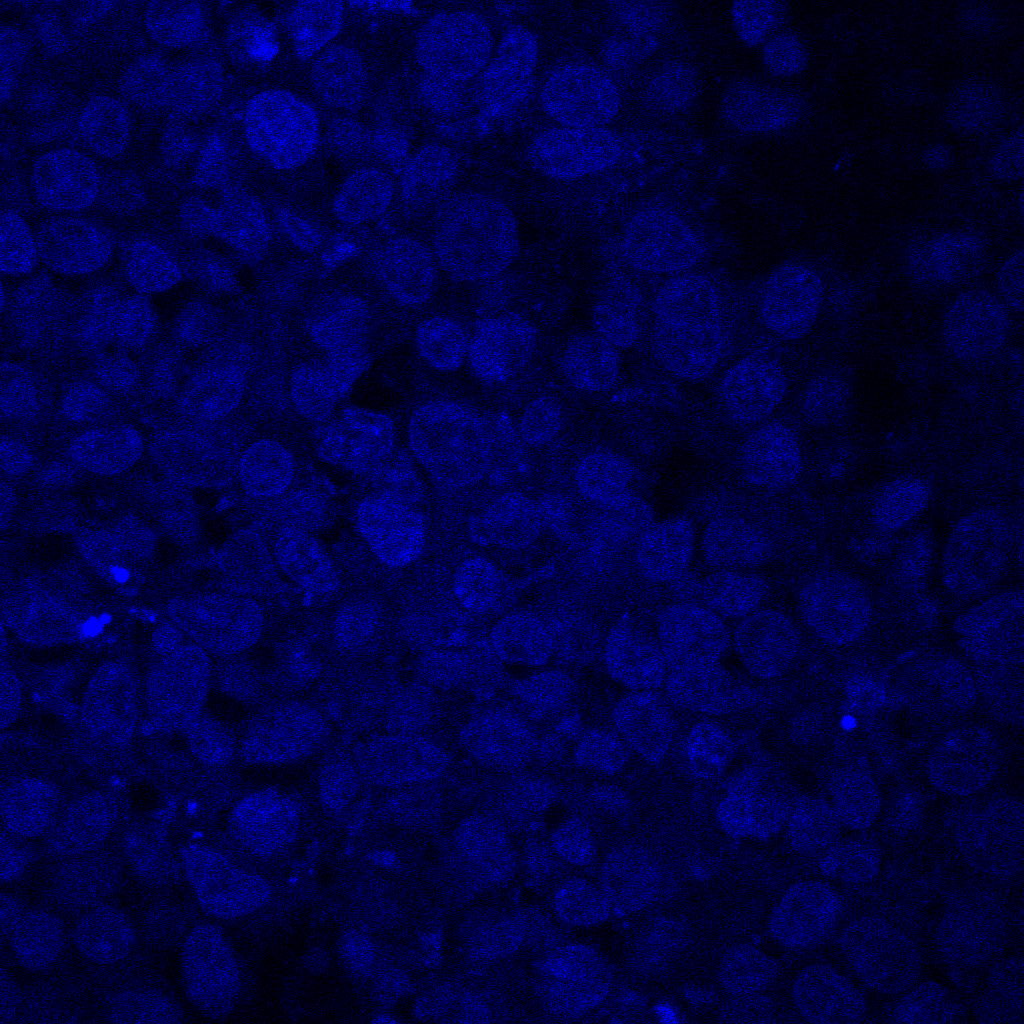

Supplement: S4 Data — (ZIP) [file ppat.1012546.s008.zip › Figure 8-10/Fig8/C/1/Vec+Flag-ASC/DAPI.tif]

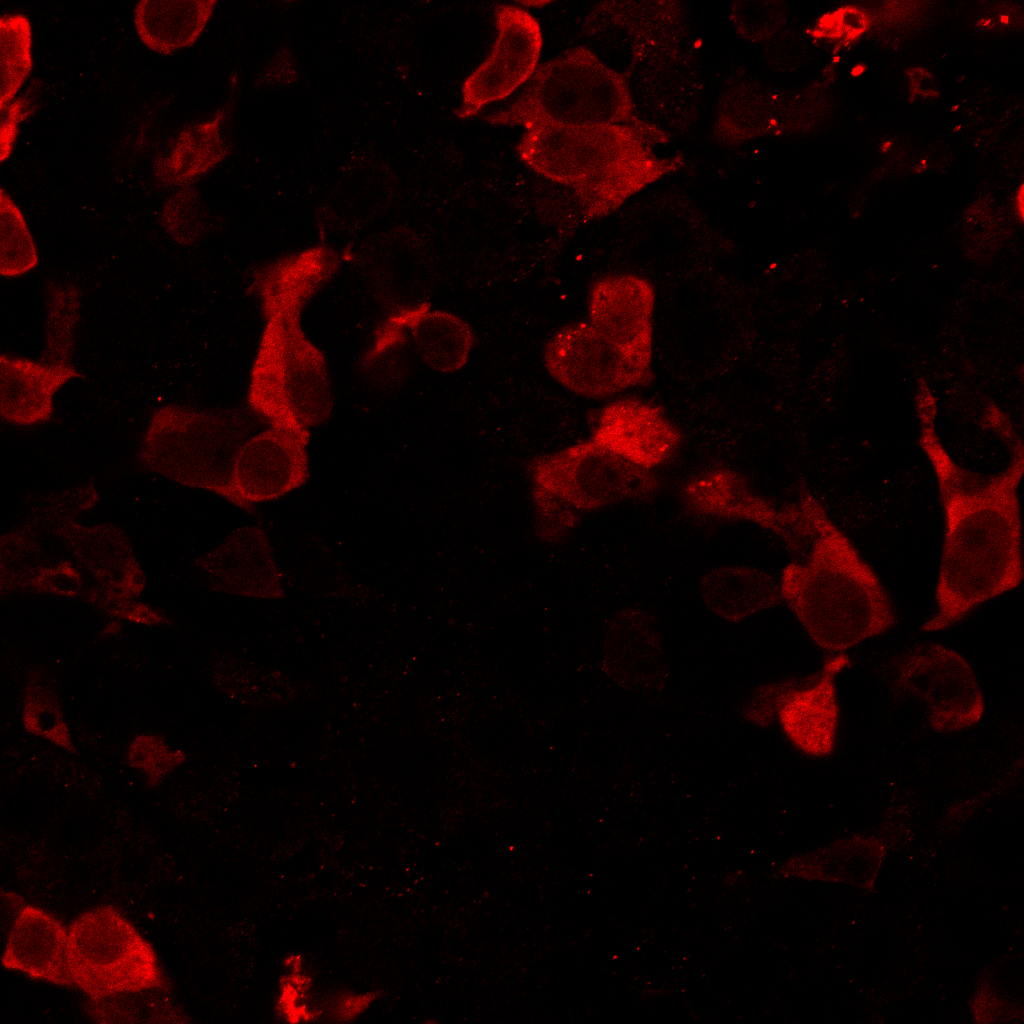

Supplement: S4 Data — (ZIP) [file ppat.1012546.s008.zip › Figure 8-10/Fig8/C/1/Vec+Flag-ASC/Flag-ASC.tif]

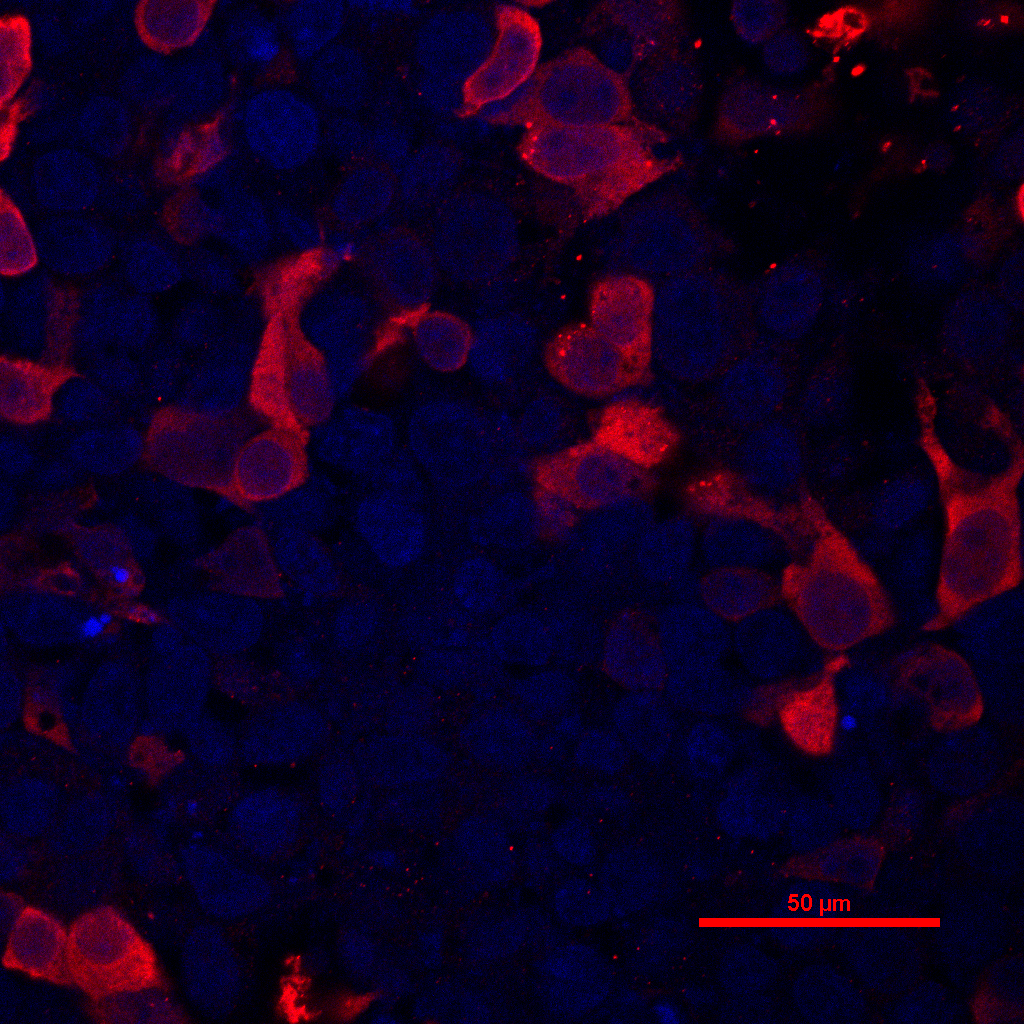

Supplement: S4 Data — (ZIP) [file ppat.1012546.s008.zip › Figure 8-10/Fig8/C/1/Vec+Flag-ASC/Merge.tif]

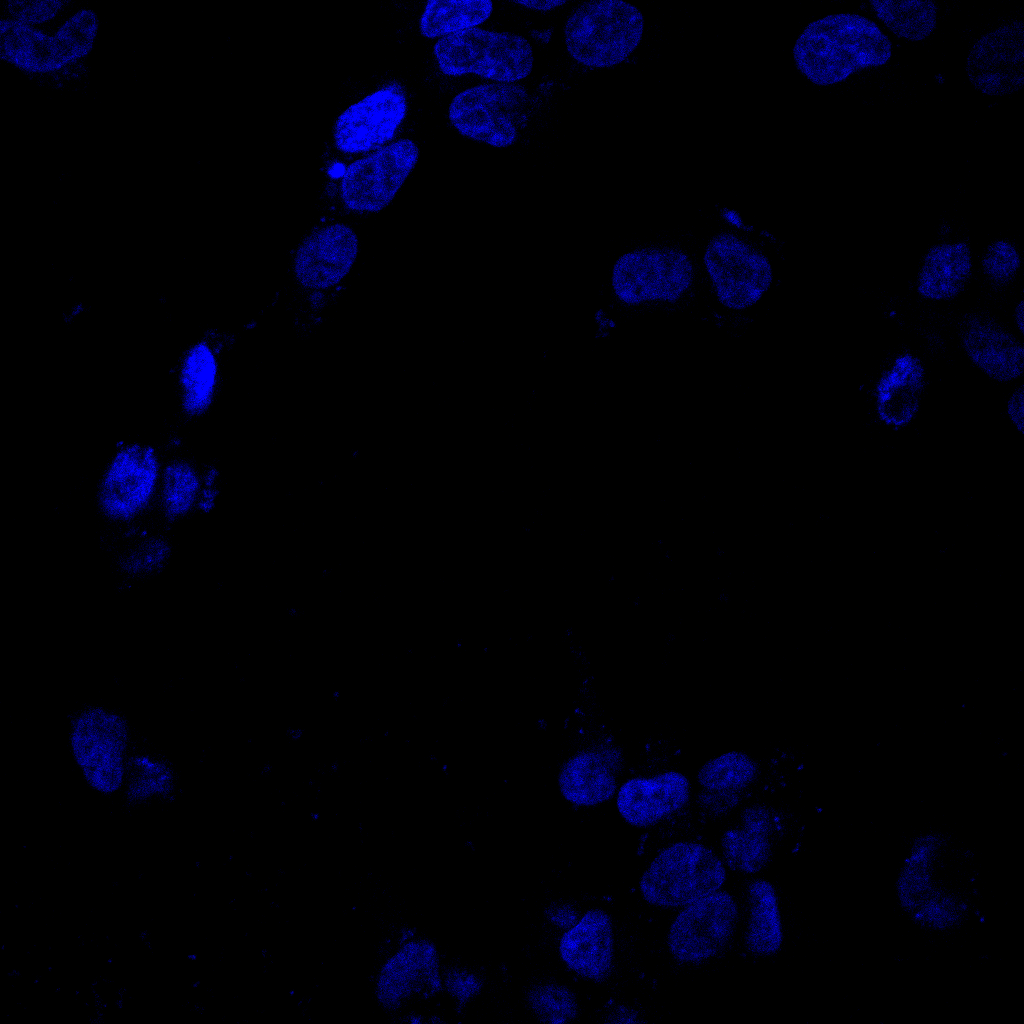

Supplement: S4 Data — (ZIP) [file ppat.1012546.s008.zip › Figure 8-10/Fig8/C/2/HA-UL4+Flag-ASC/DAPI.tif]

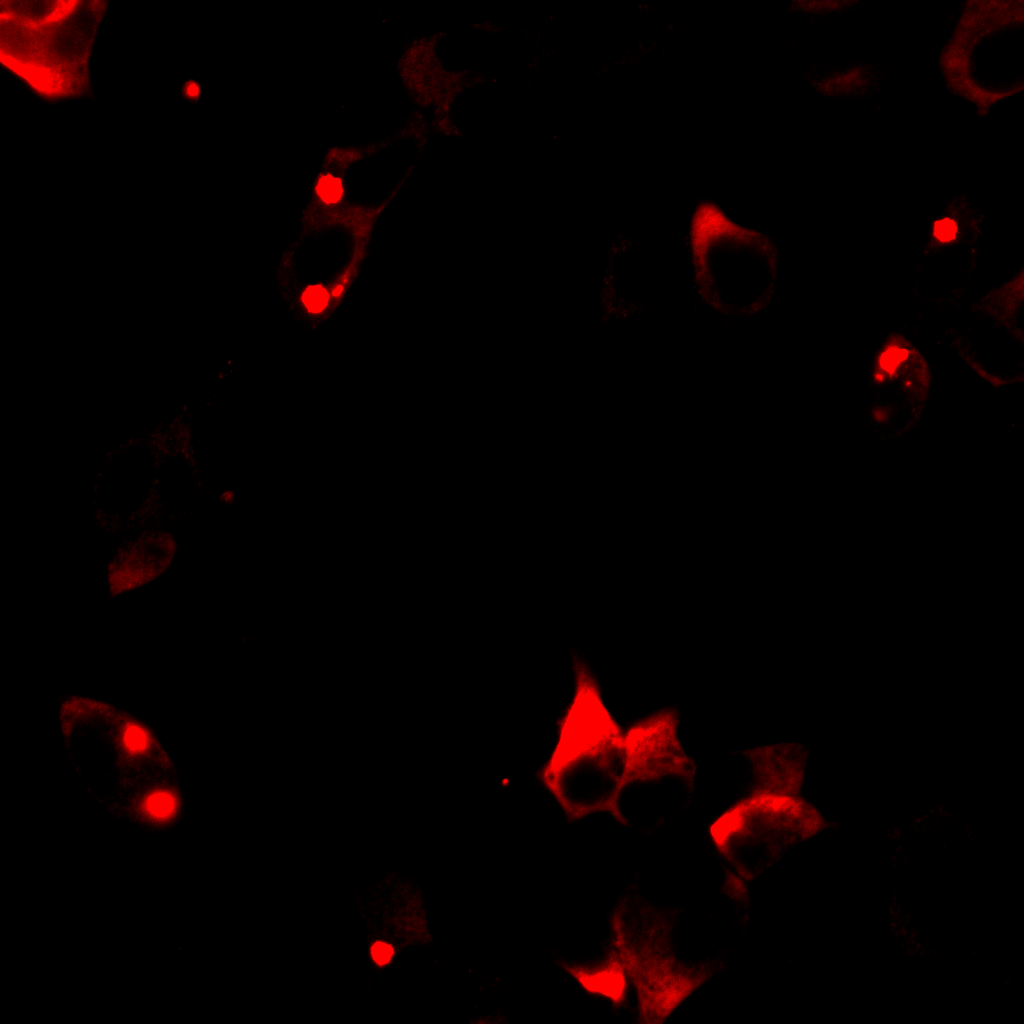

Supplement: S4 Data — (ZIP) [file ppat.1012546.s008.zip › Figure 8-10/Fig8/C/2/HA-UL4+Flag-ASC/Flag-ASC.tif]

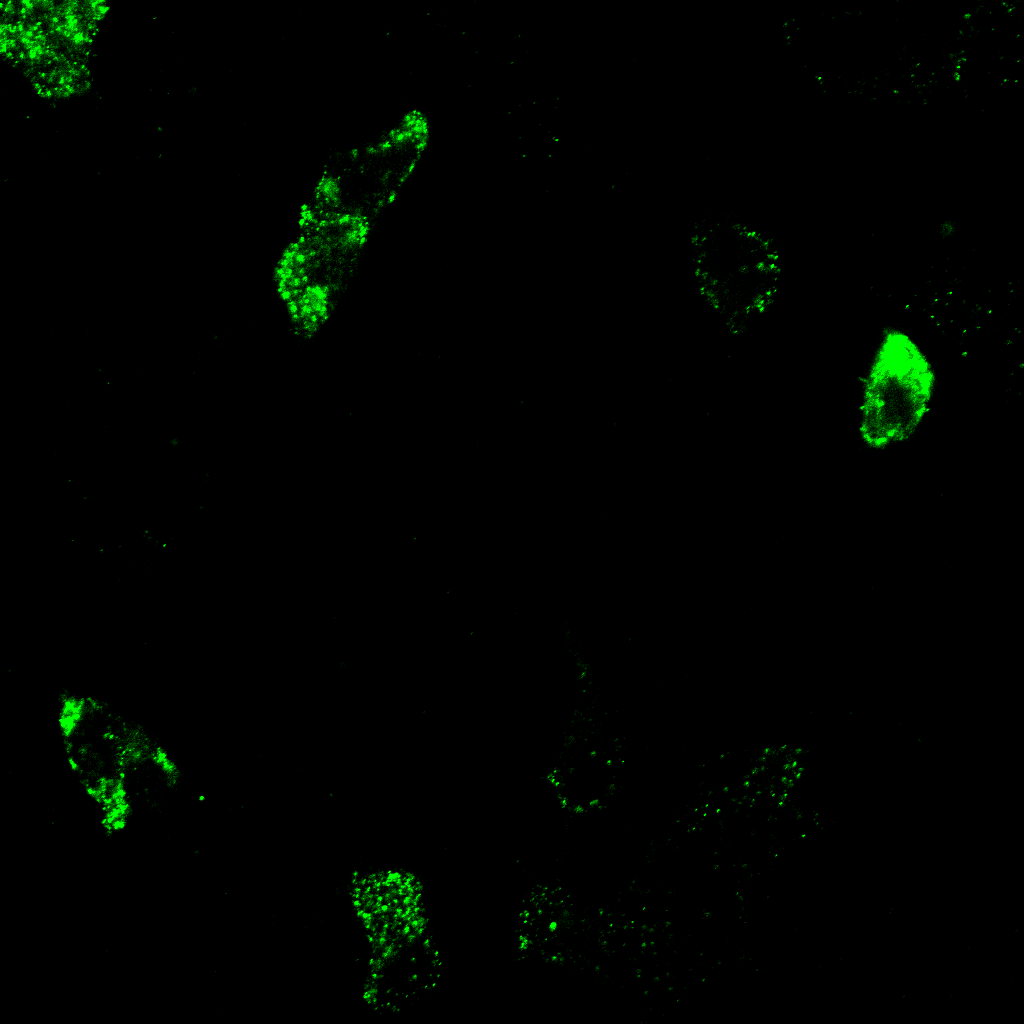

Supplement: S4 Data — (ZIP) [file ppat.1012546.s008.zip › Figure 8-10/Fig8/C/2/HA-UL4+Flag-ASC/HA-UL4.tif]

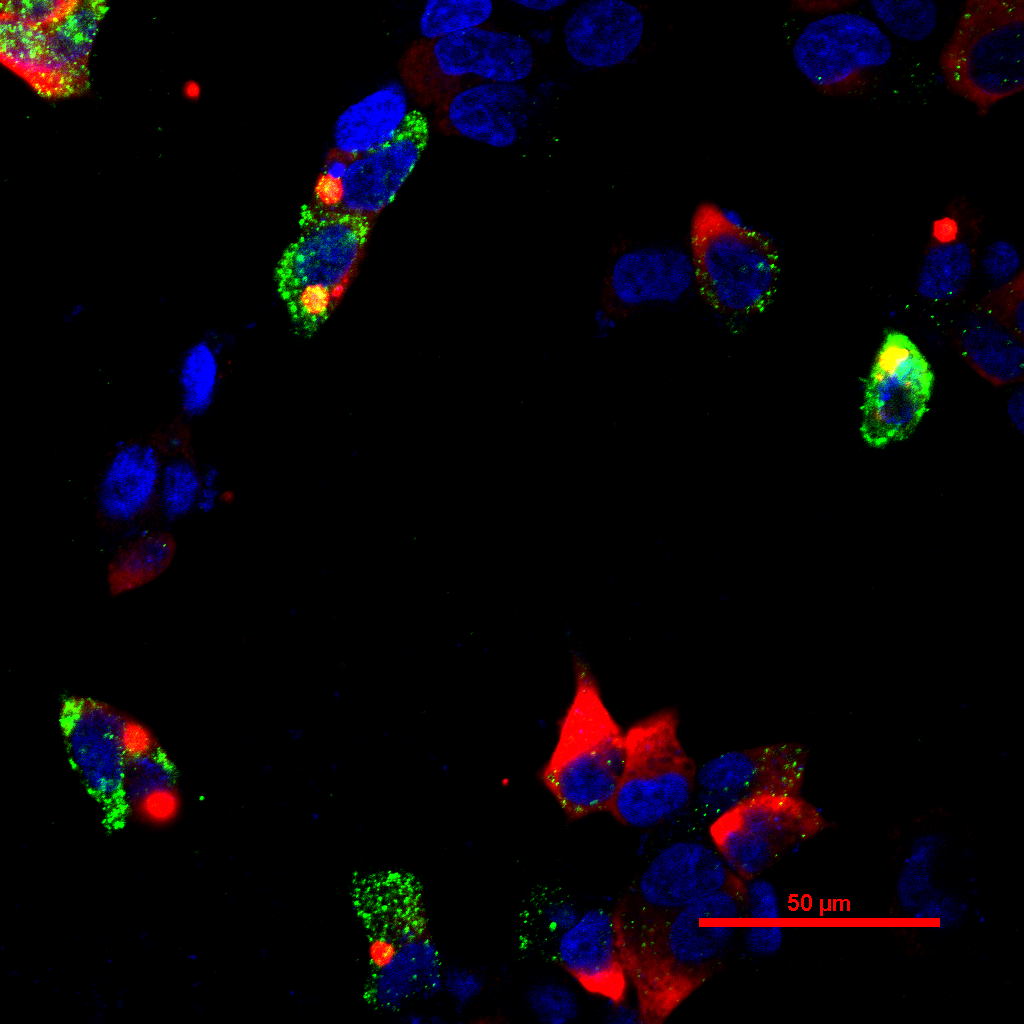

Supplement: S4 Data — (ZIP) [file ppat.1012546.s008.zip › Figure 8-10/Fig8/C/2/HA-UL4+Flag-ASC/Merge.tif]

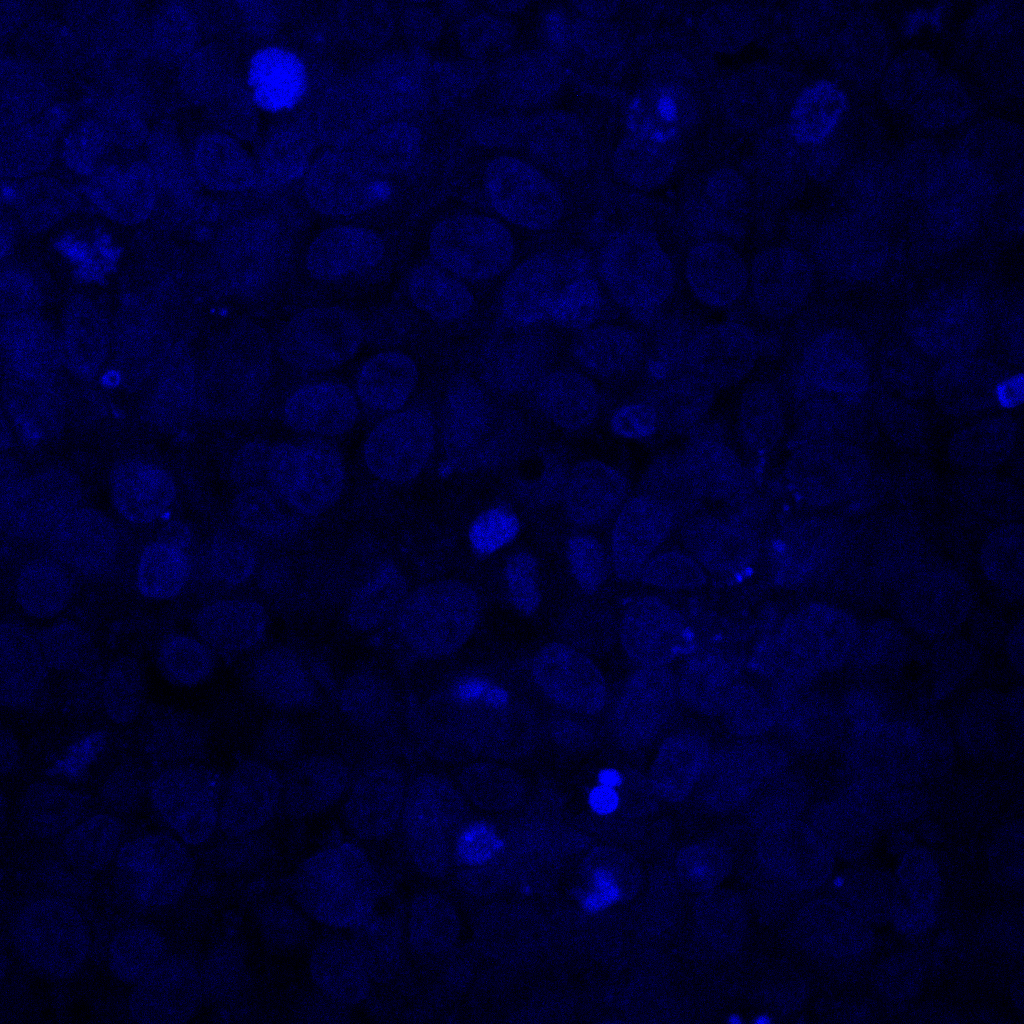

Supplement: S4 Data — (ZIP) [file ppat.1012546.s008.zip › Figure 8-10/Fig8/C/2/Vec+Flag-ASC/DAPI.tif]

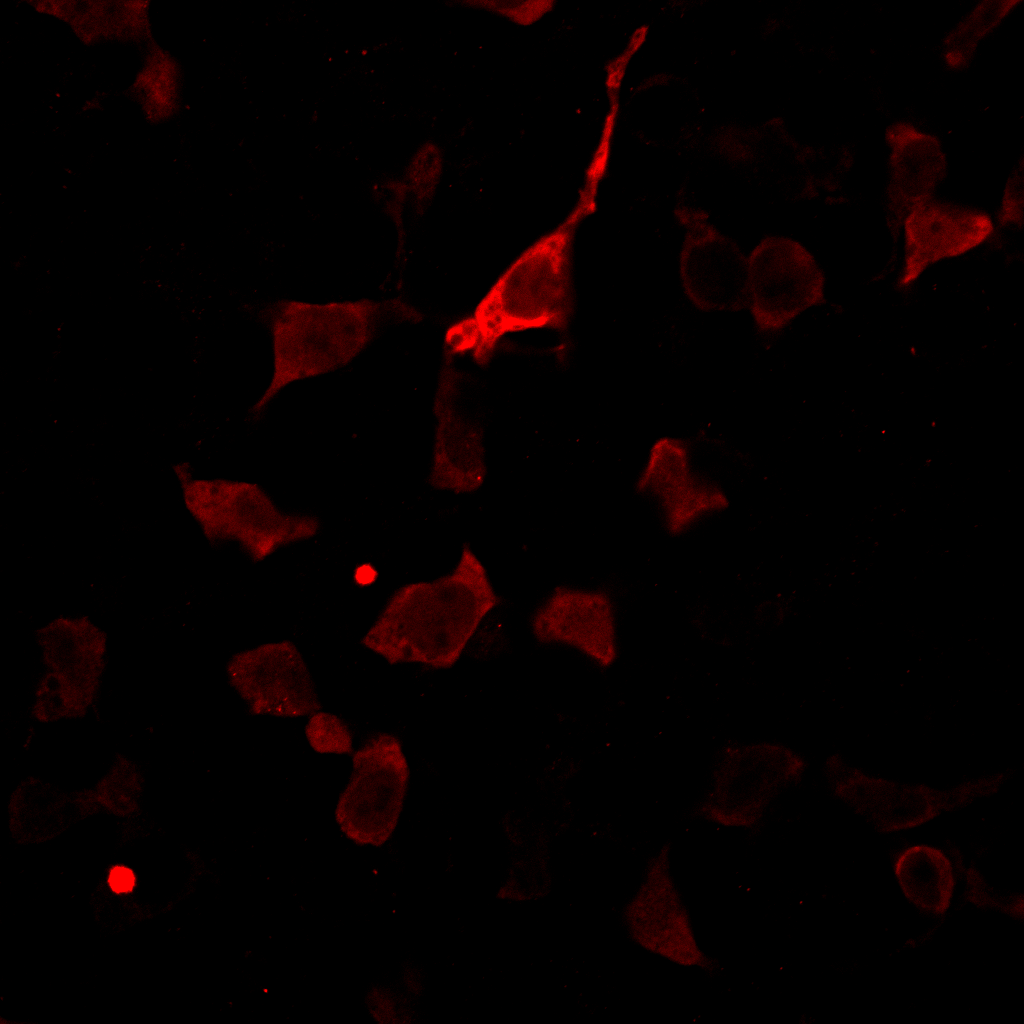

Supplement: S4 Data — (ZIP) [file ppat.1012546.s008.zip › Figure 8-10/Fig8/C/2/Vec+Flag-ASC/Flag-ASC.tif]

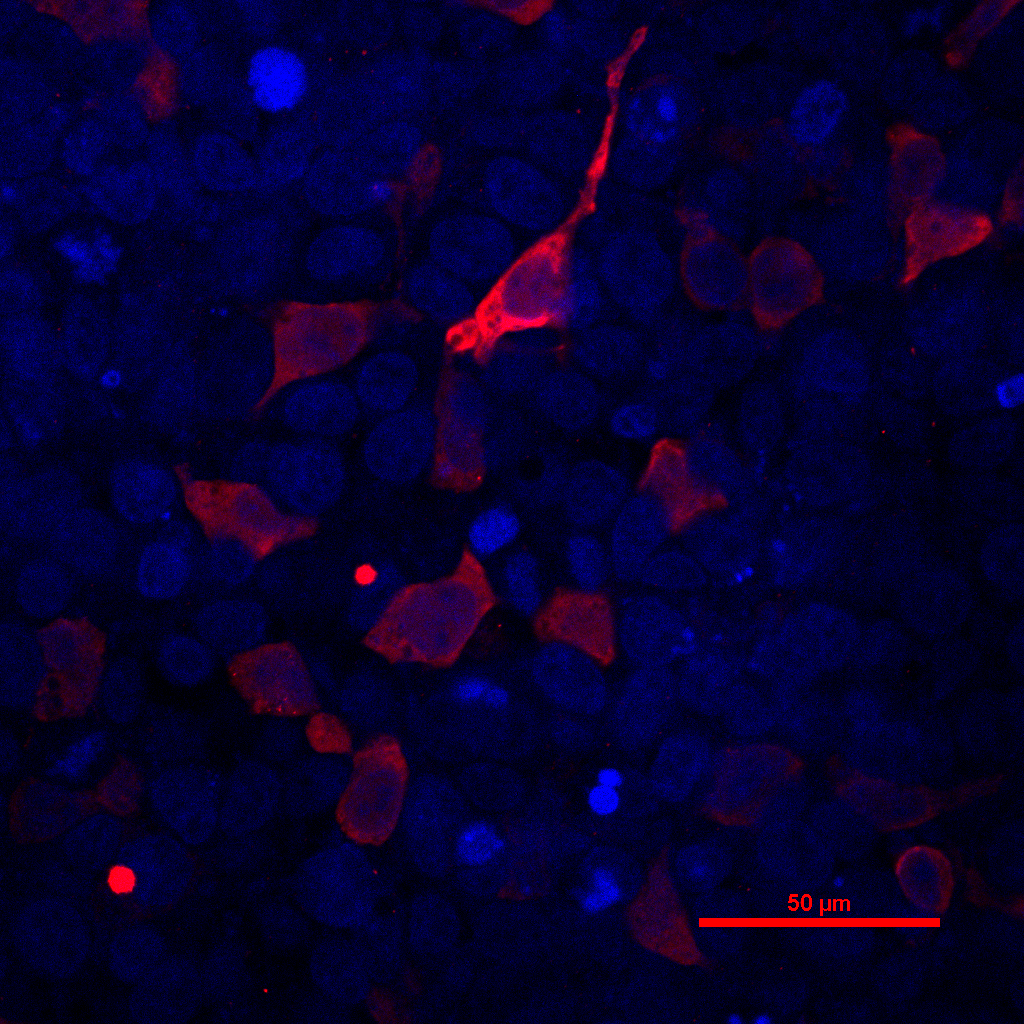

Supplement: S4 Data — (ZIP) [file ppat.1012546.s008.zip › Figure 8-10/Fig8/C/2/Vec+Flag-ASC/RGB.tif]

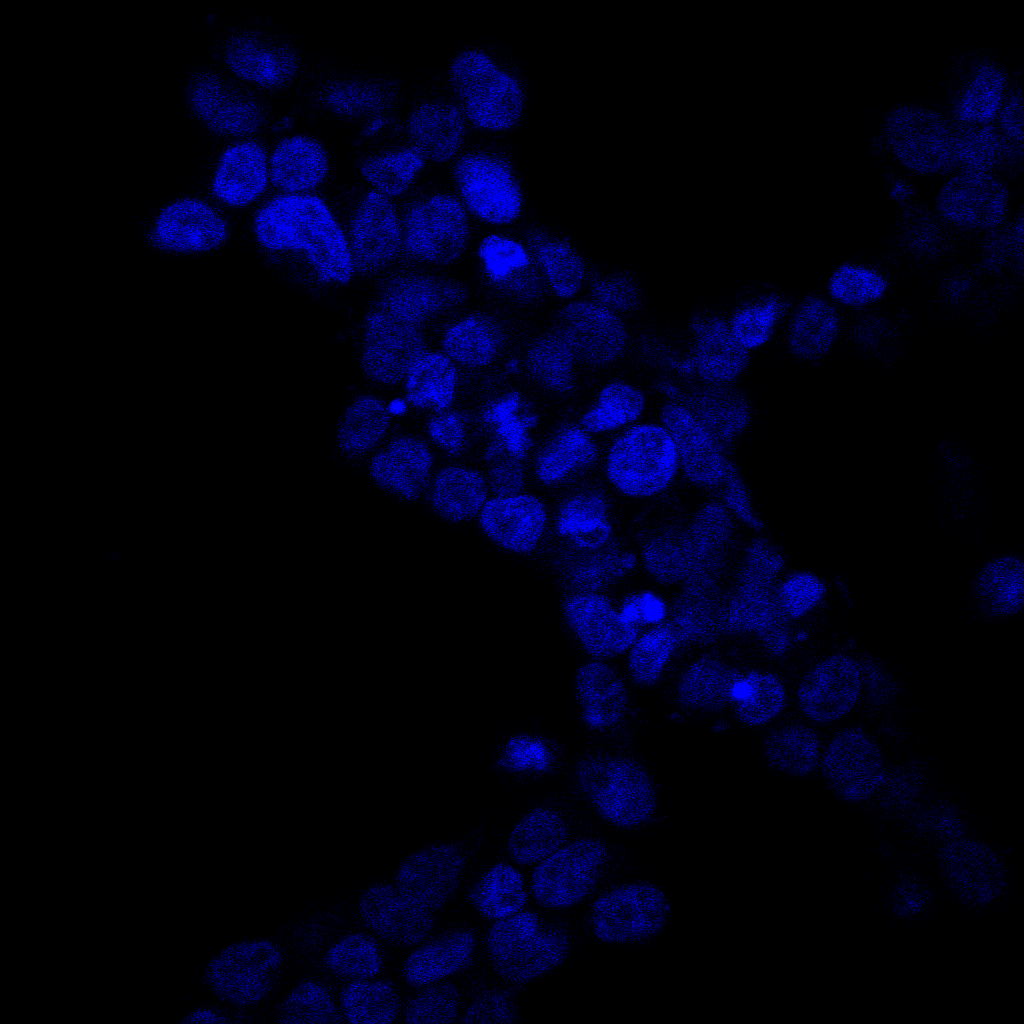

Supplement: S4 Data — (ZIP) [file ppat.1012546.s008.zip › Figure 8-10/Fig8/C/3/HA-UL4+Flag-ASC/DAPI.tif]

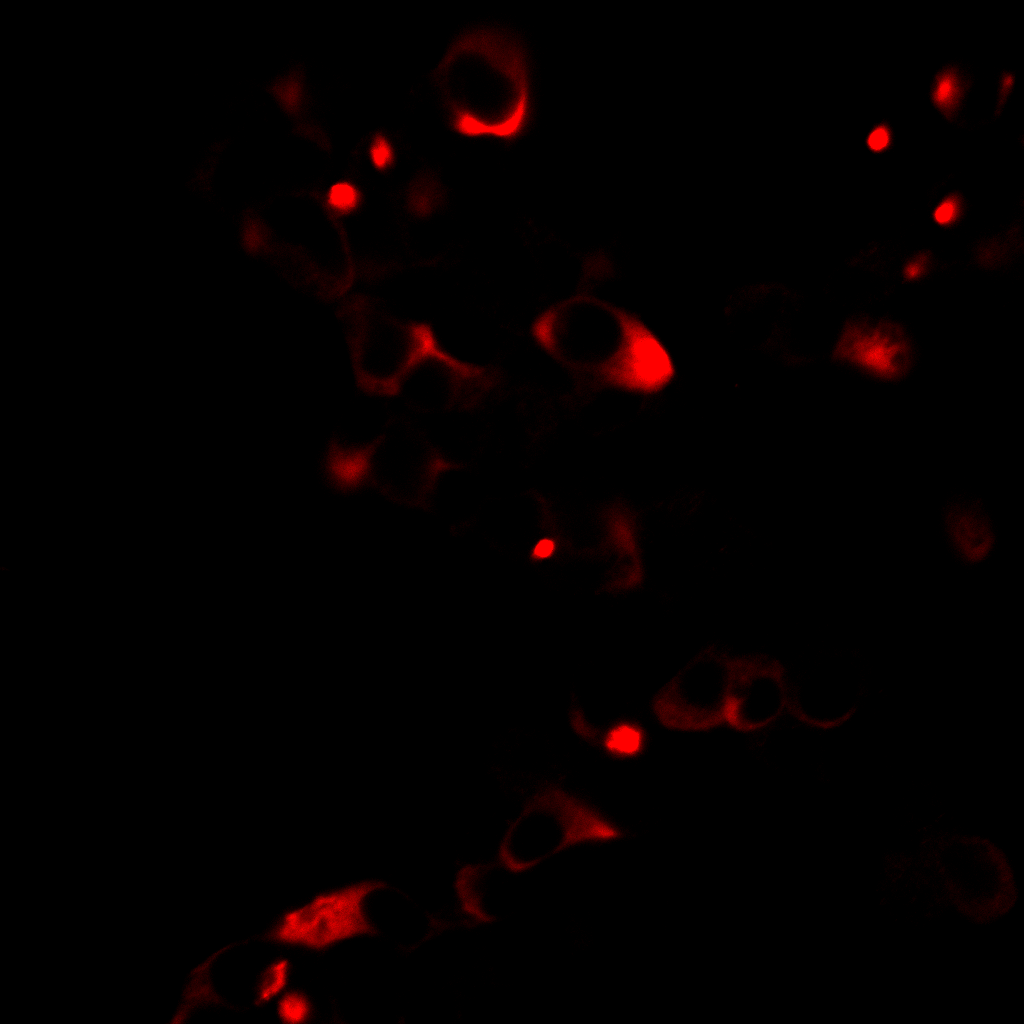

Supplement: S4 Data — (ZIP) [file ppat.1012546.s008.zip › Figure 8-10/Fig8/C/3/HA-UL4+Flag-ASC/Flag-ASC.tif]

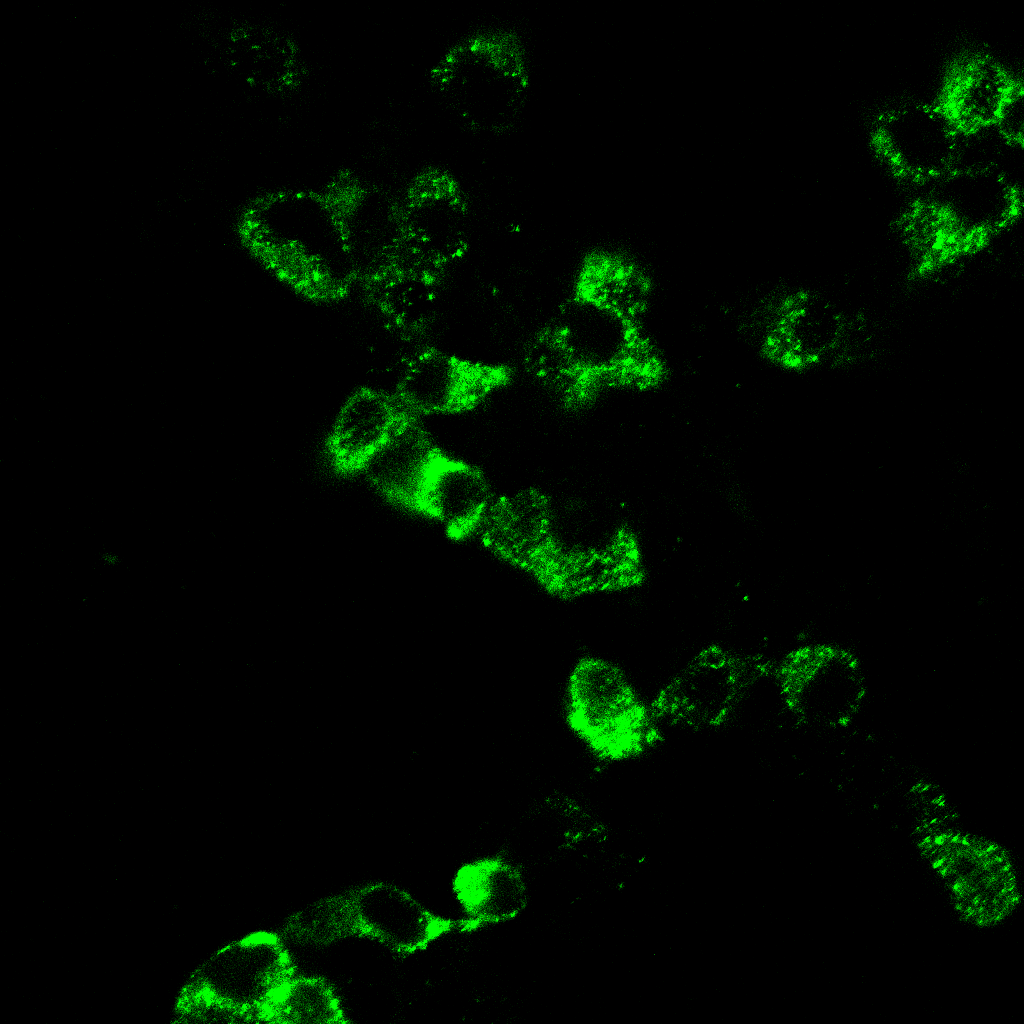

Supplement: S4 Data — (ZIP) [file ppat.1012546.s008.zip › Figure 8-10/Fig8/C/3/HA-UL4+Flag-ASC/HA-UL4.tif]

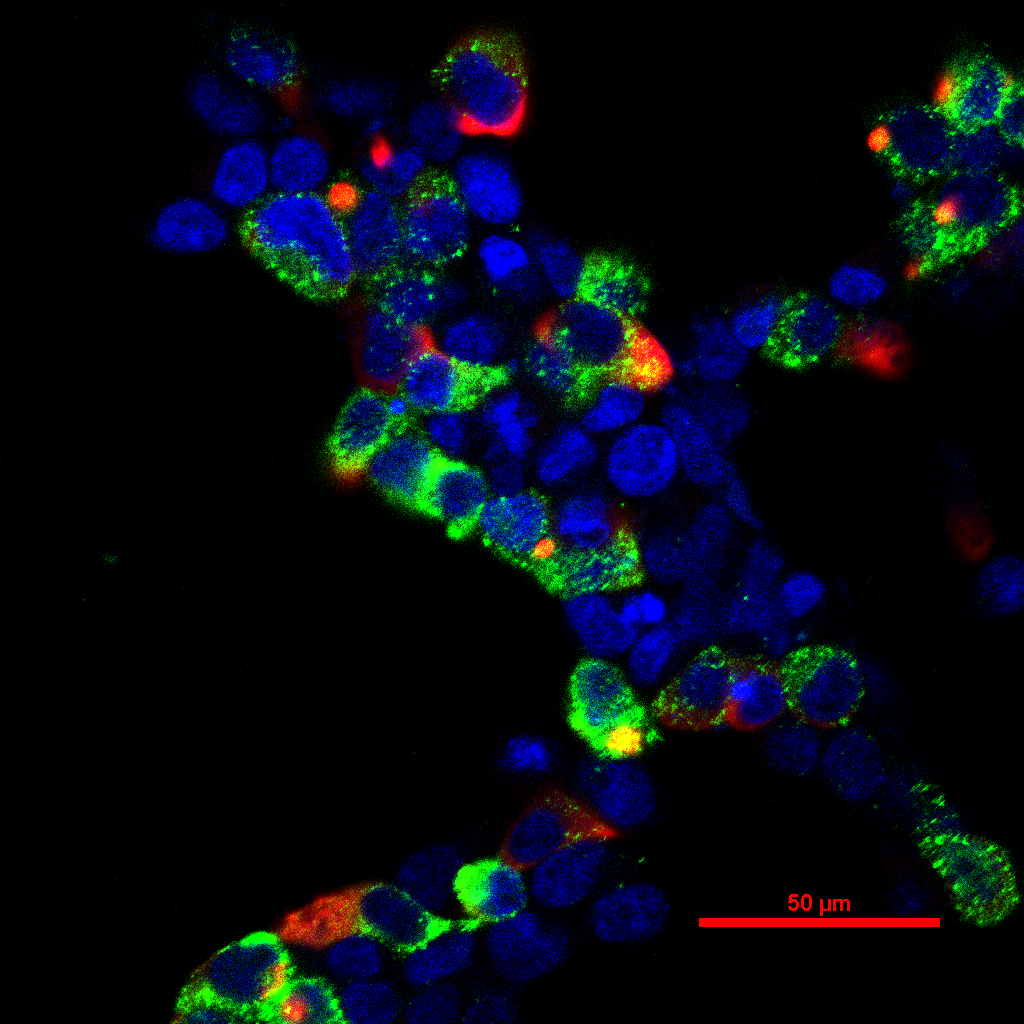

Supplement: S4 Data — (ZIP) [file ppat.1012546.s008.zip › Figure 8-10/Fig8/C/3/HA-UL4+Flag-ASC/Merge.tif]

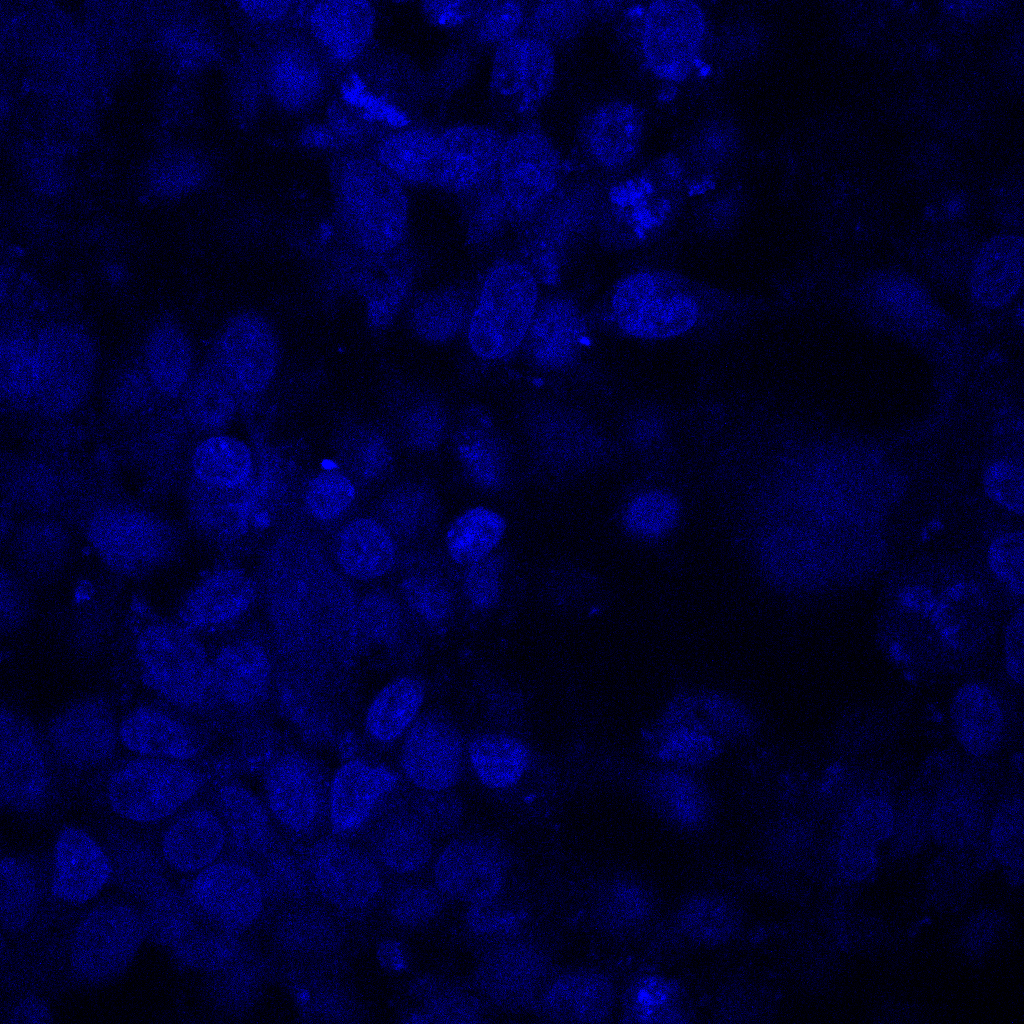

Supplement: S4 Data — (ZIP) [file ppat.1012546.s008.zip › Figure 8-10/Fig8/C/3/Vec+Flag-ASC/DAPI.tif]
