# Supplementary figures and images for: Pseudorabies Virus UL4 protein promotes the ASC-dependent inflammasome activation and pyroptosis to exacerbate inflammation (part 5 of 6)
Source: PLoS Pathog. 2024 Sep 24;20(9):e1012546. doi: 10.1371/journal.ppat.1012546 (PMC11421794; doi:10.1371/journal.ppat.1012546)

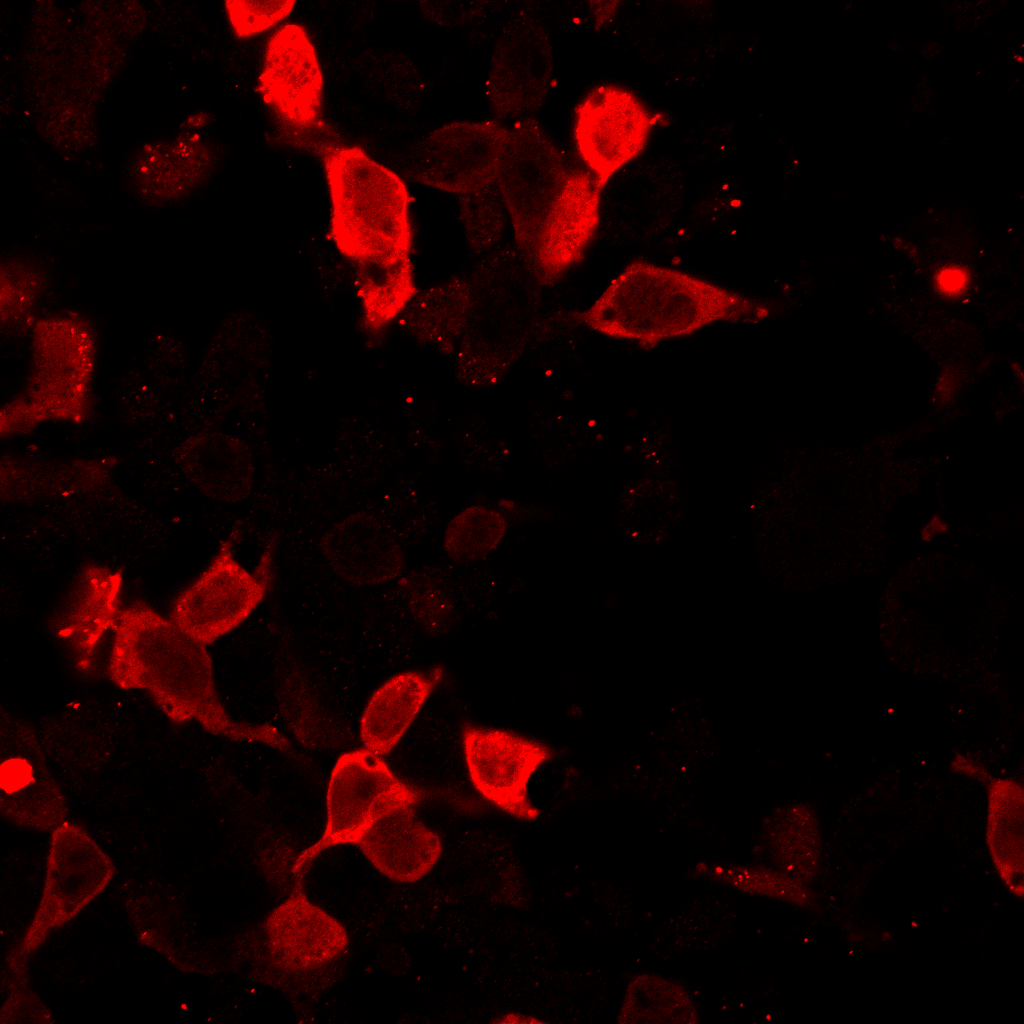

Supplement: S4 Data — (ZIP) [file ppat.1012546.s008.zip › Figure 8-10/Fig8/C/3/Vec+Flag-ASC/Flag-ASC.tif]

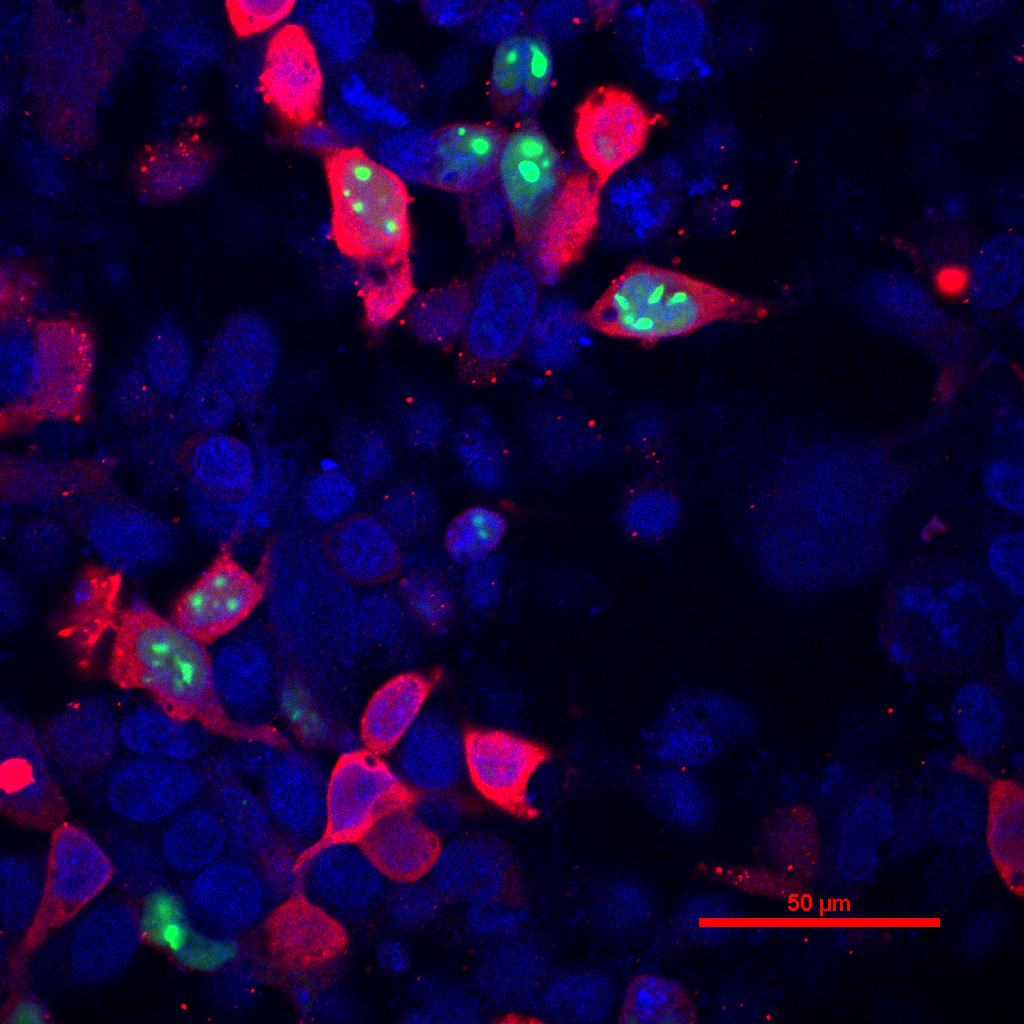

Supplement: S4 Data — (ZIP) [file ppat.1012546.s008.zip › Figure 8-10/Fig8/C/3/Vec+Flag-ASC/Merge.tif]

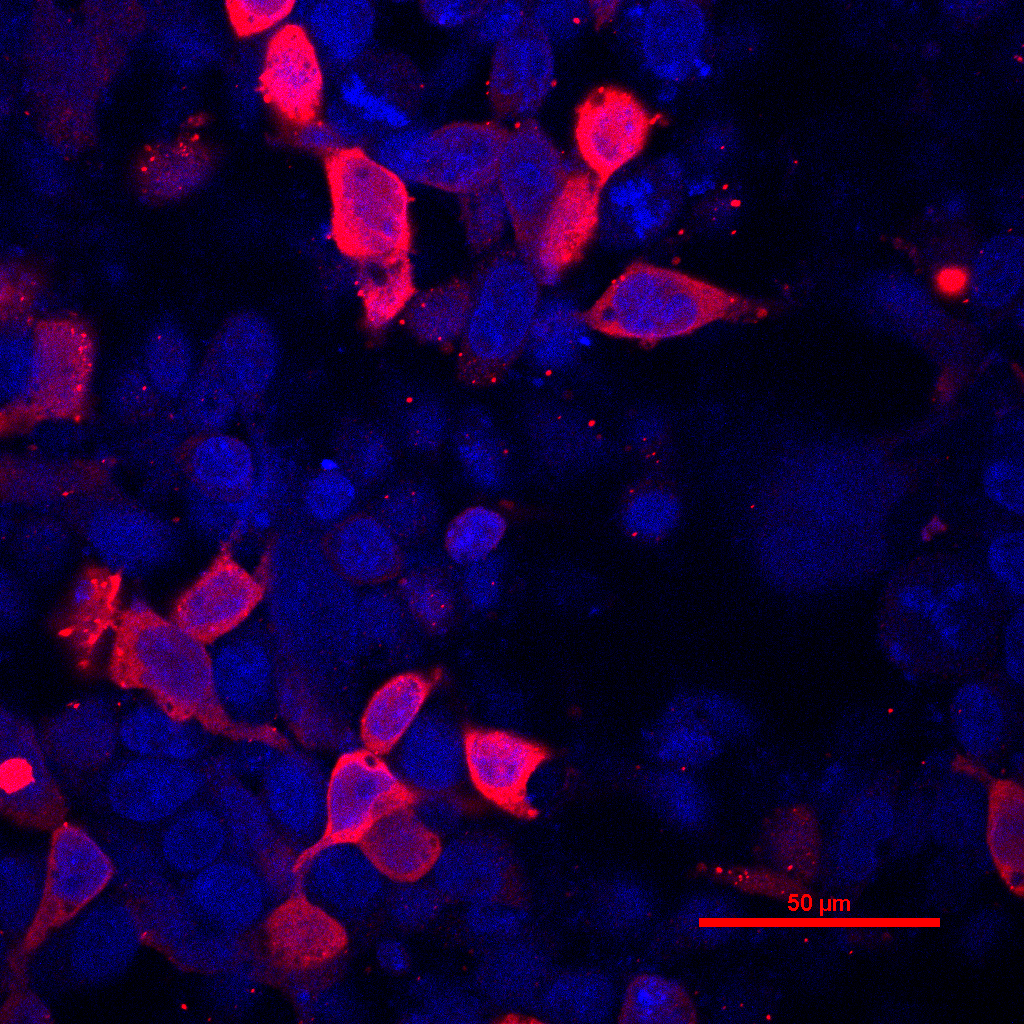

Supplement: S4 Data — (ZIP) [file ppat.1012546.s008.zip › Figure 8-10/Fig8/C/3/Vec+Flag-ASC/RGB.tif]

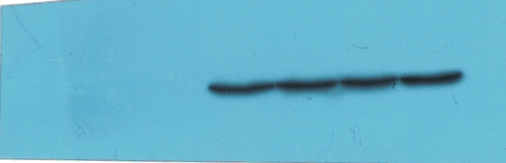

Supplement: S4 Data — (ZIP) [file ppat.1012546.s008.zip › Figure 8-10/Fig8/E/1/Actin.tif]

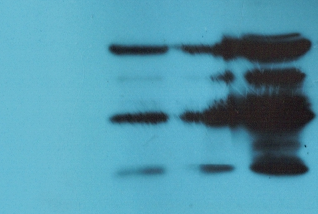

Supplement: S4 Data — (ZIP) [file ppat.1012546.s008.zip › Figure 8-10/Fig8/E/1/ASC╣╤╛█╠σ.tif]

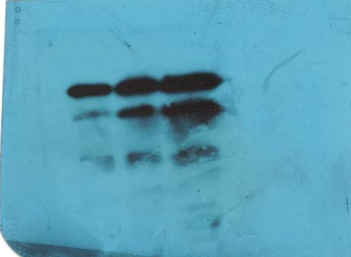

Supplement: S4 Data — (ZIP) [file ppat.1012546.s008.zip › Figure 8-10/Fig8/E/2/DSS-ASC.tif]

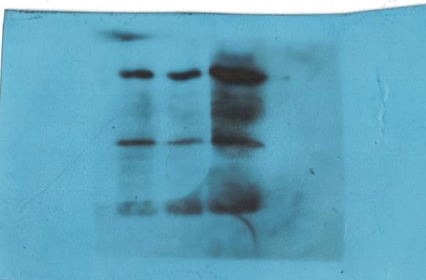

Supplement: S4 Data — (ZIP) [file ppat.1012546.s008.zip › Figure 8-10/Fig8/E/3/DSS-ASC.tif]

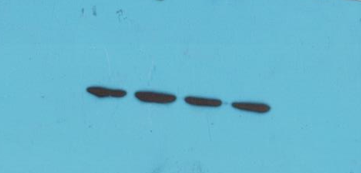

Supplement: S4 Data — (ZIP) [file ppat.1012546.s008.zip › Figure 8-10/Fig8/E/3/WCL-Actin.tif]

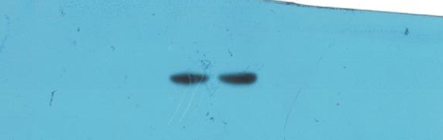

Supplement: S4 Data — (ZIP) [file ppat.1012546.s008.zip › Figure 8-10/Fig8/E/3/WCL-UL4.tif]

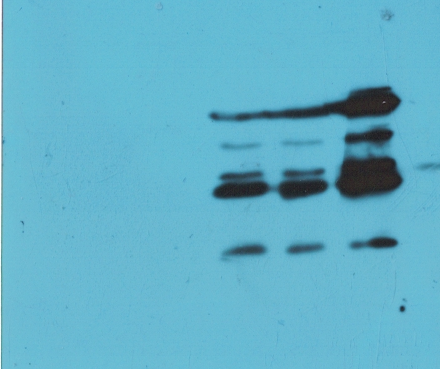

Supplement: S4 Data — (ZIP) [file ppat.1012546.s008.zip › Figure 8-10/Fig8/F/1/ASC╣╤╛█╠σ.tif]

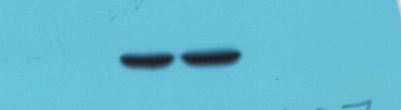

Supplement: S4 Data — (ZIP) [file ppat.1012546.s008.zip › Figure 8-10/Fig8/F/1/UL4.tif]

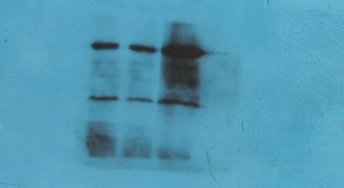

Supplement: S4 Data — (ZIP) [file ppat.1012546.s008.zip › Figure 8-10/Fig8/F/2/DSS-ASC.tif]

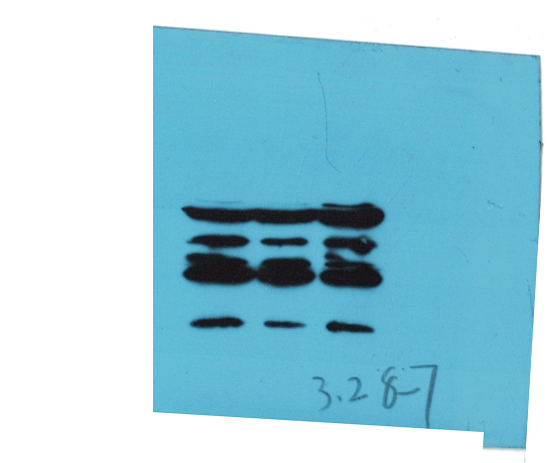

Supplement: S4 Data — (ZIP) [file ppat.1012546.s008.zip › Figure 8-10/Fig8/F/3/DSS-ASC.tif]

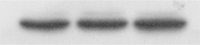

Supplement: S4 Data — (ZIP) [file ppat.1012546.s008.zip › Figure 8-10/Fig8/G/1/Actin.tif]

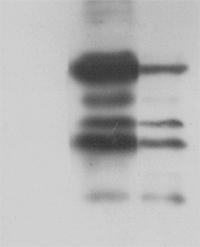

Supplement: S4 Data — (ZIP) [file ppat.1012546.s008.zip › Figure 8-10/Fig8/G/1/DSS-ASC.tif]

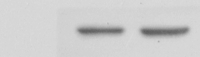

Supplement: S4 Data — (ZIP) [file ppat.1012546.s008.zip › Figure 8-10/Fig8/G/1/UL4.tif]

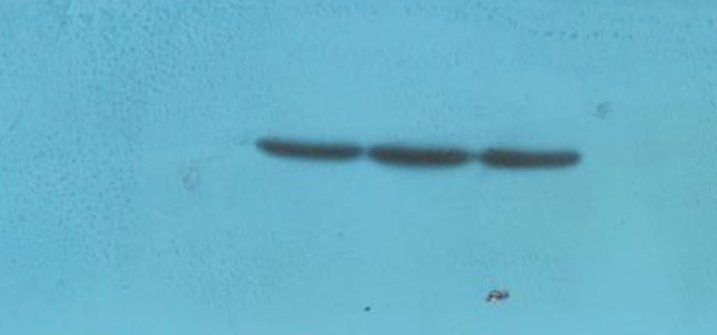

Supplement: S4 Data — (ZIP) [file ppat.1012546.s008.zip › Figure 8-10/Fig8/G/2/WCL-Actin.tif]

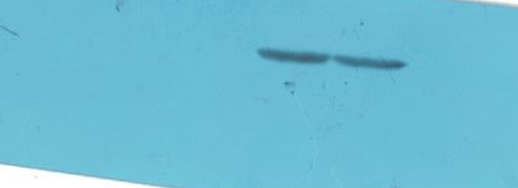

Supplement: S4 Data — (ZIP) [file ppat.1012546.s008.zip › Figure 8-10/Fig8/G/2/WCL-UL4.tif]

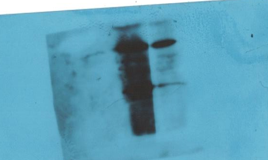

Supplement: S4 Data — (ZIP) [file ppat.1012546.s008.zip › Figure 8-10/Fig8/G/3/DSS-ASC.tif]

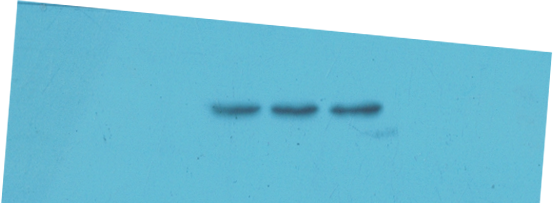

Supplement: S4 Data — (ZIP) [file ppat.1012546.s008.zip › Figure 8-10/Fig8/H/1/Actin.tif]

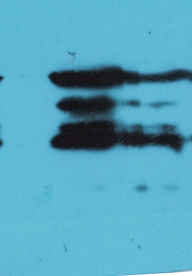

Supplement: S4 Data — (ZIP) [file ppat.1012546.s008.zip › Figure 8-10/Fig8/H/1/ASC.tif]

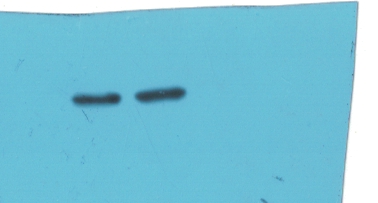

Supplement: S4 Data — (ZIP) [file ppat.1012546.s008.zip › Figure 8-10/Fig8/H/1/UL4.tif]

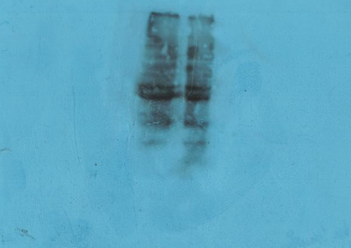

Supplement: S4 Data — (ZIP) [file ppat.1012546.s008.zip › Figure 8-10/Fig8/H/3/DSS-ASC.tif]

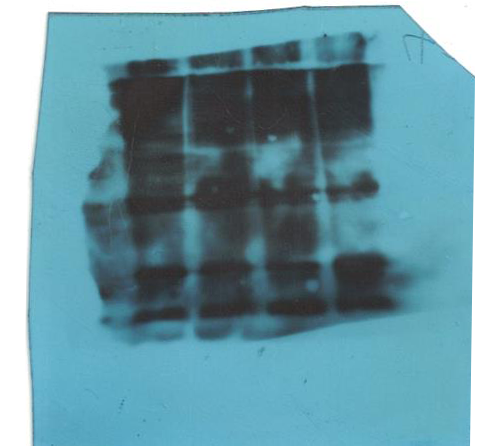

Supplement: S4 Data — (ZIP) [file ppat.1012546.s008.zip › Figure 8-10/Fig9/A/A-1/IP-HA-ub-2.tif]

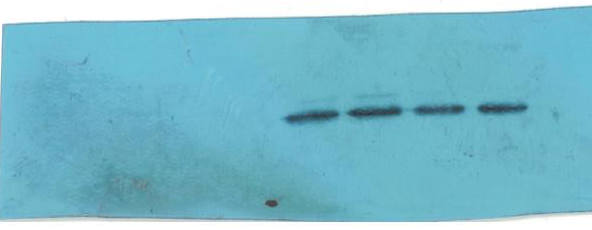

Supplement: S4 Data — (ZIP) [file ppat.1012546.s008.zip › Figure 8-10/Fig9/A/A-1/WCL-Actin-2.tif]

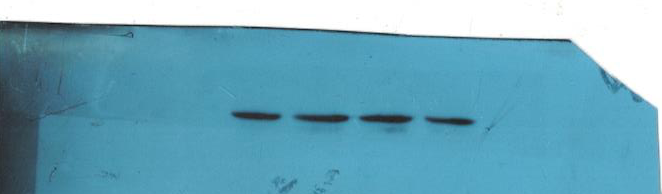

Supplement: S4 Data — (ZIP) [file ppat.1012546.s008.zip › Figure 8-10/Fig9/A/A-1/WCL-ASC-2.tif]

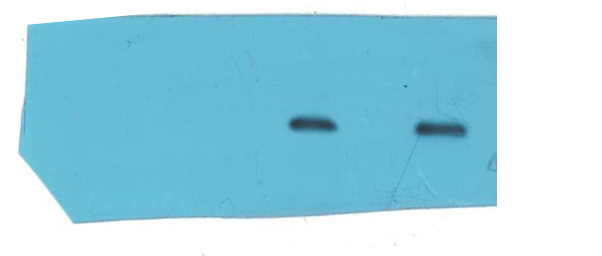

Supplement: S4 Data — (ZIP) [file ppat.1012546.s008.zip › Figure 8-10/Fig9/A/A-1/WCL-UL4-2.tif]

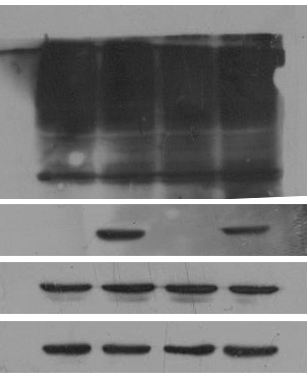

Supplement: S4 Data — (ZIP) [file ppat.1012546.s008.zip › Figure 8-10/Fig9/A/A-2/2.tif]

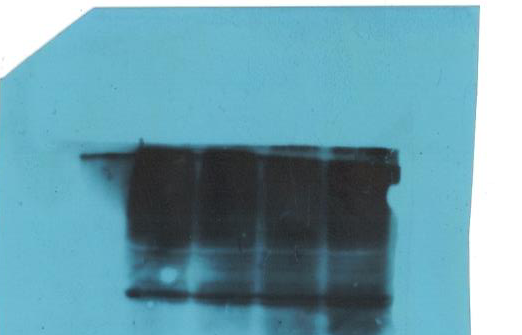

Supplement: S4 Data — (ZIP) [file ppat.1012546.s008.zip › Figure 8-10/Fig9/A/A-2/IP-HA-ub.tif]

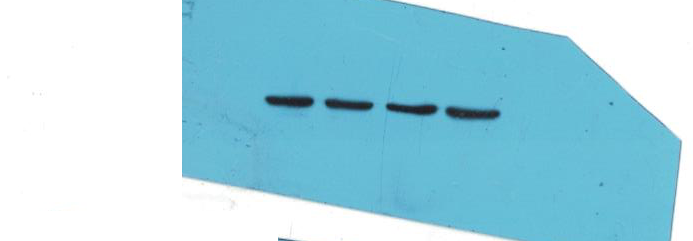

Supplement: S4 Data — (ZIP) [file ppat.1012546.s008.zip › Figure 8-10/Fig9/A/A-2/WCL-Actin.tif]

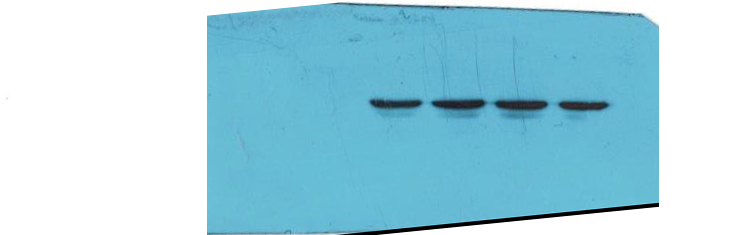

Supplement: S4 Data — (ZIP) [file ppat.1012546.s008.zip › Figure 8-10/Fig9/A/A-2/WCL-ASC.tif]

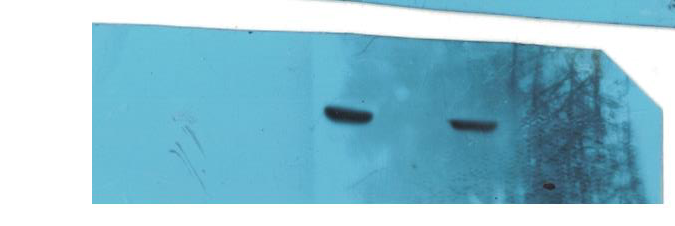

Supplement: S4 Data — (ZIP) [file ppat.1012546.s008.zip › Figure 8-10/Fig9/A/A-2/WCL-UL4.tif]

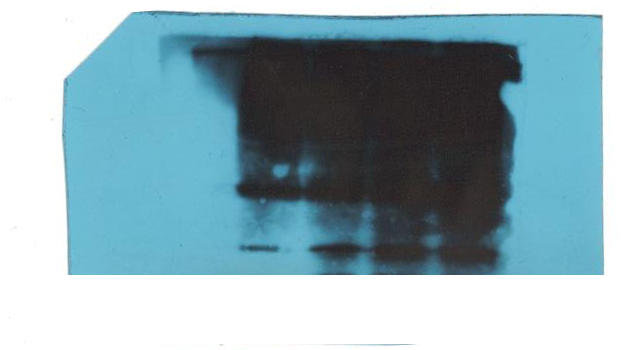

Supplement: S4 Data — (ZIP) [file ppat.1012546.s008.zip › Figure 8-10/Fig9/A/A-3/IP-HA-ub.tif]

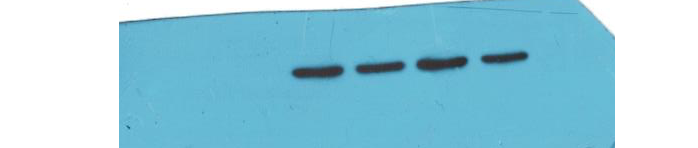

Supplement: S4 Data — (ZIP) [file ppat.1012546.s008.zip › Figure 8-10/Fig9/A/A-3/WCL-Actin.tif]

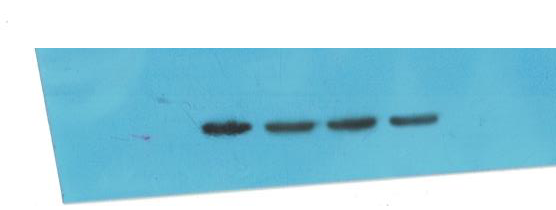

Supplement: S4 Data — (ZIP) [file ppat.1012546.s008.zip › Figure 8-10/Fig9/A/A-3/WCL-ASC.tif]

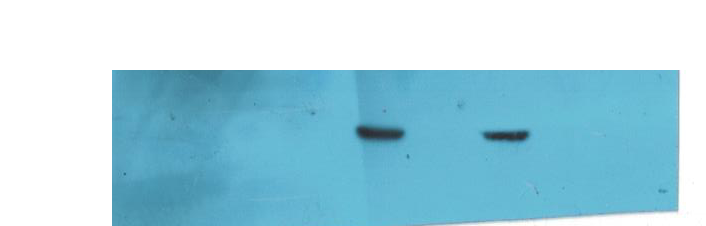

Supplement: S4 Data — (ZIP) [file ppat.1012546.s008.zip › Figure 8-10/Fig9/A/A-3/WCL-UL4.tif]

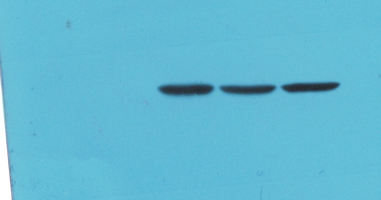

Supplement: S4 Data — (ZIP) [file ppat.1012546.s008.zip › Figure 8-10/Fig9/B/B-1/Actin.tif]

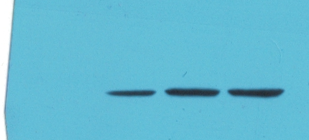

Supplement: S4 Data — (ZIP) [file ppat.1012546.s008.zip › Figure 8-10/Fig9/B/B-1/ASC.tif]

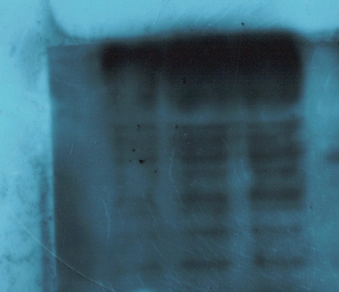

Supplement: S4 Data — (ZIP) [file ppat.1012546.s008.zip › Figure 8-10/Fig9/B/B-1/UB.tif]

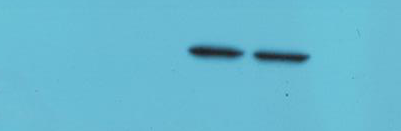

Supplement: S4 Data — (ZIP) [file ppat.1012546.s008.zip › Figure 8-10/Fig9/B/B-1/UL4.tif]

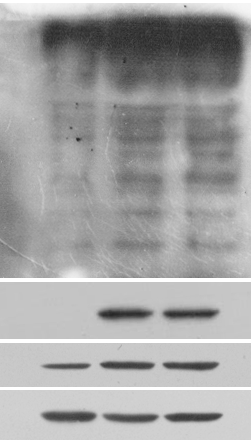

Supplement: S4 Data — (ZIP) [file ppat.1012546.s008.zip › Figure 8-10/Fig9/B/B-1/UL4▓╗╙░╧∞3d╖║╦╪╗»╦«╞╜.tif]

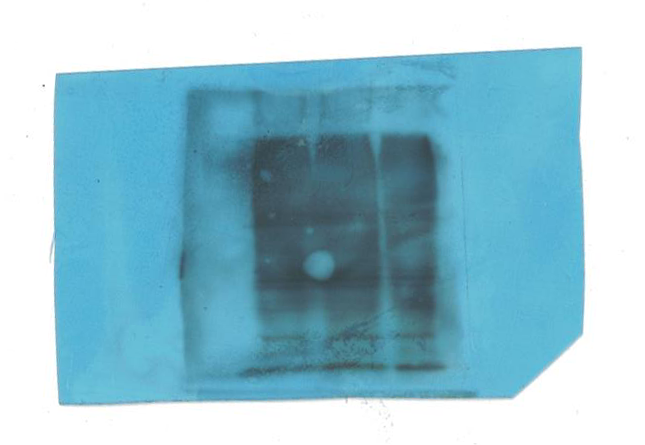

Supplement: S4 Data — (ZIP) [file ppat.1012546.s008.zip › Figure 8-10/Fig9/B/B-2/HA-ub.tif]

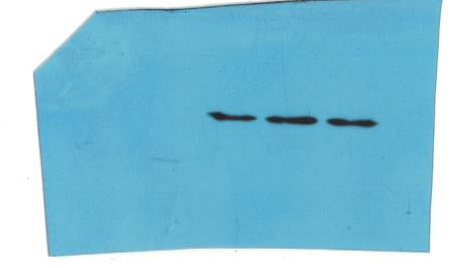

Supplement: S4 Data — (ZIP) [file ppat.1012546.s008.zip › Figure 8-10/Fig9/B/B-2/WCL-Actin.tif]

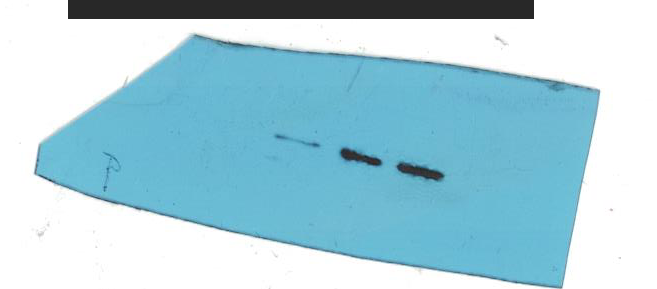

Supplement: S4 Data — (ZIP) [file ppat.1012546.s008.zip › Figure 8-10/Fig9/B/B-2/WCL-ASC.tif]

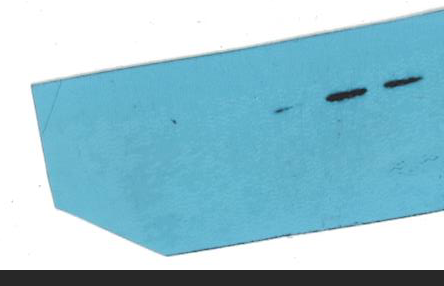

Supplement: S4 Data — (ZIP) [file ppat.1012546.s008.zip › Figure 8-10/Fig9/B/B-3/Asc.tif]

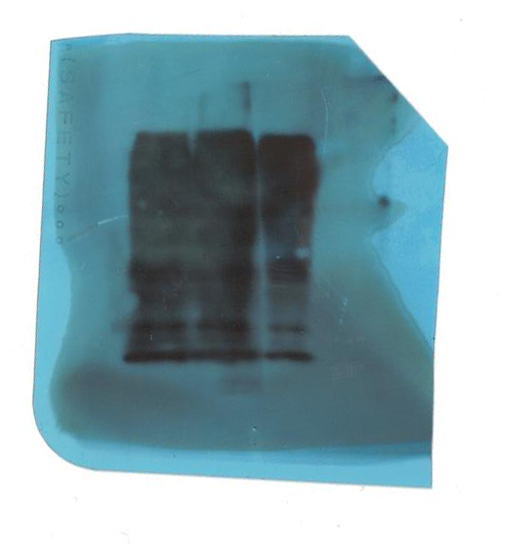

Supplement: S4 Data — (ZIP) [file ppat.1012546.s008.zip › Figure 8-10/Fig9/B/B-3/HA-ub.tif]

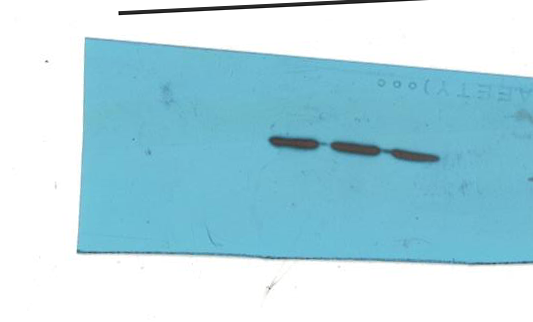

Supplement: S4 Data — (ZIP) [file ppat.1012546.s008.zip › Figure 8-10/Fig9/B/B-3/WCL-Actin.tif]

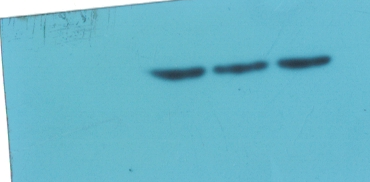

Supplement: S4 Data — (ZIP) [file ppat.1012546.s008.zip › Figure 8-10/Fig9/C/C-1/Actin.tif]

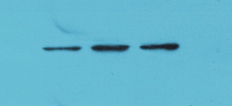

Supplement: S4 Data — (ZIP) [file ppat.1012546.s008.zip › Figure 8-10/Fig9/C/C-1/ASC.tif]

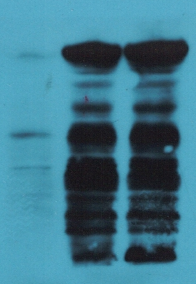

Supplement: S4 Data — (ZIP) [file ppat.1012546.s008.zip › Figure 8-10/Fig9/C/C-1/UB.tif]

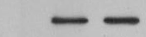

Supplement: S4 Data — (ZIP) [file ppat.1012546.s008.zip › Figure 8-10/Fig9/C/C-1/UL4.tif]

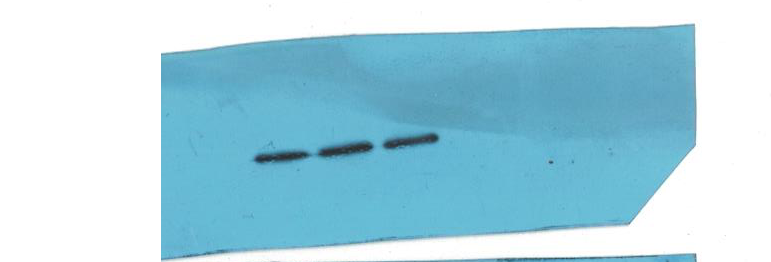

Supplement: S4 Data — (ZIP) [file ppat.1012546.s008.zip › Figure 8-10/Fig9/C/C-2/Actin.tif]

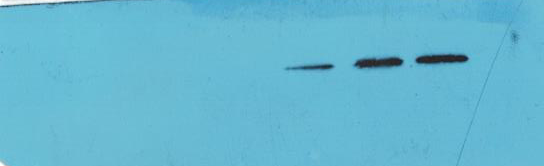

Supplement: S4 Data — (ZIP) [file ppat.1012546.s008.zip › Figure 8-10/Fig9/C/C-2/ASC.tif]

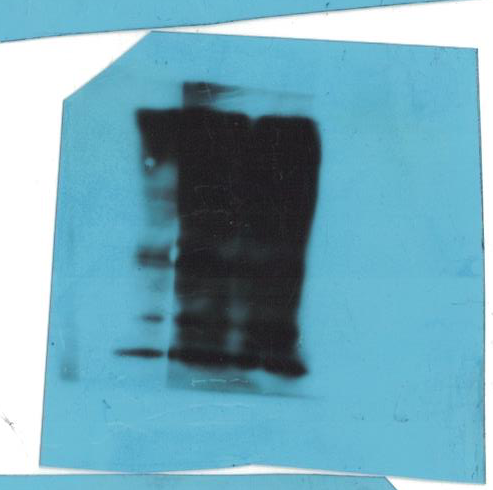

Supplement: S4 Data — (ZIP) [file ppat.1012546.s008.zip › Figure 8-10/Fig9/C/C-2/ub.tif]

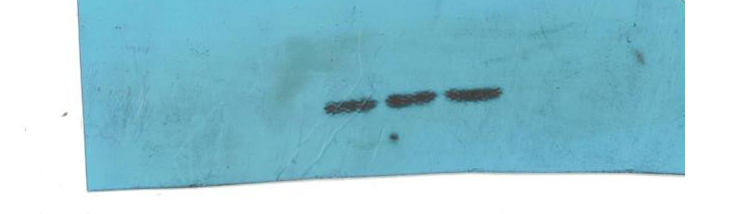

Supplement: S4 Data — (ZIP) [file ppat.1012546.s008.zip › Figure 8-10/Fig9/C/C-3/Actin.tif]

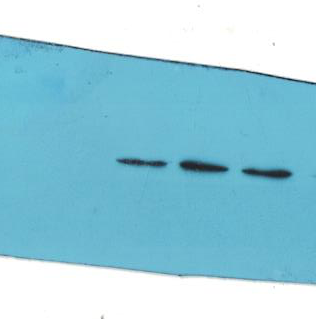

Supplement: S4 Data — (ZIP) [file ppat.1012546.s008.zip › Figure 8-10/Fig9/C/C-3/ASC.tif]

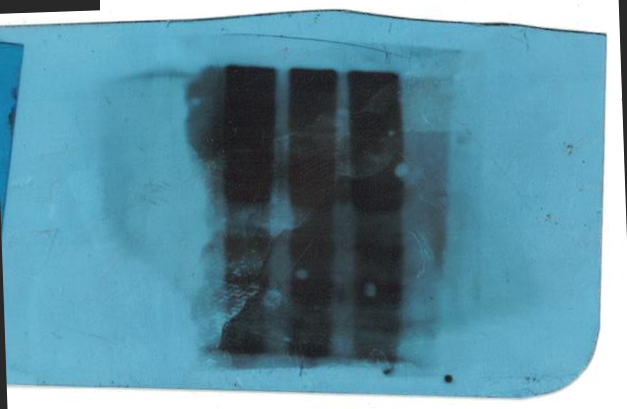

Supplement: S4 Data — (ZIP) [file ppat.1012546.s008.zip › Figure 8-10/Fig9/C/C-3/UB.tif]

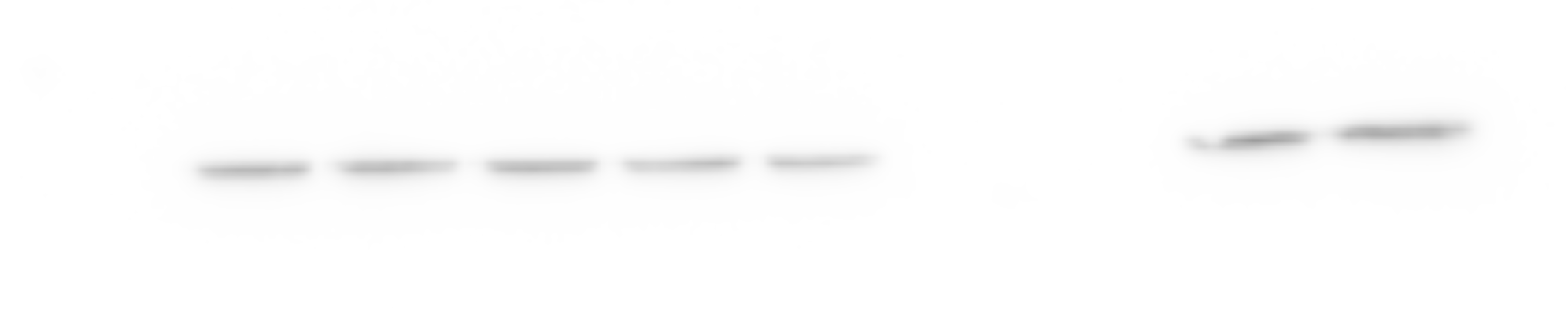

Supplement: S5 Data — (ZIP) [file ppat.1012546.s009.zip › Figure S1-4/FigS1/A/A-1/Actin/Actin-1.tif]

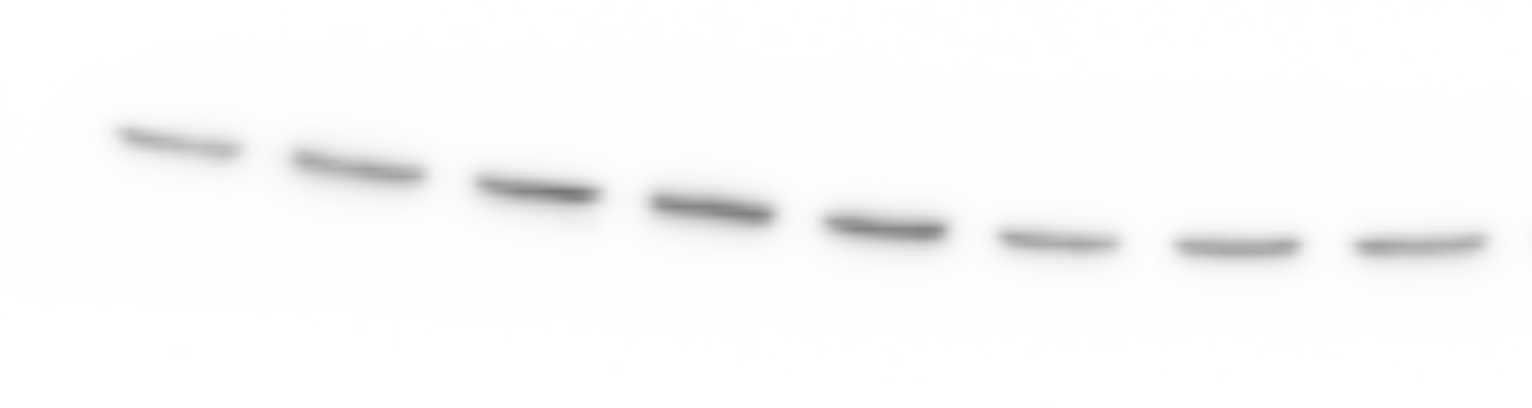

Supplement: S5 Data — (ZIP) [file ppat.1012546.s009.zip › Figure S1-4/FigS1/A/A-1/Actin/Actin-10.tif]

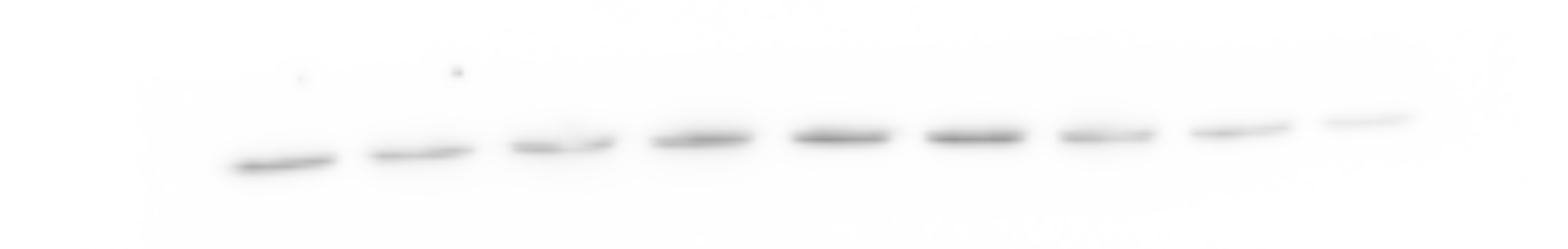

Supplement: S5 Data — (ZIP) [file ppat.1012546.s009.zip › Figure S1-4/FigS1/A/A-1/Actin/Actin-2.tif]

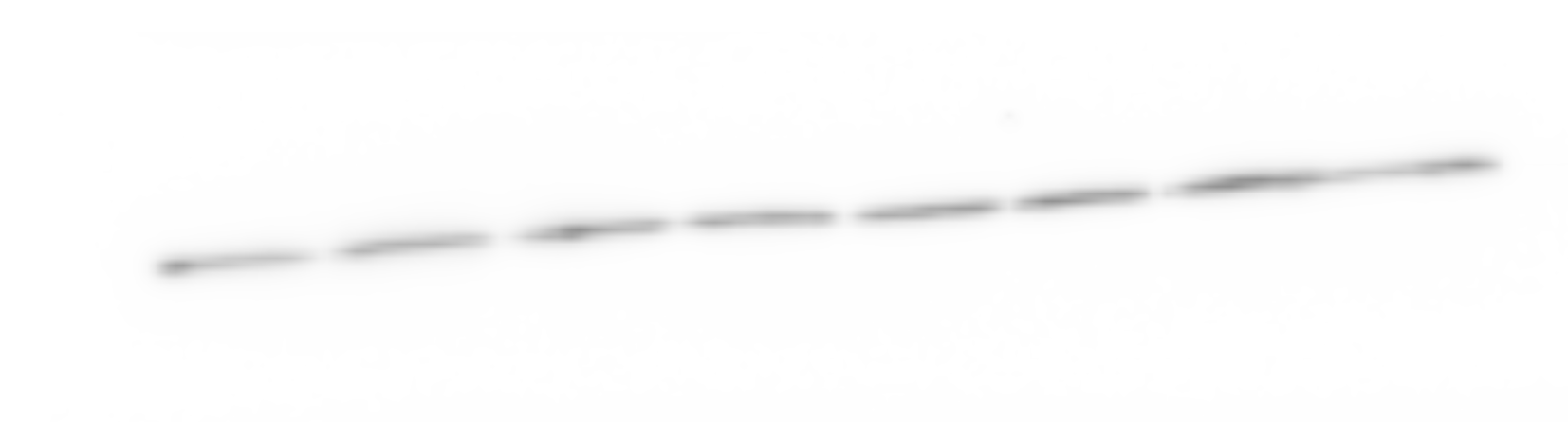

Supplement: S5 Data — (ZIP) [file ppat.1012546.s009.zip › Figure S1-4/FigS1/A/A-1/Actin/Actin-3.tif]

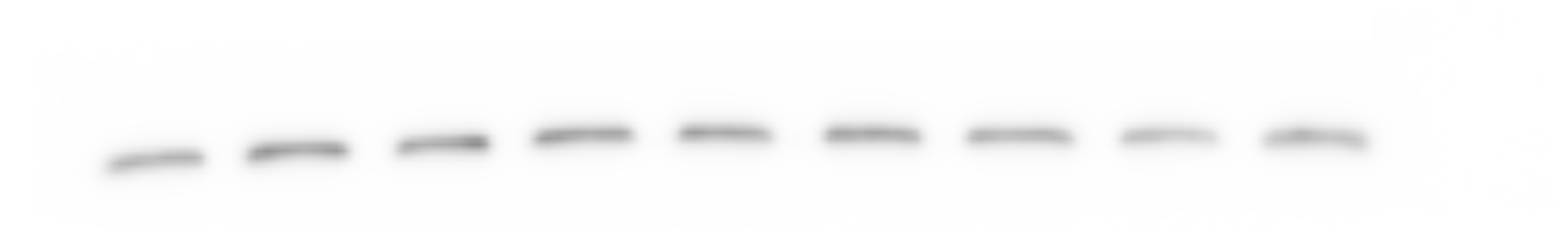

Supplement: S5 Data — (ZIP) [file ppat.1012546.s009.zip › Figure S1-4/FigS1/A/A-1/Actin/Actin-4.tif]

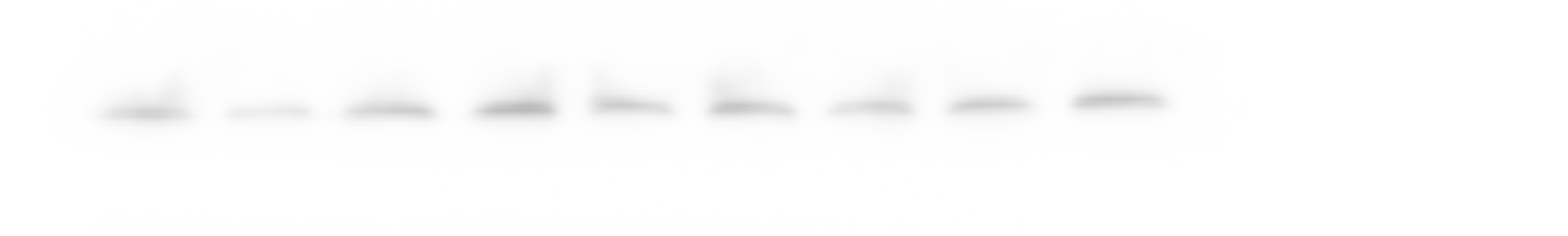

Supplement: S5 Data — (ZIP) [file ppat.1012546.s009.zip › Figure S1-4/FigS1/A/A-1/Actin/Actin-5.tif]

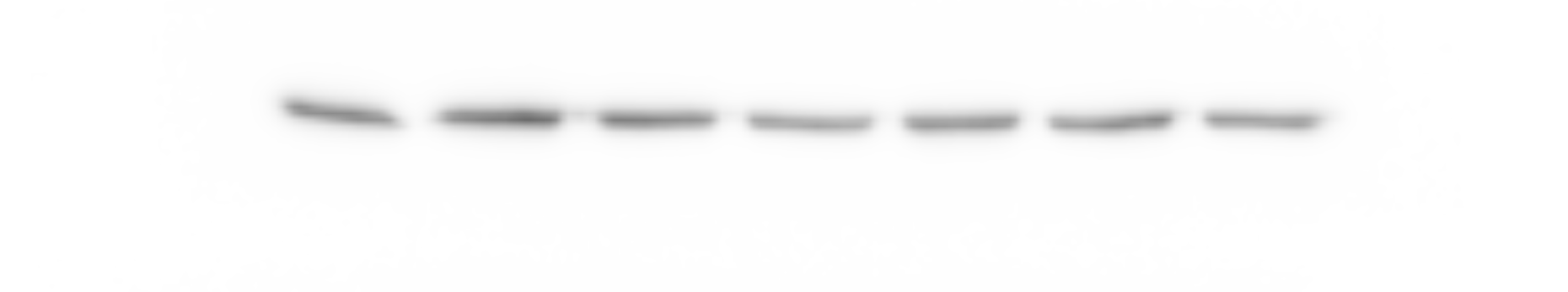

Supplement: S5 Data — (ZIP) [file ppat.1012546.s009.zip › Figure S1-4/FigS1/A/A-1/Actin/Actin-6.tif]

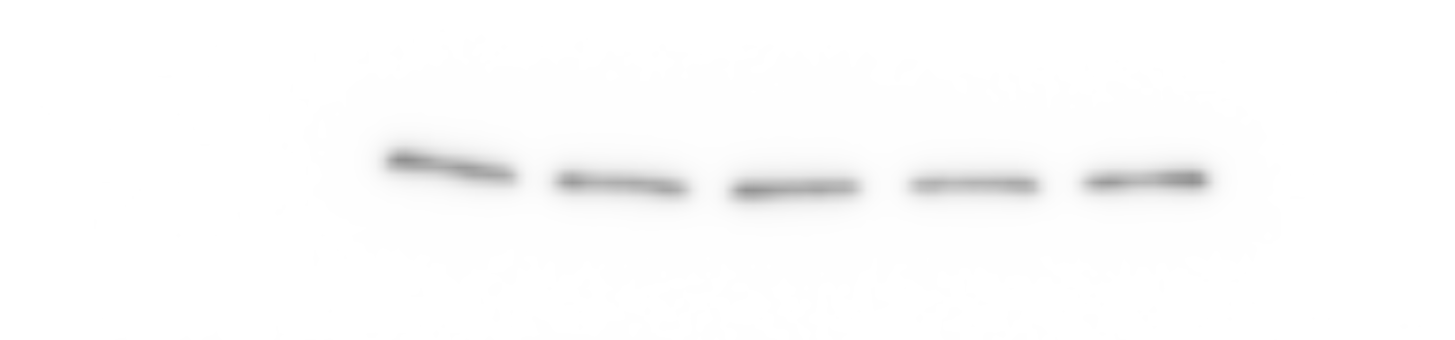

Supplement: S5 Data — (ZIP) [file ppat.1012546.s009.zip › Figure S1-4/FigS1/A/A-1/Actin/Actin-7.tif]

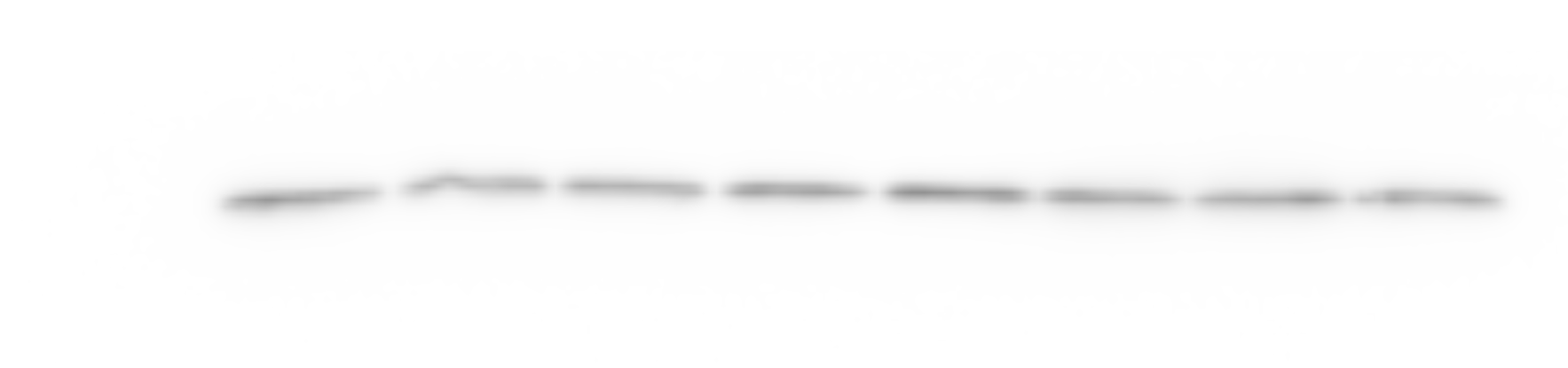

Supplement: S5 Data — (ZIP) [file ppat.1012546.s009.zip › Figure S1-4/FigS1/A/A-1/Actin/Actin-8.tif]

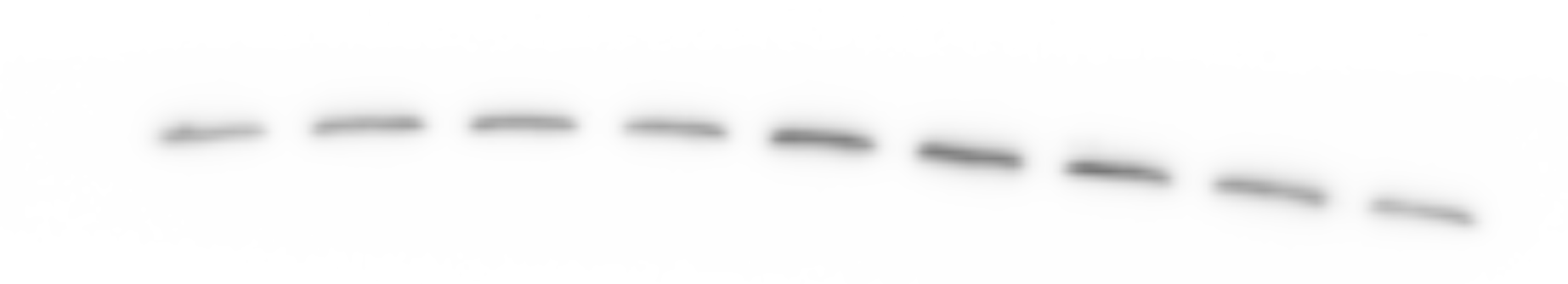

Supplement: S5 Data — (ZIP) [file ppat.1012546.s009.zip › Figure S1-4/FigS1/A/A-1/Actin/Actin-9.tif]

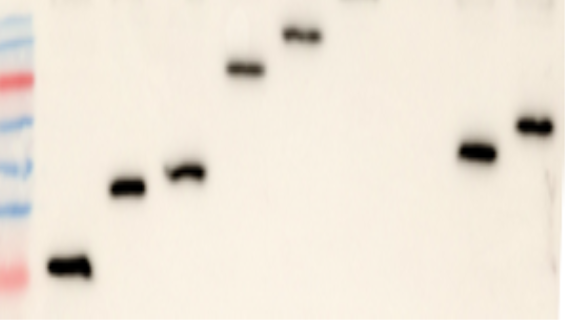

Supplement: S5 Data — (ZIP) [file ppat.1012546.s009.zip › Figure S1-4/FigS1/A/A-1/Anti-GFP/1.tif]

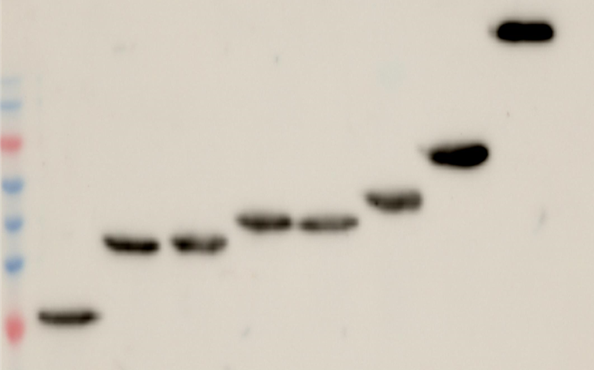

Supplement: S5 Data — (ZIP) [file ppat.1012546.s009.zip › Figure S1-4/FigS1/A/A-1/Anti-GFP/10.tif]

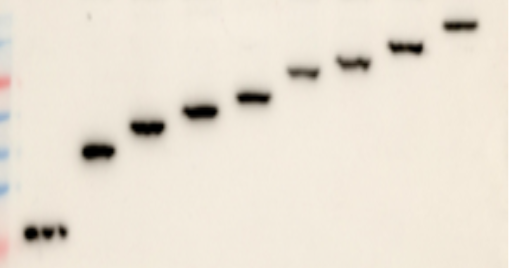

Supplement: S5 Data — (ZIP) [file ppat.1012546.s009.zip › Figure S1-4/FigS1/A/A-1/Anti-GFP/2.tif]

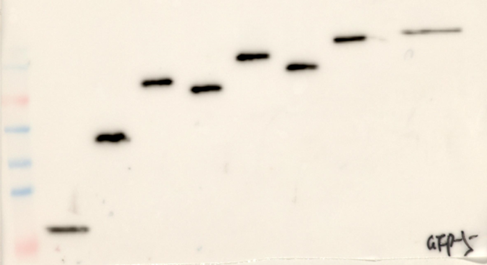

Supplement: S5 Data — (ZIP) [file ppat.1012546.s009.zip › Figure S1-4/FigS1/A/A-1/Anti-GFP/3.tif]

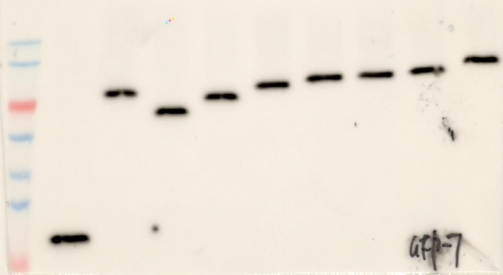

Supplement: S5 Data — (ZIP) [file ppat.1012546.s009.zip › Figure S1-4/FigS1/A/A-1/Anti-GFP/4.tif]

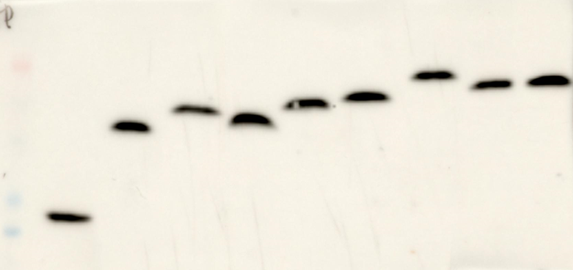

Supplement: S5 Data — (ZIP) [file ppat.1012546.s009.zip › Figure S1-4/FigS1/A/A-1/Anti-GFP/5.tif]

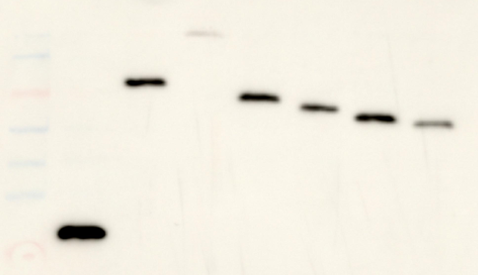

Supplement: S5 Data — (ZIP) [file ppat.1012546.s009.zip › Figure S1-4/FigS1/A/A-1/Anti-GFP/6.tif]

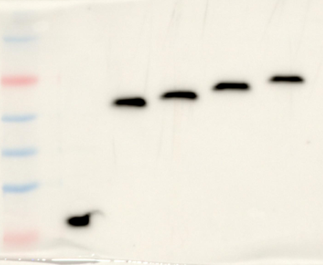

Supplement: S5 Data — (ZIP) [file ppat.1012546.s009.zip › Figure S1-4/FigS1/A/A-1/Anti-GFP/7.tif]

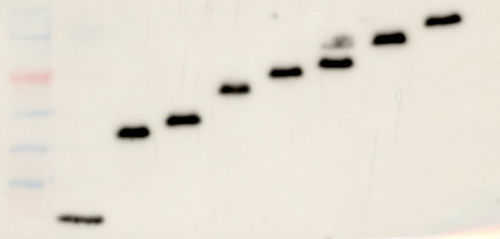

Supplement: S5 Data — (ZIP) [file ppat.1012546.s009.zip › Figure S1-4/FigS1/A/A-1/Anti-GFP/8.tif]

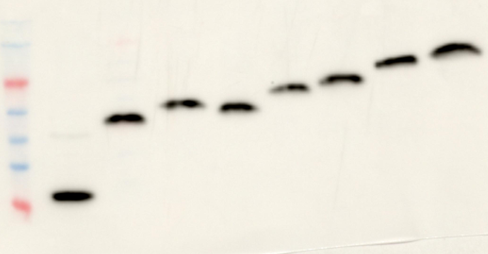

Supplement: S5 Data — (ZIP) [file ppat.1012546.s009.zip › Figure S1-4/FigS1/A/A-1/Anti-GFP/9.tif]

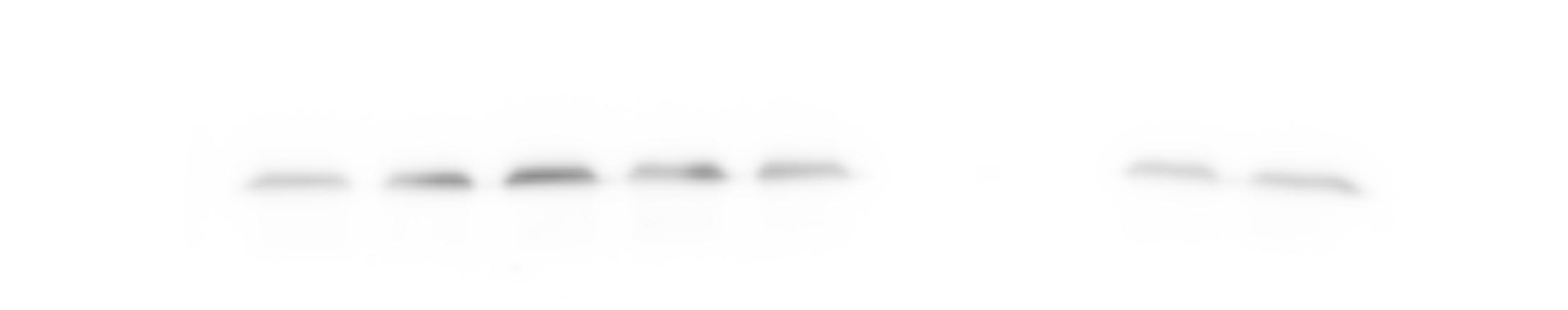

Supplement: S5 Data — (ZIP) [file ppat.1012546.s009.zip › Figure S1-4/FigS1/A/A-2/Actin/1.tif]

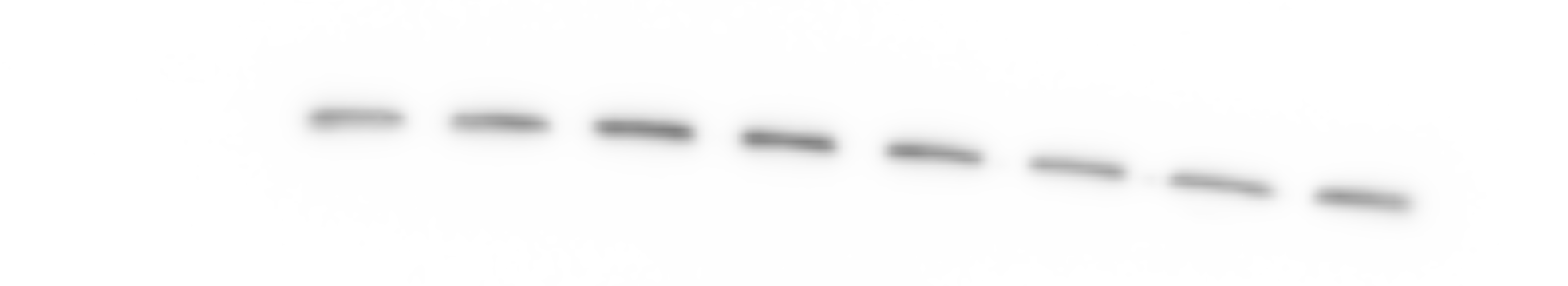

Supplement: S5 Data — (ZIP) [file ppat.1012546.s009.zip › Figure S1-4/FigS1/A/A-2/Actin/10.tif]

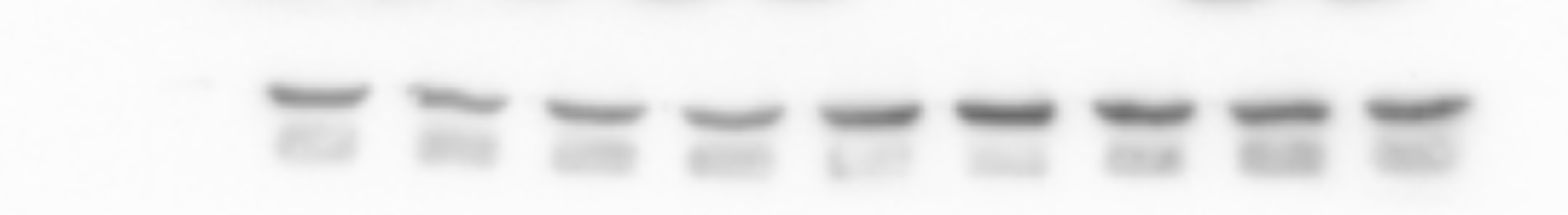

Supplement: S5 Data — (ZIP) [file ppat.1012546.s009.zip › Figure S1-4/FigS1/A/A-2/Actin/2.tif]

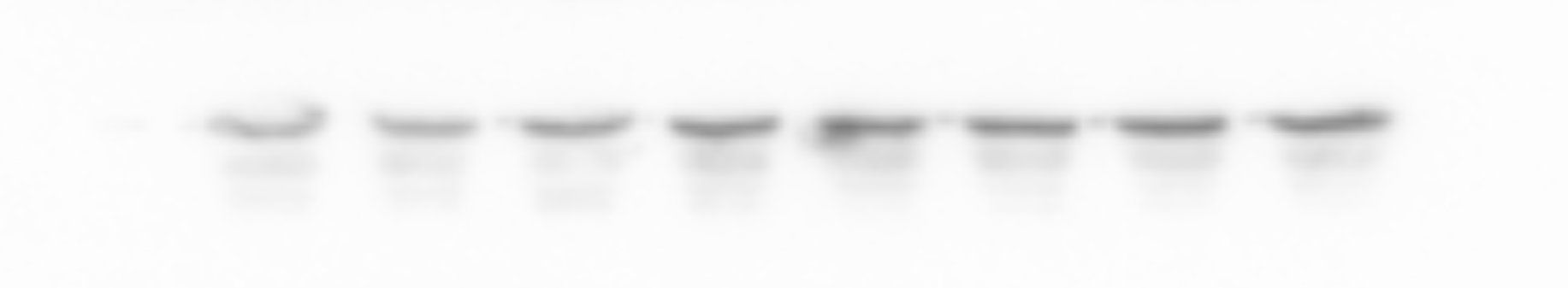

Supplement: S5 Data — (ZIP) [file ppat.1012546.s009.zip › Figure S1-4/FigS1/A/A-2/Actin/3.tif]

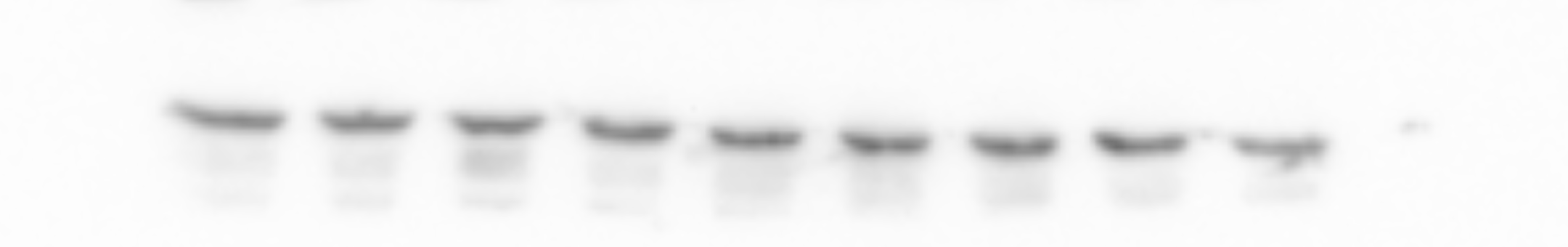

Supplement: S5 Data — (ZIP) [file ppat.1012546.s009.zip › Figure S1-4/FigS1/A/A-2/Actin/4.tif]

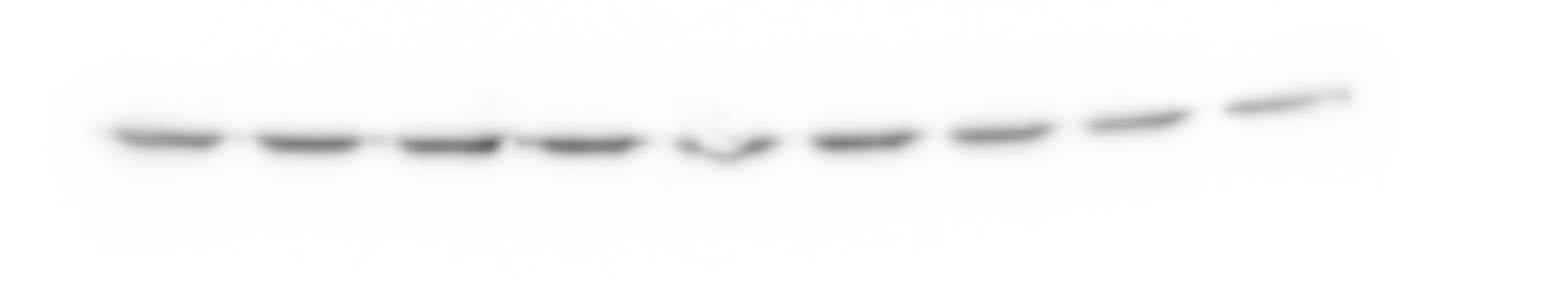

Supplement: S5 Data — (ZIP) [file ppat.1012546.s009.zip › Figure S1-4/FigS1/A/A-2/Actin/5.tif]

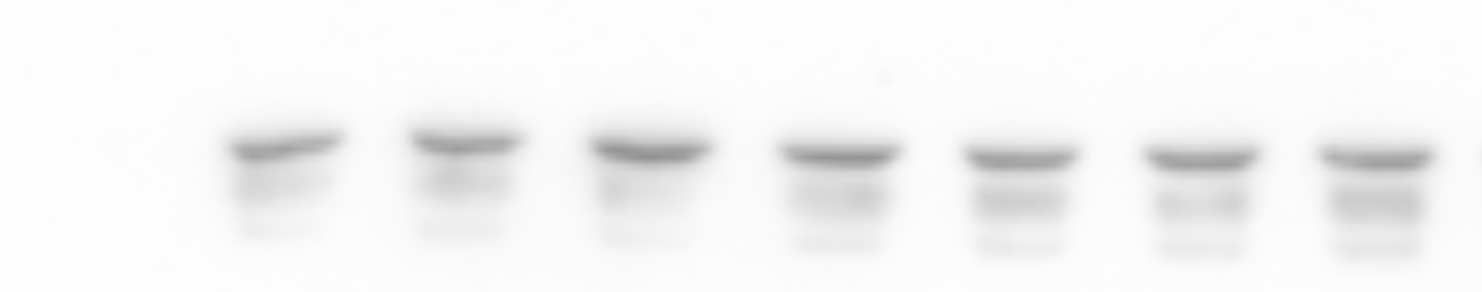

Supplement: S5 Data — (ZIP) [file ppat.1012546.s009.zip › Figure S1-4/FigS1/A/A-2/Actin/6.tif]

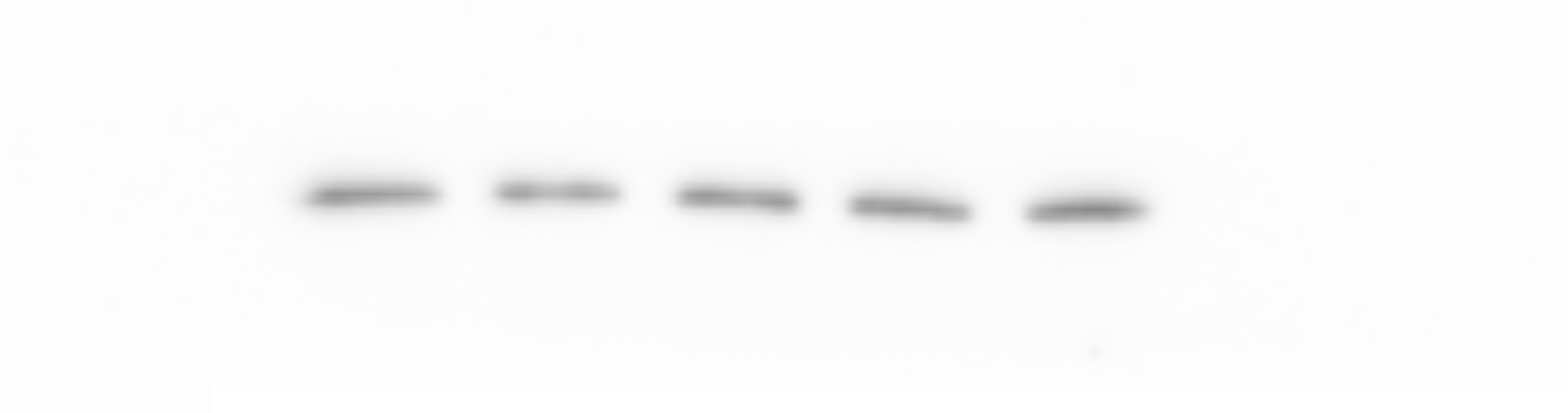

Supplement: S5 Data — (ZIP) [file ppat.1012546.s009.zip › Figure S1-4/FigS1/A/A-2/Actin/7.tif]

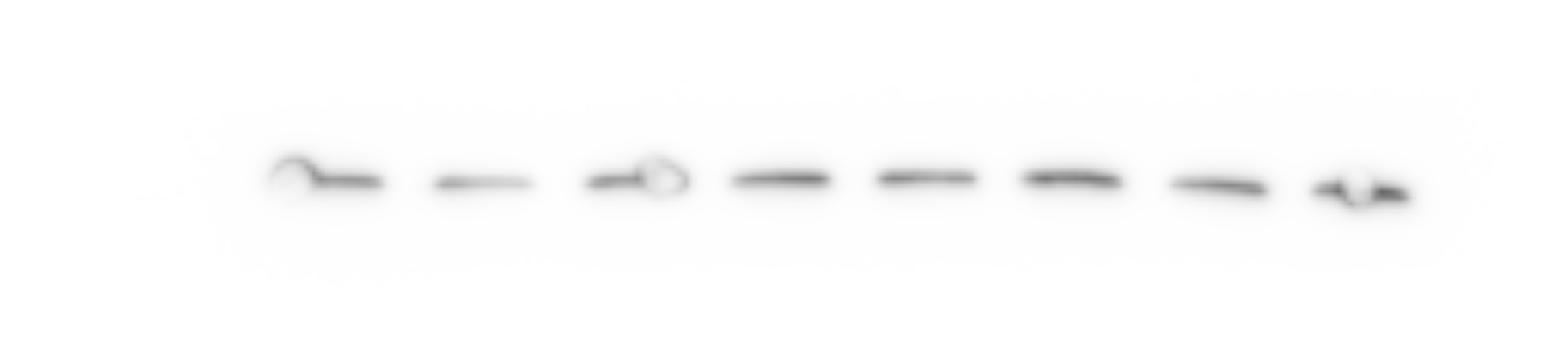

Supplement: S5 Data — (ZIP) [file ppat.1012546.s009.zip › Figure S1-4/FigS1/A/A-2/Actin/8.tif]

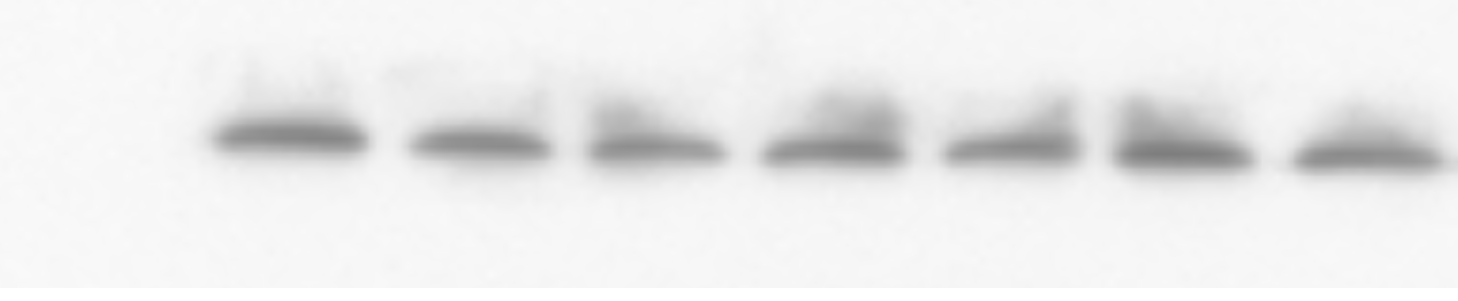

Supplement: S5 Data — (ZIP) [file ppat.1012546.s009.zip › Figure S1-4/FigS1/A/A-2/Actin/9.tif]

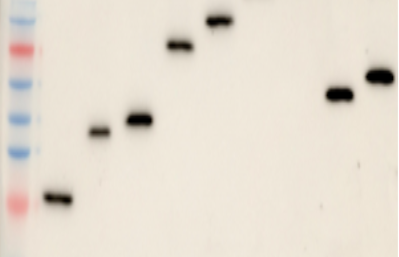

Supplement: S5 Data — (ZIP) [file ppat.1012546.s009.zip › Figure S1-4/FigS1/A/A-2/Anti-GFP/1.tif]

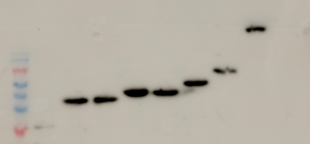

Supplement: S5 Data — (ZIP) [file ppat.1012546.s009.zip › Figure S1-4/FigS1/A/A-2/Anti-GFP/10.tif]

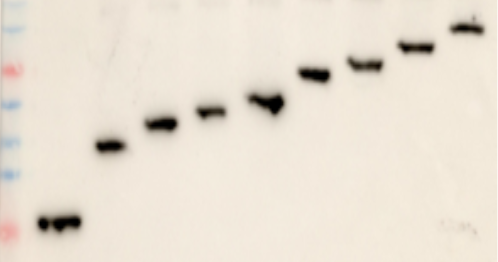

Supplement: S5 Data — (ZIP) [file ppat.1012546.s009.zip › Figure S1-4/FigS1/A/A-2/Anti-GFP/2.tif]

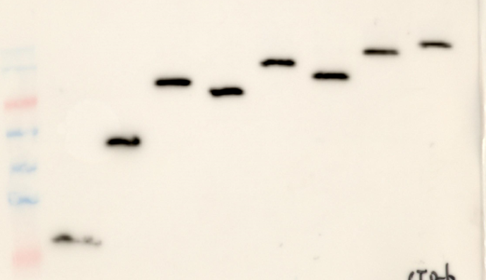

Supplement: S5 Data — (ZIP) [file ppat.1012546.s009.zip › Figure S1-4/FigS1/A/A-2/Anti-GFP/3.tif]

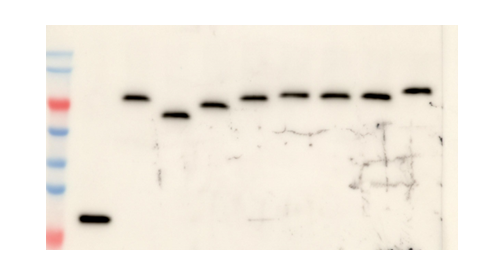

Supplement: S5 Data — (ZIP) [file ppat.1012546.s009.zip › Figure S1-4/FigS1/A/A-2/Anti-GFP/4.tif]

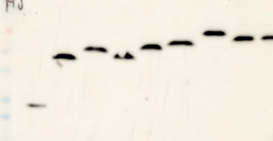

Supplement: S5 Data — (ZIP) [file ppat.1012546.s009.zip › Figure S1-4/FigS1/A/A-2/Anti-GFP/5.tif]

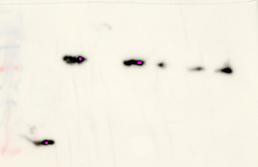

Supplement: S5 Data — (ZIP) [file ppat.1012546.s009.zip › Figure S1-4/FigS1/A/A-2/Anti-GFP/6.tif]

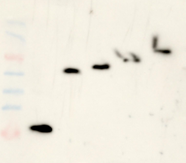

Supplement: S5 Data — (ZIP) [file ppat.1012546.s009.zip › Figure S1-4/FigS1/A/A-2/Anti-GFP/7.tif]

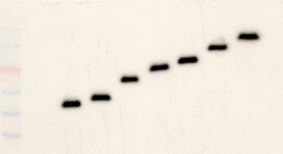

Supplement: S5 Data — (ZIP) [file ppat.1012546.s009.zip › Figure S1-4/FigS1/A/A-2/Anti-GFP/8.tif]

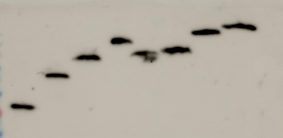

Supplement: S5 Data — (ZIP) [file ppat.1012546.s009.zip › Figure S1-4/FigS1/A/A-2/Anti-GFP/9.tif]

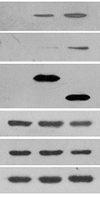

Supplement: S5 Data — (ZIP) [file ppat.1012546.s009.zip › Figure S1-4/FigS1/B/B-1/293t-╫¬╚╛╞Σ╦√▓í╢╛╡░░╫-1.tif]

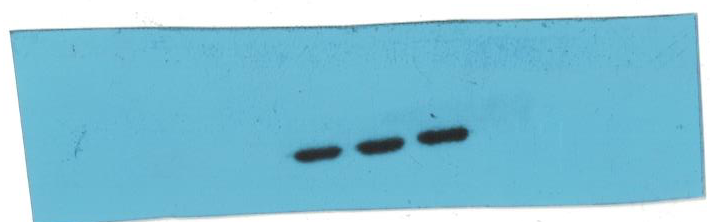

Supplement: S5 Data — (ZIP) [file ppat.1012546.s009.zip › Figure S1-4/FigS1/B/B-1/FigS1-Actin.tif]

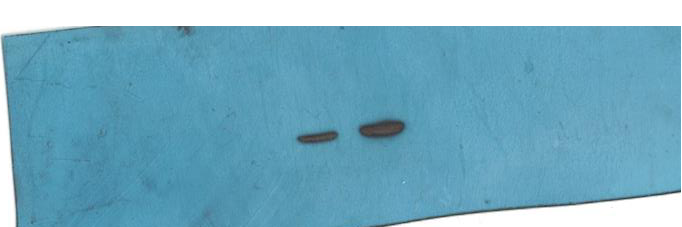

Supplement: S5 Data — (ZIP) [file ppat.1012546.s009.zip › Figure S1-4/FigS1/B/B-1/Sup-CASP1 p10.tif]
