# Supplementary figures and images for: Pseudorabies Virus UL4 protein promotes the ASC-dependent inflammasome activation and pyroptosis to exacerbate inflammation (part 6 of 6)
Source: PLoS Pathog. 2024 Sep 24;20(9):e1012546. doi: 10.1371/journal.ppat.1012546 (PMC11421794; doi:10.1371/journal.ppat.1012546)

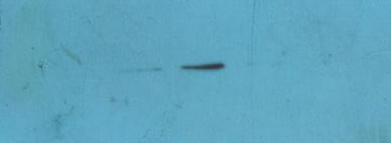

Supplement: S5 Data — (ZIP) [file ppat.1012546.s009.zip › Figure S1-4/FigS1/B/B-1/Sup-IL-1a┬ P17.tif]

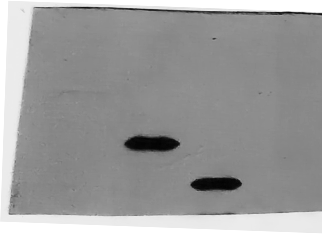

Supplement: S5 Data — (ZIP) [file ppat.1012546.s009.zip › Figure S1-4/FigS1/B/B-1/WCL-GFP.tif]

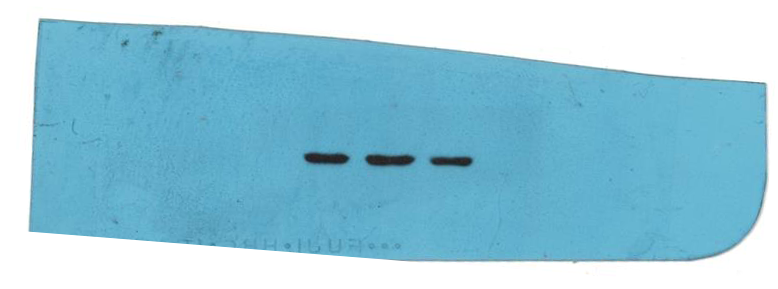

Supplement: S5 Data — (ZIP) [file ppat.1012546.s009.zip › Figure S1-4/FigS1/B/B-1/WCL-Pro-CASP1.tif]

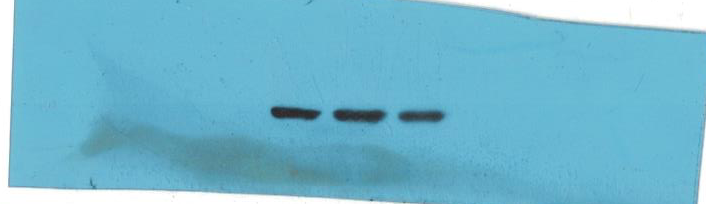

Supplement: S5 Data — (ZIP) [file ppat.1012546.s009.zip › Figure S1-4/FigS1/B/B-1/WCL-Pro-IL-la┬.tif]

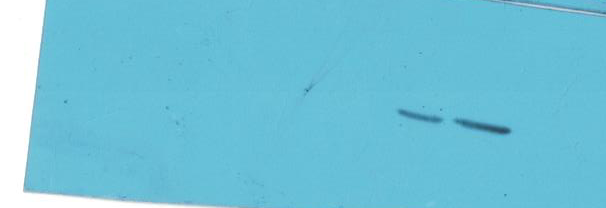

Supplement: S5 Data — (ZIP) [file ppat.1012546.s009.zip › Figure S1-4/FigS1/B/B-2/Sup-CASP1 p10.tif]

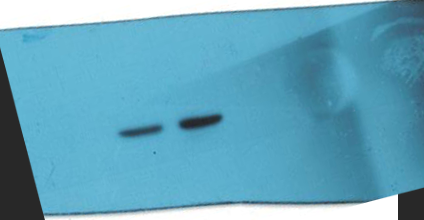

Supplement: S5 Data — (ZIP) [file ppat.1012546.s009.zip › Figure S1-4/FigS1/B/B-2/Sup-IL-1a┬ P17.tif]

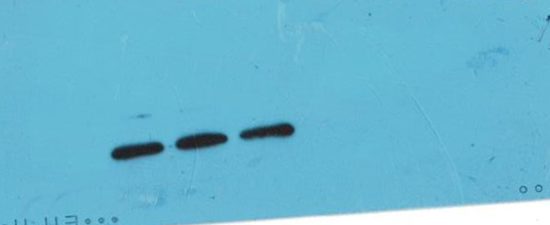

Supplement: S5 Data — (ZIP) [file ppat.1012546.s009.zip › Figure S1-4/FigS1/B/B-2/WCL-Actin.tif]

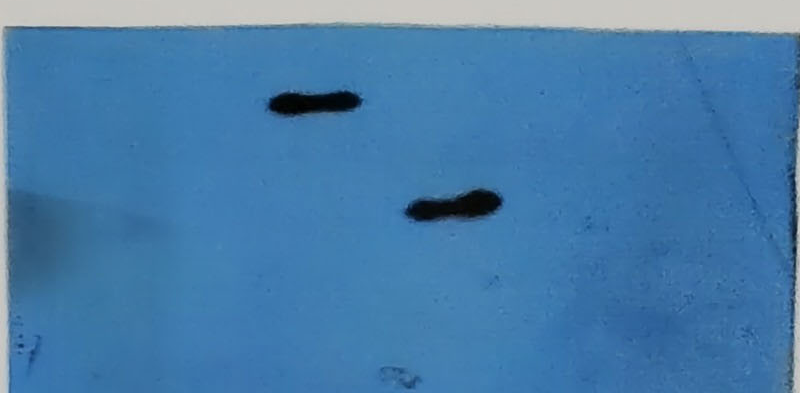

Supplement: S5 Data — (ZIP) [file ppat.1012546.s009.zip › Figure S1-4/FigS1/B/B-2/WCL-GFP.tif]

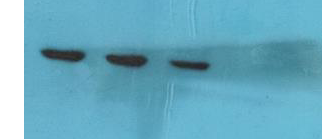

Supplement: S5 Data — (ZIP) [file ppat.1012546.s009.zip › Figure S1-4/FigS1/B/B-2/WCL-Pro-CASP1.tif]

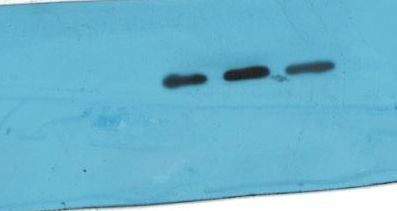

Supplement: S5 Data — (ZIP) [file ppat.1012546.s009.zip › Figure S1-4/FigS1/B/B-2/WCL-Pro-IL-la┬.tif]

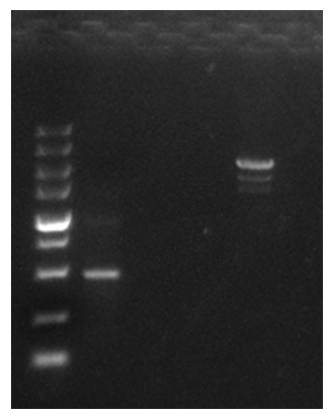

Supplement: S5 Data — (ZIP) [file ppat.1012546.s009.zip › Figure S1-4/FigS2/A/PRV╟├│2UL4.tif]

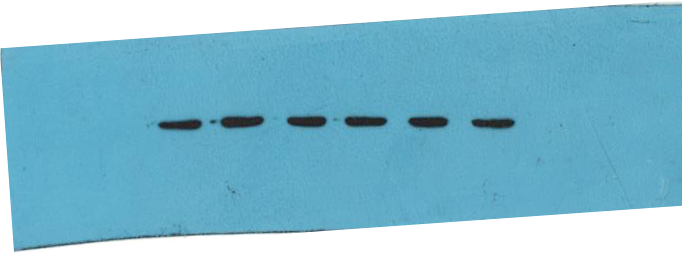

Supplement: S5 Data — (ZIP) [file ppat.1012546.s009.zip › Figure S1-4/FigS2/C/1/Actin.tif]

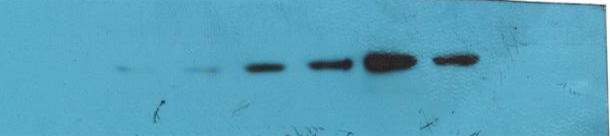

Supplement: S5 Data — (ZIP) [file ppat.1012546.s009.zip › Figure S1-4/FigS2/C/1/EP0.tif]

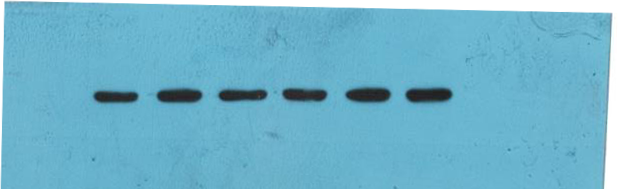

Supplement: S5 Data — (ZIP) [file ppat.1012546.s009.zip › Figure S1-4/FigS2/C/2/Actin.tif]

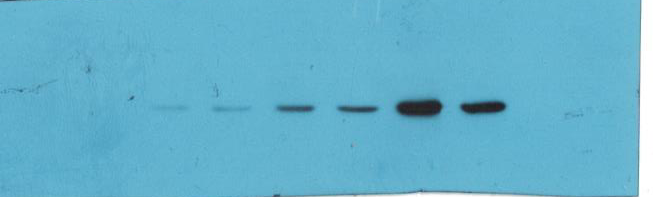

Supplement: S5 Data — (ZIP) [file ppat.1012546.s009.zip › Figure S1-4/FigS2/C/2/EP0.tif]

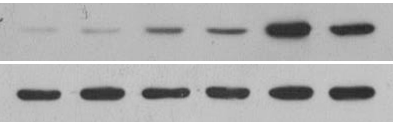

Supplement: S5 Data — (ZIP) [file ppat.1012546.s009.zip › Figure S1-4/FigS2/C/2/╫Θ═╝.tif]

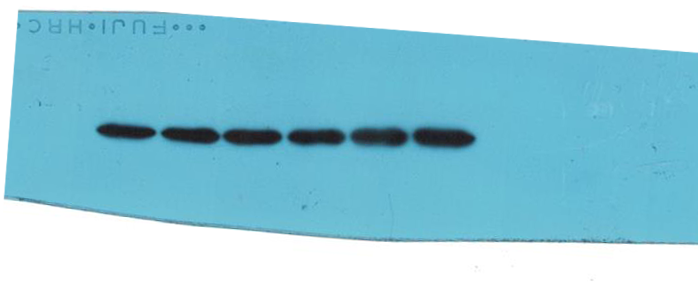

Supplement: S5 Data — (ZIP) [file ppat.1012546.s009.zip › Figure S1-4/FigS2/D/1/Actin.tif]

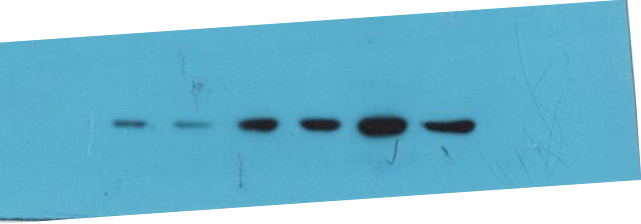

Supplement: S5 Data — (ZIP) [file ppat.1012546.s009.zip › Figure S1-4/FigS2/D/1/EP0.tif]

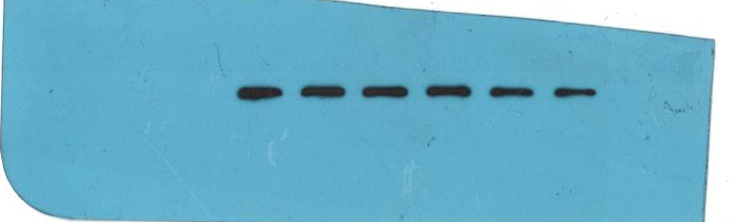

Supplement: S5 Data — (ZIP) [file ppat.1012546.s009.zip › Figure S1-4/FigS2/D/2/Actin.tif]

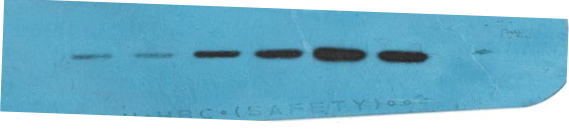

Supplement: S5 Data — (ZIP) [file ppat.1012546.s009.zip › Figure S1-4/FigS2/D/2/EP0.tif]

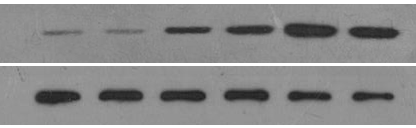

Supplement: S5 Data — (ZIP) [file ppat.1012546.s009.zip › Figure S1-4/FigS2/D/2/╫Θ═╝.tif]

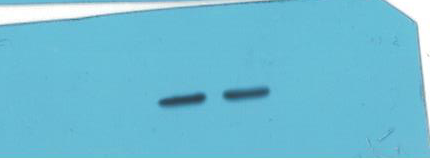

Supplement: S5 Data — (ZIP) [file ppat.1012546.s009.zip › Figure S1-4/FigS2/G/1/Sup-Pro-CASP1-p10.tif]

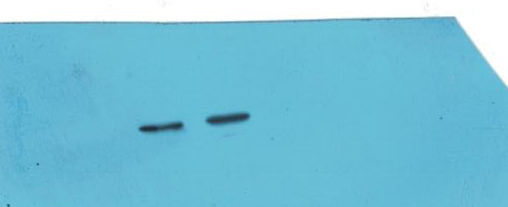

Supplement: S5 Data — (ZIP) [file ppat.1012546.s009.zip › Figure S1-4/FigS2/G/1/Sup-Pro-IL-1a┬ p17.tif]

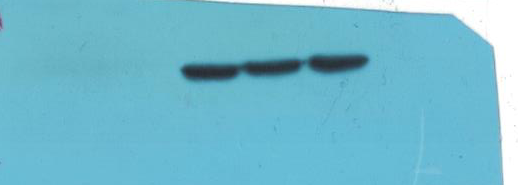

Supplement: S5 Data — (ZIP) [file ppat.1012546.s009.zip › Figure S1-4/FigS2/G/1/WCL-Actin.tif]

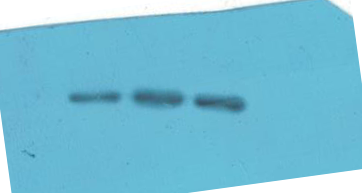

Supplement: S5 Data — (ZIP) [file ppat.1012546.s009.zip › Figure S1-4/FigS2/G/1/WCL-Pro-CASP1.tif]

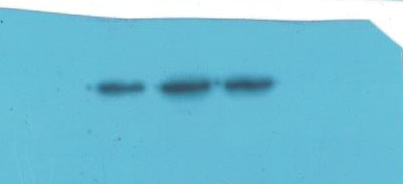

Supplement: S5 Data — (ZIP) [file ppat.1012546.s009.zip › Figure S1-4/FigS2/G/1/WCL-Pro-IL-1a┬.tif]

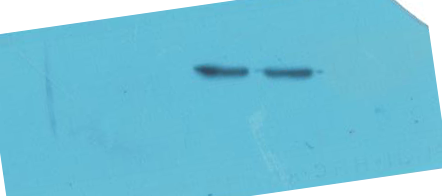

Supplement: S5 Data — (ZIP) [file ppat.1012546.s009.zip › Figure S1-4/FigS2/G/1/WCL-UL4.tif]

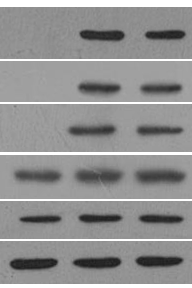

Supplement: S5 Data — (ZIP) [file ppat.1012546.s009.zip › Figure S1-4/FigS2/G/2/3D-1.tif]

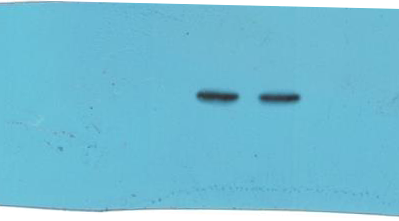

Supplement: S5 Data — (ZIP) [file ppat.1012546.s009.zip › Figure S1-4/FigS2/G/2/Sup-Pro-CASP1-p10.tif]

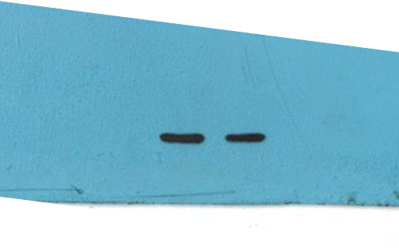

Supplement: S5 Data — (ZIP) [file ppat.1012546.s009.zip › Figure S1-4/FigS2/G/2/Sup-Pro-IL-1a┬-p17.tif]

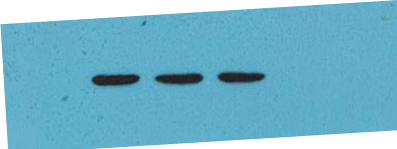

Supplement: S5 Data — (ZIP) [file ppat.1012546.s009.zip › Figure S1-4/FigS2/G/2/WCL-Actin.tif]

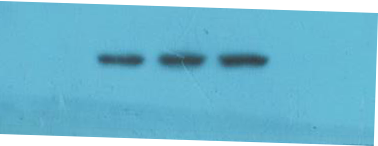

Supplement: S5 Data — (ZIP) [file ppat.1012546.s009.zip › Figure S1-4/FigS2/G/2/WCL-Pro-CASP1.tif]

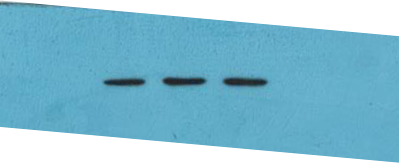

Supplement: S5 Data — (ZIP) [file ppat.1012546.s009.zip › Figure S1-4/FigS2/G/2/WCL-Pro-IL-1a┬.tif]

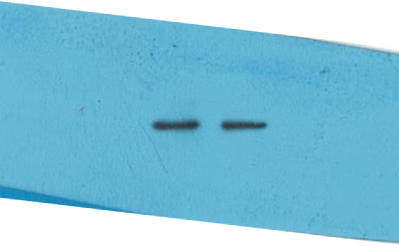

Supplement: S5 Data — (ZIP) [file ppat.1012546.s009.zip › Figure S1-4/FigS2/G/2/WCL-UL4.tif]

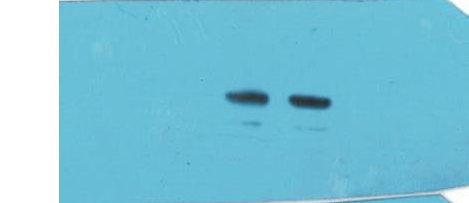

Supplement: S5 Data — (ZIP) [file ppat.1012546.s009.zip › Figure S1-4/FigS2/H/1/Sup-Pro-CASP1-P10.tif]

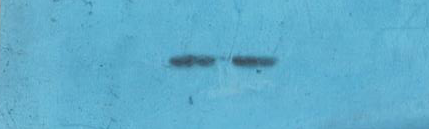

Supplement: S5 Data — (ZIP) [file ppat.1012546.s009.zip › Figure S1-4/FigS2/H/1/Sup-Pro-IL-1a┬-P17.tif]

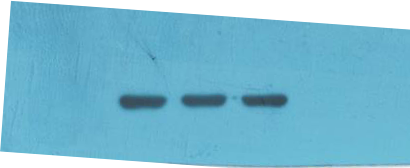

Supplement: S5 Data — (ZIP) [file ppat.1012546.s009.zip › Figure S1-4/FigS2/H/1/WCL-Actin.tif]

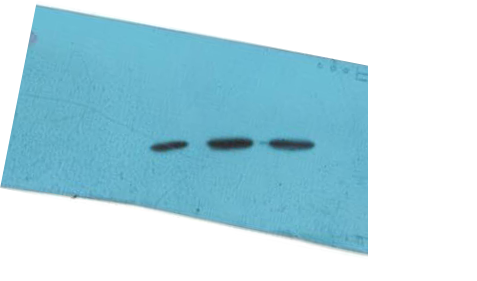

Supplement: S5 Data — (ZIP) [file ppat.1012546.s009.zip › Figure S1-4/FigS2/H/1/WCL-Pro-CASP1.tif]

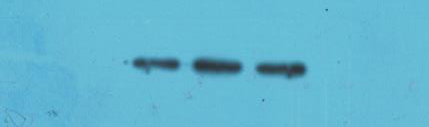

Supplement: S5 Data — (ZIP) [file ppat.1012546.s009.zip › Figure S1-4/FigS2/H/1/WCL-Pro-IL-1a┬.tif]

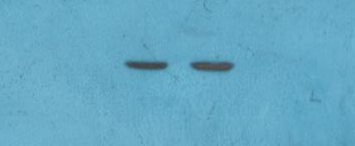

Supplement: S5 Data — (ZIP) [file ppat.1012546.s009.zip › Figure S1-4/FigS2/H/1/WCL-UL4.tif]

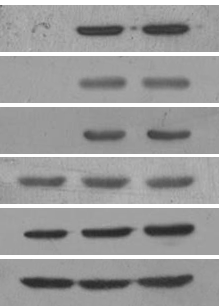

Supplement: S5 Data — (ZIP) [file ppat.1012546.s009.zip › Figure S1-4/FigS2/H/2/bmdm.tif]

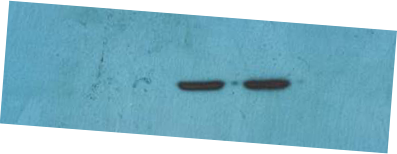

Supplement: S5 Data — (ZIP) [file ppat.1012546.s009.zip › Figure S1-4/FigS2/H/2/Sup-P10.tif]

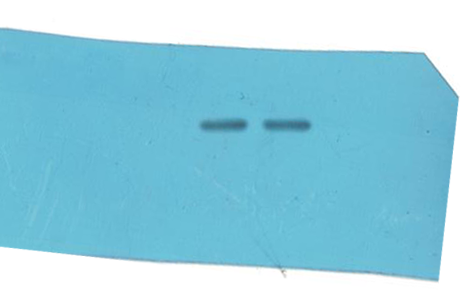

Supplement: S5 Data — (ZIP) [file ppat.1012546.s009.zip › Figure S1-4/FigS2/H/2/Sup-P17.tif]

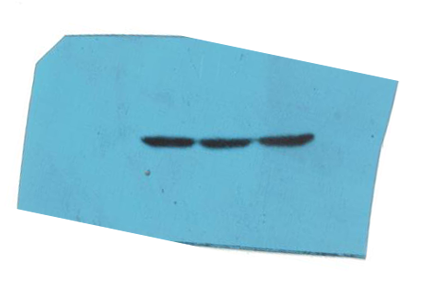

Supplement: S5 Data — (ZIP) [file ppat.1012546.s009.zip › Figure S1-4/FigS2/H/2/WCL-Actin.tif]

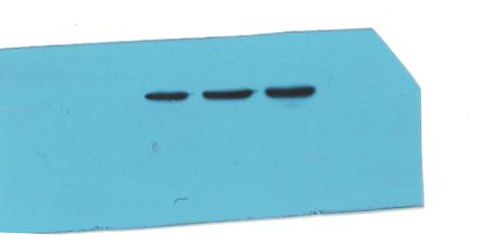

Supplement: S5 Data — (ZIP) [file ppat.1012546.s009.zip › Figure S1-4/FigS2/H/2/WCL-Pro-CASP1.tif]

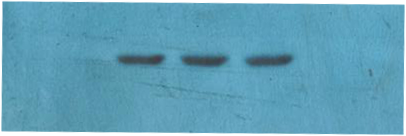

Supplement: S5 Data — (ZIP) [file ppat.1012546.s009.zip › Figure S1-4/FigS2/H/2/WCL-Pro-IL-1a┬.tif]

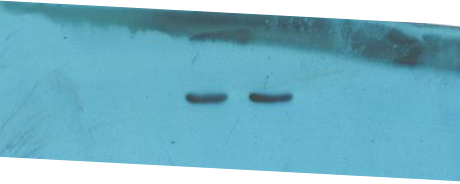

Supplement: S5 Data — (ZIP) [file ppat.1012546.s009.zip › Figure S1-4/FigS2/H/2/WCL-UL4.tif]

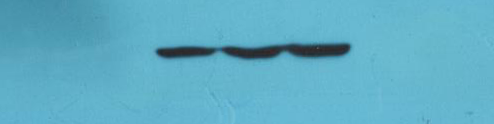

Supplement: S5 Data — (ZIP) [file ppat.1012546.s009.zip › Figure S1-4/FigS3/E/1/Actin.tif]

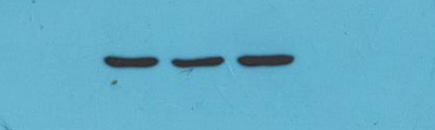

Supplement: S5 Data — (ZIP) [file ppat.1012546.s009.zip › Figure S1-4/FigS3/E/1/ASC.tif]

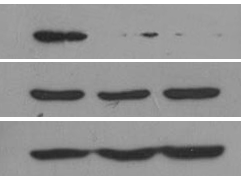

Supplement: S5 Data — (ZIP) [file ppat.1012546.s009.zip › Figure S1-4/FigS3/E/1/Merge.tif]

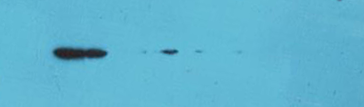

Supplement: S5 Data — (ZIP) [file ppat.1012546.s009.zip › Figure S1-4/FigS3/E/1/p-ASC.tif]

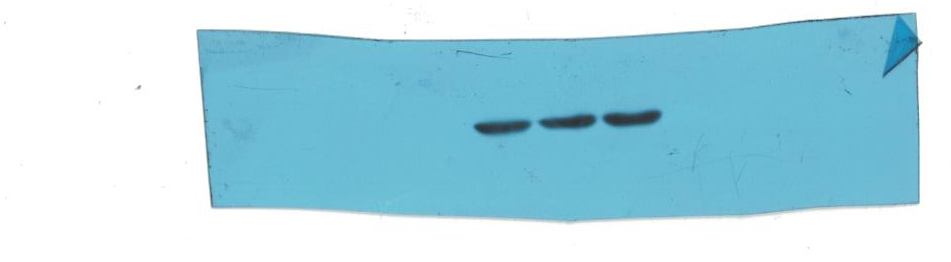

Supplement: S5 Data — (ZIP) [file ppat.1012546.s009.zip › Figure S1-4/FigS3/E/2/Actin.tif]

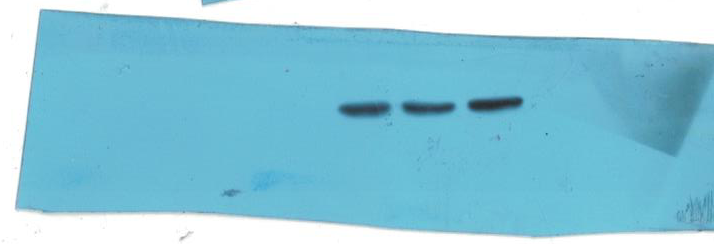

Supplement: S5 Data — (ZIP) [file ppat.1012546.s009.zip › Figure S1-4/FigS3/E/2/ASC.tif]

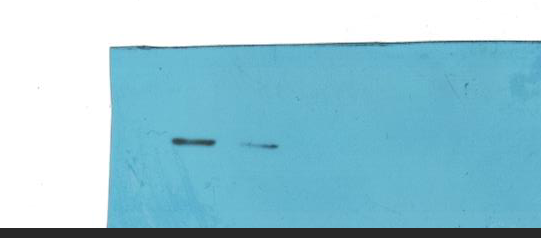

Supplement: S5 Data — (ZIP) [file ppat.1012546.s009.zip › Figure S1-4/FigS3/E/2/p-ASC.tif]

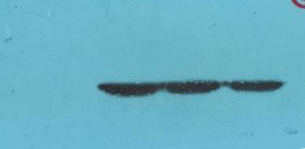

Supplement: S5 Data — (ZIP) [file ppat.1012546.s009.zip › Figure S1-4/FigS3/F/1/Actin.tif]

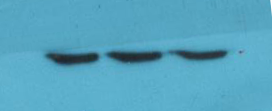

Supplement: S5 Data — (ZIP) [file ppat.1012546.s009.zip › Figure S1-4/FigS3/F/1/ASC.tif]

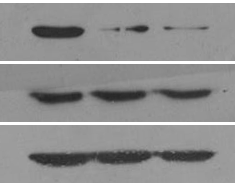

Supplement: S5 Data — (ZIP) [file ppat.1012546.s009.zip › Figure S1-4/FigS3/F/1/merge.tif]

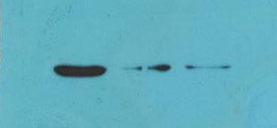

Supplement: S5 Data — (ZIP) [file ppat.1012546.s009.zip › Figure S1-4/FigS3/F/1/p-ASC.tif]

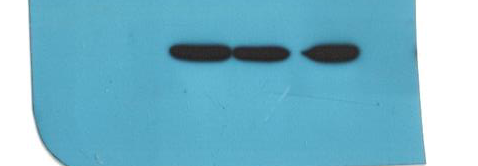

Supplement: S5 Data — (ZIP) [file ppat.1012546.s009.zip › Figure S1-4/FigS3/F/2/Actin.tif]

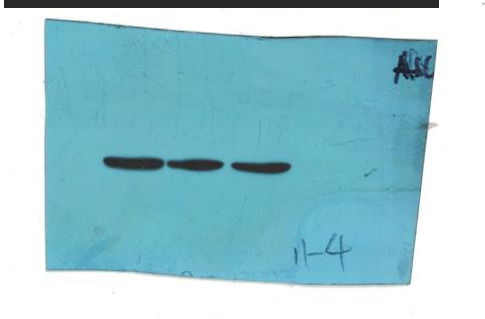

Supplement: S5 Data — (ZIP) [file ppat.1012546.s009.zip › Figure S1-4/FigS3/F/2/ASC.tif]

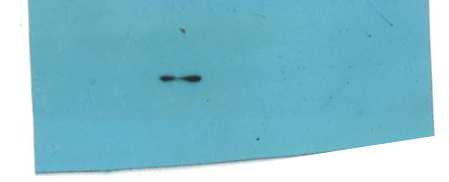

Supplement: S5 Data — (ZIP) [file ppat.1012546.s009.zip › Figure S1-4/FigS3/F/2/p-ASC.tif]

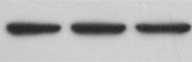

Supplement: S5 Data — (ZIP) [file ppat.1012546.s009.zip › Figure S1-4/FigS4/G/G-1/Brain-Actin.tif]

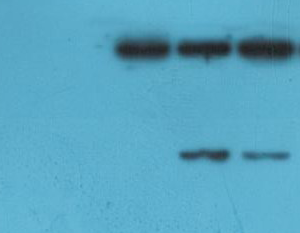

Supplement: S5 Data — (ZIP) [file ppat.1012546.s009.zip › Figure S1-4/FigS4/G/G-1/Brain-GSDMD.tif]

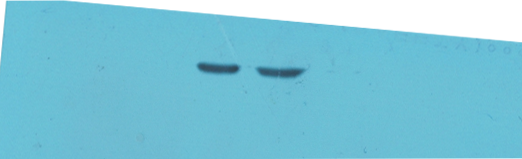

Supplement: S5 Data — (ZIP) [file ppat.1012546.s009.zip › Figure S1-4/FigS4/G/G-1/Brain-UL4-N.tif]

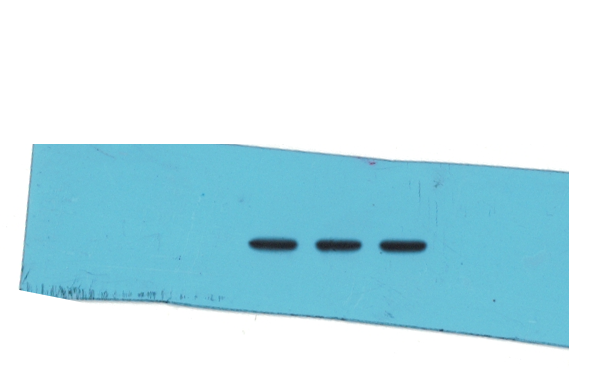

Supplement: S5 Data — (ZIP) [file ppat.1012546.s009.zip › Figure S1-4/FigS4/G/G-1/Lung-Actin.tif]

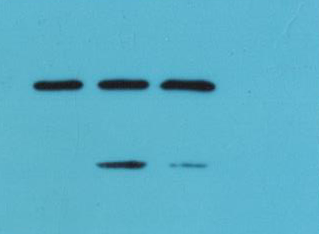

Supplement: S5 Data — (ZIP) [file ppat.1012546.s009.zip › Figure S1-4/FigS4/G/G-1/Lung-GSDMD.tif]

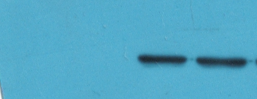

Supplement: S5 Data — (ZIP) [file ppat.1012546.s009.zip › Figure S1-4/FigS4/G/G-1/Lung-UL4.tif]

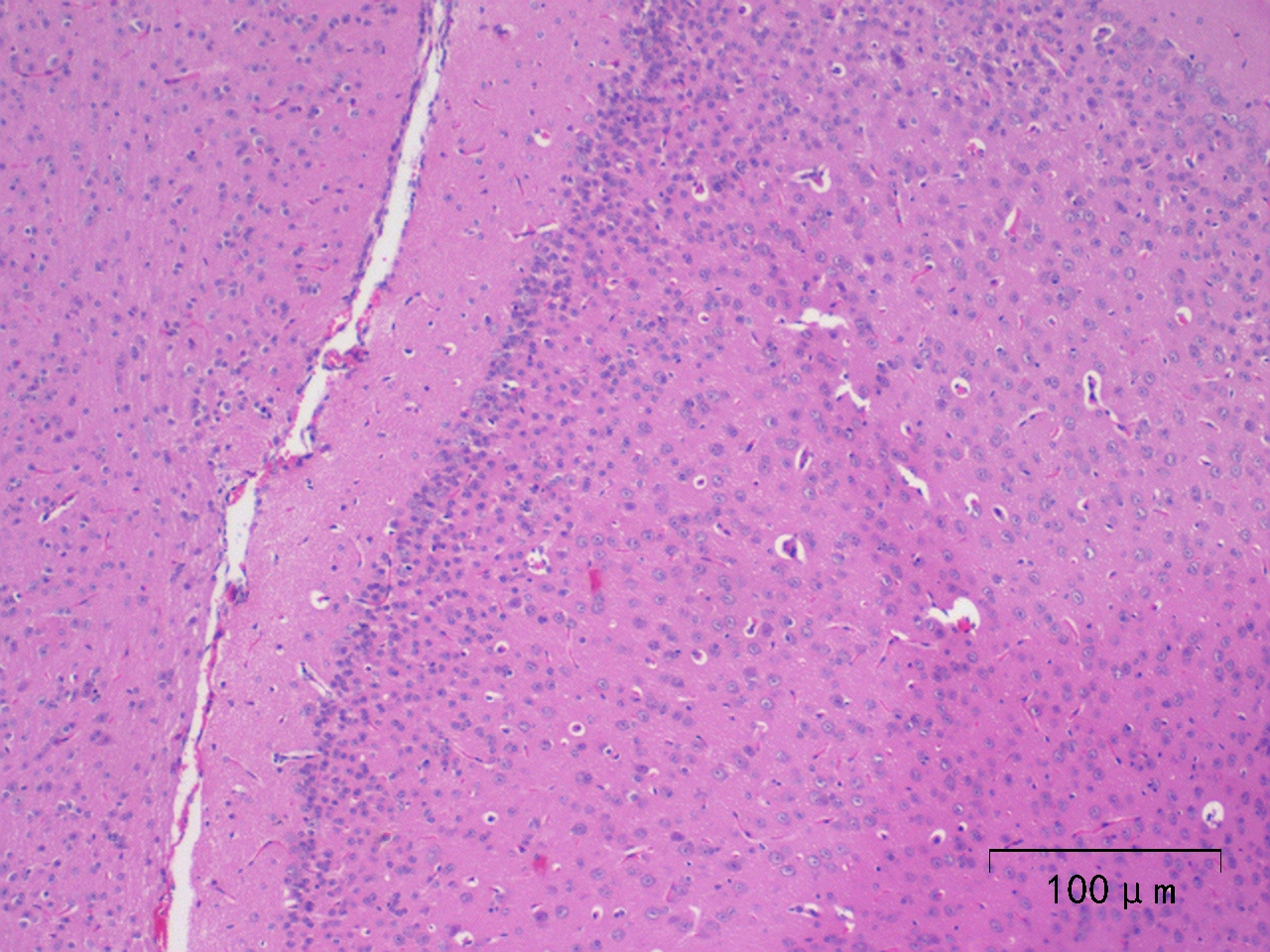

Supplement: S5 Data — (ZIP) [file ppat.1012546.s009.zip › Figure S1-4/FigS4/J/Mock-Brain/57.jpg]

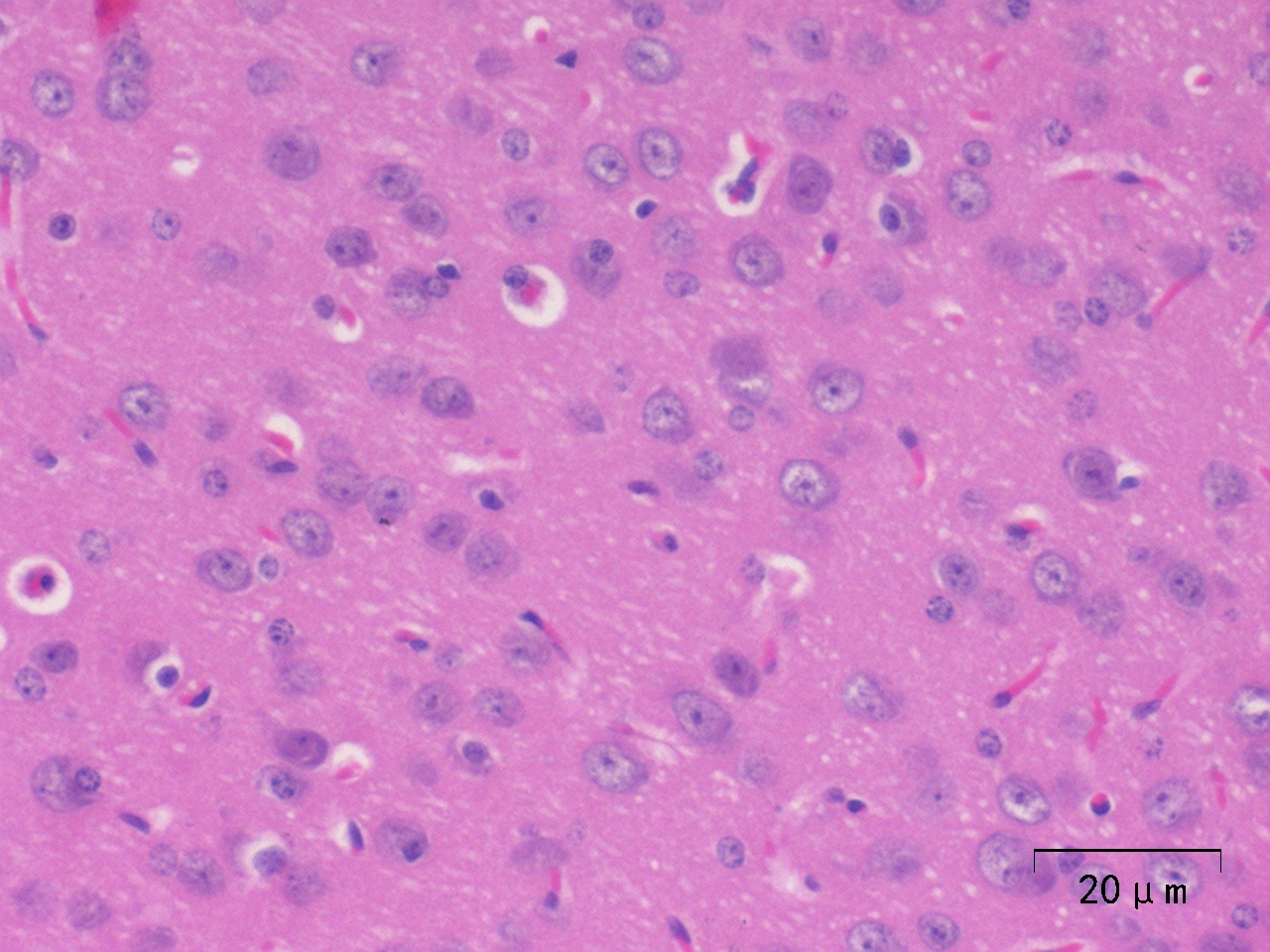

Supplement: S5 Data — (ZIP) [file ppat.1012546.s009.zip › Figure S1-4/FigS4/J/Mock-Brain/58.jpg]

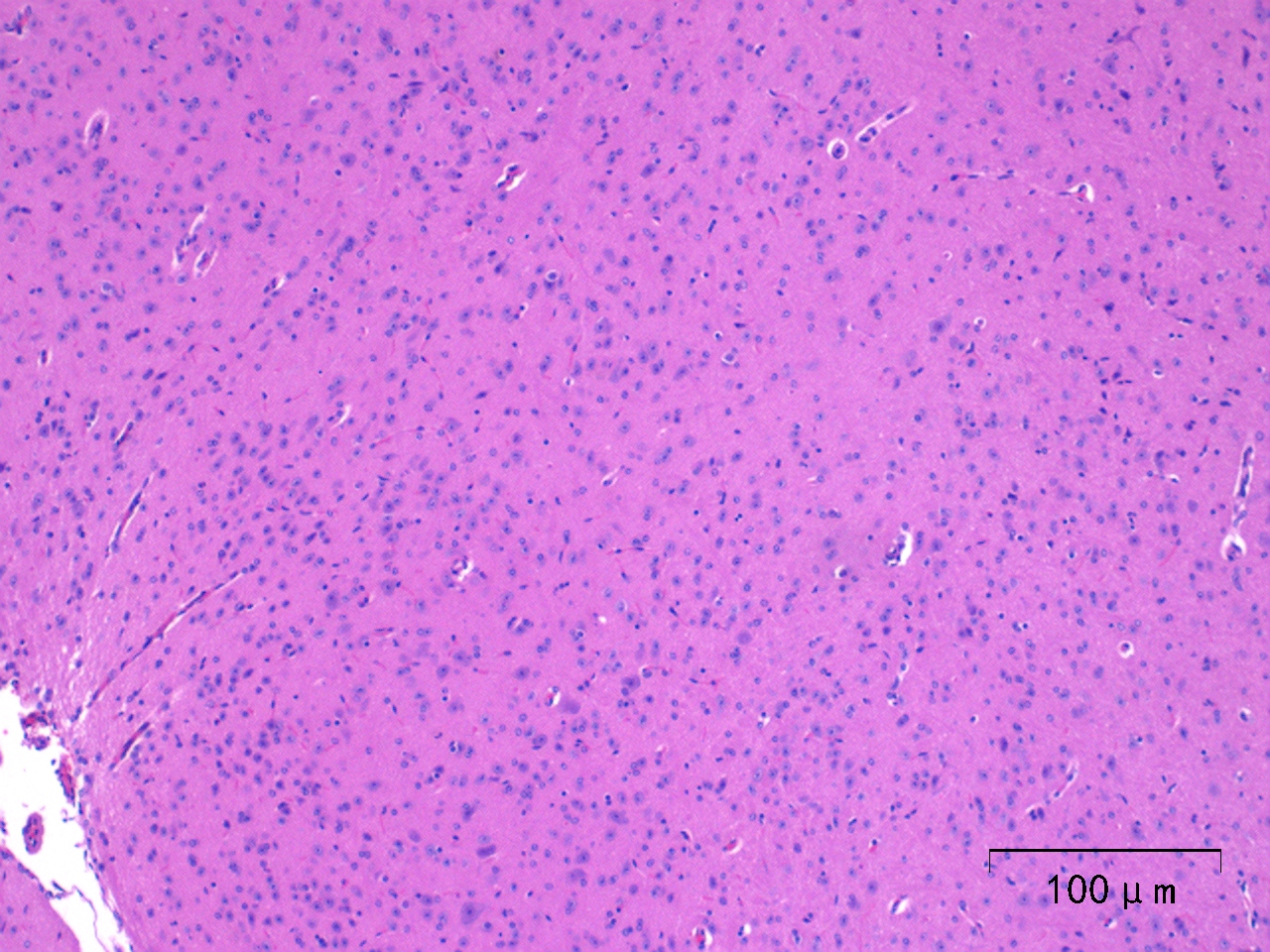

Supplement: S5 Data — (ZIP) [file ppat.1012546.s009.zip › Figure S1-4/FigS4/J/Mock-Brain/59.jpg]

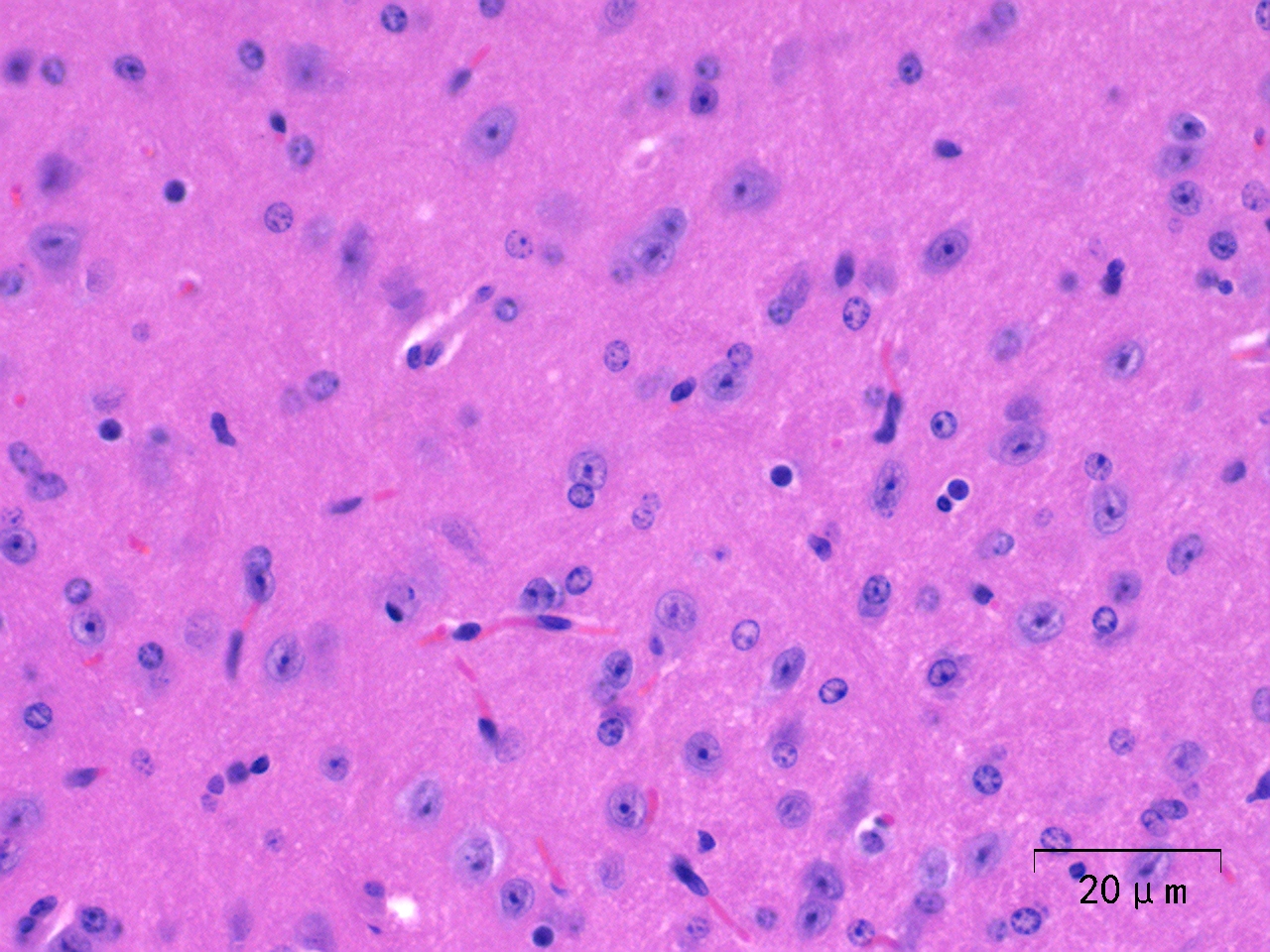

Supplement: S5 Data — (ZIP) [file ppat.1012546.s009.zip › Figure S1-4/FigS4/J/Mock-Brain/60.jpg]

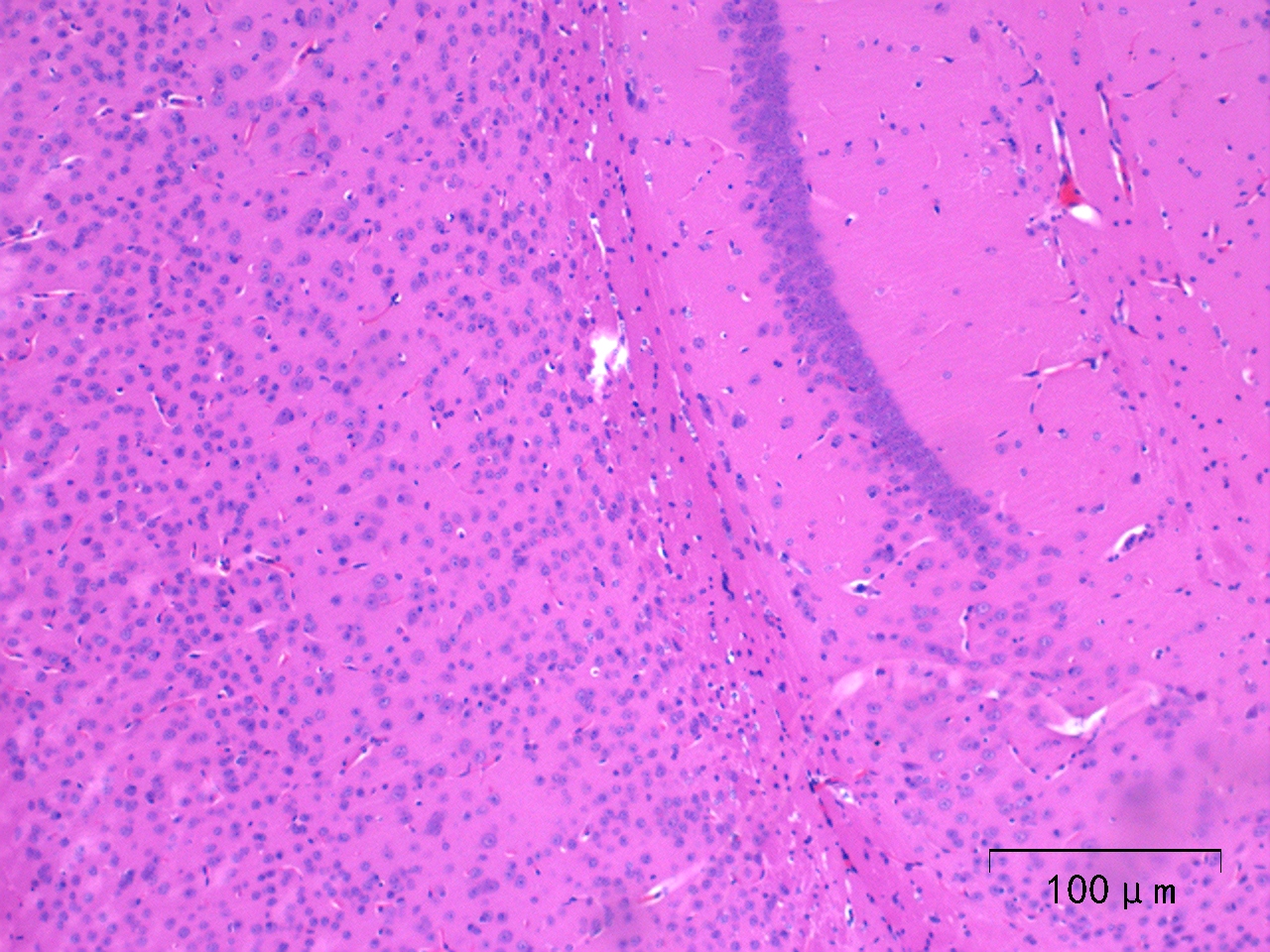

Supplement: S5 Data — (ZIP) [file ppat.1012546.s009.zip › Figure S1-4/FigS4/J/Mock-Brain/61.jpg]

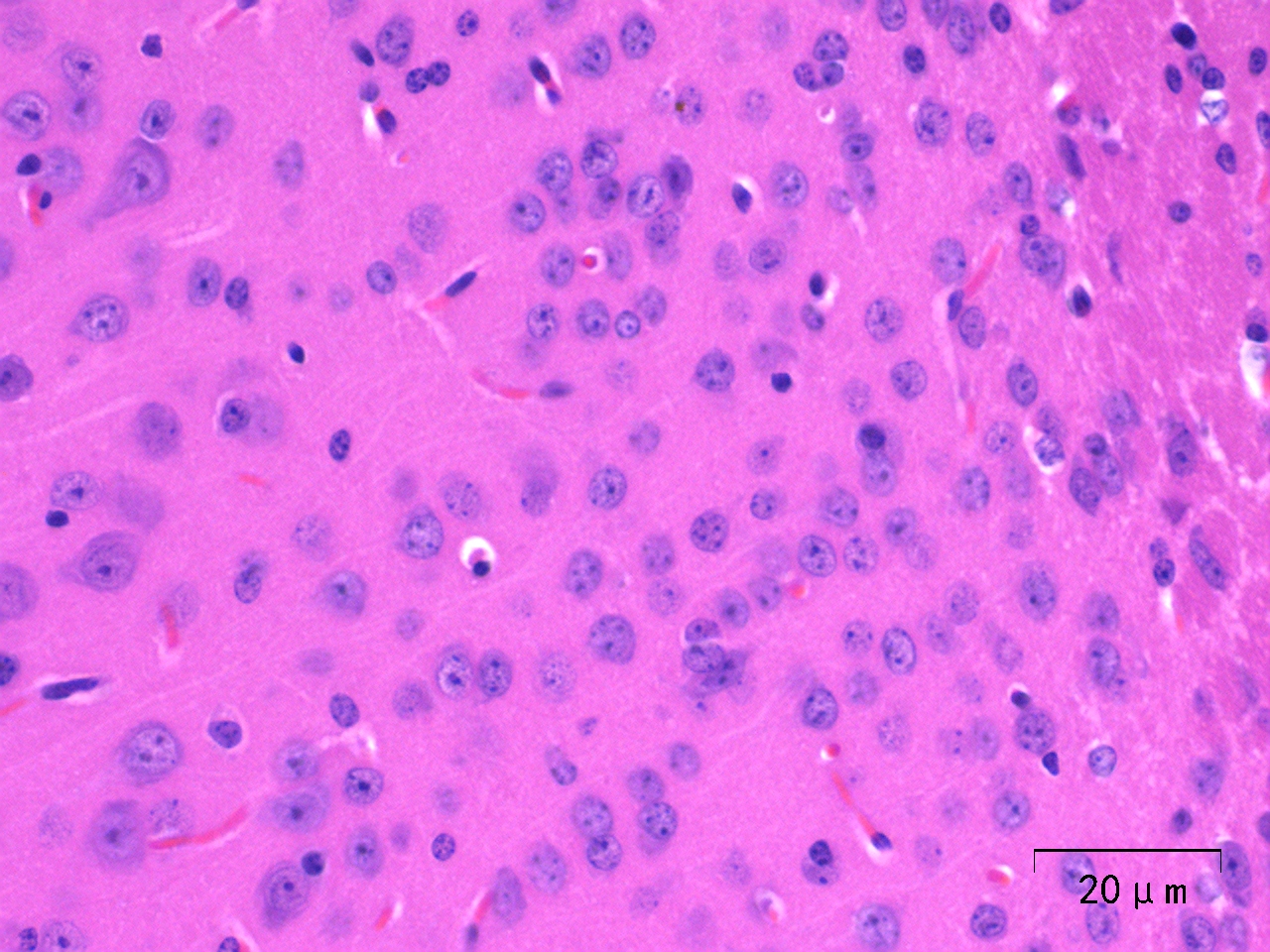

Supplement: S5 Data — (ZIP) [file ppat.1012546.s009.zip › Figure S1-4/FigS4/J/Mock-Brain/62.jpg]

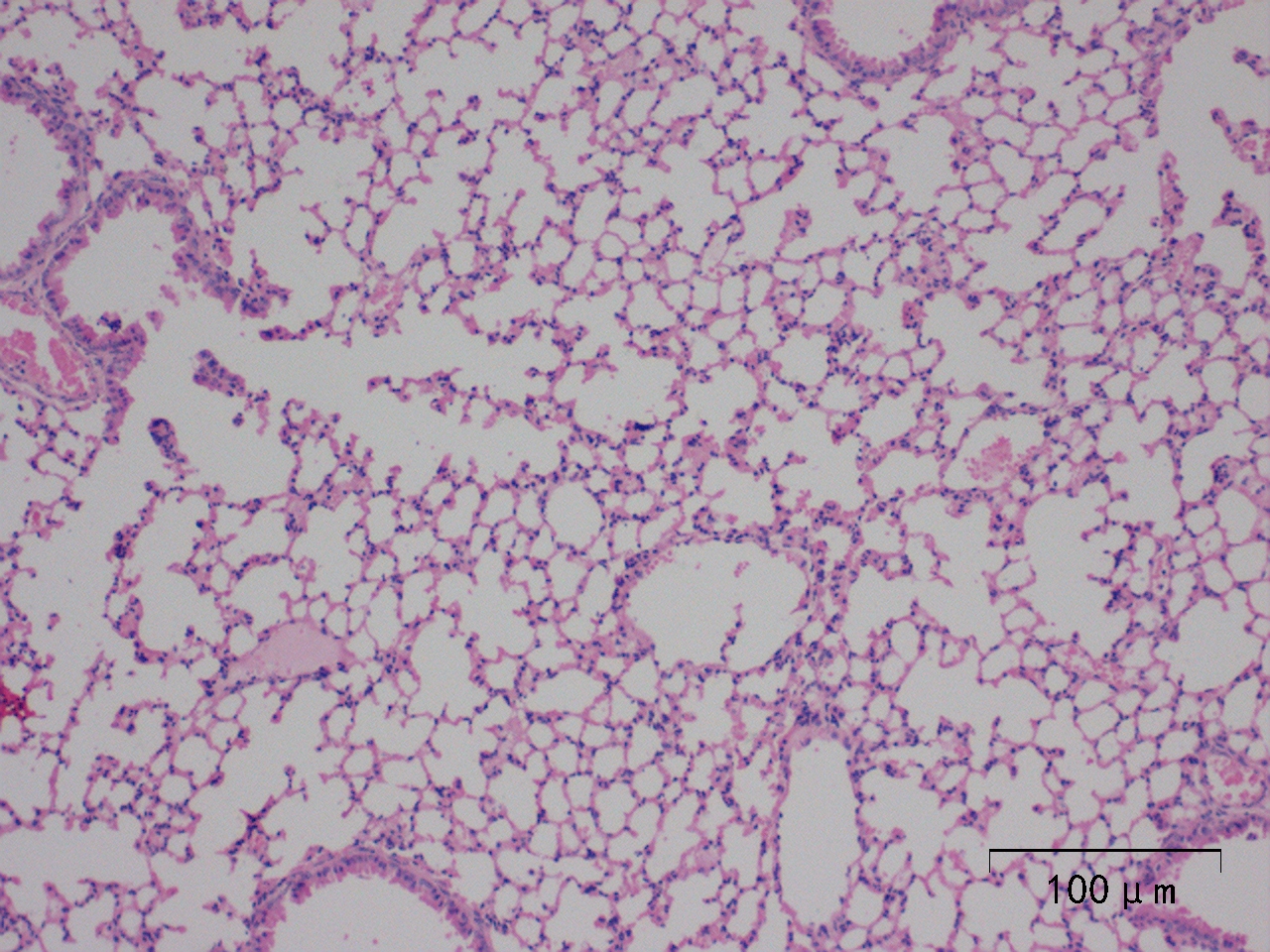

Supplement: S5 Data — (ZIP) [file ppat.1012546.s009.zip › Figure S1-4/FigS4/J/Mock-Lung/39.jpg]

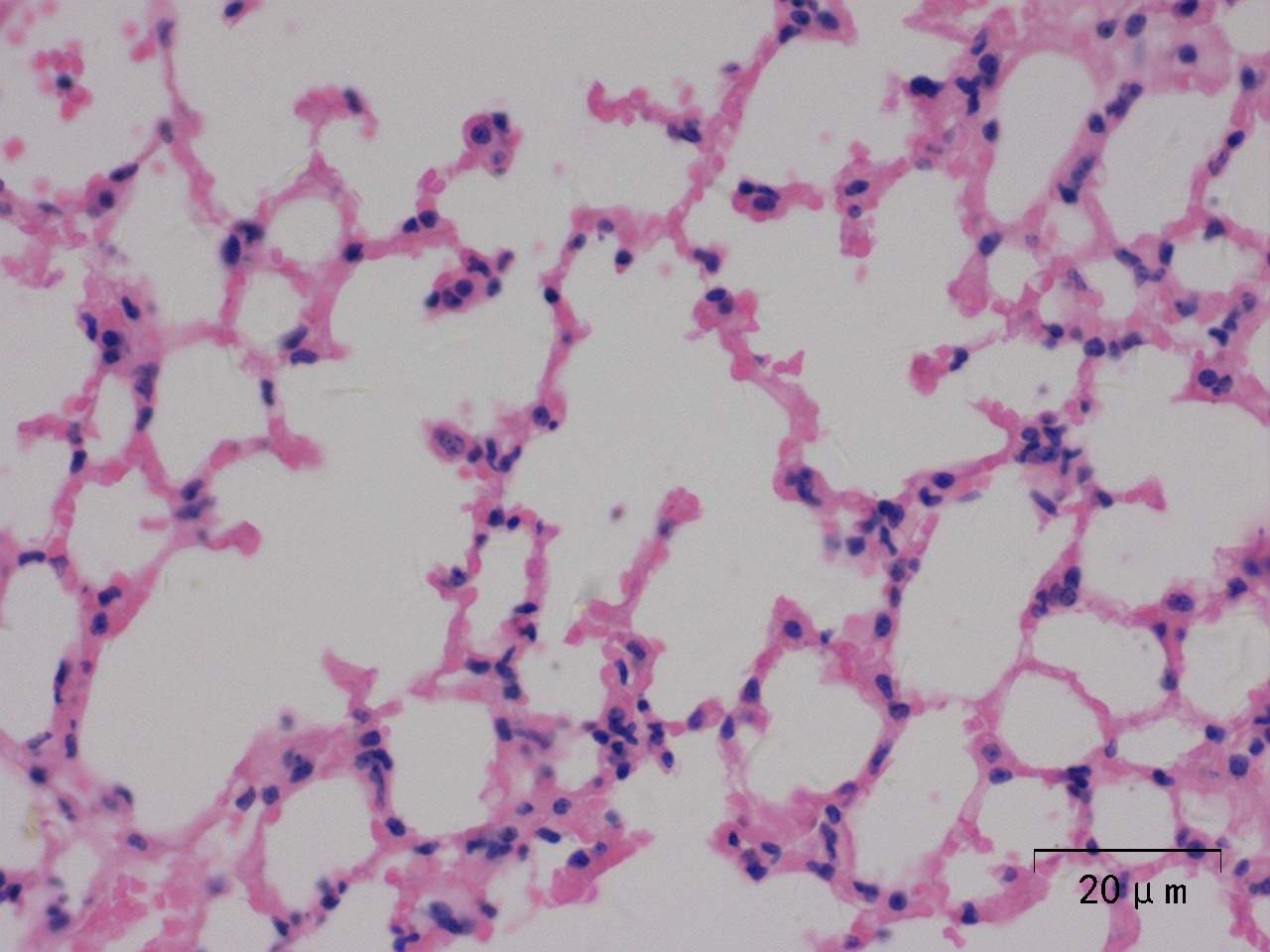

Supplement: S5 Data — (ZIP) [file ppat.1012546.s009.zip › Figure S1-4/FigS4/J/Mock-Lung/40.jpg]

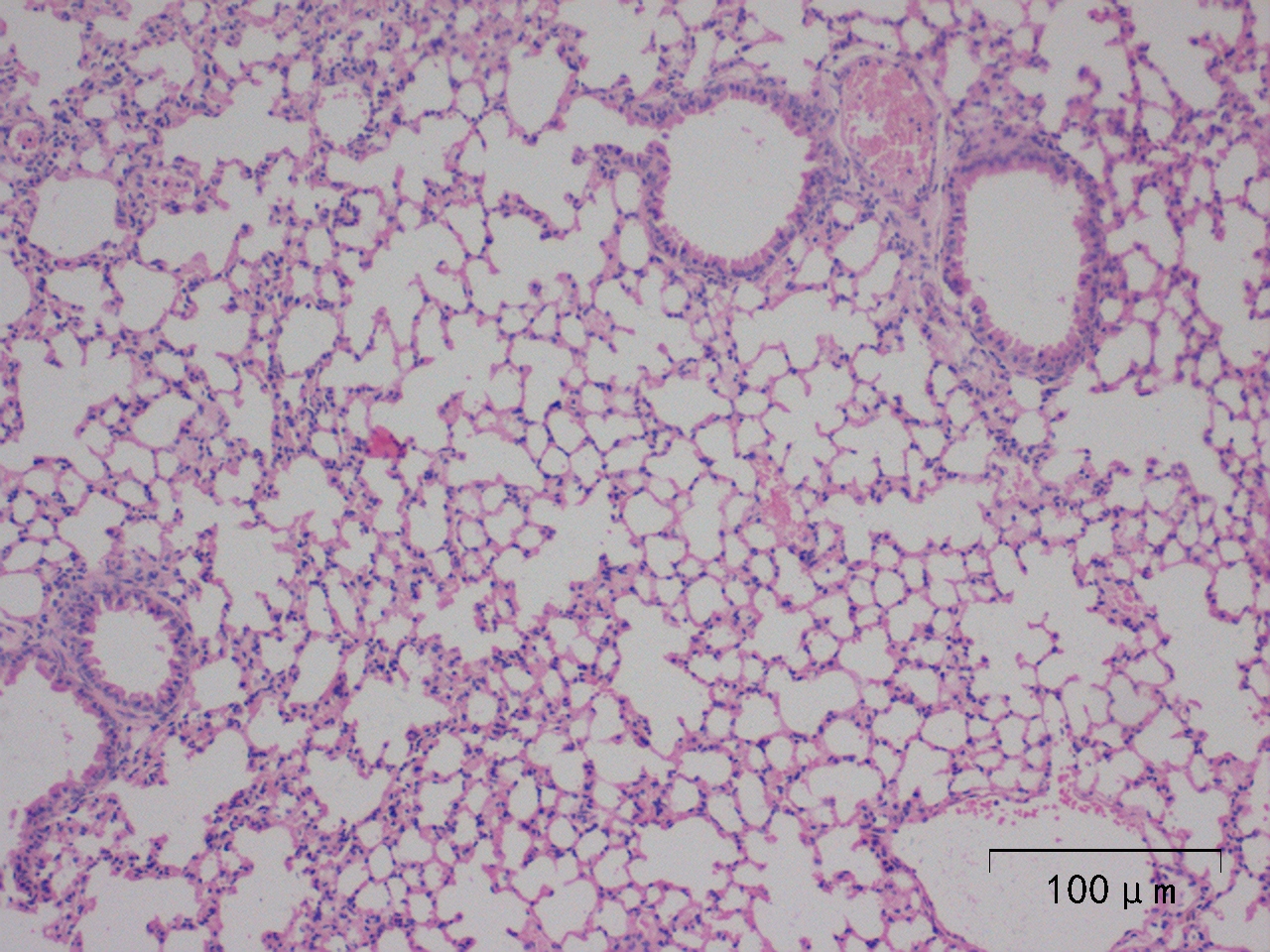

Supplement: S5 Data — (ZIP) [file ppat.1012546.s009.zip › Figure S1-4/FigS4/J/Mock-Lung/41.jpg]

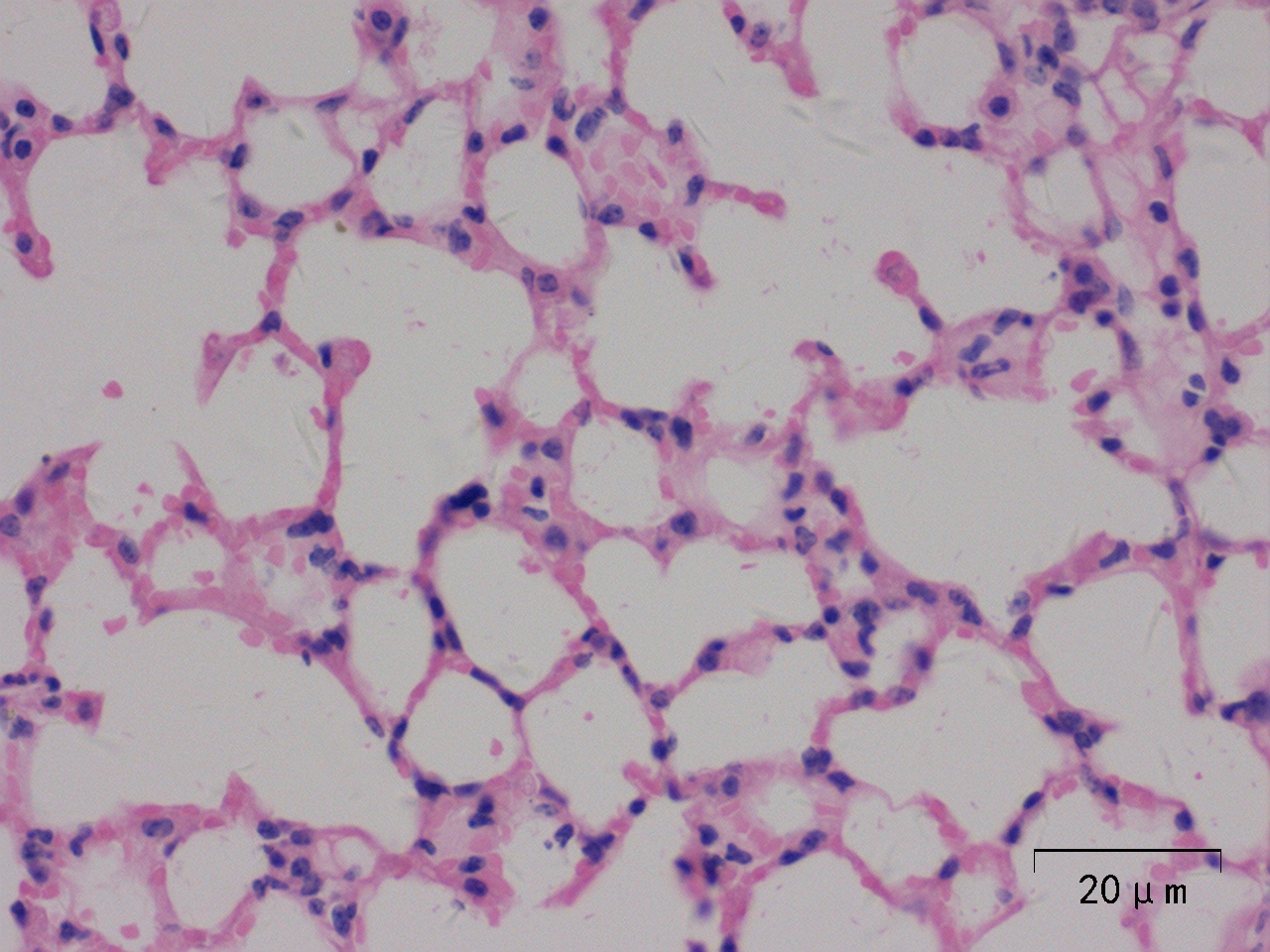

Supplement: S5 Data — (ZIP) [file ppat.1012546.s009.zip › Figure S1-4/FigS4/J/Mock-Lung/42.jpg]

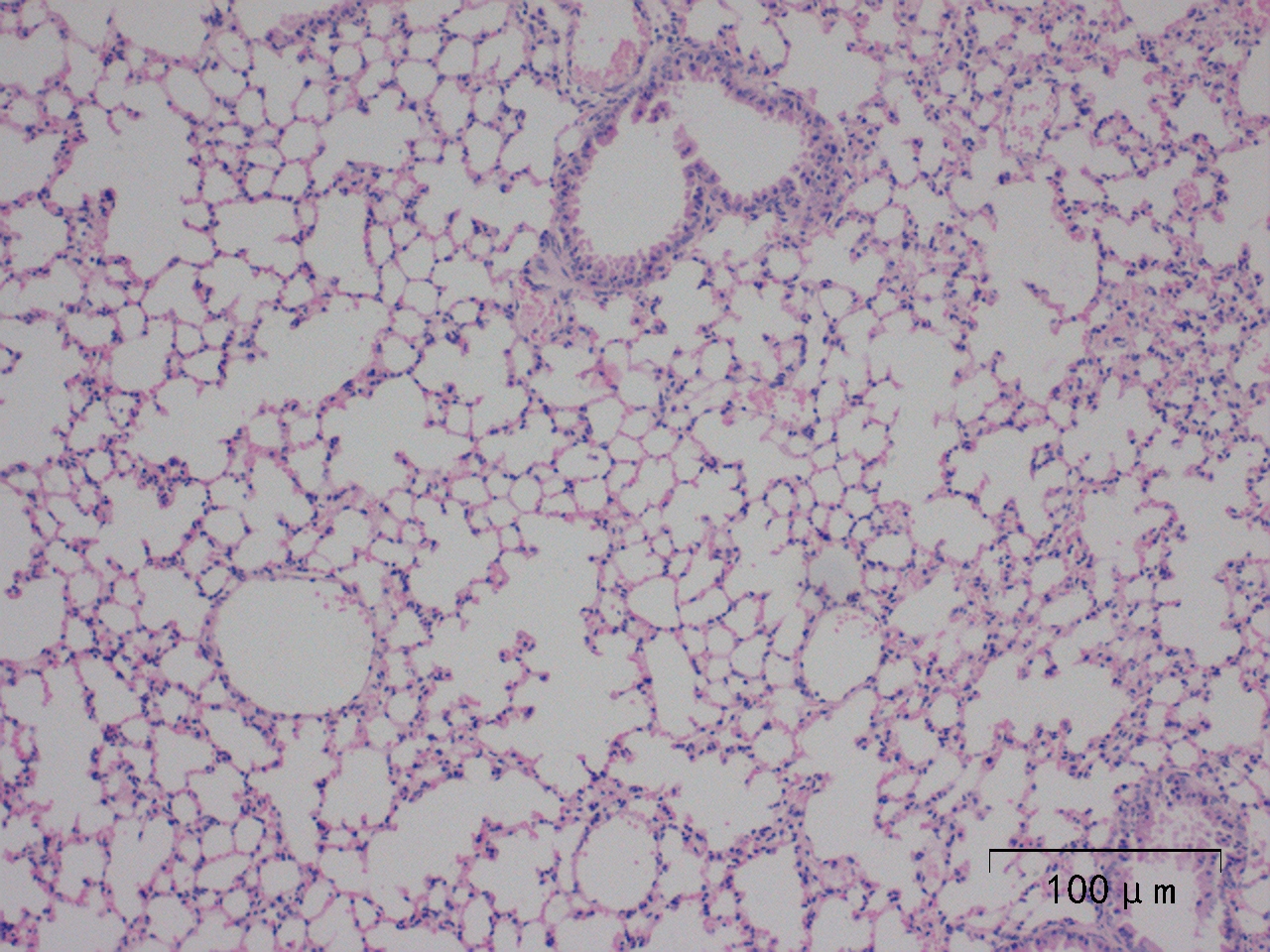

Supplement: S5 Data — (ZIP) [file ppat.1012546.s009.zip › Figure S1-4/FigS4/J/Mock-Lung/43.jpg]

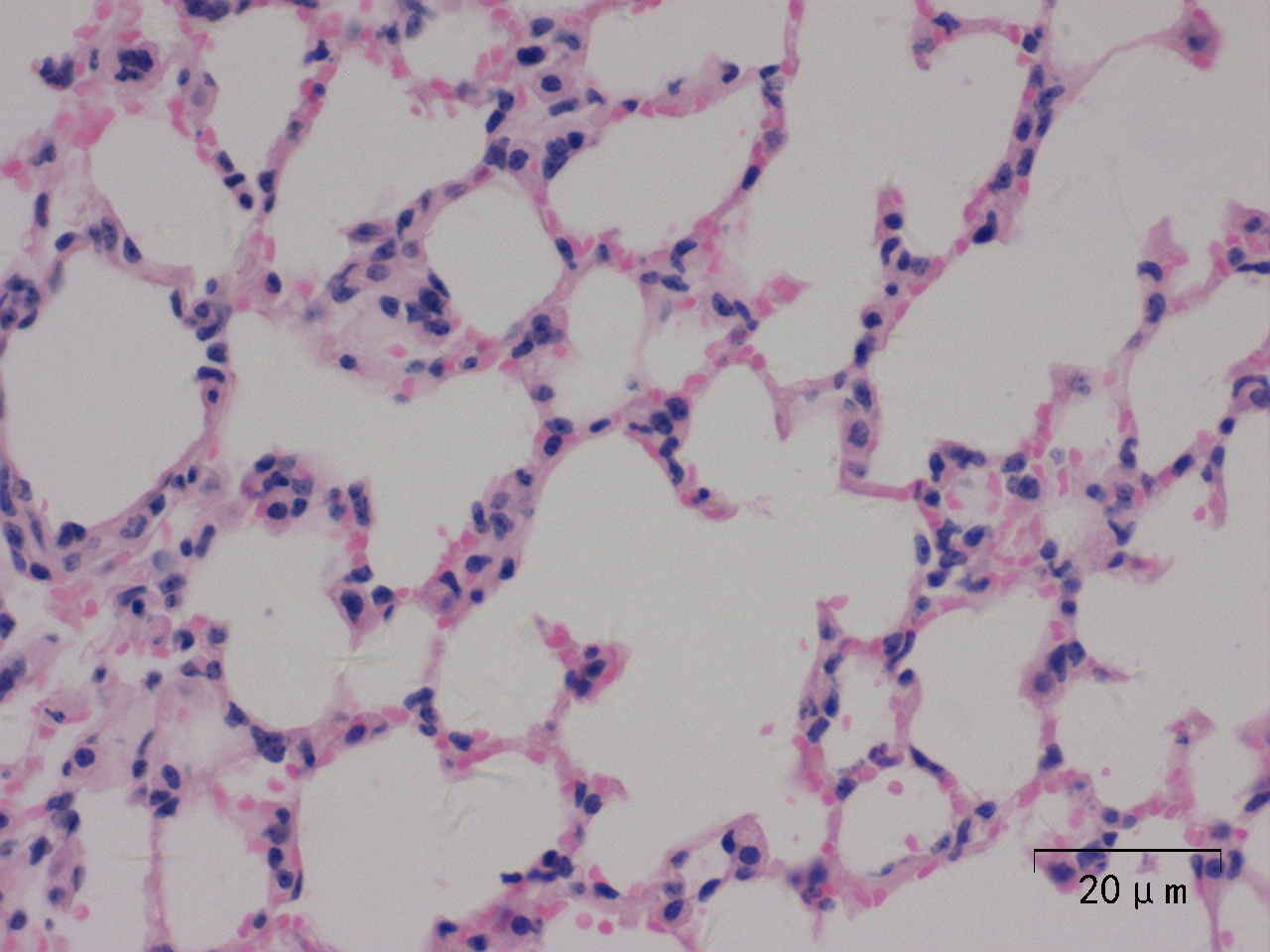

Supplement: S5 Data — (ZIP) [file ppat.1012546.s009.zip › Figure S1-4/FigS4/J/Mock-Lung/44.jpg]

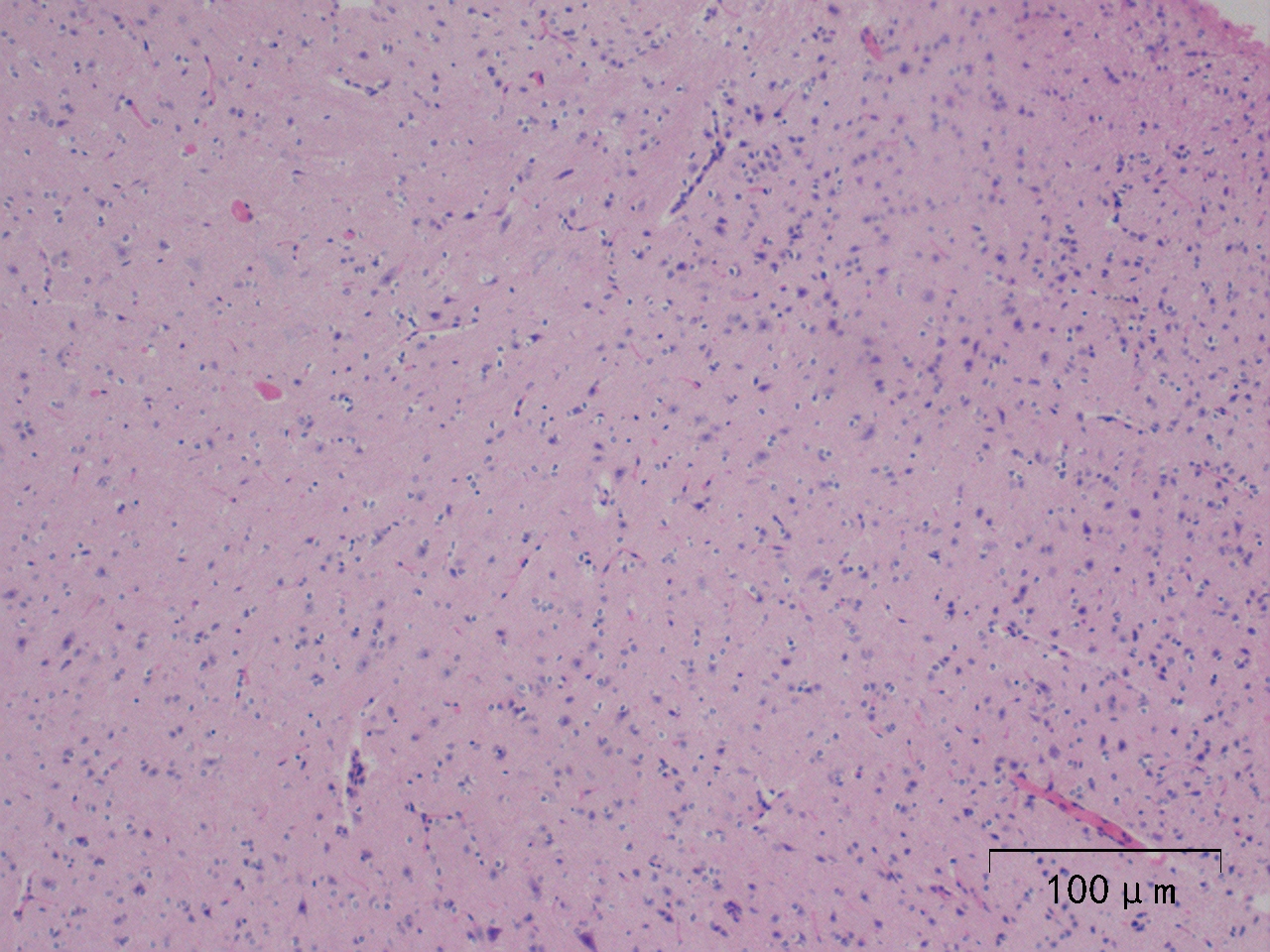

Supplement: S5 Data — (ZIP) [file ppat.1012546.s009.zip › Figure S1-4/FigS4/J/PRV-UL4mut-Brain/64.jpg]

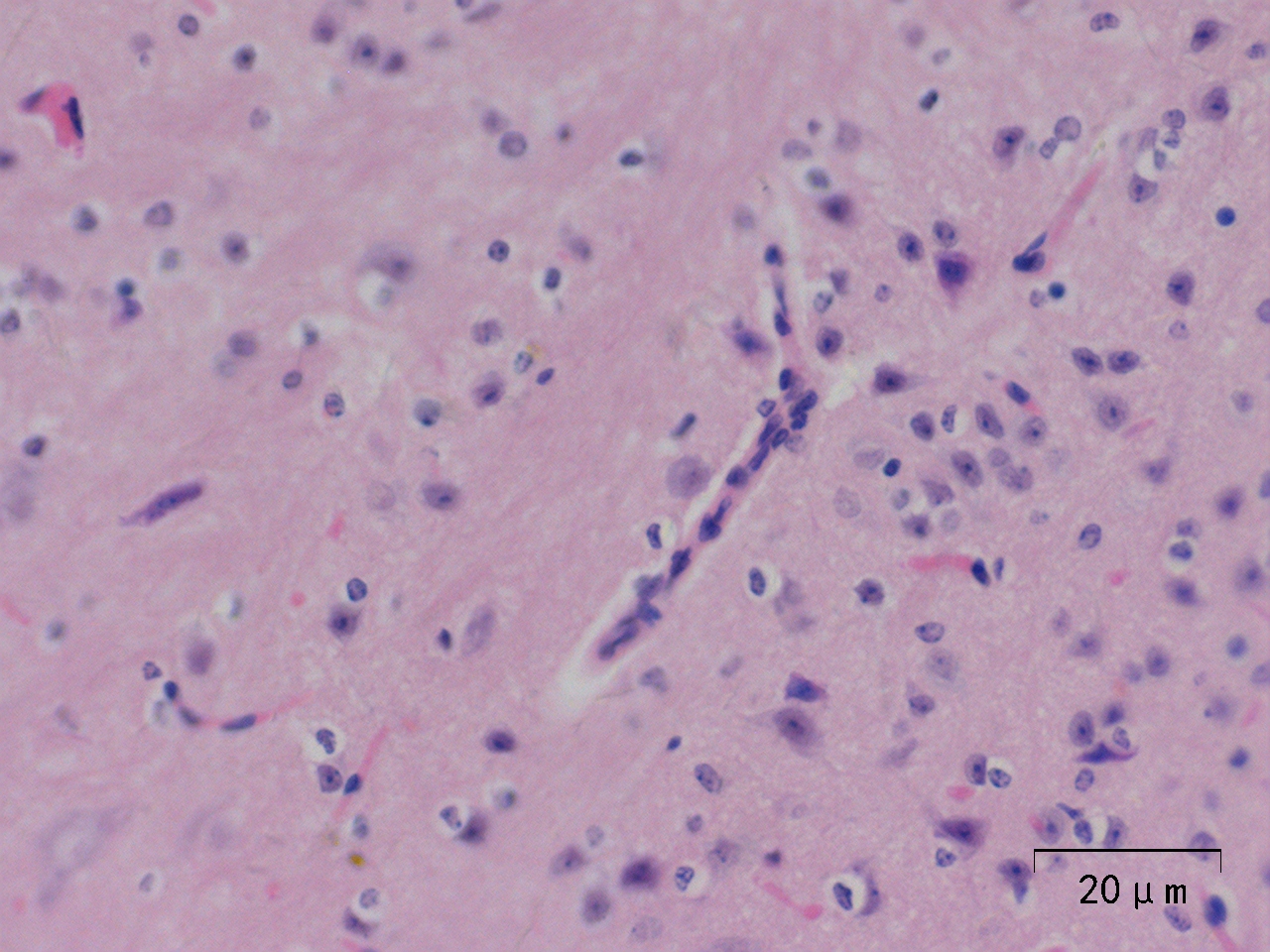

Supplement: S5 Data — (ZIP) [file ppat.1012546.s009.zip › Figure S1-4/FigS4/J/PRV-UL4mut-Brain/65.jpg]

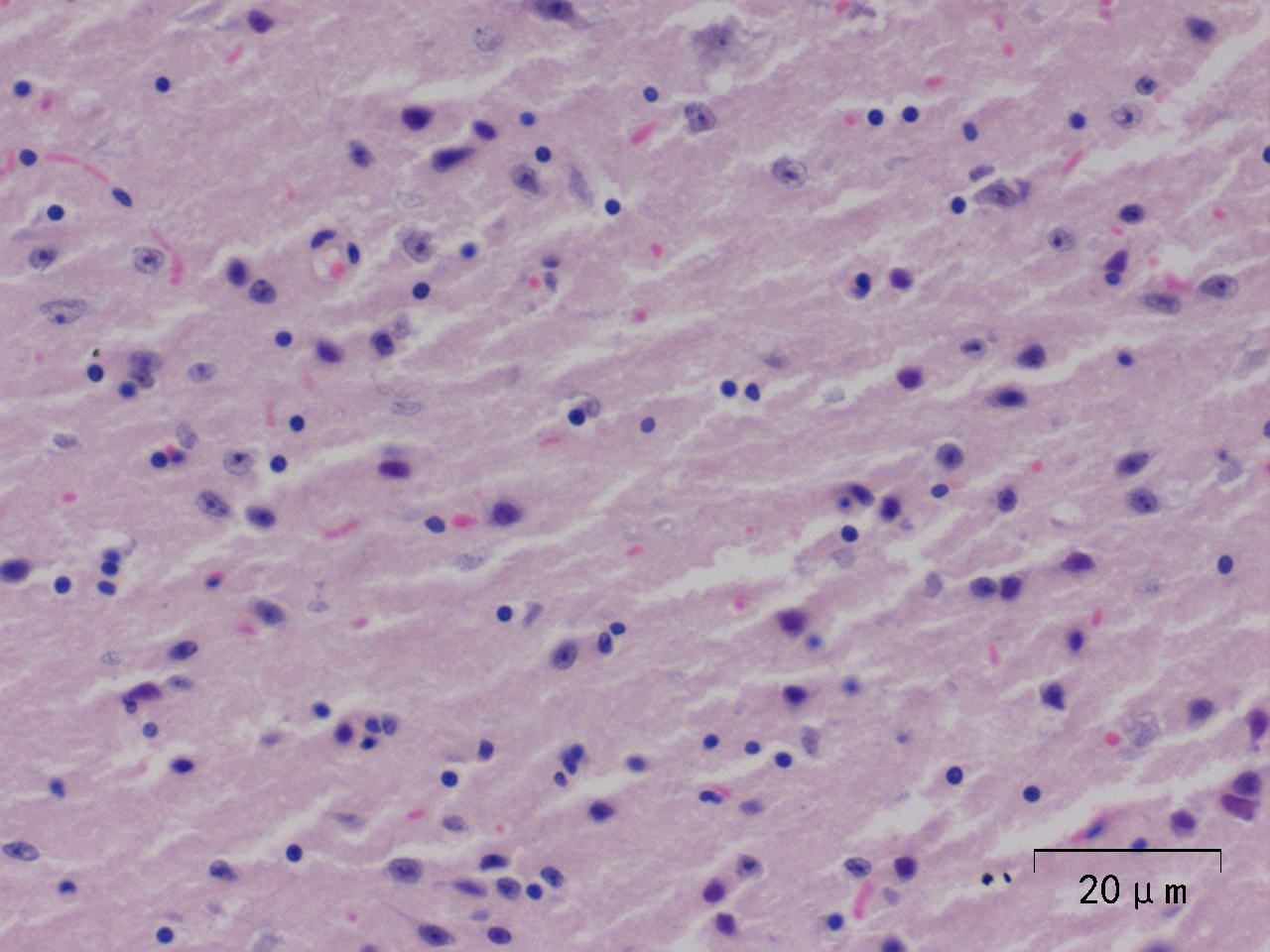

Supplement: S5 Data — (ZIP) [file ppat.1012546.s009.zip › Figure S1-4/FigS4/J/PRV-UL4mut-Brain/66.jpg]

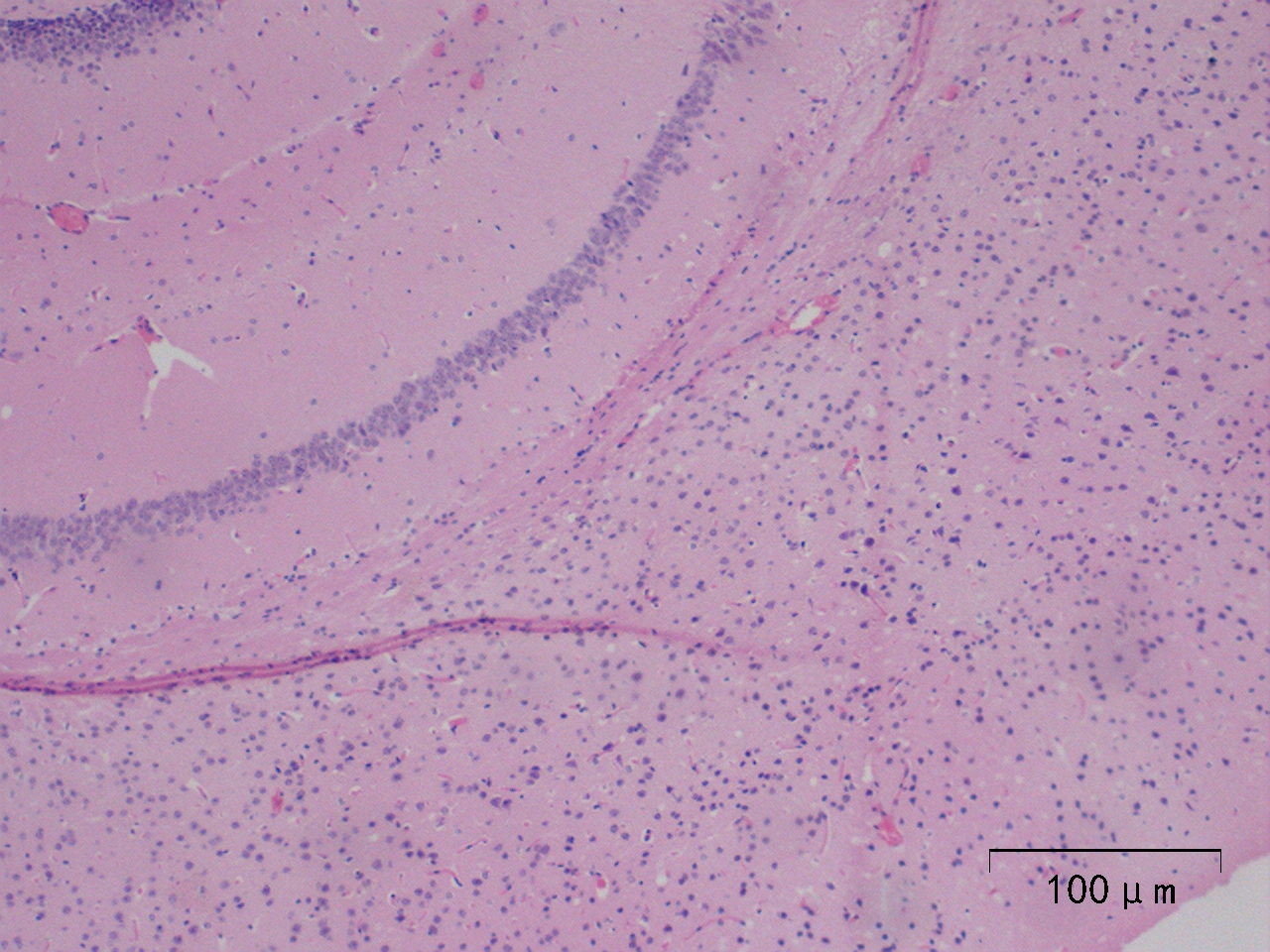

Supplement: S5 Data — (ZIP) [file ppat.1012546.s009.zip › Figure S1-4/FigS4/J/PRV-UL4mut-Brain/67.jpg]

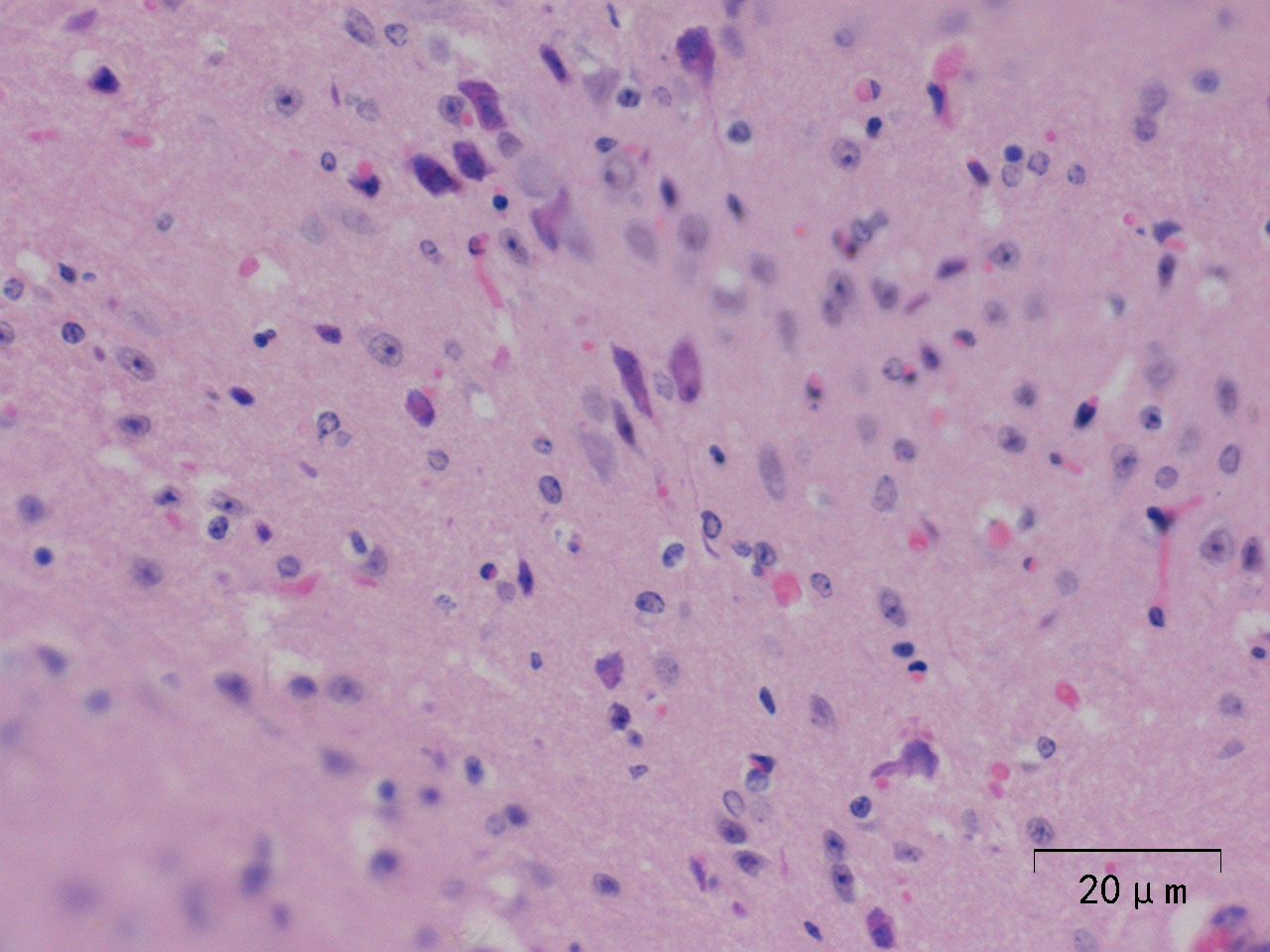

Supplement: S5 Data — (ZIP) [file ppat.1012546.s009.zip › Figure S1-4/FigS4/J/PRV-UL4mut-Brain/68.jpg]

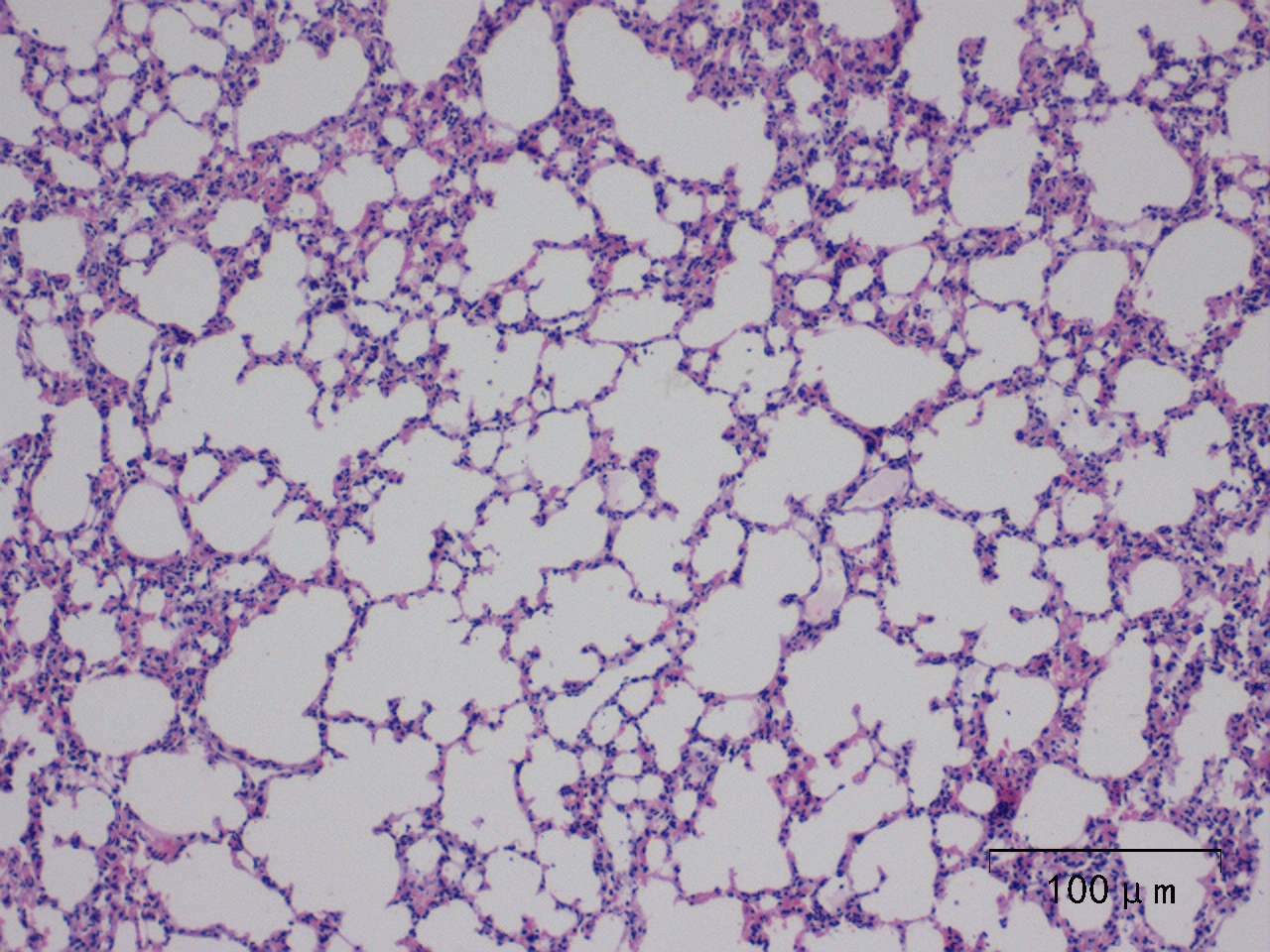

Supplement: S5 Data — (ZIP) [file ppat.1012546.s009.zip › Figure S1-4/FigS4/J/PRV-UL4mut-Lung/45.jpg]

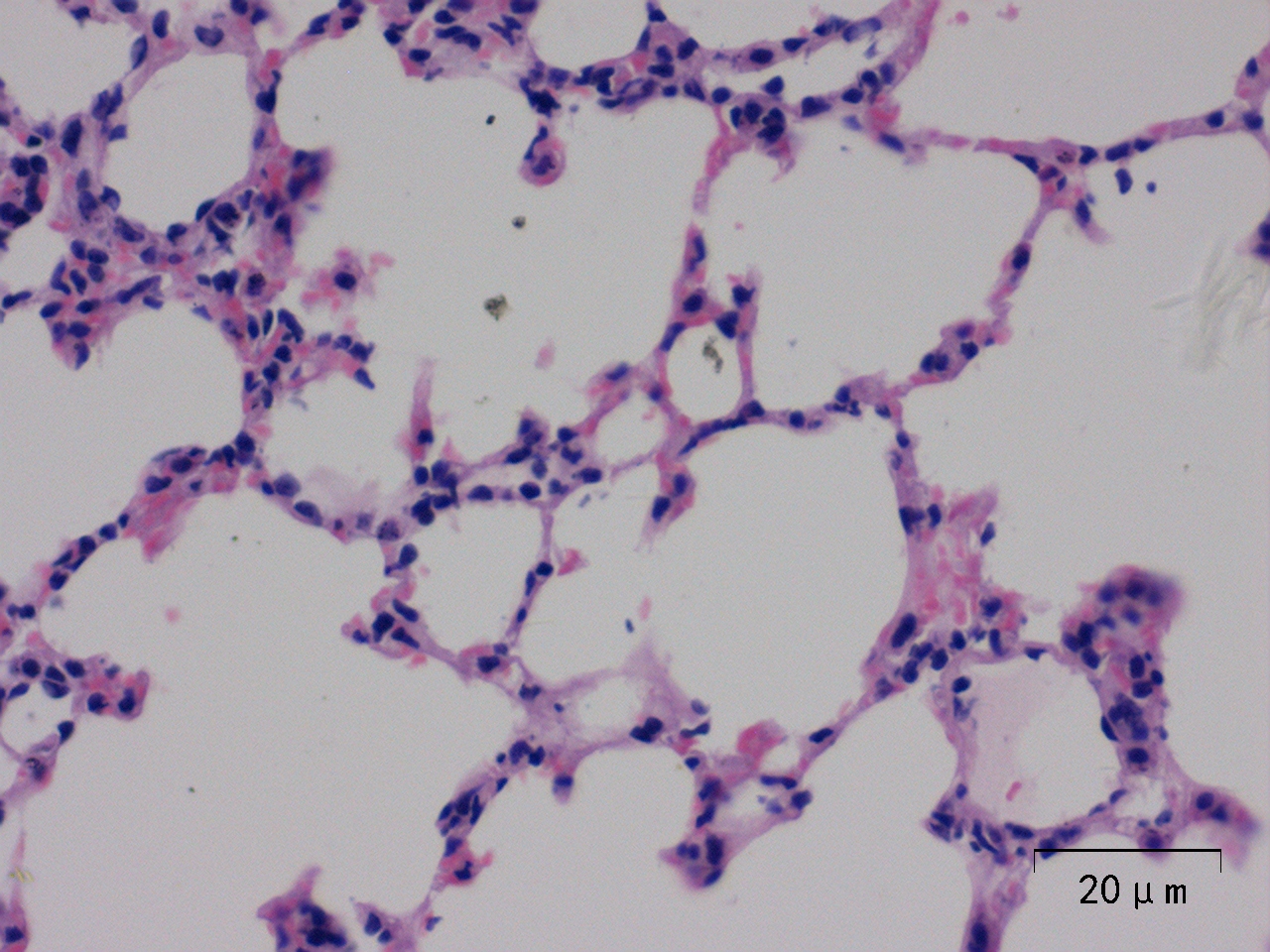

Supplement: S5 Data — (ZIP) [file ppat.1012546.s009.zip › Figure S1-4/FigS4/J/PRV-UL4mut-Lung/46.jpg]

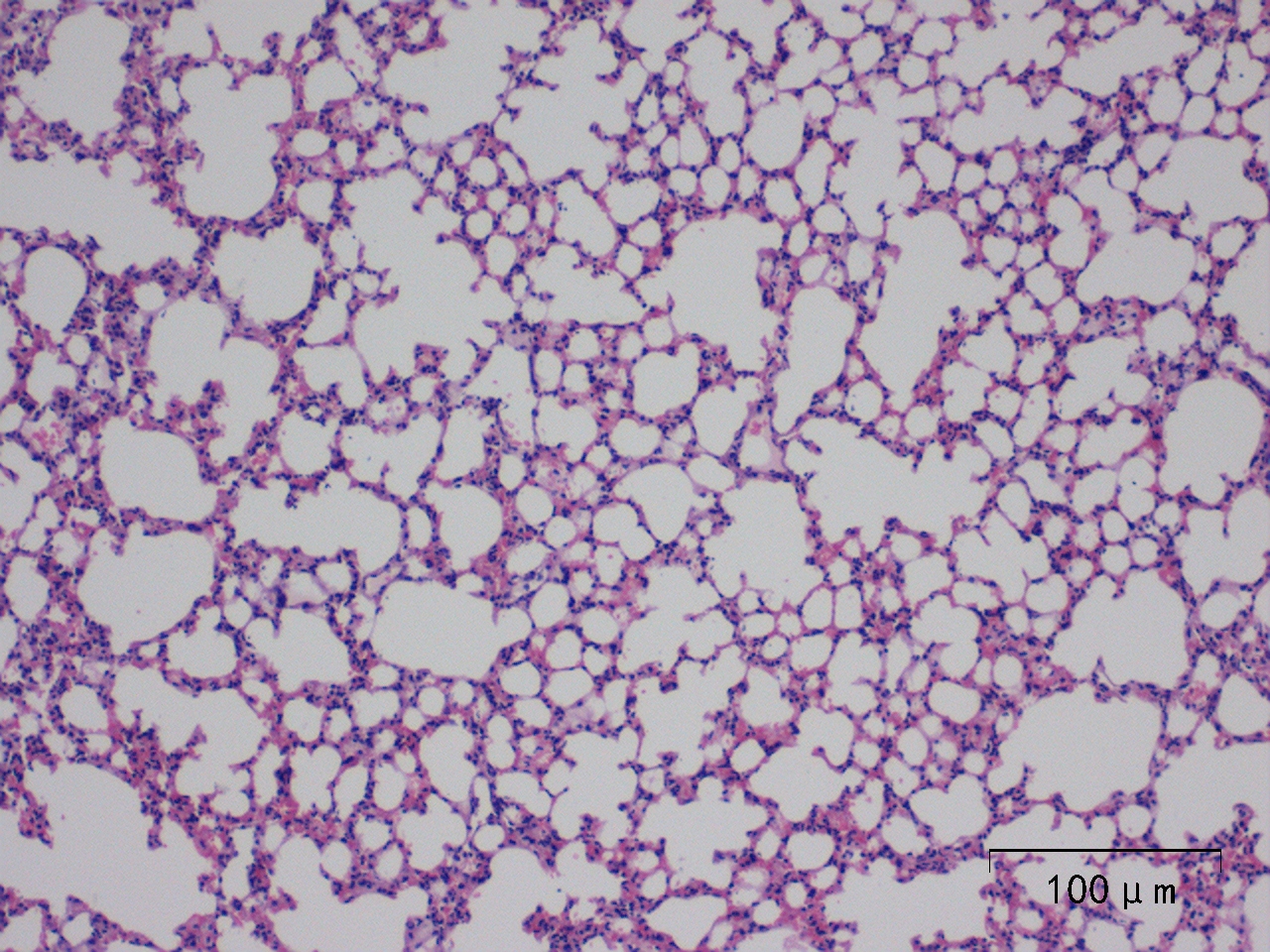

Supplement: S5 Data — (ZIP) [file ppat.1012546.s009.zip › Figure S1-4/FigS4/J/PRV-UL4mut-Lung/47.jpg]

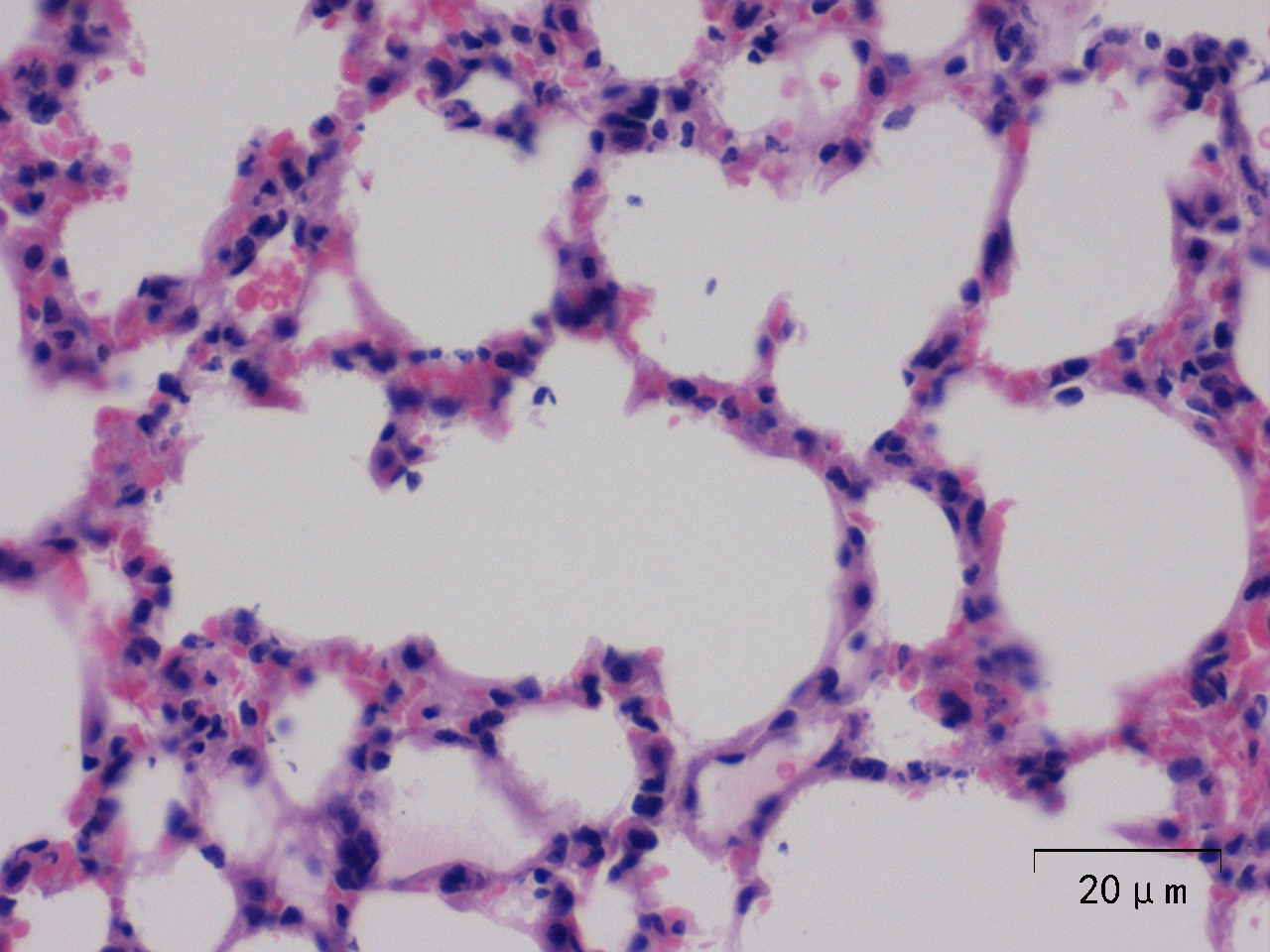

Supplement: S5 Data — (ZIP) [file ppat.1012546.s009.zip › Figure S1-4/FigS4/J/PRV-UL4mut-Lung/48.jpg]
